# Global, regional, and national burden of diseases and injuries for adults 70 years and older: systematic analysis for the Global Burden of Disease 2019 Study

**DOI:** 10.1136/bmj-2021-068208

**Published:** 2022-03-10

**Authors:** GBD 2019 Ageing Collaborators, Stefanos Tyrovolas, Andy Stergachis, Varsha Sarah Krish, Angela Y Chang, Vegard Skirbekk, Joseph L Dieleman, Somnath Chatterji, Foad Abd-Allah, Mohammad Abdollahi, Aidin Abedi, Hassan Abolhassani, Akine Eshete Abosetugn, Lucas Guimarães Abreu, Michael R M Abrigo, Abdulaziz Khalid Abu Haimed, Maryam Adabi, Oladimeji M Adebayo, Isaac Akinkunmi Adedeji, Victor Adekanmbi, Olatunji O Adetokunboh, Davoud Adham, Shailesh M Advani, Mohsen Afarideh, Gina Agarwal, Mohammad Aghaali, Seyed Mohammad Kazem Aghamir, Anurag Agrawal, Sohail Ahmad, Tauseef Ahmad, Keivan Ahmadi, Mehdi Ahmadi, Muktar Beshir Ahmed, Rufus Olusola Akinyemi, Ziyad Al-Aly, Khurshid Alam, Fahad Mashhour Alanezi, Turki M Alanzi, Jacqueline Elizabeth Alcalde-Rabanal, Biresaw Wassihun Alemu, Samar Al-Hajj, Robert Kaba Alhassan, Saqib Ali, Gianfranco Alicandro, Mehran Alijanzadeh, Vahid Alipour, Syed Mohamed Aljunid, François Alla, Majid Abdulrahman Hamad Almadi, Amir Almasi-Hashiani, Abdulaziz M Almulhim, Rajaa M Al-Raddadi, Arya Aminorroaya, Fatemeh Amiri, Arianna Maever L Amit, Dickson A Amugsi, Etsay Woldu Anbesu, Robert Ancuceanu, Deanna Anderlini, Tudorel Andrei, Catalina Liliana Andrei, Sofia Androudi, Mina Anjomshoa, Fereshteh Ansari, Alireza Ansari-Moghaddam, Carl Abelardo T Antonio, Benny Antony, Davood Anvari, Razique Anwer, Jalal Arabloo, Morteza Arab-Zozani, Johan Ärnlöv, Malke Asaad, Mehran Asadi-Aliabadi, Ali A Asadi-Pooya, Maha Moh'd Wahbi Atout, Marcel Ausloos, Floriane Ausloos, Beatriz Paulina Ayala Quintanilla, Getinet Ayano, Martin Amogre Ayanore, Yared Asmare Aynalem, Samad Azari, Zelalem Nigussie Azene, Ebrahim Babaee, Ashish D Badiye, Arun Balachandran, Maciej Banach, Srikanta K Banerjee, Palash Chandra Banik, Suzanne Lyn Barker-Collo, Sanjay Basu, Bernhard T Baune, Mohsen Bayati, Bayisa Abdissa Baye, Neeraj Bedi, Ettore Beghi, Yannick Béjot, Michelle L Bell, Isabela M Bensenor, Akshaya Srikanth Bhagavathula, Pankaj Bhardwaj, Krittika Bhattacharyya, Suraj Bhattarai, Zulfiqar A Bhutta, Sadia Bibi, Ali Bijani, Boris Bikbov, Antonio Biondi, Binyam Minuye Birihane, Atanu Biswas, Tone Bjørge, Somayeh Bohlouli, Srinivasa Rao Bolla, Archith Boloor, Soufiane Boufous, Dejana Braithwaite, Hermann Brenner, Andrew M Briggs, Nikolay Ivanovich Briko, Gabrielle B Britton, Sharath Burugina Nagaraja, Reinhard Busse, Zahid A Butt, Florentino Luciano Caetano dos Santos, Luis Alberto Cámera, Josip Car, Rosario Cárdenas, Giulia Carreras, Juan J Carrero, Felix Carvalho, Joao Mauricio Castaldelli-Maia, Carlos A Castañeda-Orjuela, Giulio Castelpietra, Franz Castro, Ester Cerin, Muge Cevik, Joht Singh Chandan, Alex R Chang, Jaykaran Charan, Vijay Kumar Chattu, Pankaj Chaturvedi, Sarika Chaturvedi, Prachi P Chavan, Simiao Chen, Nicolas Cherbuin, Ken Lee Chin, Daniel Youngwhan Cho, Mohiuddin Ahsanul Kabir Chowdhury, Dinh-Toi Chu, Sheng-Chia Chung, Michael T Chung, Liliana G Ciobanu, Vera Marisa Costa, Ewerton Cousin, Michael H Criqui, Marita Cross, Saad M A Dahlawi, Giovanni Damiani, Lalit Dandona, Rakhi Dandona, Parnaz Daneshpajouhnejad, Jai K Das, Rajat Das Gupta, Claudio Alberto Dávila-Cervantes, Kairat Davletov, Farah Deeba, Diego De Leo, Edgar Denova-Gutiérrez, Nikolaos Dervenis, Rupak Desai, Samath Dhamminda Dharmaratne, Govinda Prasad Dhungana, Mostafa Dianatinasab, Diana Dias da Silva, Daniel Diaz, Martin Dichgans, Shirin Djalalinia, Klara Dokova, Fariba Dorostkar, Leila Doshmangir, Bruce B Duncan, Andre Rodrigues Duraes, Arielle Wilder Eagan, Mohammad Ebrahimi Kalan, David Edvardsson, Andem Effiong, Joshua R Ehrlich, Islam Y Elgendy, Shaimaa I El-Jaafary, Iman El Sayed, Maysaa El Sayed Zaki, Maha El Tantawi, Babak Eshrati, Khalil Eskandari, Sharareh Eskandarieh, Saman Esmaeilnejad, Atkilt Esaiyas Etisso, Pawan Sirwan Faris, Andre Faro, Farshad Farzadfar, Mehdi Fazlzadeh, Valery L Feigin, Seyed-Mohammad Fereshtehnejad, Eduarda Fernandes, Pietro Ferrara, Manuela L Ferreira, Irina Filip, Florian Fischer, James L Fisher, Nataliya A Foigt, Morenike Oluwatoyin Folayan, Artem Alekseevich Fomenkov, Masoud Foroutan, Richard Charles Franklin, Marisa Freitas, Mohamed M Gad, Silvano Gallus, Amiran Gamkrelidze, Abadi Kahsu Gebre, Leake G Gebremeskel, Fatemeh Ghaffarifar, Mansour Ghafourifard, Mahsa Ghajarzadeh, Reza Ghanei Gheshlagh, Ahmad Ghashghaee, Nermin Ghith, Asadollah Gholamian, Tiffany K Gill, Richard F Gillum, Iago Giné-Vázquez, Giorgia Giussani, Mustefa Glagn, Elena V Gnedovskaya, Myron Anthony Godinho, Salime Goharinezhad, Sameer Vali Gopalani, Giuseppe Gorini, Alessandra C Goulart, Michal Grivna, Harish Chander Gugnani, Rafael Alves Guimarães, Yuming Guo, Rahul Gupta, Reyna Alma Gutiérrez, Abdul Hafiz, Arvin Haj-Mirzaian, Arya Haj-Mirzaian, Randah R Hamadeh, Samer Hamidi, Graeme J Hankey, Hamidreza Haririan, Josep Maria Haro, Ahmed I Hasaballah, Maryam Hashemian, Abdiwahab Hashi, Shoaib Hassan, Amr Hassan, Soheil Hassanipour, Simon I Hay, Khezar Hayat, Reza Heidari-Soureshjani, Delia Hendrie, Claudiu Herteliu, Hung Chak Ho, Chi Linh Hoang, Ramesh Holla, Praveen Hoogar, Naznin Hossain, Mostafa Hosseini, Mehdi Hosseinzadeh, Mihaela Hostiuc, Sorin Hostiuc, Mowafa Househ, Mohamed Hsairi, Guoqing Hu, Ayesha Humayun, Bing-Fang Hwang, Ivo Iavicoli, Segun Emmanuel Ibitoye, Olayinka Stephen Ilesanmi, Irena M Ilic, Milena D Ilic, Leeberk Raja Inbaraj, Usman Iqbal, Seyed Sina Naghibi Irvani, Md.Mohaimenul Islam, Hiroyasu Iso, Rebecca Q Ivers, Chinwe Juliana Iwu, Chidozie C D Iwu, Jalil Jaafari, Mohammad Ali Jahani, Mihajlo Jakovljevic, Farzad Jalilian, Hosna Janjani, Manthan Dilipkumar Janodia, Tahereh Javaheri, Ensiyeh Jenabi, Ravi Prakash Jha, John S Ji, Oommen John, Jost B Jonas, Jacek Jerzy Jozwiak, Mikk Jürisson, Ali Kabir, Zubair Kabir, Rizwan Kalani, Rohollah Kalhor, Tanuj Kanchan, Neeti Kapoor, Behzad Karami Matin, André Karch, Ayele Semachew Kasa, Gbenga A Kayode, Ali Kazemi Karyani, Yousef Saleh Khader, Nauman Khalid, Mohammad Khammarnia, Maseer Khan, Ejaz Ahmad Khan, Khaled Khatab, Mahalaqua Nazli Khatib, Maryam Khayamzadeh, Habibolah Khazaie, Abdullah T Khoja, Young-Eun Kim, Yun Jin Kim, Ruth W Kimokoti, Sezer Kisa, Adnan Kisa, Mika Kivimäki, Cameron J Kneib, Sonali Kochhar, Ali Koolivand, Jacek A Kopec, Anirudh Kotlo, Ai Koyanagi, Kewal Krishan, Barthelemy Kuate Defo, G Anil Kumar, Nithin Kumar, Manasi Kumar, Om P Kurmi, Dian Kusuma, Ben Lacey, Dharmesh Kumar Lal, Ratilal Lalloo, Tea Lallukka, Jennifer O Lam, Faris Hasan Lami, Iván Landires, Van Charles Lansingh, Anders O Larsson, Savita Lasrado, Paolo Lauriola, Carlo La Vecchia, Janet L Leasher, Georgy Lebedev, Paul H Lee, Shaun Wen Huey Lee, James Leigh, Matilde Leonardi, Andrew S Levey, Miriam Levi, Shanshan Li, Shai Linn, Xuefeng Liu, Alan D Lopez, Platon D Lopukhov, Stefan Lorkowski, Paulo A Lotufo, Rafael Lozano, Alessandra Lugo, Raimundas Lunevicius, Mohammed Madadin, Ralph Maddison, Phetole Walter Mahasha, Morteza Mahmoudi, Azeem Majeed, Shokofeh Maleki, Afshin Maleki, Reza Malekzadeh, Deborah Carvalho Malta, Abdullah A Mamun, Navid Manafi, Fariborz Mansour-Ghanaei, Borhan Mansouri, Mohammad Ali Mansournia, Santi Martini, Francisco Rogerlândio Martins-Melo, Seyedeh Zahra Masoumi, João Massano, Pallab K Maulik, Mohsen Mazidi, John J McGrath, Martin McKee, Man Mohan Mehndiratta, Fereshteh Mehri, Fabiola Mejia-Rodriguez, Walter Mendoza, Ritesh G Menezes, George A Mensah, Alibek Mereke, Tuomo J Meretoja, Atte Meretoja, Tomislav Mestrovic, Tomasz Miazgowski, Irmina Maria Michalek, Ted R Miller, Edward J Mills, Andreea Mirica, Erkin M Mirrakhimov, Hamed Mirzaei, Maryam Mirzaei, Mehdi Mirzaei-Alavijeh, Philip B Mitchell, Babak Moazen, Masoud Moghadaszadeh, Efat Mohamadi, Yousef Mohammad, Dara K Mohammad, Shadieh Mohammadi, Abdollah Mohammadian-Hafshejani, Shafiu Mohammed, Ali H Mokdad, Alex Molassiotis, Natalie C Momen, Stefania Mondello, Masoud Moradi, Maziar Moradi-Lakeh, Farhad Moradpour, Rahmatollah Moradzadeh, Paula Moraga, Lidia Morawska, Rintaro Mori, Seyyed Meysam Mousavi, Amin Mousavi Khaneghah, Ulrich Otto Mueller, Satinath Mukhopadhyay, Moses K Muriithi, Mehdi Naderi, Ahamarshan Jayaraman Nagarajan, Behshad Naghshtabrizi, Mukhammad David Naimzada, Farid Najafi, Jobert Richie Nansseu, Rawlance Ndejjo, Ionut Negoi, Ruxandra Irina Negoi, Subas Neupane, Georges Nguefack-Tsague, Josephine W Ngunjiri, Cuong Tat Nguyen, Huong Lan Thi Nguyen, Rajan Nikbakhsh, Chukwudi A Nnaji, Marzieh Nojomi, Shuhei Nomura, Bo Norrving, Jean Jacques Noubiap, Christoph Nowak, Virginia Nuñez-Samudio, Felix Akpojene Ogbo, In-Hwan Oh, Morteza Oladnabi, Andrew T Olagunju, Jacob Olusegun Olusanya, Bolajoko Olubukunola Olusanya, Muktar Omer Omer, Obinna E Onwujekwe, Sergej M Ostojic, Adrian Oțoiu, Nikita Otstavnov, Stanislav S Otstavnov, Mayowa O Owolabi, Mahesh P A, Jagadish Rao Padubidri, Songhomitra Panda-Jonas, Anamika Pandey, Carlo Irwin Able Panelo, Deepak Kumar Pasupula, Hamidreza Pazoki Toroudi, Jonathan Pearson-Stuttard, Amy E Peden, Veincent Christian Filipino Pepito, Emmanuel K Peprah, Jeevan Pereira, Konrad Pesudovs, Hai Quang Pham, Michael R Phillips, Thomas Pilgrim, Marina Pinheiro, Michael A Piradov, Meghdad Pirsaheb, Roman V Polibin, Suzanne Polinder, Maarten J Postma, Hadi Pourjafar, Akram Pourshams, Sergio I Prada, Sanjay Prakash, Dimas Ria Angga Pribadi, Elisabetta Pupillo, Zahiruddin Quazi Syed, Navid Rabiee, Amir Radfar, Ata Rafiee, Alberto Raggi, Fakher Rahim, Vafa Rahimi-Movaghar, Mohammad Hifz Ur Rahman, Muhammad Aziz Rahman, Kiana Ramezanzadeh, Chhabi Lal Ranabhat, Annemarei Ranta, Sowmya J Rao, Vahid Rashedi, Prateek Rastogi, Priya Rathi, Salman Rawaf, David Laith Rawaf, Lal Rawal, Reza Rawassizadeh, Andre M N Renzaho, Bhageerathy Reshmi, Serge Resnikoff, Nima Rezaei, Negar Rezaei, Aziz Rezapour, Seyed Mohammad Riahi, Daniela Ribeiro, Ana Isabel Ribeiro, Jennifer Rickard, Leonardo Roever, Michele Romoli, Dietrich Rothenbacher, Enrico Rubagotti, Susan Fred Rumisha, Seyedmohammad Saadatagah, Siamak Sabour, Perminder S Sachdev, Masoumeh Sadeghi, Ehsan Sadeghi, Sahar Saeedi Moghaddam, Rajesh Sagar, Mohammad Ali Sahraian, S. Mohammad Sajadi, Nasir Salam, Marwa Rashad Salem, Hamideh Salimzadeh, Omar Mukhtar Salman, Abdallah M Samy, Juan Sanabria, Lidia Sanchez Riera, Itamar S Santos, Milena M Santric-Milicevic, Sivan Yegnanarayana Iyer Saraswathy, Arash Sarveazad, Brijesh Sathian, Thirunavukkarasu Sathish, Davide Sattin, Silvia Schiavolin, Maria Inês Schmidt, Aletta Elisabeth Schutte, David C Schwebel, Falk Schwendicke, Subramanian Senthilkumaran, Sadaf G Sepanlou, Feng Sha, Omid Shafaat, Saeed Shahabi, Amira A Shaheen, Masood Ali Shaikh, Marina Shakhnazarova, Mehran Shams-Beyranvand, MohammadBagher Shamsi, Morteza Shamsizadeh, Kiomars Sharafi, B Suresh Kumar Shetty, Kenji Shibuya, Wondimeneh Shibabaw Shiferaw, Mika Shigematsu, Jae Il Shin, Rahman Shiri, Reza Shirkoohi, Kerem Shuval, Inga Dora Sigfusdottir, Rannveig Sigurvinsdottir, João Pedro Silva, Biagio Simonetti, Jasvinder A Singh, Pushpendra Singh, Ambrish Singh, Dhirendra Narain Sinha, Søren T Skou, Valentin Yurievich Skryabin, Emma U R Smith, Mohammad Reza Sobhiyeh, Amin Soheili, Shahin Soltani, Joan B Soriano, Ireneous N Soyiri, Emma Elizabeth Spurlock, Chandrashekhar T Sreeramareddy, Dan J Stein, Leo Stockfelt, Mark A Stokes, Saverio Stranges, Jacob L Stubbs, Agus Sudaryanto, Mu'awiyyah Babale Sufiyan, Hafiz Ansar Rasul Suleria, Rizwan Suliankatchi Abdulkader, Gerhard Sulo, Rafael Tabarés-Seisdedos, Takahiro Tabuchi, Biruk Wogayehu Taddele, Amir Taherkhani, Masih Tajdini, Md Ismail Tareque, Yonas Getaye Tefera, Mohamad-Hani Temsah, Zemenu Tadesse Tessema, Kavumpurathu Raman Thankappan, Rekha Thapar, Amanda G Thrift, Mariya Vladimirovna Titova, Hamid Reza Tohidinik, Marcello Tonelli, Mathilde Touvier, Marcos Roberto Tovani-Palone, Bach Xuan Tran, Ravensara S Travillian, Alexander C Tsai, Aristidis Tsatsakis, Riaz Uddin, Saif Ullah, Chukwuma David Umeokonkwo, Bhaskaran Unnikrishnan, Marco Vacante, Pascual R Valdez, Aaron van Donkelaar, Santosh Varughese, Tommi Juhani Vasankari, Yasser Vasseghian, Yousef Veisani, Narayanaswamy Venketasubramanian, Francesco S Violante, Vasily Vlassov, Yasir Waheed, Yanzhong Wang, Fang Wang, Yuan-Pang Wang, Jingkai Wei, Robert G Weintraub, Jordan Weiss, Ronny Westerman, Taweewat Wiangkham, Charles D A Wolfe, Ai-Min Wu, Seyed Hossein Yahyazadeh Jabbari, Kazumasa Yamagishi, Yuichiro Yano, Sanni Yaya, Vahid Yazdi-Feyzabadi, Yordanos Gizachew Yeshitila, Mohammed Zewdu Yimmer, Paul Yip, Naohiro Yonemoto, Seok-Jun Yoon, Mustafa Z Younis, Zabihollah Yousefi, Taraneh Yousefinezhadi, Chuanhua Yu, Yong Yu, Hasan Yusefzadeh, Syed Saoud Zaidi, Sojib Bin Zaman, Maryam Zamanian, Hadi Zarafshan, Mikhail Sergeevich Zastrozhin, Zhi-Jiang Zhang, Jianrong Zhang, Yunquan Zhang, Xiu-Ju George Zhao, Cong Zhu, Georgios A Kotsakis, Nicholas J Kassebaum

## Abstract

**Objectives:**

To use data from the Global Burden of Diseases, Injuries, and Risk Factors Study 2019 (GBD 2019) to estimate mortality and disability trends for the population aged ≥70 and evaluate patterns in causes of death, disability, and risk factors.

**Design:**

Systematic analysis.

**Setting:**

Participants were aged ≥70 from 204 countries and territories, 1990-2019.

**Main outcomes measures:**

Years of life lost, years lived with disability, disability adjusted life years, life expectancy at age 70 (LE-70), healthy life expectancy at age 70 (HALE-70), proportion of years in ill health at age 70 (PYIH-70), risk factors, and data coverage index were estimated based on standardised GBD methods.

**Results:**

Globally the population of older adults has increased since 1990 and all cause death rates have decreased for men and women. However, mortality rates due to falls increased between 1990 and 2019. The probability of death among people aged 70-90 decreased, mainly because of reductions in non-communicable diseases. Globally disability burden was largely driven by functional decline, vision and hearing loss, and symptoms of pain. LE-70 and HALE-70 showed continuous increases since 1990 globally, with certain regional disparities. Globally higher LE-70 resulted in higher HALE-70 and slightly increased PYIH-70. Sociodemographic and healthcare access and quality indices were positively correlated with HALE-70 and LE-70. For high exposure risk factors, data coverage was moderate, while limited data were available for various dietary, environmental or occupational, and metabolic risks.

**Conclusions:**

Life expectancy at age 70 has continued to rise globally, mostly because of decreases in chronic diseases. Adults aged ≥70 living in high income countries and regions with better healthcare access and quality were found to experience the highest life expectancy and healthy life expectancy. Disability burden, however, remained constant, suggesting the need to enhance public health and intervention programmes to improve wellbeing among older adults.

## Introduction

For the first time in history, most newborns might live into their 70s and beyond.^1^ With the global population experiencing extra years of life, the health and wellbeing of older adults is paramount so that they can continue to be actively engaged in society.^2^ However, if added years are spent in poor health, health systems will face increased healthcare expenses due to increased demand.^3^ To conceptualise years of life spent in good health, a variety of ageing indicators have been developed. Healthy and successful ageing, and frailty, project high or low wellbeing in older people, respectively.^4^ ^5^ ^6^ Ageing research suggests that functional decline and health loss are more reflective of healthy ageing than chronological age.^7^ Consequently, surveillance of the older population’s health is essential to capture its ageing status. Variations in the definition of old age exist that account for chronological age or for remaining life expectancy.^8^ Epidemiological data indicate that population ageing patterns are changing, with people aged ≥70 and ≥90 being the fastest growing segment in Europe, Asia, and the United States.^9^ ^10^ ^11^ In 1950, older adults represented 5% of the global population; this estimate is projected to rise to 16% by 2050.^12^ Consequently, the health and wellbeing of ageing populations have become important public health issues with wide reaching economic implications that affect medical care, in-home care and assistance, and healthcare staff.^13^

The projected ageing demographics are linked to increased burden and duration of non-communicable diseases.^7^ An analysis of the Global Burden of Disease (GBD) 2010 data established that the major causes of disability for adults aged ≥60 were musculoskeletal disorders, cardiovascular diseases, diabetes, and neurological disorders.^14^ In 2015, the World Health Organization declared that the rise in chronic conditions among older adults was a worldwide epidemic.^7^ The management of accumulated chronic conditions is likely to weigh upon healthcare financing over the next few decades in high income and in low to middle income regions.^15^ ^16^ ^17^ Low to middle income regions face an ongoing agenda of communicable diseases and will have to manage the added burden with limited resources and infrastructure. Understanding and reducing the burden of disease among older people is critical to mitigate the economic burden of ageing and build sustainability within the global health system for the next generations.^13^

While ageing draws increasing attention from policy makers and stakeholders, global epidemiological data on the burden of disease in older adults are limited. Studies from high life expectancy populations do exist,^4^ ^18^ ^19^ but most are based on localised sample populations without detailed analyses of adults older than 70.^20^ ^21^ ^22^ Epidemiological studies from WHO,^23^ including the recently published world report on ageing and health, have highlighted an increasingly ageing global population and the need for urgent public health changes.^7^ The GBD 2019 study provides annually updated global, regional, and national population data on mortality, 369 diseases and injuries, and 87 risk factors among 204 countries.^24^ ^25^ ^26^ Therefore, it provides an excellent opportunity for global and regional systematic analysis of causes of fatal and non-fatal health loss and risk factors in older adults.

The overall aim of the present study was to describe levels and trends in death and disability burden in the population aged ≥70 using GBD 2019 data. We approached this with several new metrics and assessments that leverage the GBD 2019 results. These assessments included life expectancy at age 70 (LE-70), the probability of death between ages 70 and 90 (20q70), assessment of diseases and injuries leading to changes in 20q70 through causal decomposition, calculating healthy life expectancy at age 70 (HALE-70), and the proportion of remaining years in ill health at age 70 (PYIH-70). To provide context to these analyses, we further evaluated the historical relation between LE-70, HALE-70, and PYIH-70 with two societal proxies for development: the sociodemographic index (SDI) and the healthcare access and quality (HAQ) index. Data coverage underpinning the GBD estimates was also assessed. Overall, this study provides a comprehensive and detailed assessment of the health of older adults.

## Methods

Extensive details on the methods used to derive each of the measures in GBD 2019 have been published previously.^24^ ^25^ ^26^ A brief summary of each component is presented, with emphasis on the metrics and analyses that are distinct to the present study to evaluate trends in epidemiological patterns and disease burden for people aged ≥70. Reporting was performed with adherence to the guidelines for accurate and transparent health estimates reporting (GATHER) statement (supplementary table 1). Data inputs are downloadable from the Global Health Data Exchange (http://ghdx.healthdata.org/). Results are viewable online in GBD Compare (https://vizhub.healthdata.org/gbd-compare/).

### Dimensions of GBD 2019

GBD 2019 includes estimates for 369 diseases and injuries, and 87 risk factors for 23 age groups and both sexes from 1990 to 2019 covering 204 countries and territories—22 of which were analysed subnationally—hierarchically arranged into 21 regions and 7 super regions (supplementary table 2). SDI is a composite indicator that uses the following components: country level income per capita, average educational attainment among people older than 15 years, and total fertility rate among women younger than 25 years.^24^ SDI ranges from 0 (high fertility, low education, low income) to 1 (low fertility, high income, high education). Each GBD location was assigned to a single SDI group based on its SDI value in 2019.

All deaths were assigned a single underlying cause according to the international classification of diseases, and each was mapped according to a four level mutually exclusive and collectively exhaustive GBD cause list (supplementary table 3). Level 1 differentiates between communicable, maternal, neonatal, and nutritional disorders; non-communicable diseases; and injuries. Level 2 covers 22 disease and injury aggregate clusters, such as cardiovascular diseases, respiratory diseases, and transport related injuries. Some level 2 disorders (maternal disorders, neonatal disorders, and congenital birth defects) do not cause death in adults aged ≥70, but there is burden present for the first two arising from long term sequelae of neonatal disorders (eg, cerebral palsy) and long term complications of pregnancy (eg, severe preeclampsia and eclampsia). Level 3 (174 conditions) and level 4 (301 conditions) causes represent increasingly more specific diseases and injuries. Most causes were estimated as underlying causes of death and causes of disability burden. A few causes were assessed to cause either death or disability, but not both. Examples include aortic aneurysm (death only) and periodontal disease (disability only). The GBD 2019 comparative risk assessment framework classified each of 87 risk factors and clusters of risk factors into one of three categories: behavioural, environmental or occupational, and metabolic.^26^

### All cause mortality and cause specific mortality

Mortality estimation methods have been extensively described elsewhere.^24^ ^25^ Briefly, all available global data including vital registration, sample registration, household surveys, censuses, disease registries, notification systems, and police records were identified, extracted, and standardised. Standardised methods were then applied to produce internally consistent estimates of population, fertility, net migration, all cause mortality, and cause specific mortality. All cause mortality estimates for each location, sex, and year were inputs to the calculation of overall life expectancy and LE-70. Years of life lost were the product of cause specific mortality rates and remaining GBD standard life expectancy at age of death.

### Non-fatal disease burden and disability adjusted life years

Estimation methods for prevalence, incidence, years lived with disability, and disability adjusted life years have been described elsewhere.^25^ Briefly, global datasets were assembled as for the mortality models with the addition of administrative datasets (hospital discharges and insurance claims) and published scientific studies. Data were standardised to a single reference definition using meta-regression—bayesian, regularised, trimmed (MR-BRT), a meta-regression framework developed for GBD 2019.^27^ Most causes used DisMod-MR 2.1, a bayesian meta-regression tool developed for GBD, to generate internally consistent models of prevalence, incidence, remission, and excess mortality. The proportion experiencing each type of non-fatal sequela were then calculated separately and paired with corresponding global GBD disability weights derived from worldwide population surveys to calculate years of life lived with disability.^28^ Finally, a microsimulation adjusted for years lived with disability to account for comorbidity. The simulation assumed independent comorbidity between different diseases but was run separately for each age group, sex, location, and year based on extensive testing during GBD 2010, which revealed age and sex explained most comorbidities.^29^ Disability adjusted life years were the sum of years of life lost and years lived with disability.

### Specialised metrics of ageing

We calculated the probability of death between ages 70 and 90 (20q70) and paired this with cause specific mortality results in a decomposition analysis to understand how trends in 20q70 between 1990 and 2019 related to temporal trends in specific GBD causes. Next, we used previously described methods^24^ to calculate HALE-70, evaluating age specific mortality and years lived with disability rates for each age group. Finally, we calculated PYIH-70: (LE-70−HALE-70)/LE-70.

### Risk factor exposure and attributable burden

Risk factor exposure, relative risk, and population attributable fraction estimation methods have been extensively described previously.^26^ Briefly, exposure models drew on similar data sources as non-fatal estimates. A continuous distribution of exposure was estimated for several risk factors (eg, high body mass index) known to have a spectrum of associated severity and outcome using ensemble distribution methods developed for GBD. For risk factors, exposure data were modelled by applying either spatiotemporal Gaussian process regression or DisMod-MR 2.1, bayesian statistical models.^24^ ^26^ Quantitative relative risks were estimated for each risk-outcome pair, then paired with corresponding exposure estimates to calculate the population attributable fraction for each risk-outcome pair. Population attributable fractions were multiplied by the outcome rates to calculate attributable years lived with disability, years of life lost, and disability adjusted life years.

Summary exposure value is a measure ranging from 0 (lowest) to 1 (highest) developed for GBD to capture exposure, magnitude of relative risk, and attributable burden in a single number. Summary exposure values allow comparison of intensity of exposure from the perspective of adverse health outcomes across risk factors and across different demographical groups. We evaluated the relation between total attributable disability adjusted life years to each risk factor in the population aged ≥70 and the annualised rate of change in summary exposure values from 1990 to 2019 in those aged ≥70. The annualised rate of change was calculated as log transformed (final estimates/initial estimates)/(No of years).

### Epidemiological transition: historical relation with SDI and HAQ index

We assessed the epidemiological transition in ageing metrics (LE-70, HALE-70, and PYIH-70) as a function of summary measures of societal development and health system performance, including SDI and HAQ index. The HAQ index is a composite metric developed for GBD 2016 and subsequently updated. It is based on comparative risk standardised mortality rates for healthcare sensitive diseases, ranges from 0 (worst) to 100 (best), and represents a health centric assessment of development to complement SDI.^30^ For each metric, we incorporated all observed location specific estimates from 1990 to 2019 in MR-BRT,^27^ including an intercept and a cubic spline on either SDI or HAQ index, depending on the model version, to predict the historical average relation between them. Models were fit in log space and an offset of 1×10^−7^ was used. The observed value for each location year was compared with the expected value, which was the result of the spline model, to calculate the observed to expected ratio.

### Uncertainty and data coverage index for population aged ≥70

For all results, we report 95% uncertainty intervals derived from 1000 draws from the posterior distribution of each step in the estimation process according to established GBD methods.

The geographical and temporal representativeness of the data sources for non-fatal health outcomes were estimated through a measure we term the data coverage index (DCI). DCI was calculated in two ways for non-fatal disease burden and risk factor exposure. Firstly, we calculated the proportion of countries and territories with input data for each level 3 cause or risk factor (DCI by cause or risk). Secondly, we calculated the proportion of cause or risk factor with any input data for each country and territory (DCI by country). We compared the DCI for all ages with that for the population aged ≥70 only to highlight potential data gaps.

### Patient and public involvement

The GBD study is a global collaborative scientific endeavour involving more than 7500 people from around 150 countries. Enrolment as a GBD collaborator is a public facing process without specific limitations placed on educational degree or professional status. All collaborators were invited to review and comment on the manuscript according to their personal involvement and expertise.

## Results

### Demographics, mortality, and morbidity trends

**Fig 1:**
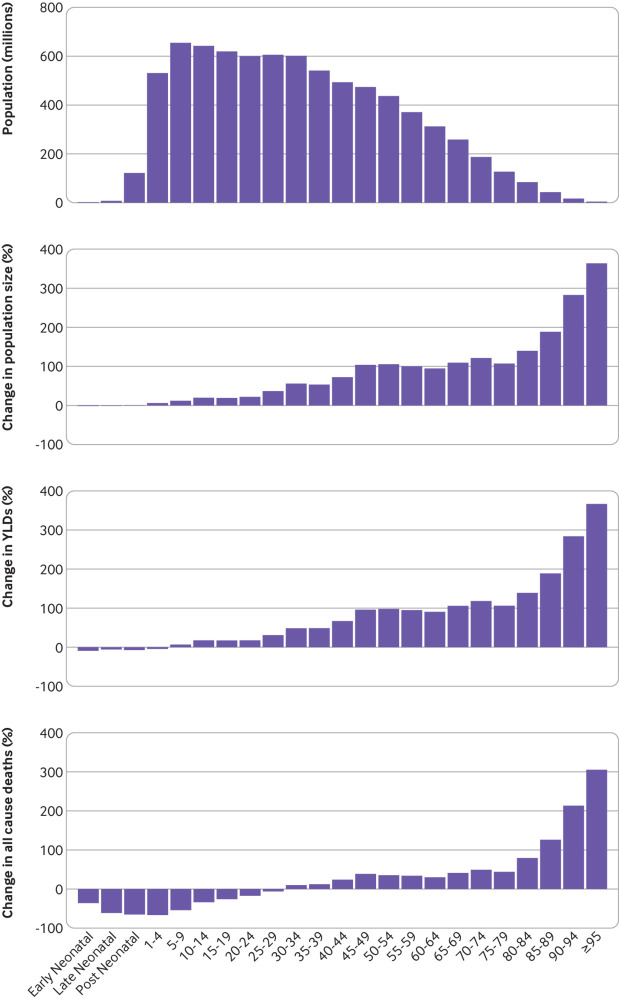
Global population, years lived with disability, and all-cause mortality transition by age group, 1990-2019. Distribution of global population by age group was estimated as simple difference from 1990 to 2019 (top panel), or as percentage change during the same period (second panel). Percentage differences in years lived with disability and all cause mortality as estimated by GBD 2019 are also provided for all age groups from 1990 to 2019 (lower two panels) to indicate quality of life lost due to illness before death and to quantify all cause mortality. All age groups have been included to provide a comparator when assessing health loss in older adults. GBD=Global Burden of Diseases, Injuries, and Risk Factors Study; YLD=years lived with disability

From 1990 to 2019, the size of the global population aged ≥70 increased (fig 1). The 70−79 year old age group grew 115.4%, while the proportion of adults aged 80-94 increased by 164.7%. The population aged ≥95 grew by 363.7%. These trends were consistent across all SDI groups and GBD super regions (supplementary figs 1-12). In 2019, there were 168.3 million more people aged 70-79, 90.1 million more people aged 80-94, and 3.7 million more people aged ≥95 than in 1990. Globally all cause deaths increased while death rates decreased for men and women aged ≥70 between 1990 and 2019 (supplementary table 4), a pattern that was followed for nearly all specific causes of death. Globally during the same period, rates for years lived with disability increased only slightly in people aged ≥70 (+0.7%, 95% uncertainty interval 0.0% to 1.4%) and in all SDI groups. Globally all cause years lived with disability rates decreased for men but increased for women aged ≥70 between 1990 and 2019 (supplementary table 5).

### LE-70, HALE-70, and PYIH-70

**Fig 2:**
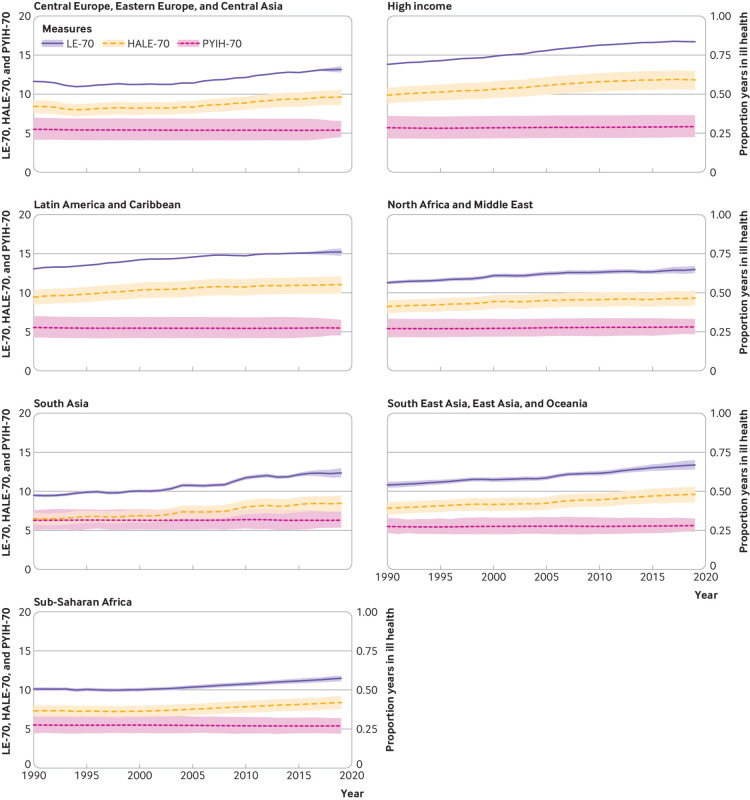
Life expectancy at age 70 (LE-70), healthy life expectancy at age 70 (HALE-70), and proportion of life years spent in ill health at age 70 (PYIH-70) by location for both sexes, 1990-2019. Shaded sections indicate 95% uncertainty intervals. GBD=Global Burden of Diseases, Injuries, and Risk Factors Study

**Fig 3:**
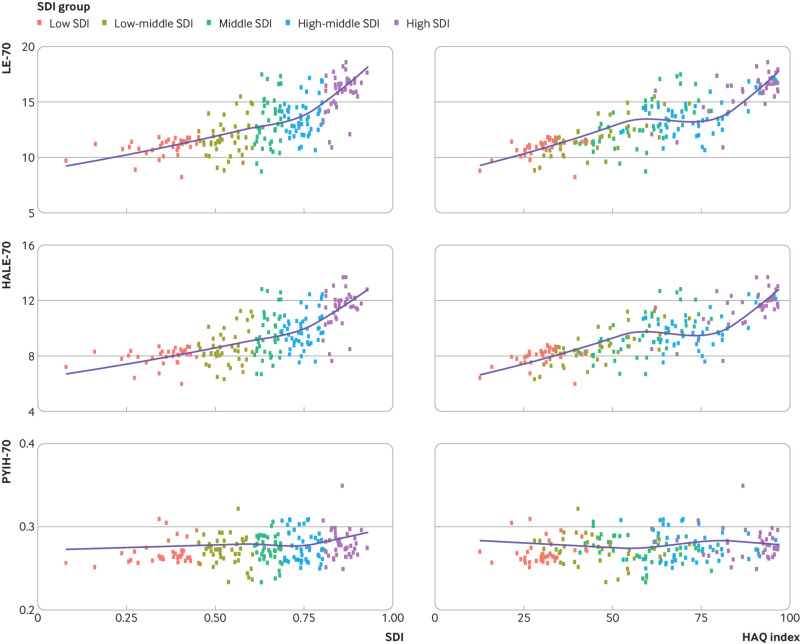
Epidemiological transition between life expectancy at age 70 (LE-70), healthy life expectancy at age 70 (HALE-70), proportion of years spent in ill health at age 70 (PYIH-70), and sociodemographic index (SDI) and healthcare access and quality (HAQ) index for both sexes, 2019. Dots represent countries and different colour coding indicates SDI categorisation

Globally LE-70 increased for men and women in 2019 (men: 12.88, 95% uncertainty level 12.53 to 13.26; women: 15.21, 14.88 to 15.55) compared with 1990 (men: 10.60, 10.43 to 10.80; women: 12.82, 12.68 to 12.99; table 1, supplementary tables 6-8). Globally HALE-70 increased for men (from 7.72 (6.92 to 8.42) healthy years in 1990 to 9.35 (8.43 to 10.27) in 2019) and women (from 9.10 (8.12 to 10.0) healthy years in 1990 to 10.69 (9.52 to 11.76) in 2019). Improvements in HALE-70 were slightly faster than LE-70 globally, which equated to a minimal increase in PYIH-70. All super regions saw improvements in LE-70, higher HALE-70, and a relatively stagnated PYIH-70 (fig 2, supplementary figs 13 and 14 by sex). We found associations between SDI, HAQ index, and each of LE-70, HALE-70, and PYIH-70 (fig 3; supplementary figs 15 and 16 by sex). Low regional variability was noted in LE-70, HALE-70, and PYIH-70 within countries of the same regional clusters.

**Table 1:** Life expectancy, healthy life expectancy, and proportion of years spent in ill health for the population 70 years and older by sociodemographic index and location for both sexes in 1990, 2005, and 2019

	LE-70					HALE-70						PYIH-70			
	1990	2005	2019				1990	2005	2019		1990		2005	2019
**Global**	11.8 (11.7 to 12)	12.8 (12.7 to 12.9)	14.1 (13.9 to 14.4)				8.48 (7.57 to 9.29)	9.18 (8.24 to 10)	10.1 (9.01 to 11)		0.28 (0.22 to 0.35)		0.28 (0.22 to 0.35)	0.29 (0.23 to 0.35)
Low SDI	9.53 (9.34 to 9.74)	10.3 (10.1 to 10.5)	11.5 (11.2 to 11.8)				6.79 (6.07 to 7.48)	7.32 (6.55 to 8.07)	8.2 (7.31 to 9.04)		0.29 (0.23 to 0.35)		0.29 (0.23 to 0.35)	0.29 (0.24 to 0.35)
Low‐middle SDI	10 (9.88 to 10.2)	11.1 (10.9 to 11.2)	12.3 (11.9 to 12.7)				7.09 (6.33 to 7.81)	7.8 (6.94 to 8.57)	8.62 (7.66 to 9.55)		0.29 (0.24 to 0.36)		0.3 (0.24 to 0.36)	0.3 (0.25 to 0.36)
Middle SDI	11.2 (10.9 to 11.5)	11.9 (11.7 to 12.1)	13.3 (12.8 to 13.7)				8.06 (7.19 to 8.83)	8.59 (7.72 to 9.39)	9.49 (8.54 to 10.4)		0.28 (0.23 to 0.34)		0.28 (0.22 to 0.34)	0.28 (0.24 to 0.34)
High‐middle SDI	11.8 (11.7 to 12)	12.6 (12.5 to 12.7)	14.5 (14.1 to 14.8)				8.55 (7.66 to 9.35)	9.14 (8.21 to 9.97)	10.4 (9.38 to 11.4)		0.28 (0.22 to 0.34)		0.28 (0.22 to 0.34)	0.28 (0.23 to 0.34)
High SDI	13.8 (13.7 to 13.8)	15.5 (15.5 to 15.5)	16.7 (16.6 to 16.8)				9.81 (8.76 to 10.8)	11.1 (9.87 to 12.1)	11.8 (10.5 to 13)		0.29 (0.22 to 0.36)		0.29 (0.22 to 0.36)	0.29 (0.23 to 0.37)
**Central Europe, Eastern Europe, and Central Asia**	11.6 (11.6 to 11.6)	11.4 (11.4 to 11.4)	13.2 (12.8 to 13.6)				8.43 (7.55 to 9.22)	8.34 (7.48 to 9.1)	9.62 (8.59 to 10.5)		0.27 (0.21 to 0.35)		0.27 (0.2 to 0.34)	0.27 (0.23 to 0.33)
Central Asia	12.1 (12 to 12.2)	9.84 (9.76 to 9.93)	11 (10.6 to 11.4)				9.1 (8.27 to 9.86)	7.46 (6.8 to 8.07)	8.26 (7.49 to 9.04)		0.25 (0.19 to 0.31)		0.24 (0.19 to 0.3)	0.25 (0.21 to 0.29)
Central Europe	11.3 (11.2 to 11.3)	12.4 (12.4 to 12.4)	13.9 (13.2 to 14.6)				8.15 (7.3 to 8.9)	9.03 (8.1 to 9.85)	10.1 (8.97 to 11.2)		0.28 (0.21 to 0.35)		0.27 (0.21 to 0.34)	0.27 (0.23 to 0.32)
Eastern Europe	11.8 (11.7 to 11.8)	11.1 (11.1 to 11.1)	13.3 (12.8 to 13.9)				8.49 (7.59 to 9.29)	8.09 (7.24 to 8.83)	9.73 (8.62 to 10.7)		0.28 (0.21 to 0.35)		0.27 (0.21 to 0.35)	0.27 (0.22 to 0.33)
**High income**	13.8 (13.8 to 13.8)	15.6 (15.6 to 15.6)	16.7 (16.7 to 16.7)				9.87 (8.82 to 10.8)	11.1 (9.92 to 12.2)	11.8 (10.6 to 13)		0.29 (0.22 to 0.36)		0.29 (0.22 to 0.36)	0.29 (0.22 to 0.37)
Australasia	13.8 (13.7 to 13.8)	16.1 (16 to 16.1)	17.1 (17 to 17.2)				9.8 (8.74 to 10.8)	11.3 (10.1 to 12.5)	12 (10.6 to 13.2)		0.29 (0.22 to 0.36)		0.3 (0.23 to 0.37)	0.3 (0.23 to 0.37)
High income Asia Pacific	14.4 (14.4 to 14.4)	16.9 (16.9 to 16.9)	18.3 (18.3 to 18.4)				10.6 (9.53 to 11.5)	12.4 (11.2 to 13.5)	13.5 (12.2 to 14.7)		0.27 (0.2 to 0.34)		0.27 (0.2 to 0.34)	0.26 (0.2 to 0.33)
High income North America	14.3 (14.3 to 14.3)	15.2 (15.2 to 15.2)	16 (16 to 16.1)				9.72 (8.55 to 10.8)	10.3 (9.05 to 11.4)	10.5 (9.18 to 11.8)		0.32 (0.24 to 0.4)		0.32 (0.25 to 0.4)	0.34 (0.27 to 0.43)
Southern Latin America	12.8 (12.8 to 12.8)	14 (13.9 to 14)	14.6 (14.5 to 14.8)				9.56 (8.65 to 10.4)	10.4 (9.37 to 11.3)	10.8 (9.73 to 11.8)		0.25 (0.19 to 0.32)		0.26 (0.19 to 0.33)	0.26 (0.2 to 0.33)
Western Europe	13.5 (13.4 to 13.5)	15.4 (15.4 to 15.4)	16.5 (16.5 to 16.6)				9.81 (8.82 to 10.7)	11.2 (10 to 12.2)	12 (10.8 to 13.1)		0.27 (0.21 to 0.34)		0.27 (0.21 to 0.35)	0.28 (0.21 to 0.35)
**Latin America and Caribbean**	13.1 (13 to 13.1)	14.6 (14.5 to 14.6)	15.2 (14.7 to 15.7)				9.44 (8.45 to 10.3)	10.6 (9.51 to 11.6)	11 (9.91 to 12.1)		0.28 (0.21 to 0.35)		0.27 (0.21 to 0.34)	0.27 (0.23 to 0.33)
Andean Latin America	13.5 (13.1 to 13.9)	14.8 (14.4 to 15.2)	15.4 (1 4.4 to 1 6.5)				10.1 (9.05 to 11)	11 (9.89 to 12)	11.4 (10.1 to 12.7)		0.25 (0.21 to 0.31)		0.26 (0.21 to 0.31)	0.26 (0.23 to 0.3)
Caribbean	13.2 (13.1 to 13.3)	14.1 (13.9 to 14.3)	14.6 (13.8 to 15.4)				9.89 (8.95 to 10.7)	10.5 (9.48 to 11.4)	10.7 (9.54 to 11.8)		0.25 (0.19 to 0.32)		0.26 (0.2 to 0.32)	0.26 (0.23 to 0.31)
Central Latin America	13.3 (13.3 to 13.4)	14.7 (14.7 to 14.8)	15.4 (14.6 to 16.1)				9.55 (8.52 to 10.5)	10.7 (9.58 to 11.7)	11.1 (9.82 to 12.3)		0.28 (0.22 to 0.36)		0.27 (0.21 to 0.35)	0.28 (0.24 to 0.33)
Tropical Latin America	12.6 (12.5 to 12.6)	14.4 (14.4 to 14.5)	15.3 (15.1 to 15.4)				8.98 (8.01 to 9.86)	10.4 (9.32 to 11.4)	11.1 (9.94 to 12.1)		0.28 (0.22 to 0.36)		0.28 (0.21 to 0.35)	0.27 (0.22 to 0.34)
**North Africa and Middle East**	11.3 (11.1 to 11.5)	12.4 (12.2 to 12.7)	13 (12.5 to 13.4)				8.23 (7.38 to 9.04)	9.01 (8.11 to 9.88)	9.33 (8.35 to 10.3)		0.27 (0.21 to 0.33)		0.28 (0.22 to 0.33)	0.28 (0.23 to 0.33)
**South Asia**	9.49 (9.29 to 9.69)	10.8 (10.5 to 11)	12.4 (11.8 to 13)				6.51 (5.77 to 7.24)	7.37 (6.5 to 8.18)	8.46 (7.41 to 9.5)		0.31 (0.25 to 0.38)		0.31 (0.26 to 0.38)	0.32 (0.27 to 0.37)
**South East Asia, East Asia, and Oceania**	10.8 (10.5 to 11.2)	11.7 (11.5 to 12)	13.4 (12.8 to 14)				7.84 (7.02 to 8.62)	8.47 (7.63 to 9.26)	9.62 (8.62 to 10.6)		0.27 (0.23 to 0.33)		0.28 (0.23 to 0.33)	0.28 (0.24 to 0.33)
East Asia	10.6 (10.2 to 11.2)	11.6 (11.3 to 12)	13.5 (12.8 to 14.3)				7.76 (6.96 to 8.56)	8.45 (7.61 to 9.24)	9.76 (8.69 to 10.8)		0.27 (0.23 to 0.32)		0.27 (0.23 to 0.33)	0.28 (0.24 to 0.32)
Oceania	10.3 (9.83 to 10.8)	10.3 (9.7 to 10.9)	10.7 (9.82 to 11.5)				7.34 (6.51 to 8.14)	7.35 (6.47 to 8.19)	7.6 (6.58 to 8.6)		0.29 (0.24 to 0.34)		0.29 (0.25 to 0.33)	0.29 (0.25 to 0.33)
South East Asia	11.5 (11.3 to 11.7)	12 (11.8 to 12.2)	12.8 (12.4 to 13.3)				8.14 (7.26 to 8.94)	8.57 (7.68 to 9.42)	9.1 (8.08 to 10)		0.29 (0.24 to 0.36)		0.29 (0.23 to 0.35)	0.29 (0.25 to 0.35)
**Sub‐Saharan Africa**	10.1 (9.88 to 10.3)	10.4 (10.1 to 10.6)	11.5 (11.1 to 11.8)				7.33 (6.59 to 8.04)	7.54 (6.79 to 8.24)	8.39 (7.55 to 9.2)		0.28 (0.22 to 0.33)		0.27 (0.22 to 0.33)	0.27 (0.22 to 0.32)
Central sub‐Saharan Africa	9.32 (8.83 to 9.79)	9.83 (9.4 to 10.3)	10.9 (10.1 to 11.6)				6.73 (6 to 7.47)	7.13 (6.4 to 7.86)	7.96 (7.01 to 8.89)		0.28 (0.24 to 0.32)		0.27 (0.24 to 0.32)	0.27 (0.23 to 0.3)
Eastern sub‐Saharan Africa	9.37 (9.16 to 9.59)	10.2 (10 to 10.4)	11.4 (11.1 to 11.7)				6.81 (6.11 to 7.45)	7.44 (6.68 to 8.12)	8.34 (7.51 to 9.1)		0.27 (0.22 to 0.33)		0.27 (0.22 to 0.33)	0.27 (0.22 to 0.32)
Southern sub‐Saharan Africa	12.9 (12.7 to 13.1)	10.9 (10.8 to 11.1)	12.4 (12.2 to 12.6)				9.19 (8.24 to 10.1)	7.78 (6.99 to 8.55)	8.82 (7.92 to 9.71)		0.29 (0.23 to 0.35)		0.29 (0.23 to 0.35)	0.29 (0.23 to 0.35)
Western sub‐Saharan Africa	10.2 (9.84 to 10.5)	10.4 (10 to 10.9)	11.5 (11 to 11.9)				7.4 (6.63 to 8.11)	7.65 (6.87 to 8.4)	8.42 (7.54 to 9.27)		0.27 (0.23 to 0.33)		0.27 (0.23 to 0.32)	0.27 (0.22 to 0.31)

2005 has been selected as midpoint of period 1990-2019, allowing comparisons between almost equal time periods. Data in parentheses are 95% uncertainty intervals.

LE-70=life expectancy at age 70; HALE-70=healthy life expectancy at age 70; PYIH-70=proportion of years in ill health at age 70.

More than 90% of the 204 countries and territories had increased LE-70 and HALE-70 between 1990 and 2019. The largest increases in LE-70 for men were in Singapore, South Korea, Bermuda, Maldives, and Luxembourg. In contrast, there were decreases of at least two years in LE-70 in Uzbekistan, Tajikistan, Nicaragua, Honduras, and Azerbaijan. Despite trends of slight PYIH-70 increases with increasing SDI and HAQ index, only 74 of 204 countries had decreased PYIH-70 between 1990 and 2019. The biggest improvements, in order, were seen in Côte d’Ivoire, Iraq, Singapore, Ukraine, and Kyrgyzstan, all of which saw declines of at least 1% in PYIH-70. In contrast, 64 countries had increases in PYIH-70 of at least 2%, including Sri Lanka, US, Seychelles, Georgia, Lebanon, and Syrian Arab Republic. LE-70 was higher in women than in men in 195 of 204 countries by an average of 1.89 years. The only exceptions were Afghanistan, Algeria, Egypt, Jordan, Marshall Islands, Mauritania, Qatar, Syrian Arab Republic, and Tokelau, where LE-70 was higher in men in 2019.

### Causal decomposition of changes in probability of death

**Fig 4:**
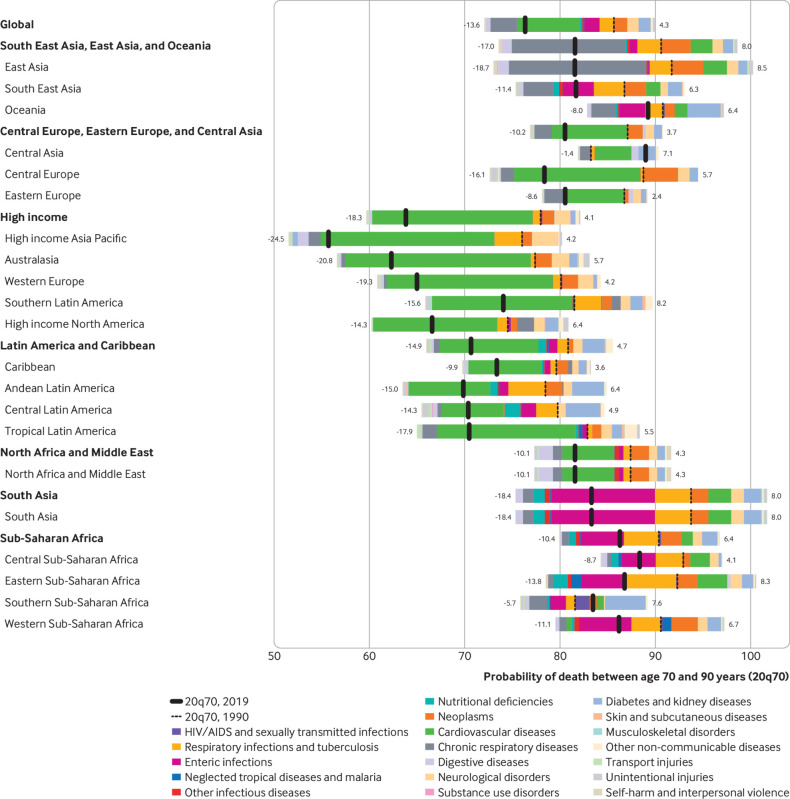
Relation between level 2 causes of death and changes in probability of death between ages 70 and 90 years (20q70) for both sexes by location, 1990-2019. Different colour bars represent different causes of death. All causes to the right of the dotted black line increased from 1990 to 2019, and all those to the left decreased over the same time period. At the global level, the probability of death decreased mainly due to reductions in cardiovascular diseases, chronic respiratory diseases, respiratory infections and tuberculosis, and enteric infections (−13.6% in total), while the probability of death increased due to increases in neoplasms, neurological disorders, diabetes, and kidney diseases (+4.3% in total). 20q70=probability ‘q’ of death for a period of 20 years starting at age 70

We performed causal decomposition of probability of death for the population aged 70-90 from 1990 to 2019 for level 2 GBD causes (fig 4; supplementary figs 17-19). Nearly all regions saw declines in probability of death from age 70 to 90 (thick black line on the left of dotted black line), with the only exceptions being Central Asia (83.2% probability of death in 1990 vs 89.0% in 2019) and southern sub-Saharan Africa (81.6% probability of death in 1990 vs 83.5% in 2019). Broad declines were mostly due to reductions in mortality from cardiovascular diseases and chronic respiratory diseases; declines would have been even greater if not for increases in mortality attributed to neoplasms, diabetes and kidney diseases, and neurological disorders in men and women. Trends in falls and unintentional injuries are important to highlight as proxies for frailty in older adults. In 121 countries, the probability of death by unintentional injury and falls decreased between 1990 and 2019.

### Leading causes of mortality and morbidity

**Fig 5:**
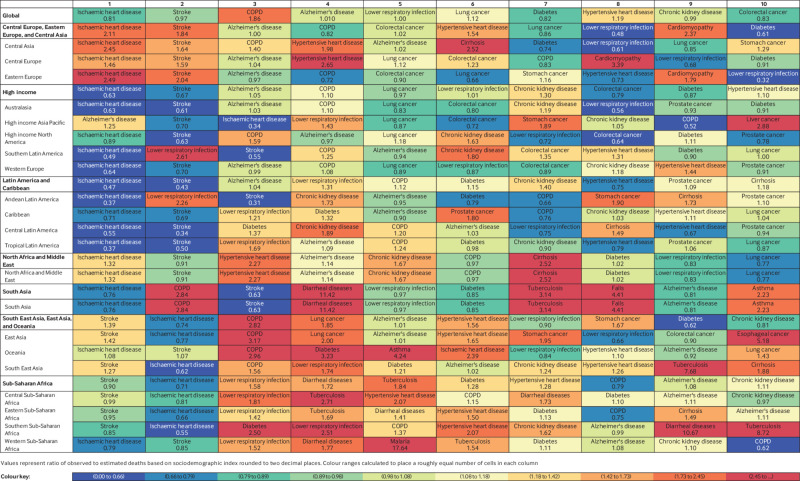
Ten leading causes of total deaths with ratio of observed to expected deaths in 2019 by location for population aged ≥70, both sexes. Causes are ranked according to global estimates of deaths and colour coded based on ratio of observed to expected rates. Shades of blue represent lower observed deaths than expected rates based on sociodemographic index whereas red indicates observed deaths exceeded expected rates. Ratios are listed in each cell; ratios greater than one indicate that observed levels exceeded expected levels based on sociodemographic index. COPD=chronic obstructive pulmonary disease

In 2019, the most notable causes of disease burden were cardiovascular diseases, neoplasms, and chronic respiratory diseases, while the least burden was caused by other infectious diseases and unintentional injuries (among other causes) for men and women (data given in online supplementary material). Globally the top five level 3 causes of death in people aged ≥70 in 2019 were ischaemic heart disease, stroke, chronic obstructive pulmonary disease, Alzheimer’s disease and other dementias, and lower respiratory infections (fig 5; supplementary figs 20 and 21 by country and sex). Although ischaemic heart disease, stroke, colorectal cancer, diabetes, and chronic kidney disease remained among the leading causes of death globally, observed levels of deaths were generally lower than those expected based on SDI (ratio of observed to expected levels less than one; fig 5). Increases in death rates in people aged ≥70 from 1990 to 2019 were noted for Alzheimer’s disease and other dementias (+29.28%), lung cancer (+11.74%), diabetes (+16.35%), and chronic kidney disease (+31.95%) (data given in online supplementary material**)**. Based on the observed to expected mortality ratios, Alzheimer’s disease and other dementias (ratio 1.05) was expected to have slightly higher estimates than the observed proportion of 29.28%, while for diabetes (ratio 0.82) the increase was expected to be a bit lower than 31.95%. Falls also increased 15.28% and were ranked 13th in 2019. Neoplasms showed heterogeneity, with half of cancers (15 of 30) increasing and half decreasing. The top two neoplasms—tracheal, bronchus and lung cancer, and colorectal cancer—both increased (while lung cancer had an observed to expected ratio of 1.12 and the ratio for colorectal cancer was 0.83; fig 5), and the largest increase was in pancreatic cancer (+32.48%, ranked 18th overall). Cancers of the stomach (−33.34%), prostate (−3.74%), breast (−9.28%), oesophagus (−14.55%), and liver (−10.48%) all decreased from 1990 to 2019 in people aged ≥70 (data given in online supplementary material).

**Fig 6:**
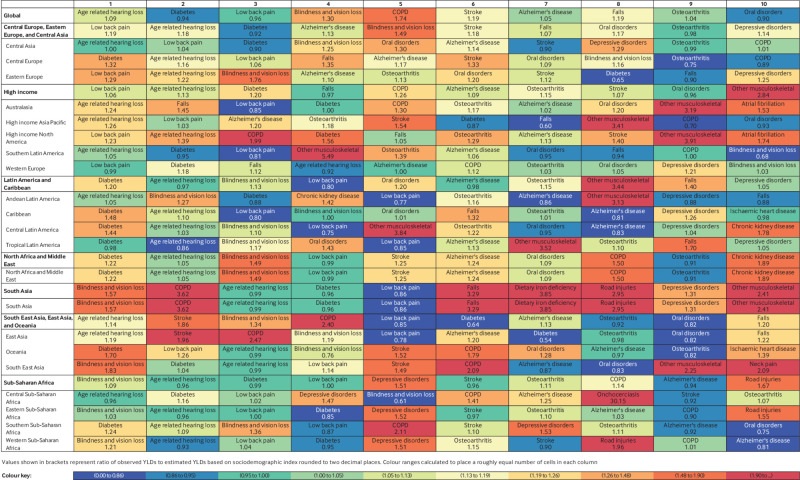
Ten leading causes of total years lived with disability (YLDs) with ratio of observed to expected YLDs in 2019 by location for population aged ≥70, both sexes. Causes are ranked according to global estimates of YLDs and colour coded based on ratio of observed to expected rates. Shades of blue represent lower observed YLDs than expected rates based on sociodemographic index whereas red indicates observed YLDs exceeded expected rates. Ratios are listed in each cell; ratios greater than one indicate that observed levels exceeded expected levels based on sociodemographic index. COPD=chronic obstructive pulmonary disease

Globally, the top 5 level 3 causes of years lived with disability in people aged ≥70 included age related hearing loss, diabetes, low back pain, blindness and vision loss, and chronic obstructive pulmonary disease (fig 6, supplementary figs 22 and 23 by country and sex). Observed years lived with disability due to chronic obstructive pulmonary disease were almost two times (observed to expected ratio 1.8) higher than expected levels worldwide. Among all 10 leading causes of years lived with disability, observed estimates were almost equal (observed to expected ratio >0.9) or exceeded the levels expected based on SDI (fig 6). Leading causes of disability in older people were largely consistent across locations, but with variations in ranking. Age related hearing loss was the leading cause in 47 countries, while diabetes ranked first in 98 countries. Notably, Alzheimer’s disease and other dementias was in the top five in 51 countries, osteoarthritis in 28 countries, oral disorders in 29 countries, and chronic obstructive pulmonary disease in 50 countries (supplementary figs 22 and 23).

### Attributable burden and risk factor exposure trends

In 2019, 280 million disability adjusted life years (95% uncertainty interval 261.3 to 297.9), or 57.7% of the total, were attributable to risk factors in people aged ≥70; this includes 87.9 (79.4 to 95.8), 147.8 (134.8 to 163.4), and 172.0 (155.4 to 189.3) million disability adjusted life years attributable to environmental, behavioural, and metabolic risks, respectively. This represents an increase in the total risk attributable disability adjusted life years from the estimated 165.3 million (157.8 to 172.5) in 1990, but a decrease in the proportion that were risk attributable in 1990 (61.9%). The top five risk factors in 2019 were high systolic blood pressure, high fasting plasma glucose, smoking, high low density lipoprotein cholesterol, and high body mass index. The only substantial change in ranking was a decline of 70.1% in burden attributable to household air pollution (data shown in online supplementary material).

**Fig 7:**
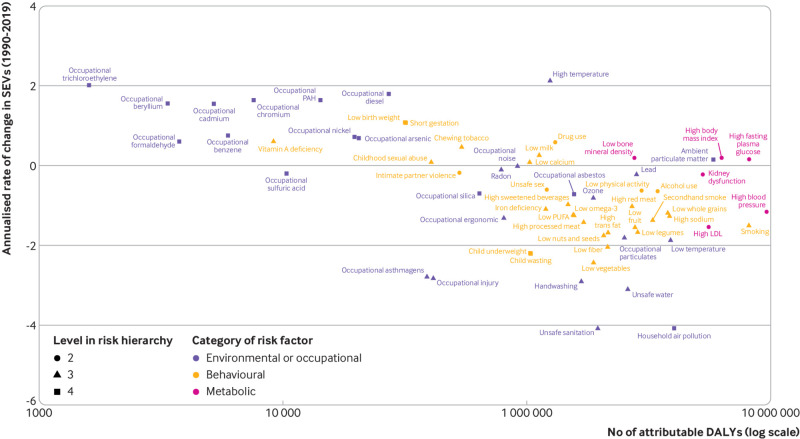
Comparison of annualised rate of change in risk exposure measured by summary exposure values (SEVs) for population aged ≥70 (both sexes) from 1990 to 2019 with total attributable disability adjusted life years (DALYs) for all risk factors in 2019. The fraction of disability adjusted life years attributed to each risk factor is depicted in relation to their corresponding population summary exposure values in 2019. Risk factors are colour coded by environmental or occupational (purple), behavioural (yellow), or metabolic (pink) risk factors, and different levels of the risk hierarchy are indicated by different shapes. LDL=low density lipoprotein; PUFA=polyunsaturated fat

A comparison of annualised rate of change in risk exposure measured by summary exposure values from 1990 to 2019 with total attributable disability adjusted life years in 2019 shows that the biggest risk factors for health loss were mainly those with the largest cumulative improvements in exposure (fig 7, supplementary figs 24-36). Those with an annualised rate of change in summary exposure values of at least 2% decline and to which at least 100 000 disability adjusted life years globally are attributed include household air pollution, unsafe water, low dietary fibre, unsafe sanitation, low vegetables, poor handwashing, child wasting, and child underweight (which includes protein energy malnutrition in adults as well), and occupational injuries and asthmagens. Notable differences existed among diverse sociodemographic levels and super regions (supplementary figs 24-36). Analysing common risks among men and women in the high SDI group, drug use, followed by low birth weight or short gestation, had the highest increase in summary exposure values and attributable disability adjusted life years. In comparison, the middle SDI group had the highest increases for high temperature (one of two climate indicators, the other being low temperature) and high body mass index. The low-middle and low SDI groups noted the highest annualised rate of change in parallel with attributable disability adjusted life years related to ambient particulate matter air pollution and high body mass index.

### DCI for population aged ≥70 

Between 1990 and 2019, data coverage for all risks and non-fatal outcomes in the population aged ≥70 remained at low levels compared with that for all ages (supplementary tables 9-12). Across GBD locations, between 1990 and 2005, and between 2005 and 2019, 87 countries increased data completeness for risk factors for older adults and 117 countries increased DCI percentage for all ages. Meanwhile, the DCI percentage for non-fatal outcomes in adults aged ≥70 increased in 57 countries, while the DCI percentage for all ages increased in 198 countries. Non-fatal DCI percentage for the population aged ≥70 decreased in 125 countries. Analysing only risk factor data completeness between the same time periods and age groups showed that nine risk factors had 0% completeness for both periods among those aged ≥70 (supplementary table 10). For the entire period, all risk factors completeness for older adults was at an equal or lower level compared with that of all ages. Additionally, for all ages and those aged ≥70, data completeness decreased between 1990 and 2005, and between 2005 and 2019 for 16 and 32 risks, respectively. Traditionally high burden modifiable risks, including dietary risks, high cholesterol levels, and high fasting plasma glucose, had low to moderate data availability for the population aged ≥70 (DCI range 29%−63%; supplementary table 10). Similar patterns were noted for environmental and occupational risks. Furthermore, when we analysed completeness for non-fatal causes, we found that 52 non-fatal causes had zero coverage for both periods for the population aged ≥70, while 53 non-fatal causes had 100% completeness in all ages and the population aged ≥70.

## Discussion

### Life expectancy, fatal and non-fatal causes, and risk factor patterns

Adults aged ≥70 were more likely to live longer in 2019 than in 1990 in almost all countries. In the global population aged ≥70, an extension of life was recorded of almost two years in total (LE-70) or almost 1.5 years free of disease (HALE-70). Disease burden was closely associated with societal development and aggregate healthcare quality, but starting at the age of 70, relatively low regional variability was found for LE-70, which could be attributed to a lack of variation in SDI and HAQ index within regions. Women generally lived longer but had a higher proportion of those years spent in ill health.

Steady increases in LE-70 have been described even before 1990,^31^ with variation of life expectancy thought to be a complex function of age specific mortality, risk factor exposures, and biomedical advances.^32^ This supports our findings of low regional variability in life expectancy beyond aged 70. Further, healthy ageing trends are not random^33^ because evidence exists that determinants as diverse as lifestyle and socioeconomic development have predictable effects on healthy ageing.^34^ Our study showed widespread decreases in cardiovascular and chronic respiratory diseases, and in some cancers. Nonetheless, increases in deaths were found to be caused by neurological disorders, falls, and some cancer types that have not been historically targeted by prevention programmes. The fatal and non-fatal burden of injuries due to falls increased in several countries, suggesting that functional loss will have a role in the burden of disease among older adults.^35^ Interventions targeting diseases that progressively impair physical functionality^36^ might be required to alter this pattern. The main disability drivers globally were disorders related to functional status (eg, Alzheimer’s disease and other dementias, and stroke), conditions associated with longstanding pain (eg, low back pain, neck pain, osteoarthritis, road injuries), deficits in sensory organ functioning (eg, age related hearing loss, blindness and vision loss), and oral disorders. Alzheimer’s disease and other dementias have an important role in people’s functional status, and based on our analysis, are contributing to higher mortality and morbidity rates than expected among older adults, a fact that is also supported by the literature.^37^ ^38^ Projections suggest that the older population is expected to exceed 20% of the global population by 2050.^39^ This growing number of older people is likely to present a challenge in terms of health needs and care costs.^40^

### Disability burden and functional loss among older adults

Conditions limiting physical function, pain symptoms, and sensory organ deficits were the main drivers of morbidity among the older population. Importantly, our global analysis showed that among the 10 leading causes of death and disability, four were the same: blindness, hearing loss, low back pain, respiratory disease, oral disorders (total tooth loss), and falls represent a group of causes that feature direct functional decline,^41^ while other causes of years lived with disability, such as diabetes, are indirectly related to disability and functional loss.^7^ ^42^ Sex stratified analysis showed a similar pattern, with differentiating drivers being falls for older women and strokes for older men.^43^ ^44^ This information could help public health policy makers implement new tailored programmes to control and prevent functional loss and disability progression among older people.

Before this study, information was limited as to whether today’s older adults live extra years of life in better health than their ancestors^34^ or whether there is support for the theories of equilibrium of morbidity and delayed ageing.^45^ ^46^ Under the compression of morbidity scenario, increases in life expectancy are coupled with decreases in the proportion of life spent in ill health because of shifts in future disease patterns that delay disease onset. However, the expansion of morbidity scenario supports a life expectancy increase coupled with increases in life spent in ill health occurring due to advances in medicine, while disease patterns remain similar.^47^ ^48^ Our findings of a strong association between higher SDI and HAQ index levels with LE-70 and HALE-70 and relatively stagnated trends in PYIH-70 were consistent with previous regional analyses.^14^ ^49^ Some reported relations were not as strong for some measures of health (PYIH-70); this could be attributed to a relatively similar experience of ageing across the development spectrum, with accumulation of deficits proceeding as a function of biology more than environment. Additionally, summary measures of development and healthcare quality might not be as closely associated with healthy ageing as they are for health outcomes in younger populations. The above findings might also reflect that comparatively fewer data are available to quantify epidemiology in older adults.

### Policy implications

The present findings have three main implications for health policy and data collection. Firstly, country specific benchmarks can be used to develop and implement health intervention programmes to address and reduce the burden of disability in older adults while tracking regional estimates.^50^ These programmes need to account for the increase in healthcare spending due to population ageing, particularly relating to long term healthcare.^51^ ^52^ ^53^ Without preemptive planning, even among socioeconomically developed countries, a projected lack of long term care services might overwhelm the hospital system.^54^ Secondly, the cause and risk specific insights from this analysis could help to draft policies focusing on prevention of functional loss and disability progression among older people, specifically targeting men and women, and different sociodemographic levels. Policy efforts to reduce exposure to smoking and ambient and household air pollution have paid dividends, and should continue and expand. With growing evidence showing that older people are particularly vulnerable to environmental risk factors,^55^ ^56^ similar widespread efforts are needed to tackle increasing exposure to other risk factors, including the oncoming effects of climate change such as extreme weather events, natural disasters, and wildfires. While some accumulation of disease is related to altered biological metabolism and epigenetic signals,^57^ other conditions are preventable. The degree of associated disability could be limited by a combination of healthy ageing surveillance at a population level and redevelopment of healthcare services towards sustainable development in a rapidly ageing society.^58^ ^59^

Thirdly, evaluation of data coverage showed that categorically there are fewer health data available relating to older adults. Specifically, the coverage of risk factor data for the population aged ≥70 decreased in almost 30% of the GBD locations, while since 1990 no information is available for nine risk factors in older adults. This comparative lack of data could represent an imminent threat and highlights the urgent need for surveillance mechanisms in locations with low coverage of risk factors and non-fatal disease burden data in older populations.^60^

Because our analysis covers a period before the covid-19 pandemic, we were unable to analyse the effect of covid-19 on mortality. International studies indicate that most of the deaths have occurred in older adults, with a case fatality rate for the population aged ≥70 of over 10%, which could even increase to 30%.^61^ Hospital admissions and mortality rates for covid-19 are also strongly associated with older age.^62^ Covid-19 will probably be one of the top ranked causes of death and disability adjusted life years in people aged ≥70 for 2020, with studies from high income countries having consistently reported that older adults are disproportionately affected by the ongoing pandemic. Additionally, covid-19 might have long term health implications related to functional decline and health related quality of life among older people.^63^ Although exploring the impacts of covid-19 is beyond the scope of this study, our analysis should serve as a baseline for evaluating the impacts in the coming years.

### Strengths and limitations of this study

This research has several strengths. It has added knowledge about the burden of disease and disability in older adults by analysing data for 204 countries and territories. It has also evaluated health data coverage at global and regional levels for the population aged ≥70. However, this analysis has several limitations. Firstly, because it is based on GBD 2019, it shares the overall limitations described in previous publications,^24^ ^25^ ^26^ including challenges in fully quantifying all sources of uncertainty, lags in data availability, and variation in coding practices and other biases. Secondly, overall input data were limited, especially in lower SDI settings. Thirdly, although we examined a number of associations between plausibly related factors such as SDI, conclusions cannot be drawn about causal relations. Fourthly, the GBD study treats conditions individually when determining mortality and morbidity estimates; however, because multimorbidity is highly prevalent in older populations, it is plausible that this approach leads to overestimation of total disability.^64^ Therefore, individual cause risk might not be fully reflected in population level averages because when the cause manifests in people with a high burden, it could account for a higher proportion of ill health and premature mortality.

### Conclusions

Globally adults aged ≥70 were found to live substantially longer in 2019 than in 1990, particularly owing to decreases in death due to cardiovascular diseases and chronic respiratory diseases. However, disability burden rates are following a stable pattern mostly attributable to functional decline, injuries due to falls, hearing loss, and back pain. Globally monitoring mortality and morbidity risk factors is crucial to sustain and advance research and health policy among older adults. Regions with lower sociodemographic development and healthcare quality performed at lower levels, highlighting the areas in greatest need. Our findings show we should develop and implement targeted strategies aimed at functional ability, sensory organ deficits, symptoms of pain, and unintentional falls. Programmes need to address country specific sociodemographic and cultural development because universal plans might be inefficient. Public health strategies will require a coherent ageing health policy, targeted data coverage, and consistent collaboration among stakeholders to succeed. The present estimates could serve as a healthy ageing benchmark for countries working to focus ageing policies on key risk factors and determinants, improve healthcare access and quality, and lower healthcare costs.

**What is already known on this topic**

The global population is living longer and the health and wellbeing of older adults has become a major public health issue
While it is recognised that populations are ageing differently, global epidemiological data on the burden of diseases in adults aged ≥70 are lacking

**What this study adds**

In 2019, adults aged ≥70 were found to live more than two years longer compared with 1990 life expectancy estimates
Sociodemographic development and healthcare access and quality were major determinants of life expectancy and healthy life expectancy in adults aged ≥70; disability burden followed a stable pattern across the development spectrum
Specific healthy ageing policies are needed with targeted data coverage for adults aged ≥70 and consistent collaboration among stakeholders

## Data Availability

Data of the GBD study are publicly available at https://www.healthdata.org/results/data-visualizations.

## Web extra

Extra material supplied by authors

Web appendix: Supplementary material

Web appendix: Acknowledgments and declarations

## Notes

Contributors: ST is first author. GAK and NJK are joint senior authors. ST, GAK, AS, and NJK designed the study and drafted the initial manuscript. ST, GAK, VK, and NJK analysed the data. All other authors contributed data, interpreted the data, or revised the manuscript. All authors read and approved the final manuscript. NJK is the guarantor. The corresponding author attests that all listed authors meet authorship criteria and that no others meeting the criteria have been omitted.

The corresponding author (NJK) affirms that the manuscript is an honest, accurate, and transparent account of the study being reported; that no important aspects of the study have been omitted; and that any discrepancies from the study as planned (and, if relevant, registered) have been explained. The GBD study is compliant with the Guidelines for Accurate and Transparent Health Estimates Reporting (GATHER).

Dissemination to participants and related patient and public communities: We plan to disseminate these research findings to a wider community via press releases, featuring on the healthdata.org website, via social media platforms, and presentation at international fora.

Provenance and peer review: Not commissioned; externally peer reviewed.
