# Supplementary material for: Global, regional, and national burden of diseases and injuries for adults 70 years and older: systematic analysis for the Global Burden of Disease 2019 Study
Source: BMJ. 2022 Mar 10;376:e068208. doi: 10.1136/bmj-2021-068208 (PMC9316948; doi:10.1136/bmj-2021-068208)
Supplement: Supplementary file 1 — Web appendix: Supplementary material [file tyrs068208.ww1.pdf]

Supplementary Material (Appendix) for: Global, regional, and national burden of diseases and injuries for adults 70 years and older: systematic analysis for the Global Burden of Disease 2019 Study

This appendix provides specific methodological detail, supplemental figures, and more detailed results for "Global, regional, and national burden of diseases and injuries for adults 70 years and older: systematic analysis for the Global Burden of Disease 2019 Study."

Contents

GBD 2017 Ageing Collaborators 5

Global Burden of Disease overview 31

GATHER statement 31

GBD cause list and location hierarchy 31

Socio-demographic Index (SDI) 31

Data sources 32

Mortality and death cause-specific estimation process for those over 70 years old 32

Non-fatal modelling methods for those 70 years and older 32

Healthcare Access and Quality (HAQ) Index 33

References 33

Figure S1. Global population change by age group for both sexes combined (top panel), population percentagechange during the same period (upper middle panel), rate of change in years lived with disability (YLDs; lower middle panel), and rate of change in All-cause mortality (bottom panel) between 1990 and 2019 in Low Socio-demographic Index (SDI) regions. 34

Figure S2. Global population change by age group for both sexes combined (top panel), population percentage change during the same period (upper middle panel), rate of change in years lived with disability (YLDs; lower middle panel), and rate of change in All-cause mortality (bottom panel) between 1990 and 2019 in Low-middle Socio-demographic Index (SDI) regions. 35

Figure S3. Global population change by age group for both sexes combined (top panel), population percentage change during the same period (upper middle panel), rate of change in years lived with disability (YLDs; lowermiddle panel), and rate of change in All-cause mortality (bottom panel) between 1990 and 2019 in Middle Socio-demographic Index (SDI) regions. 36

Figure S4. Global population change by age group for both sexes combined (top panel), population percentagechange during the same period (upper middle panel), rate of change in years lived with disability (YLDs; lower middle panel), and rate of change in All-cause mortality (bottom panel) between 1990 and 2019 in High-middle Socio-demographic Index (SDI) regions. 37

Figure S5. Global population change by age group for both sexes combined (top panel), population percentage change during the same period (upper middle panel), rate of change in years lived with disability (YLDs; lower middle panel), and rate of change in All-cause mortality (bottom panel) between 1990 and 2019 in High Socio- demographic Index (SDI) regions. 38

Figure S6. Global population change by age group for both sexes combined (top panel), population

percentage change during the same period (upper middle panel), rate of change in years lived with disability (YLDs; lower middle panel), and rate of change in All-cause mortality (bottom panel) between 1990 and 2019 in the Central Europe, Eastern Europe and Central Asia GBD super-region.39

Figure S7. Global population change by age group for both sexes combined (top panel), population percentage change during the same period (upper middle panel), rate of change in years lived with disability (YLDs; lower middle panel), and rate of change in All-cause mortality (bottom panel) between 1990 and 2019 in the High- income GBD super-region. 40

Figure S8. Global population change by age group for both sexes combined (top panel), population percentage change during the same period (upper middle panel), rate of change in years lived with disability (YLDs; lower middle panel), and rate of change in All-cause mortality (bottom panel) between 1990 and 2019 in the Latin America and Caribbean GBD super-region. 41

Figure S9. Global population change by age group for both sexes combined (top panel), population percentage change during the same period (upper middle panel), rate of change in years lived with disability (YLDs; lower middle panel), and rate of change in All-cause mortality (bottom panel) between 1990 and 2019 in the North Africa and Middle East super-region. 42

Figure S10. Global population change by age group for both sexes combined (top panel), population percentage change during the same period (upper middle panel), rate of change in years lived with disability (YLDs; lower middle panel), and rate of change in All-cause mortality (bottom panel) between 1990 and 2019 in the South Asia GBD super-region. 43

Figure S11. Global population change by age group for both sexes combined (top panel), population percentage change during the same period (upper middle panel), rate of change in years lived with disability (YLDs; lower middle panel), and rate of change in All-cause mortality (bottom panel) between 1990 and 2019 inthe Southeast Asia, East Asia and Oceania GBD super-region. 44

Figure S12. Global population change by age group for both sexes combined (top panel), population percentage change during the same period (upper middle panel), rate of change in years lived with disability (YLDs; lower middle panel), and rate of change in All-cause mortality (bottom panel) between 1990 and 2019 in the Sub-Saharan Africa GBD super-region 45

Figure S13. Life expectancy at 70+ (LE-70), healthy life expectancy at 70+ (HALE-70), and proportion of life- years spent in ill health (PYIH-70) at age 70+ GBD by super-region between 1990 and 2019, in males. 46

Figure S14. Life expectancy at 70+ (LE-70), healthy life expectancy at 70+ (HALE-70), and proportion of life- years spent in ill health (PYIH-70) at age 70+ GBD by super-region between 1990 and 2019, in females. 47

Figure S15. Life expectancy (LE-70), healthy life expectancy (HALE-70) and proportion of life-years spent in ill health at age 70+ (PYIH-70) versus Socio-demographic Index (SDI) and Healthcare Access and Quality (HAQ) Index in 2019, in males. 48

Figure S16. Life expectancy (LE-70), healthy life expectancy (HALE-70) and proportion of life-years spent in ill health at age 70+ (PYIH-70) versus Socio-demographic Index (SDI) and Healthcare Access and Quality (HAQ) Index in 2019, in females. 49

Figure S17. Decomposition of the probability of death, for all GBD locations, for the population aged 70to 90 years old, from 1990 to 2019, in both sexes. 50

Figure S18. Decomposition of the probability of death, for all GBD locations, for the population aged 70 to 90 years old, from 1990 to 2019, in males... 52

Figure S19. Decomposition of the probability of death, for all GBD locations, for the population aged 70 to 90 years old, from 1990 to 2019, in females. 54

Figure S20. Leading ten causes of total deaths with ratio of observed to expected years lived with disability in 2019, among people 70 years and older, in males, for all GBD locations. 56

Figure S21. Leading ten causes of total deaths with ratio of observed to expected years lived with disability in 2019, among people 70 years and older, in females, for all GBD locations. 59

Figure S22. Leading ten causes of total years lived with disability with ratio of observed to expected years lived with disability in 2019, among people 70 years and older, in males, for all GBD locations. 62

Figure S23. Leading ten causes of total years lived with disability with ratio of observed to expected years lived with disability in 2019, among people 70 years and older, in females, for all GBD locations. 65

Figure S24. Global annualised rate of change in risk factors summary exposure values (SEV) in people aged 70 years and older, for both sexes, from 1990 to 2019 versus number of attributable DALYs by risk factor in 2019. 68

Figure S25. Annualised rate of change in risk factors summary exposure values (SEV) in people aged 70 years and older, for both sexes, from 1990 to 2019 versus number of attributable DALYs by risk factor in 2019, in Low SDI quintile. 69

Figure S26. Annualised rate of change in risk factors summary exposure values (SEV) in people aged 70 years and older, for both sexes, from 1990 to 2019 versus number of attributable DALYs by risk factor in 2019, in Low-middle SDI quintile. 70

Figure S27. Annualised rate of change in risk factors summary exposure values (SEV) in people aged 70 years and older, for both sexes, from 1990 to 2019 versus number of attributable DALYs by risk factor in 2019, in Middle SDI quintile. 71

Figure S28. Annualised rate of change in risk factors summary exposure values (SEV) in people aged 70 years and older, for both sexes, from 1990 to 2019 versus number of attributable DALYs by risk factor in 2019, in High-middle SDI quintile. 72

Figure S29. Annualised rate of change in risk factors summary exposure values (SEV) in people aged 70 years and older, for both sexes, from 1990 to 2019 versus number of attributable DALYs by risk factor in 2019, in High SDI quintile. 73

Figure S30. Annualised rate of change in risk factors summary exposure values (SEV) in people aged 70 years and older, for both sexes, from 1990 to 2019 versus number of attributable DALYs by risk factor in 2019, in the Central Europe, Eastern Europe and Central Asia GBD super-region. 74

Figure S31. Annualised rate of change in risk factors summary exposure values (SEV) in people aged 70 years and older, for both sexes, from 1990 to 2019 versus number of attributable DALYs by risk factor in 2019, in the High-income GBD super-region. 75

Figure S32. Annualised rate of change in risk factors summary exposure values (SEV) in people aged 70 years and older, for both sexes, from 1990 to 2019 versus number of attributable DALYs by risk factor in 2019, in the Latin America and Caribbean GBD super-region 76

Figure S33. Annualised rate of change in risk factors summary exposure values (SEV) in people aged 70 years and older, for both sexes, from 1990 to 2019 versus number of attributable DALYs by risk factor in 2019, in the North Africa and Middle East super-region. 77

Figure S34. Annualised rate of change in risk factors summary exposure values (SEV) in people aged 70 years and older, for both sexes, from 1990 to 2019 versus number of attributable DALYs by risk factor in 2019, in the South Asia GBD super-region........ 78

Figure S35. Annualised rate of change in risk factors summary exposure values (SEV) in people aged 70 years and older, for both sexes, from 1990 to 2019 versus number of attributable DALYs by risk factor in 2019, in the Southeast Asia, East Asia and Oceania GBD super-region. 79

Figure S36. Annualised rate of change in risk factors summary exposure values (SEV) in people aged 70 years and older, for both sexes, from 1990 to 2019 versus number of attributable DALYs by risk factor in 2019, in the Sub-Saharan Africa GBD super-region. 80

Supplemental Tables

Supplementary Table 1: GATHER checklist of information that should be included in reports of global health estimates, with description of compliance and location of information for "Global, regional, and national burden of diseases and injuries for adults 70 years and older: a systematic analysis for the Global Burden of Disease 2019 Study" 81

Supplementary Table 2. GBD location hierarchy, with SDI quintile 83

Supplementary Table 3: List of GBD causes, with levels 86

Supplementary Table 4: Cause-specific mortality rate for 70-79 years, 80-94 yearsand 95+ years in 2019, and percentage change from 1990 to 2019 in death number and rate in 70+ years, global, for all GBD causes 89

Supplementary Table 5. Cause-specific rate of YLDs for 70-79 years, 80-94 years,and 95+ years in 2019, and percentage change from 1990 to 2019 in YLD number and rate in 70+ years, global, for all GBD causes 96

Supplementary Table 6. Life expectancy, Health-adjusted life expectancy, and proportion of years spent in ill health in all reporting locations, in 1990, 2005, 2019 for Both sexes combined 103

Supplementary Table 7. Life expectancy, Health-adjusted life expectancy, and proportion of years spent in ill health in all reporting locations, in 1990, 2005, 2019 for Males 110

Supplementary Table 8. Life expectancy, Health-adjusted life expectancy, and proportion of years spent in ill health in all reporting locations, in 1990, 2005, 2019 for Females 116

Supplementary Table 9. Data coverage index for all risk factorsfor 204 reporting locations, 1990–2005, 2005–2019 and in total by all ages and for the 70+ age group, for both sexes 122

Supplementary Table 10. Data coverage index for all reporting locations for 69 risk factors, 1990–2005, 2005–2019 and in total by all ages and for the 70+ age group, for both sexes 128

Supplementary Table 11. Data coverage index for all non-fatal causes for 204 locations,among 1990–2005, 2005–2019 and in total by all ages and for the 70+ age group, for both sexes 130

Supplementary Table 12. Data coverage index for all locations for 286 non-fatal causes,among 1990–2005, 2005–2019 and in total by all ages and for the 70+ age group, for both sexes 135

GBD 2017 Ageing Collaborators

Stefanos Tyrovolas[% 1 2 447], Andy Stergachis[4 5], Varsha Sarah Krish[6], Angela Y Chang[7], Vegard Skirbekk[8], Joseph L Dieleman[6 9], Somnath Chatterji[10], Foad Abd-Allah[11], Mohammad Abdollahi[12 13], Aidin Abedi[14], Hassan Abolhassani[15 16], Akine Eshete Abosetugn[17], Lucas Guimarães Abreu[18], Michael R M Abrigo[19], Abdulaziz Khalid Abu Haimed[20], Maryam Adabi[21], Oladimeji M Adebayo[22], Isaac Akinkunmi Adedeji[23], Victor Adekanmbi[24], Olatunji O Adetokunboh[25 26], Davoud Adham[27], Shailesh M Advani[28 29], Mohsen Afarideh[30 31], Gina Agarwal[32], Mohammad Aghaali[33], Seyed Mohammad Kazem Aghamir[34], Anurag Agrawal[35 36], Sohail Ahmad[37], Tauseef Ahmad[38], Keivan Ahmadi[39], Mehdi Ahmadi[40 41], Muktar Beshir Ahmed[42 43], Rufus Olusola Akinyemi[44 45], Ziyad Al-Aly[46 47], Khurshid Alam[48], Fahad Mashhour Alanezi[49], Turki M Alanzi[50], Jacqueline Elizabeth Alcalde-Rabanal[51], Biresaw Wassihun Alemu[52 53], Samar Al-Hajj[54 55], Robert Kaba Alhassan[56], Saqib Ali[57], Gianfranco Alicandro[58], Mehran Alijanzadeh[59], Vahid Alipour[60 61], Syed Mohamed Aljunid[62 63], François Alla[64], Majid Abdulrahman Hamad Almadi[65 66], Amir Almasi-Hashiani[67], Abdulaziz M Almulhim[68], Rajaa M Al-Raddadi[69], Arya Aminorroaya[70 71], Fatemeh Amiri[72], Arianna Maever L Amit[73 74], Dickson A Amugsi[75], Etsay Woldu Anbesu[76], Robert Ancuceanu[77], Deanna Anderlini[78 79], Tudorel Andrei[80], Catalina Liliana Andrei[81], Sofia Androudi[82], Mina Anjomshoa[83], Fereshteh Ansari[84 85], Alireza Ansari-Moghaddam[86], Carl Abelardo T Antonio[87 88], Benny Antony[89], Davood Anvari[90 91], Razique Anwer[92], Jalal Arabloo[60], Morteza Arab-Zozani[93], Johan Ärnlöv[94 95], Malke Asaad[96], Mehran Asadi-Aliabadi[97], Ali A Asadi-Pooya[98 99], Maha Moh'd Wahbi Atout[100], Marcel Ausloos[101 80], Floriane Ausloos[102], Beatriz Paulina Ayala Quintanilla[103], Getinet Ayano[104], Martin Amogre Ayanore[105], Yared Asmare Aynalem[106], Samad Azari[60], Zelalem Nigussie Azene[107], Ebrahim Babaee[97], Ashish D Badiye[108], Arun Balachandran[109 110], Maciej Banach[111 112], Srikanta K Banerjee[113], Palash Chandra Banik[114], Suzanne Lyn Barker-Collo[115], Sanjay Basu[116 117], Bernhard T Baune[118 119], Mohsen Bayati[120], Bayisa Abdissa Baye[121], Neeraj Bedi[122 123], Ettore Beghi[124], Yannick Béjot[125 126], Michelle L Bell[127], Isabela M Bensenor[128], Akshaya Srikanth Bhagavathula[129 130], Pankaj Bhardwaj[131 132], Krittika Bhattacharyya[133 134], Suraj Bhattarai[135], Zulfiqar A Bhutta[136 137], Sadia Bibi[138], Ali Bijani[139], Boris Bikbov[140], Antonio Biondi[141], Binyam Minuye Birihane[142 143], Atanu Biswas[144], Tone Bjørge[145 146], Somayeh Bohlouli[147], Srinivasa Rao Rao Bolla[148], Archith Boloor[149], Soufiane Boufous[150], Dejana Braithwaite[29], Hermann Brenner[151], Andrew M Briggs[152], Nikolay Ivanovich Briko[153], Gabrielle B Britton[154 155], Sharath Burugina Nagaraja[156], Reinhard Busse[157], Zahid A Butt[158 159], Florentino Luciano Caetano dos Santos[160], Luis Alberto Cámera[161 162], Josip Car[163 164], Rosario Cárdenas[165], Giulia Carreras[166], Juan J Carrero[167], Felix Carvalho[168], Joao Mauricio Castaldelli-Maia[169], Carlos A Castañeda-Orjuela[170 171], Giulio Castelpietra[172 173], Franz Castro[155], Ester Cerin[174 175], Muge Cevik[176 177], Joht Singh Chandan[178], Alex R Chang[179], Jaykaran Charan[180], Vijay Kumar Chattu[181], Pankaj Chaturvedi[182 183], Sarika Chaturvedi[184], Prachi P Chavan[185], Simiao Chen[186], Nicolas Cherbuin[187], Ken Lee Chin[188 189], Daniel Youngwhan Cho[190], Mohiuddin Ahsanul Kabir Chowdhury[191 192], Dinh-Toi Chu[193], Sheng-Chia Chung[194 195], Michael T Chung[196], Liliana G Ciobanu[197 198], Vera Marisa Costa[168], Ewerton Cousin[199], Michael H Criqui[200], Marita Cross[201], Saad M A Dahlawi[202], Giovanni Damiani[203 204], Lalit Dandona[205 6 206], Rakhi Dandona[205 6 9], Parnaz Daneshpajouhnejad[207 208], Jai K Das[209], Rajat Das Gupta[192 210], Claudio Alberto Dávila-Cervantes[211], Kairat Davletov[212], Farah Deeba[213], Diego De Leo[214], Edgar Denova-Gutiérrez[215], Nikolaos Dervenis[216 217], Rupak Desai[218], Samath Dhamminda Dharmaratne[219 9 6], Govinda Prasad Dhungana[220], Mostafa Dianatinasab[221 222], Diana Dias da Silva[223], Daniel Diaz[224 225], Martin Dichgans[226], Shirin Djalalinia[227], Klara Dokova[228], Fariba Dorostkar[229], Leila Doshmangir[230], Bruce B Duncan[199], Andre Rodrigues Duraes[231 232], Arielle Wilder Eagan[233 234], Mohammad Ebrahimi Kalan[235], David Edvardsson[236 237], Andem Effiong[238], Joshua R Ehrlich[239 240], Islam Y Elgendy[241 242], Shaimaa I El-Jaafary[11], Iman El Sayed[243], Maysaa El Sayed Zaki[244], Maha El Tantawi[245], Babak Eshrati[97], Khalil Eskandari[246 247], Sharareh Eskandarieh[248], Saman Esmaeilnejad[249 250], Atkilt Esaiyas Etisso[251], Pawan Sirwan Faris[252 253], Andre Faro[254], Farshad Farzadfar[70], Mehdi Fazlzadeh[255 256], Valery L Feigin[257 6 258], Seyed-Mohammad Fereshtehnejad[94 259], Eduarda Fernandes[260], Pietro Ferrara[261], Manuela L Ferreira[262], Irina Filip[263 264], Florian Fischer[265], James L Fisher[266], Nataliya A Foigt[267],

Morenike Oluwatoyin Folayan[268], Artem Alekseevich Fomenkov[269], Masoud Foroutan[270], Richard Charles Franklin[271], Marisa Freitas[260], Mohamed M Gad[272 273], Silvano Gallus[274], Amiran Gamkrelidze[275], Abadi Kahsu Gebre[276 277], Leake G Gebremeskel[278 279], Fatemeh Ghaffarifar[280], Mansour Ghafourifard[281], Mahsa Ghajarzadeh[282], Reza Ghanei Gheshlagh[283], Ahmad Ghashghaee[60 284], Nermin Ghith[285], Asadollah Gholamian[286 287], Tiffany K Gill[197], Richard F Gillum[288 289], Iago Giné-Vázquez[290 2], Giorgia Giussani[291], Mustefa Glagn[292], Elena V Gnedovskaya[293], Myron Anthony Godinho[294], Salime Goharinezhad[97], Sameer Vali Gopalani[295 296], Giuseppe Gorini[297], Alessandra C Goulart[298 128], Michal Grivna[299 300], Harish Chander Gugnani[301 302], Rafael Alves Guimarães[303], Yuming Guo[188 304], Rahul Gupta[305 306], Reyna Alma Gutiérrez[307], Abdul Hafiz[308 309], Arvin Haj-Mirzaian[310 311], Arya Haj-Mirzaian[312], Randah R Hamadeh[313], Samer Hamidi[314], Graeme J Hankey[315 316], Hamidreza Haririan[317], Josep Maria Haro[318 319], Ahmed I Hasaballah[320], Maryam Hashemian[321 322], Abdiwahab Hashi[323], Shoaib Hassan[324 325], Amr Hassan[11], Soheil Hassanipour[326 327], Simon I Hay[6 9], Khezar Hayat[328 329], Reza Heidari-Soureshjani[330], Delia Hendrie[104], Claudiu Herteliu[80 331], Hung Chak Ho[332], Chi Linh Hoang[333], Ramesh Holla[334], Praveen Hoogar[335], Naznin Hossain[336], Mostafa Hosseini[337 338], Mehdi Hosseinzadeh[339 340], Mihaela Hostiuc[341], Sorin Hostiuc[342 343], Mowafa Househ[344], Mohamed Hsairi[345], Guoqing Hu[346], Ayesha Humayun[347], Bing-Fang Hwang[348], Ivo Iavicoli[349], Segun Emmanuel Ibitoye[350], Olayinka Stephen Ilesanmi[351 352], Irena M Ilic[353], Milena D Ilic[354], Leeberk Raja Inbaraj[355], Usman Iqbal[356], Seyed Sina Naghibi Irvani[357], Md.Mohaimenul Islam[358], Hiroyasu Iso[359], Rebecca Q Ivers[360], Chinwe Juliana Iwu[361 26], Chidozie C D Iwu[362], Jalil Jaafari[363], Mohammad Ali Jahani[139], Mihajlo Jakovljevic[364 365], Farzad Jalilian[366], Hosna Janjani[256 367], Manthan Dilipkumar Janodia[368], Tahereh Javaheri[369], Ensiyeh Jenabi[370], Ravi Prakash Jha[371 372], John S Ji[373 374], Oommen John[375 376], Jost B Jonas[377 378], Jacek Jerzy Jozwiak[379], Mikk Jürisson[380], Ali Kabir[381], Zubair Kabir[382], Rizwan Kalani[383], Rohollah Kalhor[384 385], Tanuj Kanchan[386], Neeti Kapoor[108], Behzad Karami Matin[387], André Karch[388], Ayele Semachew Kasa[389], Gbenga A Kayode[390 391], Ali Kazemi Karyani[387], Yousef Saleh Khader[392], Nauman Khalid[393], Mohammad Khammarnia[394], Maseer Khan[395], Ejaz Ahmad Khan[396], Khaled Khatab[397 398], Mahalaqua Nazli Khatib[399], Maryam Khayamzadeh[400 401], Habibolah Khazaie[402], Abdullah T Khoja[403 404], Young-Eun Kim[405], Yun Jin Kim[406], Ruth W Kimokoti[407], Sezer Kisa[408], Adnan Kisa[409 410], Mika Kivimäki[411 412], Cameron J Kneib[190], Sonali Kochhar[4 413], Ali Koolivand[414], Jacek A Kopec[415 416], Anirudh Kotlo[417], Ai Koyanagi[418 419], Kewal Krishan[420], Barthelemy Kuate Defo[421 422], G Anil Kumar[205], Nithin Kumar[423], Manasi Kumar[424 425], Om P Kurmi[426 427], Dian Kusuma[428 429], Ben Lacey[430 431], Dharmesh Kumar Lal[205], Ratilal Lalloo[432], Tea Lallukka[412], Jennifer O Lam[433], Faris Hasan Lami[434], Iván Landires[435 436], Van Charles Lansingh[437 438], Anders O Larsson[439 440], Savita Lasrado[441], Paolo Lauriola[442], Carlo La Vecchia[443], Janet L Leasher[444], Georgy Lebedev[445 446], Paul H Lee[447], Shaun Wen Huey Lee[448 449], James Leigh[450], Matilde Leonardi[451], Andrew S Levey[452 453], Miriam Levi[454 455], Shanshan Li[456], Shai Linn[457], Xuefeng Liu[458], Alan D Lopez[459], Platon D Lopukhov[153], Stefan Lorkowski[460 461], Paulo A Lotufo[462], Rafael Lozano[6 9], Alessandra Lugo[274], Raimundas Lunevicius[463 464], Mohammed Madadin[465], Ralph Maddison[466], Phetole Walter Mahasha[467], Morteza Mahmoudi[468], Azeem Majeed[164], Shokofeh Maleki[469], Afshin Maleki[470], Reza Malekzadeh[322 471], Deborah Carvalho Malta[472], Abdullah A Mamun[473], Navid Manafi[474 475], Fariborz Mansour-Ghanaei[326 327], Borhan Mansouri[476], Mohammad Ali Mansournia[337], Santi Martini[477 478], Francisco Rogerlândio Martins-Melo[479], Seyedeh Zahra Masoumi[480], João Massano[481 482], Pallab K Maulik[483 376], Mohsen Mazidi[484], John J McGrath[485 486], Martin McKee[487], Man Mohan Mehndiratta[488 489], Fereshteh Mehri[490], Fabiola Mejia-Rodriguez[215], Walter Mendoza[491], Ritesh G Menezes[492], George A Mensah[493 494], Alibek Mereke[495 212], Tuomo J Meretoja[496 497], Atte Meretoja[498 499], Tomislav Mestrovic[500 501], Tomasz Miazgowski[502], Irmina Maria Michalek[503], Ted R Miller[504 104], Edward J Mills[505], Andreea Mirica[80], Erkin M Mirrakhimov[506 507], Hamed Mirzaei[508], Maryam Mirzaei[509], Mehdi Mirzaei-Alavijeh[366], Philip B Mitchell[510], Babak Moazen[186 511], Masoud Moghadaszadeh[512 513], Efat Mohamadi[514], Yousef Mohammad[515], Dara K Mohammad[516 517], Shadieh Mohammadi[470 518], Abdollah Mohammadian-Hafshejani[519], Shafiu Mohammed[520 186], Ali H Mokdad[6 9], Alex Molassiotis[447], Natalie C Momen[486], Stefania Mondello[521], Masoud Moradi[387], Maziar Moradi-Lakeh[97], Farhad Moradpour[522], Rahmatollah Moradzadeh[67], Paula Moraga[523], Lidia Morawska[524], Rintaro Mori[525 526], Seyyed Meysam

Mousavi[527], Amin Mousavi Khaneghah[528], Ulrich Otto Mueller[529 530], Satinath Mukhopadhyay[531], Moses K Muriithi[532], Mehdi Naderi[469], Ahamarshan Jayaraman Nagarajan[533 534], Behshad Naghshtabrizi[535], Mukhammad David Naimzada[536 537], Farid Najafi[538 387], Jobert Richie Nansseu[539 540], Rawlance Ndejjo[541], Ionut Negoi[542 543], Ruxandra Irina Negoi[544 545], Subas Neupane[546], Georges Nguefack-Tsague[540], Josephine W Ngunjiri[547], Cuong Tat Nguyen[548], Huong Lan Thi Nguyen[548], Rajan Nikbakhsh[311], Chukwudi A Nnaji[361 549], Marzieh Nojomi[97 550], Shuhei Nomura[551 552], Bo Norrving[553], Jean Jacques Noubiap[554], Christoph Nowak[555], Virginia Nunez-Samudio[556 557], Felix Akpojene Ogbo[558], In-Hwan Oh[559], Morteza Oladnabi[560], Andrew T Olagunju[561 562], Jacob Olusegun Olusanya[563], Bolajoko Olubukunola Olusanya[563], Muktar Omer Omer[323], Obinna E Onwujekwe[564], Sergej M Ostojic[565], Adrian Oțoiu[80], Nikita Otstavnov[536], Stanislav S Otstavnov[536 566], Mayowa O Owolabi[567 568], Mahesh P A[569], Jagadish Rao Padubidri[570], Songhomitra Panda-Jonas[571], Anamika Pandey[572], Carlo Irwin Able Panelo[573], Deepak Kumar Pasupula[574], Hamidreza Pazoki Toroudi[575 576], Jonathan Pearson-Stuttard[117], Amy E Peden[360 577], Veincent Christian Filipino Pepito[578], Emmanuel K Peprah[579], Jeevan Pereira[580], Konrad Pesudovs[581], Hai Quang Pham[548], Michael R Phillips[582 583], Thomas Pilgrim[584], Marina Pinheiro[585], Michael A Piradov[258], Meghdad Pirsaheb[387], Roman V Polibin[153], Suzanne Polinder[586], Maarten J Postma[587 588], Hadi Pourjafar[589 590], Akram Pourshams[322], Sergio I Prada[591 592], Sanjay Prakash[593], Dimas Ria Angga Pribadi[594], Elisabetta Pupillo[124], Zahiruddin Quazi Syed[595], Navid Rabiee[596], Amir Radfar[597], Ata Rafiee[598], Alberto Raggi[451], Fakher Rahim[599 600], Vafa Rahimi-Movaghar[601], Mohammad Hifz Ur Rahman[602], Muhammad Aziz Rahman[603 236], Kiana Ramezanzadeh[604], Chhabi Lal Ranabhat[605 606], Annemarei Ranta[607 608], Sowmya J Rao[609], Vahid Rashedi[610], Prateek Rastogi[611], Priya Rathi[334], Salman Rawaf[164 612], David Laith Rawaf[613 614], Lal Rawal[615], Reza Rawassizadeh[616], Andre M N Renzaho[617 618], Bhageerathy Reshmi[619 620], Serge Resnikoff[581 621], Nima Rezaei[16 622], Negar Rezaei[70 31], Aziz Rezapour[60], Seyed Mohammad Riahi[623], Daniela Ribeiro[624], Ana Isabel Ribeiro[625], Jennifer Rickard[626 627], Leonardo Roever[628], Michele Romoli[629 630], Dietrich Rothenbacher[631], Enrico Rubagotti[632], Susan Fred Rumisha[633 634], Seyedmohammad Saadatagah[635], Siamak Sabour[636], Perminder S Sachdev[637 638], Masoumeh Sadeghi[639], Ehsan Sadeghi[387], Sahar Saeedi Moghaddam[70], Rajesh Sagar[640], Mohammad Ali Sahraian[248], S. Mohammad Sajadi[641 642], Nasir Salam[643], Marwa Rashad Salem[644], Hamideh Salimzadeh[322], Omar Mukhtar Salman[645], Abdallah M Samy[646], Juan Sanabria[647 648], Lidia Sanchez Riera[649 262], Itamar S Santos[128 298], Milena M Santric-Milicevic[353 650], Sivan Yegnanarayana Iyer Saraswathy[651 652], Arash Sarveazad[653], Brijesh Sathian[654 655], Thirunavukkarasu Sathish[656], Davide Sattin[451], Silvia Schiavolin[451], Maria Inês Schmidt[199], Aletta Elisabeth Schutte[360 657], David C Schwebel[658], Falk Schwendicke[659], Subramanian Senthilkumaran[660], Sadaf G Sepanlou[322 471], Feng Sha[661], Omid Shafaat[312 662], Saeed Shahabi[663], Amira A Shaheen[664], Masood Ali Shaikh[665], Marina Shakhnazarova[666], Mehran Shams-Beyranvand[667], MohammadBagher Shamsi[509], Morteza Shamsizadeh[668], Kiomars Sharafi[387], B Suresh Kumar Shetty[611], Kenji Shibuya[669], Wondimeneh Shibabaw Shiferaw[106], Mika Shigematsu[670], Jae Il Shin[671], Rahman Shiri[672], Reza Shirkoohi[673 674], Kerem Shuval[457], Inga Dora Sigfusdottir[675 676], Rannveig Sigurvinsdottir[675], João Pedro Silva[168], Biagio Simonetti[677 678], Jasvinder A Singh[679 680], Pushpendra Singh[681], Ambrish Singh[89], Dhirendra Narain Sinha[682 683], Søren T Skou[684 685], Valentin Yurievich Skryabin[686], Emma U R Smith[687 688], Mohammad Reza Sobhiyeh[689], Amin Soheili[690], Shahin Soltani[387], Joan B Soriano[691 692], Ireneous N Soyiri[693], Emma Elizabeth Spurlock[6], Chandrashekhar T Sreeramareddy[694], Dan J Stein[695], Leo Stockfelt[696], Mark A Stokes[697], Saverio Stranges[698 699], Jacob L Stubbs[700], Agus Sudaryanto[701], Mu'awiyyah Babale Sufiyan[702], Hafiz Ansar Rasul Suleria[703], Rizwan Suliankatchi Abdulkader[704 705], Gerhard Sulo[706], Rafael Tabarés-Seisdedos[707 708], Takahiro Tabuchi[709], Biruk Wogayehu Taddele[710], Amir Taherkhani[711], Masih Tajdini[71], Md Ismail Tareque[712], Yonas Getaye Tefera[713], Mohamad-Hani Temsah[714], Zemenu Tadesse Tessema[715], Kavumpurathu Raman Thankappan[716], Rekha Thapar[423], Amanda G Thrift[717], Mariya Vladimirovna Titova[718 719], Hamid Reza Tohidinik[720 337], Marcello Tonelli[721], Mathilde Touvier[722 723], Marcos Roberto Tovani-Palone[724 725], Bach Xuan Tran[726], Ravensara S Travillian[6], Alexander C Tsai[727 728], Aristidis Tsatsakis[729], Riaz Uddin[730 731], Saif Ullah[138], Chukwuma David Umeokonkwo[732], Bhaskaran Unnikrishnan[733], Marco Vacante[141], Pascual R Valdez[734 735], Aaron van Donkelaar[736 737], Santosh Varughese[738], Tommi Juhani Vasankari[739], Yasser

Vasseghian[339], Yousef Veisani[740], Narayanaswamy Venketasubramanian[741 742], Francesco S Violante[743 744], Vasily Vlassov[745], Yasir Waheed[746], Yanzhong Wang[747], Fang Wang[748], Yuan-Pang Wang[169], Jingkai Wei[749], Robert G Weintraub[750 751], Jordan Weiss[752], Ronny Westerman[753], Taweewat Wiangkham[754], Charles D A Wolfe[747 755], Ai-Min Wu[756], Seyed Hossein Yahyazadeh Jabbari[757], Kazumasa Yamagishi[758 759], Yuichiro Yano[760], Sanni Yaya[761 762], Vahid Yazdi-Feyzabadi[763 764], Yordanos Gizachew Yeshitila[765], Mohammed Zewdu Yimmer[766], Paul Yip[767 768], Naohiro Yonemoto[769 770], Seok-Jun Yoon[405], Mustafa Z Younis[771 772], Zabihollah Yousefi[773], Taraneh Yousefinezhadi[774], Chuanhua Yu[775 776], Yong Yu[777], Hasan Yusefzadeh[778], Syed Saoud Zaidi[779], Sojib Bin Zaman[780 191], Maryam Zamanian[67], Hadi Zarafshan[781], Mikhail Sergeevich Zastrozhin[782 783], Zhi-Jiang Zhang[784], Jianrong Zhang[785 786], Yunquan Zhang[787 788], Xiu-Ju George Zhao[789 748], Cong Zhu[790], Georgios A Kotsakis*[3 4], and Nicholas J Kassebaum*[& 4 6 9 791].

[%]First author
*Co-senior authors
[&]Corresponding author

1 St. John of God Health Park, San Juan de Dios Sanitary Park, Barcelona, Spain.

2 Biomedical Research Networking Center for Mental Health Network (CiberSAM), Madrid, Spain.

3 Department of Periodontics, University of Texas Health Science Center at San Antonio, San Antonio, TX, USA.

4 Department of Global Health, University of Washington, Seattle, WA, USA.

5 Department of Pharmacy, University of Washington, Seattle, WA, USA.

6 Institute for Health Metrics and Evaluation, University of Washington, Seattle, WA, USA.

7 University of Washington, Seattle, WA, USA.

8 Centre for Fertility and Health, Norwegian Institute of Public Health, Oslo, Norway.

9 Department of Health Metrics Sciences, School of Medicine, University of Washington, Seattle, WA, USA.

10 Division of Data, Analytics and Delivery for Impact, World Health Organization , Geneva, Switzerland.

11 Department of Neurology, Cairo University, Cairo, Egypt.

12 The Institute of Pharmaceutical Sciences (TIPS), Tehran University of Medical Sciences, Tehran, Iran.

13 School of Pharmacy, Tehran University of Medical Sciences, Tehran, Iran.

14 Department of Orthopaedic Surgery, University of Southern California, Los Angeles, CA, USA.

15 Department of Laboratory Medicine, Karolinska University Hospital, Huddinge, Sweden.

16 Research Center for Immunodeficiencies, Tehran University of Medical Sciences, Tehran, Iran.

17 Department of Public Health, Debre Berhan University, Debre Brehan, Ethiopia.

18 Department of Pediatric Dentistry, Federal University of Minas Gerais, Belo Horizonte, Brazil.

19 Department of Research, Philippine Institute for Development Studies, Quezon City, Philippines.

20 Internal Medicine Department, Imam Abdulrahman Bin Faisal University, Dammam, Saudi Arabia.

21 Hamadan University of Medical Sciences, Hamadan, Iran.

22 College of Medicine, University College Hospital, Ibadan, Ibadan, Nigeria.

23 Department of Sociology, Olabisi Onabanjo University, Ago-Iwoye, Nigeria.

24 Department of Population Health Sciences, King's College London, London, England.

25 Centre of Excellence for Epidemiological Modelling and Analysis, Stellenbosch University, Stellenbosch, South Africa.

26 Department of Global Health, Stellenbosch University, Cape Town, South Africa.
27 School of Health, Ardabil University of Medical Science, Ardabil, Iran.
28 Social Behavioral Research Branch, National Institute of Health, Bethesda, MD, USA.
29 Division of Hematology and Oncology, Georgetown University, Washington, DC, USA.
30 Department of Dermatology, Mayo Clinic, Rochester, MN, USA.
31 Endocrinology and Metabolism Research Center, Tehran University of Medical Sciences, Tehran, Iran.
32 Department of Family Medicine, McMaster University, Hamilton, ON, Canada.
33 Department of Epidemiology and Biostatistics, Qom University of Medical Sciences, Qom, Iran.
34 Urology Research Center, Tehran University of Medical Sciences, Tehran, Iran.
35 Institute of Genomics and Integrative Biology, Council of Scientific & Industrial Research, Delhi, India.
36 Department of Internal Medicine, Baylor College of Medicine, Houston, TX, USA.
37 Faculty of Pharmacy, MAHSA University, Kuala Langat, Malaysia.
38 Department of Epidemiology and Health Statistics, Southeast University, Nanjing, China.
39 Lincoln Medical School, Universities of Nottingham & Lincoln, Lincoln, UK.
40 Environmental Technologies Research Center, Ahvaz Jundishapur University of Medical Sciences, Ahvaz, Iran.
41 Department of Environmental Health Engineering, Ahvaz Jundishapur University of Medical Sciences, Ahvaz, Iran.
42 Department of Epidemiology, Jimma University, Jimma, Ethiopia.
43 Australian Center for Precision Health, University of South Australia, Adelaide, SA, Australia.
44 Institute for Advanced Medical Research and Training, University of Ibadan, Ibadan, Nigeria.
45 Institute of Neuroscience, Newcastle University, Newcastle upon Tyne, UK.
46 John T. Milliken Department of Internal Medicine, Washington University in St. Louis, St. Louis, MO, USA.
47 Clinical Epidemiology Center, Department of Veterans Affairs, St Louis, MO, USA.
48 Murdoch Business School, Murdoch University, Perth, WA, Australia.
49 Imam Abdulrahman Bin Faisal University, Dammam, Saudi Arabia.
50 Health Information Management and Technology Department, Imam Abdulrahman Bin Faisal University, Dammam, Saudi Arabia.
51 Center for Health System Research, National Institute of Public Health, Cuernavaca, Mexico.
52 College of Medicine and Health Science, Arba Minch University, Arba Minch, Ethiopia.
53 Department of Midwifery, Arba Minch University, Injbara, Ethiopia.
54 Faculty of Health Sciences, Health Management and Policy, American University of Beirut, Beirut, Lebanon.
55 British Columbia Injury Research Prevention Unit, British Columbia Children's Hospital Research Institute, Vancouver, BC, Canada.
56 Institute of Health Research, University of Health and Allied Sciences, Ho, Ghana.
57 Department of Information Systems, Sultan Qaboos University, Muscat, Oman.
58 Directorate for Social Statistics and Population Census, Italian National Institute of Statistics, Rome, Italy.

59 Social Determinants of Health Research Center, Qazvin University of Medical Sciences, Qazvin, Iran.
60 Health Management and Economics Research Center, Iran University of Medical Sciences, Tehran, Iran.
61 Department of Health Economics, Iran University of Medical Sciences, Tehran, Iran.
62 Department of Health Policy and Management, Kuwait University, Safat, Kuwait.
63 International Centre for Casemix and Clinical Coding, National University of Malaysia, Bandar Tun Razak, Malaysia.
64 Bordeaux School of Public Health, University of Bordeaux, Bordeaux, France.
65 College of Medicine, King Saud University, Riyadh, Saudi Arabia.
66 Division of Gastroenterology & Hepatology, McGill University, Montreal, QC, Canada.
67 Department of Epidemiology, Arak University of Medical Sciences, Arak, Iran.
68 College of Medicine, Imam Abdulrahman Bin Faisal University, Dammam, Saudi Arabia.
69 Department of Community Medicine, King Abdulaziz University, Jeddah, Saudi Arabia.
70 Non-communicable Diseases Research Center, Tehran University of Medical Sciences, Tehran, Iran.
71 Tehran Heart Center, Tehran University of Medical Sciences, Tehran, Iran.
72 Department of Radiology and Nuclear Medicine, Kermanshah University of Medical Sciences, Kermanshah, Iran.
73 Department of Epidemiology and Biostatistics, University of the Philippines Manila, Manila, Philippines.
74 School of Public Health, Johns Hopkins University, Baltimore, MD, USA.
75 Maternal and Child Wellbeing Unit, African Population and Health Research Center, Nairobi, Kenya.
76 Department of Public Health, Samara University, Samara, Ethiopia.
77 Department of Pharmacy, Carol Davila University of Medicine and Pharmacy, Bucharest, Romania.
78 Centre for Sensorimotor Performance, The University of Queensland, Brisbane, Australia.
79 Neurology Department, Royal Brisbane and Women’s Hospital, Brisbane, QLD, Australia.
80 Department of Statistics and Econometrics, Bucharest University of Economic Studies, Bucharest, Romania.
81 Department of Cardiology, Carol Davila University of Medicine and Pharmacy, Bucharest, Romania.
82 Department of Medicine, University of Thessaly, Volos, Greece.
83 Social Determinants of Health Research Center, Rafsanjan University of Medical Sciences, Rafsanjan, Iran.
84 Research Center for Evidence Based Medicine, Tabriz University of Medical Sciences, Tabriz, Iran.
85 Razi Vaccine and Serum Research Institute, Agricultural Research, Education, and Extension Organization (AREEO), Tehran, Iran.
86 Department of Epidemiology and Biostatistics, Zahedan University of Medical Sciences, Zahedan, Iran.
87 Department of Health Policy and Administration, University of the Philippines Manila, Manila, Philippines.

88 Department of Applied Social Sciences, Hong Kong Polytechnic University, Hong Kong, China.
89 Menzies Institute for Medical Research, University of Tasmania, Hobart, TAS, Australia.
90 Department of Parasitology, Mazandaran University of Medical Sciences, Sari, Iran.
91 Department of Parasitology, Iranshahr University of Medical Sciences, Iranshahr, Iran.
92 Department of Pathology, Imam Mohammad Ibn Saud Islamic University, Riyadh, Saudi Arabia.
93 Social Determinants of Health Research Center, Birjand University of Medical Sciences, Birjand, Iran.
94 Department of Neurobiology, Care Sciences and Society, Karolinska Institutet, Stockholm, Sweden.
95 School of Health and Social Studies, Dalarna University, Falun, Sweden.
96 Department of Plastic Surgery, University of Texas, Houston, TX, USA.
97 Preventive Medicine and Public Health Research Center, Iran University of Medical Sciences, Tehran, Iran.
98 Epilepsy Research Center, Shiraz University of Medical Sciences, Shiraz, Iran.
99 Department of Neurology, Thomas Jefferson University, Philadelphia, PA, USA.
100 Faculty of Nursing, Philadelphia University, Amman, Jordan.
101 School of Business, University of Leicester, Leicester, UK.
102 Gastro-enterology Department, University of Liège, Liège, Belgium.
103 The Judith Lumley Centre, La Trobe University, Melbourne, VIC, Australia.
104 School of Public Health, Curtin University, Perth, WA, Australia.
105 Department of Health Policy Planning and Management, University of Health and Allied Sciences, Ho, Ghana.
106 Department of Nursing, Debre Berhan University, Debre Berhan, Ethiopia.
107 Department of Reproductive Health, University of Gondar, Gondar, Ethiopia.
108 Department of Forensic Science, Government Institute of Forensic Science, Nagpur, India.
109 Department of Demography, University of Groningen, Groningen, Netherlands.
110 Population Research Centre, Institute for Social and Economic Change, Bengaluru, India.
111 Department of Hypertension, Medical University of Lodz, Lodz, Poland.
112 Polish Mothers' Memorial Hospital Research Institute, Lodz, Poland.
113 School of Health Sciences, Walden University, Minneapolis, MN, USA.
114 Department of Non-communicable Diseases, Bangladesh University of Health Sciences, Dhaka, Bangladesh.
115 School of Psychology, University of Auckland, Auckland, New Zealand.
116 Center for Primary Care, Harvard University, Boston, MA, USA.
117 School of Public Health, Imperial College London, London, UK.
118 Department of Psychiatry, University of Münster, Münster, Germany.
119 Department of Psychiatry, Melbourne Medical School, Melbourne, Australia.
120 Health Human Resources Research Center, Shiraz University of Medical Sciences, Shiraz, Iran.
121 Department of Public Health, Ambo University, Ambo, Ethiopia.
122 Department of Community Medicine, Gandhi Medical College Bhopal, Bhopal, India.
123 Jazan University, Jazan, Saudi Arabia.
124 Department of Neuroscience, Mario Negri Institute for Pharmacological Research, Milan, Italy.

125 Department of Neurology, University Hospital of Dijon, Dijon, France.
126 Dijon Stroke Registry - UFR Sciences Santé, University of Burgundy, Dijon, France.
127 School of the Environment, Yale University, New Haven, CT, USA.
128 Department of Internal Medicine, University of São Paulo, São Paulo, Brazil.
129 Department of Internal Medicine, United Arab Emirates University, Al Ain, United Arab Emirates.
130 Department of Social and Clinical Pharmacy, Charles University, Prague, Czech Republic.
131 Department of Community Medicine and Family Medicine, All India Institute of Medical Sciences, Jodhpur, India.
132 School of Public Health, All India Institute of Medical Sciences, Jodhpur, India.
133 Department of Statistical and Computational Genomics, National Institute of Biomedical Genomics, Kalyani, India.
134 Department of Statistics, University of Calcutta, Kolkata, India.
135 Department of Global Health, Global Institute for Interdisciplinary Studies, Kathmandu, Nepal.
136 Centre for Global Child Health, University of Toronto, Toronto, ON, Canada.
137 Centre of Excellence in Women & Child Health, Aga Khan University, Karachi, Pakistan.
138 Institute of Soil and Environmental Sciences, University of Agriculture, Faisalabad, Faisalabad, Pakistan.
139 Social Determinants of Health Research Center, Babol University of Medical Sciences, Babol, Iran.
140 Mario Negri Institute for Pharmacological Research, Ranica, Italy.
141 Department of General Surgery and Medical-Surgical Specialties, University of Catania, Catania, Italy.
142 Ethiopian Public Health Institute, Addis Ababa, Ethiopia.
143 Department of Nursing, Debre Tabor University, Debretabor, Ethiopia.
144 Department of Neurology, Institute of Post-Graduate Medical Education and Research and Seth Sukhlal Karnani Memorial Hospital, Kolkata, India.
145 Department of Global Public Health and Primary Care, University of Bergen, Bergen, Norway.
146 Cancer Registry of Norway, Oslo, Norway.
147 Department of Veterinary Medicine, Islamic Azad University, Kermanshah, Iran.
148 Department of Biomedical Sciences, Nazarbayev University, Nur-Sultan City, Kazakhstan.
149 Department of Internal Medicine, Manipal Academy of Higher Education, Mangalore, India.
150 Transport and Road Safety (TARS) Research Centre, University of New South Wales, Sydney, NSW, Australia.
151 Division of Clinical Epidemiology and Aging Research, German Cancer Research Center, Heidelberg, Germany.
152 School of Physiotherapy and Exercise Science, Curtin University, Perth, WA, Australia.
153 Department of Epidemiology and Evidence-Based Medicine, I.M. Sechenov First Moscow State Medical University, Moscow, Russia.
154 Neuroscience Unit, Institute for Scientific Research and High Technology Services, Panama City, Panama.
155 Gorgas Memorial Institute for Health Studies, Panama City, Panama.
156 Department of Community Medicine, Employee State Insurance Post Graduate Institute of Medical Sciences and Research, Bangalore, India.
157 Department of Health Care Management, Technical University of Berlin, Berlin, Germany.

158 School of Public Health and Health Systems, University of Waterloo, Waterloo, ON, Canada.
159 Al Shifa School of Public Health, Al Shifa Trust Eye Hospital, Rawalpindi, Pakistan.
160 Institute of Microengineering, Federal Polytechnic School of Lausanne, Lausanne, Switzerland.
161 Internal Medicine Department, Hospital Italiano de Buenos Aires, Buenos Aires, Argentina.
162 Board of Directors, Argentine Society of Medicine, Buenos Aires, Argentina.
163 Centre for Population Health Sciences, Nanyang Technological University, Singapore, Singapore.
164 Department of Primary Care and Public Health, Imperial College London, London, UK.
165 Department of Health Care, Metropolitan Autonomous University, Mexico City, Mexico.
166 Institute for Cancer Research, Prevention and Clinical Network, Florence, Italy.
167 Department of Medical Epidemiology and Biostatistics, Karolinska Institutet, Stockholm, Sweden.
168 Research Unit on Applied Molecular Biosciences (UCIBIO), University of Porto, Porto, Portugal.
169 Department of Psychiatry, University of São Paulo, São Paulo, Brazil.
170 Colombian National Health Observatory, National Institute of Health, Bogota, Colombia.
171 Epidemiology and Public Health Evaluation Group, National University of Colombia, Bogota, Colombia.
172 Department of Medicine, University of Udine, Udine, Italy.
173 Department of Mental Health, Healthcare Agency 'Friuli Occidentale', Pordenone, Italy.
174 Mary MacKillop Institute for Health Research, Australian Catholic University, Melbourne, VIC, Australia.
175 School of Public Health, University of Hong Kong, Hong Kong, China.
176 Infection and Global Health Research, University of St Andrews, St Andrews, UK.
177 Regional Infectious Diseases Unit, NHS National Services Scotland, Edinburgh, UK.
178 Institute of Applied Health Research, University of Birmingham, Birmingham, UK.
179 Kidney Health Research Institute, Geisinger Health System, Danville, PA, USA.
180 Department of Pharmacology, All India Institute of Medical Sciences, Jodhpur, India.
181 Department of Medicine, University of Toronto, Toronto, ON, Canada.
182 Center for Cancer Epidemiology, Tata Memorial Hospital, Navi Mumbai, India.
183 Department of Head Neck Surgery, Tata Memorial Hospital, Mumbai, India.
184 Research Department, Dr. D. Y. Patil University, Pune, India.
185 Department of Epidemiology and Environmental Health, University at Buffalo, Buffalo, TN, USA.
186 Heidelberg Institute of Global Health (HIGH), Heidelberg University, Heidelberg, Germany.
187 Research School of Population Health, Australian National University, Canberra, ACT, Australia.
188 Department of Epidemiology and Preventive Medicine, Monash University, Melbourne, VIC, Australia.
189 Melbourne Medical School, University of Melbourne, Parkville, VIC, Australia.
190 Division of Plastic Surgery, University of Washington, Seattle, WA, USA.
191 Maternal and Child Health Division, International Centre for Diarrhoeal Disease Research, Bangladesh, Dhaka, Bangladesh.
192 Department of Epidemiology and Biostatistics, University of South Carolina, Columbia, SC, USA.
193 Faculty of Biology, Hanoi National University of Education, Hanoi, Vietnam.
194 Department of Health Informatics, University College London, London, UK.
195 Health Data Research UK, London, UK.

196 Department of Otolaryngology, Wayne State University, Detroit, MI, USA.
197 Adelaide Medical School, University of Adelaide, Adelaide, SA, Australia.
198 School of Pharmacy and Medical Sciences, University of South Australia, Adelaide, SA, Australia.
199 Postgraduate Program in Epidemiology, Federal University of Rio Grande do Sul, Porto Alegre, Brazil.
200 Department of Family Medicine and Public Health, University of California San Diego, La Jolla, CA, USA.
201 Faculty of Medicine and Health, University of Sydney, Sydney, Australia.
202 Environmental Health Department, Imam Abdulrahman Bin Faisal University, Dammam, Saudi Arabia.
203 Clinical Dermatology, IRCCS Istituto Ortopedico Galeazzi, University of Milan, Milan, Italy.
204 Department of Dermatology, Case Western Reserve University, Cleveland, OH, USA.
205 Public Health Foundation of India, Gurugram, India.
206 Indian Council of Medical Research, New Delhi, India.
207 Department of Pathology, Johns Hopkins University School of Medicine, Baltimore, MD, USA.
208 Department of Pathology, Isfahan University of Medical Sciences, Isfahan, Iran.
209 Division of Women and Child Health, Aga Khan University, Karachi, Pakistan.
210 James P Grant School of Public Health, BRAC University, Dhaka, Bangladesh.
211 Department of Population and Development, Latin American Faculty of Social Sciences Mexico, Mexico City, Mexico.
212 Health Research Institute, Al Farabi Kazakh National University, Almaty, Kazakhstan.
213 Centre for Interdisciplinary Research in Basic Sciences, Jamia Millia Islamia, Delhi, India.
214 Australian Institute for Suicide Research and Prevention, Griffith University, Mount Gravatt, QLD, Australia.
215 Center for Nutrition and Health Research, National Institute of Public Health, Cuernavaca, Mexico.
216 St Paul’s Eye Unit, Royal Liverpool University Hospital, Liverpool, UK.
217 Department of Ophthalmology, Aristotle University of Thessaloniki, Thessaloniki, Greece.
218 Division of Cardiology, Atlanta Veterans Affairs Medical Center, Decatur, GA, USA.
219 Department of Community Medicine, University of Peradeniya, Peradeniya, Sri Lanka.
220 Department of Microbiology, Far Western University, Mahendranagar, Nepal.
221 Department of Epidemiology and Biostatistics, Shahroud University of Medical Sciences, Shahroud, Iran.
222 Department of Epidemiology, Shiraz University of Medical Sciences, Shiraz, Iran.
223 Laboratory of Toxicology, University of Porto, Porto, Portugal.
224 Center of Complexity Sciences, National Autonomous University of Mexico, Mexico City, Mexico.
225 Faculty of Veterinary Medicine and Zootechnics, Autonomous University of Sinaloa, Culiacan Rosales, Mexico.
226 Institute for Stroke and Dementia Research, Ludwig Maximilians University, Munich, Germany.
227 Development of Research and Technology Center, Ministry of Health and Medical Education, Tehran, Iran.
228 Department of Social Medicine and Health Care Organisation, Medical University of Varna, Varna, Bulgaria.

229 Department of Medical Laboratory Sciences, Iran University of Medical Sciences, Tehran, Iran.
230 Department of Health Policy and Management, Tabriz University of Medical Sciences, Tabriz, Iran.
231 School of Medicine, Federal University of Bahia, Salvador, Brazil.
232 Department of Internal Medicine, Escola Bahiana de Medicina e Saúde Pública (Bahiana School of Medicine and Public Health), Salvador, Brazil.
233 Department of Global Health and Social Medicine, Harvard University, Boston, MA, USA.
234 Department of Social Services, Tufts Medical Center, Boston, MA, USA.
235 Department of Epidemiology, Florida International University, Miami, FL, USA.
236 School of Nursing and Midwifery, La Trobe University, Melbourne, VIC, Australia.
237 Department of Nursing, Umeå University, Umea, Sweden.
238 Centre for Clinical Epidemiology and Biostatistics, University of Newcastle, Newcastle, NSW, Australia.
239 Department of Ophthalmology and Visual Sciences, University of Michigan, Ann Arbor, MI, USA.
240 Institute for Health Care Policy and Innovation, University of Michigan, Ann Arbor, MI, USA.
241 Division of Cardiology, Massachusetts General Hospital, Boston, MA, USA.
242 Division of Cardiology, Harvard University, Boston, MA, USA.
243 Biomedical Informatics and Medical Statistics Department, Alexandria University, Alexandria, Egypt.
244 Department of Clinical Pathology, Mansoura University, Mansoura, Egypt.
245 Pediatric Dentistry and Dental Public Health Department, Alexandria University, Alexandria, Egypt.
246 Department of Medicinal Chemistry, Kerman University of Medical Sciences, Kerman, Iran.
247 Pharmaceutics Research Center, Kerman University of Medical Sciences, Kerman, Iran.
248 Multiple Sclerosis Research Center, Tehran University of Medical Sciences, Tehran, Iran.
249 Department of Physiology, Tarbiat Modares University, Tehran, Iran.
250 Tehran Medical Sciences Branch, Islamic Azad University, Tehran, Iran.
251 Unit of Medical Physiology, Hawassa University, Hawassa, Ethiopia.
252 Department of Biology and Biotechnology 'Lazzaro Spallanzani', University of Pavia, Pavia, Italy.
253 Department of Biology, Cihan University-Erbil, Erbil, Iraq.
254 Department of Psychology, Federal University of Sergipe, São Cristóvão, Brazil.
255 Department of Environmental Health Engineering, Ardabil University of Medical Science, Ardabil, Iran.
256 Department of Environmental Health Engineering, Tehran University of Medical Sciences, Tehran, Iran.
257 National Institute for Stroke and Applied Neurosciences, Auckland University of Technology, Auckland, New Zealand.
258 Research Center of Neurology, Moscow, Russia.
259 Division of Neurology, University of Ottawa, Ottawa, ON, Canada.
260 Associated Laboratory for Green Chemistry (LAQV), University of Porto, Porto, Portugal.
261 Research Center on Public Health, University of Milan Bicocca, Monza, Italy.
262 Institute of Bone and Joint Research (IBJR), University of Sydney, Sydney, NSW, Australia.
263 Psychiatry Department, Kaiser Permanente, Fontana, CA, USA.
264 School of Health Sciences, A.T. Still University, Mesa, AZ, USA.

265 Institute of Gerontological Health Services and Nursing Research, Ravensburg-Weingarten University of Applied Sciences, Weingarten, Germany.
266 James Cancer Hospital, Ohio State University, Columbus, OH, USA.
267 Institute of Gerontology, National Academy of Medical Sciences of Ukraine, Kyiv, Ukraine.
268 Department of Child Dental Health, Obafemi Awolowo University, Ile-Ife, Nigeria.
269 Department of Cell Biology and Biotechnology, Timiryazev Institute of Plant Physiology, Moscow, Russia.
270 Department of Medical Parasitology, Abadan Faculty of Medical Sciences, Abadan, Iran.
271 School of Public Health, Medical, and Veterinary Sciences, James Cook University, Douglas, QLD, Australia.
272 Department of Cardiovascular Medicine, Cleveland Clinic, Cleveland, OH, USA.
273 Gillings School of Global Public Health, University of North Carolina Chapel Hill, Chapel Hill, NC, USA.
274 Department of Environmental Health Sciences, Mario Negri Institute for Pharmacological Research, Milan, Italy.
275 National Center for Disease Control and Public Health, Tbilisi, Georgia.
276 School of Pharmacy, Mekelle University, Mekelle, Ethiopia.
277 School of Medical and Health Sciences, Edith Cowan University, Perth, Australia.
278 School of Pharmacy, Aksum University, Aksum, Ethiopia.
279 Department of Pharmacy, Mekelle University, Mekelle, Ethiopia.
280 Department of Parasitology and Entomology, Tarbiat Modares University, Tehran, Iran.
281 Department of Medical Surgical Nursing, Tabriz University of Medical Sciences, Tabriz, Iran.
282 Department of Neurology, Tehran University of Medical Sciences, Tehran, Iran.
283 Faculty of Nursing and Midwifery, Kurdistan University of Medical Sciences, Sanandaj, Iran.
284 Student Research Committee, Iran University of Medical Sciences, Tehran, Iran.
285 Research Group for Genomic Epidemiology, Technical University of Denmark, Copenhagen, Denmark.
286 Young Researchers and Elite Club, Islamic Azad University, Rasht, Iran.
287 Department of Biology, Islamic Azad University, Tehran, Iran.
288 Division of General Internal Medicine, Howard University, Washington, DC, USA.
289 Department of Community and Family Medicine, Howard University, Washington, DC, USA.
290 Research, Innovation and Teaching Unit, San Juan de Dios Sanitary Park, Sant Boi de Llobregat, Spain.
291 Laboratory of Neurological Disorders, Mario Negri Institute for Pharmacological Research, Milan, Italy.
292 Department of Public Health, Arba Minch University, Arba Minch, Ethiopia.
293 Third Department of Neurology, Research Center of Neurology, Moscow, Russia.
294 School of Public Health and Community Medicine, University of New South Wales, Kensington, NSW, Australia.
295 Hudson College of Public Health, University of Oklahoma Health Sciences Center, Oklahoma City, OK, USA.
296 Department of Health and Social Affairs, Government of the Federated States of Micronesia, Palikir, Federated States of Micronesia.

297 Oncological Network, Prevention and Research Institute, Institute for Cancer Research, Prevention and Clinical Network, Florence, Italy.
298 Center for Clinical and Epidemiological Research, University of São Paulo, São Paulo, Brazil.
299 Institute of Public Health Department, United Arab Emirates University, Al Ain, United Arab Emirates.
300 Department of Public Health and Preventive Medicine, Charles University, Prague, Czech Republic.
301 Department of Microbiology, Saint James School of Medicine, The Valley, Anguilla.
302 Department of Epidemiology, Saint James School of Medicine, The Valley, Anguilla.
303 Institute of Tropical Pathology and Public Health (IPTSP), Federal University of Goias, Goiânia, Brazil.
304 Department of Epidemiology, Binzhou Medical University, Yantai City, China.
305 Medical Resources Department, March of Dimes, Arlington, VA, USA.
306 Department of Health Policy, Management and Leadership, West Virginia University School of Public Health, Morgantown, WV.
307 Department of Epidemiology and Psychosocial Research, Ramón de la Fuente Muñiz National Institute of Psychiatry , Mexico City, Mexico.
308 College of Medicine, Umm Al Qura University, Makkah, Saudi Arabia.
309 MSc Epidemiology Programme, London School of Hygiene & Tropical Medicine, London, UK.
310 Department of Pharmacology, Tehran University of Medical Sciences, Tehran, Iran.
311 Obesity Research Center, Shahid Beheshti University of Medical Sciences, Tehran, Iran.
312 Department of Radiology and Radiological Science, Johns Hopkins University, Baltimore, MD, USA.
313 Department of Family and Community Medicine, Arabian Gulf University, Manama, Bahrain.
314 School of Health and Environmental Studies, Hamdan Bin Mohammed Smart University, Dubai, United Arab Emirates.
315 Medical School, University of Western Australia, Perth, WA, Australia.
316 Department of Neurology, Sir Charles Gairdner Hospital, Perth, WA, Australia.
317 Tabriz University of Medical Sciences, Tabriz, Iran.
318 Research Unit, University of Barcelona, Sant Boi de Llobregat, Barcelona, Spain.
319 Biomedical Research Networking Center for Mental Health Network (CiberSAM), Barcelona, Spain.
320 Department of Zoology and Entomology, Al Azhar University, Cairo, Egypt.
321 Biology Department, Utica College, Utica, NY, USA.
322 Digestive Diseases Research Institute, Tehran University of Medical Sciences, Tehran, Iran.
323 Department of Public Health, Jigjiga University, Jijiga, Ethiopia.
324 Center for International Health (CIH), University of Bergen, Bergen, Norway.
325 Bergen Center for Ethics and Priority Setting (BCEPS), University of Bergen, Bergen, Norway.
326 Gastrointestinal and Liver Diseases Research Center, Guilan University of Medical Sciences, Rasht, Iran.
327 Caspian Digestive Disease Research Center, Guilan University of Medical Sciences, Rasht, Iran.
328 Institute of Pharmaceutical Sciences, University of Veterinary and Animal Sciences, Lahore, Pakistan.

329 Department of Pharmacy Administration and Clinical Pharmacy, Xian Jiaotong University, Xian, China.
330 School of Nursing and Midwifery, Tehran University of Medical Sciences, Tehran, Iran.
331 School of Business, London South Bank University, London, UK.
332 Department of Urban Planning and Design, University of Hong Kong, Hong Kong, China.
333 Center of Excellence in Behavioral Medicine, Nguyen Tat Thanh University, Ho Chi Minh City, Vietnam.
334 Kasturba Medical College, Manipal Academy of Higher Education, Manipal, India.
335 Centre for Bio Cultural Studies (CBiCS), Manipal Academy of Higher Education, Manipal, India.
336 Department of Pharmacology, Bangladesh Industrial Gases Limited, Tangail, Bangladesh.
337 Department of Epidemiology and Biostatistics, Tehran University of Medical Sciences, Tehran, Iran.
338 Pediatric Chronic Kidney Disease Research Center, Tehran University of Medical Sciences, Tehran, Iran.
339 Institute of Research and Development, Duy Tan University, Da Nang, Vietnam.
340 Department of Computer Science, University of Human Development, Sulaymaniyah, Iraq.
341 Department of Internal Medicine, Carol Davila University of Medicine and Pharmacy, Bucharest, Romania.
342 Department of Legal Medicine and Bioethics, Carol Davila University of Medicine and Pharmacy, Bucharest, Romania.
343 Clinical Legal Medicine Department, National Institute of Legal Medicine Mina Minovici, Bucharest, Romania.
344 College of Science and Engineering, Hamad Bin Khalifa University, Doha, Qatar.
345 Faculty of Medicine of Tunis, University Tunis El Manar, Tunis, Tunisia.
346 Department of Epidemiology and Health Statistics, Central South University, Changsha, China.
347 Department of Public Health and Community Medicine, Shaikh Khalifa Bin Zayed Al-Nahyan Medical College, Lahore, Pakistan.
348 Department of Occupational Safety and Health, China Medical University, Taichung, Taiwan.
349 Department of Public Health, University of Naples Federico II, Naples, Italy.
350 Department of Health Promotion and Education, University of Ibadan, Ibadan, Nigeria.
351 Department of Community Medicine, University of Ibadan, Ibadan, Nigeria.
352 Department of Community Medicine, University College Hospital, Ibadan, Ibadan, Nigeria.
353 Faculty of Medicine, University of Belgrade, Belgrade, Serbia.
354 Department of Epidemiology, University of Kragujevac, Kragujevac, Serbia.
355 Division of Community Health and Family Medicine, Bangalore Baptist Hospital, Bangalore, India.
356 College of Public Health, Taipei Medical University, Taipei, Taiwan.
357 Research Institute for Endocrine Sciences, Shahid Beheshti University of Medical Sciences, Tehran, Iran.
358 Graduate Institute of Biomedical Informatics, Taipei Medical University, Taipei, Taiwan.
359 Department of Social Medicine, Osaka University, Suita-city, Japan.
360 School of Public Health and Community Medicine, University of New South Wales, Sydney, NSW, Australia.
361 South African Medical Research Council, Cape Town, South Africa.

362 Department of Biochemistry and Microbiology, University of Fort Hare, Alice, South Africa.
363 Department of Environmental Health Engineering, Guilan University of Medical Sciences, Rasht, Iran.
364 N.A. Semashko Department of Public Health and Healthcare, I.M. Sechenov First Moscow State Medical University, Moscow, Russia.
365 Department of Global Health, Economics and Policy, University of Kragujevac, Kragujevac, Serbia.
366 Social Development and Health Promotion Research Center, Kermanshah University of Medical Sciences, Kermanshah, Iran.
367 Kermanshah University of Medical Sciences, Kermanshah, Iran.
368 Manipal College of Pharmaceutical Sciences, Manipal Academy of Higher Education, Manipal, India.
369 Health Informatic Lab, Boston University, Boston, MA, USA.
370 Autism Spectrum Disorders Research Center, Hamadan University of Medical Sciences, Hamadan, Iran.
371 Department of Community Medicine, Dr. Baba Saheb Ambedkar Medical College & Hospital, Delhi, India.
372 Department of Community Medicine, Banaras Hindu University, Varanasi, India.
373 Environmental Research Center, Duke Kunshan University, Kunshan, China.
374 Nicholas School of the Environment, Duke University, Durham, NC, USA.
375 Renal and Cardiovascular Division, The George Institute for Global Health, New Delhi, India.
376 School of Medicine, University of New South Wales, Sydney, NSW, Australia.
377 Department of Ophthalmology, Heidelberg University, Mannheim, Germany.
378 Beijing Institute of Ophthalmology, Beijing Tongren Hospital, Beijing, China.
379 Department of Family Medicine and Public Health, University of Opole, Opole, Poland.
380 Institute of Family Medicine and Public Health, University of Tartu, Tartu, Estonia.
381 Minimally Invasive Surgery Research Center, Iran University of Medical Sciences, Tehran, Iran.
382 School of Public Health, University College Cork, Cork, Ireland.
383 Department of Neurology, University of Washington, Seattle, WA, USA.
384 Institute for Prevention of Non-communicable Diseases, Qazvin University of Medical Sciences, Qazvin, Iran.
385 Health Services Management Department, Qazvin University of Medical Sciences, Qazvin, Iran.
386 Department of Forensic Medicine and Toxicology, All India Institute of Medical Sciences, Jodhpur, India.
387 Research Center for Environmental Determinants of Health, Kermanshah University of Medical Sciences, Kermanshah, Iran.
388 Institute for Epidemiology and Social Medicine, University of Münster, Münster, Germany.
389 Department of Adult Health Nursing, Bahir Dar University, Bahir Dar, Ethiopia.
390 International Research Center of Excellence, Institute of Human Virology Nigeria, Abuja, Nigeria.
391 Julius Centre for Health Sciences and Primary Care, Utrecht University, Utrecht, Netherlands.
392 Department of Public Health, Jordan University of Science and Technology, Irbid, Jordan.
393 School of Food and Agricultural Sciences, University of Management and Technology, Lahore, Pakistan.
394 Health Promotion Research Center, Zahedan University of Medical Sciences, Zahedan, Iran.

395 Epidemiology Department, Jazan University, Jazan, Saudi Arabia.
396 Department of Epidemiology and Biostatistics, Health Services Academy, Islamabad, Pakistan.
397 Faculty of Health and Wellbeing, Sheffield Hallam University, Sheffield, UK.
398 College of Arts and Sciences, Ohio University, Zanesville, OH, USA.
399 Center of Global Evidence Synthesis Initiative, Datta Meghe Institute of Medical Sciences, Wardha, India.
400 Shahid Beheshti University of Medical Sciences, Tehran, Iran.
401 The Iranian Academy of Medical Sciences, Tehran, Iran.
402 Department of Psychiatry, Kermanshah University of Medical Sciences, Kermanshah, Iran.
403 Department of Public Health, Imam Mohammad Ibn Saud Islamic University, Riyadh, Saudi Arabia.
404 Department of Health Policy and Management, Johns Hopkins University, Baltimore, MD, USA.
405 Department of Preventive Medicine, Korea University, Seoul, South Korea.
406 School of Traditional Chinese Medicine, Xiamen University Malaysia, Sepang, Malaysia.
407 Department of Nutrition, Simmons University, Boston, MA, USA.
408 Department of Nursing and Health Promotion, Oslo Metropolitan University, Oslo, Norway.
409 School of Health Sciences, Kristiania University College, Oslo, Norway.
410 Global Community Health and Behavioral Sciences, Tulane University, New Orleans, LA, USA.
411 Department of Epidemiology and Public Health, University College London, London, UK.
412 Department of Public Health, University of Helsinki, Helsinki, Finland.
413 Global Healthcare Consulting, New Delhi, India.
414 Department of Environmental Health Engineering, Arak University of Medical Sciences, Arak, Iran.
415 School of Population and Public Health, University of British Columbia, Vancouver, BC, Canada.
416 Arthritis Research Canada, Richmond, BC, Canada.
417 Global Health Department, Vancouver Virology Centre, Vancouver, BC, Canada.
418 Biomedical Research Networking Center for Mental Health Network (CiberSAM), San Juan de Dios Sanitary Park, Sant Boi de Llobregat, Spain.
419 Catalan Institution for Research and Advanced Studies (ICREA), Barcelona, Spain.
420 Department of Anthropology, Panjab University, Chandigarh, India.
421 Department of Demography, University of Montreal, Montreal, QC, Canada.
422 Department of Social and Preventive Medicine, University of Montreal, Montreal, QC, Canada.
423 Department of Community Medicine, Manipal Academy of Higher Education, Mangalore, India.
424 Department of Psychiatry, University of Nairobi, Nairobi, Kenya.
425 Division of Psychology and Language Sciences, University College London, London, UK.
426 Department of Medicine, McMaster University, Hamilton, ON, Canada.
427 Institute of Occupational and Environmental Medicine, University of Birmingham, Birmingham, UK.
428 Imperial College Business School, Imperial College London, London, UK.
429 Faculty of Public Health, University of Indonesia, Depok, Indonesia.
430 Nuffield Department of Population Health, University of Oxford, Oxford, UK.
431 National Institute for Health Research (NIHR) Oxford Biomedical Research Centre, Oxford, UK.
432 School of Dentistry, The University of Queensland, Brisbane, QLD, Australia.
433 Division of Research, Kaiser Permanente, Oakland, CA, USA.

434 Department of Community and Family Medicine, University of Baghdad, Baghdad, Iraq.
435 Unit of Genetics and Public Health, Institute of Medical Sciences, Las Tablas, Panama.
436 Ministry of Health, Herrera, Panama.
437 HelpMeSee, New York, NY, USA.
438 General Director, Mexican Institute of Ophthalmology, Queretaro, Mexico.
439 Department of Medical Sciences, Uppsala University, Uppsala, Sweden.
440 Department of Clinical Chemistry and Pharmacology, Uppsala University Hospital, Uppsala, Sweden.
441 Department of Otorhinolaryngology, Father Muller Medical College, Mangalore, India.
442 Institute of Clinical Physiology, National Research Council, Pisa, Italy.
443 Department of Clinical Sciences and Community Health, University of Milan, Milan, Italy.
444 College of Optometry, Nova Southeastern University, Fort Lauderdale, FL, USA.
445 Department of Information and Internet Technologies, I.M. Sechenov First Moscow State Medical University, Moscow, Russia.
446 Federal Research Institute for Health Organization and Informatics of the Ministry of Health (FRIHOI) , Moscow, Russia.
447 School of Nursing, Hong Kong Polytechnic University, Hong Kong, China.
448 School of Pharmacy, Monash University, Bandar Sunway, Malaysia.
449 School of Pharmacy, Taylor's University Lakeside Campus, Subang Jaya, Malaysia.
450 Asbestos Diseases Research Institute, University of Sydney, Sydney, NSW, Australia.
451 Neurology, Public Health and Disability Unit, Carlo Besta Neurological Institute IRCCS, Milan, Italy.
452 Division of Nephrology, Tufts Medical Center, Boston, MA, USA.
453 School of Medicine, Tufts University, Boston, MA, USA.
454 Department of Prevention, USL Tuscany Center, Firenze, Italy.
455 Department of Health Sciences, University of Florence, Florence, Italy.
456 School of Public Health and Preventive Medicine, Monash University, Melbourne, VIC, Australia.
457 School of Public Health, University of Haifa, Haifa, Israel.
458 Department of Systems, Populations, and Leadership, University of Michigan, Ann Arbor, MI, USA.
459 School of Population and Global Health, University of Melbourne, Melbourne, VIC, Australia.
460 Institute of Nutritional Sciences, Friedrich Schiller University Jena, Jena, Germany.
461 Competence Cluster for Nutrition and Cardiovascular Health (nutriCARD), Jena, Germany.
462 Department of Medicine, University of São Paulo, São Paulo, Brazil.
463 Department of General Surgery, Liverpool University Hospitals NHS Foundation Trust, Liverpool, UK.
464 Department of Surgery, University of Liverpool, Liverpool, UK.
465 Pathology Department, Imam Abdulrahman Bin Faisal University, Dammam, Saudi Arabia.
466 Institute for Physical Activity and Nutrition, Deakin University, Burwood, VIC, Australia.
467 Grants, Innovation and Product Development Unit, South African Medical Research Council, Cape Town, South Africa.
468 Radiology and Precision Health Program, Michigan State University, East Lansing, MI, USA.
469 Clinical Research Development Center, Kermanshah University of Medical Sciences, Kermanshah, Iran.

470 Environmental Health Research Center, Kurdistan University of Medical Sciences, Sanandaj, Iran.
471 Non-communicable Disease Research Center, Shiraz University of Medical Sciences, Shiraz, Iran.
472 Department of Maternal and Child Nursing and Public Health, Federal University of Minas Gerais, Belo Horizonte, Brazil.
473 Institute for Social Science Research, The University of Queensland, Indooroopilly, QLD, Australia.
474 School of Medicine, Iran University of Medical Sciences, Tehran, Iran.
475 School of Medicine, University of Manitoba, Winnipeg, MB, Canada.
476 Substance Abuse Prevention Research Center, Kermanshah University of Medical Sciences, Kermanshah, Iran.
477 Faculty of Public Health, Universitas Airlangga (Airlangga University), Surabaya, Indonesia.
478 Indonesian Public Health Association, Surabaya, Indonesia.
479 Campus Caucaia, Federal Institute of Education, Science and Technology of Ceará, Caucaia, Brazil.
480 Department of Midwifery, Hamadan University of Medical Sciences, Hamadan, Iran.
481 Department of Neurology, University Hospital Center de Sao Joao, Porto, Portugal.
482 Department of Clinical Neurosciences and Mental Health, University of Porto, Porto, Portugal.
483 Research Division, The George Institute for Global Health, New Delhi, India.
484 Department of Twin Research and Genetic Epidemiology, King's College London, London, UK.
485 Queensland Brain Institute, The University of Queensland, Brisbane, QLD, Australia.
486 National Centre for Register-based Research, Aarhus University, Aarhus, Denmark.
487 Department of Health Services Research and Policy, London School of Hygiene & Tropical Medicine, London, UK.
488 Neurology Department, Janakpuri Super Specialty Hospital Society, New Delhi, India.
489 Department of Neurology, Govind Ballabh Institute of Medical Education and Research, New Delhi, India.
490 Nutrition Health Research Center, Iran University of Medical Sciences, Hamadan, Iran.
491 Peru Country Office, United Nations Population Fund (UNFPA), Lima, Peru.
492 Forensic Medicine Division, Imam Abdulrahman Bin Faisal University, Dammam, Saudi Arabia.
493 Center for Translation Research and Implementation Science, National Institutes of Health, Bethesda, MD, USA.
494 Department of Medicine, University of Cape Town, Cape Town, South Africa.
495 Department of Cardiovascular Surgery, Asfendiyarov Kazakh National Medical University, Almaty, Kazakhstan.
496 Breast Surgery Unit, Helsinki University Hospital, Helsinki, Finland.
497 University of Helsinki, Helsinki, Finland.
498 Neurology Unit, Helsinki University Hospital, Helsinki, Finland.
499 School of Health Sciences, University of Melbourne, Parkville, VIC, Australia.
500 Clinical Microbiology and Parasitology Unit, Dr. Zora Profozic Polyclinic, Zagreb, Croatia.
501 University Centre Varazdin, University North, Varazdin, Croatia.
502 Department of Propedeutics of Internal Diseases & Arterial Hypertension, Pomeranian Medical University, Szczecin, Poland.
503 Woman-Mother-Child Department, Lausanne University Hospital, Lausanne, Switzerland.
504 Pacific Institute for Research & Evaluation, Calverton, MD, USA.

505 Department of Health Research Methods, Evidence and Impact, McMaster University, Hamilton, ON, Canada.
506 Internal Medicine Programme, Kyrgyz State Medical Academy, Bishkek, Kyrgyzstan.
507 Department of Atherosclerosis and Coronary Heart Disease, National Center of Cardiology and Internal Disease, Bishkek, Kyrgyzstan.
508 Research Center for Biochemistry and Nutrition in Metabolic Diseases, Kashan University of Medical Sciences, Kashan, Iran.
509 Department of Sports Medicine and Rehabilitation, Kermanshah University of Medical Sciences, Kermanshah, Iran.
510 School of Psychiatry, University of New South Wales, Sydney, NSW, Australia.
511 Institute of Addiction Research (ISFF), Frankfurt University of Applied Sciences, Frankfurt, Germany.
512 Biotechnology Research Center, Tabriz University of Medical Sciences, Tabriz, Iran.
513 Molecular Medicine Research Center, Tabriz University of Medical Sciences, Tabriz, Iran.
514 Health Equity Research Center, Tehran University of Medical Sciences, Tehran, Iran.
515 Internal Medicine Department, King Saud University, Riyadh, Saudi Arabia.
516 Department of Forestry, Salahaddin University-Erbil, Erbil, Iraq.
517 Department of Medicine-Huddinge, Karolinska Institutet, Stockholm, Sweden.
518 Research Institute for Health Development, Kurdistan University of Medical Sciences, Sanandaj City, Iran.
519 Department of Epidemiology and Biostatistics, Shahrekord University of Medical Sciences, Shahrekord, Iran.
520 Health Systems and Policy Research Unit, Ahmadu Bello University, Zaria, Nigeria.
521 Department of Biomedical and Dental Sciences and Morphofunctional Imaging, Messina University, Messina, Italy.
522 Social Determinants of Health Research Center, Kurdistan University of Medical Sciences, Sanandaj, Iran.
523 Department of Mathematical Sciences, University of Bath, Bath, UK.
524 International Laboratory for Air Quality and Health, Queensland University of Technology, Brisbane, QLD, Australia.
525 Graduate School of Medicine, Kyoto University, Kyoto, Japan.
526 Asia-Pacific Regional Office, United Nations Population Fund (UNFPA), Bangkok, Thailand.
527 Department of Health Policy, Management, and Economics, Tehran University of Medical Sciences, Tehran, Iran.
528 Department of Food Science, University of Campinas (Unicamp), Campinas, Brazil.
529 Federal Institute for Population Research, Wiesbaden, Germany.
530 Center for Population and Health, Wiesbaden, Germany.
531 Department of Endocrinology & Metabolism, Institute of Post-Graduate Medical Education and Research and Seth Sukhlal Karnani Memorial Hospital, Kolkata, India.
532 School of Economics, University of Nairobi, Nairobi, Kenya.
533 Research and Analytics Department, Initiative for Financing Health and Human Development, Chennai, India.
534 Department of Research and Analytics, Bioinsilico Technologies, Chennai, India.

535 Department of Cardiology, Hamedan University of Medical Sciences, Hamadan, Iran.
536 Laboratory of Public Health Indicators Analysis and Health Digitalization, Moscow Institute of Physics and Technology, Dolgoprudny, Russia.
537 Experimental Surgery and Oncology Laboratory, Kursk State Medical University, Kursk, Russia.
538 Department of Epidemiology and Biostatistics, Kermanshah University of Medical Sciences, Kermanshah, Iran.
539 Department for the Control of Disease, Epidemics, and Pandemics, Ministry of Public Health, Yaoundé, Cameroon.
540 Department of Public Health, University of Yaoundé I, Yaoundé, Cameroon.
541 Department of Disease Control and Environmental Health, Makerere University, Kampala, Uganda.
542 Department of General Surgery, Carol Davila University of Medicine and Pharmacy, Bucharest, Romania.
543 Department of General Surgery, Emergency Hospital of Bucharest, Bucharest, Romania.
544 Department of Anatomy and Embryology, Carol Davila University of Medicine and Pharmacy, Bucharest, Romania.
545 Cardio-Aid, Bucharest, Romania.
546 Department of Health Sciences, University of Tampere, Tampere, Finland.
547 Department of Biological Sciences, University of Embu, Embu, Kenya.
548 Institute for Global Health Innovations, Duy Tan University, Hanoi, Vietnam.
549 School of Public Health and Family Medicine, University of Cape Town, Cape Town, South Africa.
550 Department of Community and Family Medicine, Iran University of Medical Sciences, Tehran, Iran.
551 Department of Health Policy and Management, Keio University, Tokyo, Japan.
552 Department of Global Health Policy, University of Tokyo, Tokyo, Japan.
553 Department of Clinical Sciences, Lund University, Lund, Sweden.
554 Centre for Heart Rhythm Disorders, University of Adelaide, Adelaide, SA, Australia.
555 Department of Neurobiology, Care Sciences and Society, Karolinska Institutet, Huddinge, Sweden.
556 Unit of Microbiology and Public Health, Institute of Medical Sciences, Las Tablas, Panama.
557 Department of Public Health, Ministry of Health, Herrera, Panama.
558 Translational Health Research Institute, Western Sydney University, Sydney, NSW, Australia.
559 Department of Preventive Medicine, Kyung Hee University, Dongdaemun-gu, South Korea.
560 Gorgan Congenital Malformations Research Center, Golestan University of Medical Sciences, Gorgan, Iran.
561 Department of Psychiatry and Behavioural Neurosciences, McMaster University, Hamilton, ON, Canada.
562 Department of Psychiatry, University of Lagos, Lagos, Nigeria.
563 Centre for Healthy Start Initiative, Lagos, Nigeria.
564 Department of Pharmacology and Therapeutics, University of Nigeria Nsukka, Enugu, Nigeria.
565 Department of Biomedical Sciences, University of Novi Sad, Novi Sad, Serbia.
566 Department of Project Management, National Research University Higher School of Economics, Moscow, Russia.
567 Department of Medicine, University of Ibadan, Ibadan, Nigeria.

568 Department of Medicine, University College Hospital, Ibadan, Ibadan, Nigeria.
569 Department of Respiratory Medicine, Jagadguru Sri Shivarathreeswara Academy of Health Education and Research, Mysore, India.
570 Department of Forensic Medicine, Manipal Academy of Higher Education, Mangalore, India.
571 Department of Ophthalmology, Heidelberg University, Heidelberg, Germany.
572 Department of Research, Public Health Foundation of India, Gurugram, India.
573 Department of Clinical Epidemiology, University of the Philippines Manila, Manila, Philippines.
574 Division of General Internal Medicine, University of Pittsburgh Medical Center, Pittsburgh, PA, USA.
575 Department of Physiology, Iran University of Medical Sciences, Tehran, Iran.
576 Physiology Research Center, Iran University of Medical Sciences, Tehran, Iran.
577 School of Public Health, Medical, and Veterinary Sciences, James Cook University, Townsville, NSW, Australia.
578 Center for Research and Innovation, Ateneo De Manila University, Pasig City, Philippines.
579 School of Global Public Health, New York University, New York, NY, USA.
580 Department of Orthopedics, Yenepoya Medical College, Mangalore, India.
581 School of Optometry and Vision Science, University of New South Wales, Sydney, NSW, Australia.
582 Shanghai Mental Health Center, Shanghai Jiao Tong University, Shanghai, China.
583 Department of Psychiatry, Columbia University, New York City, NY, USA.
584 Department of Cardiology, University of Bern, Bern, Switzerland.
585 Department of Chemistry, University of Porto, Porto, Portugal.
586 Department of Public Health, Erasmus University Medical Center, Rotterdam, Netherlands.
587 University Medical Center Groningen, University of Groningen, Groningen, Netherlands.
588 School of Economics and Business, University of Groningen, Groningen, Netherlands.
589 Department of Nutrition and Food Sciences, Maragheh University of Medical Sciences, Maragheh, Iran.
590 Dietary Supplements and Probiotic Research Center, Alborz University of Medical Sciences, Karaj, Iran.
591 Clinical Research Center, Fundación Valle del Lili, Cali, Colombia.
592 Center for Studies in Social Protection and Health Economics, ICESI University, Cali, Colombia.
593 Department of Neurology, Smt. B.K.S. Medical Institute and Research Center, Vadodara, India.
594 Health Sciences Department, Muhammadiyah University of Surakarta, Sukoharjo, Indonesia.
595 Department of Community Medicine, Datta Meghe Institute of Medical Sciences, Wardha, India.
596 Department of Chemistry, Sharif University of Technology, Tehran, Iran.
597 College of Medicine, University of Central Florida, Orlando, FL, USA.
598 Department of Medicine, University of Alberta, Edmonton, AB, Canada.
599 Thalassemia and Hemoglobinopathy Research Center, Ahvaz Jundishapur University of Medical Sciences, Ahvaz, Iran.
600 Metabolomics and Genomics Research Center, Tehran University of Medical Sciences, Tehran, Iran.
601 Sina Trauma and Surgery Research Center, Tehran University of Medical Sciences, Tehran, Iran.
602 Department of Community Medicine, Maharishi Markandeshwar Institute of Medical Sciences & Research, Ambala, India.

603 School of Nursing and Healthcare Professions, Federation University Australia, Berwick, VIC, Australia.
604 Department of Pharmacology, Shahid Beheshti University of Medical Sciences, Tehran, Iran.
605 Research Department, Policy Research Institute, Kathmandu, Nepal.
606 Health and Public Policy Department, Global Center for Research and Development, Kathmandu, Nepal.
607 Department of Medicine, University of Otago, Wellington, New Zealand.
608 Department of Neurology, Capital & Coast District Health Board, Wellington, New Zealand.
609 Department of Oral Pathology, Srinivas Institute of Dental Sciences, Mangalore, India.
610 Tehran Institute of Psychiatry, Iran University of Medical Sciences, Tehran, Iran.
611 Department of Forensic Medicine and Toxicology, Manipal Academy of Higher Education, Mangalore, India.
612 Academic Public Health England, Public Health England, London, UK.
613 WHO Collaborating Centre for Public Health Education and Training, Imperial College London, London, UK.
614 University College London Hospitals, London, UK.
615 School of Health, Medical and Applied Sciences, CQ University, Sydney, NSW, Australia.
616 Department of Computer Science, Boston University, Boston, MA, USA.
617 School of Social Sciences and Psychology, Western Sydney University, Penrith, NSW, Australia.
618 Translational Health Research Institute, Western Sydney University, Penrith, NSW, Australia.
619 Department of Health Information Management, Manipal Academy of Higher Education, Manipal, India.
620 Manipal Academy of Higher Education, Manipal, India.
621 Brien Holden Vision Institute, Sydney, Australia.
622 Network of Immunity in Infection, Malignancy and Autoimmunity (NIIMA), Universal Scientific Education and Research Network (USERN), Tehran, Iran.
623 Cardiovascular Diseases Research Center, Birjand University of Medical Sciences, Birjand, Iran.
624 Department of Chemical Sciences, University of Porto, Porto, Portugal.
625 Epidemiology Research Unit Institute of Public Health (EPIUnit-ISPUP), University of Porto, Porto, Portugal.
626 Department of Surgery, University of Minnesota, Minneapolis, MN, USA.
627 Department of Surgery, University Teaching Hospital of Kigali, Kigali, Rwanda.
628 Department of Clinical Research, Federal University of Uberlândia, Uberlândia, Brazil.
629 Department of Neuroscience, University of Perugia, Perugia, Italy.
630 Department of Neurology, Rimini ‘Infermi’ Hospital - AUSL Romagna, Rimini, Italy.
631 Institute of Epidemiology and Medical Biometry, Ulm University, Ulm, Germany.
632 Agrosavia, Palmira, Colombia.
633 Malaria Atlas Project, University of Oxford, Oxford, UK.
634 Department of Health Statistics, National Institute for Medical Research, Dar es Salaam, Tanzania.
635 Department of Cardiovascular Medicine, Mayo Clinic, Rochester, MN, USA.
636 Department of Epidemiology, Shahid Beheshti University of Medical Sciences, Tehran, Iran.
637 School of Psychiatry, University of New South Wales, Kensington, NSW, Australia.
638 Neuropsychiatric Institute, Prince of Wales Hospital, Randwick, NSW, Australia.

639 Cardiac Rehabilitation Research Center, Isfahan University of Medical Sciences, Isfahan, Iran.
640 Department of Psychiatry, All India Institute of Medical Sciences, New Delhi, India.
641 Department of Phytochemistry, Soran University, Soran, Iraq.
642 Department of Nutrition, Cihan University-Erbil, Kurdistan Region, Iraq.
643 Department of Microbiology, Central University of Punjab, Bathinda, India.
644 Department of Public Health and Community Medicine , Cairo University, Giza, Egypt.
645 Department of Global Health and Development, London School of Hygiene & Tropical Medicine, London, UK.
646 Department of Entomology, Ain Shams University, Cairo, Egypt.
647 Department of Surgery, Marshall University, Huntington, WV, USA.
648 Department of Nutrition and Preventive Medicine, Case Western Reserve University, Cleveland, OH, USA.
649 Rheumatology Department, University Hospitals Bristol NHS Foundation Trust, Bristol, UK.
650 School of Public Health and Health Management, University of Belgrade, Belgrade, Serbia.
651 Department of Community Medicine, PSG Institute of Medical Sciences and Research, Coimbatore, India.
652 PSG-FAIMER South Asia Regional Institute, Coimbatore, India.
653 Colorectal Research Center, Iran University of Medical Sciences, Tehran, Iran.
654 Department of Geriatrics and Long Term Care, Hamad Medical Corporation, Doha, Qatar.
655 Faculty of Health & Social Sciences, Bournemouth University, Bournemouth, UK.
656 Population Health Research Institute, McMaster University, Hamilton, ON, Canada.
657 The George Institute for Global Health, Sydney, NSW, Australia.
658 Department of Psychology, University of Alabama at Birmingham, Birmingham, AL, USA.
659 Oral Diagnosis, Digital Health and Health Services Research, Charité University Medical Center Berlin, Berlin, Germany.
660 Emergency Department, Manian Medical Centre, Erode, India.
661 Center for Biomedical Information Technology, Shenzhen Institutes of Advanced Technology, Shenzhen, China.
662 Department of Radiology and Interventional Neuroradiology, Isfahan University of Medical Sciences, Isfahan, Iran.
663 Health Policy Research Center, Shiraz University of Medical Sciences, Shiraz, Iran.
664 Public Health Division, An-Najah National University, Nablus, Palestine.
665 Independent Consultant, Karachi, Pakistan.
666 National Centre for Disease Control (NCDC), Tbilisi, Georgia.
667 School of Medicine, Alborz University of Medical Sciences, Karaj, Iran.
668 Faculty of Caring Science, Work Life, and Social Welfare, University of Borås, Borås, Sweden.
669 Institute for Population Health, King's College London, London, UK.
670 National Institute of Infectious Diseases, Tokyo, Japan.
671 College of Medicine, Yonsei University, Seoul, South Korea.
672 Finnish Institute of Occupational Health, Helsinki, Finland.
673 Cancer Research Institute, Tehran University of Medical Sciences, Tehran, Iran.
674 Cancer Biology Research Center, Tehran University of Medical Sciences, Tehran, Iran.

675 Department of Psychology, Reykjavik University, Reykjavik, Iceland.
676 Department of Health and Behavior Studies, Columbia University, New York, NY, USA.
677 Department of Law, Economics, Management and Quantitative Methods, University of Sannio, Benevento, Italy.
678 WSB University in Gdańsk, Gdansk, Poland.
679 School of Medicine, University of Alabama at Birmingham, Birmingham, AL, USA.
680 Medicine Service, US Department of Veterans Affairs (VA), Birmingham, AL, USA.
681 Department of Humanities and Social Sciences, Indian Institute of Technology, Roorkee, Roorkee, India.
682 Department of Epidemiology, School of Preventive Oncology, Patna, India.
683 Department of Epidemiology, Healis Sekhsaria Institute for Public Health, Mumbai, India.
684 Department of Sports Science and Clinical Biomechanics, University of Southern Denmark, Odense, Denmark.
685 Department of Physiotherapy and Occupational Therapy, Næstved-Slagelse-Ringsted Hospitals, Slagelse, Denmark.
686 Department No.16, Moscow Research and Practical Centre on Addictions, Moscow, Russia.
687 Institute of Bone and Joint Research (IBJR), University of Sydney, St Leonards, NSW, Australia.
688 Australian New Zealand Clinical Trials Registry (ANZCTR), NHMRC Clinical Trials Centre, University of Sydney, Camperdown, NSW, Australia.
689 Department of Vascular and Endovascular Surgery, Kermanshah University of Medical Sciences, Kermanshah, Iran.
690 Nursing Care Research Center, Semnan University of Medical Sciences, Semnan, Iran.
691 Hospital Universitario de La Princesa (Princess University Hospital), Autonomous University of Madrid, Madrid, Spain.
692 Centro de Investigación Biomédica en Red Enfermedades Respiratorias (CIBERES-Center for Biomedical Research in Respiratory Diseases Network), Madrid, Spain.
693 Hull York Medical School, University of Hull, Hull City, UK.
694 Division of Community Medicine, International Medical University, Kuala Lumpur, Malaysia.
695 Risk and Resilience in Mental Disorders Unit, South African Medical Research Council, Cape Town, South Africa.
696 Occupational and Environmental Medicine Department, University of Gothenburg, Gothenburg, Sweden.
697 Department of Psychology, Deakin University, Burwood, VIC, Australia.
698 Department of Epidemiology & Biostatistics, The University of Western Ontario, London, ON, Canada.
699 Department of Population Health, Luxembourg Institute of Health, Strassen, Luxembourg.
700 Department of Psychiatry, University of British Columbia, Vancouver, BC, Canada.
701 Nursing Department, Muhammadiyah University of Surakarta, Surakarta, Indonesia.
702 Department of Community Medicine, Ahmadu Bello University, Zaria, Nigeria.
703 Department of Agriculture and Food Systems, University of Melbourne, Melbourne, VIC, Australia.
704 Department of Statistics, Manonmaniam Sundaranar University, Abishekapatti, Tirunelveli, India.
705 National Institute of Epidemiology, Indian Council of Medical Research, Chennai, India.

706 Norwegian Institute of Public Health, Bergen, Norway.
707 Department of Medicine, University of Valencia, Valencia, Spain.
708 Carlos III Health Institute, Biomedical Research Networking Center for Mental Health Network (CiberSAM), Madrid, Spain.
709 Cancer Control Center, Osaka International Cancer Institute, Osaka, Japan.
710 Department of Pharmacy, Arbaminch College of Health Sciences, Arbaminch, Ethiopia.
711 Research Center for Molecular Medicine, Hamadan University of Medical Sciences, Hamadan, Iran.
712 Department of Population Science and Human Resource Development, University of Rajshahi, Rajshahi, Bangladesh.
713 Department of Clinical Pharmacy, University of Gondar, Gondar, Ethiopia.
714 Pediatric Intensive Care Unit, King Saud University, Riyadh, Saudi Arabia.
715 Department of Epidemiology and Biostatistics, University of Gondar, Gondar, Ethiopia.
716 Department of Public Health and Community Medicine, Central University of Kerala, Kasaragod, India.
717 Department of Medicine, Monash University, Melbourne, VIC, Australia.
718 K.A. Timiryazev Institute of Plant Physiology, Russian Academy of Sciences, Moscow, Russia.
719 Laboratory of Public Health Indicators Analysis and Health Digitalization, Moscow Institute of Physics and Technology, Moscow, Russia.
720 HIV/STI Surveillance Research Center, and WHO Collaborating Center for HIV Surveillance, Kerman University of Medical Sciences, Kerman, Iran.
721 Department of Medicine, University of Calgary, Calgary, AB, Canada.
722 Nutritional Epidemiology Research Team (EREN), National Institute for Health and Medical Research (INSERM), Paris, France.
723 Health, Medicine and Human Biology, Sorbonne Paris Nord University, Bobigny, France.
724 Department of Pathology and Legal Medicine, University of São Paulo, Ribeirão Preto, Brazil.
725 Modestum LTD, London, UK.
726 Department of Health Economics, Hanoi Medical University, Hanoi, Vietnam.
727 Department of Psychiatry, Massachusetts General Hospital, Boston, MA, USA.
728 Mbarara University of Science and Technology, Mbarara, Uganda.
729 Department of Medicine, University of Crete, Heraklion, Greece.
730 Institute for Physical Activity and Nutrition, Deakin University, Melbourne, QLD, Australia.
731 School of Health and Rehabilitation Sciences, The University of Queensland, Brisbane, Australia.
732 Department of Community Medicine, Alex Ekwueme Federal University Teaching Hospital Abakaliki, Abakaliki, Nigeria.
733 Kasturba Medical College, Manipal Academy of Higher Education, Mangalore, India.
734 Argentine Society of Medicine, Buenos Aires, Argentina.
735 Velez Sarsfield Hospital, Buenos Aires, Argentina.
736 Department of Physics and Atmospheric Science, Dalhousie University, Halifax, NS, Canada.
737 Department of Energy, Environmental and Chemical Engineering, Washington University in St. Louis, St. Louis, MO, USA.
738 Department of Nephrology, Christian Medical College and Hospital (CMC), Vellore, India.
739 UKK Institute, Tampere, Finland.

740 Psychosocial Injuries Research Center, Ilam University of Medical Sciences, Ilam, Iran.
741 Raffles Neuroscience Centre, Raffles Hospital, Singapore, Singapore.
742 Yong Loo Lin School of Medicine, National University of Singapore, Singapore, Singapore.
743 Department of Medical and Surgical Sciences, University of Bologna, Bologna, Italy.
744 Occupational Health Unit, Sant'Orsola Malpighi Hospital, Bologna, Italy.
745 Department of Health Care Administration and Economics, National Research University Higher School of Economics, Moscow, Russia.
746 Foundation University Medical College, Foundation University Islamabad, Islamabad, Pakistan.
747 School of Population Health and Environmental Sciences, King's College London, London, UK.
748 School of Health Sciences, Wuhan University, Wuhan, China.
749 Department of Epidemiology and Biostatistics, George Washington University, Washington, DC, USA.
750 Cardiology Department, Royal Children's Hospital, Melbourne, VIC, Australia.
751 Critical Care and Neurosciences, Murdoch Childrens Research Institute, Melbourne, VIC, Australia.
752 Department of Demography, University of California Berkeley, Berkley, CA, USA.
753 Competence Center of Mortality-Follow-Up of the German National Cohort, Federal Institute for Population Research, Wiesbaden, Germany.
754 Department of Physical Therapy, Naresuan University, Phitsanulok, Thailand.
755 National Institute for Health Research (NIHR) Biomedical Research Centre, Guy's and St.Thomas' Hospital and Kings College London, London, UK.
756 Department of Orthopaedics, Wenzhou Medical University, Wenzhou, China.
757 Clinical Cancer Research Center, Milad General Hospital, Tehran, Iran.
758 Research and Development Center for Health Services, University of Tsukuba, Tsukuba, Japan.
759 Graduate School of Medicine, Osaka University, Suita, Japan.
760 Department of Family Medicine and Community Health, Duke University, Durham, NC, USA.
761 School of International Development and Global Studies, University of Ottawa, Ottawa, ON, Canada.
762 The George Institute for Global Health, University of Oxford, Oxford, UK.
763 Health Services Management Research Center, Kerman University of Medical Sciences, Kerman, Iran.
764 Department of Health Management, Policy, and Economics, Kerman University of Medical Sciences, Kerman, Iran.
765 Department of Nursing, Arba Minch University, Arba Minch, Ethiopia.
766 Department of Anatomy, Mekelle University, Mekelle, Ethiopia.
767 Centre for Suicide Research and Prevention, University of Hong Kong, Hong Kong, China.
768 Department of Social Work and Social Administration, University of Hong Kong, Hong Kong, China.
769 Department of Neuropsychopharmacology, National Center of Neurology and Psychiatry, Kodaira, Japan.
770 Department of Public Health, Juntendo University, Tokyo, Japan.
771 Department of Health Policy and Management, Jackson State University, Jackson, MS, USA.
772 School of Medicine, Tsinghua University, Beijing, China.

773 Department of Environmental Health, Mazandaran University of Medical Sciences, Sari, Iran.
774 Injury Prevention and Safety Promotion Research Center, Shahid Beheshti University of Medical Sciences, Tehran, Iran.
775 Department of Epidemiology and Biostatistics, Wuhan University, Wuhan, China.
776 Global Health Institute, Wuhan University, Wuhan, China.
777 School of Public Health and Management, Hubei University of Medicine, Shiyan, China.
778 Department of Healthcare Management and Economics, Urmia University of Medical Science, Urmia, Iran.
779 Department of Pharmaceutics, Dow University of Health Sciences, Karachi, Pakistan.
780 The School of Clinical Sciences at Monash Health, Monash University, Melbourne, VIC, Australia.
781 Psychiatry and Psychology Research Center, Tehran University of Medical Sciences, Tehran, Iran.
782 Laboratory of Genetics and Genomics, Moscow Research and Practical Centre on Addictions, Moscow, Russia.
783 Addictology Department, Russian Medical Academy of Continuous Professional Education, Moscow, Russia.
784 School of Medicine, Wuhan University, Wuhan, China.
785 Department of General Practice, University of Melbourne, Melbourne, Australia.
786 Victorian Comprehensive Cancer Centre, Melbourne, Australia.
787 School of Public Health, Wuhan University of Science and Technology, Wuhan, China.
788 Hubei Province Key Laboratory of Occupational Hazard Identification and Control, Wuhan University of Science and Technology, Wuhan, China.
789 School of Biology and Pharmaceutical Engineering, Wuhan Polytechnic University, Wuhan, China.
790 Department of Epidemiology, Human Genetics, and Environmental Sciences, University of Texas Health Science Center at Houston, Houston, TX, USA.
791 Department of Anesthesiology & Pain Medicine, University of Washington, Seattle, WA, USA.

Global Burden of Disease overview

GATHER statement

This study is in compliance with the Guidelines for Accurate and Transparent Health Estimates Reporting (GATHER) statement. The GBD 2019 capstone papers[1–3] and their respectivesupplementary documents contain the general methods, data sources, model selection information, performance and limitation information for the GBD 2019 analyses includingdetailed GATHER documentation. Supplementary table 1 contains GATHER compliance information for this publication.

GBD cause list and location hierarchy

The GBD cause list can be found in the supplementary table 2, consisting of a hierarchy that ismutually exclusive and collectively exhaustive. The GBD location hierarchy, including levels, for GBD superregions, regions, countries and sub-country analysis is presented in supplementary table 3. Details on the overall GBD 1990–2019 cause list have been documented and can be found elsewhere[1,3]. This paper uses the same cause list in the populationaged 70 years and older.

Socio-demographic Index (SDI)

The Socio-demographic Index (SDI) is a composite indicator of development status initially constructed for

GBD 2015 whose components are strongly correlated with health outcomes. SDI in GBD 2019 incorporated changes that replaced the total fertility rate (TRF) compoment with the total fertility rate (TFU25) in females under the age of 25 years from previous GBD cycles. Specific SDI methodology information is described elsewhere.[4]

Data sources

A complete list of sources used in the GBD 2019 analyses is available from the GBD 2019 DataInput Sources Tool (http://ghdx.healthdata.org/gbd-2019/data-input-sources).

Mortality and death cause-specific estimation process for those over 70 years old

Mortality and death cause estimates for the population aged 70 years and older had followed the standard procedure methodology of GBD 2019 and were generated using Cause of Death Ensemble modelling (CODEm). The CODEm approach has been described elsewhere[1,3].

The cause of death (CoD) database contains seven types of data sources: vital registration (VR), verbal autopsy (VA), cancer registry (CR), police records, sibling history, surveillance, and survey/census. When all the types of data are collected in various formats, then the input data are standardised to run through central CoD machinery to then to be uploaded to the database. A specific tool has been developed through GBD called age-sex splitting, that takes aggregated age groups, and likewise the “both sexes combined” grouping, and divides them into what their constituent age groups would likely have been using respective cause-specific and country- specific age distributions. This allows the GBD study to include age categories from early neonatal (0-6 days) to the age group of 95 years and older. This calculation formula is described in detailed elsewhere.[1,3]

For certain causes of death, mortality rates reported in vital registration systems are impossible to reconcile with observed trends in disease prevalence and excess mortality. These include dementia, Parkinson’s disease, and atrial fibrillation and flutter. It is well known that dementiasand Parkinson’s disease are highly prevalent in the older population, starting mildy even from the age of 70.[5,1] GBD 2017 as well as 2019 made an effort to calculate the known bias regardingthese causes of death data by detecting the proportion of deaths that had to be assigned to these causes, and after that, to assess the GBD causes and garbage codes to which these kind of deathswere inaccurately assigned. As described in the past GBD methodologies (GBD 2017 and 2015),[1,6] the aforementioned causes of death were processed in CODCorrect in a manner that inappropriately led to changes in mortality estimates for causes that were not related to dementia,Parkinson’s, and atrial fibrillation and flutter. In GBD 2016, a huge literature review[7] was applied to assess the causes of death mostly related to Parkinson’s and Alzheimer’s diseases, helping in further CodCorrect adjustments, while in GBD 2017 and 2019 a more refined process,even further limiting bias, was used.[1,5] The current study, by applying this miscoding correction for the aforementioned diseases, is calibrating the mortality rates for those 70 years and older with the minimum possible bias.

Non-fatal modelling methods for those 70 years and older

Non-fatal estimates for the population aged 70 years and older were modelled using the DisMod-MR 2.1 tool. Morbidity modelling methods used in the same manner as described in detail in theGBD 2019 methodology have been documented elsewhere.[2,8]

A group of behavioural, environmental and occupational, and metabolic risks that contribute todisease burden were evaluated in GBD 2019. The same group of risk factors was used in the current analysis to identify those that are closely related to the ageing population. The methodology regarding risk factor estimation and evaluation is described in detail elsewhere.[2,4]

Healthcare Access and Quality (HAQ) Index

The HAQ Index in GBD 2016 used 32 causes for which death should not occur in the presence of effective care to approximate personal health-care access and quality by location and over time. A risk-standardised cause-specific death methodology due to non-cancers by location-year,replaced the local combined exposure of environmental and behavioural risks with the global level of exposure applied, to isolate potential effects of personal health-care access and quality from underlying risk factor patterns. Each cause transformed to a scale of 0–100, with 0 as the first percentile (worst) observed between 1990 and 2016, and 100 as the 99th percentile (best); these thresholds were set at the country level, and then applied to subnational locations. The rest of the HAQ Index methodology is described in detail elsewhere.[9]

References

1. GBD 2019 Demographics Collaborators. Global age-sex-specific fertility, mortality, healthy life expectancy (HALE), and population estimates in 204 countries and territories, 1950-2019: a comprehensive demographic analysis for the Global Burden of Disease Study 2019. Lancet 2020; 396: 1160–203.
2. GBD 2019 Risk Factors Collaborators. Global burden of 87 risk factors in 204 countries and territories, 1990-2019: a systematic analysis for the Global Burden of Disease Study 2019. Lancet 2020; 396: 1223–49.
3. Vos T, Lim SS, Abbafati C, et al. Global burden of 369 diseases and injuries in 204 countries and territories, 1990–2019: a systematic analysis for the Global Burden of Disease Study 2019. The Lancet 2020; 396: 1204–22.
4. Stanaway JD, Afshin A, Gakidou E, et al. Global, regional, and national comparative risk assessment of 84 behavioural, environmental and occupational, and metabolic risks or clusters of risks for 195 countries and territories, 1990–2017: a systematic analysis for the Global Burden of Disease Study 2017. The Lancet 2018; 392: 1923–94.
5. Roth GA, Abate D, Abate KH, et al. Global, regional, and national age-sex-specific mortality for 282 causes of death in 195 countries and territories, 1980–2017: a systematic analysis for the Global Burden of Disease Study 2017. The Lancet 2018; 392: 1736–88.
6. GBD 2015 Mortality and Causes of Death Collaborators. Global, regional, and national life expectancy, all-cause mortality, and cause-specific mortality for 249 causes of death, 1980- 2015: a systematic analysis for the Global Burden of Disease Study 2015. Lancet 2016; 388: 1459–544.
7. GBD 2016 Causes of Death Collaborators. Global, regional, and national age-sex specific mortality for 264 causes of death, 1980-2016: a systematic analysis for the Global Burden of Disease Study 2016. Lancet 2017; 390: 1151–210.
8. GBD 2017 DALYs and HALE Collaborators. Global, regional, and national disability-adjusted life-years (DALYs) for 359 diseases and injuries and healthy life expectancy (HALE) for 195 countries and territories, 1990-2017: a systematic analysis for the Global Burden of Disease Study 2017. Lancet 2018; 392: 1859–922.
9. GBD 2016 Healthcare Access and Quality Collaborators. Measuring performance on the Healthcare Access and Quality Index for 195 countries and territories and selected subnational locations: a systematic analysis from the Global Burden of Disease Study 2016. Lancet 2018; 391: 2236–71.

Figure S1. Global population change by age group for both sexes combined (top panel), population percentagechange during the same period (upper middle panel), rate of change in years lived with disability (YLDs; lower middle panel), and rate of change in All-cause mortality (bottom panel) between 1990 and 2019 in Low Socio-demographic Index (SDI) regions.

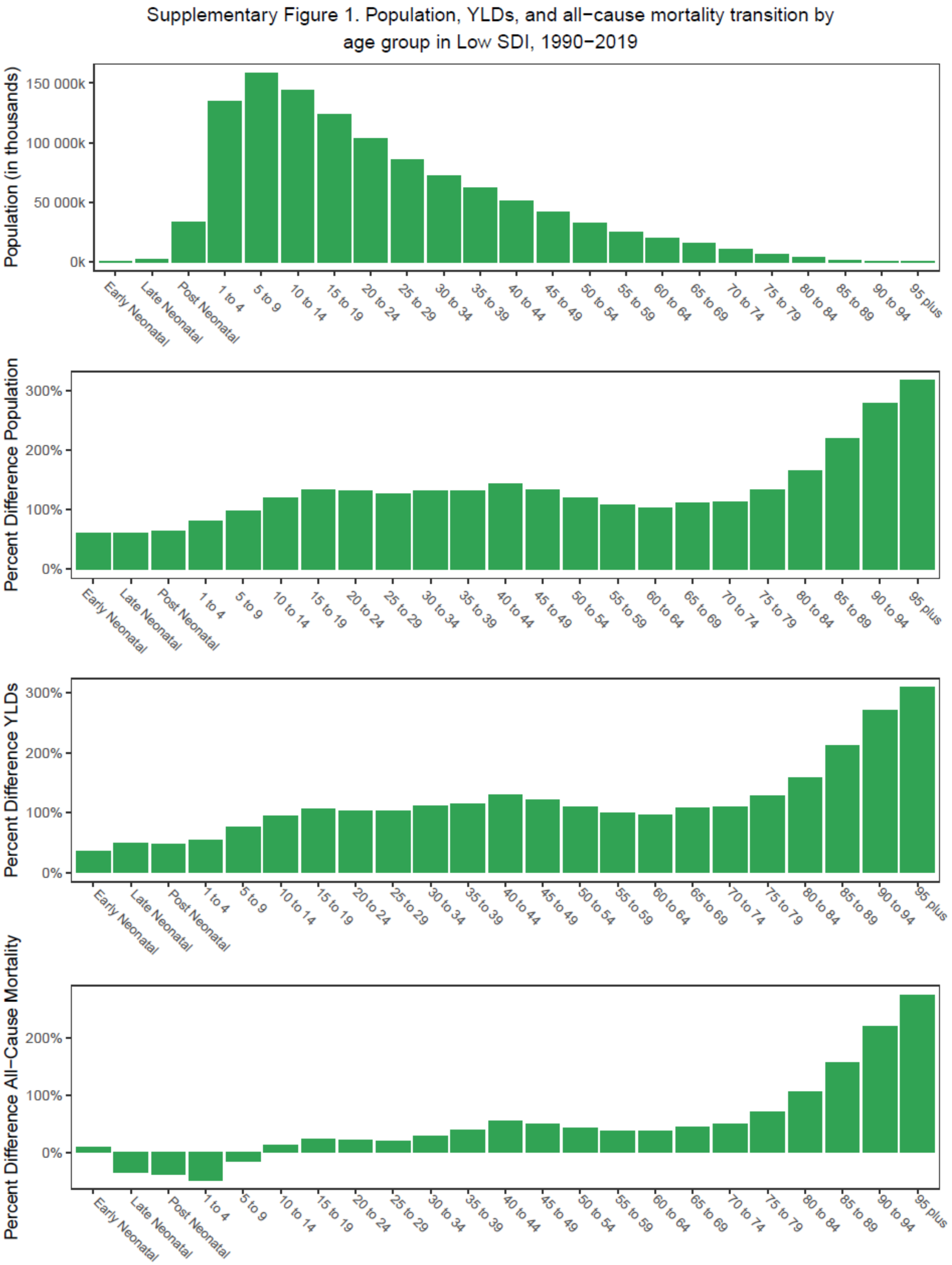

Figure S2. Global population change by age group for both sexes combined (top panel), population percentage change during the same period (upper middle panel), rate of change in years lived with disability (YLDs; lower middle panel), and rate of change in All-cause mortality (bottom panel) between 1990 and 2019 in Low-middle Socio-demographic Index (SDI) regions.

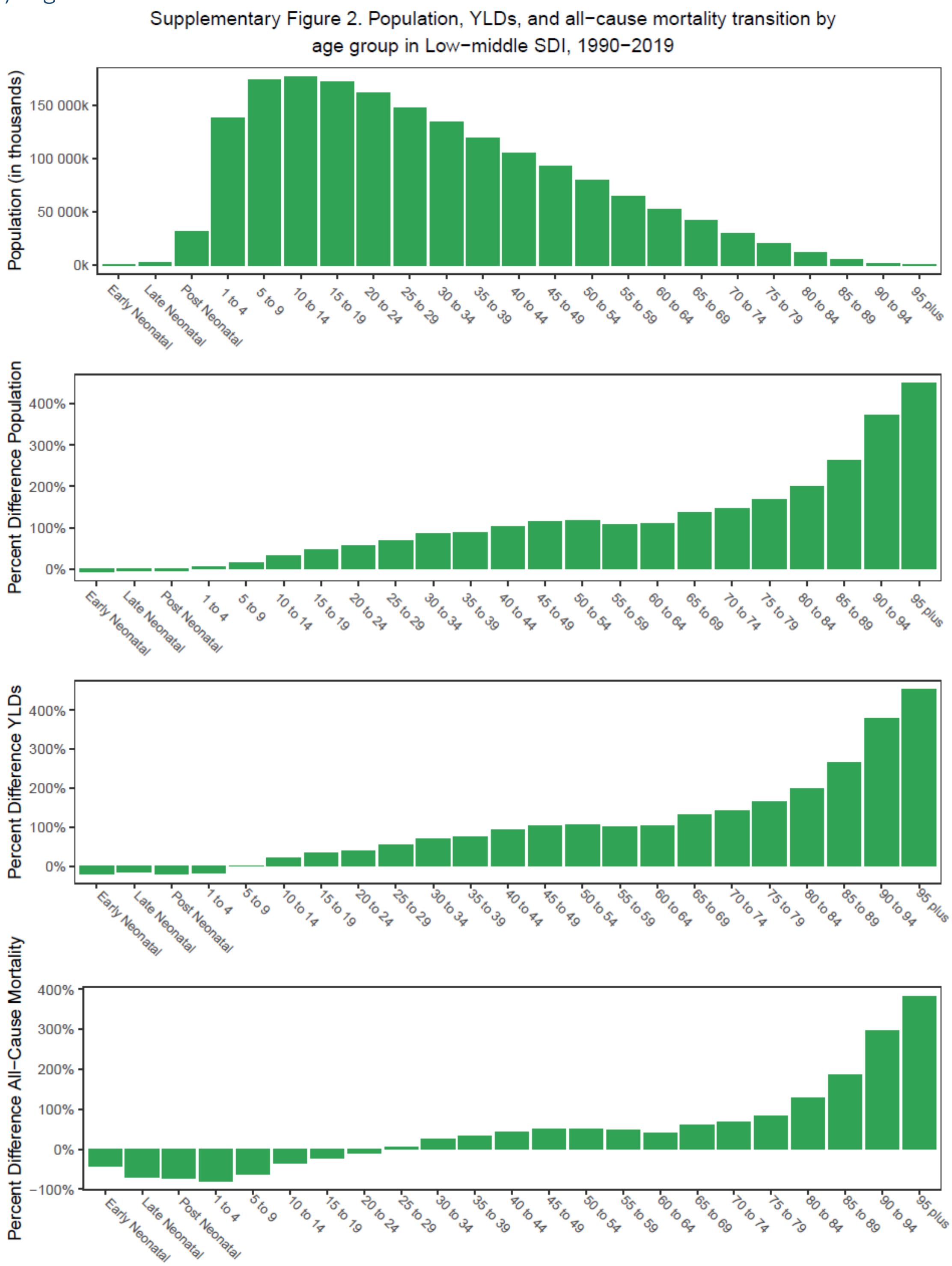

Figure S3. Global population change by age group for both sexes combined (top panel), population percentage change during the same period (upper middle panel), rate of change in years lived with disability (YLDs; lowermiddle panel), and rate of change in All-cause mortality (bottom panel) between 1990 and 2019 in Middle Socio-demographic Index (SDI) regions.

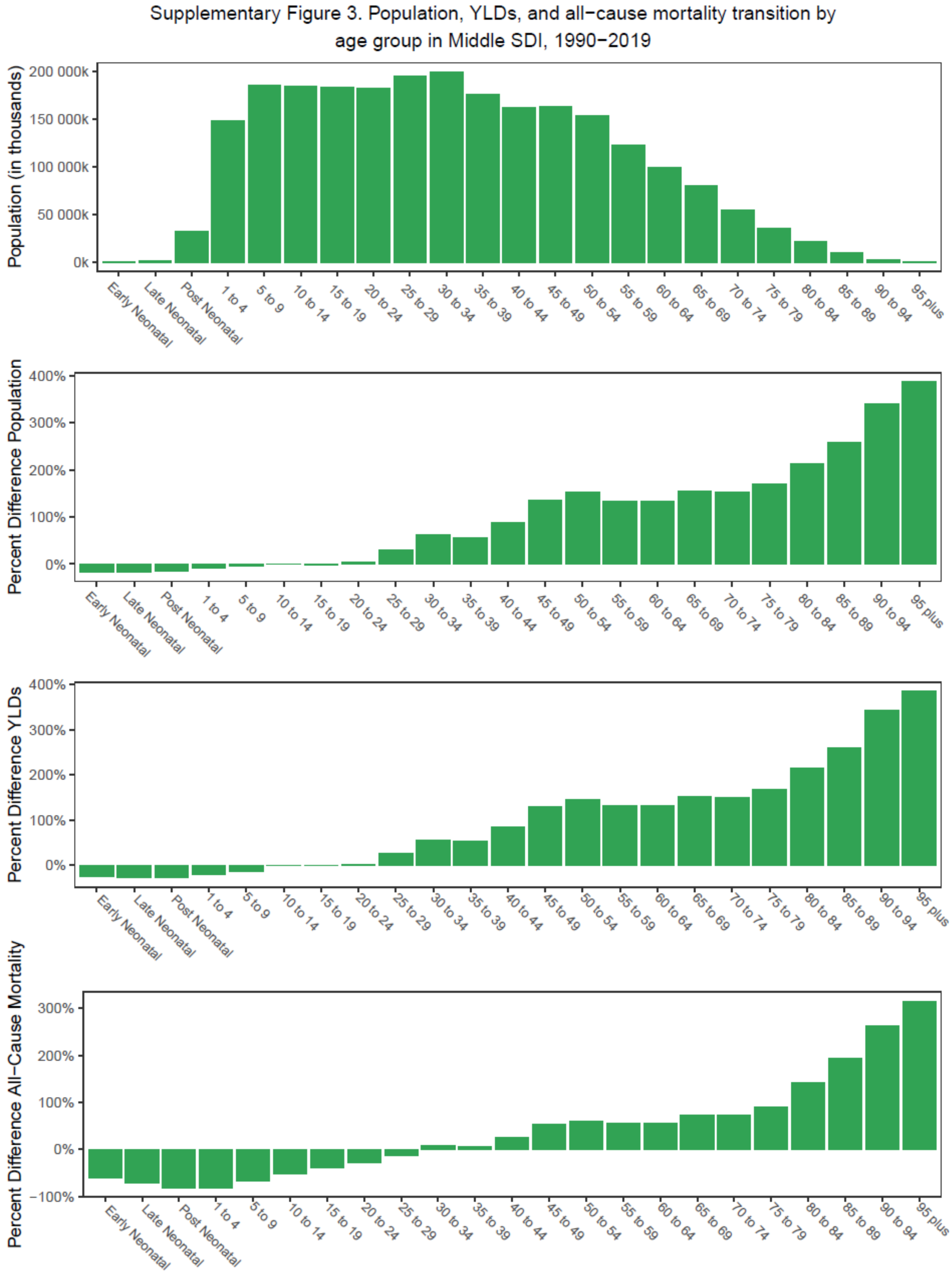

Figure S4. Global population change by age group for both sexes combined (top panel), population percentagechange during the same period (upper middle panel), rate of change in years lived with disability (YLDs; lower middle panel), and rate of change in All-cause mortality (bottom panel) between 1990 and 2019 in High-middle Socio-demographic Index (SDI) regions.

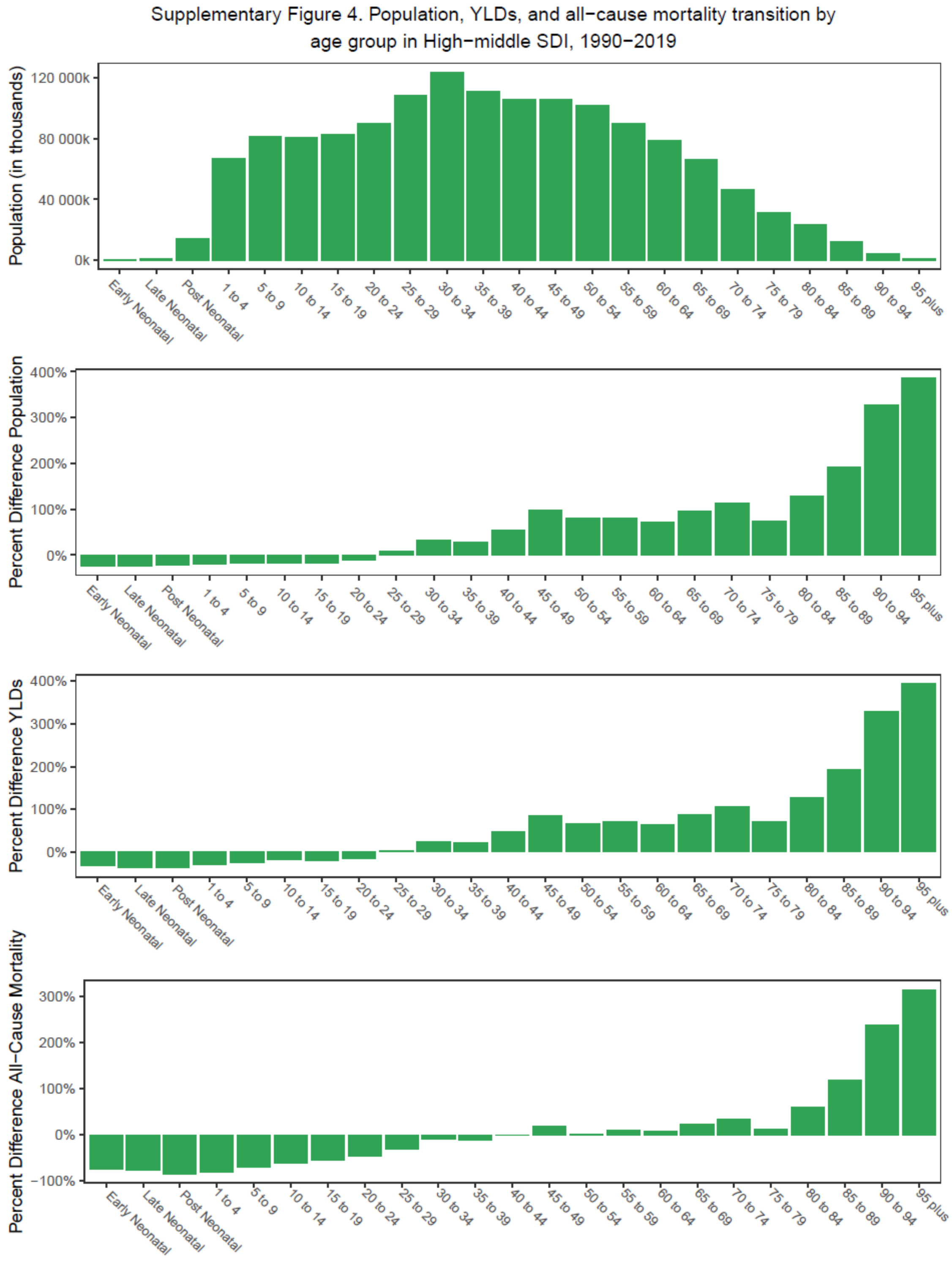

Figure S5. Global population change by age group for both sexes combined (top panel), population percentage change during the same period (upper middle panel), rate of change in years lived with disability (YLDs; lower middle panel), and rate of change in All-cause mortality (bottom panel) between 1990 and 2019 in High Socio- demographic Index (SDI) regions.

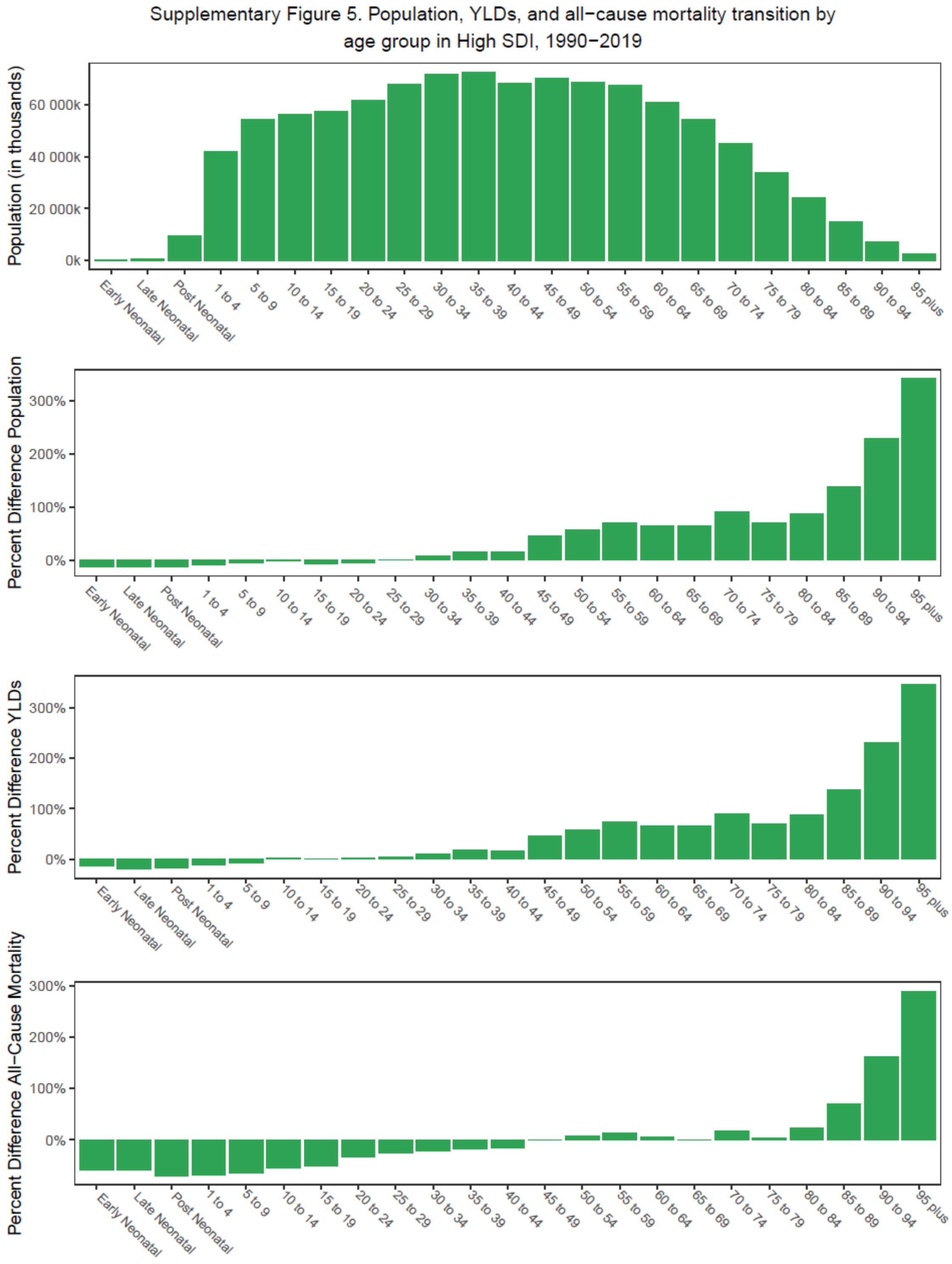

Figure S6. Global population change by age group for both sexes combined (top panel), population percentage change during the same period (upper middle panel), rate of change in years lived with disability (YLDs; lower middle panel), and rate of change in All-cause mortality (bottom panel) between 1990 and 2019 in the Central Europe, Eastern Europe and Central Asia GBD super-region.

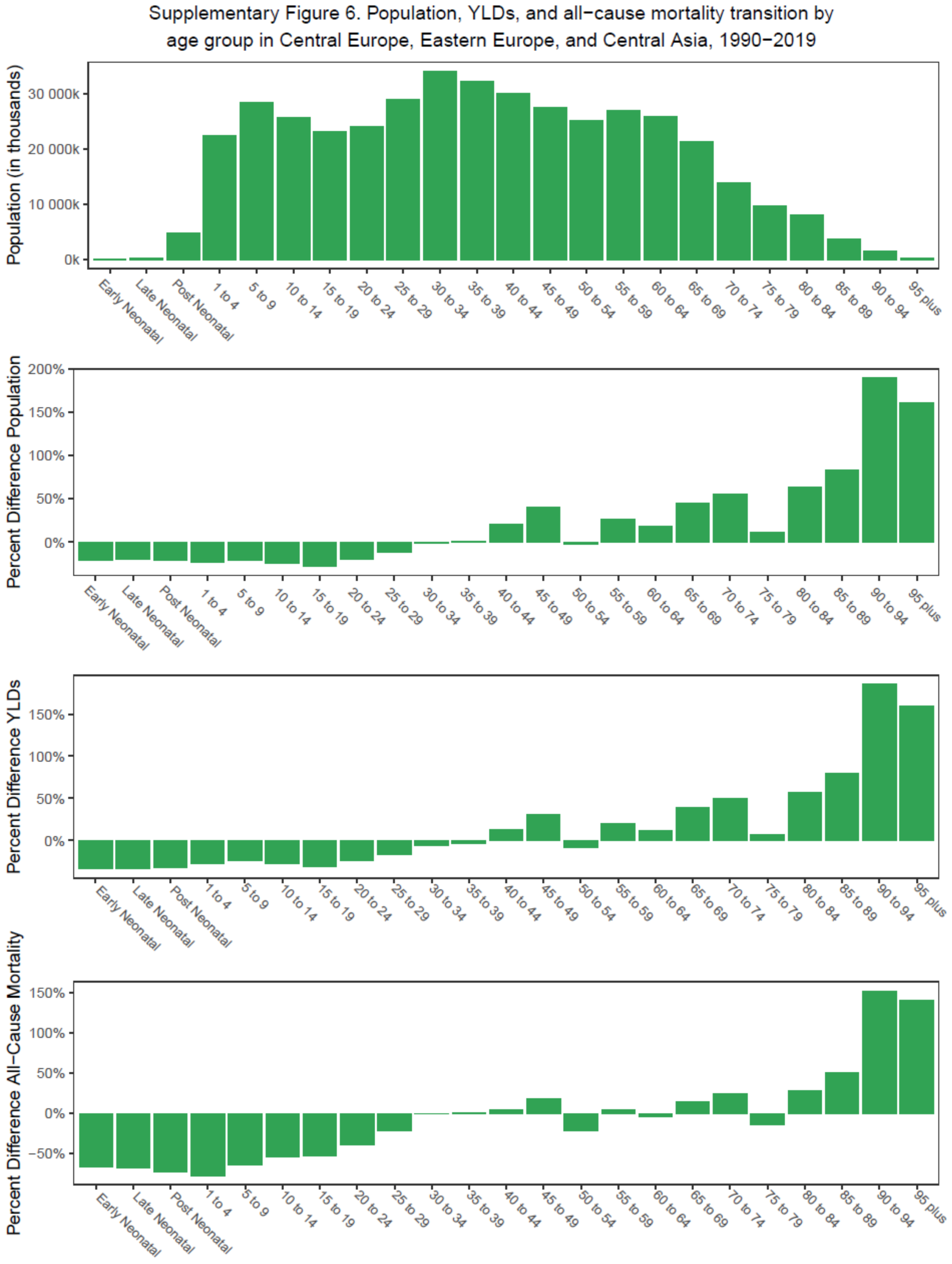

Figure S7. Global population change by age group for both sexes combined (top panel), population percentage change during the same period (upper middle panel), rate of change in years lived with disability (YLDs; lower middle panel), and rate of change in All-cause mortality (bottom panel) between 1990 and 2019 in the High- income GBD super-region.

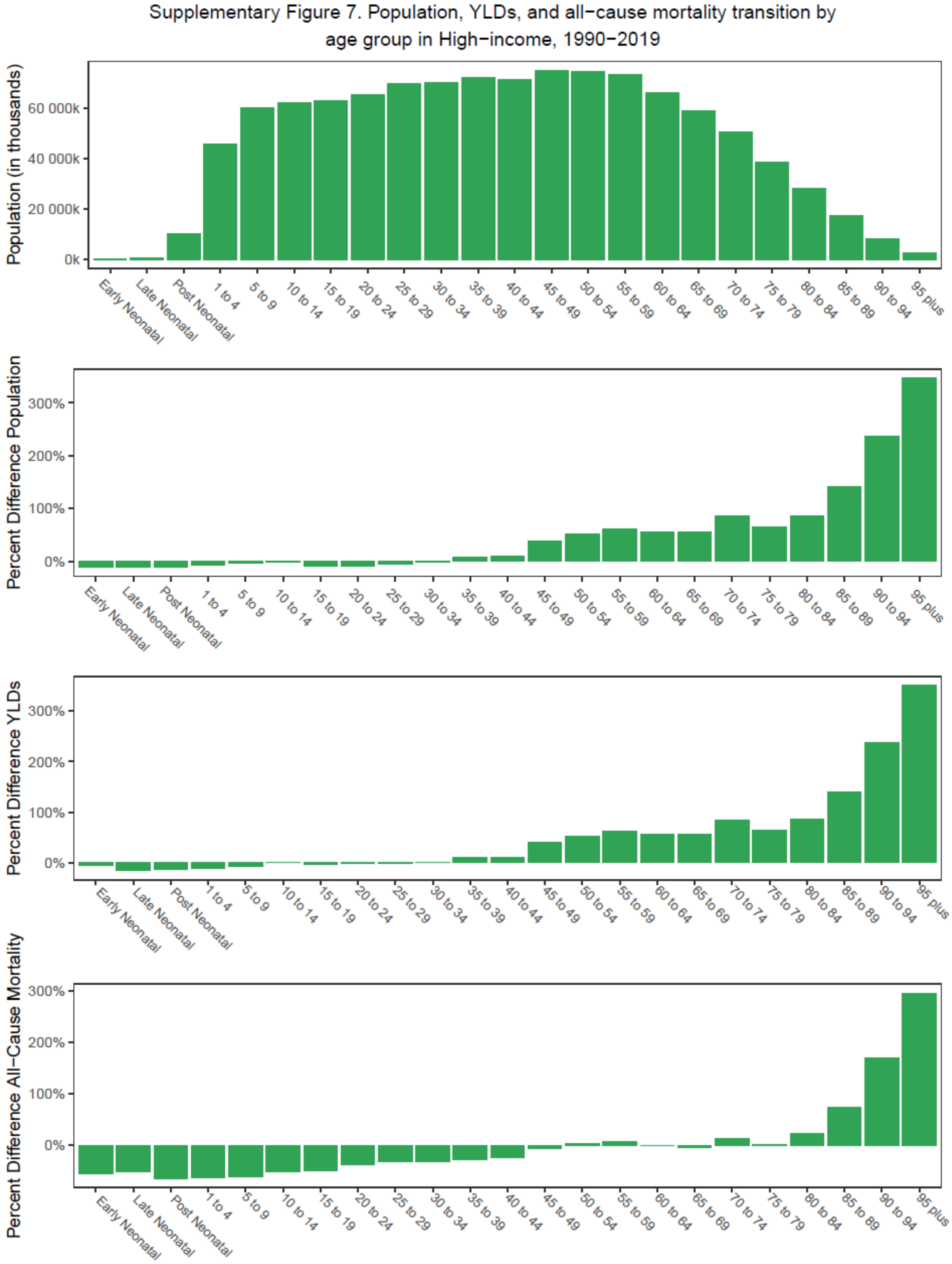

Figure S8. Global population change by age group for both sexes combined (top panel), population percentage change during the same period (upper middle panel), rate of change in years lived with disability (YLDs; lower middle panel), and rate of change in All-cause mortality (bottom panel) between 1990 and 2019 in the Latin America and Caribbean GBD super-region.

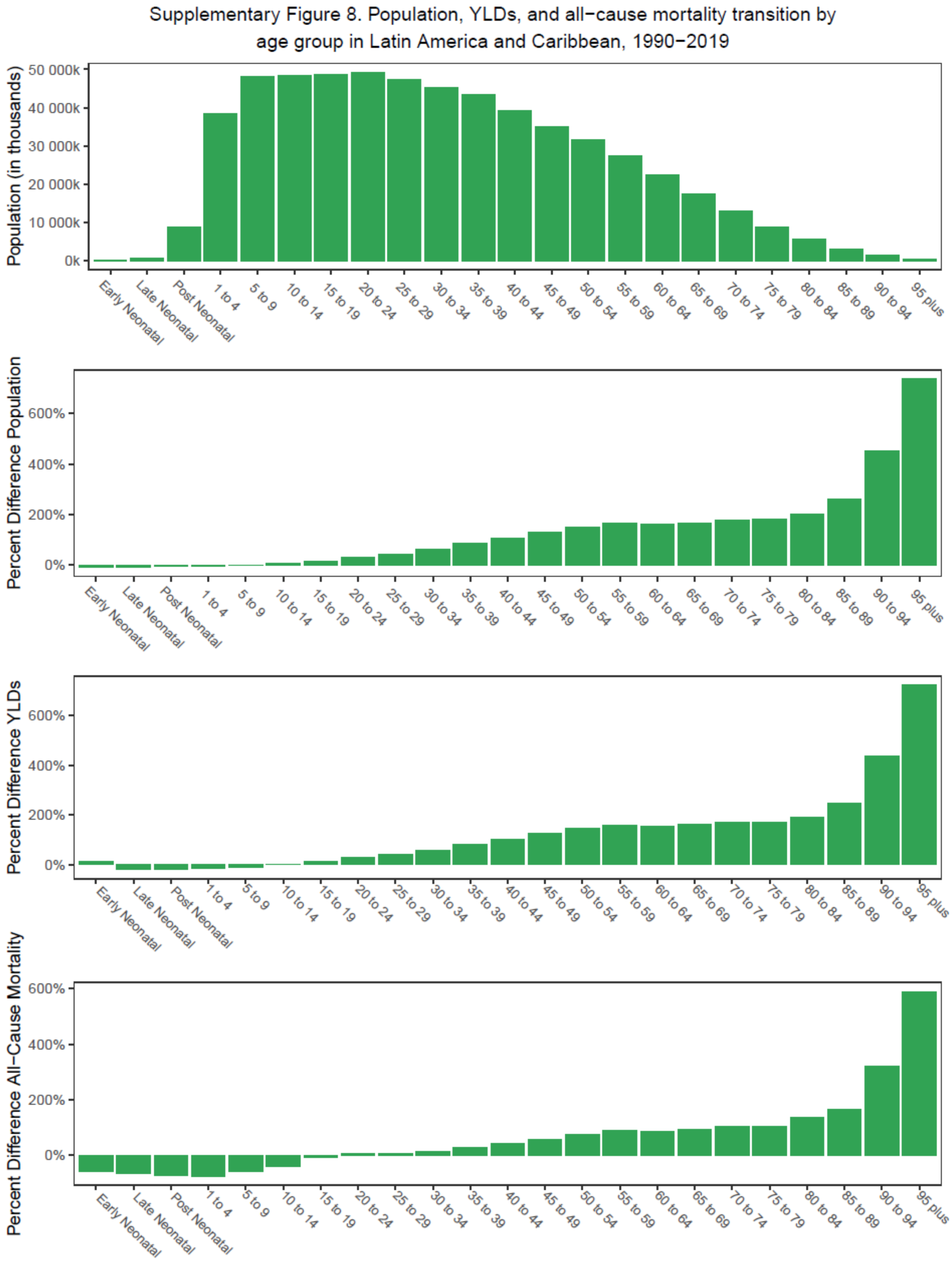

Figure S9. Global population change by age group for both sexes combined (top panel), population percentage change during the same period (upper middle panel), rate of change in years lived with disability (YLDs; lower middle panel), and rate of change in All-cause mortality (bottom panel) between 1990 and 2019 in the North Africa and Middle East super-region.

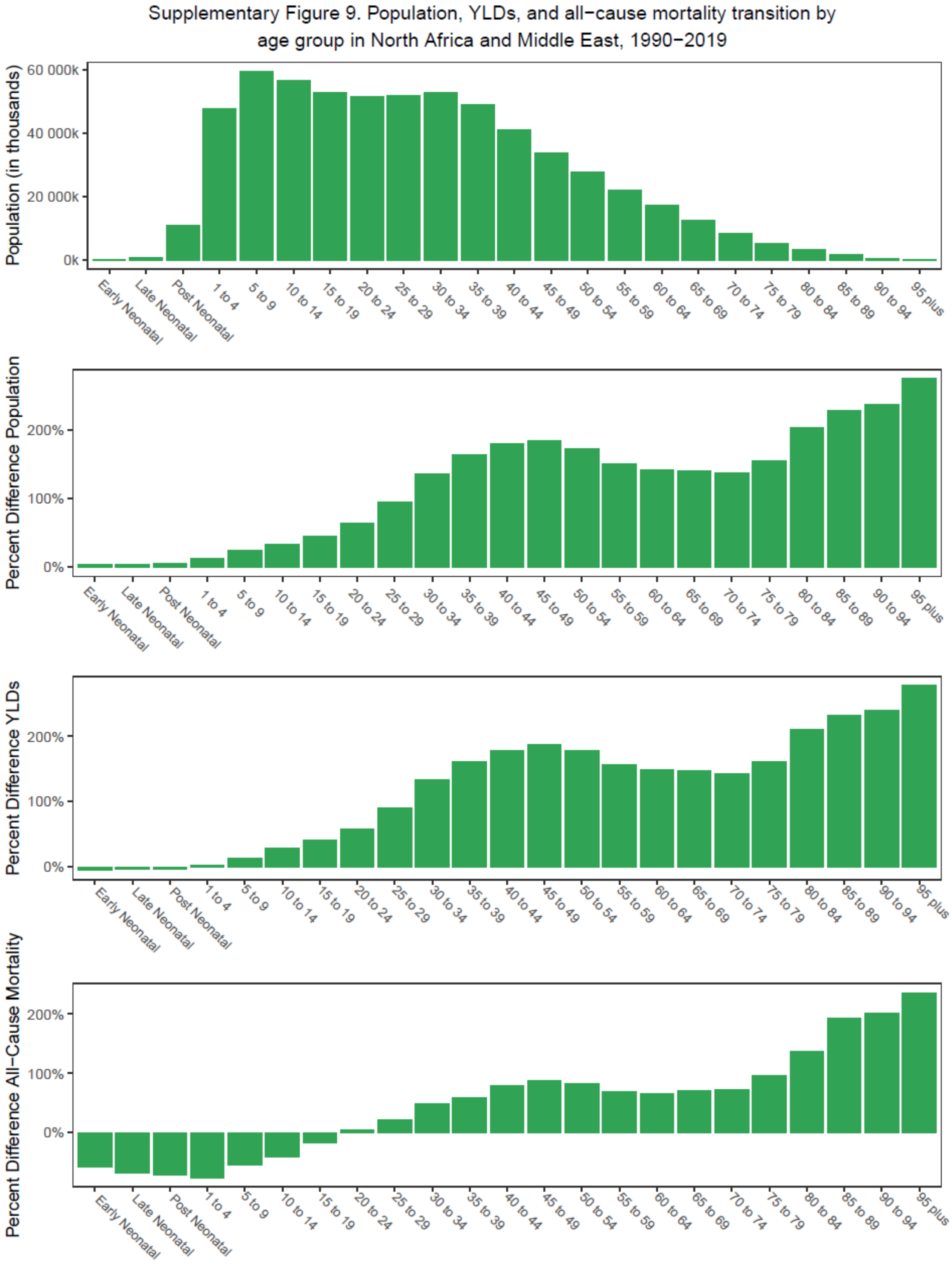

Figure S10. Global population change by age group for both sexes combined (top panel), population percentage change during the same period (upper middle panel), rate of change in years lived with disability (YLDs; lower middle panel), and rate of change in All-cause mortality (bottom panel) between 1990 and 2019 in the South Asia GBD super-region.

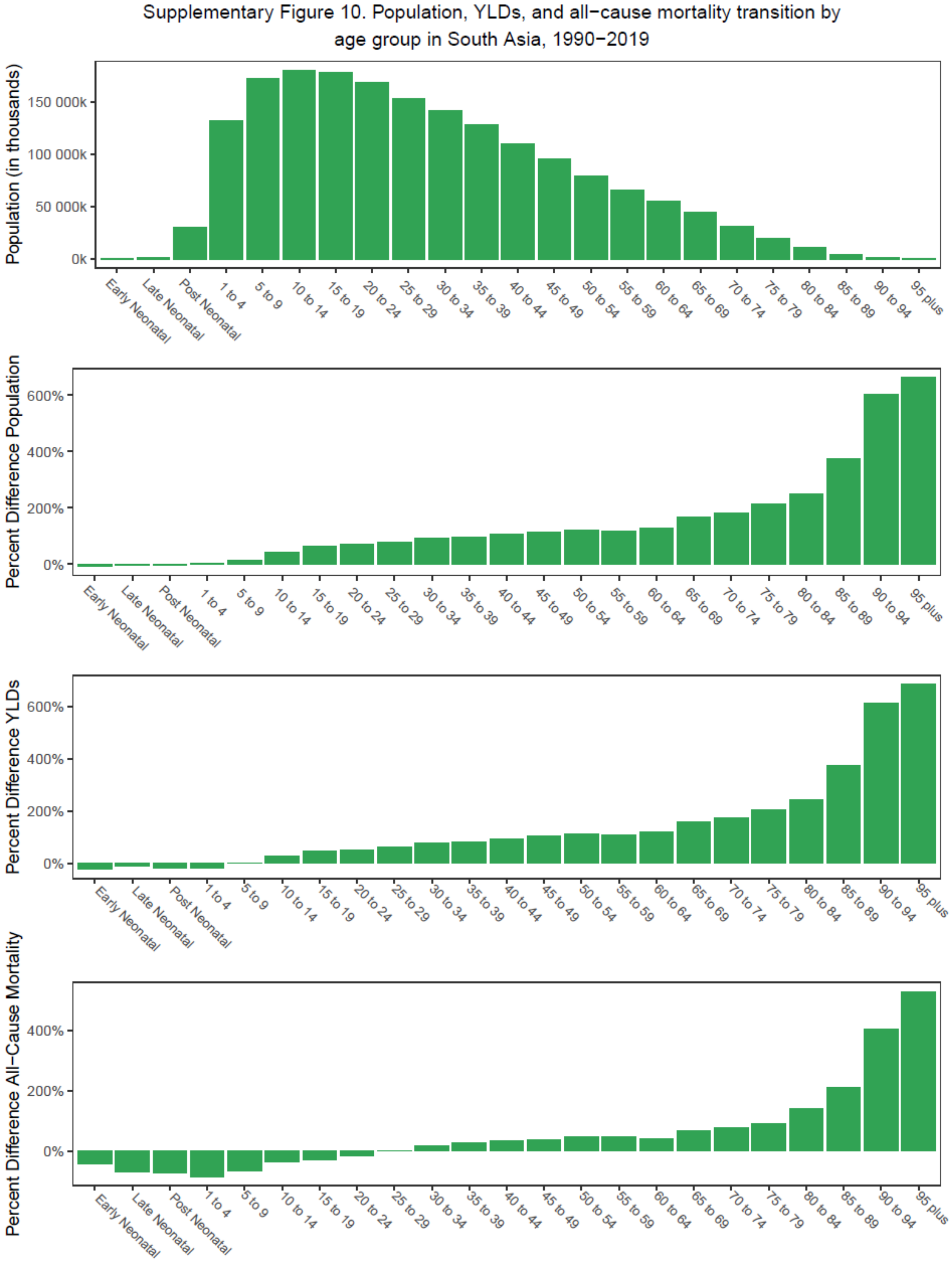

Figure S11. Global population change by age group for both sexes combined (top panel), population percentage change during the same period (upper middle panel), rate of change in years lived with disability (YLDs; lower middle panel), and rate of change in All-cause mortality (bottom panel) between 1990 and 2019 inthe Southeast Asia, East Asia and Oceania GBD super-region.

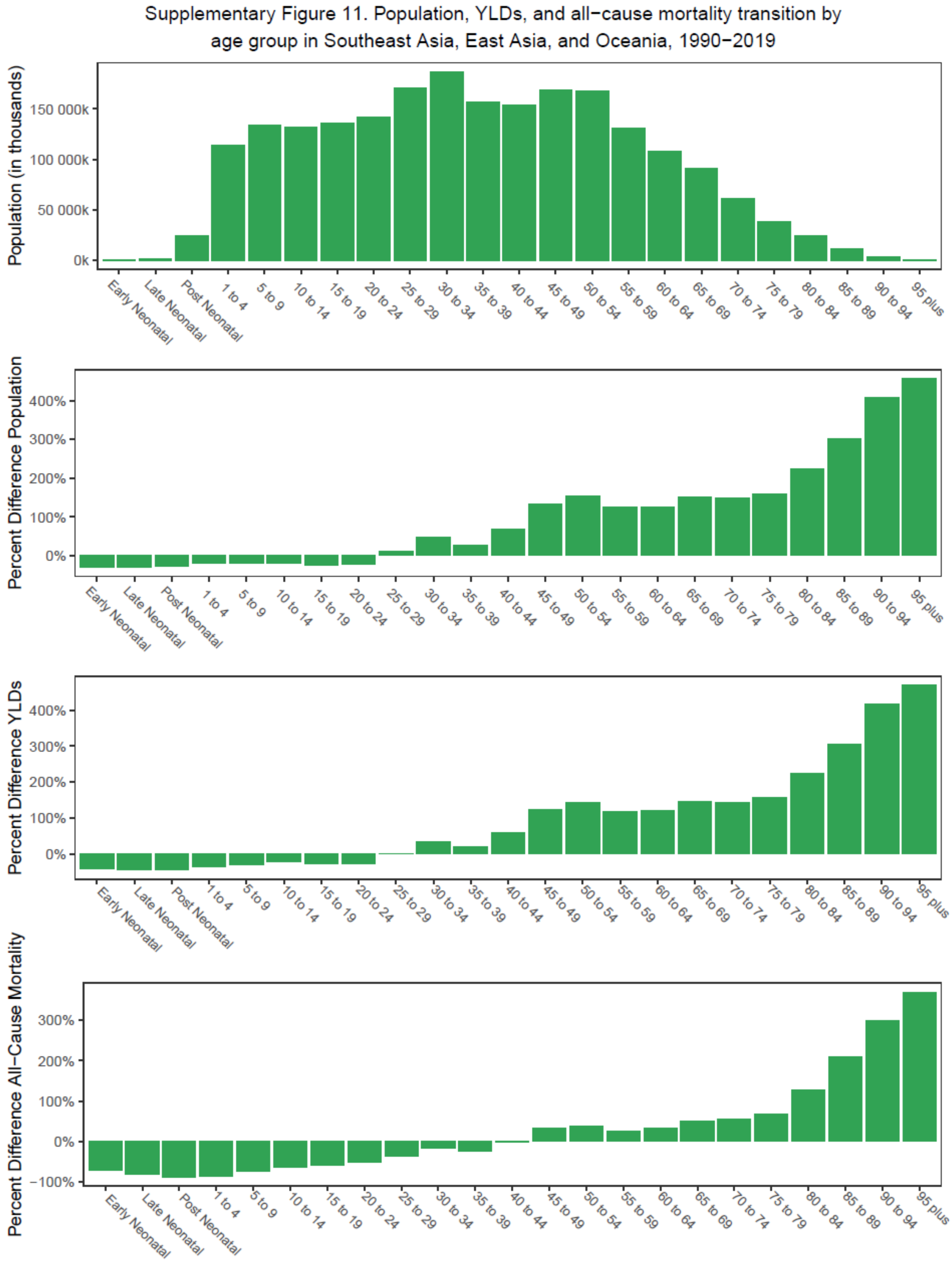

Figure S12. Global population change by age group for both sexes combined (top panel), population percentage change during the same period (upper middle panel), rate of change in years lived with disability (YLDs; lower middle panel), and rate of change in All-cause mortality (bottom panel) between 1990 and 2019 in the Sub-Saharan Africa GBD super-region

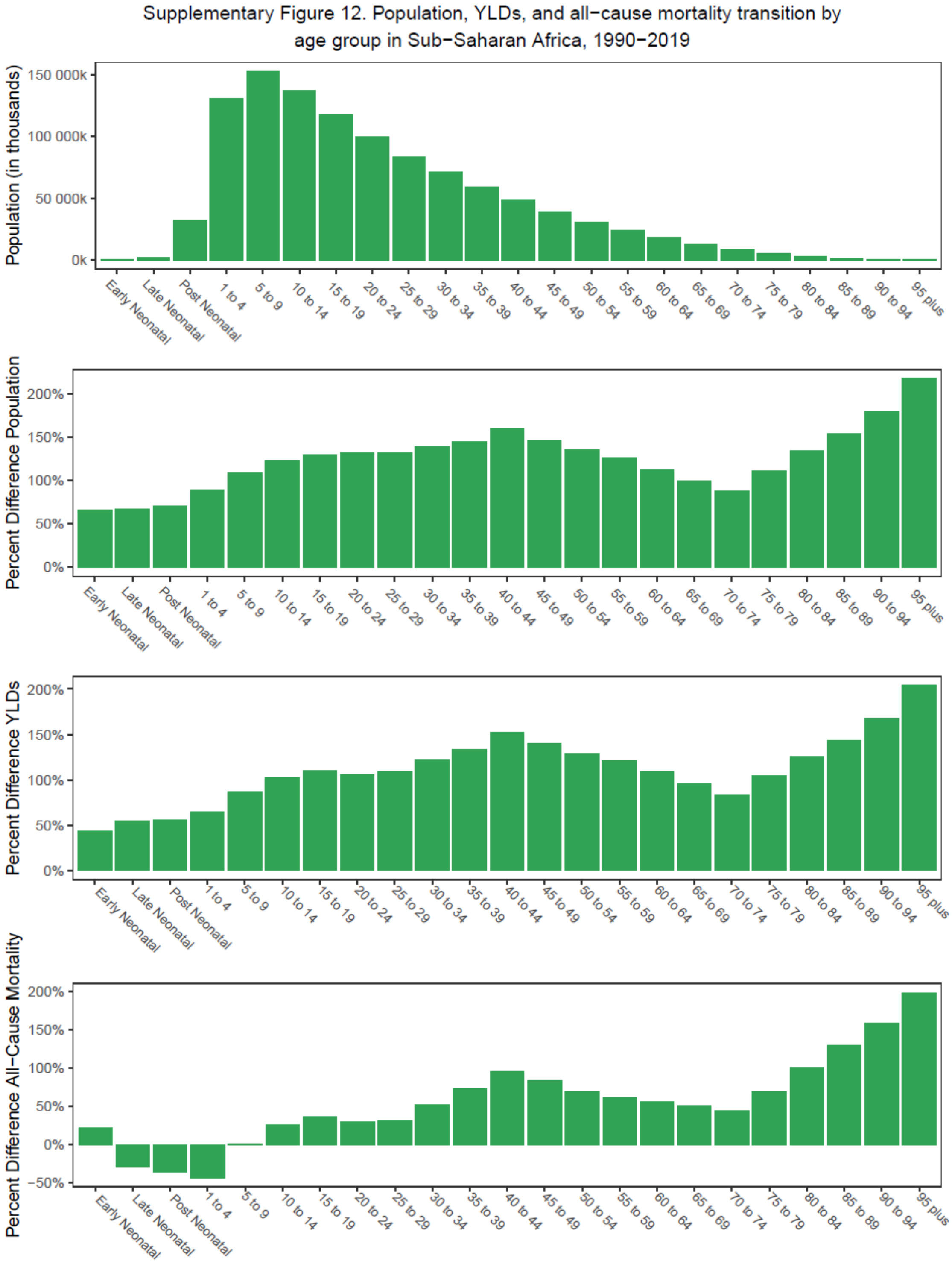

Figure S13. Life expectancy at 70+ (LE-70), healthy life expectancy at 70+ (HALE-70), and proportion of life- years spent in ill health (PYIH-70) at age 70+ GBD by super-region between 1990 and 2019, in males.

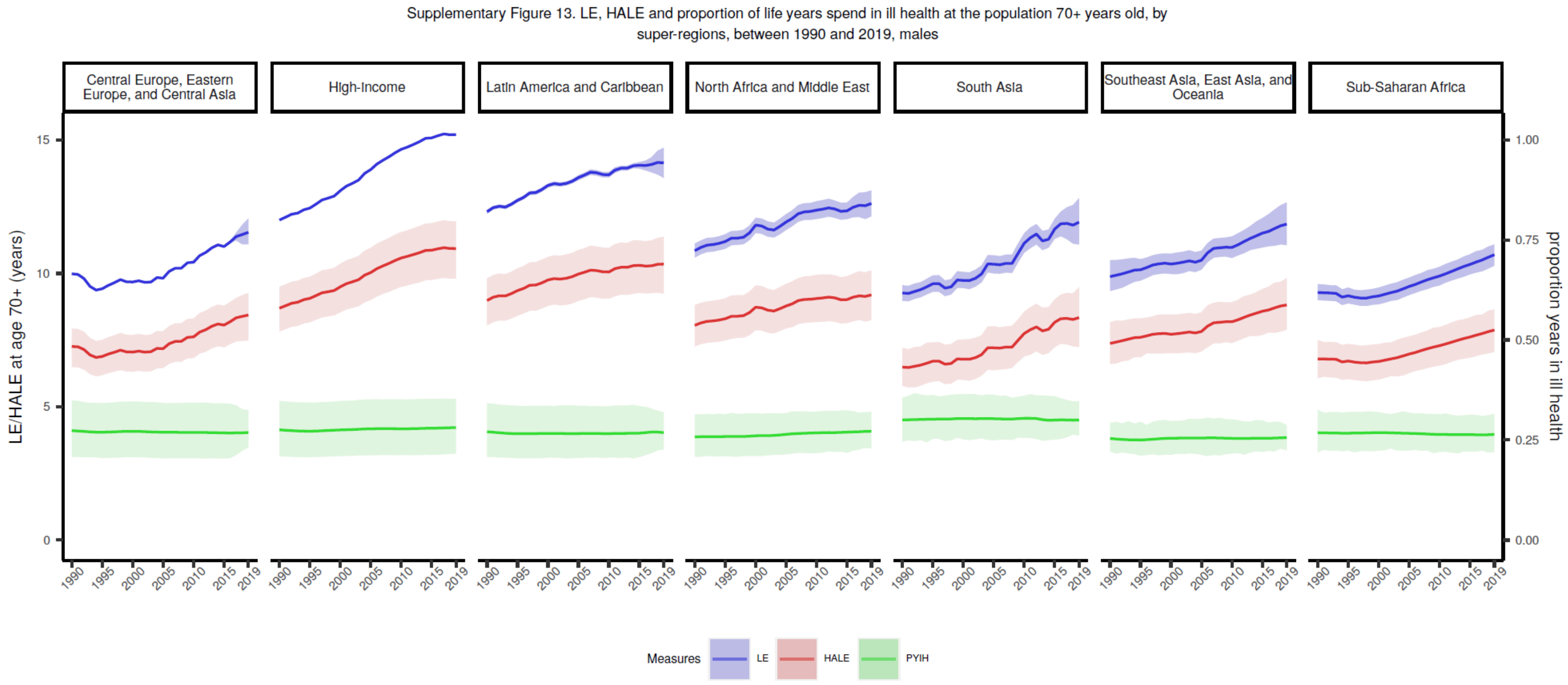

Figure S14. Life expectancy at 70+ (LE-70), healthy life expectancy at 70+ (HALE-70), and proportion of life- years spent in ill health (PYIH-70) at age 70+ GBD by super-region between 1990 and 2019, in females.

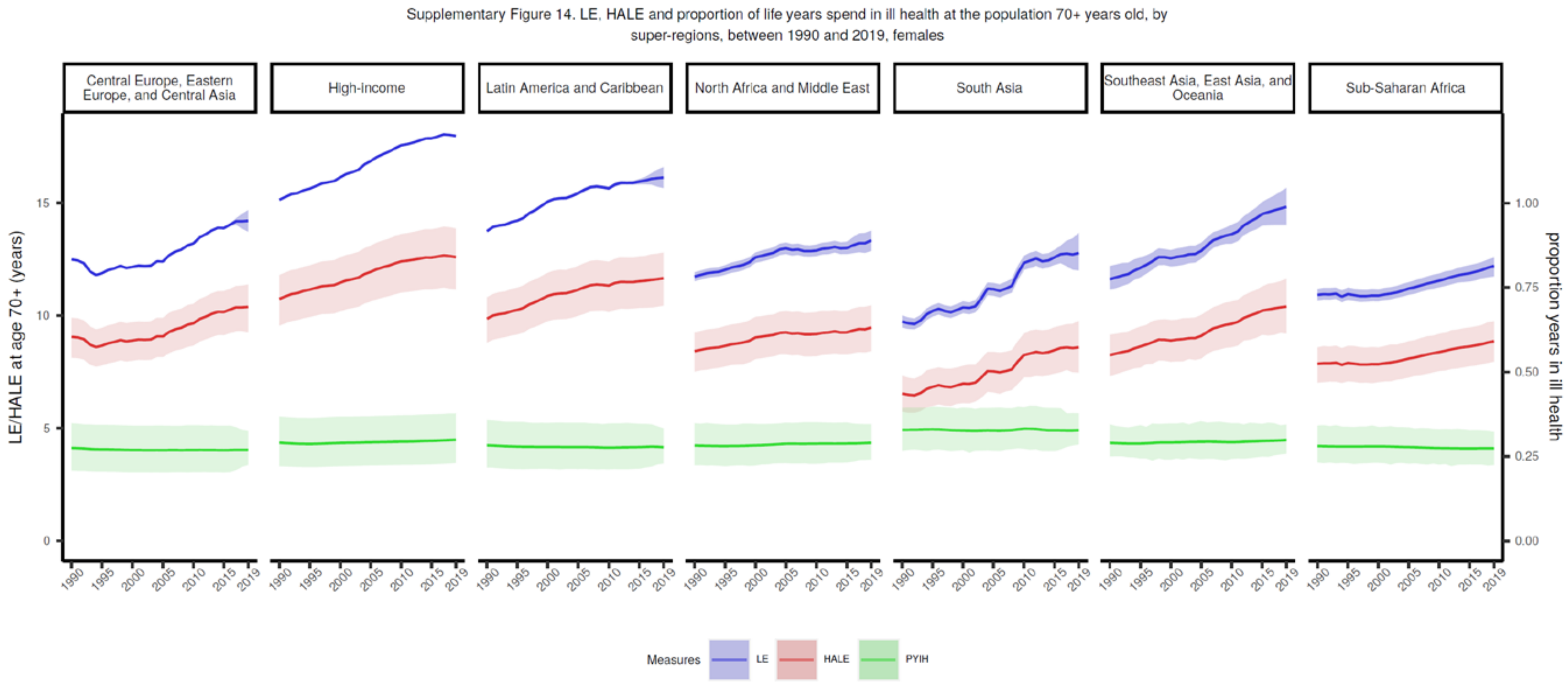

Figure S15. Life expectancy (LE-70), healthy life expectancy (HALE-70) and proportion of life-years spent in ill health at age 70+ (PYIH-70) versus Socio-demographic Index (SDI) and Healthcare Access and Quality (HAQ) Index in 2019, in males.

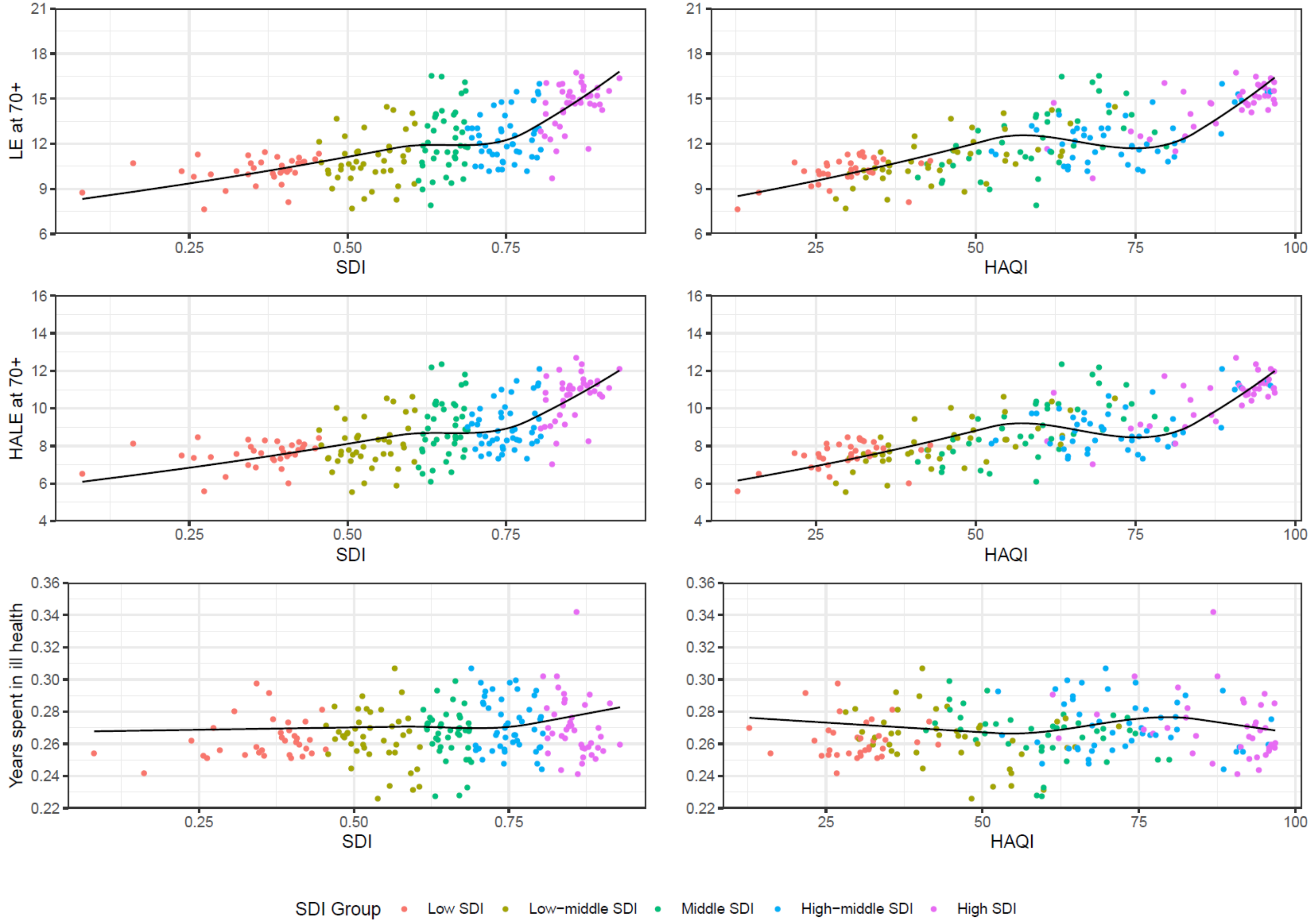

Figure S16. Life expectancy (LE-70), healthy life expectancy (HALE-70) and proportion of life-years spent in ill health at age 70+ (PYIH-70) versus Socio-demographic Index (SDI) and Healthcare Access and Quality (HAQ) Index in 2019, in females.

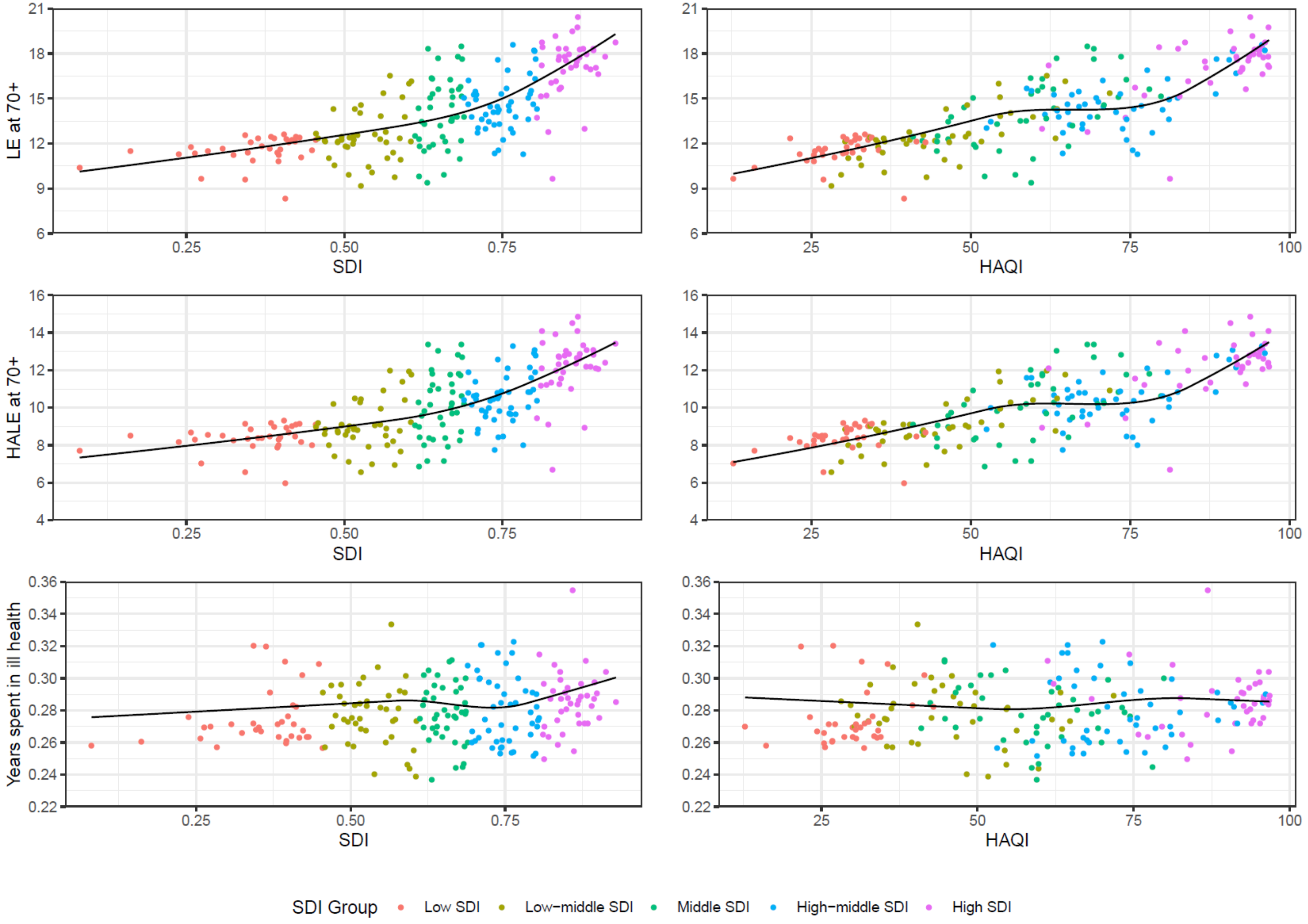

Figure S17. Decomposition of the probability of death, for all GBD locations, for the population aged 70to 90 years old, from 1990 to 2019, in both sexes.

Both sexes refer to combined estimates for males and females. Probability of death between 70 and 90 years of age (denoted using standard demographer notation as 20q70, the probability 'q' of death for a period of 20 years starting at age 70) is plotted globally and by Global Burden of Disease super-region and region for 1990 (dashed vertical line) and 2019 (solid vertical line). The relative contribution of that change due to different causes is indicated by different coloured bars. All causes to the right of the dashed line increased from 1990 to 2019 and all those to the left decreased over the same time period. As can be seen at the global level, the probability of death for those 70 to 90 years decreased mainly due to cardiovascular diseases, chronic respiratory diseases, respiratory infections and tuberculosis and enteric infections (-13.6% in aggregate), while probability of death increased owing to increases in neoplasms, neurological disorders, and diabetes and kidney diseases (+4.3% in aggregate). ***COLOR LEGEND:***

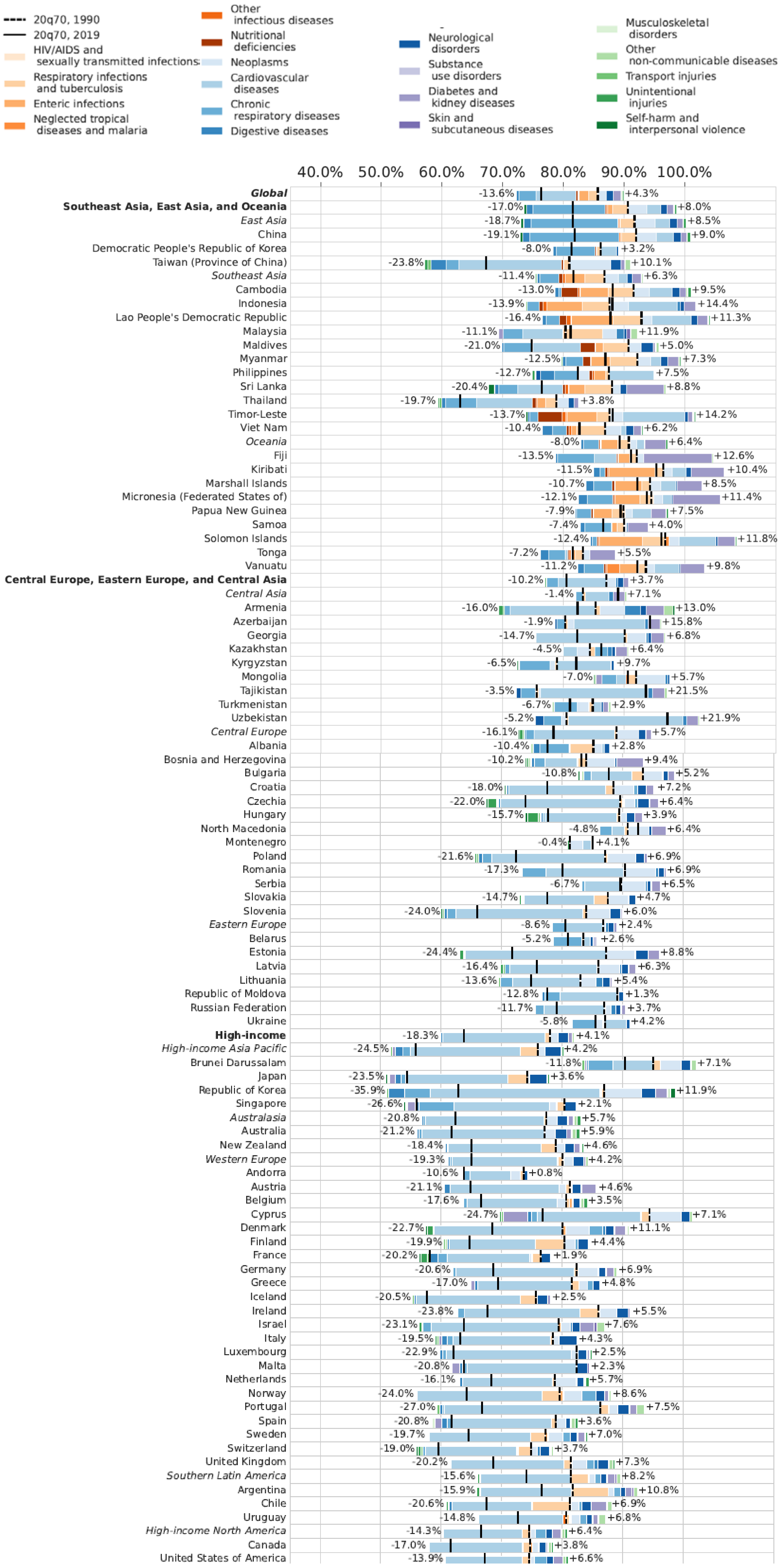

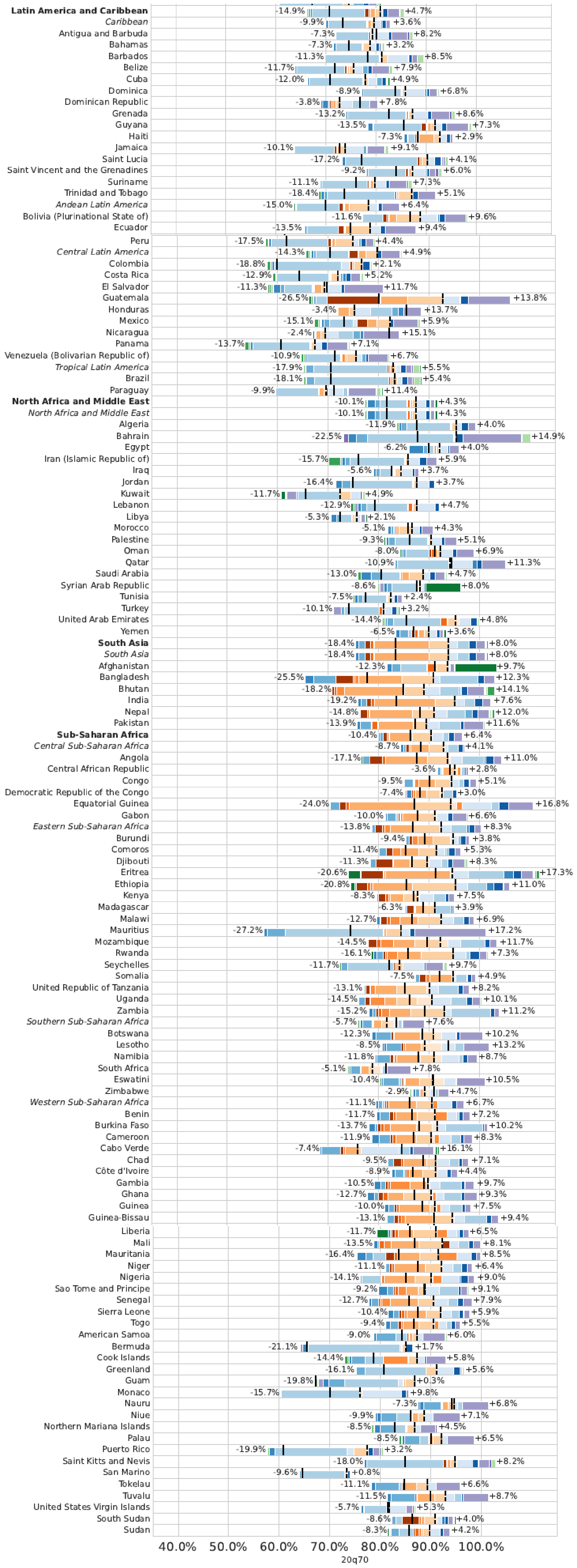
Latin America and Caribbean -14.9% +4.7%
Caribbean -9.9% +3.6%
Antigua and Barbuda -7.3% +8.2%
Bahamas -7.3% +3.2%
Barbados -11.3% +8.5%
Belize -11.7% +7.9%
Cuba -12.0% +4.9%
Dominica -8.9% +6.8%
Dominican Republic -3.8% +7.8%
Grenada -13.2% +8.6%
Guyana -13.5% +7.3%
Haiti -7.3% +2.9%
Jamaica -10.1% +9.1%
Saint Lucia -17.2% +4.1%
Saint Vincent and the Grenadines -9.2% +6.0%
Suriname -11.1% +7.3%
Trinidad and Tobago -18.4% +5.1%
Andean Latin America -15.0% +6.4%
Bolivia (Plurinational State of) -11.6% +9.6%
Ecuador -13.5% +9.4%
Peru -17.5% +4.4%
Central Latin America -14.3% +4.9%
Colombia -18.8% +2.1%
Costa Rica -12.9% +5.2%
El Salvador -11.3% +11.7%
Guatemala -26.5% +13.8%
Honduras -3.4% +13.7%
Mexico -15.1% +5.9%
Nicaragua -2.4% +15.1%
Panama -13.7% +7.1%
Venezuela (Bolivarian Republic of) -10.9% +6.7%
Tropical Latin America -17.9% +5.5%
Brazil -18.1% +5.4%
Paraguay -9.9% +11.4%
North Africa and Middle East -10.1% +4.3%
North Africa and Middle East -10.1% +4.3%
Algeria -11.9% +4.0%
Bahrain -22.5% +14.9%
Egypt -6.2% +4.0%
Iran (Islamic Republic of) -15.7% +5.9%
Iraq -5.6% +3.7%
Jordan -16.4% +3.7%
Kuwait -11.7% +4.9%
Lebanon -12.9% +4.7%
Libya -5.3% +2.1%
Morocco -5.1% +4.3%
Palestine -9.3% +5.1%
Oman -8.0% +6.9%
Qatar -10.9% +11.3%
Saudi Arabia -13.0% +4.7%
Syrian Arab Republic -8.6% +8.0%
Tunisia -7.5% +2.4%
Turkey -10.1% +3.2%
United Arab Emirates -14.4% +4.8%
Yemen -6.5% +3.6%
South Asia -18.4% +8.0%
South Asia -18.4% +8.0%
Afghanistan -12.3% +9.7%
Bangladesh -25.5% +12.3%
Bhutan -18.2% +14.1%
India -19.2% +7.6%
Nepal -14.8% +12.0%
Pakistan -13.9% +11.6%
Sub-Saharan Africa -10.4% +6.4%
Central Sub-Saharan Africa -8.7% +4.1%
Angola -17.1% +11.0%
Central African Republic -3.6% +2.8%
Congo -9.5% +5.1%
Democratic Republic of the Congo -7.4% +3.0%
Equatorial Guinea -24.0% +16.8%
Gabon -10.0% +6.6%
Eastern Sub-Saharan Africa -13.8% +8.3%
Burundi -9.4% +3.8%
Comoros -11.4% +5.3%
Djibouti -11.3% +8.3%
Eritrea -20.6% +17.3%
Ethiopia -20.8% +11.0%
Kenya -8.3% +7.5%
Madagascar -6.3% +3.9%
Malawi -12.7% +6.9%
Mauritius -27.2% +17.2%
Mozambique -14.5% +11.7%
Rwanda -16.1% +7.3%
Seychelles -11.7% +9.7%
Somalia -7.5% +4.9%
United Republic of Tanzania -13.1% +8.2%
Uganda -14.5% +10.1%
Zambia -15.2% +11.2%
Southern Sub-Saharan Africa -5.7% +7.6%
Botswana -12.3% +10.2%
Lesotho -8.5% +13.2%
Namibia -11.8% +8.7%
South Africa -5.1% +7.8%
Eswatini -10.4% +10.5%
Zimbabwe -2.9% +4.7%
Western Sub-Saharan Africa -11.1% +6.7%
Benin -11.7% +7.2%
Burkina Faso -13.7% +10.2%
Cameroon -11.9% +8.3%
Cabo Verde -7.4% +16.1%
Chad -9.5% +7.1%
Côte d'Ivoire -8.9% +4.4%
Gambia -10.5% +9.7%
Ghana -12.7% +9.3%
Guinea -10.0% +7.5%
Guinea-Bissau -13.1% +9.4%
Liberia -11.7% +6.5%
Mali -13.5% +8.1%
Mauritania -16.4% +8.5%
Niger -11.1% +6.4%
Nigeria -14.1% +9.0%
Sao Tome and Principe -9.2% +9.1%
Senegal -12.7% +7.9%
Sierra Leone -10.4% +5.9%
Togo -9.4% +5.5%
American Samoa -9.0% +6.0%
Bermuda -21.1% +1.7%
Cook Islands -14.4% +5.8%
Greenland -16.1% +5.6%
Guam -19.8% +0.3%
Monaco -15.7% +9.8%
Nauru -7.3% +6.8%
Niue -9.9% +7.1%
Northern Mariana Islands -8.5% +4.5%
Palau -8.5% +6.5%
Puerto Rico -19.9% +3.2%
Saint Kitts and Nevis -18.0% +8.2%
San Marino -9.6% +0.8%
Tokelau -11.1% +6.6%
Tuvalu -11.5% +8.7%
United States Virgin Islands -5.7% +5.3%
South Sudan -8.6% +4.0%
Sudan -8.3% +4.2%
40.0% 50.0% 60.0% 70.0% 80.0% 90.0% 100.0%
20q70

Figure S18. Decomposition of the probability of death, for all GBD locations, for the population aged 70 to 90 years old, from 1990 to 2019, in males

Probability of death between 70 and 90 years of age (denoted using standard demographer notation as 20q70, the probability 'q' of death for a period of 20 years starting at age 70) is plotted globally and by Global Burden of Disease super-region and region for 1990 (dashed vertical line) and 2019 (solid vertical line). The relative contribution of that change due to different causes is indicated by different coloured bars. All causes to the right of the dashed line increased from 1990 to 2019 and all those to the left decreased over the same time period. As can be seen at the global level, the probability of death for the males 70 to 90 years decreased mainly due to cardiovascular diseases, chronic respiratory diseases, respiratory infections and tuberculosis and enteric infections (-12.7% in aggregate), while probability of death increased owing to increases in neoplasms, neurological disorders, and diabetes and kidney diseases (+4.8% in aggregate). COLOR LEGEND:

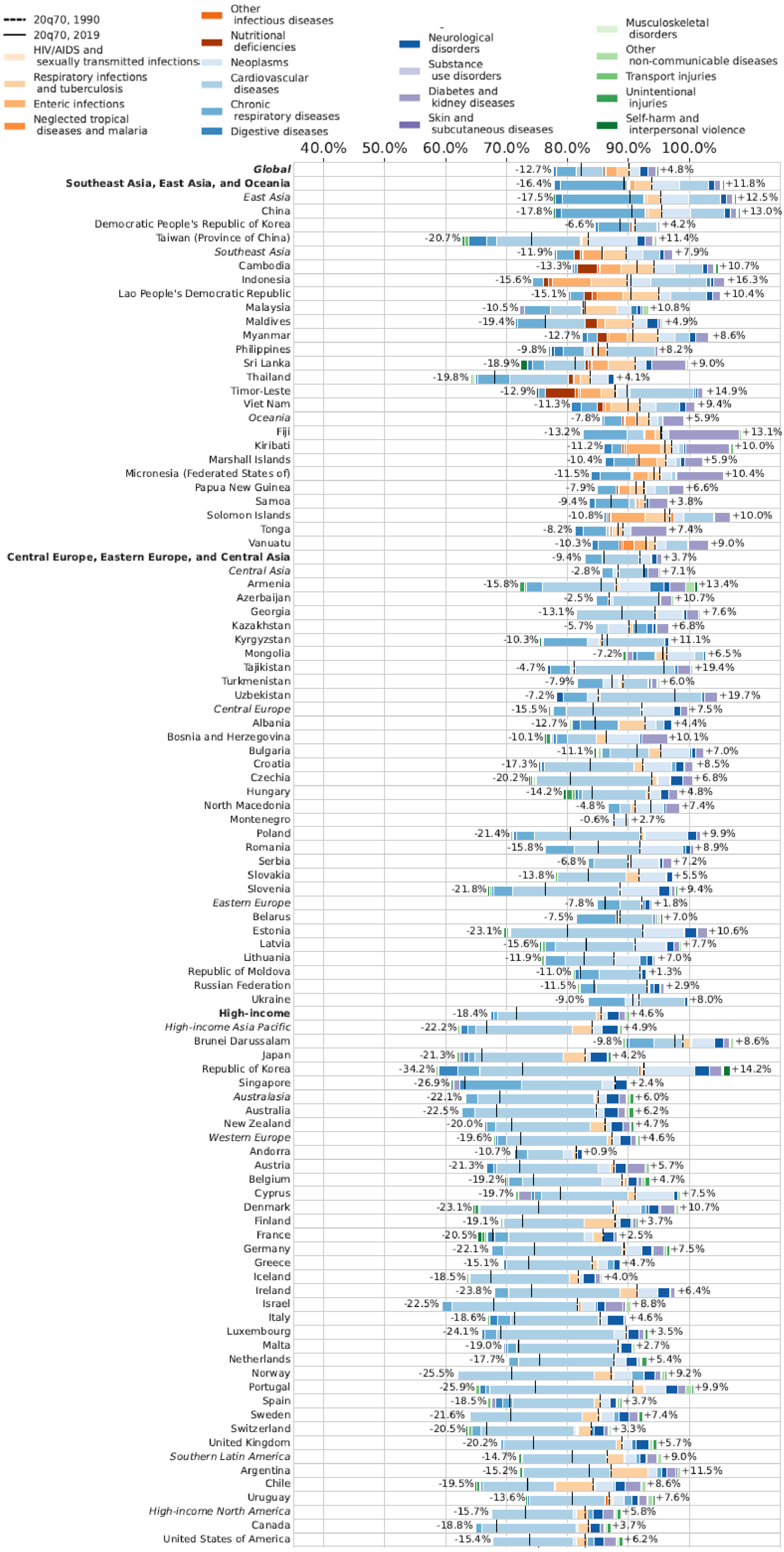

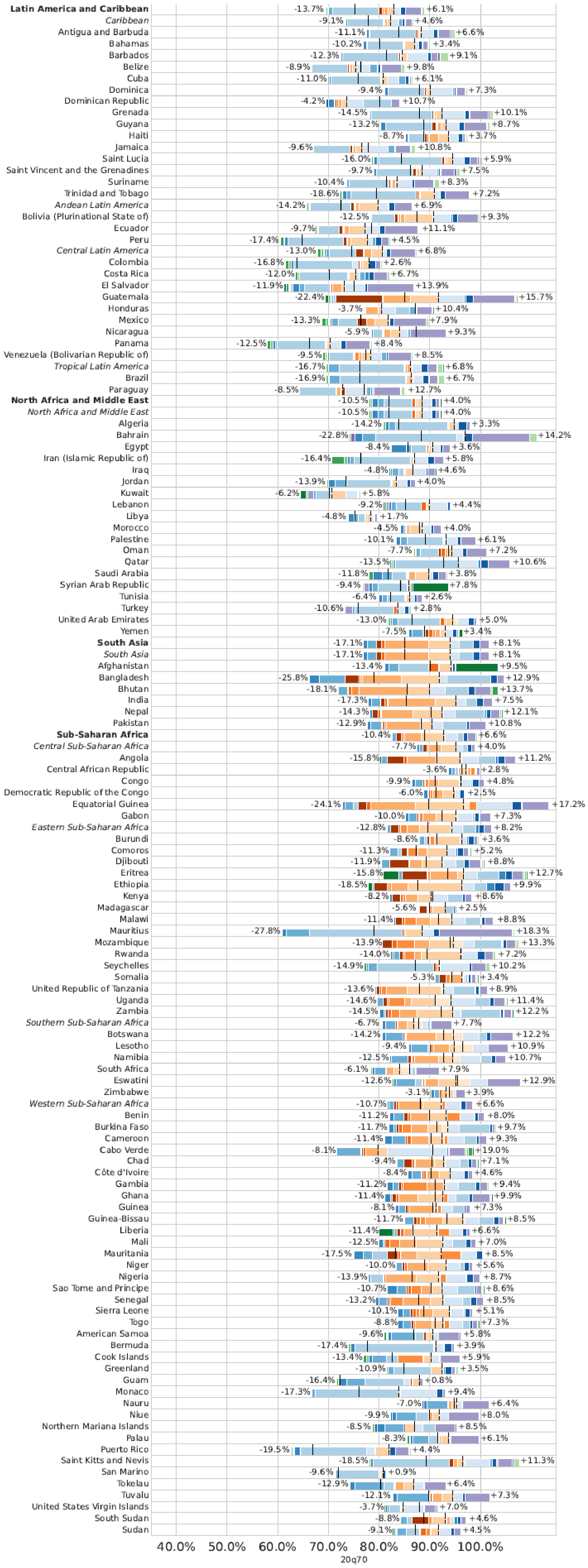
Latin America and Caribbean -13.7% +6.1%
Caribbean -9.1% +4.6%
Antigua and Barbuda -11.1% +6.6%
Bahamas -10.2% +3.4%
Barbados -12.3% +9.1%
Belize -8.9% +9.8%
Cuba -11.0% +6.1%
Dominica -9.4% +7.3%
Dominican Republic -4.2% +10.7%
Grenada -14.5% +10.1%
Guyana -13.2% +8.7%
Haiti -8.7% +3.7%
Jamaica -9.6% +10.8%
Saint Lucia -16.0% +5.9%
Saint Vincent and the Grenadines -9.7% +7.5%
Suriname -10.4% +8.3%
Trinidad and Tobago -18.6% +7.2%
Andean Latin America -14.2% +6.9%
Bolivia (Plurinational State of) -12.5% +9.3%
Ecuador -9.7% +11.1%
Peru -17.4% +4.5%
Central Latin America -13.0% +6.8%
Colombia -16.8% +2.6%
Costa Rica -12.0% +6.7%
El Salvador -11.9% +13.9%
Guatemala -22.4% +15.7%
Honduras -3.7% +10.4%
Mexico -13.3% +7.9%
Nicaragua -5.9% +9.3%
Panama -12.5% +8.4%
Venezuela (Bolivarian Republic of) -9.5% +8.5%
Tropical Latin America -16.7% +6.8%
Brazil -16.9% +6.7%
Paraguay -8.5% +12.7%
North Africa and Middle East -10.5% +4.0%
North Africa and Middle East -10.5% +4.0%
Algeria -14.2% +3.3%
Bahrain -22.8% +14.2%
Egypt -8.4% +3.6%
Iran (Islamic Republic of) -16.4% +5.8%
Iraq -4.8% +4.6%
Jordan -13.9% +4.0%
Kuwait -6.2% +5.8%
Lebanon -9.2% +4.4%
Libya -4.8% +1.7%
Morocco -4.5% +4.0%
Palestine -10.1% +6.1%
Oman -7.7% +7.2%
Qatar -13.5% +10.6%
Saudi Arabia -11.8% +3.8%
Syrian Arab Republic -9.4% +7.8%
Tunisia -6.4% +2.6%
Turkey -10.6% +2.8%
United Arab Emirates -13.0% +5.0%
Yemen -7.5% +3.4%
South Asia -17.1% +8.1%
South Asia -17.1% +8.1%
Afghanistan -13.4% +9.5%
Bangladesh -25.8% +12.9%
Bhutan -18.1% +13.7%
India -17.3% +7.5%
Nepal -14.3% +12.1%
Pakistan -12.9% +10.8%
Sub-Saharan Africa -10.4% +6.6%
Central Sub-Saharan Africa -7.7% +4.0%
Angola -15.8% +11.2%
Central African Republic -3.6% +2.8%
Congo -9.9% +4.8%
Democratic Republic of the Congo -6.0% +2.5%
Equatorial Guinea -24.1% +17.2%
Gabon -10.0% +7.3%
Eastern Sub-Saharan Africa -12.8% +8.2%
Burundi -8.6% +3.6%
Comoros -11.3% +5.2%
Djibouti -11.9% +8.8%
Eritrea -15.8% +12.7%
Ethiopia -18.5% +9.9%
Kenya -8.2% +8.6%
Madagascar -5.6% +2.5%
Malawi -11.4% +8.8%
Mauritius -27.8% +18.3%
Mozambique -13.9% +13.3%
Rwanda -14.0% +7.2%
Seychelles -14.9% +10.2%
Somalia -5.3% +3.4%
United Republic of Tanzania -13.6% +8.9%
Uganda -14.6% +11.4%
Zambia -14.5% +12.2%
Southern Sub-Saharan Africa -6.7% +7.7%
Botswana -14.2% +12.2%
Lesotho -9.4% +10.9%
Namibia -12.5% +10.7%
South Africa -6.1% +7.9%
Eswatini -12.6% +12.9%
Zimbabwe -3.1% +3.9%
Western Sub-Saharan Africa -10.7% +6.6%
Benin -11.2% +8.0%
Burkina Faso -11.7% +9.7%
Cameroon -11.4% +9.3%
Cabo Verde -8.1% +19.0%
Chad -9.4% +7.1%
Côte d'Ivoire -8.4% +4.6%
Gambia -11.2% +9.4%
Ghana -11.4% +9.9%
Guinea -8.1% +7.3%
Guinea-Bissau -11.7% +8.5%
Liberia -11.4% +6.6%
Mali -12.5% +7.0%
Mauritania -17.5% +8.5%
Niger -10.0% +5.6%
Nigeria -13.9% +8.7%
Sao Tome and Principe -10.7% +8.6%
Senegal -13.2% +8.5%
Sierra Leone -10.1% +5.1%
Togo -8.8% +7.3%
American Samoa -9.6% +5.8%
Bermuda -17.4% +3.9%
Cook Islands -13.4% +5.9%
Greenland -10.9% +3.5%
Guam -16.4% +0.8%
Monaco -17.3% +9.4%
Nauru -7.0% +6.4%
Niue -9.9% +8.0%
Northern Mariana Islands -8.5% +8.5%
Palau -8.3% +6.1%
Puerto Rico -19.5% +4.4%
Saint Kitts and Nevis -18.5% +11.3%
San Marino -9.6% +0.9%
Tokelau -12.9% +6.4%
Tuvalu -12.1% +7.3%
United States Virgin Islands -3.7% +7.0%
South Sudan -8.8% +4.6%
Sudan -9.1% +4.5%
40.0% 50.0% 60.0% 70.0% 80.0% 90.0% 100.0%
20q70

Figure S19. Decomposition of the probability of death, for all GBD locations, for the population aged 70 to 90 years old, from 1990 to 2019, in females.

Probability of death between 70 and 90 years of age (denoted using standard demographer notation as 20q70, the probability 'q' of death for a period of 20 years starting at age 70) is plotted globally and by Global Burden of Disease super-region and region for 1990 (dashed vertical line) and 2019 (solid vertical line). The relative contribution of that change due to different causes is indicated by different coloured bars. All causes to the right of the dashed line increased from 1990 to 2019 and all those to the left decreased over the same time period. As can be seen at the global level, the probability of death for those 70 to 90 years decreased mainly due to cardiovascular diseases, chronic respiratory diseases, respiratory infections and tuberculosis and enteric infections (-14.6% in aggregate), while probability of death increased owing to increases in neoplasms, neurological disorders, and diabetes and kidney diseases (+3.6% in aggregate). ***COLOR LEGEND:***

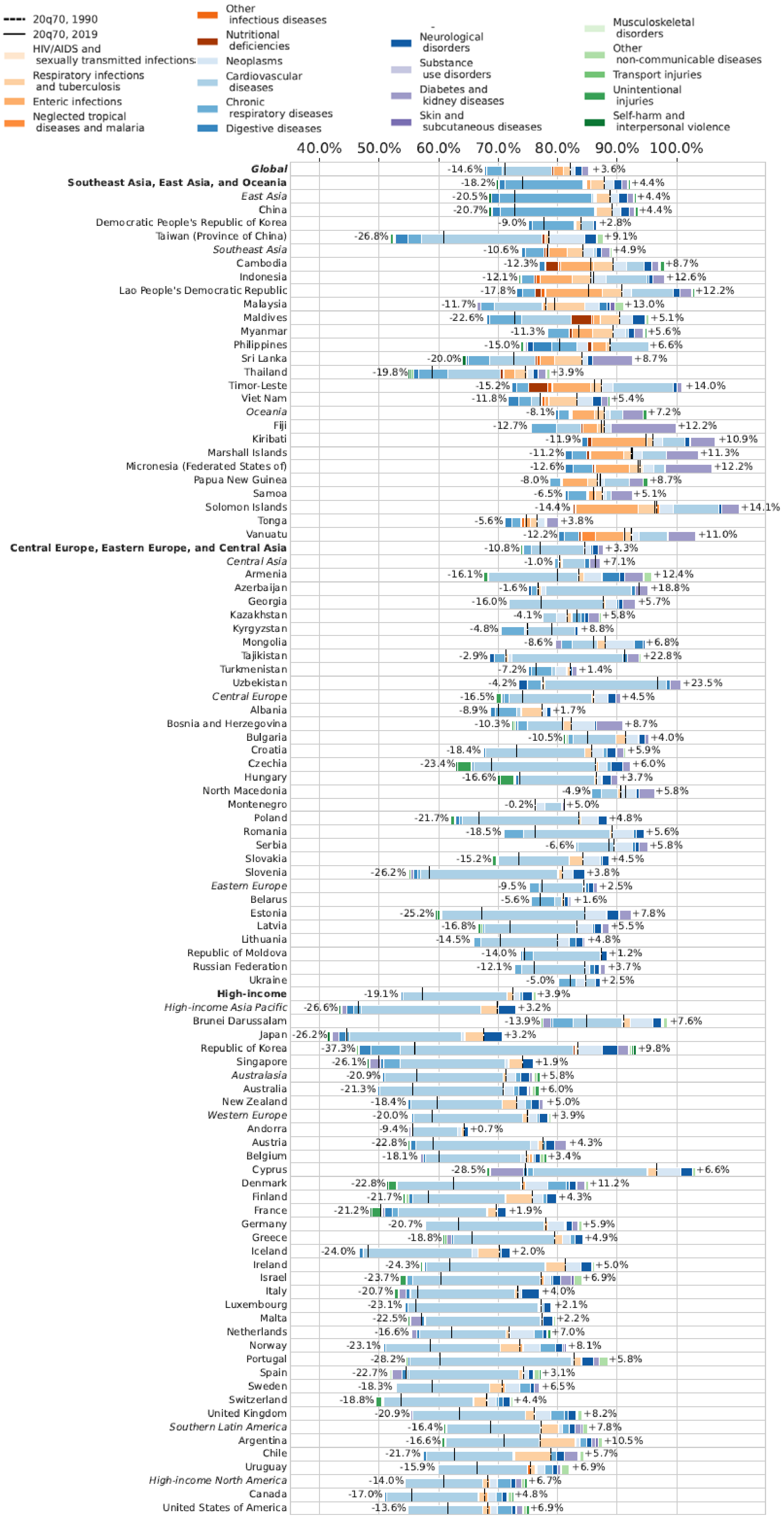

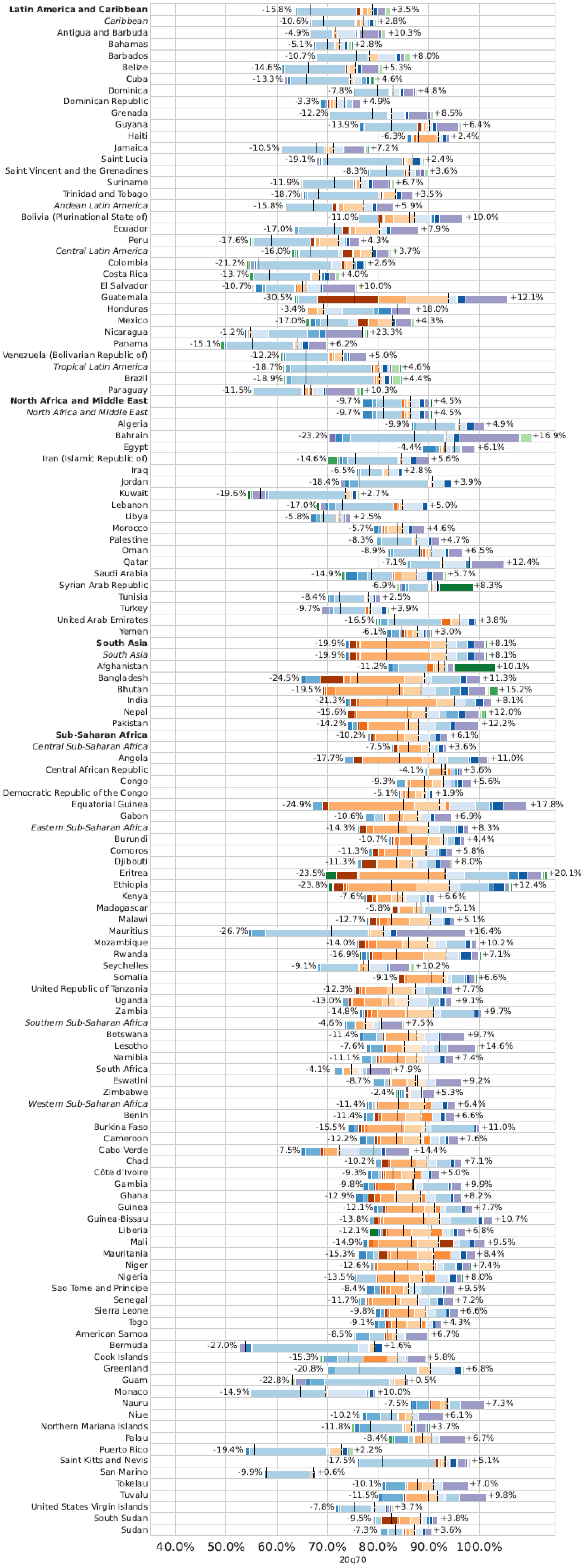
Latin America and Caribbean -15.8% +3.5%
Caribbean -10.6% +2.8%
Antigua and Barbuda -4.9% +10.3%
Bahamas -5.1% +2.8%
Barbados -10.7% +8.0%
Belize -14.6% +5.3%
Cuba -13.3% +4.6%
Dominica -7.8% +4.8%
Dominican Republic -3.3% +4.9%
Grenada -12.2% +8.5%
Guyana -13.9% +6.4%
Haiti -6.3% +2.4%
Jamaica -10.5% +7.2%
Saint Lucia -19.1% +2.4%
Saint Vincent and the Grenadines -8.3% +3.6%
Suriname -11.9% +6.7%
Trinidad and Tobago -18.7% +3.5%
Andean Latin America -15.8% +5.9%
Bolivia (Plurinational State of) -11.0% +10.0%
Ecuador -17.0% +7.9%
Peru -17.6% +4.3%
Central Latin America -16.0% +3.7%
Colombia -21.2% +2.6%
Costa Rica -13.7% +4.0%
El Salvador -10.7% +10.0%
Guatemala -30.5% +12.1%
Honduras -3.4% +18.0%
Mexico -17.0% +4.3%
Nicaragua -1.2% +23.3%
Panama -15.1% +6.2%
Venezuela (Bolivarian Republic of) -12.2% +5.0%
Tropical Latin America -18.7% +4.6%
Brazil -18.9% +4.4%
Paraguay -11.5% +10.3%
North Africa and Middle East -9.7% +4.5%
North Africa and Middle East -9.7% +4.5%
Algeria -9.9% +4.9%
Bahrain -23.2% +16.9%
Egypt -4.4% +6.1%
Iran (Islamic Republic of) -14.6% +5.6%
Iraq -6.5% +2.8%
Jordan -18.4% +3.9%
Kuwait -19.6% +2.7%
Lebanon -17.0% +5.0%
Libya -5.8% +2.5%
Morocco -5.7% +4.6%
Palestine -8.3% +4.7%
Oman -8.9% +6.5%
Qatar -7.1% +12.4%
Saudi Arabia -14.9% +5.7%
Syrian Arab Republic -6.9% +8.3%
Tunisia -8.4% +2.5%
Turkey -9.7% +3.9%
United Arab Emirates -16.5% +3.8%
Yemen -6.1% +3.0%
South Asia -19.9% +8.1%
South Asia -19.9% +8.1%
Afghanistan -11.2% +10.1%
Bangladesh -24.5% +11.3%
Bhutan -19.5% +15.2%
India -21.3% +8.1%
Nepal -15.6% +12.0%
Pakistan -14.2% +12.2%
Sub-Saharan Africa -10.2% +6.1%
Central Sub-Saharan Africa -7.5% +3.6%
Angola -17.7% +11.0%
Central African Republic -4.1% +3.6%
Congo -9.3% +5.6%
Democratic Republic of the Congo -5.1% +1.9%
Equatorial Guinea -24.9% +17.8%
Gabon -10.6% +6.9%
Eastern Sub-Saharan Africa -14.3% +8.3%
Burundi -10.7% +4.4%
Comoros -11.3% +5.8%
Djibouti -11.3% +8.0%
Eritrea -23.5% +20.1%
Ethiopia -23.8% +12.4%
Kenya -7.6% +6.6%
Madagascar -5.8% +5.1%
Malawi -12.7% +5.1%
Mauritius -26.7% +16.4%
Mozambique -14.0% +10.2%
Rwanda -16.9% +7.1%
Seychelles -9.1% +10.2%
Somalia -9.1% +6.6%
United Republic of Tanzania -12.3% +7.7%
Uganda -13.0% +9.1%
Zambia -14.8% +9.7%
Southern Sub-Saharan Africa -4.6% +7.5%
Botswana -11.4% +9.7%
Lesotho -7.6% +14.6%
Namibia -11.1% +7.4%
South Africa -4.1% +7.9%
Eswatini -8.7% +9.2%
Zimbabwe -2.4% +5.3%
Western Sub-Saharan Africa -11.4% +6.4%
Benin -11.4% +6.6%
Burkina Faso -15.5% +11.0%
Cameroon -12.2% +7.6%
Cabo Verde -7.5% +14.4%
Chad -10.2% +7.1%
Côte d'Ivoire -9.3% +5.0%
Gambia -9.8% +9.9%
Ghana -12.9% +8.2%
Guinea -12.1% +7.7%
Guinea-Bissau -13.8% +10.7%
Liberia -12.1% +6.8%
Mali -14.9% +9.5%
Mauritania -15.3% +8.4%
Niger -12.6% +7.4%
Nigeria -13.5% +8.0%
Sao Tome and Principe -8.4% +9.5%
Senegal -11.7% +7.2%
Sierra Leone -9.8% +6.6%
Togo -9.1% +4.3%
American Samoa -8.5% +6.7%
Bermuda -27.0% +1.6%
Cook Islands -15.3% +5.8%
Greenland -20.8% +6.8%
Guam -22.8% +0.5%
Monaco -14.9% +10.0%
Nauru -7.5% +7.3%
Niue -10.2% +6.1%
Northern Mariana Islands -11.8% +3.7%
Palau -8.4% +6.7%
Puerto Rico -19.4% +2.2%
Saint Kitts and Nevis -17.5% +5.1%
San Marino -9.9% +0.6%
Tokelau -10.1% +7.0%
Tuvalu -11.5% +9.8%
United States Virgin Islands -7.8% +3.7%
South Sudan -9.5% +3.8%
Sudan -7.3% +3.6%
40.0% 50.0% 60.0% 70.0% 80.0% 90.0% 100.0%
20q70

Figure S20. Leading ten causes of total deaths with ratio of observed to expected years lived with disability in 2019, among people 70 years and older, in males, for all GBD locations.

Causes are ranked based on global estimates of deaths. Causes are color coded (shown in color key) based on the ratio of observed to expected (O:E) rates on that basis of SDI from the epidemiologic transition analysis. Shades of blue represent much lower observed deaths and YLDs than expected levels based on SDI, whereas red shows that observed deaths and YLDs exceed expected levels. When the ratio exceeds 1 means that observed levels exceeded the expected levels on the basis of SDI. O:E ratios are listed in each cell.

Figure 20. Leading 10 causes of total deaths with ratio of observed to expected deaths in 2019, by location, males

	1	2	3	4	5	6	7	8	9	10
**Global**	Ischemic heart disease 0.8	Total cancers 0.88	Stroke 1.04	COPD 1.69	Lung cancer 1.04	Lower respiratory infect 1	Alzheimer's disease 1	Chronic kidney disease 0.97	Prostate cancer 0.64	Diabetes 0.87
**Central Europe, Eastern Europe, and Central Asia**	Ischemic heart disease 2.01	Total cancers 0.89	Stroke 1.85	Lung cancer 0.97	COPD 0.91	Alzheimer's disease 1.08	Colorectal cancer 1.15	Prostate cancer 0.74	Hypertensive heart disease 1.9	Lower respiratory infect 0.57
**Central Asia**	Ischemic heart disease 2.48	Stroke 1.76	Total cancers 0.85	COPD 1.31	Hypertensive heart disease 2.35	Cirrhosis 2.26	Lung cancer 0.91	Alzheimer's disease 1.08	Stomach cancer 1.42	Diabetes 0.78
Armenia	Ischemic heart disease 1.52	Total cancers 0.99	Stroke 0.73	COPD 1.3	Lung cancer 1.48	Alzheimer's disease 1.14	Cirrhosis 2.58	Diabetes 1.02	Hypertensive heart disease 1.65	Prostate cancer 0.7
Azerbaijan	Ischemic heart disease 2.81	Stroke 1.79	Total cancers 0.84	COPD 0.93	Hypertensive heart disease 2.59	Cirrhosis 2.89	Stomach cancer 1.92	Lung cancer 0.85	Alzheimer's disease 1.11	Diabetes 0.79
Georgia	Ischemic heart disease 1.56	Stroke 2.14	Total cancers 0.86	Hypertensive heart disease 6.49	Alzheimer's disease 1.1	Lung cancer 1.15	COPD 0.67	Prostate cancer 0.71	Diabetes 0.94	Cirrhosis 1.7
Kazakhstan	Ischemic heart disease 1.86	Stroke 2.27	Total cancers 0.83	COPD 3.01	Lung cancer 0.9	Cirrhosis 2.68	Alzheimer's disease 1.1	Lower respiratory infect 0.94	Stomach cancer 1.41	Colorectal cancer 0.92
Kyrgyzstan	Ischemic heart disease 2.04	Stroke 0.95	Total cancers 0.59	COPD 1.35	Alzheimer's disease 1.04	Cirrhosis 1.2	Stomach cancer 1.2	Hypertensive heart disease 0.81	Lung cancer 0.6	Prostate cancer 0.34
Mongolia	Ischemic heart disease 1.82	Total cancers 2.25	Stroke 1.81	Liver cancer 24.09	Stomach cancer 3.88	Lung cancer 2.18	Cirrhosis 3.41	Total burden related to hep 8.53	Esophageal cancer 8.89	Total burden related to hep 7.15
Tajikistan	Ischemic heart disease 3.12	Stroke 1.8	Total cancers 0.98	Hypertensive heart disease 3.28	COPD 0.82	Lower respiratory infect 0.84	Stomach cancer 2.23	Diabetes 0.88	Cirrhosis 1.41	Alzheimer's disease 1.06
Turkmenistan	Ischemic heart disease 2.3	Stroke 1.37	Total cancers 0.5	Alzheimer's disease 1.08	Cirrhosis 1.82	Hypertensive heart disease 1.27	COPD 0.42	Chronic kidney disease 0.65	Lung cancer 0.36	Prostate cancer 0.33
Uzbekistan	Ischemic heart disease 4.26	Stroke 1.88	Total cancers 0.72	Cirrhosis 2.42	Diabetes 1.1	Hypertensive heart disease 1.8	COPD 0.65	Lower respiratory infect 0.79	Chronic kidney disease 0.85	Lung cancer 0.61
**Central Europe**	Ischemic heart disease 1.39	Total cancers 1.03	Stroke 1.62	Lung cancer 1.15	COPD 0.87	Colorectal cancer 1.4	Alzheimer's disease 1.11	Prostate cancer 0.89	Hypertensive heart disease 3.01	Lower respiratory infect 0.76
Albania	Ischemic heart disease 1.03	Stroke 1.74	Total cancers 0.9	Lung cancer 1.31	COPD 0.7	Alzheimer's disease 1.1	Prostate cancer 0.86	Lower respiratory infect 0.55	Stomach cancer 0.98	Other cardiovascular 2.93
Bosnia and Herzegovina	Ischemic heart disease 1.09	Total cancers 1.17	Stroke 1.75	Lung cancer 1.59	Diabetes 2.01	COPD 0.95	Colorectal cancer 1.65	Prostate cancer 0.85	Alzheimer's disease 1.04	Cardiomyopathy 3.6
Bulgaria	Ischemic heart disease 1.83	Stroke 2.97	Total cancers 0.91	Hypertensive heart disease 9.43	Lung cancer 0.87	COPD 0.87	Colorectal cancer 1.47	Prostate cancer 0.89	Alzheimer's disease 1.08	Other cardiovascular 4.89
Croatia	Total cancers 1.14	Ischemic heart disease 1.23	Stroke 1.46	Lung cancer 1.1	COPD 1.07	Colorectal cancer 1.68	Prostate cancer 1.07	Alzheimer's disease 1.11	Hypertensive heart disease 2.46	Falls 2.13
Czechia	Ischemic heart disease 1.5	Total cancers 0.95	Stroke 1.03	Lung cancer 0.91	Colorectal cancer 1.21	COPD 0.82	Prostate cancer 0.88	Alzheimer's disease 1.12	Lower respiratory infect 0.93	Diabetes 1.54
Hungary	Ischemic heart disease 1.52	Total cancers 1.09	Stroke 1.09	Lung cancer 1.21	COPD 1.18	Colorectal cancer 1.72	Hypertensive heart disease 3.86	Alzheimer's disease 1.08	Prostate cancer 0.86	Diabetes 0.92
Montenegro	Stroke 3.45	Ischemic heart disease 1.51	Total cancers 1.08	Lung cancer 1.62	Prostate cancer 1.13	Cardiomyopathy 6.43	Alzheimer's disease 1.16	Atrial fibrillation 3.87	Colorectal cancer 0.99	Chronic kidney disease 1.24
North Macedonia	Stroke 3.81	Ischemic heart disease 1.47	Total cancers 1.14	Lung cancer 1.25	Hypertensive heart disease 4.6	Diabetes 2	COPD 1	Prostate cancer 1	Colorectal cancer 1.33	Cardiomyopathy 4.61
Poland	Total cancers 1.16	Ischemic heart disease 1.25	Stroke 1	Lung cancer 1.46	Colorectal cancer 1.45	Alzheimer's disease 1.19	Prostate cancer 0.98	COPD 0.79	Lower respiratory infect 0.98	Cardiomyopathy 3.57
Romania	Ischemic heart disease 1.4	Stroke 2.01	Total cancers 0.83	Hypertensive heart disease 4.1	COPD 0.86	Lung cancer 0.85	Alzheimer's disease 1.05	Cardiomyopathy 4.95	Colorectal cancer 1.21	Prostate cancer 0.67
Serbia	Ischemic heart disease 1.45	Stroke 2.51	Total cancers 1.05	Lung cancer 1.18	COPD 1.02	Colorectal cancer 1.52	Prostate cancer 0.92	Diabetes 1.38	Alzheimer's disease 1.02	Chronic kidney disease 1.41
Slovakia	Ischemic heart disease 1.83	Total cancers 1	Stroke 1.25	Lung cancer 0.94	Colorectal cancer 1.58	Lower respiratory infect 1.28	Alzheimer's disease 1.09	Prostate cancer 0.86	COPD 0.64	Hypertensive heart disease 1.92
Slovenia	Total cancers 1.02	Ischemic heart disease 0.67	Stroke 0.96	Prostate cancer 1.21	Lung cancer 0.86	Alzheimer's disease 1.22	Colorectal cancer 1.14	COPD 0.76	Lower respiratory infect 0.99	Hypertensive heart disease 3.02
**Eastern Europe**	Ischemic heart disease 2.43	Stroke 2.07	Total cancers 0.78	Lung cancer 0.83	COPD 0.87	Alzheimer's disease 1.05	Colorectal cancer 0.97	Prostate cancer 0.66	Stomach cancer 1.27	Lower respiratory infect 0.41
Belarus	Ischemic heart disease 2.81	Total cancers 0.86	Stroke 1.44	Lung cancer 0.96	COPD 0.78	Alzheimer's disease 1.04	Prostate cancer 0.74	Colorectal cancer 1.15	Stomach cancer 1.33	Alcohol use disorders 13.18
Estonia	Ischemic heart disease 1.54	Total cancers 1	Hypertensive heart disease 10.79	Stroke 0.81	Lung cancer 1.01	Prostate cancer 1.17	Alzheimer's disease 1.16	Colorectal cancer 1	COPD 0.53	Chronic kidney disease 1.08
Latvia	Ischemic heart disease 1.96	Total cancers 0.96	Stroke 1.96	Lung cancer 0.92	Prostate cancer 1.12	Alzheimer's disease 1.07	Colorectal cancer 0.98	COPD 0.46	Hypertensive heart disease 2.09	Stomach cancer 1.25
Lithuania	Ischemic heart disease 2.29	Total cancers 0.89	Stroke 1.47	Lung cancer 0.8	Prostate cancer 1.01	Alzheimer's disease 1.09	COPD 0.71	Colorectal cancer 0.9	Stomach cancer 1.36	Bladder cancer 1.12
Republic of Moldova	Ischemic heart disease 1.83	Stroke 1.06	Total cancers 0.56	Hypertensive heart disease 2.51	Alzheimer's disease 1.01	COPD 0.69	Cirrhosis 1.84	Colorectal cancer 1.03	Prostate cancer 0.47	Lung cancer 0.51
Russian Federation	Ischemic heart disease 2.07	Stroke 2.42	Total cancers 0.78	Lung cancer 0.84	COPD 0.95	Alzheimer's disease 1.05	Colorectal cancer 0.93	Prostate cancer 0.63	Stomach cancer 1.39	Lower respiratory infect 0.47
Ukraine	Ischemic heart disease 3.33	Stroke 1.59	Total cancers 0.74	COPD 0.73	Alzheimer's disease 1.07	Lung cancer 0.76	Colorectal cancer 1.1	Prostate cancer 0.64	Stomach cancer 1.01	Parkinson's disease 0.92
**High-income**	Total cancers 0.85	Ischemic heart disease 0.64	Stroke 0.67	Lung cancer 0.87	COPD 1	Alzheimer's disease 1.05	Lower respiratory infect 1.06	Prostate cancer 0.76	Colorectal cancer 0.75	Chronic kidney disease 1.28
**Australasia**	Total cancers 0.82	Ischemic heart disease 0.63	Stroke 0.56	COPD 0.97	Prostate cancer 0.93	Lung cancer 0.69	Alzheimer's disease 1	Colorectal cancer 0.73	Chronic kidney disease 1.15	Lower respiratory infect 0.48
Australia	Total cancers 0.82	Ischemic heart disease 0.6	Stroke 0.54	COPD 0.94	Lung cancer 0.7	Prostate cancer 0.92	Alzheimer's disease 1	Colorectal cancer 0.7	Chronic kidney disease 1.17	Diabetes 1.01
New Zealand	Total cancers 0.82	Ischemic heart disease 0.79	Stroke 0.62	COPD 1.11	Prostate cancer 0.96	Alzheimer's disease 1.01	Lung cancer 0.63	Colorectal cancer 0.93	Chronic kidney disease 1.07	Lower respiratory infect 0.53
**High-income Asia Pacific**	Total cancers 0.82	Lower respiratory infect 1.81	Stroke 0.78	Ischemic heart disease 0.33	Lung cancer 0.86	Alzheimer's disease 1.32	Stomach cancer 2.1	COPD 0.58	Colorectal cancer 0.68	Chronic kidney disease 1.09
Brunei Darussalam	Total cancers 1.36	Ischemic heart disease 1.45	Diabetes 7.99	Stroke 1.85	COPD 2.6	Lower respiratory infect 4.18	Lung cancer 1.45	Chronic kidney disease 4.49	Colorectal cancer 1.68	Prostate cancer 1.01
Japan	Total cancers 0.81	Lower respiratory infect 1.82	Ischemic heart disease 0.33	Stroke 0.73	Alzheimer's disease 1.35	Lung cancer 0.83	Stomach cancer 2.14	Colorectal cancer 0.69	COPD 0.53	Chronic kidney disease 1.07
Republic of Korea	Total cancers 0.88	Stroke 1.15	Lung cancer 1.03	Ischemic heart disease 0.36	Lower respiratory infect 1.53	COPD 0.91	Alzheimer's disease 1.15	Stomach cancer 2.04	Liver cancer 3.94	Diabetes 1.84
Singapore	Total cancers 0.6	Lower respiratory infect 3.33	Ischemic heart disease 0.55	Lung cancer 0.71	Stroke 0.47	Alzheimer's disease 1.05	COPD 0.55	Colorectal cancer 0.65	Liver cancer 2.51	Chronic kidney disease 0.91
**High-income North America**	Total cancers 0.8	Ischemic heart disease 0.9	COPD 1.3	Lung cancer 0.91	Stroke 0.59	Alzheimer's disease 0.96	Prostate cancer 0.78	Chronic kidney disease 1.66	Lower respiratory infect 0.68	Colorectal cancer 0.57
Canada	Total cancers 0.79	Ischemic heart disease 0.71	Lung cancer 0.84	COPD 1	Stroke 0.53	Prostate cancer 0.84	Alzheimer's disease 0.95	Colorectal cancer 0.67	Lower respiratory infect 0.65	Chronic kidney disease 1.11
Greenland	Total cancers 1.4	Ischemic heart disease 0.74	Stroke 1.22	Lung cancer 2.26	COPD 1.51	Lower respiratory infect 1.35	Colorectal cancer 1.5	Alzheimer's disease 0.97	Falls 3.2	Chronic kidney disease 1.2
United States of America	Total cancers 0.8	Ischemic heart disease 0.93	COPD 1.33	Lung cancer 0.91	Stroke 0.6	Alzheimer's disease 0.96	Prostate cancer 0.77	Chronic kidney disease 1.73	Lower respiratory infect 0.69	Colorectal cancer 0.56
**Southern Latin America**	Total cancers 1.08	Ischemic heart disease 0.51	Lower respiratory infect 2.5	Stroke 0.6	COPD 1.15	Prostate cancer 1.25	Chronic kidney disease 1.84	Lung cancer 0.92	Alzheimer's disease 0.92	Colorectal cancer 1.38
Argentina	Total cancers 1.12	Ischemic heart disease 0.56	Lower respiratory infect 3.32	Stroke 0.57	COPD 1.25	Chronic kidney disease 2.03	Prostate cancer 1.22	Lung cancer 1.04	Colorectal cancer 1.64	Alzheimer's disease 0.91
Chile	Total cancers 0.94	Ischemic heart disease 0.39	Stroke 0.67	Prostate cancer 1.25	COPD 0.83	Lower respiratory infect 1.03	Chronic kidney disease 1.56	Alzheimer's disease 0.94	Stomach cancer 2.07	Lung cancer 0.58
Uruguay	Total cancers 1.41	Ischemic heart disease 0.48	Stroke 0.63	COPD 1.49	Prostate cancer 1.52	Lung cancer 1.44	Lower respiratory infect 1.21	Colorectal cancer 1.9	Alzheimer's disease 0.9	Chronic kidney disease 1.22
**Western Europe**	Total cancers 0.9	Ischemic heart disease 0.65	Stroke 0.68	Lung cancer 0.85	COPD 1.01	Prostate cancer 0.91	Alzheimer's disease 0.98	Lower respiratory infect 0.84	Colorectal cancer 0.87	Chronic kidney disease 1.1
Andorra	Total cancers 1.07	Ischemic heart disease 0.53	Lung cancer 0.96	COPD 1.35	Colorectal cancer 1.26	Stroke 0.61	Prostate cancer 0.92	Alzheimer's disease 0.98	Lower respiratory infect 0.66	Chronic kidney disease 1.12
Austria	Total cancers 0.76	Ischemic heart disease 0.94	Stroke 0.56	Prostate cancer 0.86	Lung cancer 0.61	COPD 0.75	Alzheimer's disease 0.9	Chronic kidney disease 1.58	Colorectal cancer 0.7	Diabetes 1.28
Belgium	Total cancers 0.95	Ischemic heart disease 0.62	Lung cancer 1.11	COPD 1.33	Stroke 0.72	Lower respiratory infect 1.36	Prostate cancer 0.98	Alzheimer's disease 0.94	Colorectal cancer 0.85	Chronic kidney disease 1.11
Cyprus	Total cancers 0.8	Ischemic heart disease 0.88	Stroke 0.82	COPD 1.3	Lung cancer 0.81	Diabetes 2.83	Prostate cancer 0.99	Chronic kidney disease 2.11	Alzheimer's disease 0.96	Colorectal cancer 0.71
Denmark	Total cancers 0.93	Ischemic heart disease 0.69	COPD 1.53	Stroke 0.92	Lung cancer 0.82	Prostate cancer 1.3	Lower respiratory infect 1.19	Colorectal cancer 0.91	Alzheimer's disease 0.92	Diabetes 1.48
Finland	Ischemic heart disease 1.18	Total cancers 0.74	Stroke 0.82	Prostate cancer 0.95	Lung cancer 0.66	Alzheimer's disease 0.9	COPD 0.7	Colorectal cancer 0.57	Falls 1.48	Pancreatic cancer 1.05
France	Total cancers 0.92	Ischemic heart disease 0.43	Stroke 0.53	Lung cancer 0.84	Prostate cancer 0.94	Alzheimer's disease 0.99	Colorectal cancer 0.88	Lower respiratory infect 0.76	COPD 0.56	Falls 1.49
Germany	Total cancers 0.81	Ischemic heart disease 1	Stroke 0.79	Lung cancer 0.68	COPD 0.9	Prostate cancer 0.89	Alzheimer's disease 0.92	Colorectal cancer 0.74	Chronic kidney disease 1.76	Lower respiratory infect 0.72
Greece	Total cancers 0.99	Ischemic heart disease 0.68	Stroke 1.04	Lung cancer 1.37	COPD 0.89	Alzheimer's disease 0.92	Lower respiratory infect 0.96	Prostate cancer 0.88	Chronic kidney disease 1.3	Colorectal cancer 0.86
Iceland	Total cancers 0.73	Ischemic heart disease 0.9	Stroke 0.6	Prostate cancer 0.99	Alzheimer's disease 0.95	Lung cancer 0.57	Lower respiratory infect 0.88	COPD 0.67	Colorectal cancer 0.57	Parkinson's disease 1.24
Ireland	Total cancers 0.85	Ischemic heart disease 0.87	COPD 1.38	Stroke 0.69	Lung cancer 0.73	Prostate cancer 1	Lower respiratory infect 1.23	Colorectal cancer 0.87	Alzheimer's disease 0.95	Chronic kidney disease 1.15
Israel	Total cancers 0.79	Ischemic heart disease 0.47	Chronic kidney disease 2.18	Stroke 0.47	Alzheimer's disease 0.9	Diabetes 1.79	Lung cancer 0.68	COPD 0.59	Lower respiratory infect 0.74	Colorectal cancer 0.89
Italy	Total cancers 0.94	Ischemic heart disease 0.55	Stroke 0.69	Lung cancer 1.03	Alzheimer's disease 1.15	COPD 0.9	Prostate cancer 0.71	Colorectal cancer 0.92	Hypertensive heart disease 2.09	Diabetes 1.1
Luxembourg	Total cancers 0.76	Ischemic heart disease 0.7	Stroke 0.83	Lung cancer 0.78	COPD 0.95	Prostate cancer 0.81	Alzheimer's disease 0.9	Colorectal cancer 0.69	Lower respiratory infect 0.73	Chronic kidney disease 1.22
Malta	Ischemic heart disease 0.87	Total cancers 0.73	Stroke 0.62	Lung cancer 0.86	COPD 0.83	Lower respiratory infect 1.11	Alzheimer's disease 0.9	Colorectal cancer 0.82	Prostate cancer 0.56	Diabetes 1.07
Monaco	Total cancers 1.29	Ischemic heart disease 0.8	Lung cancer 1.46	Stroke 0.84	Lower respiratory infect 1.2	Prostate cancer 1.01	Colorectal cancer 1.04	Alzheimer's disease 0.95	COPD 0.76	Bladder cancer 1.7
Netherlands	Total cancers 1.02	Ischemic heart disease 0.57	Lung cancer 1.03	COPD 1.42	Stroke 0.77	Prostate cancer 1.11	Colorectal cancer 0.91	Alzheimer's disease 1.07	Lower respiratory infect 1.05	Falls 1.38
Norway	Total cancers 0.77	Ischemic heart disease 0.72	Stroke 0.81	Prostate cancer 1.27	COPD 1.14	Lower respiratory infect 1.11	Lung cancer 0.54	Alzheimer's disease 0.97	Colorectal cancer 0.75	Falls 1.4
Portugal	Total cancers 0.99	Stroke 0.9	Ischemic heart disease 0.35	Lower respiratory infect 1.75	COPD 1.07	Prostate cancer 1.02	Colorectal cancer 1.47	Alzheimer's disease 0.88	Lung cancer 0.78	Chronic kidney disease 1.18
San Marino	Total cancers 1.18	Ischemic heart disease 0.54	Stroke 0.9	Lung cancer 0.91	Prostate cancer 1.15	Colorectal cancer 1.02	Alzheimer's disease 1	Stomach cancer 2.65	COPD 0.56	Lower respiratory infect 0.67
Spain	Total cancers 0.98	Ischemic heart disease 0.39	COPD 1.56	Stroke 0.49	Lung cancer 1.08	Colorectal cancer 1.32	Alzheimer's disease 0.86	Prostate cancer 0.78	Lower respiratory infect 0.68	Chronic kidney disease 1.05
Sweden	Total cancers 0.74	Ischemic heart disease 0.88	Stroke 0.78	Prostate cancer 1.16	Alzheimer's disease 0.91	COPD 0.65	Lung cancer 0.45	Colorectal cancer 0.67	Lower respiratory infect 0.67	Chronic kidney disease 0.99
Switzerland	Total cancers 0.66	Ischemic heart disease 0.78	Stroke 0.64	Prostate cancer 0.94	Alzheimer's disease 0.99	Lung cancer 0.49	COPD 0.72	Colorectal cancer 0.46	Chronic kidney disease 1.28	Falls 1.15
United Kingdom	Total cancers 0.96	Ischemic heart disease 0.71	COPD 1.36	Stroke 0.7	Lower respiratory infect 1.54	Lung cancer 0.9	Prostate cancer 1.1	Alzheimer's disease 1	Colorectal cancer 0.86	Parkinson's disease 1.08

**Latin America and Caribbean**	Total cancers<br>0.81	Ischemic heart disease<br>0.49	Stroke<br>0.45	COPD<br>0.95	Lower respiratory infect<br>1.21	Chronic kidney disease<br>1.37	Alzheimer's disease<br>1.09	Prostate cancer<br>1.09	Diabetes<br>1.18	Lung cancer<br>0.65
**Andean Latin America**	Total cancers<br>0.82	Ischemic heart disease<br>0.37	Lower respiratory infect<br>1.99	Stroke<br>0.31	Chronic kidney disease<br>1.49	Prostate cancer<br>1.1	Alzheimer's disease<br>0.98	COPD<br>0.54	Stomach cancer<br>1.58	Cirrhosis<br>1.56
Bolivia (Plurinational State of)	Total cancers<br>1.33	Ischemic heart disease<br>0.62	Lower respiratory infect<br>2.15	Stroke<br>0.54	Chronic kidney disease<br>2.15	Prostate cancer<br>1.79	COPD<br>0.96	Stomach cancer<br>2.75	Diabetes<br>1.16	Cirrhosis<br>1.94
Ecuador	Total cancers<br>0.87	Ischemic heart disease<br>0.44	Chronic kidney disease<br>2.17	Stroke<br>0.36	Lower respiratory infect<br>1.49	Prostate cancer<br>1.18	COPD<br>0.81	Diabetes<br>1.1	Alzheimer's disease<br>1.03	Stomach cancer<br>1.73
Peru	Total cancers<br>0.7	Ischemic heart disease<br>0.3	Lower respiratory infect<br>2.15	Alzheimer's disease<br>0.94	Stroke<br>0.24	Prostate cancer<br>0.94	Chronic kidney disease<br>1.06	Stomach cancer<br>1.26	Cirrhosis<br>1.37	Interstitial lung disease<br>12.57
**Caribbean**	Total cancers<br>1.01	Ischemic heart disease<br>0.7	Stroke<br>0.67	Prostate cancer<br>1.8	Lower respiratory infect<br>1.15	COPD<br>0.71	Alzheimer's disease<br>0.97	Diabetes<br>1.21	Lung cancer<br>0.91	Chronic kidney disease<br>1
Antigua and Barbuda	Total cancers<br>1.15	Prostate cancer<br>3.49	Ischemic heart disease<br>0.6	Stroke<br>0.87	Diabetes<br>3.06	Lower respiratory infect<br>1.68	Chronic kidney disease<br>2.44	Hypertensive heart disease<br>2.86	Alzheimer's disease<br>1.03	Stomach cancer<br>1.12
Bahamas	Total cancers<br>1.06	Ischemic heart disease<br>0.69	Prostate cancer<br>3.04	Stroke<br>0.91	Hypertensive heart disease<br>7.15	Diabetes<br>2.34	Lower respiratory infect<br>1.36	Chronic kidney disease<br>1.92	Alzheimer's disease<br>1.06	Colorectal cancer<br>0.85
Barbados	Total cancers<br>1.29	Prostate cancer<br>3.42	Ischemic heart disease<br>0.46	Stroke<br>0.79	Diabetes<br>3.23	Lower respiratory infect<br>1.66	Alzheimer's disease<br>1	Chronic kidney disease<br>1.46	Colorectal cancer<br>1.27	Urinary diseases<br>5.59
Belize	Total cancers<br>0.85	Ischemic heart disease<br>0.45	Stroke<br>0.46	Prostate cancer<br>1.69	Lower respiratory infect<br>1.47	Diabetes<br>1.45	Chronic kidney disease<br>1.32	COPD<br>0.7	Alzheimer's disease<br>0.99	Hypertensive heart disease<br>1.07
Bermuda	Total cancers<br>1.09	Ischemic heart disease<br>0.89	Prostate cancer<br>2.07	Stroke<br>0.72	Lung cancer<br>0.84	Alzheimer's disease<br>1.08	Colorectal cancer<br>1.01	Diabetes<br>1.63	Chronic kidney disease<br>1.32	Lower respiratory infect<br>0.74
Cuba	Total cancers<br>1.13	Ischemic heart disease<br>0.64	Stroke<br>0.54	Lower respiratory infect<br>1.72	Prostate cancer<br>1.57	Lung cancer<br>1.44	Alzheimer's disease<br>0.97	COPD<br>0.74	Falls<br>3.64	Colorectal cancer<br>1.11
Dominica	Total cancers<br>1.79	Prostate cancer<br>5.26	Ischemic heart disease<br>0.53	Stroke<br>0.85	Diabetes<br>2.74	Hypertensive heart disease<br>4.34	Chronic kidney disease<br>2.57	Lower respiratory infect<br>1.65	COPD<br>0.85	Alzheimer's disease<br>1.01
Dominican Republic	Ischemic heart disease<br>0.9	Total cancers<br>1.01	Stroke<br>0.73	Prostate cancer<br>1.9	Cirrhosis<br>1.77	Alzheimer's disease<br>0.89	Hypertensive heart disease<br>1.29	Diabetes<br>0.83	COPD<br>0.46	Lower respiratory infect<br>0.63
Grenada	Total cancers<br>1.52	Ischemic heart disease<br>0.65	Prostate cancer<br>4.1	Stroke<br>0.86	Diabetes<br>2.64	Chronic kidney disease<br>2.44	Lower respiratory infect<br>2.13	Hypertensive heart disease<br>1.71	COPD<br>0.63	Colorectal cancer<br>1.25
Guyana	Ischemic heart disease<br>0.9	Stroke<br>1.22	Total cancers<br>1.01	Prostate cancer<br>2.7	Diabetes<br>2.51	Hypertensive heart disease<br>2.92	Lower respiratory infect<br>1.78	Chronic kidney disease<br>1.6	COPD<br>0.58	Alzheimer's disease<br>0.96
Haiti	Ischemic heart disease<br>1.1	Total cancers<br>1.36	Stroke<br>1.12	Prostate cancer<br>2.88	COPD<br>0.94	Lower respiratory infect<br>0.98	Diabetes<br>1.09	Hypertensive heart disease<br>1.51	Chronic kidney disease<br>1.05	Alzheimer's disease<br>1
Jamaica	Total cancers<br>1.09	Stroke<br>0.89	Ischemic heart disease<br>0.38	Prostate cancer<br>2.51	Diabetes<br>3.07	COPD<br>0.81	Alzheimer's disease<br>0.96	Chronic kidney disease<br>1.5	Hypertensive heart disease<br>1.87	Lung cancer<br>0.79
Puerto Rico	Total cancers<br>0.62	Ischemic heart disease<br>0.5	Diabetes<br>3.19	Prostate cancer<br>1.05	Alzheimer's disease<br>1.04	Chronic kidney disease<br>1.97	Stroke<br>0.41	COPD<br>0.78	Lower respiratory infect<br>0.7	Colorectal cancer<br>0.66
Saint Kitts and Nevis	Total cancers<br>1.34	Stroke<br>1.6	Ischemic heart disease<br>0.78	Prostate cancer<br>4.05	Diabetes<br>3.1	Chronic kidney disease<br>3.42	Lower respiratory infect<br>2.1	Hypertensive heart disease<br>3.21	COPD<br>0.7	Colorectal cancer<br>1.07
Saint Lucia	Total cancers<br>1.31	Prostate cancer<br>3.28	Stroke<br>0.85	Ischemic heart disease<br>0.4	Diabetes<br>2.41	COPD<br>1.06	Chronic kidney disease<br>1.68	Lower respiratory infect<br>1.29	Alzheimer's disease<br>1.02	Hypertensive heart disease<br>1.62
Saint Vincent and the Grenadines	Total cancers<br>1.33	Ischemic heart disease<br>0.65	Prostate cancer<br>3.42	Stroke<br>0.7	Diabetes<br>2.41	Lower respiratory infect<br>1.32	Hypertensive heart disease<br>1.91	Chronic kidney disease<br>1.36	Alzheimer's disease<br>0.93	COPD<br>0.47
Suriname	Total cancers<br>0.94	Ischemic heart disease<br>0.53	Stroke<br>0.82	Prostate cancer<br>1.82	Chronic kidney disease<br>1.86	Diabetes<br>1.43	Lower respiratory infect<br>1.28	Alzheimer's disease<br>0.98	COPD<br>0.55	Hypertensive heart disease<br>1.2
Trinidad and Tobago	Ischemic heart disease<br>0.81	Total cancers<br>0.78	Diabetes<br>5.65	Stroke<br>0.95	Prostate cancer<br>2.22	Chronic kidney disease<br>2.17	Alzheimer's disease<br>0.99	Lower respiratory infect<br>0.72	Hypertensive heart disease<br>1.92	COPD<br>0.5
United States Virgin Islands	Total cancers<br>1.38	Ischemic heart disease<br>1.56	Prostate cancer<br>3.7	Stroke<br>1.07	Diabetes<br>3.16	Chronic kidney disease<br>2.49	Hypertensive heart disease<br>4.47	Colorectal cancer<br>1.34	Lung cancer<br>0.69	Lower respiratory infect<br>1.01
**Central Latin America**	Ischemic heart disease<br>0.56	Total cancers<br>0.71	Chronic kidney disease<br>1.85	COPD<br>0.98	Stroke<br>0.35	Diabetes<br>1.46	Alzheimer's disease<br>1.08	Prostate cancer<br>0.94	Lower respiratory infect<br>0.72	Cirrhosis<br>1.5
Colombia	Ischemic heart disease<br>0.44	Total cancers<br>0.66	COPD<br>1.02	Alzheimer's disease<br>1.02	Stroke<br>0.27	Prostate cancer<br>0.79	Chronic kidney disease<br>0.9	Lower respiratory infect<br>0.45	Stomach cancer<br>0.98	Hypertensive heart disease<br>0.69
Costa Rica	Total cancers<br>0.91	Ischemic heart disease<br>0.46	COPD<br>0.95	Alzheimer's disease<br>1.12	Stroke<br>0.32	Prostate cancer<br>1.11	Chronic kidney disease<br>1.41	Stomach cancer<br>1.76	Lower respiratory infect<br>0.58	Colorectal cancer<br>0.98
El Salvador	Ischemic heart disease<br>0.61	Total cancers<br>0.74	Chronic kidney disease<br>2.49	Lower respiratory infect<br>1.18	Stroke<br>0.31	Alzheimer's disease<br>1.07	Prostate cancer<br>0.97	COPD<br>0.51	Diabetes<br>0.9	Stomach cancer<br>1.26
Guatemala	Ischemic heart disease<br>0.61	Total cancers<br>0.92	Lower respiratory infect<br>2.15	Chronic kidney disease<br>1.91	Stroke<br>0.42	Diabetes<br>1.36	Prostate cancer<br>1.4	COPD<br>0.49	Alzheimer's disease<br>1.1	Cirrhosis<br>1.39
Honduras	Ischemic heart disease<br>1.08	Total cancers<br>1.03	Stroke<br>0.91	COPD<br>1.31	Chronic kidney disease<br>1.76	Cirrhosis<br>1.7	Alzheimer's disease<br>1.28	Prostate cancer<br>1.11	Lower respiratory infect<br>0.56	Liver cancer<br>3.89
Mexico	Ischemic heart disease<br>0.55	Total cancers<br>0.63	Diabetes<br>2.16	Chronic kidney disease<br>2.38	COPD<br>1.13	Stroke<br>0.31	Alzheimer's disease<br>1.12	Cirrhosis<br>2.22	Prostate cancer<br>0.81	Lower respiratory infect<br>0.7
Nicaragua	Ischemic heart disease<br>1.13	Total cancers<br>1.07	Chronic kidney disease<br>3.52	Stroke<br>0.56	COPD<br>1	Diabetes<br>1.53	Prostate cancer<br>1.62	Cirrhosis<br>1.83	Alzheimer's disease<br>1.12	Lower respiratory infect<br>0.58
Panama	Total cancers<br>0.69	Ischemic heart disease<br>0.37	Stroke<br>0.51	Alzheimer's disease<br>1.08	Prostate cancer<br>1.09	Chronic kidney disease<br>1.51	Diabetes<br>1.16	COPD<br>0.61	Lower respiratory infect<br>0.72	Lung cancer<br>0.48
Venezuela (Bolivarian Republic of)	Ischemic heart disease<br>0.74	Total cancers<br>0.95	Stroke<br>0.51	Prostate cancer<br>1.51	Chronic kidney disease<br>1.5	Diabetes<br>1.28	COPD<br>0.72	Alzheimer's disease<br>1.06	Hypertensive heart disease<br>1.41	Lower respiratory infect<br>0.65
**Tropical Latin America**	Total cancers<br>0.85	Ischemic heart disease<br>0.39	Stroke<br>0.56	COPD<br>1.09	Lower respiratory infect<br>1.55	Alzheimer's disease<br>1.15	Prostate cancer<br>1.06	Diabetes<br>1.01	Chronic kidney disease<br>0.9	Lung cancer<br>0.71
Brazil	Total cancers<br>0.85	Ischemic heart disease<br>0.39	Stroke<br>0.56	COPD<br>1.1	Lower respiratory infect<br>1.56	Alzheimer's disease<br>1.16	Prostate cancer<br>1.06	Diabetes<br>1	Chronic kidney disease<br>0.88	Lung cancer<br>0.71
Paraguay	Total cancers<br>0.85	Ischemic heart disease<br>0.51	Stroke<br>0.6	Diabetes<br>1.47	Chronic kidney disease<br>1.51	COPD<br>0.82	Alzheimer's disease<br>1.1	Lower respiratory infect<br>1.1	Prostate cancer<br>1.08	Lung cancer<br>0.89
**North Africa and Middle East**	Ischemic heart disease<br>1.2	Total cancers<br>0.67	Stroke<br>0.82	COPD<br>0.87	Hypertensive heart disease<br>2.23	Alzheimer's disease<br>1.14	Chronic kidney disease<br>1.39	Cirrhosis<br>2.18	Lower respiratory infect<br>0.78	Diabetes<br>0.9
**North Africa and Middle East**	Ischemic heart disease<br>1.2	Total cancers<br>0.67	Stroke<br>0.82	COPD<br>0.87	Hypertensive heart disease<br>2.23	Alzheimer's disease<br>1.14	Chronic kidney disease<br>1.39	Cirrhosis<br>2.18	Lower respiratory infect<br>0.78	Diabetes<br>0.9
Afghanistan	Ischemic heart disease<br>1.79	Stroke<br>1.06	Total cancers<br>1.08	Conflict & terror<br>1658.3	Hypertensive heart disease<br>2.81	Lower respiratory infect<br>0.66	Chronic kidney disease<br>1.48	COPD<br>0.72	Alzheimer's disease<br>1.39	Stomach cancer<br>2.02
Algeria	Ischemic heart disease<br>1.1	Stroke<br>0.82	Total cancers<br>0.5	Hypertensive heart disease<br>2.11	Alzheimer's disease<br>1.19	Chronic kidney disease<br>1.24	COPD<br>0.64	Lower respiratory infect<br>0.78	Diabetes<br>0.57	Lung cancer<br>0.49
Bahrain	Ischemic heart disease<br>1.06	Diabetes<br>8.21	Total cancers<br>0.81	Stroke<br>0.67	COPD<br>1.14	Chronic kidney disease<br>2.47	Lung cancer<br>0.98	Alzheimer's disease<br>1.28	Lower respiratory infect<br>1.06	Prostate cancer<br>0.69
Egypt	Ischemic heart disease<br>1.49	Cirrhosis<br>9.03	Total cancers<br>0.48	Stroke<br>0.58	Total burden related to hep<br>15.47	Hypertensive heart disease<br>2.77	Chronic kidney disease<br>1.58	Total burden related to hep<br>9.84	COPD<br>0.76	Alzheimer's disease<br>1.05
Iran (Islamic Republic of)	Ischemic heart disease<br>0.88	Total cancers<br>0.7	Stroke<br>0.64	Alzheimer's disease<br>1.13	Hypertensive heart disease<br>2.15	COPD<br>0.68	Chronic kidney disease<br>0.97	Diabetes<br>0.82	Stomach cancer<br>1.44	Lower respiratory infect<br>0.66
Iraq	Ischemic heart disease<br>1.47	Stroke<br>1.44	Total cancers<br>0.74	Chronic kidney disease<br>2.4	Diabetes<br>1.78	Alzheimer's disease<br>1.16	Lung cancer<br>0.93	Hypertensive heart disease<br>1.34	COPD<br>0.4	Prostate cancer<br>0.5
Jordan	Ischemic heart disease<br>0.74	Total cancers<br>0.57	Stroke<br>0.9	Diabetes<br>2.09	Hypertensive heart disease<br>3.57	Chronic kidney disease<br>1.99	Alzheimer's disease<br>1.12	Lung cancer<br>0.59	COPD<br>0.5	Lower respiratory infect<br>0.7
Kuwait	Ischemic heart disease<br>0.92	Total cancers<br>0.43	Stroke<br>1.04	Lower respiratory infect<br>2.04	Alzheimer's disease<br>1.26	Diabetes<br>1.88	Hypertensive heart disease<br>3.15	Chronic kidney disease<br>1.34	Lung cancer<br>0.34	COPD<br>0.41
Lebanon	Ischemic heart disease<br>1.69	Total cancers<br>1.04	Stroke<br>0.43	Prostate cancer<br>1.12	Lung cancer<br>1.13	Hypertensive heart disease<br>2.67	Alzheimer's disease<br>1.16	Lower respiratory infect<br>1.06	COPD<br>0.71	Chronic kidney disease<br>1.3
Libya	Ischemic heart disease<br>0.96	Total cancers<br>0.65	Stroke<br>0.71	Alzheimer's disease<br>1.29	Hypertensive heart disease<br>2.33	Chronic kidney disease<br>1.44	COPD<br>0.66	Lung cancer<br>0.84	Lower respiratory infect<br>0.75	Diabetes<br>0.78
Morocco	Ischemic heart disease<br>1.64	Stroke<br>1.05	Total cancers<br>0.67	Hypertensive heart disease<br>2.2	Chronic kidney disease<br>1.37	COPD<br>0.67	Alzheimer's disease<br>1.14	Lower respiratory infect<br>0.69	Diabetes<br>0.67	Lung cancer<br>0.95
Oman	Ischemic heart disease<br>2.77	Stroke<br>1.88	Total cancers<br>0.57	Diabetes<br>4.57	Lower respiratory infect<br>2.54	Road injuries<br>9.83	COPD<br>1.03	Alzheimer's disease<br>1.41	Chronic kidney disease<br>1.72	Hypertensive heart disease<br>2.82
Palestine	Ischemic heart disease<br>1.17	Total cancers<br>1.1	Stroke<br>1.09	Diabetes<br>2.47	Hypertensive heart disease<br>2.03	Lung cancer<br>1.6	Chronic kidney disease<br>1.49	Lower respiratory infect<br>1.04	COPD<br>0.64	Alzheimer's disease<br>1.15
Qatar	Ischemic heart disease<br>1.84	Total cancers<br>0.79	Diabetes<br>8.83	Stroke<br>0.73	Chronic kidney disease<br>3.47	Lung cancer<br>0.61	Cirrhosis<br>3.37	Road injuries<br>6.72	Liver cancer<br>3.6	Total burden related to hep<br>6.14
Saudi Arabia	Ischemic heart disease<br>1.43	Stroke<br>1.58	Total cancers<br>0.38	Chronic kidney disease<br>4.13	Cirrhosis<br>3.9	Lower respiratory infect<br>1.41	Alzheimer's disease<br>1.31	COPD<br>0.7	Diabetes<br>1.42	Total burden related to hep<br>6.33
Sudan	Ischemic heart disease<br>1.56	Stroke<br>1.02	Total cancers<br>0.76	Hypertensive heart disease<br>2.54	COPD<br>0.7	Chronic kidney disease<br>1.05	Alzheimer's disease<br>1.05	Lower respiratory infect<br>0.63	Cirrhosis<br>1.04	Stomach cancer<br>1.31
Syrian Arab Republic	Ischemic heart disease<br>1.64	Stroke<br>0.73	Total cancers<br>0.5	Conflict & terror<br>2998.5	Alzheimer's disease<br>1.14	Chronic kidney disease<br>1.01	COPD<br>0.56	Lower respiratory infect<br>0.75	Cirrhosis<br>1.14	Diabetes<br>0.51
Tunisia	Ischemic heart disease<br>1.22	Stroke<br>0.86	Total cancers<br>0.63	Alzheimer's disease<br>1.2	Hypertensive heart disease<br>1.97	Lung cancer<br>1.03	COPD<br>0.68	Chronic kidney disease<br>1.23	Lower respiratory infect<br>0.82	Diabetes<br>0.68
Turkey	Ischemic heart disease<br>0.85	Total cancers<br>0.81	Stroke<br>0.8	COPD<br>1.64	Lung cancer<br>1.26	Alzheimer's disease<br>1.15	Chronic kidney disease<br>1.52	Lower respiratory infect<br>0.91	Hypertensive heart disease<br>2.18	Diabetes<br>1.21
United Arab Emirates	Ischemic heart disease<br>1.64	Total cancers<br>0.72	Stroke<br>2.05	Diabetes<br>5.81	Lower respiratory infect<br>2.99	Hypertensive heart disease<br>11.02	Chronic kidney disease<br>4.6	COPD<br>1.04	Lung cancer<br>0.51	Road injuries<br>8.72
Yemen	Ischemic heart disease<br>1.89	Stroke<br>1.1	Total cancers<br>0.86	Hypertensive heart disease<br>2.64	COPD<br>0.73	Lower respiratory infect<br>0.56	Alzheimer's disease<br>1.14	Chronic kidney disease<br>0.85	Stomach cancer<br>1.73	Asthma<br>1.34
**South Asia**	Ischemic heart disease<br>0.8	COPD<br>2.4	Stroke<br>0.64	Total cancers<br>0.58	Diarrheal diseases<br>7.99	Lower respiratory infect<br>0.87	Diabetes<br>0.95	Tuberculosis<br>2.89	Alzheimer's disease<br>0.83	Chronic kidney disease<br>0.71
**South Asia**	Ischemic heart disease<br>0.8	COPD<br>2.4	Stroke<br>0.64	Total cancers<br>0.58	Diarrheal diseases<br>7.99	Lower respiratory infect<br>0.87	Diabetes<br>0.95	Tuberculosis<br>2.89	Alzheimer's disease<br>0.83	Chronic kidney disease<br>0.71
Bangladesh	Stroke<br>1.15	Ischemic heart disease<br>0.59	Total cancers<br>0.61	COPD<br>1.18	Diabetes<br>0.92	Diarrheal diseases<br>1.77	Lower respiratory infect<br>0.48	Tuberculosis<br>1.23	Alzheimer's disease<br>0.8	Cirrhosis<br>0.8
Bhutan	Ischemic heart disease<br>0.92	COPD<br>2.03	Total cancers<br>0.72	Stroke<br>0.61	Diabetes<br>1.07	Diarrheal diseases<br>1.71	Lower respiratory infect<br>0.51	Chronic kidney disease<br>0.88	Cirrhosis<br>1.11	Alzheimer's disease<br>0.91
India	Ischemic heart disease<br>0.8	COPD<br>2.6	Stroke<br>0.55	Total cancers<br>0.54	Diarrheal diseases<br>14.3	Lower respiratory infect<br>0.99	Diabetes<br>0.93	Tuberculosis<br>3.86	Falls<br>4.26	Chronic kidney disease<br>0.75
Nepal	COPD<br>3.81	Ischemic heart disease<br>0.98	Stroke<br>0.79	Total cancers<br>0.85	Tuberculosis<br>1.42	Lower respiratory infect<br>0.55	Cirrhosis<br>1.21	Asthma<br>2.02	Other malignant neoplasms<br>6.23	Chronic kidney disease<br>0.75
Pakistan	Ischemic heart disease<br>1.12	COPD<br>2.17	Total cancers<br>0.94	Stroke<br>0.79	Diarrheal diseases<br>3.03	Diabetes<br>1.36	Tuberculosis<br>1.56	Chronic kidney disease<br>1.09	Lower respiratory infect<br>0.61	Alzheimer's disease<br>0.88
**Southeast Asia, East Asia, and Oceania**	Stroke<br>1.65	Total cancers<br>1.07	Ischemic heart disease<br>0.75	COPD<br>2.59	Lung cancer<br>1.67	Stomach cancer<br>1.86	Hypertensive heart disease<br>1.9	Lower respiratory infect<br>0.93	Alzheimer's disease<br>0.95	Colorectal cancer<br>1.04
**East Asia**	Stroke<br>1.74	Total cancers<br>1.14	Ischemic heart disease<br>0.78	COPD<br>2.84	Lung cancer<br>1.8	Stomach cancer<br>2.17	Hypertensive heart disease<br>2.06	Esophageal cancer<br>5.18	Alzheimer's disease<br>0.95	Colorectal cancer<br>1.05
China	Stroke<br>1.75	Total cancers<br>1.15	Ischemic heart disease<br>0.79	COPD<br>2.86	Lung cancer<br>1.83	Stomach cancer<br>2.19	Hypertensive heart disease<br>2.08	Esophageal cancer<br>5.32	Alzheimer's disease<br>0.96	Colorectal cancer<br>1.05
Democratic People's Republic of Korea	Stroke<br>1.81	COPD<br>3.41	Ischemic heart disease<br>0.77	Total cancers<br>1.05	Lung cancer<br>1.71	Lower respiratory infect<br>0.87	Stomach cancer<br>1.88	Hypertensive heart disease<br>1.25	Alzheimer's disease<br>0.81	Chronic kidney disease<br>0.5
Taiwan (Province of China)	Total cancers<br>0.81	Ischemic heart disease<br>0.42	Lower respiratory infect<br>2	Stroke<br>0.8	COPD<br>1.38	Lung cancer<br>0.9	Diabetes<br>2.53	Colorectal cancer<br>1.06	Alzheimer's disease<br>0.9	Chronic kidney disease<br>1.74
**Oceania**	Ischemic heart disease<br>1.1	COPD<br>2.6	Stroke<br>1.14	Diabetes<br>3.76	Total cancers<br>0.89	Diarrheal diseases<br>2.28	Asthma<br>3.41	Lower respiratory infect<br>0.74	Lung cancer<br>1.46	Prostate cancer<br>1.1
American Samoa	Total cancers<br>1.01	Ischemic heart disease<br>0.77	Stroke<br>1.02	Diabetes<br>4.14	COPD<br>1.72	Chronic kidney disease<br>3.17	Prostate cancer<br>1.95	Lung cancer<br>1.13	Lower respiratory infect<br>1.02	Alzheimer's disease<br>0.93
Cook Islands	Total cancers<br>1	Ischemic heart disease<br>0.64	Diabetes<br>5.58	Prostate cancer<br>2.39	Stroke<br>0.82	Hypertensive heart disease<br>7.1	Lower respiratory infect<br>1.95	Lung cancer<br>1.22	Chronic kidney disease<br>1.98	COPD<br>0.84
Fiji	Ischemic heart disease<br>1.4	Diabetes<br>9.21	Stroke<br>0.97	Total cancers<br>0.67	Asthma<br>15.44	Chronic kidney disease<br>2.64	COPD<br>1.2	Hypertensive heart disease<br>2.09	Lower respiratory infect<br>1.26	Prostate cancer<br>1.01
Guam	Ischemic heart disease<br>1.19	Total cancers<br>0.62	Stroke<br>0.73	Lung cancer<br>0.9	COPD<br>0.96	Alzheimer's disease<br>0.93	Chronic kidney disease<br>1.69	Prostate cancer<br>0.7	Diabetes<br>1.29	Lower respiratory infect<br>0.63
Kiribati	Stroke<br>2.15	Ischemic heart disease<br>0.92	Diabetes<br>4.89	Total cancers<br>1.09	COPD<br>1.8	Asthma<br>7.31	Tuberculosis<br>6.16	Diarrheal diseases<br>7.4	Chronic kidney disease<br>1.48	Lower respiratory infect<br>1.05
Marshall Islands	Ischemic heart disease<br>1.17	Stroke<br>1.31	Total cancers<br>1.09	COPD<br>1.63	Diabetes<br>2.14	Lower respiratory infect<br>1.22	Chronic kidney disease<br>1.51	Lung cancer<br>1.74	Prostate cancer<br>1.34	Hypertensive heart disease<br>1.5
Micronesia (Federated States of)	Ischemic heart disease<br>1.18	Stroke<br>1.38	Total cancers<br>1.16	Diabetes<br>4.04	COPD<br>2.04	Chronic kidney disease<br>2.52	Lower respiratory infect<br>1.61	Lung cancer<br>1.8	Prostate cancer<br>1.46	Hypertensive heart disease<br>1.76
Nauru	Ischemic heart disease<br>1.41	Stroke<br>1.44	Total cancers<br>1.2	Diabetes<br>3.98	COPD<br>2.15	Chronic kidney disease<br>2.36	Lower respiratory infect<br>1.89	Lung cancer<br>1.95	Prostate cancer<br>1.44	Hypertensive heart disease<br>1.53
Niue	Ischemic heart disease<br>1.17	Total cancers<br>0.96	Stroke<br>1.31	Diabetes<br>5.19	COPD<br>1.8	Chronic kidney disease<br>2.67	Lower respiratory infect<br>1.58	Prostate cancer<br>1.37	Lung cancer<br>1.34	Alzheimer's disease<br>1
Northern Mariana Islands	Total cancers<br>1.12	Ischemic heart disease<br>0.95	Stroke<br>1.44	Lung cancer<br>1.82	COPD<br>1.83	Diabetes<br>3.53	Chronic kidney disease<br>3.59	Prostate cancer<br>1.18	Lower respiratory infect<br>1.38	Alzheimer's disease<br>1.04
Palau	Ischemic heart disease<br>1.26	Total cancers<br>0.92	Stroke<br>1.47	Diabetes<br>4.95	Lower respiratory infect<br>3.59	COPD<br>2.22	Chronic kidney disease<br>4.34	Prostate cancer<br>1.43	Lung cancer<br>1.14	Asthma<br>9.74
Papua New Guinea	COPD<br>2.93	Ischemic heart disease<br>0.98	Stroke<br>1.12	Diabetes<br>3.35	Total cancers<br>0.93	Diarrheal diseases<br>2.11	Asthma<br>3.01	Lower respiratory infect<br>0.58	Lung cancer<br>1.77	Prostate cancer<br>1.1
Samoa	Ischemic heart disease<br>0.89	Stroke<br>1.04	Total cancers<br>0.59	COPD<br>1.88	Diabetes<br>2.67	Lower respiratory infect<br>1.47	Chronic kidney disease<br>1.78	Alzheimer's disease<br>1.04	Hypertensive heart disease<br>1.38	Prostate cancer<br>0.62

Solomon Islands	Ischemic heart disease 2.19	Stroke 1.97	Total cancers 1.18	COPD 2.27	Lower respiratory infect 1.91	Diabetes 2.66	Diarrheal diseases 2.85	Lung cancer 2.29	Asthma 2.43	Prostate cancer 1.28
Tokelau	Ischemic heart disease 0.67	Total cancers 0.75	Stroke 0.71	Diabetes 2.03	COPD 1.24	Chronic kidney disease 1.35	Lower respiratory infect 1.06	Alzheimer's disease 0.96	Prostate cancer 0.99	Lung cancer 1.16
Tonga	Total cancers 1.32	Ischemic heart disease 0.75	Diabetes 3.35	COPD 1.96	Stroke 0.63	Lower respiratory infect 1.76	Chronic kidney disease 1.94	Lung cancer 1.82	Prostate cancer 1.48	Alzheimer's disease 0.94
Tuvalu	Ischemic heart disease 1	Stroke 1.07	Total cancers 0.96	Diabetes 2.57	COPD 1.65	Chronic kidney disease 1.67	Lower respiratory infect 1.34	Prostate cancer 1.29	Lung cancer 1.46	Alzheimer's disease 1
Vanuatu	Ischemic heart disease 1.49	Stroke 1.42	Total cancers 1.06	COPD 1.86	Diabetes 1.69	Lower respiratory infect 0.98	Diarrheal diseases 2.8	Lung cancer 1.75	Chronic kidney disease 1.17	Prostate cancer 1.3
**Southeast Asia**	Stroke 1.31	Ischemic heart disease 0.64	Total cancers 0.78	COPD 1.65	Lower respiratory infect 1.73	Diabetes 1.25	Lung cancer 1.07	Tuberculosis 7.32	Chronic kidney disease 1.1	Alzheimer's disease 0.92
Cambodia	Stroke 1.3	Ischemic heart disease 0.87	Total cancers 1.23	Lower respiratory infect 2.17	COPD 1.21	Tuberculosis 2.88	Lung cancer 2.26	Cirrhosis 1.94	Upper digest diseases 4.46	Diabetes 0.93
Indonesia	Stroke 1.73	Ischemic heart disease 0.73	Total cancers 0.78	COPD 2	Diarrheal diseases 52.59	Diabetes 1.5	Tuberculosis 14.37	Cirrhosis 2.86	Lung cancer 1.12	Hypertensive heart disease 2.03
Lao People's Democratic Republic	Stroke 1.5	Ischemic heart disease 1	Total cancers 0.94	COPD 1.54	Lower respiratory infect 1.42	Tuberculosis 2.69	Chronic kidney disease 1.32	Lung cancer 1.68	Diabetes 0.92	Asthma 2.19
Malaysia	Ischemic heart disease 0.92	Lower respiratory infect 4.74	Total cancers 0.71	Stroke 1.05	COPD 1.3	Lung cancer 0.78	Alzheimer's disease 0.95	Chronic kidney disease 1.23	Colorectal cancer 1.13	Cirrhosis 1.51
Maldives	Ischemic heart disease 0.79	Stroke 0.65	Total cancers 0.61	COPD 0.95	Chronic kidney disease 1.25	Alzheimer's disease 0.91	Diabetes 0.77	Lower respiratory infect 0.39	Lung cancer 0.75	Prostate cancer 0.4
Mauritius	Ischemic heart disease 0.65	Diabetes 4.8	Total cancers 0.59	Stroke 0.8	Chronic kidney disease 3.71	COPD 0.97	Hypertensive heart disease 2.18	Lower respiratory infect 0.98	Alzheimer's disease 0.9	Prostate cancer 0.65
Myanmar	Stroke 1.84	COPD 2.67	Total cancers 0.99	Ischemic heart disease 0.6	Diabetes 1.67	Lower respiratory infect 1.17	Asthma 3.32	Tuberculosis 2.67	Lung cancer 1.57	Hypertensive heart disease 1.14
Philippines	Ischemic heart disease 0.72	Stroke 0.83	Lower respiratory infect 3.42	Total cancers 0.67	COPD 1.45	Chronic kidney disease 1.54	Hypertensive heart disease 2.08	Tuberculosis 9.57	Diabetes 1.08	Alzheimer's disease 0.9
Seychelles	Total cancers 1.3	Ischemic heart disease 0.68	Lower respiratory infect 4.25	Stroke 0.85	Prostate cancer 2.36	COPD 1.16	Hypertensive heart disease 3.76	Chronic kidney disease 2.24	Lung cancer 0.81	Colorectal cancer 1.41
Sri Lanka	Ischemic heart disease 0.71	Stroke 0.81	Total cancers 0.49	Diabetes 2.72	Asthma 17.04	COPD 1.05	Chronic kidney disease 1.33	Lower respiratory infect 1.04	Alzheimer's disease 0.96	Hypertensive heart disease 1.41
Thailand	Total cancers 0.75	Ischemic heart disease 0.32	Stroke 0.5	Lower respiratory infect 1.57	COPD 0.98	Lung cancer 1.03	Alzheimer's disease 0.88	Chronic kidney disease 1.22	Liver cancer 5.41	Diabetes 0.64
Timor-Leste	Stroke 1.4	Ischemic heart disease 0.95	Total cancers 0.83	COPD 1.54	Lower respiratory infect 1.69	Tuberculosis 2.68	Diarrheal diseases 3.07	Chronic kidney disease 1.01	Lung cancer 1.29	Hypertensive heart disease 1.28
Viet Nam	Stroke 1.92	Ischemic heart disease 0.72	Total cancers 0.94	COPD 1.77	Lower respiratory infect 1.23	Tuberculosis 8.51	Diabetes 1.16	Lung cancer 1.32	Alzheimer's disease 1.13	Chronic kidney disease 1.01
**Sub-Saharan Africa**	Total cancers 0.99	Ischemic heart disease 0.68	Stroke 0.85	Lower respiratory infect 1.58	Tuberculosis 1.81	Diarrheal diseases 1.87	COPD 0.79	Diabetes 1.41	Prostate cancer 1.64	Chronic kidney disease 1.12
**Central Sub-Saharan Africa**	Stroke 0.98	Lower respiratory infect 1.98	Total cancers 1	Ischemic heart disease 0.66	Tuberculosis 2.78	COPD 0.94	Diarrheal diseases 2.24	Diabetes 1.58	Hypertensive heart disease 1.81	Prostate cancer 1.42
Angola	Stroke 1.06	Total cancers 1.09	Ischemic heart disease 0.67	Lower respiratory infect 2.14	Tuberculosis 3.67	Diarrheal diseases 3.79	Diabetes 1.71	Cirrhosis 2	Prostate cancer 1.55	COPD 0.67
Central African Republic	Tuberculosis 2.93	Diarrheal diseases 2.88	Stroke 1.21	Lower respiratory infect 1.54	Ischemic heart disease 0.92	Total cancers 1.07	COPD 1.21	Diabetes 1.67	Hypertensive heart disease 2.17	Chronic kidney disease 1.27
Congo	Total cancers 1.03	Stroke 0.96	Ischemic heart disease 0.62	Lower respiratory infect 2.66	Diabetes 1.79	Tuberculosis 6.85	Diarrheal diseases 14.61	COPD 0.9	Prostate cancer 1.52	Hypertensive heart disease 1.91
Democratic Republic of the Congo	Lower respiratory infect 1.9	Stroke 0.95	Total cancers 0.96	Ischemic heart disease 0.66	Tuberculosis 2.5	COPD 1	Diarrheal diseases 1.76	Diabetes 1.46	Hypertensive heart disease 1.82	Prostate cancer 1.35
Equatorial Guinea	Total cancers 0.97	Stroke 0.96	Lower respiratory infect 3.79	Ischemic heart disease 0.48	Diabetes 3.07	Prostate cancer 1.53	COPD 1.15	Chronic kidney disease 2.02	Malaria 8517	Tuberculosis 13.62
Gabon	Total cancers 1.12	Ischemic heart disease 0.59	Stroke 1.02	Lower respiratory infect 3.89	Diabetes 3.31	Prostate cancer 1.62	Chronic kidney disease 2.14	Tuberculosis 15.35	Hypertensive heart disease 2.75	COPD 1
**Eastern Sub-Saharan Africa**	Stroke 0.94	Ischemic heart disease 0.66	Total cancers 0.91	Lower respiratory infect 1.45	Tuberculosis 1.69	Diarrheal diseases 1.7	COPD 0.79	Diabetes 1.37	Cirrhosis 1.51	Chronic kidney disease 1.04
Burundi	Stroke 0.96	Ischemic heart disease 0.81	Tuberculosis 1.66	Lower respiratory infect 1.19	Total cancers 0.91	Diarrheal diseases 1.24	COPD 1.05	Diabetes 1.16	Prostate cancer 1.24	Chronic kidney disease 0.93
Comoros	Ischemic heart disease 0.65	Total cancers 0.89	Stroke 0.8	Lower respiratory infect 1.61	Tuberculosis 2.56	COPD 0.73	Diarrheal diseases 2.49	Diabetes 1.25	Prostate cancer 1.35	Chronic kidney disease 1.02
Djibouti	Total cancers 1.06	Ischemic heart disease 0.74	Stroke 0.93	Lower respiratory infect 1.85	Tuberculosis 2.5	Diabetes 1.51	Prostate cancer 1.59	Diarrheal diseases 2.25	COPD 0.58	Chronic kidney disease 1.13
Eritrea	Lower respiratory infect 2.62	Stroke 1.02	Tuberculosis 4.02	Ischemic heart disease 0.75	Total cancers 0.95	Diarrheal diseases 3.85	COPD 0.9	Diabetes 1.38	Cirrhosis 1.56	Prostate cancer 1.35
Ethiopia	Stroke 0.74	Lower respiratory infect 1.29	Ischemic heart disease 0.57	Total cancers 0.69	Diarrheal diseases 1.72	Tuberculosis 1.11	Cirrhosis 1.75	COPD 0.73	Diabetes 1.31	Chronic kidney disease 1.05
Kenya	Stroke 0.98	Lower respiratory infect 2.52	Total cancers 0.88	Ischemic heart disease 0.53	Diarrheal diseases 5.57	Tuberculosis 3.99	Cirrhosis 2.48	COPD 0.88	Diabetes 1.36	Prostate cancer 1.31
Madagascar	Ischemic heart disease 0.94	Stroke 1.19	Lower respiratory infect 1.38	Total cancers 0.72	Tuberculosis 2.21	COPD 1.1	Diarrheal diseases 1.75	Hypertensive heart disease 1.88	Diabetes 0.96	Cirrhosis 1.12
Malawi	Stroke 1.02	Total cancers 1.03	Ischemic heart disease 0.67	Lower respiratory infect 1.66	Tuberculosis 2.84	Diarrheal diseases 2.38	Diabetes 1.56	COPD 0.77	Cirrhosis 1.71	Chronic kidney disease 1.11
Mozambique	Stroke 1.48	Tuberculosis 2.39	Ischemic heart disease 0.84	Lower respiratory infect 1.38	Total cancers 0.97	Diabetes 1.7	Diarrheal diseases 1.04	COPD 0.76	Hypertensive heart disease 1.87	Malaria 4.49
Rwanda	Stroke 0.98	Total cancers 1.04	Ischemic heart disease 0.57	Lower respiratory infect 1.6	COPD 1.03	Tuberculosis 2.03	Diabetes 1.46	Diarrheal diseases 2.03	Prostate cancer 1.51	Cirrhosis 1.55
Somalia	Tuberculosis 0.66	Stroke 1.02	Ischemic heart disease 1.07	Lower respiratory infect 0.7	Total cancers 0.97	Diarrheal diseases 0.32	COPD 1.06	Diabetes 1.3	Cirrhosis 0.96	Hypertensive heart disease 1.64
South Sudan	Stroke 0.78	Total cancers 0.92	Ischemic heart disease 0.6	Diarrheal diseases 2.71	Lower respiratory infect 1.24	Tuberculosis 2.07	COPD 0.75	Diabetes 1.21	Cirrhosis 1.35	Chronic kidney disease 1.05
Uganda	Total cancers 1.42	Stroke 0.93	Ischemic heart disease 0.63	Lower respiratory infect 1.58	Tuberculosis 2.53	Prostate cancer 2.71	COPD 0.96	Diabetes 1.68	Diarrheal diseases 1.89	Chronic kidney disease 1.18
United Republic of Tanzania	Ischemic heart disease 0.78	Stroke 0.93	Total cancers 1.03	Lower respiratory infect 1.46	Tuberculosis 2.13	Diarrheal diseases 2.18	Diabetes 1.33	Prostate cancer 1.48	COPD 0.56	Chronic kidney disease 1
Zambia	Stroke 1.47	Total cancers 1.09	Ischemic heart disease 0.6	Lower respiratory infect 2.11	Tuberculosis 4.52	Diabetes 1.67	Cirrhosis 2.43	COPD 0.83	Hypertensive heart disease 2.29	Diarrheal diseases 3.8
**Southern Sub-Saharan Africa**	Total cancers 1.03	Ischemic heart disease 0.52	Stroke 0.77	Lower respiratory infect 2.56	Diabetes 2.5	COPD 1.37	Prostate cancer 1.8	Tuberculosis 9.88	Chronic kidney disease 1.75	Diarrheal diseases 11.36
Botswana	Total cancers 1.2	Ischemic heart disease 0.71	Stroke 1.14	Lower respiratory infect 3.97	Diabetes 3.15	COPD 1.86	Prostate cancer 2.14	Chronic kidney disease 1.92	Tuberculosis 10.01	HIV/AIDS 529.21
Eswatini	Total cancers 1.59	Diabetes 4.94	Stroke 1.13	Ischemic heart disease 0.65	Lower respiratory infect 2.96	COPD 1.9	Chronic kidney disease 2.79	Tuberculosis 9.68	Prostate cancer 2.28	Diarrheal diseases 21.04
Lesotho	Total cancers 1.64	Stroke 1.37	Tuberculosis 8.76	COPD 2.11	Ischemic heart disease 0.67	Lower respiratory infect 2.72	Diarrheal diseases 10.84	Diabetes 3.24	Prostate cancer 2.6	Chronic kidney disease 2.15
Namibia	Stroke 1.19	Ischemic heart disease 0.69	Total cancers 0.89	Lower respiratory infect 3.88	COPD 2	Diabetes 2.31	Prostate cancer 1.77	Diarrheal diseases 30.58	Tuberculosis 9.29	Chronic kidney disease 1.58
South Africa	Total cancers 0.96	Ischemic heart disease 0.47	Stroke 0.72	Diabetes 2.66	Lower respiratory infect 2.43	COPD 1.45	Prostate cancer 1.66	Chronic kidney disease 1.76	Hypertensive heart disease 2.04	Tuberculosis 12.09
Zimbabwe	Total cancers 1.47	Ischemic heart disease 0.88	Tuberculosis 7.89	Lower respiratory infect 2.73	Stroke 0.77	Prostate cancer 2.86	Diarrheal diseases 4.66	Chronic kidney disease 1.54	COPD 0.66	Diabetes 1.2
**Western Sub-Saharan Africa**	Ischemic heart disease 0.73	Total cancers 1.03	Stroke 0.78	Lower respiratory infect 1.49	Prostate cancer 1.99	Malaria 16.22	Tuberculosis 1.48	Diarrheal diseases 1.76	COPD 0.65	Diabetes 1.18
Benin	Ischemic heart disease 0.81	Stroke 0.96	Total cancers 1.1	Lower respiratory infect 1.53	Malaria 12.7	Diarrheal diseases 1.31	Tuberculosis 0.99	COPD 0.68	Prostate cancer 1.58	Chronic kidney disease 1.19
Burkina Faso	Ischemic heart disease 1.17	Lower respiratory infect 1.32	Total cancers 1.09	Stroke 0.71	Diarrheal diseases 0.88	Malaria 5.52	Tuberculosis 0.66	Diabetes 1.17	Prostate cancer 1.5	Chronic kidney disease 1.03
Cabo Verde	Total cancers 2.16	Ischemic heart disease 1	Stroke 0.94	Lower respiratory infect 2.35	Prostate cancer 3.33	Stomach cancer 3.14	Chronic kidney disease 1.26	Alzheimer's disease 0.96	COPD 0.5	Diabetes 1.01
Cameroon	Total cancers 1.13	Ischemic heart disease 0.78	Stroke 0.92	Lower respiratory infect 2.39	Malaria 423.2	Diabetes 1.5	Prostate cancer 1.75	Chronic kidney disease 1.5	Tuberculosis 2.44	COPD 0.66
Chad	Ischemic heart disease 0.92	Lower respiratory infect 1.18	Total cancers 1.08	Stroke 0.81	Diarrheal diseases 1.01	Tuberculosis 0.76	COPD 0.72	Prostate cancer 1.42	Chronic kidney disease 1.08	Diabetes 0.82
Côte d'Ivoire	Ischemic heart disease 0.86	Total cancers 1.13	Lower respiratory infect 1.9	Stroke 0.83	Malaria 55.91	Tuberculosis 1.64	Prostate cancer 1.58	Chronic kidney disease 1.31	COPD 0.67	Diabetes 1.13
Gambia	Ischemic heart disease 1.01	Lower respiratory infect 2.18	Stroke 0.88	Total cancers 0.85	Tuberculosis 2.25	COPD 0.77	Chronic kidney disease 1.25	Diarrheal diseases 1.45	Liver cancer 7.35	Diabetes 1.06
Ghana	Lower respiratory infect 3.93	Total cancers 1.02	Ischemic heart disease 0.66	Stroke 0.84	Tuberculosis 8.91	Prostate cancer 1.99	COPD 1.01	Diabetes 1.62	Malaria 954.89	Chronic kidney disease 1.17
Guinea	Total cancers 1.44	Lower respiratory infect 1.71	Ischemic heart disease 0.84	Stroke 0.82	Tuberculosis 1.19	Diarrheal diseases 1.28	Prostate cancer 1.8	COPD 0.74	Liver cancer 8.69	Chronic kidney disease 1.11
Guinea-Bissau	Ischemic heart disease 1.12	Lower respiratory infect 2.18	Total cancers 1.24	Stroke 1.05	Tuberculosis 1.8	Diarrheal diseases 1.9	COPD 0.91	Chronic kidney disease 1.45	Prostate cancer 1.66	Diabetes 1.24
Liberia	Ischemic heart disease 0.76	Total cancers 0.99	Stroke 0.7	Lower respiratory infect 1.24	Malaria 18.3	Diarrheal diseases 1.27	Tuberculosis 1.08	Prostate cancer 1.48	Chronic kidney disease 1.07	Diabetes 0.91
Mali	Ischemic heart disease 0.87	Stroke 0.8	Total cancers 0.94	Diarrheal diseases 1.27	Tuberculosis 0.67	Lower respiratory infect 0.46	COPD 0.7	Chronic kidney disease 0.97	Alzheimer's disease 1.08	Diabetes 0.77
Mauritania	Ischemic heart disease 0.61	Total cancers 0.92	Lower respiratory infect 1.78	Stroke 0.59	Malaria 366.79	Prostate cancer 1.46	Diarrheal diseases 2.87	Alzheimer's disease 1.05	Chronic kidney disease 0.99	Diabetes 0.88
Niger	Ischemic heart disease 0.95	Lower respiratory infect 0.81	Stroke 0.74	Total cancers 0.95	Diarrheal diseases 0.51	Malaria 2.65	Tuberculosis 0.35	COPD 0.68	Prostate cancer 1.35	Chronic kidney disease 0.94
Nigeria	Total cancers 0.99	Ischemic heart disease 0.63	Stroke 0.75	Lower respiratory infect 1.48	Prostate cancer 2.45	Malaria 604.77	Diarrheal diseases 4.34	Tuberculosis 3.29	Diabetes 1.28	COPD 0.59
Sao Tome and Principe	Ischemic heart disease 0.97	Total cancers 1.28	Lower respiratory infect 2.5	Stroke 0.89	COPD 1.53	Chronic kidney disease 1.62	Cirrhosis 1.87	Prostate cancer 1.13	Lung cancer 1.43	Falls 4.07
Senegal	Ischemic heart disease 0.8	Total cancers 1.12	Stroke 0.72	Lower respiratory infect 1.15	Diarrheal diseases 1.97	Prostate cancer 1.74	COPD 0.66	Chronic kidney disease 1.27	Malaria 16.19	Tuberculosis 1.12
Sierra Leone	Ischemic heart disease 0.87	Lower respiratory infect 1.54	Total cancers 0.98	Stroke 0.77	Diarrheal diseases 2.03	Malaria 12.7	Tuberculosis 1	COPD 0.63	Prostate cancer 1.43	Chronic kidney disease 0.93
Togo	Ischemic heart disease 1	Total cancers 1.11	Lower respiratory infect 2.21	Stroke 0.89	Malaria 52.27	Tuberculosis 2.1	Diarrheal diseases 2.18	COPD 0.73	Prostate cancer 1.65	Chronic kidney disease 1.19

Values shown in brackets represent the ratio of observed deaths to predicted deaths on the basis of Socio-Demographic Index (SDI), rounded to two (2) digits. Color ranges (shown below) were calculated to place a roughly equal number of cells into each bin.

COLOR KEY:	[0.00,0.65)	[0.65,0.78)	[0.78,0.9)	[0.9,0.99)	[0.99,1.09)	[1.09,1.25)	[1.25,1.5)	[1.5,1.87)	[1.87,2.69)	[2.69,...)

Figure S21. Leading ten causes of total deaths with ratio of observed to expected years lived with disability in 2019, among people 70 years and older, in females, for all GBD locations.

Causes are ranked based on global estimates of deaths. Causes are color coded (shown in color key) based on the ratio of observed to expected (O:E) rates on that basis of SDI from the epidemiologic transition analysis. Shades of blue represent much lower observed deaths and YLDs than expected levels based on SDI, whereas red shows that observed deaths and YLDs exceed expected levels. When the ratio exceeds 1 means that observed levels exceeded the expected levels on the basis of SDI. O:E ratios are listed in each cell.

Figure 21. Leading 10 causes of total deaths with ratio of observed to expected deaths in 2019, by location, females

	1	2	3	4	5	6	7	8	9	10
**Global**	Ischemic heart disease 0.82	Stroke 0.92	Total cancers 0.89	COPD 2.12	Alzheimer's disease 1.01	Lower respiratory infect 0.99	Hypertensive heart disease 1.15	Diabetes 0.79	Chronic kidney disease 1.02	Diarrheal diseases 5.39
Central Europe, Eastern Europe, and Central Asia	Ischemic heart disease 2.17	Stroke 1.83	Total cancers 0.79	Alzheimer's disease 0.97	Hypertensive heart disease 1.4	Colorectal cancer 0.93	COPD 0.72	Breast cancer 0.78	Diabetes 0.62	Cardiomyopathy 2.44
**Central Asia**	Ischemic heart disease 2.44	Stroke 1.56	Total cancers 0.83	Hypertensive heart disease 1.81	Alzheimer's disease 1	COPD 1.5	Cirrhosis 2.78	Diabetes 0.71	Lower respiratory infect 0.58	Chronic kidney disease 0.6
Armenia	Ischemic heart disease 1.69	Stroke 0.79	Total cancers 0.98	Alzheimer's disease 1.04	COPD 1.83	Diabetes 1.21	Hypertensive heart disease 1.45	Cirrhosis 3.65	Urinary diseases 5.19	Breast cancer 1.09
Azerbaijan	Ischemic heart disease 3.27	Stroke 2.02	Total cancers 0.87	Hypertensive heart disease 2.52	Cirrhosis 4.66	Alzheimer's disease 1.07	COPD 1.35	Diabetes 0.8	Stomach cancer 1.94	Chronic kidney disease 0.98
Georgia	Ischemic heart disease 1.29	Stroke 1.54	Hypertensive heart disease 4.22	Total cancers 0.71	Alzheimer's disease 0.98	Diabetes 0.7	COPD 0.64	Breast cancer 0.99	Lower respiratory infect 0.39	Atrial fibrillation 1.05
Kazakhstan	Ischemic heart disease 2.01	Stroke 2.04	Total cancers 0.75	COPD 2.64	Alzheimer's disease 0.99	Diabetes 0.83	Cirrhosis 2.75	Lower respiratory infect 0.76	Hypertensive heart disease 0.99	Cardiomyopathy 2.94
Kyrgyzstan	Ischemic heart disease 1.99	Stroke 0.77	Total cancers 0.67	Alzheimer's disease 0.99	COPD 1.52	Hypertensive heart disease 0.52	Cirrhosis 1.37	Colorectal cancer 0.73	Stomach cancer 0.83	Lower respiratory infect 0.22
Mongolia	Ischemic heart disease 1.49	Total cancers 2.35	Stroke 1.3	Liver cancer 32.42	Total burden related to hep 16.05	Cirrhosis 5.49	Alzheimer's disease 0.98	Stomach cancer 3.07	Esophageal cancer 13.24	Total burden related to hep 8.94
Tajikistan	Ischemic heart disease 2.86	Stroke 1.52	Total cancers 1	Hypertensive heart disease 2.24	Alzheimer's disease 1	COPD 1.05	Lower respiratory infect 0.73	Diabetes 0.65	Cirrhosis 1.86	Stomach cancer 1.89
Turkmenistan	Ischemic heart disease 2.03	Stroke 1.07	Total cancers 0.49	Alzheimer's disease 0.98	Hypertensive heart disease 0.89	Cirrhosis 1.98	COPD 0.5	Diabetes 0.3	Chronic kidney disease 0.48	Atrial fibrillation 0.87
Uzbekistan	Ischemic heart disease 4.63	Stroke 1.64	Total cancers 0.86	Diabetes 0.85	Hypertensive heart disease 1.56	Cirrhosis 3.29	Alzheimer's disease 0.97	COPD 1.07	Chronic kidney disease 0.81	Lower respiratory infect 0.76
**Central Europe**	Ischemic heart disease 1.52	Stroke 1.58	Total cancers 0.96	Alzheimer's disease 1.01	Hypertensive heart disease 2.5	Colorectal cancer 1.09	Cardiomyopathy 3.58	Diabetes 0.91	Breast cancer 0.98	COPD 0.77
Albania	Stroke 1.53	Ischemic heart disease 0.92	Total cancers 0.72	Alzheimer's disease 1.04	COPD 0.68	Lower respiratory infect 0.46	Other cardiovascular 2.17	Lung cancer 1.11	Hypertensive heart disease 0.49	Colorectal cancer 0.66
Bosnia and Herzegovina	Ischemic heart disease 1.33	Stroke 1.95	Total cancers 1.14	Diabetes 2.37	Alzheimer's disease 0.98	Colorectal cancer 1.24	Cardiomyopathy 4.45	COPD 0.94	Hypertensive heart disease 1.01	Breast cancer 1.14
Bulgaria	Ischemic heart disease 2.02	Stroke 2.85	Hypertensive heart disease 7.36	Total cancers 0.83	Alzheimer's disease 0.99	Colorectal cancer 1.11	Diabetes 0.93	COPD 0.87	Breast cancer 0.96	Other cardiovascular 2.83
Croatia	Ischemic heart disease 1.51	Stroke 1.41	Total cancers 1.02	Alzheimer's disease 1.04	Hypertensive heart disease 2.51	Falls 2.87	Colorectal cancer 1.15	COPD 0.95	Breast cancer 1.18	Diabetes 1.01
Czechia	Ischemic heart disease 1.66	Total cancers 0.95	Stroke 1.07	Alzheimer's disease 1.02	Diabetes 1.47	Lower respiratory infect 0.82	Colorectal cancer 0.97	Breast cancer 0.91	COPD 0.79	Lung cancer 0.95
Hungary	Ischemic heart disease 1.67	Total cancers 1.06	Stroke 1.01	Alzheimer's disease 1.01	Hypertensive heart disease 3.18	COPD 1.22	Colorectal cancer 1.26	Lung cancer 1.52	Breast cancer 1.01	Diabetes 0.86
Montenegro	Stroke 3.94	Ischemic heart disease 1.38	Total cancers 0.91	Cardiomyopathy 9.4	Alzheimer's disease 1.07	Diabetes 1.22	Breast cancer 1.17	Lung cancer 1.29	Atrial fibrillation 2.13	Colorectal cancer 0.81
North Macedonia	Stroke 4.46	Ischemic heart disease 1.74	Total cancers 1.13	Hypertensive heart disease 5.06	Diabetes 2.63	Alzheimer's disease 1.07	Cardiomyopathy 6.57	COPD 1.2	Breast cancer 1.34	Colorectal cancer 1.25
Poland	Ischemic heart disease 1.28	Total cancers 1.03	Stroke 1.05	Alzheimer's disease 1.04	Colorectal cancer 1.17	Cardiomyopathy 4.26	Hypertensive heart disease 1.39	Lower respiratory infect 0.72	Lung cancer 1.24	Breast cancer 0.95
Romania	Ischemic heart disease 1.55	Stroke 1.99	Total cancers 0.76	Hypertensive heart disease 3.35	Alzheimer's disease 0.97	Cardiomyopathy 5.4	COPD 0.76	Colorectal cancer 0.85	Breast cancer 0.81	Lower respiratory infect 0.52
Serbia	Stroke 3.17	Ischemic heart disease 2	Total cancers 1.16	Hypertensive heart disease 3.03	Diabetes 1.79	Alzheimer's disease 0.93	Cardiomyopathy 6.06	Breast cancer 1.51	Colorectal cancer 1.21	COPD 1.09
Slovakia	Ischemic heart disease 2.19	Total cancers 0.92	Stroke 1.13	Alzheimer's disease 1.02	Lower respiratory infect 1.06	Colorectal cancer 1.15	Hypertensive heart disease 1.5	Breast cancer 0.95	Diabetes 0.71	Chronic kidney disease 0.77
Slovenia	Total cancers 0.88	Ischemic heart disease 0.6	Stroke 0.78	Alzheimer's disease 1.04	Hypertensive heart disease 2.82	Cardiomyopathy 5.69	Lower respiratory infect 0.78	Falls 1.69	Colorectal cancer 0.87	Breast cancer 0.98
**Eastern Europe**	Ischemic heart disease 2.52	Stroke 2.02	Total cancers 0.67	Alzheimer's disease 0.94	Colorectal cancer 0.86	COPD 0.59	Breast cancer 0.66	Hypertensive heart disease 0.68	Cardiomyopathy 1.85	Atrial fibrillation 0.91
Belarus	Ischemic heart disease 2.58	Stroke 1.3	Total cancers 0.64	Alzheimer's disease 0.97	Colorectal cancer 0.83	COPD 0.56	Stomach cancer 1.08	Atrial fibrillation 0.88	Breast cancer 0.51	Alcohol use disorders 51.65
Estonia	Ischemic heart disease 1.63	Hypertensive heart disease 8.44	Total cancers 0.88	Stroke 0.72	Alzheimer's disease 1.01	Colorectal cancer 0.98	Chronic kidney disease 1.29	Breast cancer 0.74	Lung cancer 0.73	Pancreatic cancer 1.04
Latvia	Ischemic heart disease 2.04	Stroke 2.02	Total cancers 0.8	Alzheimer's disease 0.94	Hypertensive heart disease 1.75	Colorectal cancer 0.88	Breast cancer 0.76	Diabetes 0.62	Pancreatic cancer 1.01	COPD 0.42
Lithuania	Ischemic heart disease 2.56	Stroke 1.47	Total cancers 0.71	Alzheimer's disease 0.98	Colorectal cancer 0.74	Breast cancer 0.63	Hypertensive heart disease 0.89	COPD 0.47	Pancreatic cancer 0.8	Atrial fibrillation 0.79
Republic of Moldova	Ischemic heart disease 1.88	Stroke 0.91	Total cancers 0.54	Alzheimer's disease 0.96	Hypertensive heart disease 2.07	Cirrhosis 2.61	COPD 0.67	Colorectal cancer 0.74	Breast cancer 0.58	Atrial fibrillation 0.68
Russian Federation	Ischemic heart disease 2.27	Stroke 2.4	Total cancers 0.7	Alzheimer's disease 0.93	Colorectal cancer 0.9	COPD 0.62	Breast cancer 0.68	Hypertensive heart disease 0.79	Diabetes 0.63	Cardiomyopathy 1.98
Ukraine	Ischemic heart disease 3.24	Stroke 1.45	Total cancers 0.57	Alzheimer's disease 0.97	Colorectal cancer 0.75	COPD 0.54	Atrial fibrillation 1.18	Breast cancer 0.65	Cardiomyopathy 2.11	Stomach cancer 0.74
**High-income**	Total cancers 0.91	Ischemic heart disease 0.62	Alzheimer's disease 1.05	Stroke 0.67	Lower respiratory infect 0.97	COPD 1.24	Lung cancer 1.2	Chronic kidney disease 1.31	Colorectal cancer 0.83	Hypertensive heart disease 1.1
**Australasia**	Total cancers 0.91	Ischemic heart disease 0.64	Alzheimer's disease 1.04	Stroke 0.65	COPD 1.33	Lung cancer 1.19	Colorectal cancer 0.87	Chronic kidney disease 1.23	Lower respiratory infect 0.65	Atrial fibrillation 1.48
Australia	Total cancers 0.9	Ischemic heart disease 0.61	Alzheimer's disease 1.04	Stroke 0.63	COPD 1.28	Lung cancer 1.16	Chronic kidney disease 1.27	Colorectal cancer 0.83	Lower respiratory infect 0.63	Atrial fibrillation 1.47
New Zealand	Total cancers 0.97	Ischemic heart disease 0.8	Stroke 0.76	Alzheimer's disease 1.05	COPD 1.6	Colorectal cancer 1.13	Lung cancer 1.35	Lower respiratory infect 0.76	Atrial fibrillation 1.5	Breast cancer 0.86
**High-income Asia Pacific**	Total cancers 0.76	Alzheimer's disease 1.22	Stroke 0.65	Ischemic heart disease 0.34	Lower respiratory infect 1.13	Colorectal cancer 0.75	Lung cancer 0.88	Chronic kidney disease 1.03	Stomach cancer 1.63	Pancreatic cancer 0.92
Brunei Darussalam	Total cancers 1.46	Ischemic heart disease 1.26	Stroke 1.43	Diabetes 6.03	COPD 3.14	Lower respiratory infect 3.13	Lung cancer 2.62	Chronic kidney disease 3.19	Alzheimer's disease 1.11	Colorectal cancer 1.47
Japan	Total cancers 0.76	Alzheimer's disease 1.24	Stroke 0.6	Ischemic heart disease 0.32	Lower respiratory infect 1.09	Colorectal cancer 0.76	Chronic kidney disease 1.02	Lung cancer 0.87	Stomach cancer 1.63	Pancreatic cancer 0.95
Republic of Korea	Total cancers 0.76	Stroke 1.02	Ischemic heart disease 0.48	Alzheimer's disease 1.1	Lower respiratory infect 1.23	Diabetes 1.85	Lung cancer 0.95	Colorectal cancer 0.68	COPD 0.77	Hypertensive heart disease 1.45
Singapore	Total cancers 0.67	Lower respiratory infect 3.09	Ischemic heart disease 0.52	Alzheimer's disease 1	Stroke 0.49	Lung cancer 0.97	Colorectal cancer 0.81	Chronic kidney disease 1.21	Hypertensive heart disease 1.08	Urinary diseases 2.54
**High-income North America**	Total cancers 0.95	Ischemic heart disease 0.88	Alzheimer's disease 0.98	Stroke 0.67	COPD 2.03	Lung cancer 1.82	Chronic kidney disease 1.6	Lower respiratory infect 0.75	Breast cancer 0.89	Colorectal cancer 0.71
Canada	Total cancers 0.95	Ischemic heart disease 0.71	Alzheimer's disease 0.95	Stroke 0.6	Lung cancer 1.66	COPD 1.41	Lower respiratory infect 0.79	Colorectal cancer 0.81	Breast cancer 0.92	Falls 1.29
Greenland	Total cancers 1.77	Ischemic heart disease 0.66	Stroke 1.01	COPD 3.98	Lung cancer 4.87	Alzheimer's disease 1.03	Colorectal cancer 1.91	Lower respiratory infect 1.31	Falls 4.01	Pancreatic cancer 2.64
United States of America	Total cancers 0.95	Ischemic heart disease 0.9	Alzheimer's disease 0.98	Stroke 0.67	COPD 2.11	Lung cancer 1.83	Chronic kidney disease 1.65	Lower respiratory infect 0.74	Breast cancer 0.89	Colorectal cancer 0.7
**Southern Latin America**	Total cancers 1.2	Ischemic heart disease 0.48	Lower respiratory infect 2.69	Stroke 0.52	Alzheimer's disease 0.95	Chronic kidney disease 1.77	COPD 1.38	Hypertensive heart disease 1.24	Colorectal cancer 1.32	Breast cancer 1.38
Argentina	Total cancers 1.21	Ischemic heart disease 0.53	Lower respiratory infect 3.38	Stroke 0.47	Alzheimer's disease 0.96	COPD 1.47	Chronic kidney disease 1.81	Hypertensive heart disease 1.24	Breast cancer 1.59	Colorectal cancer 1.4
Chile	Total cancers 1.13	Stroke 0.63	Ischemic heart disease 0.38	Alzheimer's disease 0.93	Lower respiratory infect 1.27	Chronic kidney disease 1.87	COPD 1.21	Hypertensive heart disease 1.44	Diabetes 1.06	Colorectal cancer 1.01
Uruguay	Total cancers 1.43	Stroke 0.66	Ischemic heart disease 0.39	Alzheimer's disease 0.95	Lower respiratory infect 1.38	Colorectal cancer 1.86	COPD 1.14	Breast cancer 1.76	Chronic kidney disease 1.06	Hypertensive heart disease 0.83
**Western Europe**	Total cancers 0.95	Ischemic heart disease 0.63	Stroke 0.71	Alzheimer's disease 0.99	COPD 1.18	Lower respiratory infect 0.89	Hypertensive heart disease 1.48	Colorectal cancer 0.92	Chronic kidney disease 1.25	Breast cancer 1.06
Andorra	Total cancers 0.8	Ischemic heart disease 0.62	Alzheimer's disease 0.97	Stroke 0.56	Falls 2.16	Hypertensive heart disease 2.21	Lower respiratory infect 0.91	COPD 1.11	Chronic kidney disease 1.37	Colorectal cancer 0.68
Austria	Ischemic heart disease 0.98	Total cancers 0.83	Alzheimer's disease 0.95	Stroke 0.55	Hypertensive heart disease 2.33	Chronic kidney disease 2.06	COPD 0.84	Breast cancer 0.99	Diabetes 1.19	Cardiomyopathy 2.85
Belgium	Total cancers 0.96	Ischemic heart disease 0.57	Stroke 0.73	Alzheimer's disease 0.97	Lower respiratory infect 1.39	COPD 1.32	Breast cancer 1.24	Colorectal cancer 0.94	Chronic kidney disease 1.33	Falls 1.5
Cyprus	Total cancers 0.9	Ischemic heart disease 0.99	Stroke 1.1	Diabetes 3.29	Alzheimer's disease 1.05	COPD 1.69	Chronic kidney disease 2.41	Hypertensive heart disease 2.43	Breast cancer 1.2	Nonrheum valv diseases 3.17
Denmark	Total cancers 1.13	Ischemic heart disease 0.69	Stroke 0.86	COPD 2.48	Alzheimer's disease 0.93	Lung cancer 1.73	Lower respiratory infect 1.3	Colorectal cancer 1.12	Breast cancer 1.24	Diabetes 1.24
Finland	Ischemic heart disease 1.15	Total cancers 0.85	Stroke 0.87	Alzheimer's disease 0.95	Hypertensive heart disease 1.64	Breast cancer 0.82	Colorectal cancer 0.64	Lung cancer 0.77	Falls 1.16	Pancreatic cancer 1.18
France	Total cancers 0.9	Alzheimer's disease 1.03	Ischemic heart disease 0.4	Stroke 0.52	Lower respiratory infect 0.8	Colorectal cancer 0.91	Falls 1.61	Breast cancer 1.06	COPD 0.61	Chronic kidney disease 0.76
Germany	Ischemic heart disease 1.05	Total cancers 0.91	Stroke 0.81	Alzheimer's disease 0.95	Hypertensive heart disease 3.15	Chronic kidney disease 2.18	COPD 1.07	Colorectal cancer 0.9	Breast cancer 1.07	Lower respiratory infect 0.64
Greece	Ischemic heart disease 0.88	Stroke 1.39	Total cancers 0.99	Alzheimer's disease 0.96	Lower respiratory infect 1.33	COPD 1.38	Chronic kidney disease 1.66	Hypertensive heart disease 1.4	Breast cancer 1.3	Colorectal cancer 0.9
Iceland	Total cancers 0.76	Ischemic heart disease 0.64	Alzheimer's disease 0.96	Stroke 0.51	Lower respiratory infect 1	COPD 1.26	Lung cancer 1.35	Breast cancer 0.71	Colorectal cancer 0.55	Atrial fibrillation 1.02
Ireland	Total cancers 1.03	Ischemic heart disease 0.83	Stroke 0.76	Alzheimer's disease 0.99	Lower respiratory infect 1.69	COPD 2.09	Lung cancer 1.48	Colorectal cancer 0.86	Breast cancer 1.05	Chronic kidney disease 1.05
Israel	Total cancers 1.04	Ischemic heart disease 0.48	Alzheimer's disease 0.96	Stroke 0.51	Diabetes 1.98	Chronic kidney disease 2.17	Lower respiratory infect 0.88	Colorectal cancer 1.06	Breast cancer 1.22	COPD 0.9
Italy	Total cancers 0.93	Ischemic heart disease 0.56	Stroke 0.71	Alzheimer's disease 1.05	Hypertensive heart disease 2.15	Diabetes 1.14	COPD 0.88	Colorectal cancer 0.85	Breast cancer 1	Chronic kidney disease 0.97
Luxembourg	Total cancers 0.85	Ischemic heart disease 0.67	Stroke 0.77	Alzheimer's disease 0.91	COPD 1.2	Lower respiratory infect 0.8	Chronic kidney disease 1.39	Colorectal cancer 0.81	Breast cancer 1.01	Lung cancer 0.8
Malta	Ischemic heart disease 0.85	Total cancers 0.74	Stroke 0.59	Alzheimer's disease 0.94	Lower respiratory infect 1.19	Diabetes 1.02	Breast cancer 1.03	Chronic kidney disease 1.03	Colorectal cancer 0.73	Hypertensive heart disease 0.65
Monaco	Total cancers 1.54	Ischemic heart disease 0.88	Stroke 1.05	Alzheimer's disease 0.98	Lung cancer 1.99	Lower respiratory infect 1.31	Breast cancer 1.52	Colorectal cancer 1.1	Pancreatic cancer 2	COPD 0.92
Netherlands	Total cancers 1.13	Ischemic heart disease 0.55	Stroke 0.87	Alzheimer's disease 1.01	COPD 1.74	Lower respiratory infect 1.15	Colorectal cancer 1.13	Lung cancer 1.31	Breast cancer 1.26	Falls 1.68
Norway	Total cancers 0.89	Ischemic heart disease 0.74	Stroke 0.83	Alzheimer's disease 1	Lower respiratory infect 1.25	COPD 1.61	Colorectal cancer 1.02	Falls 1.44	Lung cancer 1.03	Atrial fibrillation 1.28
Portugal	Stroke 0.86	Total cancers 0.9	Ischemic heart disease 0.38	Alzheimer's disease 0.95	Lower respiratory infect 1.57	COPD 1.25	Diabetes 1.08	Chronic kidney disease 1.34	Colorectal cancer 1.09	Hypertensive heart disease 0.81
San Marino	Total cancers 1.1	Ischemic heart disease 0.57	Stroke 0.89	Alzheimer's disease 1.01	Colorectal cancer 1.04	Hypertensive heart disease 1.54	Other cardiovascular 3.99	Stomach cancer 2.56	COPD 0.85	Breast cancer 0.96
Spain	Total cancers 0.85	Ischemic heart disease 0.39	Stroke 0.53	Alzheimer's disease 0.99	COPD 1.32	Chronic kidney disease 1.32	Lower respiratory infect 0.67	Colorectal cancer 1.04	Hypertensive heart disease 0.97	Diabetes 0.77
Sweden	Total cancers 0.92	Ischemic heart disease 0.89	Stroke 0.81	Alzheimer's disease 0.93	COPD 1.2	Atrial fibrillation 1.83	Lung cancer 1.1	Colorectal cancer 0.86	Lower respiratory infect 0.7	Breast cancer 0.94
Switzerland	Total cancers 0.7	Ischemic heart disease 0.84	Alzheimer's disease 1	Stroke 0.69	Hypertensive heart disease 2.34	Chronic kidney disease 1.54	Falls 1.19	Breast cancer 0.9	Lower respiratory infect 0.61	COPD 0.83
United Kingdom	Total cancers 1.2	Ischemic heart disease 0.63	Stroke 0.81	Lower respiratory infect 2.03	Alzheimer's disease 0.95	COPD 2.14	Lung cancer 1.9	Breast cancer 1.28	Colorectal cancer 0.99	Atrial fibrillation 1.18

**Latin America and Caribbean**	Ischemic heart disease 0.46	Total cancers 0.92	Stroke 0.42	Alzheimer's disease 1	Diabetes 1.13	Lower respiratory infect 1.41	COPD 1.39	Chronic kidney disease 1.43	Hypertensive heart disease 0.73	Colorectal cancer 0.83
**Andean Latin America**	Total cancers 1.2	Ischemic heart disease 0.37	Lower respiratory infect 2.57	Stroke 0.32	Chronic kidney disease 2.01	Alzheimer's disease 0.92	Diabetes 0.84	Stomach cancer 2.42	COPD 0.89	Cirrhosis 1.98
Bolivia (Plurinational State of)	Total cancers 1.85	Ischemic heart disease 0.67	Lower respiratory infect 2.92	Stroke 0.6	Diabetes 1.56	Chronic kidney disease 2.59	COPD 1.59	Stomach cancer 4.01	Alzheimer's disease 1	Cirrhosis 2.38
Ecuador	Total cancers 1.23	Ischemic heart disease 0.43	Stroke 0.38	Chronic kidney disease 2.68	Lower respiratory infect 1.87	Diabetes 1.17	Alzheimer's disease 0.95	Hypertensive heart disease 1.29	COPD 1.17	Cirrhosis 2.39
Peru	Total cancers 1.05	Lower respiratory infect 2.75	Ischemic heart disease 0.29	Alzheimer's disease 0.9	Stroke 0.23	Chronic kidney disease 1.58	Stomach cancer 2.09	Diabetes 0.49	Interstitial lung disease 13.73	Cirrhosis 1.67
**Caribbean**	Ischemic heart disease 0.72	Total cancers 0.98	Stroke 0.7	Diabetes 1.4	Alzheimer's disease 0.86	Lower respiratory infect 1.27	Hypertensive heart disease 1.02	COPD 0.85	Chronic kidney disease 1.05	Falls 2.88
Antigua and Barbuda	Ischemic heart disease 0.77	Total cancers 1.13	Stroke 1.02	Diabetes 3.84	Lower respiratory infect 2.14	Hypertensive heart disease 2.86	Chronic kidney disease 2.52	Alzheimer's disease 0.93	Breast cancer 1.61	Colorectal cancer 1.36
Bahamas	Total cancers 0.87	Ischemic heart disease 0.71	Stroke 0.91	Hypertensive heart disease 5.14	Diabetes 2.81	Alzheimer's disease 0.89	Lower respiratory infect 1.38	Chronic kidney disease 1.83	Breast cancer 1.47	Colorectal cancer 0.92
Barbados	Total cancers 1.32	Stroke 0.98	Ischemic heart disease 0.59	Diabetes 4.13	Lower respiratory infect 2.1	Alzheimer's disease 0.9	Breast cancer 1.87	Colorectal cancer 1.68	Chronic kidney disease 1.62	Decubitus ulcer 52.36
Belize	Ischemic heart disease 0.42	Total cancers 0.81	Diabetes 1.71	Stroke 0.43	Lower respiratory infect 1.66	Alzheimer's disease 0.89	Chronic kidney disease 1.46	Hypertensive heart disease 0.93	COPD 0.54	Other cardiovascular 2.1
Bermuda	Total cancers 0.91	Ischemic heart disease 0.63	Alzheimer's disease 0.92	Stroke 0.49	Colorectal cancer 1.1	Diabetes 1.27	Lower respiratory infect 0.76	Breast cancer 1.17	Lung cancer 1.02	Chronic kidney disease 0.99
Cuba	Ischemic heart disease 0.67	Total cancers 1.13	Stroke 0.53	Lower respiratory infect 1.79	Alzheimer's disease 0.85	Falls 6.8	Colorectal cancer 1.54	COPD 0.94	Lung cancer 2.39	Hypertensive heart disease 0.67
Dominica	Total cancers 1.34	Ischemic heart disease 0.75	Stroke 0.99	Diabetes 3.73	Hypertensive heart disease 3.7	Chronic kidney disease 2.77	Alzheimer's disease 0.92	Lower respiratory infect 1.64	Other cardiovascular 5.06	Breast cancer 1.62
Dominican Republic	Ischemic heart disease 0.87	Stroke 0.69	Total cancers 0.95	Alzheimer's disease 0.88	Diabetes 0.8	Hypertensive heart disease 0.98	Lower respiratory infect 0.8	Chronic kidney disease 0.96	Cirrhosis 1.91	COPD 0.63
Grenada	Stroke 0.93	Total cancers 1.31	Ischemic heart disease 0.6	Diabetes 2.78	Lower respiratory infect 2.76	Chronic kidney disease 2.07	Alzheimer's disease 0.9	Hypertensive heart disease 1.69	Breast cancer 1.55	Colorectal cancer 1.36
Guyana	Ischemic heart disease 0.95	Stroke 1.21	Diabetes 2.53	Total cancers 0.95	Hypertensive heart disease 2.62	Lower respiratory infect 1.96	Chronic kidney disease 1.67	Alzheimer's disease 0.86	Breast cancer 1.12	Colorectal cancer 1.06
Haiti	Ischemic heart disease 1.41	Stroke 1.58	Total cancers 1.23	Diabetes 2.38	Lower respiratory infect 1.27	Hypertensive heart disease 1.46	COPD 0.86	Alzheimer's disease 0.83	Chronic kidney disease 0.98	Breast cancer 1.59
Jamaica	Stroke 0.92	Diabetes 3.61	Ischemic heart disease 0.39	Total cancers 0.94	Alzheimer's disease 0.84	Hypertensive heart disease 1.49	Lower respiratory infect 0.7	Chronic kidney disease 1.09	Breast cancer 1.25	Colorectal cancer 1.01
Puerto Rico	Ischemic heart disease 0.51	Total cancers 0.65	Diabetes 3.32	Alzheimer's disease 0.88	Stroke 0.38	Chronic kidney disease 1.94	COPD 1.25	Lower respiratory infect 0.8	Hypertensive heart disease 1.07	Colorectal cancer 0.74
Saint Kitts and Nevis	Stroke 1.75	Ischemic heart disease 0.77	Total cancers 1.16	Diabetes 2.99	Lower respiratory infect 2.11	Chronic kidney disease 3.1	Alzheimer's disease 0.87	Hypertensive heart disease 1.97	Breast cancer 1.61	Colorectal cancer 1.08
Saint Lucia	Stroke 0.83	Total cancers 0.96	Ischemic heart disease 0.38	Diabetes 2.37	Alzheimer's disease 0.91	Lower respiratory infect 1.32	Hypertensive heart disease 1.5	Chronic kidney disease 1.49	COPD 0.66	Breast cancer 1.06
Saint Vincent and the Grenadines	Ischemic heart disease 0.85	Stroke 0.84	Total cancers 1.25	Diabetes 2.8	Hypertensive heart disease 2.08	Lower respiratory infect 1.52	Alzheimer's disease 0.86	Chronic kidney disease 1.58	Breast cancer 1.66	Other cardiovascular 3.08
Suriname	Stroke 0.8	Ischemic heart disease 0.5	Total cancers 0.93	Diabetes 1.23	Alzheimer's disease 0.91	Chronic kidney disease 1.81	Lower respiratory infect 1.14	Hypertensive heart disease 1.22	COPD 0.64	Colorectal cancer 1.12
Trinidad and Tobago	Ischemic heart disease 0.86	Diabetes 5.05	Stroke 0.9	Total cancers 0.75	Alzheimer's disease 0.86	Chronic kidney disease 1.6	Hypertensive heart disease 1.35	Lower respiratory infect 0.76	Breast cancer 1.05	Colorectal cancer 0.8
United States Virgin Islands	Ischemic heart disease 1.51	Total cancers 1.1	Stroke 0.84	Diabetes 3.18	Alzheimer's disease 0.94	Hypertensive heart disease 2.66	Chronic kidney disease 2.13	Colorectal cancer 1.4	Breast cancer 1.43	Lung cancer 1.11
**Central Latin America**	Ischemic heart disease 0.55	Total cancers 0.86	Stroke 0.34	Diabetes 1.31	Alzheimer's disease 0.99	Chronic kidney disease 1.94	COPD 1.56	Lower respiratory infect 0.77	Hypertensive heart disease 0.67	Cirrhosis 1.47
Colombia	Ischemic heart disease 0.42	Total cancers 0.85	Alzheimer's disease 0.97	COPD 1.7	Stroke 0.27	Chronic kidney disease 0.82	Hypertensive heart disease 0.63	Diabetes 0.45	Lower respiratory infect 0.54	Stomach cancer 1.13
Costa Rica	Total cancers 1.02	Ischemic heart disease 0.4	Alzheimer's disease 1.01	Stroke 0.31	COPD 1.56	Chronic kidney disease 1.37	Hypertensive heart disease 0.84	Colorectal cancer 1.08	Stomach cancer 1.76	Lower respiratory infect 0.54
El Salvador	Ischemic heart disease 0.58	Total cancers 0.93	Alzheimer's disease 0.96	Stroke 0.29	Chronic kidney disease 1.68	Lower respiratory infect 1.09	Diabetes 0.95	COPD 0.87	Stomach cancer 1.49	Cirrhosis 1.15
Guatemala	Ischemic heart disease 0.62	Total cancers 1.08	Lower respiratory infect 2.03	Diabetes 1.48	Stroke 0.38	Chronic kidney disease 1.8	Alzheimer's disease 0.98	Stomach cancer 2.83	COPD 0.68	Cirrhosis 1.42
Honduras	Ischemic heart disease 0.97	Stroke 0.94	Total cancers 1.4	COPD 1.79	Chronic kidney disease 2.07	Cirrhosis 2.57	Alzheimer's disease 1.08	Lung cancer 3.28	Hypertensive heart disease 0.69	Upper digest diseases 3.36
Mexico	Ischemic heart disease 0.59	Total cancers 0.79	Diabetes 1.95	Chronic kidney disease 2.82	Stroke 0.32	COPD 1.87	Alzheimer's disease 1.01	Lower respiratory infect 0.79	Cirrhosis 2.17	Hypertensive heart disease 0.65
Nicaragua	Ischemic heart disease 0.95	Total cancers 1.07	Stroke 0.49	Chronic kidney disease 2.29	Diabetes 1.34	COPD 1.17	Alzheimer's disease 1	Hypertensive heart disease 0.83	Cirrhosis 1.31	Lower respiratory infect 0.49
Panama	Ischemic heart disease 0.36	Total cancers 0.76	Stroke 0.43	Alzheimer's disease 0.98	Diabetes 1.28	COPD 1.13	Chronic kidney disease 1.29	Lower respiratory infect 0.72	Colorectal cancer 0.77	Hypertensive heart disease 0.5
Venezuela (Bolivarian Republic of)	Ischemic heart disease 0.6	Total cancers 0.98	Stroke 0.44	Alzheimer's disease 0.99	Diabetes 1.01	Chronic kidney disease 1.36	Hypertensive heart disease 1.04	COPD 1.04	Lower respiratory infect 0.68	Lung cancer 1.63
**Tropical Latin America**	Total cancers 0.9	Ischemic heart disease 0.35	Stroke 0.45	Lower respiratory infect 1.81	Alzheimer's disease 1.06	Diabetes 0.97	COPD 1.46	Hypertensive heart disease 0.75	Chronic kidney disease 0.9	Urinary diseases 5.26
Brazil	Total cancers 0.9	Ischemic heart disease 0.34	Stroke 0.45	Lower respiratory infect 1.82	Alzheimer's disease 1.06	COPD 1.48	Diabetes 0.95	Hypertensive heart disease 0.74	Chronic kidney disease 0.88	Urinary diseases 5.32
Paraguay	Ischemic heart disease 0.42	Total cancers 0.88	Stroke 0.5	Diabetes 1.56	Alzheimer's disease 1.03	Lower respiratory infect 1.15	Chronic kidney disease 1.44	Hypertensive heart disease 0.94	COPD 0.67	Colorectal cancer 0.9
**North Africa and Middle East**	Ischemic heart disease 1.47	Stroke 1.01	Total cancers 0.75	Hypertensive heart disease 2.31	Alzheimer's disease 1.13	Chronic kidney disease 2.05	Diabetes 1.13	Cirrhosis 3.1	COPD 1.21	Lower respiratory infect 0.92
**North Africa and Middle East**	Ischemic heart disease 1.47	Stroke 1.01	Total cancers 0.75	Hypertensive heart disease 2.31	Alzheimer's disease 1.13	Chronic kidney disease 2.05	Diabetes 1.13	Cirrhosis 3.1	COPD 1.21	Lower respiratory infect 0.92
Afghanistan	Ischemic heart disease 2.25	Stroke 1.41	Total cancers 1.57	Conflict & terror 2868.46	Hypertensive heart disease 2.4	Diabetes 1.52	Chronic kidney disease 1.97	COPD 1.25	Lower respiratory infect 0.68	Alzheimer's disease 1.25
Algeria	Ischemic heart disease 1.62	Stroke 1.12	Total cancers 0.63	Hypertensive heart disease 2.6	Chronic kidney disease 2.31	Alzheimer's disease 1.19	Diabetes 0.76	Lower respiratory infect 1.23	COPD 1.07	Cirrhosis 1.43
Bahrain	Ischemic heart disease 1.52	Diabetes 9.01	Total cancers 1.02	Stroke 0.93	Chronic kidney disease 3.47	COPD 2.01	Alzheimer's disease 1.27	Lower respiratory infect 1.44	Breast cancer 1.46	Cirrhosis 3
Egypt	Ischemic heart disease 2.79	Cirrhosis 19.78	Stroke 1.04	Hypertensive heart disease 3.83	Total burden related to hep 22.35	Chronic kidney disease 3.8	Total cancers 0.68	Diabetes 1.29	Total burden related to hep 20.47	Lower respiratory infect 1.74
Iran (Islamic Republic of)	Ischemic heart disease 1.14	Stroke 0.78	Total cancers 0.79	Hypertensive heart disease 2.32	Alzheimer's disease 1.12	Diabetes 1.02	Chronic kidney disease 1.42	COPD 1.16	Lower respiratory infect 0.85	Stomach cancer 1.53
Iraq	Ischemic heart disease 1.44	Stroke 1.35	Total cancers 0.67	Diabetes 1.56	Chronic kidney disease 2.3	Alzheimer's disease 1.09	Hypertensive heart disease 1.3	Breast cancer 0.69	Lower respiratory infect 0.43	Lung cancer 0.96
Jordan	Ischemic heart disease 1	Stroke 1.24	Total cancers 0.72	Hypertensive heart disease 4.49	Diabetes 2.5	Chronic kidney disease 3.45	Alzheimer's disease 1.11	Lower respiratory infect 1.03	Breast cancer 0.9	Colorectal cancer 0.8
Kuwait	Ischemic heart disease 0.74	Total cancers 0.5	Stroke 0.79	Alzheimer's disease 1.1	Lower respiratory infect 1.81	Hypertensive heart disease 2.98	Diabetes 2.01	Chronic kidney disease 1.82	Breast cancer 0.61	Colorectal cancer 0.49
Lebanon	Ischemic heart disease 1.41	Total cancers 1.1	Stroke 0.46	Alzheimer's disease 1.21	Hypertensive heart disease 2.12	Chronic kidney disease 1.8	Breast cancer 1.63	Colorectal cancer 1.16	Lower respiratory infect 0.76	COPD 0.81
Libya	Ischemic heart disease 1.08	Stroke 0.82	Total cancers 0.66	Alzheimer's disease 1.26	Chronic kidney disease 2.32	Hypertensive heart disease 1.92	Diabetes 0.85	Lower respiratory infect 0.85	COPD 0.8	Cirrhosis 1.82
Morocco	Ischemic heart disease 1.61	Stroke 1.07	Hypertensive heart disease 1.81	Total cancers 0.62	Chronic kidney disease 1.71	Alzheimer's disease 1.03	Diabetes 0.7	Lower respiratory infect 0.67	COPD 0.68	Tuberculosis 3.29
Oman	Ischemic heart disease 3.61	Stroke 1.89	Total cancers 0.82	Diabetes 4.62	Lower respiratory infect 2.77	Hypertensive heart disease 3.69	Alzheimer's disease 1.35	Chronic kidney disease 2.25	COPD 1.46	Other cardiovascular 3.93
Palestine	Ischemic heart disease 1.17	Stroke 1.22	Total cancers 1.13	Diabetes 2.28	Hypertensive heart disease 2.06	Alzheimer's disease 1.07	Chronic kidney disease 1.66	Lower respiratory infect 0.96	Cirrhosis 1.6	Colorectal cancer 1.51
Qatar	Ischemic heart disease 4.99	Diabetes 17.7	Total cancers 1.57	Chronic kidney disease 10.31	Stroke 1.75	Cirrhosis 7.64	Lower respiratory infect 3.4	Breast cancer 2.05	Colorectal cancer 1.77	COPD 1.73
Saudi Arabia	Ischemic heart disease 1.74	Stroke 1.82	Chronic kidney disease 5.17	Total cancers 0.56	Alzheimer's disease 1.19	Lower respiratory infect 1.68	Cirrhosis 5.88	COPD 1.34	Diabetes 1.39	Total burden related to hep 6.22
Sudan	Ischemic heart disease 1.65	Stroke 1.23	Hypertensive heart disease 1.87	Total cancers 0.69	Alzheimer's disease 0.98	Chronic kidney disease 1.22	Lower respiratory infect 0.64	COPD 0.67	Diabetes 0.48	Cirrhosis 1.18
Syrian Arab Republic	Ischemic heart disease 2.21	Stroke 0.98	Conflict & terror 4346.82	Total cancers 0.66	Chronic kidney disease 1.69	Alzheimer's disease 1.18	Diabetes 0.63	COPD 1.06	Lower respiratory infect 0.99	Cirrhosis 1.99
Tunisia	Ischemic heart disease 1.14	Stroke 0.84	Total cancers 0.56	Alzheimer's disease 1.14	Hypertensive heart disease 1.89	Chronic kidney disease 1.64	Lower respiratory infect 0.79	Diabetes 0.58	COPD 0.78	Colorectal cancer 0.71
Turkey	Ischemic heart disease 1	Stroke 0.95	Total cancers 0.79	Alzheimer's disease 1.17	Hypertensive heart disease 2.52	COPD 1.83	Diabetes 1.71	Chronic kidney disease 2.08	Lower respiratory infect 0.98	Colorectal cancer 0.79
United Arab Emirates	Ischemic heart disease 2.61	Total cancers 0.87	Stroke 2.53	Diabetes 7.35	Chronic kidney disease 7.71	Lower respiratory infect 4.46	Hypertensive heart disease 7.92	Alzheimer's disease 1.27	COPD 1.61	Colorectal cancer 0.87
Yemen	Ischemic heart disease 1.9	Stroke 1.21	Hypertensive heart disease 2.06	Total cancers 0.74	Alzheimer's disease 1.05	COPD 0.88	Lower respiratory infect 0.56	Chronic kidney disease 0.9	Asthma 1.22	Diabetes 0.48
**South Asia**	Ischemic heart disease 0.72	COPD 3.62	Diarrheal diseases 14.84	Stroke 0.61	Total cancers 0.75	Lower respiratory infect 1.1	Diabetes 0.77	Falls 5.51	Alzheimer's disease 0.79	Asthma 2.8
**South Asia**	Ischemic heart disease 0.72	COPD 3.62	Diarrheal diseases 14.84	Stroke 0.61	Total cancers 0.75	Lower respiratory infect 1.1	Diabetes 0.77	Falls 5.51	Alzheimer's disease 0.79	Asthma 2.8
Bangladesh	Stroke 1.24	Ischemic heart disease 0.64	Total cancers 0.65	Diarrheal diseases 3.21	COPD 1.3	Diabetes 1	Lower respiratory infect 0.59	Alzheimer's disease 0.73	Hypertensive heart disease 0.7	Tuberculosis 1.26
Bhutan	COPD 4.14	Ischemic heart disease 0.81	Stroke 0.63	Total cancers 0.86	Diabetes 1.02	Lower respiratory infect 0.69	Alzheimer's disease 0.92	Diarrheal diseases 1.7	Chronic kidney disease 1.09	Falls 3.91
India	Ischemic heart disease 0.71	COPD 4.05	Diarrheal diseases 24.57	Stroke 0.52	Total cancers 0.71	Lower respiratory infect 1.28	Falls 6.85	Diabetes 0.7	Alzheimer's disease 0.8	Asthma 3.37
Nepal	COPD 6.24	Ischemic heart disease 0.58	Stroke 0.59	Total cancers 0.84	Diarrheal diseases 2.13	Asthma 3.27	Lower respiratory infect 0.78	Alzheimer's disease 0.86	Diabetes 0.68	Chronic kidney disease 0.89
Pakistan	Ischemic heart disease 1	Stroke 1.07	Total cancers 1.43	Diarrheal diseases 3.8	COPD 1.75	Diabetes 1.59	Hypertensive heart disease 0.98	Cirrhosis 1.91	Chronic kidney disease 1.24	Breast cancer 3.05
**Southeast Asia, East Asia, and Oceania**	Stroke 1.18	Ischemic heart disease 0.72	Total cancers 0.94	COPD 3.19	Alzheimer's disease 1.04	Hypertensive heart disease 1.35	Lung cancer 2.37	Lower respiratory infect 0.88	Diabetes 0.6	Chronic kidney disease 0.85
**East Asia**	Stroke 1.17	Ischemic heart disease 0.75	Total cancers 0.96	COPD 3.72	Alzheimer's disease 1.03	Hypertensive heart disease 1.4	Lung cancer 2.58	Lower respiratory infect 0.62	Stomach cancer 1.62	Chronic kidney disease 0.7
China	Stroke 1.17	Ischemic heart disease 0.76	Total cancers 0.97	COPD 3.76	Alzheimer's disease 1.04	Hypertensive heart disease 1.41	Lung cancer 2.65	Stomach cancer 1.64	Lower respiratory infect 0.58	Chronic kidney disease 0.67
Democratic People's Republic of Korea	Stroke 1.32	Ischemic heart disease 0.67	COPD 3.89	Total cancers 0.81	Alzheimer's disease 0.94	Lower respiratory infect 0.86	Hypertensive heart disease 0.97	Lung cancer 1.79	Diabetes 0.32	Stomach cancer 1.24
Taiwan (Province of China)	Total cancers 0.88	Ischemic heart disease 0.45	Stroke 0.67	Alzheimer's disease 0.97	Lower respiratory infect 1.78	Diabetes 3.34	Chronic kidney disease 2.47	Colorectal cancer 1.17	Lung cancer 1.32	COPD 1.12
**Oceania**	Ischemic heart disease 1.06	Stroke 1	COPD 3.64	Diabetes 2.73	Total cancers 0.95	Asthma 5.11	Diarrheal diseases 2.49	Lower respiratory infect 0.97	Alzheimer's disease 0.93	Hypertensive heart disease 0.96
American Samoa	Ischemic heart disease 0.91	Total cancers 1.22	Stroke 1.09	Diabetes 4.03	Chronic kidney disease 3.18	Alzheimer's disease 1.08	COPD 1.95	Lower respiratory infect 1.36	Breast cancer 1.72	Lung cancer 1.58
Cook Islands	Diabetes 6.59	Ischemic heart disease 0.69	Total cancers 0.83	Stroke 0.8	Hypertensive heart disease 4.97	Lower respiratory infect 1.99	Alzheimer's disease 0.98	Breast cancer 2.2	COPD 1.59	Chronic kidney disease 1.79
Fiji	Diabetes 7.46	Ischemic heart disease 1.1	Stroke 1	Total cancers 0.99	Asthma 10.56	Chronic kidney disease 1.94	Hypertensive heart disease 1.61	Alzheimer's disease 1.01	Breast cancer 1.87	Lower respiratory infect 1.19
Guam	Ischemic heart disease 1.2	Total cancers 0.6	Stroke 0.61	Alzheimer's disease 0.89	Chronic kidney disease 1.93	Diabetes 1.51	COPD 0.91	Lower respiratory infect 0.69	Lung cancer 1.19	Hypertensive heart disease 0.91
Kiribati	Ischemic heart disease 1.18	Stroke 1.39	Diabetes 3.78	Total cancers 1.58	Diarrheal diseases 14.59	Asthma 9.48	Tuberculosis 9.8	Chronic kidney disease 1.92	Lower respiratory infect 1.37	Cervical cancer 6.04
Marshall Islands	Ischemic heart disease 1.49	Stroke 1.43	Diabetes 3.31	Total cancers 1.35	COPD 1.97	Lower respiratory infect 1.7	Hypertensive heart disease 1.69	Chronic kidney disease 1.83	Diarrheal diseases 6	Asthma 3.5
Micronesia (Federated States of)	Ischemic heart disease 1.36	Stroke 1.37	Diabetes 4.05	Total cancers 1.43	COPD 2.52	Chronic kidney disease 2.77	Lower respiratory infect 2.12	Hypertensive heart disease 1.78	Alzheimer's disease 1.17	Asthma 5.03
Nauru	Ischemic heart disease 1.72	Stroke 1.61	Diabetes 4.14	Total cancers 1.43	COPD 2.72	Chronic kidney disease 2.47	Lower respiratory infect 2.35	Hypertensive heart disease 1.62	Breast cancer 2.08	Alzheimer's disease 1.14
Niue	Ischemic heart disease 1.18	Diabetes 4.95	Stroke 1.06	Total cancers 1.05	Alzheimer's disease 1.14	Lower respiratory infect 1.75	COPD 1.98	Chronic kidney disease 2.37	Hypertensive heart disease 1.31	Breast cancer 1.53
Northern Mariana Islands	Ischemic heart disease 1.11	Total cancers 1.14	Stroke 1.22	Diabetes 3.31	Chronic kidney disease 4.01	Alzheimer's disease 1.09	COPD 1.53	Lower respiratory infect 1.25	Lung cancer 1.69	Breast cancer 1.43
Palau	Ischemic heart disease 1.43	Total cancers 1.63	Diabetes 5.89	Lower respiratory infect 6.42	Stroke 1.06	COPD 4.47	Chronic kidney disease 3.35	Lung cancer 3.61	Alzheimer's disease 0.94	Breast cancer 1.72
Papua New Guinea	COPD 4.45	Ischemic heart disease 0.96	Stroke 0.9	Asthma 5.05	Total cancers 0.91	Diabetes 1.79	Diarrheal diseases 2.05	Lower respiratory infect 0.78	Falls 5.13	Hypertensive heart disease 0.86

Samoa	Ischemic heart disease 1.13	Stroke 1.08	Diabetes 2.55	Total cancers 0.88	COPD 2.66	Lower respiratory infect 2.08	Alzheimer's disease 1.23	Chronic kidney disease 1.93	Hypertensive heart disease 1.33	Asthma 6.41
Solomon Islands	Stroke 2.61	Ischemic heart disease 2.08	Lower respiratory infect 2.66	Diarrheal diseases 5.39	Total cancers 1.42	Diabetes 2.91	COPD 2.13	Asthma 2.48	Hypertensive heart disease 1.08	Falls 5.75
Tokelau	Ischemic heart disease 1.13	Stroke 1.04	Diabetes 3.29	Total cancers 1.22	COPD 2.3	Lower respiratory infect 1.9	Alzheimer's disease 1.14	Chronic kidney disease 1.92	Hypertensive heart disease 1.23	Asthma 5.06
Tonga	Ischemic heart disease 0.51	Diabetes 2.88	Stroke 0.67	Total cancers 1.12	Alzheimer's disease 1.09	Lower respiratory infect 1.58	Chronic kidney disease 1.61	COPD 1.1	Breast cancer 1.58	Asthma 2.98
Tuvalu	Ischemic heart disease 1.28	Stroke 1.21	Diabetes 3.15	Total cancers 1.2	COPD 2.22	Lower respiratory infect 1.84	Chronic kidney disease 1.72	Alzheimer's disease 1.14	Hypertensive heart disease 1.32	Asthma 4.4
Vanuatu	Ischemic heart disease 1.69	Stroke 1.46	Total cancers 1.05	Diabetes 2.16	COPD 1.73	Diarrheal diseases 4.4	Lower respiratory infect 1.23	Chronic kidney disease 1.46	Asthma 2.86	Hypertensive heart disease 1.03
**Southeast Asia**	Stroke 1.25	Ischemic heart disease 0.61	Total cancers 0.87	Lower respiratory infect 1.74	Diabetes 1.19	Alzheimer's disease 1.07	COPD 1.43	Hypertensive heart disease 1.21	Chronic kidney disease 1.37	Cirrhosis 2.11
Cambodia	Stroke 1.39	Ischemic heart disease 0.7	Lower respiratory infect 2.54	Total cancers 0.97	Cirrhosis 2.73	Alzheimer's disease 1.09	Diabetes 0.92	COPD 0.92	Hypertensive heart disease 0.96	Tuberculosis 2.58
Indonesia	Stroke 1.91	Ischemic heart disease 0.77	Total cancers 0.89	Diabetes 1.29	Hypertensive heart disease 1.99	Diarrheal diseases 41.01	Cirrhosis 4.53	COPD 1.88	Alzheimer's disease 1.03	Lower respiratory infect 1.26
Lao People's Democratic Republic	Stroke 1.41	Ischemic heart disease 1.01	Total cancers 0.93	Lower respiratory infect 1.59	Diabetes 1.18	COPD 1.19	Hypertensive heart disease 1.14	Chronic kidney disease 1.43	Alzheimer's disease 0.97	Diarrheal diseases 1.78
Malaysia	Ischemic heart disease 1.1	Lower respiratory infect 6.1	Stroke 1.26	Total cancers 1.02	Alzheimer's disease 1.02	Chronic kidney disease 2.18	COPD 1.42	Colorectal cancer 1.39	Urinary diseases 6.68	Diabetes 0.86
Maldives	Ischemic heart disease 0.68	Total cancers 0.88	Stroke 0.5	COPD 2.02	Chronic kidney disease 1.95	Alzheimer's disease 1.01	Diabetes 0.56	Lower respiratory infect 0.57	Hypertensive heart disease 0.46	Colorectal cancer 1.22
Mauritius	Ischemic heart disease 0.64	Diabetes 4.34	Stroke 0.64	Total cancers 0.67	Chronic kidney disease 3.74	Alzheimer's disease 1	Hypertensive heart disease 2.13	Lower respiratory infect 0.84	COPD 0.82	Breast cancer 0.89
Myanmar	Stroke 1.78	Ischemic heart disease 0.64	Total cancers 0.99	COPD 2.18	Diabetes 1.29	Lower respiratory infect 1	Alzheimer's disease 0.99	Hypertensive heart disease 0.93	Asthma 2.39	Chronic kidney disease 0.91
Philippines	Ischemic heart disease 0.75	Stroke 0.81	Lower respiratory infect 4.17	Total cancers 0.78	Hypertensive heart disease 1.75	Diabetes 1.03	Chronic kidney disease 1.82	Alzheimer's disease 1.02	COPD 1.06	Tuberculosis 9.7
Seychelles	Ischemic heart disease 0.74	Total cancers 1.15	Lower respiratory infect 3.79	Stroke 0.78	Hypertensive heart disease 4.05	Chronic kidney disease 3.23	Alzheimer's disease 1.03	Diabetes 1.39	COPD 1.23	Colorectal cancer 1.62
Sri Lanka	Ischemic heart disease 0.69	Stroke 0.74	Diabetes 2.57	Total cancers 0.66	Asthma 17.02	Alzheimer's disease 1.07	Chronic kidney disease 1.45	Hypertensive heart disease 1.26	Lower respiratory infect 1.02	COPD 1.13
Thailand	Total cancers 0.87	Ischemic heart disease 0.32	Stroke 0.47	Alzheimer's disease 1.02	Lower respiratory infect 1.42	Chronic kidney disease 1.87	Diabetes 0.77	COPD 0.77	Lung cancer 1.69	Liver cancer 5.2
Timor-Leste	Stroke 1.5	Ischemic heart disease 0.99	Total cancers 0.91	Lower respiratory infect 1.92	COPD 1.38	Hypertensive heart disease 1.32	Diabetes 0.83	Alzheimer's disease 1.03	Chronic kidney disease 1.19	Tuberculosis 3.05
Viet Nam	Stroke 1.23	Ischemic heart disease 0.44	Total cancers 0.86	Alzheimer's disease 1.27	Diabetes 1.23	COPD 1.41	Chronic kidney disease 1.55	Lower respiratory infect 1.06	Hypertensive heart disease 1.11	Falls 5.03
**Sub-Saharan Africa**	Stroke 0.94	Ischemic heart disease 0.74	Total cancers 1.01	Lower respiratory infect 1.58	Hypertensive heart disease 1.41	Diarrheal diseases 1.59	Diabetes 1.18	Alzheimer's disease 1.09	Tuberculosis 1.88	COPD 0.8
**Central Sub-Saharan Africa**	Ischemic heart disease 0.91	Stroke 0.99	Lower respiratory infect 1.68	Hypertensive heart disease 2.18	Total cancers 0.91	Tuberculosis 2.64	COPD 1.4	Diarrheal diseases 1.43	Alzheimer's disease 1.11	Diabetes 0.81
Angola	Ischemic heart disease 0.83	Stroke 0.98	Total cancers 0.89	Lower respiratory infect 1.55	Hypertensive heart disease 2.04	Diarrheal diseases 3.23	COPD 0.98	Alzheimer's disease 1.12	Tuberculosis 3.06	Diabetes 0.86
Central African Republic	Stroke 1.17	Ischemic heart disease 1.15	Diarrheal diseases 2.11	Tuberculosis 2.25	Lower respiratory infect 1.33	Hypertensive heart disease 2.14	Total cancers 0.92	COPD 1.5	Asthma 1.78	Diabetes 1.04
Congo	Ischemic heart disease 0.98	Stroke 1.15	Total cancers 1.09	Hypertensive heart disease 3.12	Lower respiratory infect 2.83	Diabetes 1.2	COPD 1.35	Alzheimer's disease 1.17	Tuberculosis 9.2	Diarrheal diseases 8.71
Democratic Republic of the Congo	Ischemic heart disease 0.92	Stroke 0.97	Lower respiratory infect 1.66	Hypertensive heart disease 2.14	Total cancers 0.9	Tuberculosis 2.52	COPD 1.5	Alzheimer's disease 1.1	Diarrheal diseases 1.06	Asthma 1.74
Equatorial Guinea	Ischemic heart disease 0.8	Stroke 1.08	Total cancers 1.06	Lower respiratory infect 3.74	Hypertensive heart disease 3.63	Diabetes 1.78	Alzheimer's disease 1.21	COPD 1.78	Chronic kidney disease 1.98	Breast cancer 1.54
Gabon	Ischemic heart disease 0.74	Stroke 0.87	Total cancers 1.08	Hypertensive heart disease 3.39	Lower respiratory infect 2.84	Diabetes 1.6	Alzheimer's disease 1.24	Chronic kidney disease 2.32	COPD 1.31	Breast cancer 1.62
**Eastern Sub-Saharan Africa**	Stroke 0.96	Ischemic heart disease 0.65	Lower respiratory infect 1.39	Total cancers 1.04	Hypertensive heart disease 1.56	Diarrheal diseases 1.2	Tuberculosis 1.7	Alzheimer's disease 1.13	Diabetes 0.94	Cirrhosis 1.47
Burundi	Stroke 0.98	Ischemic heart disease 0.86	Lower respiratory infect 1.29	Tuberculosis 1.89	Total cancers 0.98	Diarrheal diseases 0.96	Hypertensive heart disease 1.26	Alzheimer's disease 1.06	COPD 1	Diabetes 0.98
Comoros	Stroke 1.01	Ischemic heart disease 0.77	Lower respiratory infect 1.77	Total cancers 1.03	Hypertensive heart disease 1.77	Alzheimer's disease 1.14	Tuberculosis 3.22	Diabetes 1.02	Diarrheal diseases 1.92	Chronic kidney disease 1.07
Djibouti	Stroke 0.98	Ischemic heart disease 0.79	Total cancers 1.11	Lower respiratory infect 1.7	Hypertensive heart disease 1.76	Diabetes 1.08	Alzheimer's disease 1.18	Tuberculosis 2.69	Diarrheal diseases 1.66	Chronic kidney disease 1.07
Eritrea	Stroke 1.09	Lower respiratory infect 2.27	Ischemic heart disease 0.77	Total cancers 1.19	Hypertensive heart disease 2.22	Tuberculosis 3.37	Diarrheal diseases 2.3	Diabetes 1.23	Cirrhosis 1.84	Alzheimer's disease 1.16
Ethiopia	Stroke 0.79	Ischemic heart disease 0.71	Lower respiratory infect 1.17	Total cancers 0.94	Diarrheal diseases 1.25	Hypertensive heart disease 1.27	Alzheimer's disease 1.21	Tuberculosis 1.32	Cirrhosis 1.72	Diabetes 0.93
Kenya	Stroke 0.88	Ischemic heart disease 0.54	Lower respiratory infect 2.18	Total cancers 0.96	Hypertensive heart disease 1.6	Diarrheal diseases 3.79	Cirrhosis 2.62	Alzheimer's disease 1.11	Tuberculosis 4.32	Diabetes 0.73
Madagascar	Stroke 1.46	Ischemic heart disease 0.82	Hypertensive heart disease 2.67	Lower respiratory infect 1.62	Total cancers 0.85	Diarrheal diseases 1.85	Tuberculosis 2.58	COPD 1.02	Diabetes 0.83	Alzheimer's disease 0.85
Malawi	Stroke 0.87	Ischemic heart disease 0.59	Total cancers 1.18	Lower respiratory infect 1.32	Diarrheal diseases 1.71	Hypertensive heart disease 1.39	Tuberculosis 2.18	Alzheimer's disease 1.13	Diabetes 0.91	Cirrhosis 1.41
Mozambique	Stroke 1.2	Ischemic heart disease 0.66	Total cancers 1.16	Lower respiratory infect 1.05	Hypertensive heart disease 1.7	Tuberculosis 1.87	Alzheimer's disease 1.23	Diabetes 0.98	Malaria 5.31	Diarrheal diseases 0.44
Rwanda	Stroke 1.02	Ischemic heart disease 0.65	Total cancers 1.08	Lower respiratory infect 1.54	Hypertensive heart disease 1.48	Alzheimer's disease 1.16	Diabetes 0.99	Tuberculosis 2.26	COPD 0.91	Diarrheal diseases 1.38
Somalia	Stroke 0.97	Tuberculosis 0.63	Ischemic heart disease 1.02	Diarrheal diseases 0.36	Lower respiratory infect 0.73	Hypertensive heart disease 1.19	Total cancers 1.02	Diabetes 1.25	COPD 0.94	Cirrhosis 0.87
South Sudan	Stroke 0.85	Ischemic heart disease 0.64	Diarrheal diseases 2.25	Lower respiratory infect 1.23	Total cancers 0.97	Hypertensive heart disease 1.37	Tuberculosis 2.13	Alzheimer's disease 1	Diabetes 0.94	Cirrhosis 1.36
Uganda	Stroke 0.88	Ischemic heart disease 0.63	Total cancers 1.22	Lower respiratory infect 1.52	Hypertensive heart disease 1.33	Tuberculosis 2.33	Alzheimer's disease 1.1	Diabetes 1	Diarrheal diseases 1.3	COPD 0.66
United Republic of Tanzania	Stroke 1	Ischemic heart disease 0.59	Total cancers 1.12	Lower respiratory infect 1.57	Hypertensive heart disease 1.73	Diarrheal diseases 1.85	Alzheimer's disease 1.14	Tuberculosis 2.51	Diabetes 1	Chronic kidney disease 1.14
Zambia	Stroke 1.38	Total cancers 1.09	Hypertensive heart disease 2.71	Ischemic heart disease 0.49	Lower respiratory infect 1.83	Diarrheal diseases 3.48	Diabetes 0.93	Tuberculosis 4.28	Alzheimer's disease 1.08	Cirrhosis 2.13
**Southern Sub-Saharan Africa**	Stroke 0.9	Ischemic heart disease 0.57	Total cancers 1.07	Diabetes 2.51	Lower respiratory infect 2.48	Hypertensive heart disease 2.19	Alzheimer's disease 1	COPD 1.36	Chronic kidney disease 1.52	Diarrheal diseases 10.28
Botswana	Stroke 1.19	Ischemic heart disease 0.69	Total cancers 1.28	Diabetes 2.72	Lower respiratory infect 3.42	Hypertensive heart disease 2.37	Alzheimer's disease 1.08	COPD 1.6	Chronic kidney disease 1.55	Breast cancer 1.64
Eswatini	Stroke 1.15	Diabetes 3.22	Ischemic heart disease 0.66	Total cancers 1.25	Lower respiratory infect 2.34	Hypertensive heart disease 2.15	Diarrheal diseases 11.88	Chronic kidney disease 1.47	COPD 1.25	Alzheimer's disease 0.95
Lesotho	Stroke 1.56	Ischemic heart disease 0.75	Diabetes 3	Total cancers 1.39	Lower respiratory infect 2.21	Hypertensive heart disease 2.45	Diarrheal diseases 6.24	COPD 1.92	HIV/AIDS 436.75	Tuberculosis 5.35
Namibia	Stroke 1.11	Ischemic heart disease 0.6	Total cancers 0.91	Lower respiratory infect 2.82	Diabetes 1.78	Hypertensive heart disease 2.31	Alzheimer's disease 1.08	COPD 1.59	Diarrheal diseases 14.45	Chronic kidney disease 1.03
South Africa	Stroke 0.86	Total cancers 0.99	Ischemic heart disease 0.49	Diabetes 2.63	Lower respiratory infect 2.43	Hypertensive heart disease 2.42	Alzheimer's disease 0.99	COPD 1.47	Chronic kidney disease 1.61	Diarrheal diseases 27.4
Zimbabwe	Ischemic heart disease 1.15	Total cancers 1.74	Stroke 0.9	Lower respiratory infect 2.61	Diabetes 1.7	Alzheimer's disease 1.06	Tuberculosis 3.07	Hypertensive heart disease 0.92	Chronic kidney disease 1.16	Diarrheal diseases 1.81
**Western Sub-Saharan Africa**	Ischemic heart disease 0.86	Stroke 0.91	Lower respiratory infect 1.55	Total cancers 0.97	Diarrheal diseases 1.78	Malaria 19.44	Diabetes 1.05	Alzheimer's disease 1.1	Tuberculosis 1.65	Hypertensive heart disease 0.83
Benin	Stroke 0.99	Ischemic heart disease 0.88	Lower respiratory infect 1.43	Total cancers 1	Malaria 10.79	Diarrheal diseases 1.09	Diabetes 1.09	Alzheimer's disease 1.09	Hypertensive heart disease 0.75	Chronic kidney disease 1.14
Burkina Faso	Ischemic heart disease 1.03	Stroke 0.64	Lower respiratory infect 1.18	Total cancers 1.11	Diarrheal diseases 0.8	Malaria 6.27	Hypertensive heart disease 0.9	Alzheimer's disease 1.15	Tuberculosis 0.71	Diabetes 0.83
Cabo Verde	Ischemic heart disease 0.91	Total cancers 1.53	Stroke 0.62	Lower respiratory infect 1.66	Alzheimer's disease 1	Diabetes 1.2	Chronic kidney disease 0.92	Hypertensive heart disease 0.68	Stomach cancer 2.27	COPD 0.52
Cameroon	Ischemic heart disease 0.74	Stroke 0.9	Total cancers 1.09	Lower respiratory infect 2.06	Diabetes 1.42	Malaria 417.05	Chronic kidney disease 1.58	Alzheimer's disease 1.1	Diarrheal diseases 2.41	Tuberculosis 2.87
Chad	Ischemic heart disease 1.01	Stroke 0.87	Lower respiratory infect 1.27	Diarrheal diseases 0.93	Total cancers 1.08	Tuberculosis 0.92	Diabetes 1.19	Alzheimer's disease 1.05	Hypertensive heart disease 0.74	Cirrhosis 1.07
Côte d'Ivoire	Ischemic heart disease 0.84	Stroke 0.8	Lower respiratory infect 1.65	Total cancers 1.02	Malaria 55.76	Diabetes 1.2	Alzheimer's disease 1.06	Diarrheal diseases 1.3	Tuberculosis 1.8	Chronic kidney disease 1.18
Gambia	Ischemic heart disease 1.11	Stroke 0.96	Lower respiratory infect 2.02	Total cancers 0.87	Diabetes 1.2	Tuberculosis 2.32	Alzheimer's disease 1.13	Hypertensive heart disease 0.93	Diarrheal diseases 1.16	Chronic kidney disease 1.22
Ghana	Stroke 1.27	Ischemic heart disease 0.87	Total cancers 0.99	Lower respiratory infect 1.97	Diabetes 0.94	Chronic kidney disease 1.36	Alzheimer's disease 0.95	Malaria 981.74	Hypertensive heart disease 1.02	Diarrheal diseases 4.73
Guinea	Ischemic heart disease 0.98	Stroke 0.96	Lower respiratory infect 1.74	Total cancers 1.19	Diarrheal diseases 1.22	Diabetes 1.32	Alzheimer's disease 1.11	Tuberculosis 1.28	Hypertensive heart disease 0.89	Chronic kidney disease 1.2
Guinea-Bissau	Ischemic heart disease 1.14	Stroke 1.02	Lower respiratory infect 1.88	Total cancers 1.15	Diarrheal diseases 2.11	Diabetes 1.44	Tuberculosis 1.64	Hypertensive heart disease 0.97	Alzheimer's disease 1.06	Chronic kidney disease 1.23
Liberia	Ischemic heart disease 0.95	Stroke 0.83	Lower respiratory infect 1.44	Total cancers 1.02	Malaria 18.86	Diarrheal diseases 1.47	Diabetes 1.21	Alzheimer's disease 1.07	Chronic kidney disease 1.34	Tuberculosis 1.42
Mali	Ischemic heart disease 1.12	Stroke 0.93	Diarrheal diseases 1	Total cancers 0.97	Protein-energy malnutrition 4.16	Diabetes 1.39	Lower respiratory infect 0.61	Tuberculosis 0.81	Alzheimer's disease 1.16	Hypertensive heart disease 0.76
Mauritania	Ischemic heart disease 0.86	Stroke 0.82	Lower respiratory infect 2.09	Total cancers 1.08	Diabetes 1.26	Malaria 430.62	Alzheimer's disease 1.1	Chronic kidney disease 1.51	Diarrheal diseases 2.38	Hypertensive heart disease 0.82
Niger	Ischemic heart disease 1.13	Stroke 0.85	Lower respiratory infect 0.94	Diarrheal diseases 0.45	Total cancers 0.97	Malaria 2.46	Diabetes 1.18	Tuberculosis 0.36	Alzheimer's disease 1.11	COPD 0.83
Nigeria	Ischemic heart disease 0.78	Stroke 0.9	Total cancers 0.87	Lower respiratory infect 1.8	Diarrheal diseases 5.62	Malaria 850.47	Alzheimer's disease 1.12	Diabetes 0.92	Tuberculosis 4.49	Cirrhosis 1.83
Sao Tome and Principe	Ischemic heart disease 1.08	Stroke 1.14	Lower respiratory infect 2.8	Total cancers 1.26	Chronic kidney disease 2.26	COPD 1.43	Alzheimer's disease 1	Other cardiovascular 5.95	Asthma 2.55	Cirrhosis 1.5
Senegal	Ischemic heart disease 0.93	Stroke 0.81	Total cancers 1.08	Lower respiratory infect 1.33	Diabetes 1.31	Alzheimer's disease 1.15	Diarrheal diseases 1.29	Tuberculosis 1.93	Chronic kidney disease 1.3	Malaria 15.95
Sierra Leone	Ischemic heart disease 1.1	Stroke 0.96	Lower respiratory infect 1.64	Total cancers 1.02	Malaria 14.27	Diarrheal diseases 1.26	Diabetes 1.19	Tuberculosis 1.43	Alzheimer's disease 1.05	Hypertensive heart disease 0.69
Togo	Ischemic heart disease 0.91	Stroke 0.85	Lower respiratory infect 1.88	Total cancers 0.99	Diarrheal diseases 1.81	Malaria 43.43	Diabetes 0.98	Alzheimer's disease 1.07	Tuberculosis 1.94	Hypertensive heart disease 0.8

Values shown in brackets represent the ratio of observed deaths to predicted deaths on the basis of Socio-Demographic Index (SDI), rounded to two (2) digits. Color ranges (shown below) were calculated to place a roughly equal number of cells into each bin.

COLOR KEY:	[0.00,0.68)	[0.68,0.83)	[0.83,0.93)	[0.93,1.02)	[1.02,1.13)	[1.13,1.28)	[1.28,1.55)	[1.55,1.94)	[1.94,2.83)	[2.83,...)

Figure S22. Leading ten causes of total years lived with disability with ratio of observed to expected years lived with disability in 2019, among people 70 years and older, in males, for all GBD locations.

Causes are ranked based on global estimates of YLDs. Causes are color coded (shown in color key) based on observed to expected (O:E) rate ratio on the basis of SDI from the epidemiologic transition analysis. Shades of blue represent much lower observed deaths and YLDs than expected levels based on SDI, whereas red shows that observed deaths and YLDs exceed expected levels. When the ratio exceeds 1 means that observed levels exceeded the expected levels on the basis of SDI. O:E ratios are listed in each cell. YLDs = years of life lived with disability.

Figure 22. Leading 10 causes of total YLDs with ratio of observed to expected deaths in 2019, by location, males

	1	2	3	4	5	6	7	8	9	10
Global	Age-related hearing loss 1.09	Diabetes 0.97	COPD 1.51	Low back pain 0.9	Blindness and vision loss 1.28	Stroke 1.16	Falls 1.12	Alzheimer's disease 1.04	Osteoarthritis 1.01	Oral disorders 0.87
Central Europe, Eastern Europe, and Central Asia	Age-related hearing loss 1.09	Low back pain 1.22	Diabetes 0.93	Falls 1.45	Blindness and vision loss 1.46	Alzheimer's disease 1.18	Oral disorders 1.12	Osteoarthritis 1.06	Stroke 1.12	COPD 0.79
Central Asia	Age-related hearing loss 0.97	Diabetes 0.81	Low back pain 1.09	Blindness and vision loss 1.25	Oral disorders 1.33	Alzheimer's disease 1.19	COPD 0.97	Stroke 0.87	Osteoarthritis 0.99	Falls 1.05
Armenia	Age-related hearing loss 0.98	Low back pain 1.15	Diabetes 0.83	Blindness and vision loss 1.33	Oral disorders 1.35	Alzheimer's disease 1.22	COPD 1.19	Osteoarthritis 0.97	Ischemic heart disease 1.09	Atrial fibrillation 1.39
Azerbaijan	Age-related hearing loss 0.98	Low back pain 1.1	Diabetes 0.79	Blindness and vision loss 1.35	Oral disorders 1.31	Alzheimer's disease 1.17	Stroke 0.88	COPD 0.97	Osteoarthritis 0.96	Ischemic heart disease 1.01
Georgia	Diabetes 1.06	Age-related hearing loss 0.99	Low back pain 0.99	Blindness and vision loss 1.43	Alzheimer's disease 1.19	Oral disorders 1.21	Falls 1.53	Depressive disorders 1.6	Stroke 0.91	COPD 0.85
Kazakhstan	Age-related hearing loss 1.01	Low back pain 1.13	Diabetes 0.89	Blindness and vision loss 1.57	Oral disorders 1.43	COPD 1.28	Alzheimer's disease 1.15	Stroke 1.04	Falls 1.09	Osteoarthritis 0.95
Kyrgyzstan	Age-related hearing loss 0.95	Low back pain 1.12	Blindness and vision loss 0.9	Oral disorders 1.35	COPD 1.57	Alzheimer's disease 1.25	Diabetes 0.49	Osteoarthritis 1.06	Depressive disorders 1.18	Ischemic heart disease 0.91
Mongolia	Age-related hearing loss 0.93	Blindness and vision loss 1.16	Low back pain 1.1	Oral disorders 1.27	Alzheimer's disease 1.2	Falls 1.7	Diabetes 0.36	Osteoarthritis 1.04	Ischemic heart disease 1.09	Depressive disorders 1.26
Tajikistan	Age-related hearing loss 0.96	Diabetes 0.83	Low back pain 1.09	Blindness and vision loss 0.92	Oral disorders 1.42	Alzheimer's disease 1.26	Osteoarthritis 1.03	Ischemic heart disease 1	Atrial fibrillation 1.66	Falls 1.36
Turkmenistan	Age-related hearing loss 0.96	Low back pain 1.09	Blindness and vision loss 1.34	Diabetes 0.66	Oral disorders 1.27	Alzheimer's disease 1.22	Stroke 0.98	Osteoarthritis 1.04	Ischemic heart disease 0.98	Atrial fibrillation 1.44
Uzbekistan	Age-related hearing loss 0.94	Diabetes 0.8	Low back pain 1.08	Blindness and vision loss 1.09	Oral disorders 1.3	Stroke 0.92	Osteoarthritis 1.04	Alzheimer's disease 1.14	Atrial fibrillation 1.5	Depressive disorders 1.04
Central Europe	Diabetes 1.33	Low back pain 1.3	Age-related hearing loss 1.08	Falls 1.56	COPD 1.01	Stroke 1.28	Alzheimer's disease 1.18	Oral disorders 1.06	Mechanical forces 2.54	Blindness and vision loss 1.17
Albania	Low back pain 1.39	Age-related hearing loss 0.96	Diabetes 0.64	Mechanical forces 3.78	Oral disorders 1.09	Alzheimer's disease 1.19	Blindness and vision loss 0.77	Stroke 0.99	COPD 1.07	Falls 1.14
Bosnia and Herzegovina	Diabetes 1.5	Age-related hearing loss 0.98	Low back pain 1.22	Stroke 1.56	Falls 1.44	COPD 1.17	Oral disorders 1.08	Alzheimer's disease 1.14	Blindness and vision loss 0.9	Mechanical forces 2.67
Bulgaria	Low back pain 1.36	Diabetes 1.1	Age-related hearing loss 1.04	Stroke 1.68	Falls 1.18	Mechanical forces 3.06	COPD 0.98	Alzheimer's disease 1.15	Oral disorders 1	Blindness and vision loss 1.08
Croatia	Diabetes 1.45	Low back pain 1.25	Age-related hearing loss 1.06	Falls 1.97	COPD 1.25	Total cancers 1.18	Alzheimer's disease 1.17	Stroke 1.22	Oral disorders 1.14	Blindness and vision loss 1.21
Czechia	Diabetes 2.32	Low back pain 1.28	Age-related hearing loss 1.08	Falls 1.47	COPD 0.88	Alzheimer's disease 1.18	Total cancers 0.92	Oral disorders 1.11	Stroke 1.15	Ischemic heart disease 1.63
Hungary	Diabetes 1.46	Low back pain 1.43	Age-related hearing loss 1.05	Falls 1.75	COPD 1.31	Stroke 1.38	Alzheimer's disease 1.18	Oral disorders 1.08	Blindness and vision loss 1.27	Total cancers 0.88
Montenegro	Diabetes 1.53	Low back pain 1.3	Age-related hearing loss 1.06	Falls 1.46	Stroke 1.33	Oral disorders 1.17	Alzheimer's disease 1.18	Ischemic heart disease 1.43	Blindness and vision loss 1.23	Mechanical forces 2.29
North Macedonia	Diabetes 1.57	Age-related hearing loss 1.01	Low back pain 1.19	Stroke 1.89	Oral disorders 1.16	Falls 1.09	COPD 0.91	Alzheimer's disease 1.12	Blindness and vision loss 1.02	Mechanical forces 2.53
Poland	Diabetes 1.3	Age-related hearing loss 1.17	Low back pain 1.25	Falls 1.66	COPD 0.96	Alzheimer's disease 1.2	Ischemic heart disease 1.5	Oral disorders 1	Osteoarthritis 0.87	Mechanical forces 2.32
Romania	Low back pain 1.32	Age-related hearing loss 1.03	Diabetes 0.87	Falls 1.48	Stroke 1.5	Alzheimer's disease 1.17	COPD 0.99	Mechanical forces 2.8	Blindness and vision loss 1.09	Oral disorders 1
Serbia	Diabetes 1.48	Low back pain 1.33	Age-related hearing loss 1.03	Stroke 1.66	COPD 1.19	Falls 1.16	Oral disorders 1.15	Alzheimer's disease 1.12	Blindness and vision loss 1.12	Mechanical forces 2.47
Slovakia	Low back pain 1.34	Falls 1.91	Age-related hearing loss 1.07	Diabetes 1.09	Stroke 1.36	Total cancers 1.04	Oral disorders 1.16	Alzheimer's disease 1.17	Mechanical forces 2.77	Ischemic heart disease 1.54
Slovenia	Falls 1.83	Low back pain 1.28	Diabetes 1.23	Age-related hearing loss 1	Alzheimer's disease 1.21	Mechanical forces 3.05	COPD 0.84	Oral disorders 1.27	Total cancers 0.81	Blindness and vision loss 1.47
Eastern Europe	Age-related hearing loss 1.13	Low back pain 1.16	Falls 1.4	Blindness and vision loss 1.75	Osteoarthritis 1.24	Diabetes 0.61	Alzheimer's disease 1.18	Oral disorders 1.13	Urinary diseases 2.86	Stroke 1.02
Belarus	Age-related hearing loss 1.06	Low back pain 1.22	Falls 1.9	Blindness and vision loss 1.37	Alzheimer's disease 1.21	Diabetes 0.52	Urinary diseases 2.72	Alcohol use disorders 6.51	Osteoarthritis 1.02	Oral disorders 0.97
Estonia	Age-related hearing loss 1.16	Low back pain 1.19	Falls 1.2	Diabetes 0.86	Total cancers 1.13	Alzheimer's disease 1.24	Urinary diseases 2.97	Depressive disorders 1.87	Osteoarthritis 0.91	Oral disorders 1.04
Latvia	Age-related hearing loss 1.13	Low back pain 1.17	Falls 1.31	Diabetes 0.79	Blindness and vision loss 1.91	Alzheimer's disease 1.19	Urinary diseases 3.19	Depressive disorders 1.92	Osteoarthritis 0.92	Stroke 1.01
Lithuania	Age-related hearing loss 1.16	Low back pain 1.2	Falls 1.43	Blindness and vision loss 2.11	Diabetes 0.7	Alzheimer's disease 1.19	Urinary diseases 3.27	Total cancers 0.84	Depressive disorders 1.8	Osteoarthritis 0.79
Republic of Moldova	Age-related hearing loss 1.01	Low back pain 1.21	Blindness and vision loss 1.58	Diabetes 0.7	Alzheimer's disease 1.2	Falls 1.4	Urinary diseases 2.62	Oral disorders 0.97	Osteoarthritis 1.1	Ischemic heart disease 1.08
Russian Federation	Age-related hearing loss 1.15	Low back pain 1.12	Falls 1.29	Blindness and vision loss 2.01	Osteoarthritis 1.35	Diabetes 0.62	Alzheimer's disease 1.18	Oral disorders 1.18	Urinary diseases 2.92	Stroke 1.1
Ukraine	Age-related hearing loss 1.08	Low back pain 1.27	Falls 1.74	Blindness and vision loss 1.3	Diabetes 0.57	Alzheimer's disease 1.17	Oral disorders 1.08	Urinary diseases 2.69	Depressive disorders 1.83	Osteoarthritis 1.02
High-income	Age-related hearing loss 1.16	Diabetes 1.25	Low back pain 1.04	COPD 1.2	Falls 0.94	Total cancers 1.01	Alzheimer's disease 1.09	Osteoarthritis 1.05	Stroke 1.06	Oral disorders 0.95
Australasia	Age-related hearing loss 1.34	Falls 1.43	Diabetes 1.04	Low back pain 0.91	Total cancers 1.26	COPD 1	Osteoarthritis 1.04	Mechanical forces 2.77	Oral disorders 1.12	Alzheimer's disease 0.94
Australia	Age-related hearing loss 1.34	Diabetes 1.07	Falls 1.43	Low back pain 0.92	Total cancers 1.26	COPD 0.99	Osteoarthritis 1.04	Mechanical forces 2.68	Oral disorders 1.1	Alzheimer's disease 0.93
New Zealand	Age-related hearing loss 1.34	Falls 1.44	Low back pain 0.89	Diabetes 0.88	Total cancers 1.25	COPD 1.02	Mechanical forces 3.22	Osteoarthritis 1.05	Oral disorders 1.22	Alzheimer's disease 1.03
High-income Asia Pacific	Age-related hearing loss 1.28	Low back pain 0.94	Diabetes 0.93	COPD 0.97	Stroke 1.49	Alzheimer's disease 1.11	Falls 0.74	Total cancers 0.79	Osteoarthritis 0.91	Oral disorders 0.94
Brunei Darussalam	Diabetes 2.86	Age-related hearing loss 1.19	COPD 1.67	Low back pain 0.86	Stroke 1.81	Osteoarthritis 0.97	Falls 0.81	Total cancers 0.8	Other musculoskeletal 1.95	Asthma 3.02
Japan	Age-related hearing loss 1.29	Low back pain 0.95	Diabetes 0.89	COPD 0.91	Stroke 1.5	Alzheimer's disease 1.13	Total cancers 0.81	Falls 0.72	Osteoarthritis 0.9	Oral disorders 0.98
Republic of Korea	Age-related hearing loss 1.27	COPD 1.35	Diabetes 1.11	Low back pain 0.88	Falls 0.89	Stroke 1.42	Osteoarthritis 0.94	Total cancers 0.7	Alzheimer's disease 0.98	Other musculoskeletal 1.94
Singapore	Age-related hearing loss 1.26	Diabetes 1.29	COPD 1.05	Low back pain 0.73	Osteoarthritis 0.95	Stroke 1.11	Alzheimer's disease 0.98	Other musculoskeletal 2.57	Falls 0.61	Total cancers 0.62
High-income North America	Diabetes 1.69	Age-related hearing loss 1.39	COPD 1.68	Low back pain 1.34	Total cancers 1.17	Osteoarthritis 1.26	Falls 0.94	Alzheimer's disease 1.24	Stroke 1.38	Atrial fibrillation 1.72
Canada	Age-related hearing loss 1.08	Total cancers 1.42	Low back pain 0.96	Diabetes 1	Falls 0.96	COPD 0.88	Alzheimer's disease 1.13	Stroke 1.28	Atrial fibrillation 1.6	Other musculoskeletal 3.3
Greenland	Age-related hearing loss 1.13	Low back pain 1.15	Falls 2.12	Stroke 2.22	Diabetes 0.93	COPD 1.48	Total cancers 1.31	Osteoarthritis 1.12	Alzheimer's disease 1.2	Atrial fibrillation 1.81
United States of America	Diabetes 1.77	Age-related hearing loss 1.43	COPD 1.79	Low back pain 1.39	Total cancers 1.14	Osteoarthritis 1.3	Falls 0.93	Alzheimer's disease 1.26	Stroke 1.39	Atrial fibrillation 1.73
Southern Latin America	Age-related hearing loss 1.16	Diabetes 0.96	Low back pain 0.8	Other musculoskeletal 4.16	Osteoarthritis 1.22	Alzheimer's disease 1.08	COPD 1	Oral disorders 0.83	Blindness and vision loss 0.69	Total cancers 0.99
Argentina	Age-related hearing loss 1.13	Diabetes 0.91	Low back pain 0.8	Other musculoskeletal 4.31	Osteoarthritis 1.24	Alzheimer's disease 1.08	COPD 1.1	Oral disorders 0.84	Blindness and vision loss 0.68	Total cancers 1.01
Chile	Age-related hearing loss 1.22	Diabetes 1.15	Low back pain 0.78	Other musculoskeletal 3.87	Osteoarthritis 1.16	Alzheimer's disease 1.08	Falls 0.92	COPD 0.74	Oral disorders 0.82	Total cancers 0.88
Uruguay	Age-related hearing loss 1.12	Diabetes 0.73	Low back pain 0.82	COPD 1.34	Alzheimer's disease 1.08	Osteoarthritis 1.27	Other musculoskeletal 4.08	Mechanical forces 2.8	Total cancers 1.42	Oral disorders 0.89
Western Europe	Diabetes 1.17	Age-related hearing loss 0.95	Low back pain 0.93	COPD 1.03	Falls 1.04	Total cancers 1.01	Alzheimer's disease 0.99	Osteoarthritis 0.99	Oral disorders 1.02	Atrial fibrillation 1.07
Andorra	Diabetes 1.08	Age-related hearing loss 0.93	Low back pain 0.91	COPD 0.98	Osteoarthritis 1.35	Total cancers 1	Falls 0.83	Alzheimer's disease 0.98	Oral disorders 1.06	Atrial fibrillation 0.94
Austria	Diabetes 1.06	Falls 1.28	Age-related hearing loss 0.84	Osteoarthritis 1.35	Low back pain 0.81	COPD 0.83	Oral disorders 1.22	Total cancers 0.87	Endo/metab/blood/immun 2.54	Alzheimer's disease 0.95
Belgium	Diabetes 1.12	Age-related hearing loss 0.95	COPD 1.28	Falls 1.31	Low back pain 0.91	Osteoarthritis 1.2	Total cancers 0.97	Oral disorders 1.22	Alzheimer's disease 0.95	Ischemic heart disease 1.01
Cyprus	Diabetes 1.55	Low back pain 0.98	Age-related hearing loss 0.87	COPD 1.17	Osteoarthritis 1.29	Total cancers 1.09	Falls 0.96	Oral disorders 1.07	Alzheimer's disease 0.97	Atrial fibrillation 0.99
Denmark	Low back pain 1.17	COPD 1.15	Age-related hearing loss 0.81	Total cancers 0.86	Diabetes 0.77	Osteoarthritis 1.09	Falls 0.72	Oral disorders 1.08	Alzheimer's disease 0.92	Stroke 0.74
Finland	Falls 1.68	Diabetes 1.21	Age-related hearing loss 0.87	Low back pain 0.86	Total cancers 1.04	COPD 0.79	Oral disorders 1.13	Alzheimer's disease 0.94	Osteoarthritis 0.84	Ischemic heart disease 1.28
France	Age-related hearing loss 0.98	Falls 1.4	Low back pain 0.89	Total cancers 1.04	Alzheimer's disease 0.98	Osteoarthritis 1	Diabetes 0.56	Oral disorders 1.04	COPD 0.51	Atrial fibrillation 1.05
Germany	Diabetes 1.5	Low back pain 1	Age-related hearing loss 0.94	COPD 0.93	Falls 0.84	Total cancers 0.81	Alzheimer's disease 1.05	Osteoarthritis 0.8	Oral disorders 1.05	Atrial fibrillation 1.03
Greece	Age-related hearing loss 0.99	Diabetes 1.07	COPD 1.49	Low back pain 0.88	Alzheimer's disease 0.96	Total cancers 1.13	Oral disorders 1.03	Depressive disorders 1.81	Falls 0.79	Osteoarthritis 0.89
Iceland	Diabetes 1.05	Age-related hearing loss 0.91	Low back pain 0.93	Osteoarthritis 1.4	Falls 0.94	COPD 0.89	Total cancers 0.93	Alzheimer's disease 0.96	Oral disorders 1.07	Atrial fibrillation 1.05
Ireland	Diabetes 1.13	Low back pain 1.07	Age-related hearing loss 0.9	COPD 1.15	Total cancers 0.99	Falls 0.79	Osteoarthritis 1.01	Oral disorders 1.19	Alzheimer's disease 0.98	Atrial fibrillation 1.04
Israel	Diabetes 0.99	Age-related hearing loss 0.87	Low back pain 0.95	Osteoarthritis 1.25	Oral disorders 1.12	Alzheimer's disease 0.96	COPD 0.77	Total cancers 0.86	Falls 0.74	Depressive disorders 1.42
Italy	Diabetes 1.21	Age-related hearing loss 1.07	Low back pain 0.94	COPD 1.06	Falls 1.16	Total cancers 1.23	Alzheimer's disease 1.12	Osteoarthritis 1.02	Blindness and vision loss 1.2	Oral disorders 0.94
Luxembourg	Diabetes 1.71	Falls 1.02	Age-related hearing loss 0.84	COPD 0.95	Low back pain 0.85	Total cancers 0.72	Osteoarthritis 0.96	Oral disorders 1.21	Alzheimer's disease 0.84	Atrial fibrillation 1.05
Malta	Diabetes 1.2	Low back pain 1.08	Age-related hearing loss 0.86	COPD 1.17	Falls 1.18	Osteoarthritis 1.11	Total cancers 0.91	Alzheimer's disease 0.96	Oral disorders 0.89	Ischemic heart disease 1.17
Monaco	Total cancers 1.23	Age-related hearing loss 0.93	Diabetes 1.04	Low back pain 0.9	COPD 0.89	Osteoarthritis 0.98	Falls 0.64	Alzheimer's disease 0.96	Oral disorders 0.99	Stroke 0.81
Netherlands	COPD 1.36	Diabetes 0.99	Age-related hearing loss 0.88	Total cancers 1.06	Low back pain 0.83	Falls 0.89	Oral disorders 1.45	Osteoarthritis 0.93	Alzheimer's disease 1.05	Atrial fibrillation 0.89
Norway	Age-related hearing loss 1.2	Falls 1.12	Diabetes 1.22	Low back pain 0.84	COPD 0.89	Total cancers 0.83	Stroke 1.36	Osteoarthritis 0.97	Alzheimer's disease 0.96	Oral disorders 1.05
Portugal	Diabetes 1.2	Age-related hearing loss 0.83	Low back pain 0.97	COPD 1.34	Total cancers 1.37	Alzheimer's disease 0.96	Osteoarthritis 1.09	Falls 1.01	Oral disorders 0.95	Depressive disorders 1.46
San Marino	Diabetes 1.11	Age-related hearing loss 0.92	Total cancers 1.18	Low back pain 0.91	COPD 0.95	Falls 0.76	Osteoarthritis 0.99	Alzheimer's disease 0.96	Oral disorders 1.06	Atrial fibrillation 0.96
Spain	Diabetes 1.28	Age-related hearing loss 0.95	COPD 1.53	Total cancers 1.47	Low back pain 0.71	Blindness and vision loss 1.26	Falls 1.04	Osteoarthritis 1.12	Alzheimer's disease 0.88	Depressive disorders 1.46
Sweden	Falls 1.21	Low back pain 0.97	Diabetes 0.97	COPD 0.82	Total cancers 0.86	Age-related hearing loss 0.59	Atrial fibrillation 1.46	Oral disorders 1.04	Osteoarthritis 0.82	Ischemic heart disease 1.55
Switzerland	Falls 1.03	Low back pain 1.04	Diabetes 1.2	Age-related hearing loss 0.86	Total cancers 0.7	COPD 0.68	Osteoarthritis 0.89	Alzheimer's disease 0.98	Oral disorders 0.94	Urinary diseases 2.25
United Kingdom	Diabetes 1.34	Age-related hearing loss 0.97	COPD 1.26	Low back pain 0.98	Total cancers 1.04	Falls 0.89	Osteoarthritis 1.09	Oral disorders 1.06	Alzheimer's disease 0.87	Atrial fibrillation 1.27

**Latin America and Caribbean**	Diabetes 1.24	Age-related hearing loss 0.96	Blindness and vision loss 1.17	Low back pain 0.75	Oral disorders 1.12	Alzheimer's disease 0.99	Osteoarthritis 1.17	Other musculoskeletal 3.29	Falls 1.36	Ischemic heart disease 0.91
**Andean Latin America**	Age-related hearing loss 1.06	Diabetes 0.86	Blindness and vision loss 1.31	Oral disorders 1.43	Low back pain 0.76	Alzheimer's disease 0.9	Osteoarthritis 1.1	Other musculoskeletal 3.09	Falls 0.95	Urinary diseases 1.43
Bolivia (Plurinational State of)	Age-related hearing loss 1.08	Diabetes 1.03	Blindness and vision loss 1.09	Oral disorders 1.46	Low back pain 0.81	Osteoarthritis 1.12	Alzheimer's disease 0.93	Other musculoskeletal 2.65	Depressive disorders 1.07	Falls 1.16
Ecuador	Age-related hearing loss 1.11	Diabetes 1.09	Blindness and vision loss 1.04	Oral disorders 1.34	Low back pain 0.74	Osteoarthritis 1.15	Alzheimer's disease 0.91	Other musculoskeletal 2.94	Falls 1.21	Urinary diseases 1.75
Peru	Age-related hearing loss 1.04	Blindness and vision loss 1.5	Diabetes 0.72	Oral disorders 1.46	Low back pain 0.76	Alzheimer's disease 0.88	Osteoarthritis 1.07	Other musculoskeletal 3.28	Ischemic heart disease 0.78	Falls 0.8
**Caribbean**	Diabetes 1.54	Age-related hearing loss 1.07	Blindness and vision loss 1.09	Low back pain 0.7	Oral disorders 0.96	Alzheimer's disease 0.86	Osteoarthritis 1.04	Total cancers 1.35	Falls 1.04	Depressive disorders 1.29
Antigua and Barbuda	Diabetes 1.87	Age-related hearing loss 1.13	Low back pain 0.68	Total cancers 1.49	Blindness and vision loss 1.27	Prostate cancer 3.18	Oral disorders 0.91	Osteoarthritis 0.94	Alzheimer's disease 0.88	Mechanical forces 1.96
Bahamas	Diabetes 1.8	Age-related hearing loss 1.21	Low back pain 0.67	Blindness and vision loss 1.64	Total cancers 0.99	Osteoarthritis 0.9	Oral disorders 0.93	Alzheimer's disease 0.87	Prostate cancer 2.01	Other musculoskeletal 2.3
Barbados	Diabetes 1.63	Age-related hearing loss 1.12	Total cancers 1.75	Low back pain 0.69	Prostate cancer 3.54	Oral disorders 0.93	Osteoarthritis 0.98	Alzheimer's disease 0.88	Other musculoskeletal 2.66	Ischemic heart disease 1.08
Belize	Age-related hearing loss 1.02	Diabetes 1	Blindness and vision loss 0.77	Low back pain 0.74	Oral disorders 0.9	Osteoarthritis 1.19	Alzheimer's disease 0.89	Ischemic heart disease 1.04	Depressive disorders 1.07	Chronic kidney disease 1.39
Bermuda	Diabetes 1.44	Age-related hearing loss 1.22	Total cancers 1.58	Low back pain 0.69	Blindness and vision loss 1.61	Prostate cancer 2.87	Osteoarthritis 0.9	Alzheimer's disease 0.91	Oral disorders 0.92	Ischemic heart disease 1.14
Cuba	Diabetes 1.51	Age-related hearing loss 1.05	Blindness and vision loss 1.24	Low back pain 0.68	Falls 1.6	Total cancers 1.83	Oral disorders 0.91	Alzheimer's disease 0.82	Depressive disorders 1.55	Osteoarthritis 1.06
Dominica	Diabetes 2.23	Age-related hearing loss 1.11	Total cancers 1.74	Blindness and vision loss 1.25	Low back pain 0.69	Prostate cancer 3.59	Oral disorders 0.93	Osteoarthritis 0.96	Alzheimer's disease 0.84	Chronic kidney disease 1.65
Dominican Republic	Age-related hearing loss 1.02	Diabetes 0.77	Blindness and vision loss 0.92	Low back pain 0.75	Oral disorders 1.01	Osteoarthritis 1.16	Alzheimer's disease 0.88	Depressive disorders 1.38	Ischemic heart disease 1.1	Road injuries 1.56
Grenada	Diabetes 1.88	Age-related hearing loss 1.05	Total cancers 2.14	Low back pain 0.7	Blindness and vision loss 0.94	Prostate cancer 4.26	Oral disorders 0.89	Osteoarthritis 1.03	Chronic kidney disease 1.99	Ischemic heart disease 0.96
Guyana	Diabetes 1.77	Age-related hearing loss 1.01	Blindness and vision loss 0.81	Low back pain 0.71	Oral disorders 0.9	Depressive disorders 1.46	Osteoarthritis 1.09	Lymphatic filariasis 8.35	Falls 1.32	Alzheimer's disease 0.84
Haiti	Diabetes 1.68	Age-related hearing loss 1.06	Blindness and vision loss 0.74	Low back pain 0.71	Oral disorders 1.18	Osteoarthritis 1.03	Alzheimer's disease 0.96	Nature disaster 187.22	Depressive disorders 0.84	Ischemic heart disease 1.03
Jamaica	Diabetes 1.71	Age-related hearing loss 1.07	Blindness and vision loss 0.98	Low back pain 0.72	Alzheimer's disease 0.86	Oral disorders 0.93	Total cancers 1.49	Osteoarthritis 1.03	Ischemic heart disease 0.96	Prostate cancer 2.86
Puerto Rico	Diabetes 2.29	Age-related hearing loss 1.2	Low back pain 0.66	Blindness and vision loss 1.59	Alzheimer's disease 0.9	Osteoarthritis 0.93	Falls 0.74	Oral disorders 0.94	Ischemic heart disease 1.25	Total cancers 0.74
Saint Kitts and Nevis	Diabetes 1.89	Age-related hearing loss 1.13	Total cancers 2.17	Prostate cancer 4.72	Low back pain 0.68	Blindness and vision loss 1.27	Osteoarthritis 0.96	Oral disorders 0.9	Depressive disorders 1.45	Chronic kidney disease 1.99
Saint Lucia	Diabetes 1.89	Age-related hearing loss 1.04	Blindness and vision loss 0.94	Low back pain 0.71	Total cancers 1.76	Oral disorders 0.89	Osteoarthritis 1.05	Alzheimer's disease 0.86	Prostate cancer 3.39	Chronic kidney disease 1.52
Saint Vincent and the Grenadines	Diabetes 1.71	Age-related hearing loss 1.02	Blindness and vision loss 0.81	Low back pain 0.7	Oral disorders 0.88	Osteoarthritis 1.11	Total cancers 1.82	Alzheimer's disease 0.87	Ischemic heart disease 0.92	Depressive disorders 1.13
Suriname	Diabetes 1.76	Age-related hearing loss 1.02	Blindness and vision loss 0.98	Low back pain 0.71	Oral disorders 0.87	Osteoarthritis 1.12	Alzheimer's disease 0.84	Depressive disorders 1.43	Ischemic heart disease 1.07	Chronic kidney disease 1.44
Trinidad and Tobago	Diabetes 2.37	Age-related hearing loss 1.15	Low back pain 0.69	Blindness and vision loss 1.31	Osteoarthritis 0.94	Oral disorders 0.91	Ischemic heart disease 1.26	Alzheimer's disease 0.86	Other musculoskeletal 2.45	Depressive disorders 1.3
United States Virgin Islands	Diabetes 1.95	Age-related hearing loss 1.2	Total cancers 1.5	Low back pain 0.67	Prostate cancer 3	Blindness and vision loss 1.64	Osteoarthritis 0.95	Oral disorders 0.78	Other musculoskeletal 2.07	Ischemic heart disease 1.1
**Central Latin America**	Diabetes 1.48	Age-related hearing loss 1.02	Blindness and vision loss 1.14	Low back pain 0.62	Other musculoskeletal 3.9	Osteoarthritis 1.27	Alzheimer's disease 0.86	Oral disorders 0.81	Chronic kidney disease 1.76	Falls 1.21
Colombia	Diabetes 1.28	Age-related hearing loss 1	Blindness and vision loss 1.14	Low back pain 0.72	Alzheimer's disease 0.92	Other musculoskeletal 4.28	Osteoarthritis 1.11	COPD 0.92	Oral disorders 0.69	Chronic kidney disease 1.38
Costa Rica	Diabetes 1.5	Age-related hearing loss 1.03	Blindness and vision loss 1.24	Low back pain 0.64	Other musculoskeletal 4.12	Alzheimer's disease 0.91	Osteoarthritis 1.06	Total cancers 1.41	Oral disorders 0.79	Chronic kidney disease 1.77
El Salvador	Diabetes 1.4	Age-related hearing loss 0.99	Blindness and vision loss 1.16	Low back pain 0.67	Alzheimer's disease 0.95	Osteoarthritis 1.16	Chronic kidney disease 1.85	Oral disorders 0.79	Other musculoskeletal 2.83	Falls 1.44
Guatemala	Diabetes 1.57	Age-related hearing loss 1.01	Blindness and vision loss 1.05	Low back pain 0.74	Oral disorders 0.9	Falls 2.04	Osteoarthritis 1.12	Alzheimer's disease 0.98	Chronic kidney disease 1.97	Depressive disorders 1.2
Honduras	Diabetes 1.66	Age-related hearing loss 1.03	Blindness and vision loss 0.78	Low back pain 0.69	Other musculoskeletal 3.59	Oral disorders 0.9	Osteoarthritis 1.15	Alzheimer's disease 1.01	Depressive disorders 1.19	Urinary diseases 1.81
Mexico	Diabetes 1.6	Age-related hearing loss 1.05	Blindness and vision loss 1.21	Osteoarthritis 1.44	Other musculoskeletal 3.89	Low back pain 0.55	Falls 1.46	Oral disorders 0.87	Chronic kidney disease 1.93	Alzheimer's disease 0.79
Nicaragua	Diabetes 1.48	Age-related hearing loss 1.02	Blindness and vision loss 0.91	Low back pain 0.68	Other musculoskeletal 3.06	Chronic kidney disease 2.19	Osteoarthritis 1.16	Oral disorders 0.87	Alzheimer's disease 1.04	Depressive disorders 1.06
Panama	Diabetes 1.49	Age-related hearing loss 1.04	Blindness and vision loss 1.62	Other musculoskeletal 4.37	Low back pain 0.64	Alzheimer's disease 0.9	Osteoarthritis 1.03	Oral disorders 0.79	Chronic kidney disease 1.64	Depressive disorders 1.23
Venezuela (Bolivarian Republic of)	Diabetes 1.29	Age-related hearing loss 1	Blindness and vision loss 0.96	Other musculoskeletal 4.08	Low back pain 0.62	Alzheimer's disease 0.88	Osteoarthritis 1.07	Oral disorders 0.79	Depressive disorders 1.33	Total cancers 1.76
**Tropical Latin America**	Diabetes 1.03	Age-related hearing loss 0.84	Blindness and vision loss 1.19	Low back pain 0.88	Oral disorders 1.4	Alzheimer's disease 1.2	Falls 1.72	Osteoarthritis 1.13	Other musculoskeletal 3.04	Ischemic heart disease 0.97
Brazil	Diabetes 1.03	Age-related hearing loss 0.84	Blindness and vision loss 1.19	Low back pain 0.88	Oral disorders 1.41	Alzheimer's disease 1.21	Falls 1.74	Osteoarthritis 1.13	Other musculoskeletal 3.03	Ischemic heart disease 0.97
Paraguay	Age-related hearing loss 1.01	Diabetes 1.01	Blindness and vision loss 1.13	Low back pain 0.86	Alzheimer's disease 1.06	Oral disorders 0.98	Other musculoskeletal 3.37	Osteoarthritis 1.07	Ischemic heart disease 1.03	Stroke 0.71
**North Africa and Middle East**	Diabetes 1.18	Age-related hearing loss 1.06	Blindness and vision loss 1.48	Low back pain 1.08	COPD 1.49	Alzheimer's disease 1.32	Oral disorders 1.21	Stroke 1.2	Osteoarthritis 0.94	Chronic kidney disease 1.84
**North Africa and Middle East**	Diabetes 1.18	Age-related hearing loss 1.06	Blindness and vision loss 1.48	Low back pain 1.08	COPD 1.49	Alzheimer's disease 1.32	Oral disorders 1.21	Stroke 1.2	Osteoarthritis 0.94	Chronic kidney disease 1.84
Afghanistan	Blindness and vision loss 1.28	Diabetes 1.53	Conflict & terror 312.66	Age-related hearing loss 1.06	Low back pain 0.98	Oral disorders 1.7	Alzheimer's disease 1.48	Stroke 1.18	Leishmaniasis 899.21	Depressive disorders 0.86
Algeria	Diabetes 1.24	Age-related hearing loss 1.02	Blindness and vision loss 1.44	Low back pain 1.03	Stroke 1.28	Alzheimer's disease 1.3	Oral disorders 1.15	COPD 1.12	Osteoarthritis 0.98	Chronic kidney disease 1.75
Bahrain	Diabetes 3.03	Age-related hearing loss 1.09	Low back pain 0.97	Blindness and vision loss 2.11	COPD 1.25	Oral disorders 1.12	Alzheimer's disease 1.31	Chronic kidney disease 2.53	Ischemic heart disease 1.29	Osteoarthritis 0.88
Egypt	Age-related hearing loss 1.03	Diabetes 0.91	Blindness and vision loss 1.46	Low back pain 1.06	COPD 1.8	Stroke 1.32	Oral disorders 1.16	Alzheimer's disease 1.26	Chronic kidney disease 1.92	Osteoarthritis 1
Iran (Islamic Republic of)	Age-related hearing loss 1.05	Diabetes 1.09	Blindness and vision loss 1.8	Low back pain 1.17	Alzheimer's disease 1.32	COPD 1.25	Oral disorders 1.07	Stroke 1.04	Osteoarthritis 0.99	Ischemic heart disease 0.99
Iraq	Diabetes 1.28	Age-related hearing loss 1.02	Blindness and vision loss 1.56	Low back pain 1.01	Stroke 1.48	Conflict & terror 737.61	Oral disorders 1.15	Alzheimer's disease 1.26	Chronic kidney disease 2.12	Ischemic heart disease 1.24
Jordan	Diabetes 1.38	Age-related hearing loss 1.09	Low back pain 1.01	Blindness and vision loss 1.55	Stroke 1.52	Oral disorders 1.24	Alzheimer's disease 1.29	COPD 0.97	Chronic kidney disease 2.13	Osteoarthritis 0.91
Kuwait	Diabetes 2.43	Age-related hearing loss 1.23	Blindness and vision loss 3.07	Low back pain 0.95	Alzheimer's disease 1.37	Oral disorders 1.22	Stroke 1.18	Ischemic heart disease 1.43	Osteoarthritis 0.78	Chronic kidney disease 2.04
Lebanon	Diabetes 1.67	Age-related hearing loss 1.06	Blindness and vision loss 1.74	COPD 1.65	Low back pain 0.91	Alzheimer's disease 1.29	Oral disorders 1.18	Stroke 1.06	Total cancers 1.41	Chronic kidney disease 1.91
Libya	Diabetes 1.64	Age-related hearing loss 1.06	Blindness and vision loss 2.04	Low back pain 0.98	COPD 1.4	Alzheimer's disease 1.25	Oral disorders 1.17	Stroke 1.05	Ischemic heart disease 1.15	Osteoarthritis 0.91
Morocco	Diabetes 1.2	Age-related hearing loss 1.02	Low back pain 1.12	Blindness and vision loss 0.89	Oral disorders 1.26	Alzheimer's disease 1.38	Stroke 1.32	COPD 1.27	Ischemic heart disease 1.28	Depressive disorders 1.32
Oman	Diabetes 1.74	Blindness and vision loss 4.23	Age-related hearing loss 1.12	Low back pain 0.97	Stroke 1.61	Oral disorders 1.16	Chronic kidney disease 2.58	Alzheimer's disease 1.3	Ischemic heart disease 1.44	Osteoarthritis 0.82
Palestine	Diabetes 1.6	Age-related hearing loss 0.98	Blindness and vision loss 1.03	Low back pain 1.03	Depressive disorders 2.02	Oral disorders 1.18	Stroke 1.21	Alzheimer's disease 1.33	Conflict & terror 276.53	Chronic kidney disease 1.98
Qatar	Diabetes 3.55	Age-related hearing loss 1.14	Low back pain 0.98	Blindness and vision loss 3.25	Oral disorders 1.12	Chronic kidney disease 2.94	Stroke 1.07	Osteoarthritis 0.81	COPD 0.71	Alzheimer's disease 1.32
Saudi Arabia	Diabetes 1.71	Blindness and vision loss 4.4	Age-related hearing loss 1.14	Falls 1.7	Low back pain 0.96	Stroke 1.6	COPD 0.94	Oral disorders 1.12	Alzheimer's disease 1.27	Osteoarthritis 0.96
Sudan	Diabetes 1.21	Blindness and vision loss 1.27	Age-related hearing loss 1.03	Low back pain 1.03	Oral disorders 1.25	Stroke 1.35	Alzheimer's disease 1.39	COPD 1.06	Osteoarthritis 1.02	Ischemic heart disease 1.17
Syrian Arab Republic	Diabetes 1.1	Age-related hearing loss 1	Conflict & terror 843.55	Blindness and vision loss 1.29	Low back pain 1.03	Oral disorders 1.18	Alzheimer's disease 1.29	Stroke 1.11	COPD 1.37	Ischemic heart disease 1.2
Tunisia	Diabetes 1.54	Age-related hearing loss 1.03	Blindness and vision loss 1.37	Low back pain 0.98	COPD 1.74	Alzheimer's disease 1.29	Oral disorders 1.16	Stroke 1.05	Depressive disorders 1.73	Osteoarthritis 0.94
Turkey	Age-related hearing loss 1.13	Diabetes 1.14	COPD 2.14	Low back pain 1.16	Blindness and vision loss 1.47	Alzheimer's disease 1.33	Oral disorders 1.32	Stroke 1.02	Chronic kidney disease 1.98	Osteoarthritis 0.82
United Arab Emirates	Diabetes 2.61	Age-related hearing loss 1.22	Stroke 2.27	Low back pain 0.94	Blindness and vision loss 4.18	COPD 0.89	Oral disorders 1.23	Chronic kidney disease 2.94	Alzheimer's disease 1.23	Osteoarthritis 0.72
Yemen	Age-related hearing loss 1.06	Blindness and vision loss 1.04	Diabetes 0.96	Low back pain 1.03	Oral disorders 1.56	Stroke 1.3	Alzheimer's disease 1.48	COPD 1.19	Depressive disorders 0.9	Osteoarthritis 0.93
**South Asia**	Blindness and vision loss 1.53	COPD 3.16	Age-related hearing loss 0.99	Diabetes 0.97	Road injuries 3.05	Low back pain 0.77	Falls 2.14	Dietary iron deficiency 4.48	Depressive disorders 1.44	Other musculoskeletal 2.67
**South Asia**	Blindness and vision loss 1.53	COPD 3.16	Age-related hearing loss 0.99	Diabetes 0.97	Road injuries 3.05	Low back pain 0.77	Falls 2.14	Dietary iron deficiency 4.48	Depressive disorders 1.44	Other musculoskeletal 2.67
Bangladesh	Blindness and vision loss 1.17	Age-related hearing loss 0.98	COPD 2.1	Diabetes 0.83	Low back pain 1.03	Depressive disorders 1.66	Road injuries 2.2	Other musculoskeletal 3.56	Alzheimer's disease 1.09	Dietary iron deficiency 2.83
Bhutan	COPD 3.01	Age-related hearing loss 0.98	Diabetes 0.95	Low back pain 1.02	Blindness and vision loss 0.71	Road injuries 2.18	Depressive disorders 1.4	Falls 2.21	Alzheimer's disease 0.95	Osteoarthritis 0.99
India	Blindness and vision loss 1.61	COPD 3.38	Age-related hearing loss 0.99	Diabetes 0.97	Road injuries 3.36	Low back pain 0.72	Falls 2.43	Dietary iron deficiency 5.01	Depressive disorders 1.44	Other musculoskeletal 2.57
Nepal	COPD 3.15	Age-related hearing loss 0.94	Blindness and vision loss 0.98	Diabetes 1.04	Low back pain 1.13	Depressive disorders 1.59	Dietary iron deficiency 2.9	Falls 2.01	Road injuries 1.42	Osteoarthritis 0.95
Pakistan	Blindness and vision loss 1.47	Age-related hearing loss 1.03	Diabetes 1.21	COPD 2.61	Low back pain 0.87	Dietary iron deficiency 3.17	Depressive disorders 1.11	Road injuries 1.54	Osteoarthritis 0.95	Oral disorders 0.8
**Southeast Asia, East Asia, and Oceania**	Age-related hearing loss 1.15	Stroke 1.78	Blindness and vision loss 1.25	Diabetes 0.65	COPD 1.68	Low back pain 0.75	Alzheimer's disease 1.09	Falls 1.09	Oral disorders 0.76	Osteoarthritis 0.9
**East Asia**	Age-related hearing loss 1.19	Stroke 1.82	COPD 1.55	Blindness and vision loss 1.12	Diabetes 0.56	Low back pain 0.67	Alzheimer's disease 1.16	Falls 1.12	Osteoarthritis 0.96	Oral disorders 0.75
China	Age-related hearing loss 1.19	Stroke 1.83	Blindness and vision loss 1.14	COPD 1.58	Diabetes 0.55	Low back pain 0.65	Alzheimer's disease 1.17	Falls 1.16	Osteoarthritis 0.96	Oral disorders 0.75
Democratic People's Republic of Korea	Age-related hearing loss 1.01	Low back pain 1.09	COPD 2.29	Stroke 1.77	Diabetes 0.64	Oral disorders 0.73	Blindness and vision loss 0.39	Osteoarthritis 0.96	Alzheimer's disease 0.95	Urinary diseases 1.47
Taiwan (Province of China)	Diabetes 1.32	Age-related hearing loss 1.09	Low back pain 1.12	Stroke 1.35	Chronic kidney disease 3.25	Alzheimer's disease 0.87	Total cancers 0.65	Osteoarthritis 0.72	COPD 0.47	Urinary diseases 2.21
**Oceania**	Diabetes 1.79	Age-related hearing loss 0.98	Low back pain 1.19	Blindness and vision loss 1.02	Stroke 1.49	Oral disorders 1.4	COPD 1.43	Ischemic heart disease 1.19	Alzheimer's disease 0.97	Osteoarthritis 0.79
American Samoa	Diabetes 2.54	Age-related hearing loss 0.98	Low back pain 1.17	Stroke 1.56	Blindness and vision loss 1.33	Oral disorders 1.11	Ischemic heart disease 1.17	Alzheimer's disease 0.85	COPD 0.78	Chronic kidney disease 1.76
Cook Islands	Diabetes 2.03	Age-related hearing loss 1.03	Low back pain 1.17	COPD 1.49	Stroke 1.75	Blindness and vision loss 1.83	Oral disorders 1.13	Alzheimer's disease 0.87	Total cancers 0.97	Osteoarthritis 0.75
Fiji	Diabetes 2.68	Age-related hearing loss 0.94	Low back pain 1.15	Blindness and vision loss 1.42	Stroke 1.52	Oral disorders 1.09	Ischemic heart disease 1.2	Chronic kidney disease 1.9	Osteoarthritis 0.81	Urinary diseases 1.53
Guam	Diabetes 1.43	Age-related hearing loss 1.11	Low back pain 1.16	Stroke 1.89	Blindness and vision loss 2.06	Oral disorders 1.14	Ischemic heart disease 1.53	Alzheimer's disease 0.89	Osteoarthritis 0.7	COPD 0.48
Kiribati	Diabetes 2.13	Age-related hearing loss 0.95	Low back pain 1.26	Stroke 2.15	Blindness and vision loss 0.84	Oral disorders 1.28	Ischemic heart disease 1.17	Osteoarthritis 0.85	Urinary diseases 1.61	Diarrheal diseases 1.82
Marshall Islands	Diabetes 2.39	Age-related hearing loss 0.93	Low back pain 1.19	Stroke 1.73	Blindness and vision loss 0.82	Oral disorders 1.18	COPD 1.06	Ischemic heart disease 1.05	Osteoarthritis 0.83	Alzheimer's disease 0.88
Micronesia (Federated States of)	Diabetes 1.62	Age-related hearing loss 0.93	Low back pain 1.22	Blindness and vision loss 0.86	Stroke 1.54	Oral disorders 1.17	COPD 1.08	Alzheimer's disease 0.88	Chronic kidney disease 1.61	Osteoarthritis 0.87
Nauru	Diabetes 1.45	Age-related hearing loss 0.92	Low back pain 1.22	Stroke 1.81	COPD 1.94	Blindness and vision loss 0.91	Oral disorders 1.1	Ischemic heart disease 1.15	Alzheimer's disease 0.87	Chronic kidney disease 1.54
Niue	Diabetes 2.19	Age-related hearing loss 0.97	Low back pain 1.19	COPD 1.63	Stroke 1.59	Blindness and vision loss 1.25	Oral disorders 1.14	Ischemic heart disease 1.25	Alzheimer's disease 0.86	Chronic kidney disease 1.66
Northern Mariana Islands	Diabetes 1.74	Age-related hearing loss 1.07	Low back pain 1.15	Stroke 1.72	Blindness and vision loss 1.72	Oral disorders 1.13	Ischemic heart disease 1.41	COPD 0.72	Alzheimer's disease 0.89	Chronic kidney disease 1.96
Palau	Diabetes 1.93	Age-related hearing loss 1.02	Low back pain 1.15	Stroke 2.02	COPD 1.57	Blindness and vision loss 1.48	Oral disorders 1.13	Falls 1.14	Ischemic heart disease 1.49	Osteoarthritis 0.76
Papua New Guinea	Diabetes 1.65	Age-related hearing loss 0.98	Blindness and vision loss 1.01	Low back pain 1.19	COPD 1.77	Stroke 1.42	Oral disorders 1.51	Lymphatic filariasis 3.49	Alzheimer's disease 1.01	Ischemic heart disease 1.16

Samoa	Diabetes 1.42	Age-related hearing loss 0.95	Low back pain 1.28	Stroke 1.63	Blindness and vision loss 1.02	Oral disorders 1.17	Alzheimer's disease 0.86	COPD 1	Ischemic heart disease 1.08	Osteoarthritis 0.85
Solomon Islands	Diabetes 1.98	Age-related hearing loss 0.98	Low back pain 1.18	Blindness and vision loss 0.77	Stroke 1.55	Oral disorders 1.53	Ischemic heart disease 1.4	COPD 0.9	Diarrheal diseases 1.6	Alzheimer's disease 1.02
Tokelau	Diabetes 1.4	Age-related hearing loss 0.92	Low back pain 1.24	COPD 2.04	Blindness and vision loss 0.92	Stroke 1.4	Oral disorders 1.13	Alzheimer's disease 0.86	Ischemic heart disease 1.11	Osteoarthritis 0.86
Tonga	Diabetes 1.47	Age-related hearing loss 0.94	Low back pain 1.26	Stroke 1.26	Oral disorders 1.15	Blindness and vision loss 0.73	Ischemic heart disease 1.26	COPD 1.16	Alzheimer's disease 0.87	Osteoarthritis 0.86
Tuvalu	Diabetes 1.48	Age-related hearing loss 0.93	Low back pain 1.24	Blindness and vision loss 0.86	COPD 1.79	Stroke 1.4	Oral disorders 1.17	Ischemic heart disease 1.09	Alzheimer's disease 0.88	Osteoarthritis 0.86
Vanuatu	Diabetes 1.61	Age-related hearing loss 0.96	Low back pain 1.37	Stroke 1.92	Blindness and vision loss 0.64	Oral disorders 1.31	Ischemic heart disease 1.34	COPD 0.89	Alzheimer's disease 0.94	Osteoarthritis 0.84
**Southeast Asia**	Diabetes 1.04	Blindness and vision loss 1.71	Age-related hearing loss 0.97	COPD 2.28	Low back pain 1.1	Stroke 1.58	Oral disorders 0.78	Alzheimer's disease 0.81	Neck pain 2.23	Ischemic heart disease 0.9
Cambodia	Diabetes 1.42	Blindness and vision loss 1.27	Age-related hearing loss 0.97	Low back pain 1.2	COPD 2.04	Stroke 1.45	Falls 2.2	Oral disorders 0.87	Alzheimer's disease 0.91	Neck pain 2.11
Indonesia	Blindness and vision loss 2.56	Age-related hearing loss 0.97	Diabetes 0.81	Low back pain 1.11	Stroke 1.9	COPD 2.18	Oral disorders 0.83	Neck pain 2.32	Ischemic heart disease 0.94	Alzheimer's disease 0.77
Lao People's Democratic Republic	Diabetes 1.45	Age-related hearing loss 0.98	Low back pain 1.17	COPD 1.96	Blindness and vision loss 0.74	Stroke 1.74	Alzheimer's disease 0.9	Neck pain 2.07	Oral disorders 0.64	Urinary diseases 1.52
Malaysia	Diabetes 1.36	Age-related hearing loss 1.05	Blindness and vision loss 2.16	Low back pain 1.03	COPD 1.52	Stroke 1.54	Oral disorders 0.97	Ischemic heart disease 1.34	Alzheimer's disease 0.85	Road injuries 1.87
Maldives	Diabetes 1.22	Age-related hearing loss 0.95	COPD 2.28	Low back pain 1.12	Blindness and vision loss 0.87	Stroke 1.34	Alzheimer's disease 0.91	Ischemic heart disease 1.13	Oral disorders 0.74	Neck pain 2.05
Mauritius	Diabetes 2.26	Age-related hearing loss 1	Low back pain 1.08	Blindness and vision loss 1.65	COPD 1.67	Stroke 1.35	Oral disorders 0.98	Alzheimer's disease 0.81	Chronic kidney disease 1.75	Neck pain 1.98
Myanmar	Diabetes 1.28	COPD 3.17	Blindness and vision loss 1.24	Age-related hearing loss 0.97	Low back pain 1.05	Stroke 1.71	Falls 1.83	Alzheimer's disease 0.87	Neck pain 2.01	Oral disorders 0.59
Philippines	COPD 3.24	Age-related hearing loss 0.98	Low back pain 1.2	Blindness and vision loss 1.26	Diabetes 0.79	Stroke 1.23	Oral disorders 0.95	Neck pain 3.02	Alzheimer's disease 0.79	Ischemic heart disease 0.89
Seychelles	Diabetes 2.22	Age-related hearing loss 1.03	Blindness and vision loss 1.91	Low back pain 1.04	COPD 1.62	Stroke 1.64	Total cancers 1.18	Oral disorders 0.72	Alzheimer's disease 0.8	Neck pain 1.93
Sri Lanka	Diabetes 2.3	Age-related hearing loss 1	Blindness and vision loss 1.65	Low back pain 1.05	COPD 2.12	Stroke 1.35	Falls 1.2	Alzheimer's disease 0.81	Oral disorders 0.68	Neck pain 2.02
Thailand	Diabetes 1.05	Age-related hearing loss 0.98	Low back pain 1.04	COPD 1.88	Blindness and vision loss 1.32	Stroke 1.12	Alzheimer's disease 0.82	Oral disorders 0.77	Other musculoskeletal 2.62	Ischemic heart disease 0.93
Timor-Leste	Blindness and vision loss 1.6	Diabetes 1.24	Age-related hearing loss 0.96	Low back pain 1.09	Stroke 1.89	COPD 2.04	Oral disorders 0.8	Neck pain 2	Alzheimer's disease 0.87	Conflict & terror 138.7
Viet Nam	Diabetes 1.1	Age-related hearing loss 0.93	Low back pain 1.17	Blindness and vision loss 1.16	COPD 2.44	Stroke 1.8	Alzheimer's disease 0.82	Falls 1.19	Neck pain 2.14	Urinary diseases 1.61
**Sub-Saharan Africa**	Age-related hearing loss 0.97	Blindness and vision loss 1.12	Diabetes 1.01	Low back pain 1.06	Depressive disorders 1.59	Road injuries 1.67	Osteoarthritis 1.17	COPD 1.07	Stroke 0.94	Alzheimer's disease 0.87
**Central Sub-Saharan Africa**	Diabetes 1.37	Age-related hearing loss 0.98	Low back pain 1.01	Onchocerciasis 39.44	Depressive disorders 1.71	Blindness and vision loss 0.6	COPD 1.09	Osteoarthritis 1.1	Stroke 0.93	Alzheimer's disease 1.09
Angola	Diabetes 1.3	Age-related hearing loss 0.96	Low back pain 1.04	Blindness and vision loss 0.85	Depressive disorders 1.97	Stroke 1.17	Osteoarthritis 1.13	COPD 0.97	Alzheimer's disease 1.07	Oral disorders 0.71
Central African Republic	Diabetes 1.56	Age-related hearing loss 1	Low back pain 0.96	Onchocerciasis 8.88	Depressive disorders 1.38	Blindness and vision loss 0.58	COPD 1.03	Osteoarthritis 1.05	Stroke 0.95	Oral disorders 1.05
Congo	Diabetes 1.21	Age-related hearing loss 0.92	Low back pain 1.03	Blindness and vision loss 0.86	Depressive disorders 2.24	Stroke 1.06	Osteoarthritis 1.17	COPD 1.09	Alzheimer's disease 1.06	Oral disorders 0.63
Democratic Republic of the Congo	Diabetes 1.41	Age-related hearing loss 0.99	Onchocerciasis 48.62	Low back pain 1	Depressive disorders 1.6	Blindness and vision loss 0.49	COPD 1.12	Osteoarthritis 1.08	Alzheimer's disease 1.11	Stroke 0.83
Equatorial Guinea	Diabetes 1.28	Age-related hearing loss 0.97	Blindness and vision loss 1.77	Low back pain 0.98	Depressive disorders 2.49	Stroke 1.08	Osteoarthritis 1.06	COPD 0.95	Alzheimer's disease 0.94	Oral disorders 0.6
Gabon	Diabetes 1.38	Age-related hearing loss 0.93	Low back pain 0.99	Blindness and vision loss 1.12	Depressive disorders 2.55	Stroke 1.09	Osteoarthritis 1.14	COPD 1.14	Alzheimer's disease 0.94	Road injuries 1.45
**Eastern Sub-Saharan Africa**	Age-related hearing loss 0.98	Blindness and vision loss 1.04	Low back pain 1.11	Diabetes 0.94	Depressive disorders 1.63	Road injuries 1.54	Osteoarthritis 1.14	Stroke 0.95	COPD 0.94	Alzheimer's disease 0.95
Burundi	Age-related hearing loss 0.98	Low back pain 1.1	Diabetes 1.04	Depressive disorders 1.25	Blindness and vision loss 0.55	COPD 1.14	Road injuries 1.31	Osteoarthritis 1.06	Conflict & terror 62.24	Stroke 0.9
Comoros	Age-related hearing loss 0.94	Low back pain 1.21	Blindness and vision loss 0.95	Diabetes 0.85	Road injuries 2.73	Depressive disorders 1.57	Stroke 1	Osteoarthritis 1.13	COPD 0.99	Alzheimer's disease 0.92
Djibouti	Age-related hearing loss 0.92	Diabetes 0.97	Low back pain 1.11	Blindness and vision loss 0.93	Depressive disorders 1.67	Stroke 1.16	Road injuries 2.1	Osteoarthritis 1.16	COPD 1	Ischemic heart disease 1.15
Eritrea	Blindness and vision loss 1.1	Age-related hearing loss 0.94	Diabetes 0.99	Low back pain 1.01	Conflict & terror 225.03	Depressive disorders 1.6	Road injuries 1.76	COPD 1.16	Osteoarthritis 1.07	Stroke 0.88
Ethiopia	Blindness and vision loss 1.13	Age-related hearing loss 1.01	Low back pain 1.02	Diabetes 0.81	Depressive disorders 1.6	Osteoarthritis 1.14	Road injuries 1.22	Alzheimer's disease 1.01	COPD 0.73	Ischemic heart disease 1.07
Kenya	Age-related hearing loss 1.21	Blindness and vision loss 1.14	Low back pain 1.25	Diabetes 0.85	Depressive disorders 2.08	COPD 1.16	Osteoarthritis 1.23	Stroke 1.01	Ischemic heart disease 1.42	Road injuries 1.89
Madagascar	Age-related hearing loss 1.01	Low back pain 1.15	Diabetes 0.88	Blindness and vision loss 0.79	Depressive disorders 1.63	COPD 1.36	Stroke 1.26	Road injuries 1.61	Osteoarthritis 1.09	Alzheimer's disease 0.93
Malawi	Diabetes 1.27	Blindness and vision loss 1.08	Age-related hearing loss 0.95	Low back pain 1.1	Depressive disorders 1.21	COPD 1.08	Stroke 1.01	Osteoarthritis 1.12	Road injuries 1.47	Ischemic heart disease 1.29
Mozambique	Diabetes 1.25	Age-related hearing loss 0.96	Blindness and vision loss 1.01	Low back pain 1.12	Road injuries 2.06	Depressive disorders 1.41	Stroke 1.41	COPD 1.01	Osteoarthritis 1.08	HIV/AIDS 23.91
Rwanda	Age-related hearing loss 0.94	Low back pain 1.21	Diabetes 0.94	Blindness and vision loss 0.68	Depressive disorders 1.72	Road injuries 2.33	COPD 1.3	Osteoarthritis 1.12	Stroke 0.89	Conflict & terror 125.03
Somalia	Age-related hearing loss 1.03	Low back pain 1.02	Blindness and vision loss 0.79	Diabetes 1.34	Depressive disorders 0.8	Road injuries 0.77	Osteoarthritis 1.04	COPD 0.92	Stroke 0.85	Dietary iron deficiency 1
South Sudan	Onchocerciasis 51.47	Blindness and vision loss 1.12	Age-related hearing loss 0.94	Low back pain 1.12	Diabetes 0.98	Depressive disorders 1.38	Road injuries 1.74	Stroke 1.2	Osteoarthritis 1.08	COPD 0.91
Uganda	Diabetes 1.27	Low back pain 1.16	Depressive disorders 2.47	Age-related hearing loss 0.71	Blindness and vision loss 0.72	Road injuries 2.02	COPD 1.1	Stroke 1.06	Osteoarthritis 1.11	Alzheimer's disease 0.94
United Republic of Tanzania	Blindness and vision loss 1.24	Age-related hearing loss 0.88	Low back pain 1.13	Diabetes 0.87	Depressive disorders 1.59	Stroke 1.13	Road injuries 1.68	Osteoarthritis 1.15	Alzheimer's disease 0.88	COPD 0.76
Zambia	Age-related hearing loss 0.92	Diabetes 1.03	Blindness and vision loss 0.96	Low back pain 1.15	Depressive disorders 1.68	COPD 1.13	Osteoarthritis 1.15	Stroke 0.88	Road injuries 1.59	Alzheimer's disease 0.88
**Southern Sub-Saharan Africa**	Diabetes 1.17	Age-related hearing loss 1.07	Blindness and vision loss 1.51	COPD 2.08	Low back pain 0.9	Osteoarthritis 1.33	Stroke 1.1	Depressive disorders 1.45	Oral disorders 0.78	Alzheimer's disease 0.86
Botswana	Diabetes 1.19	Blindness and vision loss 1.66	Age-related hearing loss 0.96	COPD 2.49	Low back pain 0.98	Stroke 1.23	Osteoarthritis 1.27	HIV/AIDS 558.14	Depressive disorders 1.61	Endo/metab/blood/immun 3.22
Eswatini	Diabetes 1.28	Age-related hearing loss 0.95	Blindness and vision loss 1.29	COPD 2.7	Low back pain 0.97	HIV/AIDS 427.89	Depressive disorders 1.68	Stroke 1.09	Osteoarthritis 1.36	Oral disorders 0.72
Lesotho	Blindness and vision loss 1.26	Diabetes 1.09	Age-related hearing loss 0.98	COPD 2.62	Low back pain 0.98	HIV/AIDS 198.63	Depressive disorders 1.76	Osteoarthritis 1.3	Stroke 1.02	Oral disorders 0.82
Namibia	Age-related hearing loss 0.98	Diabetes 0.92	Blindness and vision loss 1.34	COPD 2.69	Low back pain 1.03	Stroke 1.15	Osteoarthritis 1.24	Oral disorders 0.84	Depressive disorders 1.38	Alzheimer's disease 0.87
South Africa	Diabetes 1.17	Age-related hearing loss 1.1	Blindness and vision loss 1.61	COPD 2.12	Low back pain 0.86	Osteoarthritis 1.35	Stroke 1.12	Depressive disorders 1.44	Oral disorders 0.78	Alzheimer's disease 0.85
Zimbabwe	Diabetes 1.19	Blindness and vision loss 1.14	Age-related hearing loss 0.94	Low back pain 1.13	COPD 1.43	Depressive disorders 1.45	Osteoarthritis 1.26	Stroke 0.95	Oral disorders 0.78	Alzheimer's disease 0.95
**Western Sub-Saharan Africa**	Blindness and vision loss 1.22	Age-related hearing loss 0.94	Diabetes 0.95	Low back pain 1.08	Depressive disorders 1.56	Road injuries 2.02	Osteoarthritis 1.17	Stroke 0.88	COPD 0.91	Alzheimer's disease 0.78
Benin	Diabetes 1.25	Age-related hearing loss 0.94	Blindness and vision loss 0.99	Low back pain 1.03	Depressive disorders 1.41	Road injuries 1.9	Osteoarthritis 1.16	COPD 1.06	Stroke 0.98	Oral disorders 0.82
Burkina Faso	Blindness and vision loss 1.02	Age-related hearing loss 0.98	Diabetes 1.25	Low back pain 0.98	Depressive disorders 1.4	Road injuries 1.64	Osteoarthritis 1.11	Alzheimer's disease 0.94	COPD 0.72	Stroke 0.73
Cabo Verde	Diabetes 1.24	Age-related hearing loss 0.93	Blindness and vision loss 0.81	Low back pain 1.05	Depressive disorders 2.05	Alzheimer's disease 0.83	Stroke 1.02	Osteoarthritis 1.19	Road injuries 1.96	COPD 0.92
Cameroon	Diabetes 1.09	Age-related hearing loss 0.9	Low back pain 1.13	Blindness and vision loss 0.83	Road injuries 3.39	Depressive disorders 1.92	COPD 1.28	Osteoarthritis 1.22	Stroke 0.93	Onchocerciasis 68.25
Chad	Blindness and vision loss 0.99	Age-related hearing loss 0.97	Diabetes 1.24	Low back pain 1.04	Depressive disorders 1.38	Road injuries 1.44	Osteoarthritis 1.06	Stroke 0.92	COPD 0.88	Oral disorders 1.12
Côte d'Ivoire	Diabetes 1.13	Blindness and vision loss 1.04	Age-related hearing loss 0.93	Low back pain 1.05	Road injuries 2.19	Depressive disorders 1.45	Stroke 1.04	Osteoarthritis 1.15	COPD 0.97	Oral disorders 0.86
Gambia	Age-related hearing loss 0.94	Blindness and vision loss 0.98	Diabetes 1.05	Low back pain 1	Depressive disorders 1.37	Road injuries 1.88	COPD 1.14	Osteoarthritis 1.15	Stroke 1.01	Tuberculosis 4.12
Ghana	Diabetes 1.16	Age-related hearing loss 0.88	Blindness and vision loss 1.02	Low back pain 0.86	Depressive disorders 2.15	Road injuries 3	Stroke 1.01	COPD 1.19	Osteoarthritis 1.18	Alzheimer's disease 0.75
Guinea	Blindness and vision loss 1.08	Age-related hearing loss 0.96	Diabetes 1.08	Low back pain 1.02	Road injuries 1.78	Depressive disorders 1.22	COPD 1.12	Osteoarthritis 1.11	Stroke 0.89	Alzheimer's disease 0.88
Guinea-Bissau	Blindness and vision loss 1.13	Age-related hearing loss 0.94	Diabetes 1.16	Low back pain 1	Depressive disorders 1.29	Road injuries 1.77	COPD 1.11	Stroke 1.01	Osteoarthritis 1.11	Oral disorders 0.83
Liberia	Diabetes 1.45	Age-related hearing loss 0.94	Blindness and vision loss 0.93	Onchocerciasis 45.99	Low back pain 0.99	Depressive disorders 1.07	Road injuries 1.49	Osteoarthritis 1.16	Stroke 0.81	Alzheimer's disease 0.85
Mali	Blindness and vision loss 1.4	Age-related hearing loss 0.98	Diabetes 1.06	Low back pain 0.89	COPD 1.18	Road injuries 1.32	Depressive disorders 0.85	Osteoarthritis 1.09	Alzheimer's disease 0.94	Stroke 0.79
Mauritania	Age-related hearing loss 0.93	Blindness and vision loss 1.01	Low back pain 1.1	Diabetes 0.64	Depressive disorders 1.76	Road injuries 2.51	Osteoarthritis 1.23	Stroke 0.95	COPD 0.95	Oral disorders 0.76
Niger	Age-related hearing loss 1	Blindness and vision loss 0.96	Low back pain 0.95	Diabetes 0.89	Depressive disorders 0.87	Road injuries 0.98	Osteoarthritis 1.06	COPD 0.92	Stroke 0.84	Diarrheal diseases 1.04
Nigeria	Blindness and vision loss 1.45	Age-related hearing loss 0.94	Low back pain 1.19	Diabetes 0.78	Depressive disorders 1.8	Road injuries 2.23	Osteoarthritis 1.21	Stroke 0.83	COPD 0.76	Alzheimer's disease 0.71
Sao Tome and Principe	Diabetes 1.14	COPD 2.84	Age-related hearing loss 0.91	Blindness and vision loss 0.99	Low back pain 0.96	Stroke 1.24	Road injuries 2.46	Falls 2.2	Osteoarthritis 1.18	Depressive disorders 1.23
Senegal	Diabetes 1.53	Blindness and vision loss 1.09	Age-related hearing loss 0.94	Low back pain 0.93	Depressive disorders 1.56	Road injuries 1.77	COPD 1.08	Osteoarthritis 1.14	Stroke 0.88	Oral disorders 0.91
Sierra Leone	Age-related hearing loss 1.01	Blindness and vision loss 0.92	Low back pain 1.01	Diabetes 0.68	Depressive disorders 1.3	Road injuries 1.68	Onchocerciasis 16.64	Stroke 1.01	Osteoarthritis 1.12	COPD 0.99
Togo	Age-related hearing loss 0.93	Blindness and vision loss 1.03	Low back pain 1.03	Diabetes 0.83	Depressive disorders 1.62	COPD 1.21	Road injuries 1.88	Stroke 1.09	Osteoarthritis 1.14	Oral disorders 0.75

Values shown in brackets represent the ratio of observed YLDs to predicted YLDs on the basis of Socio-Demographic Index (SDI), rounded to two (2) digits. Color ranges (shown below) were calculated to place a roughly equal number of cells into each bin.

COLOR KEY:	[0.00,0.84)	[0.84,0.93)	[0.93,0.98)	[0.98,1.04)	[1.04,1.12)	[1.12,1.18)	[1.18,1.29)	[1.29,1.5)	[1.5,1.93)	[1.93,...)

Figure S23. Leading ten causes of total years lived with disability with ratio of observed to expected years lived with disability in 2019, among people 70 years and older, in females, for all GBD locations.

Causes are ranked based on global estimates of YLDs. Causes are color coded (shown in color key) based on the ratio of observed to expected (O:E) rates on that basis of SDI from the epidemiologic transition analysis. Shades of blue represent much lower observed deaths and YLDs than expected levels based on SDI, whereas red shows that observed deaths and YLDs exceed expected levels. When the ratio exceeds 1 means that observed levels exceeded the expected levels on the basis of SDI. O:E ratios are listed in each cell. YLDs = years of life lived with disability.

Figure 23. Leading 10 causes of total YLDs with ratio of observed to expected deaths in 2019, by location, females

	1	2	3	4	5	6	7	8	9	10
**Global**	Age-related hearing loss 1.09	Low back pain 0.99	Diabetes 0.92	Blindness and vision loss 1.31	COPD 1.99	Stroke 1.21	Alzheimer's disease 1.05	Falls 1.24	Osteoarthritis 1.06	Oral disorders 0.92
**Central Europe, Eastern Europe, and Central Asia**	Low back pain 1.17	Age-related hearing loss 1.22	Diabetes 0.92	Alzheimer's disease 1.11	Blindness and vision loss 1.5	Stroke 1.2	Oral disorders 1.19	Osteoarthritis 0.95	Falls 0.91	Depressive disorders 1.13
**Central Asia**	Age-related hearing loss 1.02	Low back pain 1.02	Diabetes 0.95	Blindness and vision loss 1.25	Oral disorders 1.28	Alzheimer's disease 1.13	Depressive disorders 1.36	Stroke 0.91	Osteoarthritis 1	COPD 1.04
Armenia	Age-related hearing loss 1.03	Low back pain 1.12	Diabetes 0.97	Blindness and vision loss 1.29	Alzheimer's disease 1.14	Oral disorders 1.29	Depressive disorders 1.3	Osteoarthritis 0.96	COPD 1.45	Stroke 0.68
Azerbaijan	Age-related hearing loss 1.02	Low back pain 1.04	Diabetes 0.91	Blindness and vision loss 1.32	Oral disorders 1.25	Alzheimer's disease 1.12	Stroke 0.97	Osteoarthritis 1.06	Depressive disorders 1.16	COPD 1.13
Georgia	Age-related hearing loss 1.05	Diabetes 1.04	Low back pain 0.95	Blindness and vision loss 1.39	Alzheimer's disease 1.11	Oral disorders 1.2	Depressive disorders 1.46	Osteoarthritis 1.03	Stroke 0.88	Falls 1.07
Kazakhstan	Diabetes 1.28	Age-related hearing loss 1.08	Low back pain 0.99	Blindness and vision loss 1.51	Oral disorders 1.34	Stroke 1.12	Alzheimer's disease 1.08	Depressive disorders 1.43	Osteoarthritis 0.92	COPD 1.19
Kyrgyzstan	Age-related hearing loss 0.97	Low back pain 1.07	Blindness and vision loss 0.93	Alzheimer's disease 1.21	Oral disorders 1.31	Depressive disorders 1.47	Diabetes 0.53	Osteoarthritis 1.08	COPD 1.46	Stroke 0.63
Mongolia	Age-related hearing loss 0.95	Low back pain 1.06	Blindness and vision loss 1.09	Oral disorders 1.25	Depressive disorders 1.46	Alzheimer's disease 1.18	Osteoarthritis 1.07	Stroke 0.69	Diabetes 0.34	Falls 1.29
Tajikistan	Age-related hearing loss 0.98	Low back pain 1.03	Diabetes 0.95	Blindness and vision loss 0.91	Oral disorders 1.39	Alzheimer's disease 1.23	Depressive disorders 1.22	Osteoarthritis 1.06	Stroke 0.49	Ischemic heart disease 1.21
Turkmenistan	Age-related hearing loss 1	Low back pain 1.04	Blindness and vision loss 1.33	Alzheimer's disease 1.16	Diabetes 0.72	Oral disorders 1.23	Stroke 0.94	Depressive disorders 1.36	Osteoarthritis 0.88	Ischemic heart disease 1.14
Uzbekistan	Age-related hearing loss 0.96	Low back pain 1.04	Diabetes 0.75	Blindness and vision loss 1.08	Oral disorders 1.25	Stroke 0.92	Depressive disorders 1.29	Osteoarthritis 1.05	Alzheimer's disease 1.15	Other musculoskeletal 1.39
**Central Europe**	Age-related hearing loss 1.21	Diabetes 1.32	Low back pain 0.94	Alzheimer's disease 1.17	Falls 1.24	Stroke 1.35	Oral disorders 1.11	Blindness and vision loss 1.15	Osteoarthritis 0.71	Depressive disorders 1
Albania	Age-related hearing loss 1.02	Low back pain 1.02	Alzheimer's disease 1.27	Diabetes 0.62	Oral disorders 1.13	Stroke 0.94	Blindness and vision loss 0.79	Falls 1.42	Osteoarthritis 0.84	Depressive disorders 0.9
Bosnia and Herzegovina	Diabetes 1.5	Age-related hearing loss 1.06	Stroke 1.65	Low back pain 0.87	Alzheimer's disease 1.17	Oral disorders 1.22	Falls 1.32	Blindness and vision loss 0.9	Depressive disorders 1.16	Osteoarthritis 0.77
Bulgaria	Age-related hearing loss 1.15	Diabetes 1.11	Low back pain 0.91	Stroke 1.73	Alzheimer's disease 1.17	Oral disorders 1.07	Depressive disorders 1.25	Blindness and vision loss 1.07	Falls 0.88	Osteoarthritis 0.73
Croatia	Falls 2.4	Diabetes 1.42	Age-related hearing loss 1.17	Low back pain 0.95	Alzheimer's disease 1.17	Stroke 1.23	Oral disorders 1.1	Depressive disorders 1.37	Blindness and vision loss 1.18	Osteoarthritis 0.68
Czechia	Diabetes 2.23	Age-related hearing loss 1.22	Low back pain 0.87	Falls 1.3	Alzheimer's disease 1.16	Stroke 1.2	Oral disorders 1.15	Blindness and vision loss 1.35	Depressive disorders 1.22	Osteoarthritis 0.64
Hungary	Diabetes 1.34	Age-related hearing loss 1.18	Falls 1.64	Low back pain 0.87	Alzheimer's disease 1.17	Stroke 1.32	Oral disorders 1.09	Blindness and vision loss 1.23	Depressive disorders 1.17	Osteoarthritis 0.7
Montenegro	Diabetes 1.69	Age-related hearing loss 1.19	Low back pain 0.89	Stroke 1.41	Alzheimer's disease 1.18	Oral disorders 1.2	Falls 0.99	Blindness and vision loss 1.22	Depressive disorders 1.24	Osteoarthritis 0.69
North Macedonia	Diabetes 1.73	Stroke 1.96	Age-related hearing loss 1.11	Low back pain 0.86	Oral disorders 1.19	Alzheimer's disease 1.19	Falls 1.32	Blindness and vision loss 1.02	Depressive disorders 1.1	Osteoarthritis 0.74
Poland	Age-related hearing loss 1.31	Diabetes 1.34	Low back pain 0.97	Alzheimer's disease 1.18	Falls 1.11	Stroke 1.13	Oral disorders 1.09	Blindness and vision loss 1.17	Osteoarthritis 0.71	Ischemic heart disease 1.69
Romania	Age-related hearing loss 1.14	Low back pain 1	Stroke 1.51	Alzheimer's disease 1.18	Diabetes 0.86	Oral disorders 1.06	Blindness and vision loss 1.08	Falls 0.92	Osteoarthritis 0.74	Depressive disorders 1.02
Serbia	Diabetes 1.53	Age-related hearing loss 1.14	Stroke 1.85	Low back pain 0.92	Alzheimer's disease 1.13	Oral disorders 1.17	Falls 1.13	Depressive disorders 1.28	Blindness and vision loss 1.1	Osteoarthritis 0.72
Slovakia	Age-related hearing loss 1.22	Low back pain 0.86	Diabetes 1.16	Falls 1.35	Alzheimer's disease 1.17	Stroke 1.37	Oral disorders 1.05	Blindness and vision loss 1.3	Depressive disorders 1.18	Osteoarthritis 0.67
Slovenia	Falls 1.72	Age-related hearing loss 1.14	Alzheimer's disease 1.16	Low back pain 0.82	Diabetes 1.23	Oral disorders 1.32	Depressive disorders 1.48	Blindness and vision loss 1.37	Stroke 0.87	Osteoarthritis 0.64
**Eastern Europe**	Low back pain 1.33	Age-related hearing loss 1.26	Blindness and vision loss 1.77	Alzheimer's disease 1.08	Osteoarthritis 1.1	Oral disorders 1.23	Stroke 1.15	Diabetes 0.67	Depressive disorders 1.18	Falls 0.71
Belarus	Low back pain 1.22	Age-related hearing loss 1.15	Alzheimer's disease 1.14	Blindness and vision loss 1.39	Stroke 1.03	Depressive disorders 1.45	Osteoarthritis 0.97	Oral disorders 1.02	Falls 1.05	Diabetes 0.53
Estonia	Age-related hearing loss 1.33	Low back pain 1.06	Alzheimer's disease 1.12	Diabetes 0.89	Depressive disorders 1.77	Blindness and vision loss 1.62	Osteoarthritis 0.83	Oral disorders 1.06	Falls 0.56	Stroke 0.74
Latvia	Low back pain 1.15	Age-related hearing loss 1.3	Alzheimer's disease 1.08	Blindness and vision loss 1.88	Diabetes 0.88	Stroke 1.2	Depressive disorders 1.54	Osteoarthritis 0.85	Falls 0.68	Oral disorders 0.93
Lithuania	Low back pain 1.11	Age-related hearing loss 1.34	Alzheimer's disease 1.08	Blindness and vision loss 2.06	Falls 0.82	Stroke 1.14	Depressive disorders 1.57	Osteoarthritis 0.7	Diabetes 0.67	Oral disorders 0.96
Republic of Moldova	Low back pain 1.23	Age-related hearing loss 1.07	Blindness and vision loss 1.37	Alzheimer's disease 1.15	Diabetes 0.72	Osteoarthritis 1.06	Oral disorders 1.02	Stroke 0.79	Depressive disorders 1.12	Falls 1
Russian Federation	Low back pain 1.35	Age-related hearing loss 1.3	Blindness and vision loss 2	Osteoarthritis 1.18	Alzheimer's disease 1.06	Stroke 1.25	Oral disorders 1.27	Diabetes 0.72	Falls 0.68	Road injuries 4
Ukraine	Low back pain 1.35	Age-related hearing loss 1.18	Alzheimer's disease 1.11	Blindness and vision loss 1.34	Oral disorders 1.21	Depressive disorders 1.53	Stroke 0.95	Diabetes 0.57	Osteoarthritis 0.92	Ischemic heart disease 1.87
**High-income**	Low back pain 1.07	Age-related hearing loss 1.11	Falls 0.99	Alzheimer's disease 1.09	Osteoarthritis 1.2	Diabetes 1.16	COPD 1.32	Stroke 1.08	Oral disorders 0.97	Other musculoskeletal 3.39
**Australasia**	Falls 1.46	Age-related hearing loss 1.15	Low back pain 0.82	Osteoarthritis 1.24	Alzheimer's disease 1.07	COPD 1.66	Diabetes 0.95	Oral disorders 1.26	Other musculoskeletal 3.76	Total cancers 1.4
Australia	Falls 1.44	Age-related hearing loss 1.15	Low back pain 0.81	Osteoarthritis 1.24	Alzheimer's disease 1.06	COPD 1.64	Diabetes 0.97	Oral disorders 1.24	Other musculoskeletal 4.23	Total cancers 1.37
New Zealand	Falls 1.56	Age-related hearing loss 1.17	Low back pain 0.85	Osteoarthritis 1.25	COPD 1.76	Alzheimer's disease 1.07	Oral disorders 1.35	Diabetes 0.86	Total cancers 1.56	Atrial fibrillation 1.68
**High-income Asia Pacific**	Low back pain 1.08	Age-related hearing loss 1.24	Alzheimer's disease 1.23	Osteoarthritis 1.32	Stroke 1.58	Other musculoskeletal 4.32	Diabetes 0.83	Falls 0.53	Oral disorders 0.93	Total cancers 0.72
Brunei Darussalam	Diabetes 2.43	Low back pain 0.95	Osteoarthritis 1.51	Stroke 1.97	Age-related hearing loss 1.11	Other musculoskeletal 3.55	COPD 1.47	Alzheimer's disease 0.98	Falls 0.74	Oral disorders 0.72
Japan	Low back pain 1.11	Alzheimer's disease 1.26	Age-related hearing loss 1.24	Osteoarthritis 1.28	Stroke 1.6	Other musculoskeletal 4.45	Falls 0.5	Diabetes 0.75	Oral disorders 0.96	Total cancers 0.74
Republic of Korea	Osteoarthritis 1.51	Low back pain 0.94	Age-related hearing loss 1.22	Diabetes 1.18	Alzheimer's disease 1.07	Stroke 1.48	Falls 0.72	Other musculoskeletal 3.72	Oral disorders 0.78	COPD 0.62
Singapore	Osteoarthritis 1.44	Age-related hearing loss 1.21	Low back pain 0.79	Diabetes 1.09	Alzheimer's disease 1.01	Stroke 1.34	Other musculoskeletal 4.44	Falls 0.47	Oral disorders 0.76	Blindness and vision loss 1.08
**High-income North America**	Low back pain 1.16	COPD 2.31	Age-related hearing loss 1.39	Falls 1.11	Diabetes 1.44	Osteoarthritis 1.31	Alzheimer's disease 1.08	Stroke 1.42	Other musculoskeletal 4.69	Total cancers 1.24
Canada	Falls 1.15	Low back pain 0.9	Age-related hearing loss 1.19	Alzheimer's disease 1	Osteoarthritis 0.97	Other musculoskeletal 5.05	COPD 1.26	Diabetes 0.92	Total cancers 1.42	Stroke 0.97
Greenland	Falls 3.16	Low back pain 1.12	COPD 3.05	Age-related hearing loss 1.08	Stroke 1.51	Osteoarthritis 1.2	Alzheimer's disease 1.09	Diabetes 0.6	Other musculoskeletal 3.2	Total cancers 1.57
United States of America	Low back pain 1.2	COPD 2.45	Age-related hearing loss 1.41	Diabetes 1.5	Falls 1.1	Osteoarthritis 1.35	Alzheimer's disease 1.09	Stroke 1.48	Other musculoskeletal 4.65	Neck pain 1.81
**Southern Latin America**	Age-related hearing loss 0.98	Diabetes 0.95	Other musculoskeletal 6.37	Low back pain 0.82	Osteoarthritis 1.47	Alzheimer's disease 1.05	Oral disorders 1.01	Falls 1.04	Blindness and vision loss 0.68	Stroke 0.61
Argentina	Age-related hearing loss 0.94	Other musculoskeletal 6.47	Diabetes 0.88	Low back pain 0.84	Osteoarthritis 1.51	Alzheimer's disease 1.06	Oral disorders 1.02	Blindness and vision loss 0.67	Falls 0.97	Endo/metab/blood/immun 1.66
Chile	Diabetes 1.26	Age-related hearing loss 1.08	Other musculoskeletal 6.13	Osteoarthritis 1.38	Low back pain 0.79	Alzheimer's disease 1.02	Falls 1.11	Oral disorders 0.99	Depressive disorders 0.96	Blindness and vision loss 0.75
Uruguay	Age-related hearing loss 0.97	Low back pain 0.85	Other musculoskeletal 6.31	Osteoarthritis 1.54	Alzheimer's disease 1.07	Oral disorders 1.06	Diabetes 0.57	Falls 1.33	Stroke 0.77	Blindness and vision loss 0.54
**Western Europe**	Low back pain 1.03	Falls 1.16	Diabetes 1.2	Alzheimer's disease 1.01	Age-related hearing loss 0.9	Osteoarthritis 1.05	COPD 1.21	Oral disorders 1.07	Depressive disorders 1.2	Blindness and vision loss 1.05
Andorra	Falls 1.52	Low back pain 0.96	Alzheimer's disease 0.97	Osteoarthritis 0.96	Age-related hearing loss 0.89	Diabetes 1.08	COPD 1.07	Oral disorders 1.1	Depressive disorders 1.12	Stroke 0.69
Austria	Low back pain 0.94	Falls 1.17	Diabetes 1.14	Alzheimer's disease 0.97	Osteoarthritis 1.04	Age-related hearing loss 0.82	Oral disorders 1.2	Stroke 0.84	COPD 0.84	Depressive disorders 0.96
Belgium	Falls 1.71	Low back pain 0.99	Diabetes 1.21	Alzheimer's disease 0.96	Age-related hearing loss 0.88	COPD 1.46	Osteoarthritis 1.02	Oral disorders 1.25	Stroke 0.7	Total cancers 1.05
Cyprus	Diabetes 1.63	Low back pain 1.02	Falls 1.25	Osteoarthritis 1.04	Age-related hearing loss 0.82	Alzheimer's disease 0.99	Oral disorders 1.12	COPD 1.13	Total cancers 1.13	Endo/metab/blood/immun 1.26
Denmark	Low back pain 1.12	COPD 1.8	Falls 0.89	Osteoarthritis 1.03	Alzheimer's disease 0.9	Age-related hearing loss 0.8	Diabetes 0.84	Oral disorders 1.07	Depressive disorders 1.2	Total cancers 0.96
Finland	Falls 1.43	Low back pain 0.89	Diabetes 1.42	Age-related hearing loss 1.03	Alzheimer's disease 0.96	Osteoarthritis 1.02	Oral disorders 1.16	Stroke 0.9	Depressive disorders 1.01	Neck pain 1.54
France	Falls 1.61	Low back pain 1.08	Alzheimer's disease 0.99	Age-related hearing loss 0.87	Osteoarthritis 1.07	Oral disorders 1.09	Depressive disorders 1.16	Diabetes 0.56	Total cancers 1.05	Stroke 0.6
Germany	Low back pain 1.04	Diabetes 1.64	Falls 0.9	Alzheimer's disease 1.02	COPD 1.27	Age-related hearing loss 0.9	Osteoarthritis 0.89	Oral disorders 1.1	Stroke 0.83	Depressive disorders 1.09
Greece	Low back pain 1.01	COPD 2	Age-related hearing loss 0.86	Alzheimer's disease 1.01	Diabetes 0.97	Osteoarthritis 0.98	Depressive disorders 1.63	Oral disorders 1.1	Falls 0.79	Stroke 0.8
Iceland	Low back pain 1.01	Osteoarthritis 1.28	COPD 1.73	Falls 0.86	Alzheimer's disease 0.98	Age-related hearing loss 0.86	Diabetes 1	Oral disorders 1.11	Blindness and vision loss 1.14	Stroke 0.7
Ireland	Low back pain 1.06	COPD 1.75	Falls 0.86	Osteoarthritis 1.01	Alzheimer's disease 0.98	Diabetes 1.04	Age-related hearing loss 0.86	Oral disorders 1.23	Depressive disorders 1.18	Total cancers 1.11
Israel	Low back pain 1.06	Diabetes 1.15	Osteoarthritis 1.23	Age-related hearing loss 0.83	Alzheimer's disease 0.99	Oral disorders 1.2	Falls 0.88	Depressive disorders 1.45	COPD 1.04	Stroke 0.62
Italy	Low back pain 1.04	Diabetes 1.23	Alzheimer's disease 1.17	Age-related hearing loss 0.97	Falls 1.2	Osteoarthritis 1.1	Blindness and vision loss 1.27	Oral disorders 1.07	COPD 1.04	Depressive disorders 1.18
Luxembourg	Low back pain 1.09	Falls 1.01	Diabetes 1.7	COPD 1.39	Osteoarthritis 0.95	Alzheimer's disease 0.82	Age-related hearing loss 0.83	Oral disorders 1.19	Depressive disorders 1.03	Total cancers 0.81
Malta	Low back pain 1.19	Diabetes 1.44	Falls 1.38	Osteoarthritis 1.14	Age-related hearing loss 0.79	Alzheimer's disease 0.99	Oral disorders 0.95	Endo/metab/blood/immun 1.52	COPD 0.85	Depressive disorders 0.89
Monaco	Low back pain 0.96	Falls 0.7	Alzheimer's disease 0.95	Osteoarthritis 0.97	Age-related hearing loss 0.9	Diabetes 1.1	COPD 1.08	Total cancers 1.42	Oral disorders 1.02	Stroke 0.93
Netherlands	Falls 1.29	Low back pain 0.89	COPD 1.74	Alzheimer's disease 1.03	Osteoarthritis 0.91	Age-related hearing loss 0.84	Oral disorders 1.35	Diabetes 0.97	Total cancers 1.15	Depressive disorders 1.05
Norway	Falls 1.22	Low back pain 0.84	Age-related hearing loss 1.21	Osteoarthritis 1.06	Diabetes 1.37	Alzheimer's disease 0.94	COPD 1.18	Oral disorders 1.2	Stroke 1.12	Total cancers 0.85
Portugal	Low back pain 1.28	Diabetes 1.28	Osteoarthritis 1.37	Alzheimer's disease 1.02	Age-related hearing loss 0.73	Depressive disorders 1.66	COPD 1.63	Oral disorders 1.05	Falls 1.07	Stroke 0.72
San Marino	Low back pain 0.97	Alzheimer's disease 0.97	Falls 0.74	Diabetes 1.2	Osteoarthritis 0.99	Age-related hearing loss 0.89	COPD 1.15	Oral disorders 1.12	Depressive disorders 1.26	Total cancers 1.06
Spain	Diabetes 1.27	Low back pain 0.91	Age-related hearing loss 0.82	Osteoarthritis 1.4	Alzheimer's disease 1	Falls 1.33	Blindness and vision loss 1.3	Depressive disorders 1.58	COPD 1.28	Oral disorders 0.78
Sweden	Low back pain 1.05	Falls 1.2	COPD 1.72	Alzheimer's disease 0.87	Diabetes 0.96	Osteoarthritis 0.77	Oral disorders 1.06	Age-related hearing loss 0.61	Depressive disorders 1.26	Stroke 0.8
Switzerland	Falls 1.06	Low back pain 1.06	Alzheimer's disease 0.96	Diabetes 1.29	Osteoarthritis 0.88	Age-related hearing loss 0.88	COPD 0.84	Oral disorders 0.98	Depressive disorders 1.1	Total cancers 0.75
United Kingdom	Low back pain 1.09	Diabetes 1.34	Falls 1.06	Age-related hearing loss 1	COPD 1.63	Osteoarthritis 1.08	Alzheimer's disease 0.87	Oral disorders 1.09	Depressive disorders 1.1	Total cancers 1.22

Latin America and Caribbean	Diabetes 1.16	Age-related hearing loss 0.97	Blindness and vision loss 1.11	Low back pain 0.84	Oral disorders 1.25	Alzheimer's disease 0.93	Other musculoskeletal 3.53	Osteoarthritis 1.13	Depressive disorders 1.02	Falls 1.42
Andean Latin America	Age-related hearing loss 1.03	Blindness and vision loss 1.24	Diabetes 0.89	Oral disorders 1.42	Low back pain 0.78	Osteoarthritis 1.21	Alzheimer's disease 0.83	Other musculoskeletal 3.16	Depressive disorders 0.87	Endo/metab/blood/immun 1.5
Bolivia (Plurinational State of)	Age-related hearing loss 1.03	Diabetes 0.96	Blindness and vision loss 1	Oral disorders 1.48	Low back pain 0.81	Osteoarthritis 1.23	Depressive disorders 1.24	Other musculoskeletal 2.01	Alzheimer's disease 0.86	Endo/metab/blood/immun 1.46
Ecuador	Diabetes 1.2	Age-related hearing loss 1.06	Oral disorders 1.32	Blindness and vision loss 0.92	Low back pain 0.74	Osteoarthritis 1.42	Other musculoskeletal 3.29	Alzheimer's disease 0.85	Depressive disorders 1.01	Endo/metab/blood/immun 1.52
Peru	Age-related hearing loss 1.02	Blindness and vision loss 1.46	Oral disorders 1.45	Diabetes 0.72	Low back pain 0.78	Alzheimer's disease 0.82	Osteoarthritis 1.11	Other musculoskeletal 3.6	Depressive disorders 0.71	Endo/metab/blood/immun 1.5
Caribbean	Diabetes 1.42	Age-related hearing loss 1.12	Low back pain 0.87	Blindness and vision loss 0.94	Falls 1.53	Oral disorders 1.06	Depressive disorders 1.24	Osteoarthritis 1	Alzheimer's disease 0.78	Stroke 0.49
Antigua and Barbuda	Diabetes 1.89	Age-related hearing loss 1.23	Low back pain 0.78	Blindness and vision loss 1.1	Oral disorders 0.99	Osteoarthritis 0.89	Alzheimer's disease 0.78	Depressive disorders 1.03	Other musculoskeletal 2.35	Chronic kidney disease 1.73
Bahamas	Diabetes 1.84	Age-related hearing loss 1.33	Low back pain 0.75	Blindness and vision loss 1.39	Osteoarthritis 0.83	Oral disorders 1.01	Alzheimer's disease 0.76	Depressive disorders 1.06	Other musculoskeletal 2.3	Stroke 0.54
Barbados	Diabetes 1.83	Age-related hearing loss 1.21	Low back pain 0.79	Oral disorders 1.01	Osteoarthritis 0.92	Alzheimer's disease 0.78	Depressive disorders 1.05	Other musculoskeletal 2.36	Blindness and vision loss 0.64	Stroke 0.53
Belize	Diabetes 1.26	Age-related hearing loss 1.04	Low back pain 0.89	Blindness and vision loss 0.69	Oral disorders 1	Osteoarthritis 1.17	Alzheimer's disease 0.83	Depressive disorders 0.99	Chronic kidney disease 1.44	Other musculoskeletal 1.52
Bermuda	Age-related hearing loss 1.36	Diabetes 1.3	Low back pain 0.77	Alzheimer's disease 0.81	Osteoarthritis 0.83	Blindness and vision loss 1.35	Oral disorders 1	Depressive disorders 1.2	Other musculoskeletal 2.55	Total cancers 0.99
Cuba	Age-related hearing loss 1.08	Diabetes 1.13	Falls 3.34	Low back pain 0.96	Blindness and vision loss 1.04	Depressive disorders 1.44	Oral disorders 1.02	Osteoarthritis 1.03	Alzheimer's disease 0.75	COPD 1.03
Dominica	Diabetes 1.87	Age-related hearing loss 1.19	Low back pain 0.81	Blindness and vision loss 1.09	Oral disorders 1.02	Alzheimer's disease 0.76	Osteoarthritis 0.92	Depressive disorders 1	Other musculoskeletal 2.53	Chronic kidney disease 1.69
Dominican Republic	Age-related hearing loss 1.04	Diabetes 0.79	Low back pain 0.81	Blindness and vision loss 0.76	Oral disorders 1.14	Depressive disorders 1.4	Osteoarthritis 1.15	Alzheimer's disease 0.82	Stroke 0.52	Other musculoskeletal 1.39
Grenada	Diabetes 1.65	Age-related hearing loss 1.09	Low back pain 0.85	Blindness and vision loss 0.83	Oral disorders 0.97	Osteoarthritis 1.01	Alzheimer's disease 0.78	Depressive disorders 0.96	Stroke 0.55	Other musculoskeletal 2.2
Guyana	Diabetes 1.75	Age-related hearing loss 1.03	Low back pain 0.86	Depressive disorders 1.44	Blindness and vision loss 0.73	Oral disorders 0.99	Osteoarthritis 1.08	Alzheimer's disease 0.77	Stroke 0.58	Falls 1.33
Haiti	Diabetes 2.06	Age-related hearing loss 1.08	Low back pain 0.89	Blindness and vision loss 0.65	Oral disorders 1.36	Depressive disorders 0.95	Osteoarthritis 1.05	Alzheimer's disease 0.84	Stroke 0.65	Endo/metab/blood/immun 1.24
Jamaica	Diabetes 1.7	Age-related hearing loss 1.11	Low back pain 0.86	Blindness and vision loss 0.87	Alzheimer's disease 0.79	Oral disorders 1.01	Osteoarthritis 1	Depressive disorders 0.99	Other musculoskeletal 2.41	Stroke 0.5
Puerto Rico	Diabetes 2.37	Age-related hearing loss 1.35	Low back pain 0.73	Alzheimer's disease 0.79	Osteoarthritis 0.85	Blindness and vision loss 1.33	Oral disorders 0.98	Falls 0.48	Depressive disorders 0.86	Chronic kidney disease 1.86
Saint Kitts and Nevis	Diabetes 1.82	Age-related hearing loss 1.22	Low back pain 0.79	Depressive disorders 1.38	Blindness and vision loss 1.09	Osteoarthritis 0.91	Oral disorders 0.98	Alzheimer's disease 0.77	Stroke 0.69	Other musculoskeletal 2.23
Saint Lucia	Diabetes 2.18	Age-related hearing loss 1.07	Low back pain 0.85	Blindness and vision loss 0.83	Oral disorders 0.97	Osteoarthritis 1.02	Alzheimer's disease 0.78	Depressive disorders 0.99	Stroke 0.56	Other musculoskeletal 2.32
Saint Vincent and the Grenadines	Diabetes 1.8	Age-related hearing loss 1.04	Low back pain 0.86	Blindness and vision loss 0.72	Oral disorders 0.98	Osteoarthritis 1.09	Alzheimer's disease 0.8	Depressive disorders 0.97	Stroke 0.55	Other musculoskeletal 2.06
Suriname	Diabetes 1.68	Age-related hearing loss 1.03	Low back pain 0.86	Blindness and vision loss 0.94	Depressive disorders 1.44	Oral disorders 0.96	Osteoarthritis 1.09	Alzheimer's disease 0.78	Stroke 0.53	Chronic kidney disease 1.39
Trinidad and Tobago	Diabetes 2.28	Age-related hearing loss 1.25	Low back pain 0.79	Blindness and vision loss 1.14	Osteoarthritis 0.89	Oral disorders 0.99	Alzheimer's disease 0.76	Depressive disorders 1.15	Other musculoskeletal 2.56	Stroke 0.56
United States Virgin Islands	Diabetes 1.99	Age-related hearing loss 1.35	Low back pain 0.75	Osteoarthritis 0.87	Blindness and vision loss 1.37	Depressive disorders 1.17	Oral disorders 0.84	Alzheimer's disease 0.77	Other musculoskeletal 2.29	Endo/metab/blood/immun 1.04
Central Latin America	Diabetes 1.41	Age-related hearing loss 1.04	Blindness and vision loss 1.07	Low back pain 0.85	Other musculoskeletal 3.8	Oral disorders 1.04	Osteoarthritis 1.19	Alzheimer's disease 0.82	Depressive disorders 0.94	Chronic kidney disease 1.8
Colombia	Diabetes 1.1	Age-related hearing loss 1.02	Blindness and vision loss 1.12	Low back pain 0.95	Other musculoskeletal 4.41	Alzheimer's disease 0.89	Osteoarthritis 1.07	Oral disorders 0.86	COPD 1.33	Endo/metab/blood/immun 1.68
Costa Rica	Diabetes 1.21	Age-related hearing loss 1.07	Blindness and vision loss 1.22	Low back pain 0.83	Other musculoskeletal 4.54	Alzheimer's disease 0.87	Oral disorders 1	Osteoarthritis 1.01	Falls 1.38	Depressive disorders 1.01
El Salvador	Diabetes 1.19	Age-related hearing loss 1.01	Blindness and vision loss 0.97	Low back pain 0.87	Alzheimer's disease 0.92	Other musculoskeletal 2.55	Oral disorders 1.03	Osteoarthritis 1.13	Depressive disorders 1	Falls 1.56
Guatemala	Diabetes 1.64	Blindness and vision loss 1.15	Age-related hearing loss 1.03	Low back pain 0.86	Oral disorders 1.23	Other musculoskeletal 1.95	Depressive disorders 1.36	Osteoarthritis 1.09	Alzheimer's disease 0.92	Chronic kidney disease 1.61
Honduras	Diabetes 1.49	Age-related hearing loss 1.05	Blindness and vision loss 0.81	Low back pain 0.89	Other musculoskeletal 1.94	Oral disorders 1.2	Depressive disorders 1.21	Osteoarthritis 1.11	Alzheimer's disease 0.92	Endo/metab/blood/immun 1.56
Mexico	Diabetes 1.6	Age-related hearing loss 1.06	Blindness and vision loss 1.05	Low back pain 0.79	Other musculoskeletal 4.31	Osteoarthritis 1.32	Oral disorders 1.12	Chronic kidney disease 2.3	Alzheimer's disease 0.75	Depressive disorders 1.03
Nicaragua	Diabetes 1.45	Age-related hearing loss 1.04	Blindness and vision loss 0.88	Low back pain 0.88	Other musculoskeletal 1.89	Oral disorders 1.16	Osteoarthritis 1.13	Alzheimer's disease 0.97	Depressive disorders 0.95	Falls 1.49
Panama	Diabetes 1.39	Age-related hearing loss 1.08	Blindness and vision loss 1.48	Low back pain 0.83	Other musculoskeletal 5.18	Alzheimer's disease 0.86	Oral disorders 0.99	Osteoarthritis 0.98	Depressive disorders 1	Endo/metab/blood/immun 1.6
Venezuela (Bolivarian Republic of)	Diabetes 1.23	Age-related hearing loss 1.01	Blindness and vision loss 1.03	Low back pain 0.86	Other musculoskeletal 3.55	Oral disorders 1.01	Alzheimer's disease 0.84	Osteoarthritis 1.03	Depressive disorders 1.07	Chronic kidney disease 1.48
Tropical Latin America	Diabetes 0.95	Age-related hearing loss 0.87	Blindness and vision loss 1.15	Oral disorders 1.45	Low back pain 0.83	Alzheimer's disease 1.09	Other musculoskeletal 3.8	Osteoarthritis 1.09	Depressive disorders 1.09	Falls 1.69
Brazil	Diabetes 0.95	Age-related hearing loss 0.86	Blindness and vision loss 1.15	Oral disorders 1.46	Low back pain 0.84	Alzheimer's disease 1.09	Other musculoskeletal 3.8	Osteoarthritis 1.09	Depressive disorders 1.09	Falls 1.7
Paraguay	Age-related hearing loss 1.05	Diabetes 0.95	Blindness and vision loss 1.09	Low back pain 0.8	Oral disorders 1.25	Alzheimer's disease 1.06	Other musculoskeletal 3.7	Osteoarthritis 1.06	Depressive disorders 1.1	Stroke 0.56
North Africa and Middle East	Diabetes 1.26	Age-related hearing loss 1.03	Blindness and vision loss 1.5	Low back pain 0.91	Stroke 1.29	Alzheimer's disease 1.17	Oral disorders 0.99	Osteoarthritis 0.9	COPD 1.52	Depressive disorders 1.01
North Africa and Middle East	Diabetes 1.26	Age-related hearing loss 1.03	Blindness and vision loss 1.5	Low back pain 0.91	Stroke 1.29	Alzheimer's disease 1.17	Oral disorders 0.99	Osteoarthritis 0.9	COPD 1.52	Depressive disorders 1.01
Afghanistan	Blindness and vision loss 1.43	Diabetes 1.91	Age-related hearing loss 1	Low back pain 0.9	Leishmaniasis 1472.36	Conflict & terror 249.67	Stroke 1.21	Oral disorders 1.44	Alzheimer's disease 1.22	COPD 1.17
Algeria	Diabetes 1.32	Blindness and vision loss 1.49	Age-related hearing loss 0.97	Stroke 1.34	Low back pain 0.88	Alzheimer's disease 1.17	Oral disorders 0.91	Osteoarthritis 0.96	Depressive disorders 0.99	Other musculoskeletal 2.65
Bahrain	Diabetes 3.13	Age-related hearing loss 1.11	Blindness and vision loss 2.12	Low back pain 0.78	Stroke 1.1	Alzheimer's disease 1.14	COPD 1.74	Osteoarthritis 0.82	Oral disorders 0.89	Depressive disorders 1.14
Egypt	Diabetes 1.05	Blindness and vision loss 1.64	Stroke 1.61	Age-related hearing loss 0.96	Low back pain 0.9	Oral disorders 0.91	Alzheimer's disease 1.16	Osteoarthritis 0.97	Depressive disorders 1.04	Chronic kidney disease 2.3
Iran (Islamic Republic of)	Diabetes 1.21	Blindness and vision loss 1.86	Age-related hearing loss 1.01	Low back pain 1.03	Alzheimer's disease 1.16	Stroke 1.05	Osteoarthritis 1.01	COPD 1.81	Depressive disorders 1.06	Oral disorders 0.78
Iraq	Diabetes 1.41	Blindness and vision loss 1.6	Age-related hearing loss 0.99	Stroke 1.64	Low back pain 0.85	Alzheimer's disease 1.14	Oral disorders 0.91	Osteoarthritis 0.92	Chronic kidney disease 2.21	Depressive disorders 0.8
Jordan	Diabetes 1.36	Stroke 1.88	Age-related hearing loss 1.08	Low back pain 0.82	Blindness and vision loss 1.66	Alzheimer's disease 1.13	Oral disorders 0.97	Depressive disorders 1.19	Osteoarthritis 0.86	Other musculoskeletal 2.9
Kuwait	Diabetes 2.4	Age-related hearing loss 1.33	Blindness and vision loss 2.94	Low back pain 0.8	Alzheimer's disease 1.14	Stroke 1.13	Osteoarthritis 0.71	Oral disorders 0.97	Other musculoskeletal 3.24	Depressive disorders 1.12
Lebanon	Diabetes 1.46	Age-related hearing loss 1.05	Blindness and vision loss 1.71	Stroke 1.31	Low back pain 0.75	Alzheimer's disease 1.15	COPD 2.16	Oral disorders 0.95	Osteoarthritis 0.85	Depressive disorders 1.11
Libya	Diabetes 1.64	Blindness and vision loss 1.88	Age-related hearing loss 1.05	Stroke 1.45	Low back pain 0.81	Alzheimer's disease 1.11	Oral disorders 0.93	Osteoarthritis 0.88	COPD 1.44	Depressive disorders 1.01
Morocco	Diabetes 1.21	Age-related hearing loss 0.97	Blindness and vision loss 0.92	Low back pain 0.95	Stroke 1.42	Alzheimer's disease 1.24	Oral disorders 1.1	Depressive disorders 1.19	Osteoarthritis 1.02	Chronic kidney disease 1.84
Oman	Diabetes 1.56	Blindness and vision loss 3.33	Age-related hearing loss 1.16	Low back pain 0.76	Stroke 1.58	Alzheimer's disease 1.14	Chronic kidney disease 3.3	Osteoarthritis 0.76	Oral disorders 0.92	Depressive disorders 1.1
Palestine	Diabetes 1.39	Age-related hearing loss 0.94	Blindness and vision loss 1.14	Stroke 1.39	Low back pain 0.87	Alzheimer's disease 1.19	Depressive disorders 1.37	Oral disorders 0.95	Osteoarthritis 1	Chronic kidney disease 1.93
Qatar	Diabetes 4.03	Blindness and vision loss 3.7	Low back pain 0.72	Age-related hearing loss 1.2	Stroke 1.44	Other musculoskeletal 2.52	Osteoarthritis 0.73	Oral disorders 0.88	Chronic kidney disease 3.08	Depressive disorders 1.08
Saudi Arabia	Blindness and vision loss 4.45	Diabetes 1.73	Stroke 2	Age-related hearing loss 1.2	Low back pain 0.73	Falls 1.07	Alzheimer's disease 1.1	Osteoarthritis 0.88	COPD 1.23	Oral disorders 0.89
Sudan	Blindness and vision loss 1.24	Age-related hearing loss 0.98	Diabetes 1.17	Stroke 1.53	Low back pain 0.87	Alzheimer's disease 1.24	Oral disorders 1.04	Osteoarthritis 1.01	Depressive disorders 0.87	Chronic kidney disease 1.73
Syrian Arab Republic	Diabetes 1.15	Blindness and vision loss 1.35	Age-related hearing loss 0.92	Low back pain 0.88	Conflict & terror 1226.76	Stroke 1.16	Oral disorders 0.95	Alzheimer's disease 1.19	Osteoarthritis 0.98	Depressive disorders 0.91
Tunisia	Diabetes 1.26	Age-related hearing loss 1.01	Blindness and vision loss 1.44	Low back pain 0.84	Alzheimer's disease 1.18	Stroke 1.09	Depressive disorders 1.45	Oral disorders 0.9	Osteoarthritis 0.92	Other musculoskeletal 2.96
Turkey	Diabetes 1.24	Age-related hearing loss 1.14	Low back pain 0.92	Alzheimer's disease 1.17	Blindness and vision loss 1.42	COPD 1.87	Stroke 1.12	Oral disorders 1.17	Osteoarthritis 0.74	Falls 0.86
United Arab Emirates	Diabetes 3.21	Stroke 2.84	Blindness and vision loss 4.15	Age-related hearing loss 1.33	Low back pain 0.65	Osteoarthritis 0.64	COPD 1.17	Oral disorders 0.94	Other musculoskeletal 2.34	Chronic kidney disease 3.44
Yemen	Blindness and vision loss 1.01	Age-related hearing loss 1.01	Diabetes 1.07	Low back pain 0.91	Stroke 1.37	Alzheimer's disease 1.28	Oral disorders 1.3	Depressive disorders 0.89	Osteoarthritis 0.95	COPD 0.95
South Asia	Blindness and vision loss 1.61	COPD 4.14	Age-related hearing loss 1	Diabetes 0.94	Falls 4.35	Low back pain 0.93	Dietary iron deficiency 3.52	Other musculoskeletal 2.3	Depressive disorders 1.23	Osteoarthritis 0.98
South Asia	Blindness and vision loss 1.61	COPD 4.14	Age-related hearing loss 1	Diabetes 0.94	Falls 4.35	Low back pain 0.93	Dietary iron deficiency 3.52	Other musculoskeletal 2.3	Depressive disorders 1.23	Osteoarthritis 0.98
Bangladesh	Blindness and vision loss 1.4	Age-related hearing loss 0.96	Low back pain 1.03	Other musculoskeletal 2.96	Diabetes 0.92	COPD 2.37	Depressive disorders 1.37	Dietary iron deficiency 1.86	Osteoarthritis 0.93	Stroke 0.73
Bhutan	COPD 3.49	Age-related hearing loss 0.98	Low back pain 1.06	Diabetes 1.01	Blindness and vision loss 0.77	Other musculoskeletal 2.39	Falls 2.95	Depressive disorders 1.2	Osteoarthritis 0.99	Dietary iron deficiency 1.72
India	Blindness and vision loss 1.64	COPD 4.5	Age-related hearing loss 1	Falls 4.97	Diabetes 0.92	Low back pain 0.91	Dietary iron deficiency 4.05	Other musculoskeletal 2.21	Depressive disorders 1.21	Osteoarthritis 1
Nepal	COPD 4.2	Blindness and vision loss 1.09	Low back pain 1.14	Age-related hearing loss 0.93	Diabetes 1.06	Depressive disorders 1.48	Falls 3.35	Other musculoskeletal 2.57	Dietary iron deficiency 1.92	Osteoarthritis 0.95
Pakistan	Blindness and vision loss 1.62	Diabetes 1.31	Age-related hearing loss 1	COPD 2.77	Low back pain 0.91	Other musculoskeletal 2.23	Depressive disorders 1.24	Osteoarthritis 0.89	Dietary iron deficiency 1.58	Stroke 0.68
Southeast Asia, East Asia, and Oceania	Age-related hearing loss 1.14	Stroke 1.91	Blindness and vision loss 1.4	COPD 3.22	Low back pain 0.92	Alzheimer's disease 1.16	Diabetes 0.63	Osteoarthritis 0.93	Oral disorders 0.86	Falls 1.28
East Asia	Age-related hearing loss 1.18	Stroke 2.04	COPD 3.57	Low back pain 0.86	Blindness and vision loss 1.23	Alzheimer's disease 1.22	Diabetes 0.52	Osteoarthritis 1	Oral disorders 0.86	Falls 1.3
China	Age-related hearing loss 1.19	Stroke 2.05	COPD 3.66	Low back pain 0.85	Blindness and vision loss 1.26	Alzheimer's disease 1.23	Diabetes 0.51	Osteoarthritis 1	Oral disorders 0.86	Falls 1.38
Democratic People's Republic of Korea	COPD 4.38	Age-related hearing loss 1.01	Stroke 1.96	Low back pain 1.16	Diabetes 0.65	Alzheimer's disease 1.07	Blindness and vision loss 0.48	Osteoarthritis 1.04	Oral disorders 0.85	Depressive disorders 0.87
Taiwan (Province of China)	Low back pain 1.05	Diabetes 1.61	Age-related hearing loss 1.28	Stroke 1.71	Alzheimer's disease 0.98	Chronic kidney disease 3.64	Osteoarthritis 0.71	Oral disorders 0.81	Other musculoskeletal 2.77	Blindness and vision loss 1.17
Oceania	Diabetes 1.61	Low back pain 1.32	Blindness and vision loss 1.09	Age-related hearing loss 1	Stroke 1.55	COPD 2.19	Oral disorders 1.18	Falls 2.07	Alzheimer's disease 0.97	Dietary iron deficiency 2.11
American Samoa	Diabetes 2.54	Low back pain 1.22	Stroke 1.85	Age-related hearing loss 1.03	Blindness and vision loss 1.43	Alzheimer's disease 0.88	Oral disorders 0.91	Osteoarthritis 0.82	COPD 1.48	Chronic kidney disease 2.12
Cook Islands	Diabetes 2.16	Low back pain 1.17	COPD 3	Age-related hearing loss 1.11	Stroke 1.79	Blindness and vision loss 2	Alzheimer's disease 0.89	Oral disorders 0.93	Osteoarthritis 0.75	Chronic kidney disease 2
Fiji	Diabetes 2.11	Low back pain 1.26	Blindness and vision loss 1.56	Age-related hearing loss 0.97	Stroke 1.63	Oral disorders 0.92	Alzheimer's disease 0.89	Osteoarthritis 0.85	Dietary iron deficiency 5.75	Other musculoskeletal 2.28
Guam	Low back pain 1.14	Diabetes 1.46	Stroke 2.08	Age-related hearing loss 1.21	Blindness and vision loss 2.07	Alzheimer's disease 0.89	Oral disorders 0.94	Osteoarthritis 0.69	Ischemic heart disease 2.06	COPD 0.82
Kiribati	Diabetes 1.45	Low back pain 1.35	Age-related hearing loss 0.98	Blindness and vision loss 0.95	Stroke 1.51	Oral disorders 1.12	Dietary iron deficiency 2.43	Osteoarthritis 0.92	Alzheimer's disease 0.96	Other musculoskeletal 1.02
Marshall Islands	Diabetes 2.39	Low back pain 1.27	Stroke 1.82	Age-related hearing loss 0.94	Blindness and vision loss 0.92	Oral disorders 1	Dietary iron deficiency 2.21	Osteoarthritis 0.89	Alzheimer's disease 0.93	Other musculoskeletal 1
Micronesia (Federated States of)	Diabetes 1.57	Low back pain 1.31	Age-related hearing loss 0.93	Stroke 1.47	Blindness and vision loss 0.95	Oral disorders 0.99	Alzheimer's disease 0.94	Osteoarthritis 0.92	COPD 1.49	Chronic kidney disease 1.74
Nauru	Diabetes 1.31	Low back pain 1.32	Stroke 1.87	Age-related hearing loss 0.94	COPD 3.8	Blindness and vision loss 1.03	Oral disorders 0.9	Osteoarthritis 0.9	Other musculoskeletal 1.73	Alzheimer's disease 0.91
Niue	Diabetes 2.15	Low back pain 1.22	Age-related hearing loss 1.02	COPD 3.25	Stroke 1.68	Blindness and vision loss 1.34	Alzheimer's disease 0.9	Oral disorders 0.94	Osteoarthritis 0.81	Chronic kidney disease 2.01
Northern Mariana Islands	Diabetes 1.75	Low back pain 1.15	Age-related hearing loss 1.16	Stroke 1.88	Blindness and vision loss 1.79	Alzheimer's disease 0.92	Oral disorders 0.93	Osteoarthritis 0.72	Falls 0.84	Chronic kidney disease 2.18
Palau	Diabetes 2.01	Low back pain 1.19	Stroke 1.9	Age-related hearing loss 1.09	COPD 3.18	Blindness and vision loss 1.58	Oral disorders 0.93	Osteoarthritis 0.77	Alzheimer's disease 0.89	Falls 1.08
Papua New Guinea	Low back pain 1.35	Diabetes 1.48	Blindness and vision loss 1.03	Age-related hearing loss 0.99	COPD 2.69	Stroke 1.44	Falls 3.16	Oral disorders 1.32	Dietary iron deficiency 1.8	Alzheimer's disease 1.01

Samoa	Diabetes 1.46	Low back pain 1.35	Age-related hearing loss 0.95	Stroke 1.64	Blindness and vision loss 1.12	Alzheimer's disease 0.92	Oral disorders 0.97	COPD 1.7	Osteoarthritis 0.89	Chronic kidney disease 1.74
Solomon Islands	Low back pain 1.34	Diabetes 1.36	Age-related hearing loss 1.01	Blindness and vision loss 0.85	Stroke 1.67	Falls 3.46	Oral disorders 1.34	COPD 1.19	Dietary iron deficiency 1.64	Osteoarthritis 0.89
Tokelau	Diabetes 1.44	Low back pain 1.31	Age-related hearing loss 0.92	COPD 3.83	Stroke 1.47	Blindness and vision loss 1.01	Alzheimer's disease 0.93	Oral disorders 0.94	Osteoarthritis 0.9	Chronic kidney disease 1.6
Tonga	Diabetes 1.34	Low back pain 1.34	Age-related hearing loss 0.94	Stroke 1.39	Blindness and vision loss 0.81	Alzheimer's disease 0.93	Oral disorders 0.96	Osteoarthritis 0.9	COPD 1.55	Ischemic heart disease 1.67
Tuvalu	Diabetes 1.56	Low back pain 1.32	Age-related hearing loss 0.93	Stroke 1.47	Blindness and vision loss 0.96	COPD 3.18	Oral disorders 0.98	Alzheimer's disease 0.94	Osteoarthritis 0.9	Dietary iron deficiency 2.68
Vanuatu	Diabetes 1.62	Low back pain 1.45	Stroke 2.11	Age-related hearing loss 0.97	Blindness and vision loss 0.71	Oral disorders 1.14	COPD 1.32	Dietary iron deficiency 1.92	Alzheimer's disease 0.98	Osteoarthritis 0.9
**Southeast Asia**	Blindness and vision loss 1.92	Low back pain 1.16	Diabetes 1.03	Age-related hearing loss 1	Stroke 1.45	COPD 1.89	Alzheimer's disease 0.91	Oral disorders 0.86	Other musculoskeletal 2.5	Neck pain 2
Cambodia	Blindness and vision loss 1.44	Diabetes 1.33	Age-related hearing loss 1	Low back pain 1.19	Stroke 1.4	Falls 2.39	Alzheimer's disease 1	Oral disorders 0.98	COPD 1.2	Other musculoskeletal 1.17
Indonesia	Blindness and vision loss 3.11	Stroke 1.93	Low back pain 1.26	Age-related hearing loss 1	Diabetes 0.7	Oral disorders 0.93	COPD 1.84	Alzheimer's disease 0.85	Other musculoskeletal 2.76	Neck pain 2.06
Lao People's Democratic Republic	Diabetes 1.58	Age-related hearing loss 1	Low back pain 1.16	Blindness and vision loss 0.86	Stroke 1.68	COPD 1.66	Alzheimer's disease 0.99	Other musculoskeletal 1.24	Neck pain 1.89	Osteoarthritis 0.72
Malaysia	Diabetes 1.49	Low back pain 1.07	Blindness and vision loss 2.21	Age-related hearing loss 1.13	Stroke 1.7	Oral disorders 1.06	Alzheimer's disease 0.95	Other musculoskeletal 3	COPD 1.44	Chronic kidney disease 2.02
Maldives	COPD 4.01	Age-related hearing loss 0.96	Diabetes 1.05	Low back pain 1.07	Blindness and vision loss 0.93	Stroke 0.99	Alzheimer's disease 1.03	Oral disorders 0.81	Other musculoskeletal 1.74	Chronic kidney disease 1.82
Mauritius	Diabetes 2.28	Age-related hearing loss 1.05	Blindness and vision loss 1.81	Low back pain 0.95	Stroke 1.21	Oral disorders 1.06	Alzheimer's disease 0.89	Chronic kidney disease 2.65	Other musculoskeletal 3.22	Depressive disorders 0.95
Myanmar	Blindness and vision loss 1.43	Diabetes 1.45	COPD 3.51	Age-related hearing loss 0.99	Low back pain 1.05	Alzheimer's disease 0.98	Stroke 0.86	Falls 1.98	Other musculoskeletal 1.2	Neck pain 1.84
Philippines	Low back pain 1.36	Age-related hearing loss 1	Blindness and vision loss 1.36	Diabetes 0.95	Stroke 1.31	Oral disorders 1.09	Alzheimer's disease 0.89	Neck pain 2.6	COPD 1.7	Other musculoskeletal 2.01
Seychelles	Diabetes 2.24	Blindness and vision loss 2.04	Age-related hearing loss 1.08	Low back pain 1	Stroke 1.49	Alzheimer's disease 0.88	Other musculoskeletal 3.3	Oral disorders 0.78	COPD 1.25	Chronic kidney disease 2.14
Sri Lanka	Diabetes 2.25	Age-related hearing loss 1.06	Blindness and vision loss 1.69	Low back pain 0.95	Stroke 1.25	Alzheimer's disease 0.91	COPD 1.68	Other musculoskeletal 2.75	Falls 1.2	Oral disorders 0.71
Thailand	Age-related hearing loss 1.02	Diabetes 1.02	Low back pain 1.01	Blindness and vision loss 1.43	Stroke 1.07	Alzheimer's disease 0.93	Other musculoskeletal 4.32	Oral disorders 0.81	Chronic kidney disease 1.91	COPD 1.28
Timor-Leste	Blindness and vision loss 1.69	Diabetes 1.16	Age-related hearing loss 0.99	Stroke 1.81	Low back pain 1.05	COPD 1.56	Oral disorders 0.88	Alzheimer's disease 0.98	Other musculoskeletal 1.13	Neck pain 1.86
Viet Nam	Blindness and vision loss 1.35	Diabetes 1.05	Age-related hearing loss 0.92	Low back pain 1.16	Stroke 1.33	COPD 2.27	Alzheimer's disease 0.94	Falls 1.92	Oral disorders 0.72	Neck pain 1.96
**Sub-Saharan Africa**	Blindness and vision loss 1.07	Age-related hearing loss 0.96	Low back pain 0.96	Diabetes 0.96	Depressive disorders 1.46	Stroke 0.97	Osteoarthritis 1.08	Alzheimer's disease 0.98	COPD 1.21	Oral disorders 0.66
**Central Sub-Saharan Africa**	Age-related hearing loss 0.95	Low back pain 1.03	Diabetes 1.01	Depressive disorders 1.37	Blindness and vision loss 0.61	Alzheimer's disease 1.33	COPD 1.63	Stroke 0.92	Osteoarthritis 1.05	Onchocerciasis 24.18
Angola	Age-related hearing loss 0.94	Low back pain 1.02	Blindness and vision loss 0.81	Diabetes 0.9	Depressive disorders 1.57	Stroke 1.12	Alzheimer's disease 1.33	COPD 1.43	Osteoarthritis 1.07	Oral disorders 0.58
Central African Republic	Age-related hearing loss 0.99	Low back pain 1.07	Diabetes 1.24	Blindness and vision loss 0.62	Depressive disorders 1.11	Alzheimer's disease 1.49	Stroke 0.96	Onchocerciasis 6.39	COPD 1.26	Osteoarthritis 1.03
Congo	Age-related hearing loss 0.89	Diabetes 0.89	Low back pain 1	Blindness and vision loss 0.84	Depressive disorders 1.69	Stroke 1.18	COPD 2.47	Alzheimer's disease 1.31	Osteoarthritis 1.09	Other musculoskeletal 1.14
Democratic Republic of the Congo	Age-related hearing loss 0.96	Low back pain 1.03	Diabetes 1.05	Depressive disorders 1.31	COPD 1.65	Alzheimer's disease 1.33	Blindness and vision loss 0.53	Onchocerciasis 29.61	Osteoarthritis 1.04	Stroke 0.84
Equatorial Guinea	Blindness and vision loss 1.77	Age-related hearing loss 0.96	Diabetes 0.92	Low back pain 0.94	Depressive disorders 1.74	Stroke 1.13	COPD 2.4	Alzheimer's disease 1.16	Osteoarthritis 0.97	Other musculoskeletal 2.16
Gabon	Age-related hearing loss 0.93	Diabetes 0.91	Low back pain 0.96	Blindness and vision loss 1.06	Depressive disorders 1.69	Alzheimer's disease 1.18	Stroke 1.07	Osteoarthritis 1.05	COPD 1.6	Other musculoskeletal 2.07
**Eastern Sub-Saharan Africa**	Blindness and vision loss 1.03	Age-related hearing loss 0.94	Low back pain 0.92	Depressive disorders 1.45	Diabetes 0.78	Stroke 0.99	Alzheimer's disease 1.08	Osteoarthritis 1.07	COPD 0.87	Dietary iron deficiency 1.28
Burundi	Age-related hearing loss 0.95	Low back pain 0.94	Depressive disorders 1.22	Diabetes 0.88	Blindness and vision loss 0.51	Stroke 1	Alzheimer's disease 1.12	Osteoarthritis 1.03	COPD 0.96	Dietary iron deficiency 1.31
Comoros	Age-related hearing loss 0.91	Blindness and vision loss 0.92	Low back pain 0.93	Depressive disorders 1.46	Stroke 1.23	Diabetes 0.66	Alzheimer's disease 1.06	Osteoarthritis 1.07	Road injuries 2.62	Dietary iron deficiency 1.48
Djibouti	Age-related hearing loss 0.89	Blindness and vision loss 0.89	Low back pain 0.85	Depressive disorders 1.55	Diabetes 0.78	Stroke 1.24	Osteoarthritis 1.09	Alzheimer's disease 1.08	Dietary iron deficiency 1.33	Road injuries 2.14
Eritrea	Blindness and vision loss 1.02	Age-related hearing loss 0.91	Depressive disorders 1.46	Low back pain 0.81	Diabetes 0.83	Stroke 0.96	Osteoarthritis 1.03	Alzheimer's disease 1.08	Dietary iron deficiency 1.52	COPD 0.97
Ethiopia	Blindness and vision loss 1.24	Age-related hearing loss 0.98	Low back pain 0.96	Depressive disorders 1.21	Diabetes 0.75	Alzheimer's disease 1.1	Osteoarthritis 1.08	Stroke 0.72	Trachoma 12.42	Falls 1.36
Kenya	Age-related hearing loss 1.18	Blindness and vision loss 1.14	Low back pain 0.92	Depressive disorders 1.78	Stroke 0.98	Diabetes 0.53	Alzheimer's disease 1.03	Osteoarthritis 1.09	COPD 1.21	Dietary iron deficiency 1.24
Madagascar	Age-related hearing loss 1	Blindness and vision loss 0.76	Low back pain 0.91	Stroke 1.43	Depressive disorders 1.45	Diabetes 0.74	COPD 1.42	Osteoarthritis 1.04	Alzheimer's disease 1.05	Dietary iron deficiency 1.58
Malawi	Blindness and vision loss 0.94	Age-related hearing loss 0.93	Low back pain 0.91	Diabetes 0.93	Depressive disorders 1.18	Stroke 0.98	Alzheimer's disease 1.08	Osteoarthritis 1.07	Dietary iron deficiency 1.41	COPD 0.76
Mozambique	Blindness and vision loss 0.98	Age-related hearing loss 0.94	Low back pain 0.96	Depressive disorders 1.28	Diabetes 0.93	Stroke 1.25	Alzheimer's disease 1.09	Osteoarthritis 1.05	Road injuries 2.2	Dietary iron deficiency 1.35
Rwanda	Age-related hearing loss 0.91	Depressive disorders 1.76	Low back pain 0.94	Blindness and vision loss 0.61	Diabetes 0.73	Alzheimer's disease 1.09	Stroke 0.89	Osteoarthritis 1.06	COPD 1.14	Conflict & terror 225.07
Somalia	Age-related hearing loss 1	Blindness and vision loss 0.73	Low back pain 0.99	Depressive disorders 0.87	Diabetes 1.23	Stroke 0.96	Dietary iron deficiency 1.47	Osteoarthritis 1.03	Alzheimer's disease 1.13	COPD 0.69
South Sudan	Onchocerciasis 86.19	Blindness and vision loss 1.26	Age-related hearing loss 0.89	Low back pain 0.9	Depressive disorders 1.33	Stroke 1.22	Diabetes 0.81	Alzheimer's disease 1.05	Osteoarthritis 1.02	Dietary iron deficiency 1.52
Uganda	Depressive disorders 2.22	Low back pain 0.92	Diabetes 1	Blindness and vision loss 0.73	Age-related hearing loss 0.65	Stroke 1.08	Alzheimer's disease 1.08	Osteoarthritis 1.06	COPD 0.9	Dietary iron deficiency 1.21
United Republic of Tanzania	Blindness and vision loss 1.2	Age-related hearing loss 0.87	Low back pain 0.88	Depressive disorders 1.53	Diabetes 0.74	Stroke 1.04	Alzheimer's disease 1.07	Osteoarthritis 1.08	Dietary iron deficiency 1.4	COPD 0.76
Zambia	Age-related hearing loss 0.88	Blindness and vision loss 0.8	Low back pain 0.85	Diabetes 0.74	Depressive disorders 1.44	Stroke 1.04	Osteoarthritis 1.08	Alzheimer's disease 1.05	Dietary iron deficiency 1.76	COPD 1.04
**Southern Sub-Saharan Africa**	Diabetes 1.28	Age-related hearing loss 1.09	Blindness and vision loss 1.29	Low back pain 0.86	Depressive disorders 1.57	Stroke 1.09	COPD 2.14	Alzheimer's disease 0.95	Osteoarthritis 1	Oral disorders 0.74
Botswana	Diabetes 1.19	Age-related hearing loss 0.97	Blindness and vision loss 1.24	Low back pain 0.89	Stroke 1.16	COPD 2.61	Depressive disorders 1.45	Alzheimer's disease 0.97	Osteoarthritis 1.06	Endo/metab/blood/immun 2.26
Eswatini	Diabetes 1.26	Age-related hearing loss 0.96	Blindness and vision loss 1.11	Low back pain 0.85	Stroke 1.05	Depressive disorders 1.44	COPD 2.12	Osteoarthritis 1.14	HIV/AIDS 951.16	Alzheimer's disease 0.99
Lesotho	Diabetes 1.18	Age-related hearing loss 1	Blindness and vision loss 1.06	Depressive disorders 2.05	Low back pain 0.87	COPD 2.15	HIV/AIDS 417.22	Stroke 0.99	Osteoarthritis 1.09	Alzheimer's disease 1.02
Namibia	Age-related hearing loss 0.98	Diabetes 0.94	Blindness and vision loss 1.16	Low back pain 0.94	Stroke 0.99	Alzheimer's disease 0.99	COPD 2.04	Depressive disorders 1.16	Osteoarthritis 1.04	Oral disorders 0.77
South Africa	Diabetes 1.28	Age-related hearing loss 1.12	Blindness and vision loss 1.36	Low back pain 0.84	Depressive disorders 1.63	Stroke 1.1	COPD 2.26	Alzheimer's disease 0.94	Osteoarthritis 0.99	Oral disorders 0.74
Zimbabwe	Diabetes 1.5	Blindness and vision loss 1.04	Age-related hearing loss 0.96	Low back pain 1.02	Stroke 1.1	Depressive disorders 1.18	COPD 1.41	Osteoarthritis 1.07	Alzheimer's disease 1.04	Oral disorders 0.76
**Western Sub-Saharan Africa**	Blindness and vision loss 1.19	Age-related hearing loss 0.92	Low back pain 1.02	Diabetes 0.95	Depressive disorders 1.47	Stroke 0.91	Osteoarthritis 1.13	COPD 1.11	Alzheimer's disease 0.82	Oral disorders 0.67
Benin	Blindness and vision loss 0.98	Age-related hearing loss 0.93	Diabetes 1.31	Low back pain 0.97	Depressive disorders 1.39	Osteoarthritis 1.14	COPD 1.27	Stroke 0.88	Alzheimer's disease 0.85	Oral disorders 0.87
Burkina Faso	Blindness and vision loss 1.01	Age-related hearing loss 0.96	Diabetes 1.34	Low back pain 1.01	Depressive disorders 1.08	Osteoarthritis 1.1	Alzheimer's disease 0.95	Road injuries 1.62	Stroke 0.64	COPD 0.81
Cabo Verde	Diabetes 1.39	Age-related hearing loss 0.91	Blindness and vision loss 0.83	Depressive disorders 1.98	Low back pain 0.89	Alzheimer's disease 0.91	Osteoarthritis 1.14	COPD 1.23	Stroke 0.79	Oral disorders 0.63
Cameroon	Age-related hearing loss 0.89	Blindness and vision loss 0.92	Diabetes 1.03	Low back pain 0.98	Depressive disorders 1.85	COPD 1.63	Osteoarthritis 1.16	Stroke 0.83	Alzheimer's disease 0.85	Road injuries 2.47
Chad	Blindness and vision loss 1	Age-related hearing loss 0.96	Diabetes 1.43	Depressive disorders 1.38	Low back pain 1.06	Stroke 0.9	Osteoarthritis 1.06	Alzheimer's disease 0.91	COPD 1.01	Oral disorders 1.22
Côte d'Ivoire	Blindness and vision loss 1.05	Diabetes 1.26	Age-related hearing loss 0.92	Low back pain 0.96	Depressive disorders 1.3	Stroke 0.96	Osteoarthritis 1.12	Alzheimer's disease 0.88	Oral disorders 0.92	COPD 1.03
Gambia	Depressive disorders 2.53	Blindness and vision loss 1.01	Age-related hearing loss 0.92	Diabetes 1.1	Low back pain 0.91	Stroke 1.01	COPD 1.35	Osteoarthritis 1.11	Alzheimer's disease 0.88	Oral disorders 0.84
Ghana	Diabetes 1	Blindness and vision loss 1.05	Age-related hearing loss 0.87	Depressive disorders 1.73	Stroke 1.21	Low back pain 0.77	Osteoarthritis 1.13	Alzheimer's disease 0.82	COPD 1.06	Road injuries 2.49
Guinea	Blindness and vision loss 1.08	Age-related hearing loss 0.94	Diabetes 1.19	Low back pain 0.97	Depressive disorders 1.34	Osteoarthritis 1.09	COPD 1.21	Alzheimer's disease 0.9	Stroke 0.82	Oral disorders 0.92
Guinea-Bissau	Blindness and vision loss 1.17	Age-related hearing loss 0.93	Diabetes 1.23	Low back pain 0.94	Depressive disorders 1.41	Stroke 0.97	Osteoarthritis 1.08	COPD 1.19	Alzheimer's disease 0.88	Road injuries 1.87
Liberia	Blindness and vision loss 0.98	Diabetes 1.44	Age-related hearing loss 0.92	Depressive disorders 1.8	Onchocerciasis 47.25	Low back pain 0.92	Osteoarthritis 1.12	Stroke 0.8	Alzheimer's disease 0.87	COPD 1.06
Mali	Blindness and vision loss 1.35	Age-related hearing loss 0.96	Diabetes 1.39	Low back pain 0.89	Depressive disorders 0.9	COPD 1.45	Osteoarthritis 1.08	Stroke 0.85	Alzheimer's disease 0.93	Oral disorders 0.92
Mauritania	Blindness and vision loss 1.03	Age-related hearing loss 0.9	Low back pain 0.89	Diabetes 0.87	Depressive disorders 1.26	Stroke 0.99	Osteoarthritis 1.17	COPD 1.33	Alzheimer's disease 0.87	Oral disorders 0.76
Niger	Blindness and vision loss 0.94	Age-related hearing loss 0.99	Low back pain 1.01	Depressive disorders 0.99	Diabetes 1.13	Osteoarthritis 1.07	COPD 1.01	Stroke 0.81	Alzheimer's disease 0.96	Oral disorders 1.25
Nigeria	Blindness and vision loss 1.4	Low back pain 1.13	Age-related hearing loss 0.92	Depressive disorders 1.66	Diabetes 0.7	Osteoarthritis 1.15	Stroke 0.89	Alzheimer's disease 0.75	COPD 1.03	Gynecological diseases 1.86
Sao Tome and Principe	Blindness and vision loss 1.01	Diabetes 1.11	Age-related hearing loss 0.9	COPD 2.66	Stroke 1.39	Low back pain 0.83	Depressive disorders 1.4	Osteoarthritis 1.13	Alzheimer's disease 0.86	Oral disorders 0.65
Senegal	Blindness and vision loss 1.17	Diabetes 1.58	Age-related hearing loss 0.92	Low back pain 0.87	Depressive disorders 1.25	Stroke 0.93	Osteoarthritis 1.11	Alzheimer's disease 0.89	Oral disorders 0.97	COPD 1.02
Sierra Leone	Age-related hearing loss 1	Blindness and vision loss 0.92	Low back pain 0.95	Depressive disorders 1.42	Diabetes 0.91	Stroke 1.06	COPD 1.27	Osteoarthritis 1.09	Alzheimer's disease 0.89	Road injuries 1.81
Togo	Blindness and vision loss 1.05	Age-related hearing loss 0.92	Low back pain 0.94	Depressive disorders 1.58	Diabetes 0.81	Stroke 0.92	Osteoarthritis 1.1	COPD 1.22	Alzheimer's disease 0.88	Oral disorders 0.79

Values shown in brackets represent the ratio of observed YLDs to predicted YLDs on the basis of Socio-Demographic Index (SDI), rounded to two (2) digits. Color ranges (shown below) were calculated to place a roughly equal number of cells into each bin.

COLOR KEY:	[0.00,0.82)	[0.82,0.91)	[0.91,0.97)	[0.97,1.02)	[1.02,1.08)	[1.08,1.17)	[1.17,1.27)	[1.27,1.45)	[1.45,1.87)	[1.87,...)

Figure S24. Global annualised rate of change in risk factors summary exposure values (SEV) in people aged 70 years and older, for both sexes, from 1990 to 2019 versus number of attributable DALYs by risk factor in 2019.

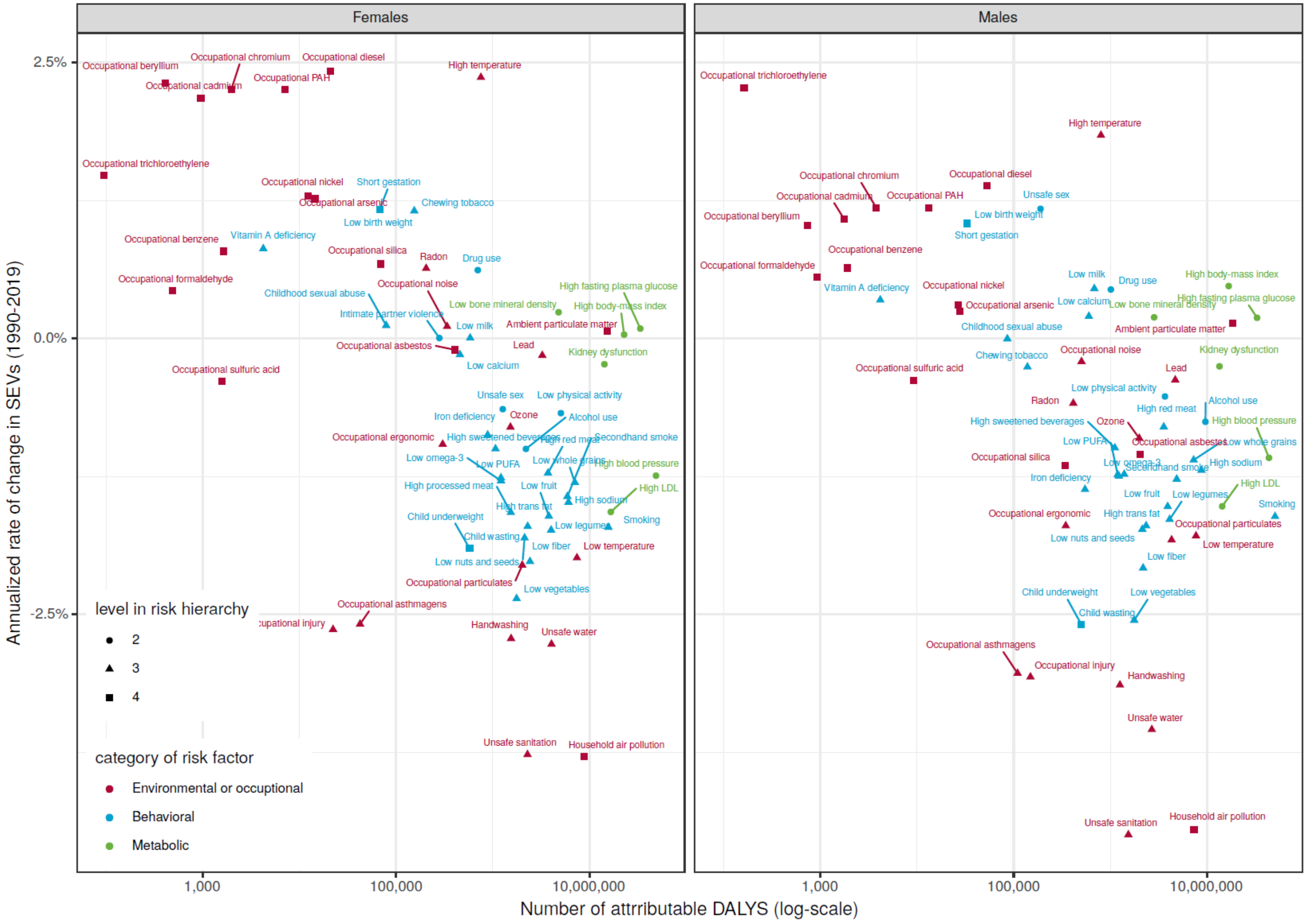

Figure S25. Annualised rate of change in risk factors summary exposure values (SEV) in people aged 70 years and older, for both sexes, from 1990 to 2019 versus number of attributable DALYs by risk factor in 2019, in Low SDI quintile.

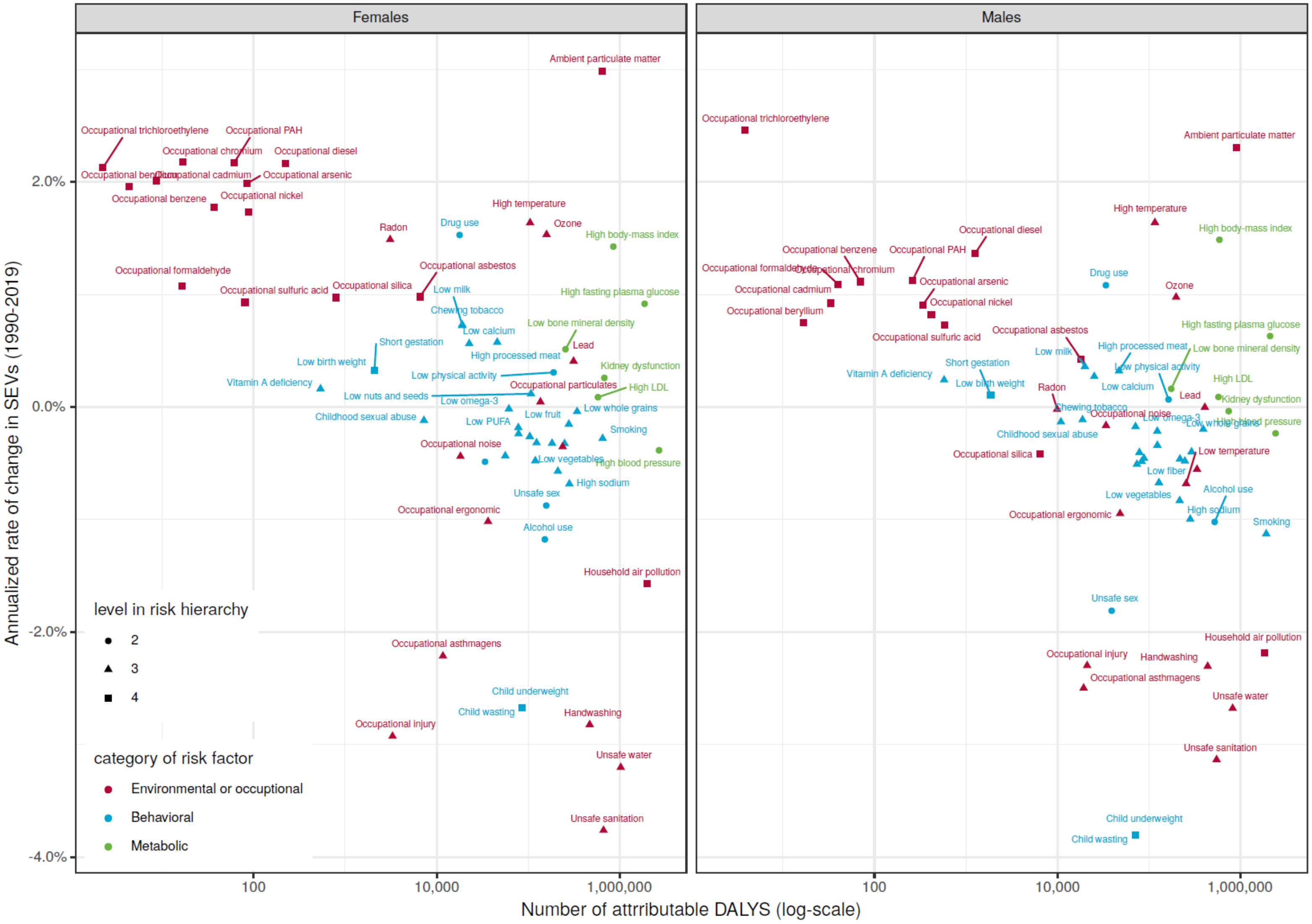

Figure S26. Annualised rate of change in risk factors summary exposure values (SEV) in people aged 70 years and older, for both sexes, from 1990 to 2019 versus number of attributable DALYs by risk factor in 2019, in Low-middle SDI quintile.

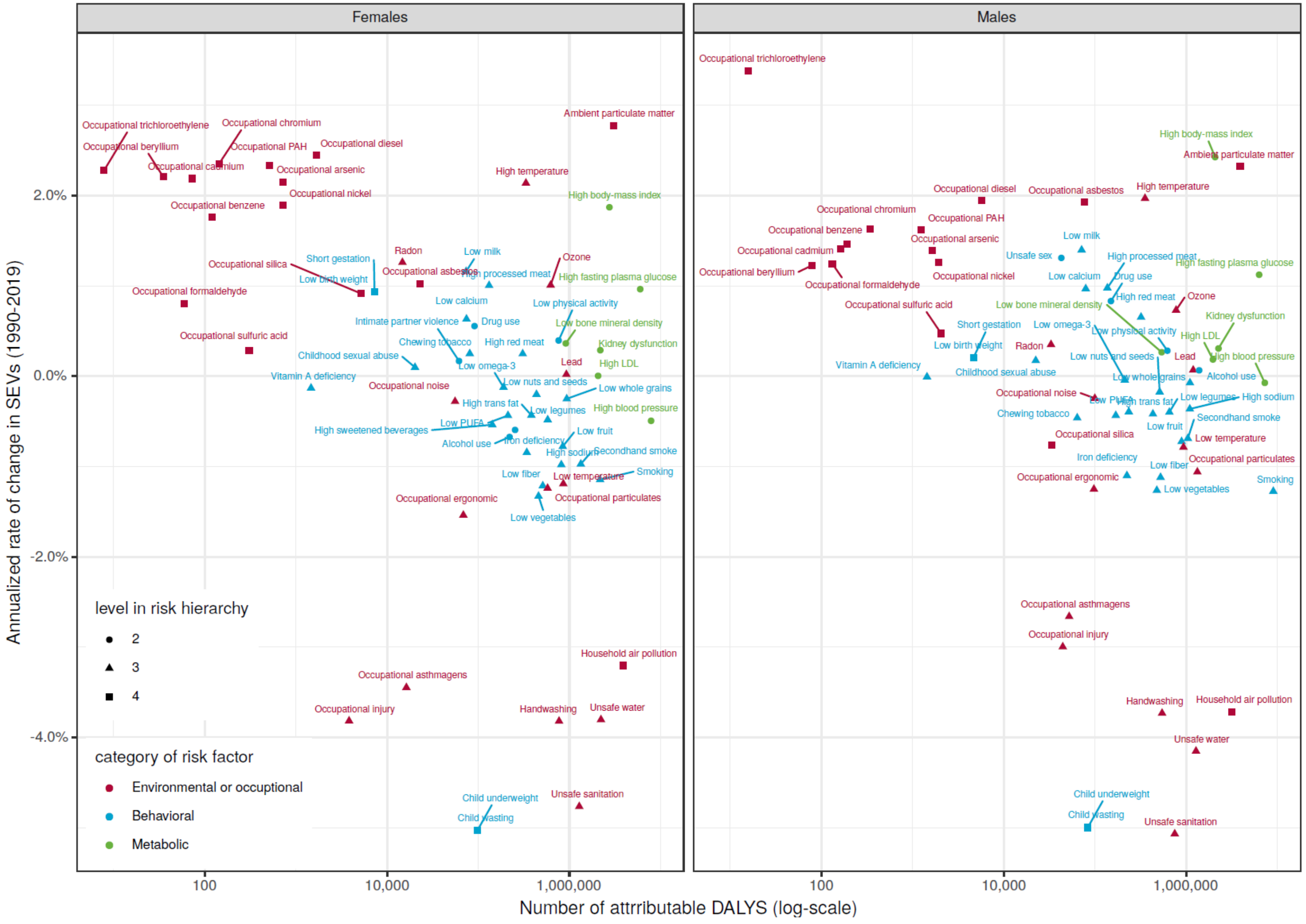

Figure S27. Annualised rate of change in risk factors summary exposure values (SEV) in people aged 70 years and older, for both sexes, from 1990 to 2019 versus number of attributable DALYs by risk factor in 2019, in Middle SDI quintile.

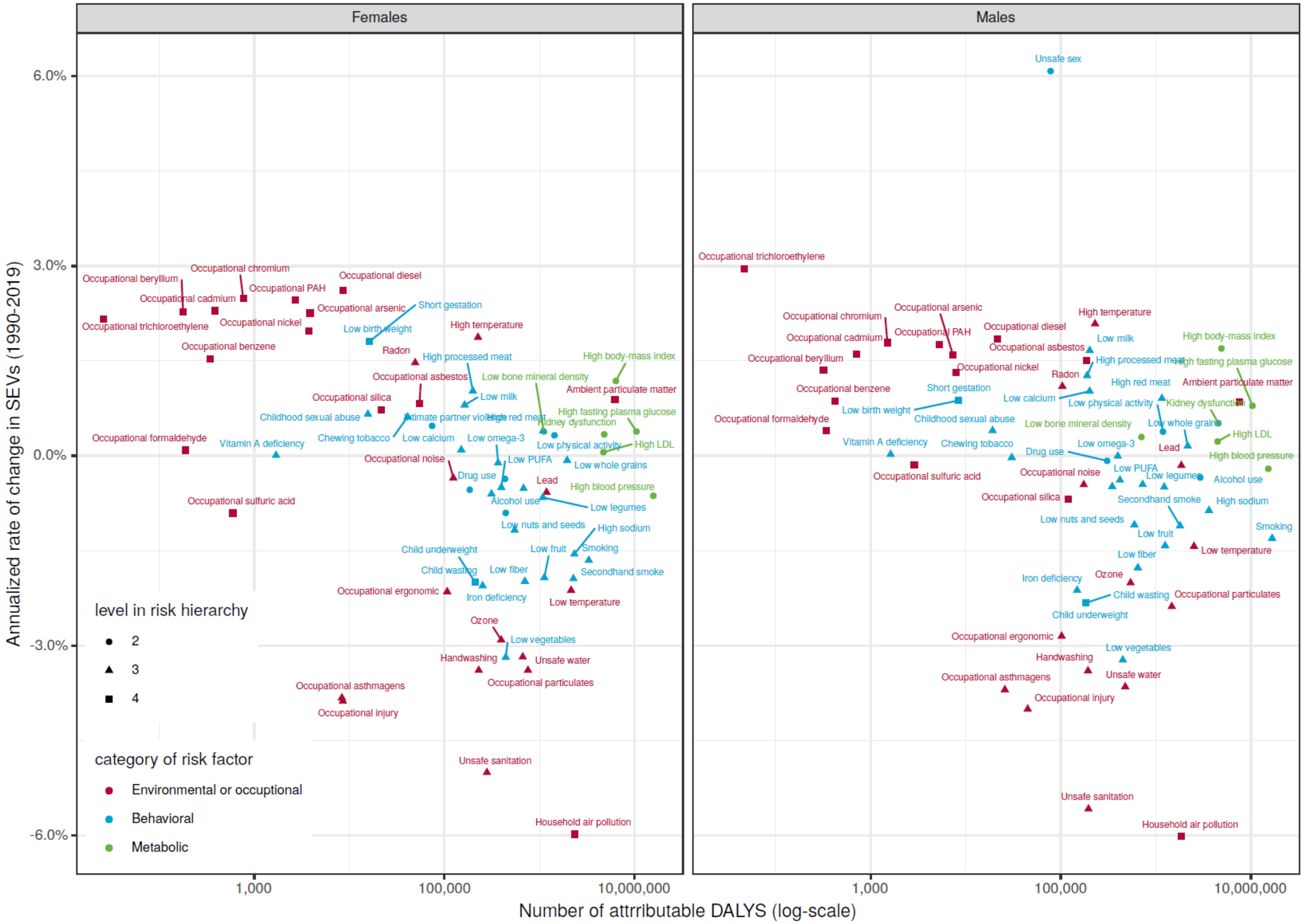

Figure S28. Annualised rate of change in risk factors summary exposure values (SEV) in people aged 70 years and older, for both sexes, from 1990 to 2019 versus number of attributable DALYs by risk factor in 2019, in High-middle SDI quintile.

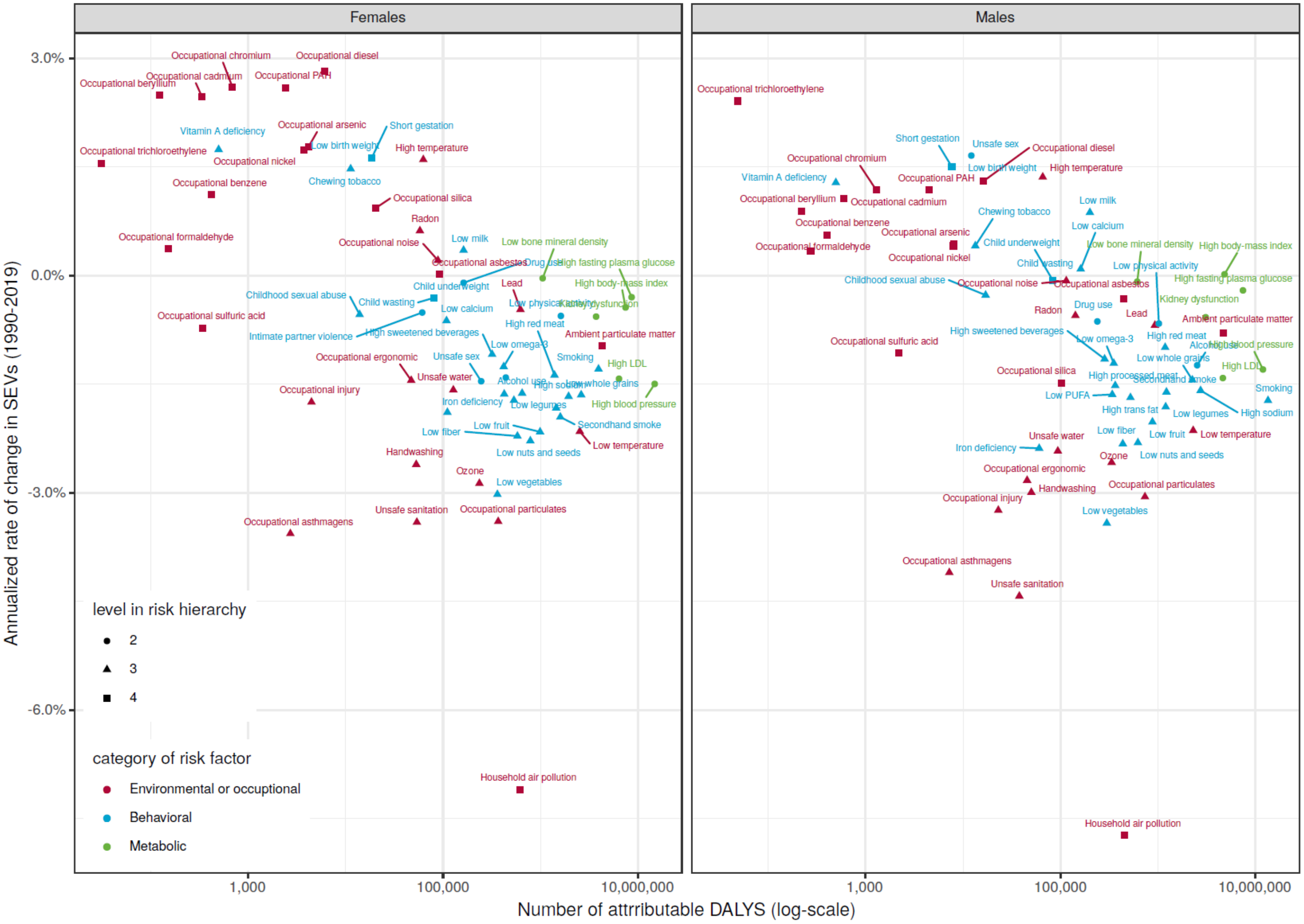

Figure S29. Annualised rate of change in risk factors summary exposure values (SEV) in people aged 70 years and older, for both sexes, from 1990 to 2019 versus number of attributable DALYs by risk factor in 2019, in High SDI quintile.

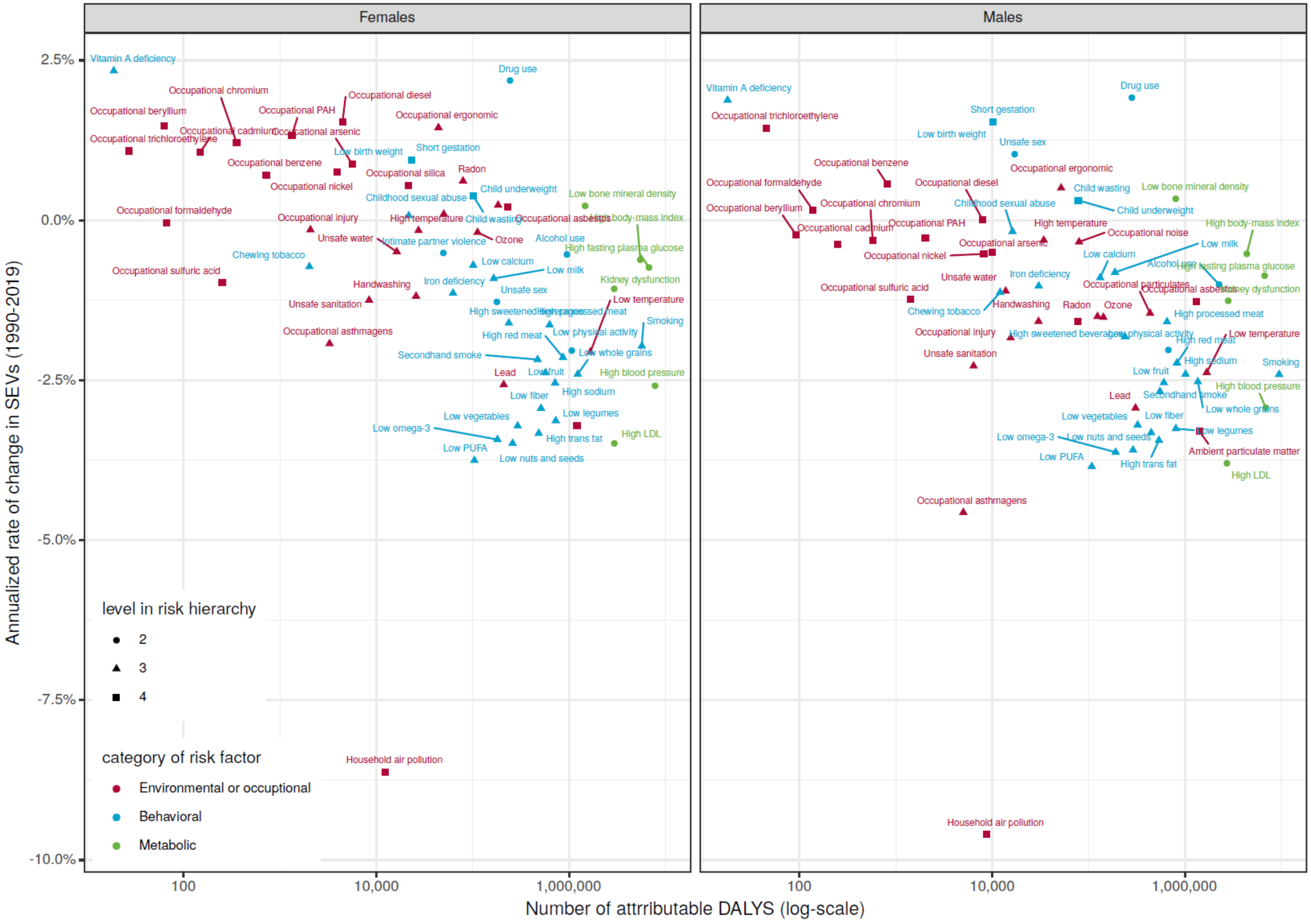

Figure S30. Annualised rate of change in risk factors summary exposure values (SEV) in people aged 70 years and older, for both sexes, from 1990 to 2019 versus number of attributable DALYs by risk factor in 2019, in the Central Europe, Eastern Europe and Central Asia GBD super-region.

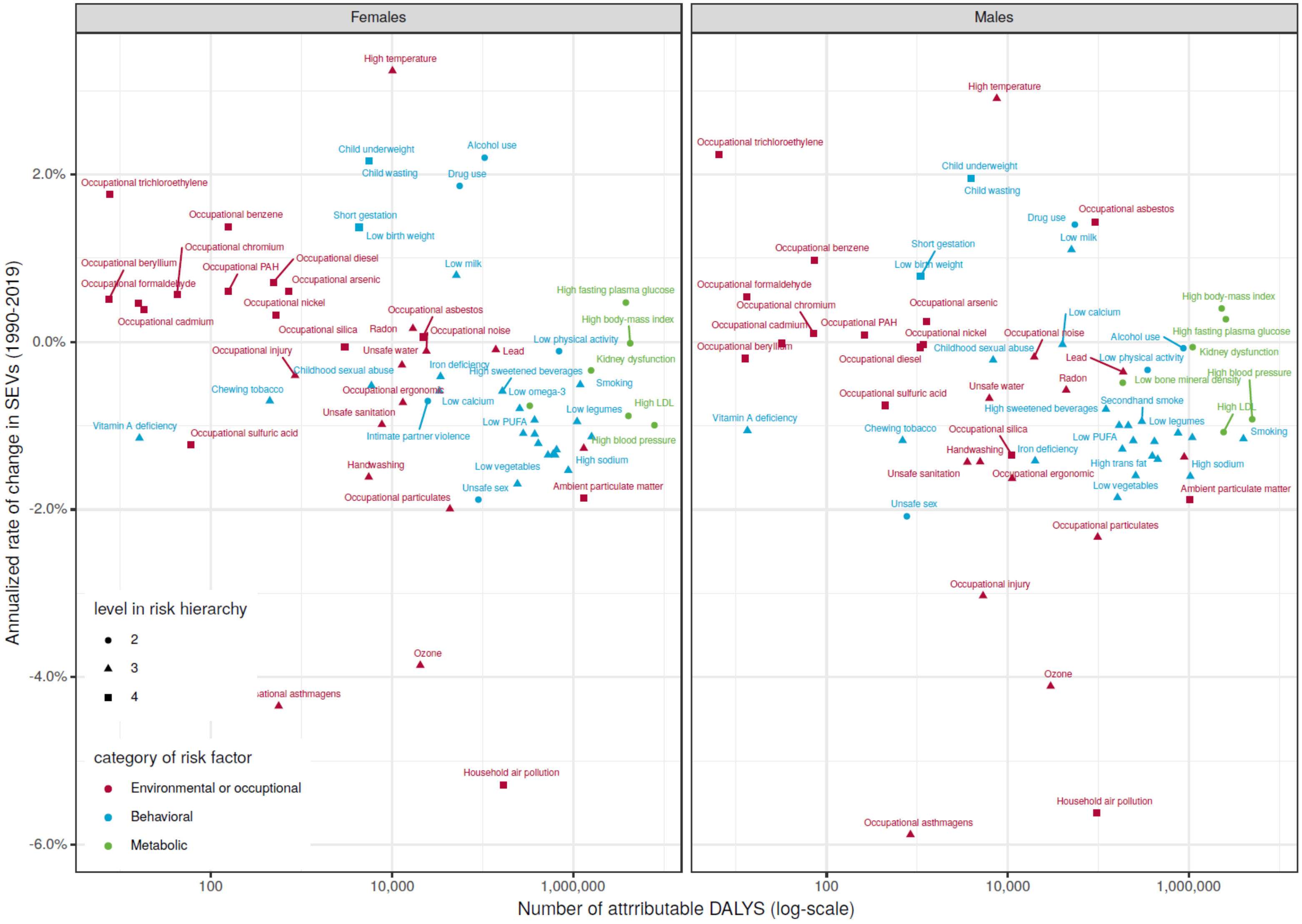

Figure S31. Annualised rate of change in risk factors summary exposure values (SEV) in people aged 70 years and older, for both sexes, from 1990 to 2019 versus number of attributable DALYs by risk factor in 2019, in the High-income GBD super-region.

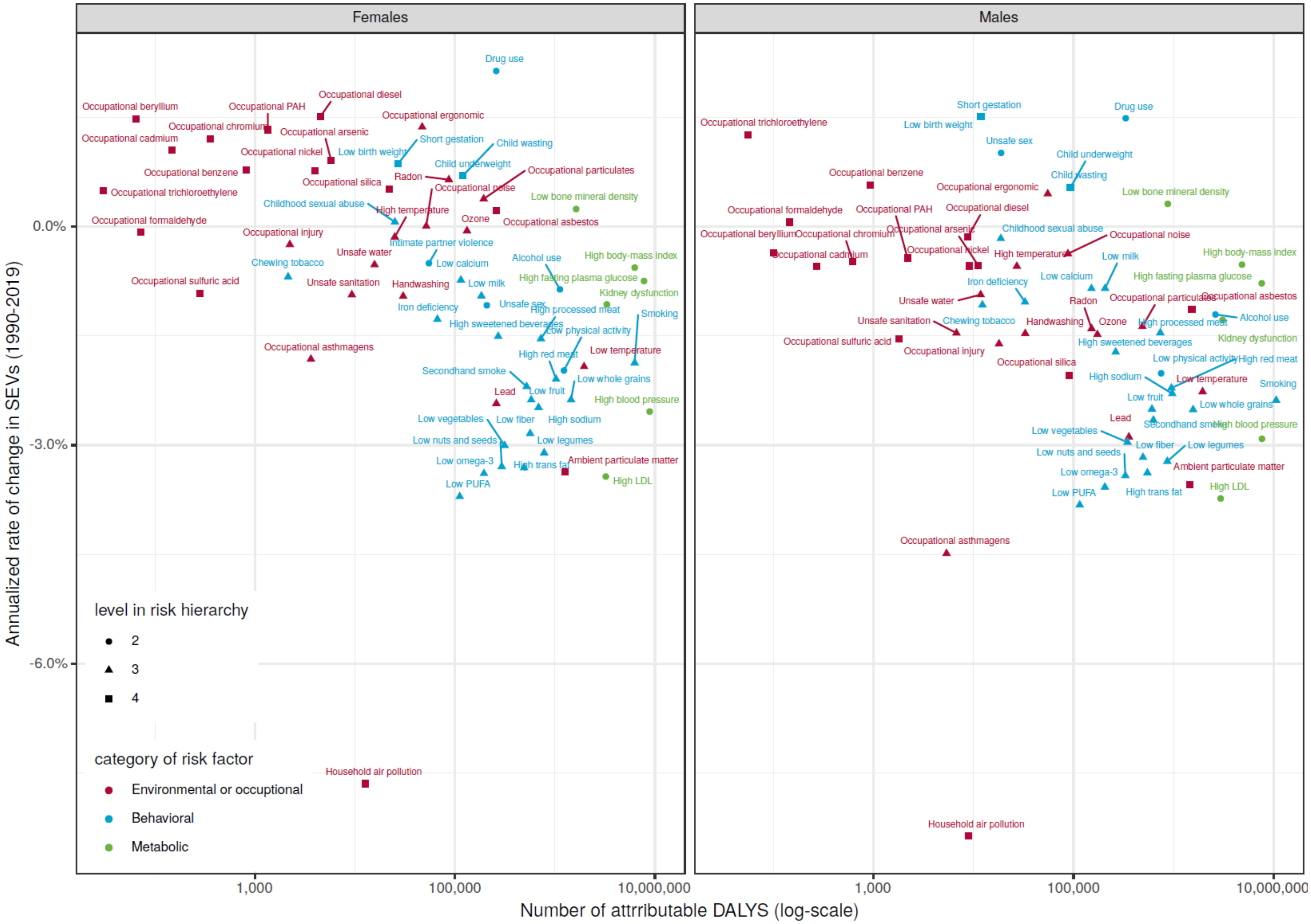

Figure S32. Annualised rate of change in risk factors summary exposure values (SEV) in people aged 70 years and older, for both sexes, from 1990 to 2019 versus number of attributable DALYs by risk factor in 2019, in the Latin America and Caribbean GBD super-region

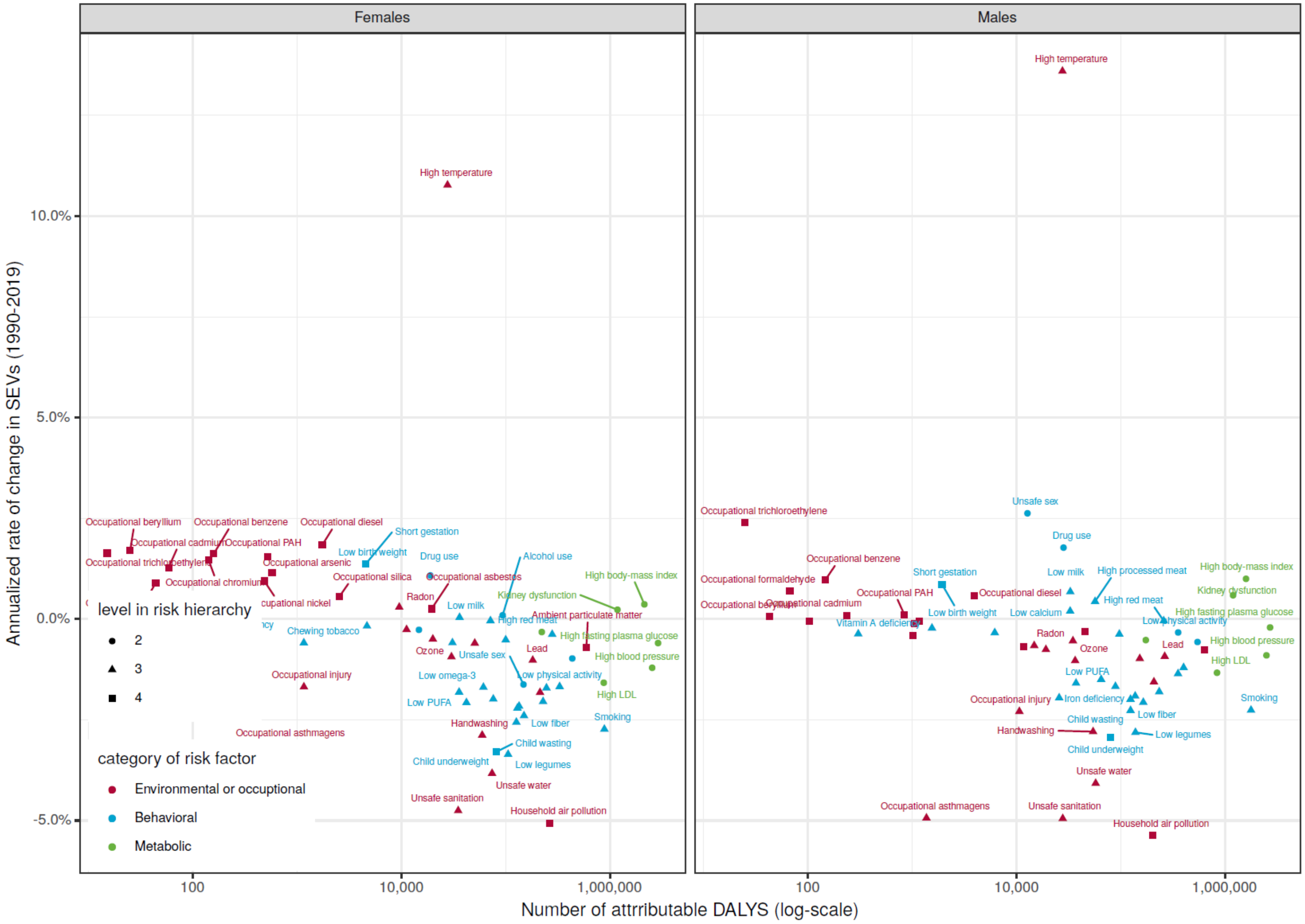

Figure S33. Annualised rate of change in risk factors summary exposure values (SEV) in people aged 70 years and older, for both sexes, from 1990 to 2019 versus number of attributable DALYs by risk factor in 2019, in the North Africa and Middle East super-region.

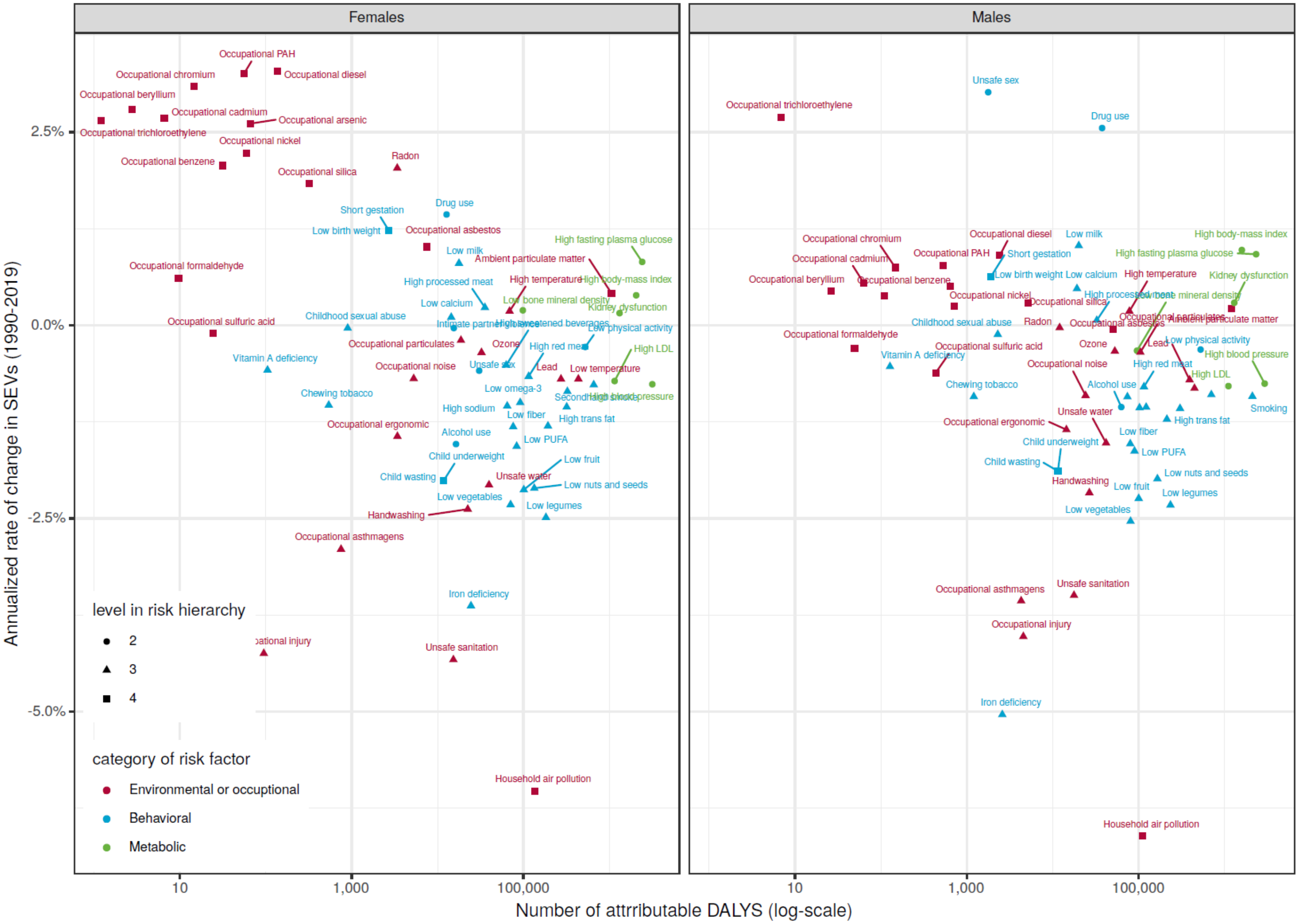

Figure S34. Annualised rate of change in risk factors summary exposure values (SEV) in people aged 70 years and older, for both sexes, from 1990 to 2019 versus number of attributable DALYs by risk factor in 2019, in the South Asia GBD super-region

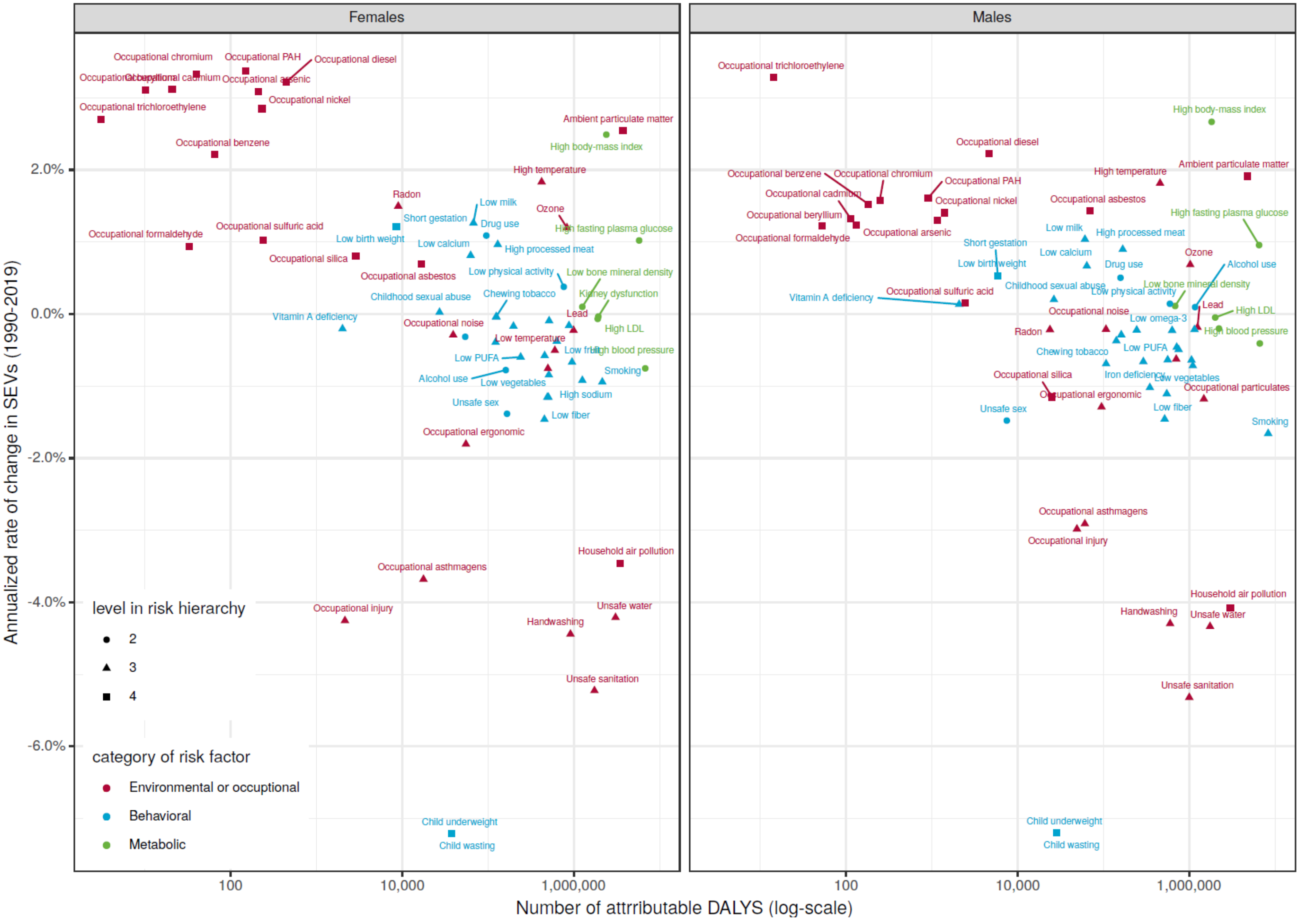

Figure S35. Annualised rate of change in risk factors summary exposure values (SEV) in people aged 70 years and older, for both sexes, from 1990 to 2019 versus number of attributable DALYs by risk factor in 2019, in the Southeast Asia, East Asia and Oceania GBD super-region.

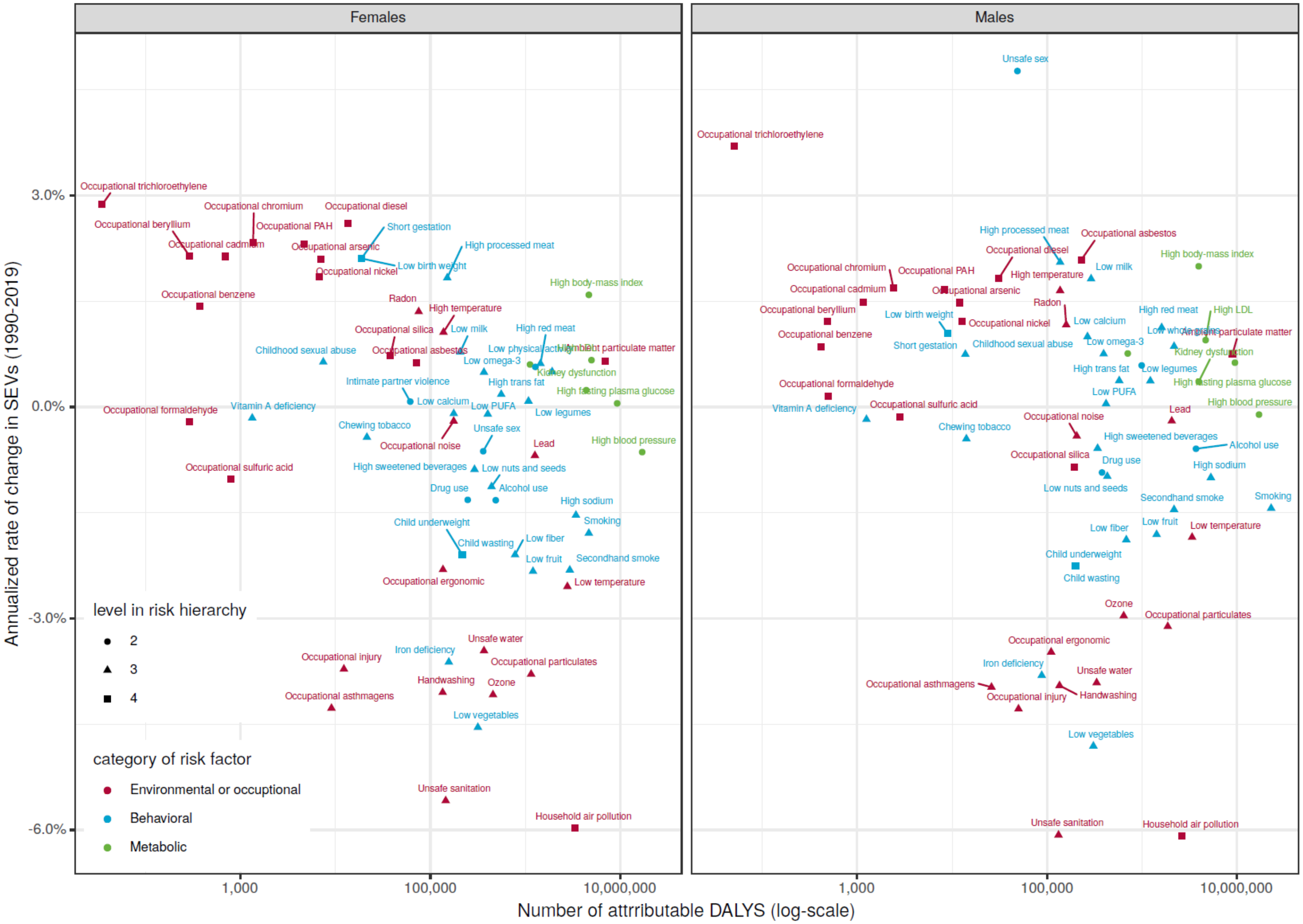

Figure S36. Annualised rate of change in risk factors summary exposure values (SEV) in people aged 70 years and older, for both sexes, from 1990 to 2019 versus number of attributable DALYs by risk factor in 2019, in the Sub-Saharan Africa GBD super-region.

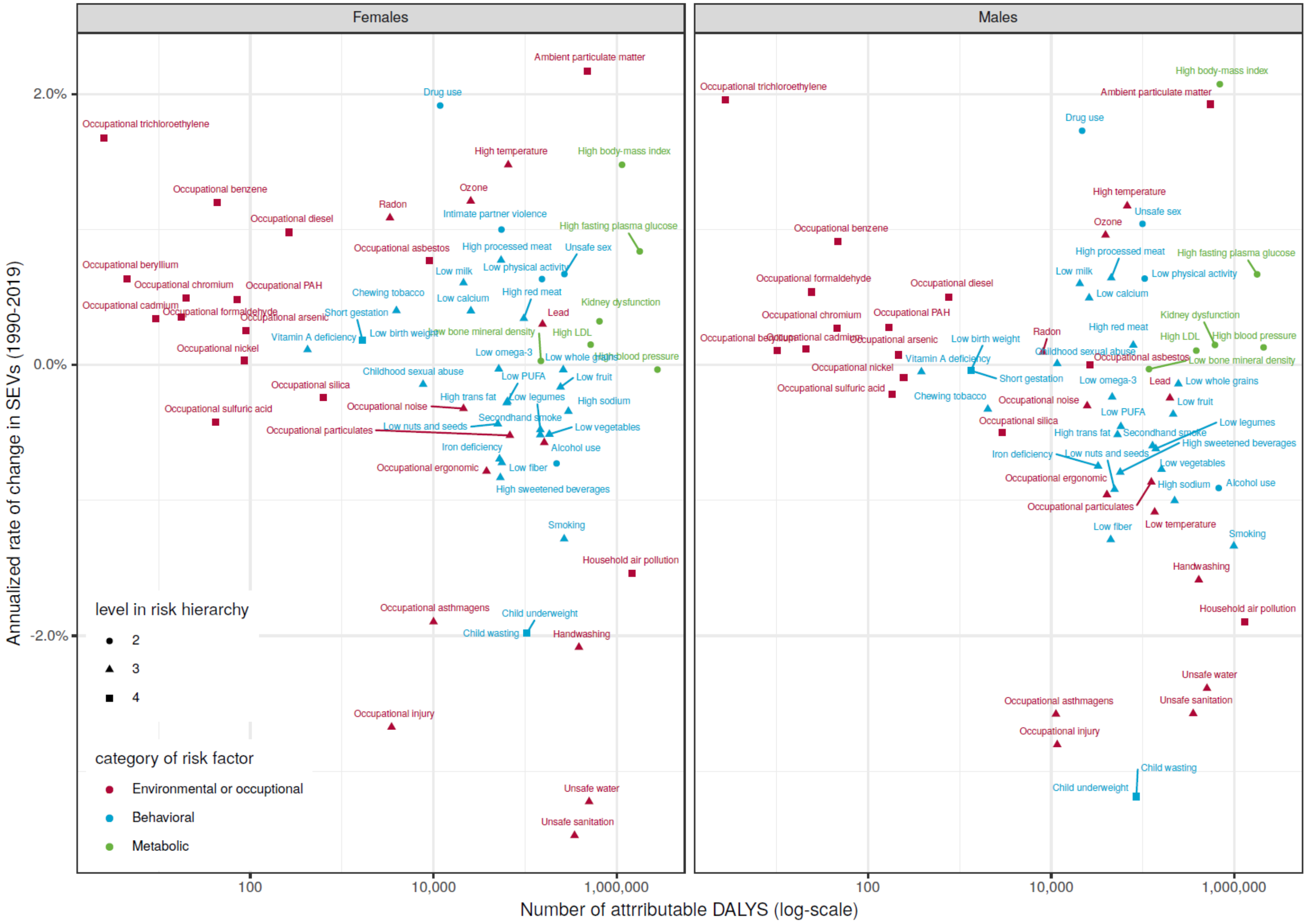

Supplementary Table 1. GATHER checklist of information that should be included in reports of global health estimates, with description of compliance and location of information for "Global, regional, and national burden of diseases and injuries for adults 70 years and older: a systematicanalysis for the Global Burden of Disease 2019 Study"			
**#**	**GATHER checklist item**	**Description ofcompliance**	**Reference**
**Objectives and funding**			
1	Define the indicators, populations, and time periods forwhich estimates were made.	Narrative provided inpaper and appendix describing indicators, definitions, and populations	Main text methods and appendix tables
2	List the funding sources for the work.	Funding sources listedin paper	Abstract and declaration of interest (Funding)
**Data Inputs**			
For all data inputs from multiple sources that are synthesized as part of the study:			
3	Describe how the data were identified and how the data were accessed.	Narrative description of data seeking methods provided	Main text (Methods) and appendix
4	Specify the inclusion and exclusion criteria. Identify all ad-hocexclusions.	Narrative about inclusion and exclusion criteria by data type provided	Main text (Methods) and appendix
5	Provide information on all included data sources and their main characteristics. For each data source used, report reference information or contact name/institution, population represented, data collection method, year(s) of data collection, sex and age range, diagnostic criteria or measurement method, and sample size, as relevant.	An interactive, online data source tool that provides metadata for data sources by component, geography, cause, risk, or impairment has been developed. Detailed descriptions of all cause-specific analyses	Main text (methods), appendix, accompanying publications, and online tools including GBD Input Data Sources Tool at https://ghdx.healthdata.org
6	Identify and describe any categories of input data that have potentially important biases (e.g., based on characteristics listed in item 5).	References on related biases are reported in the main text description	Main text (Methods) and limitations
For data inputs that contribute to the analysis but were not synthesized as part of the study:			
7	Describe and give sources for any other data inputs.	All are included in online data sources tool	https://ghdx.healthdata.org
For all data inputs:			
8	Provide all data inputs in a file format from which data can beefficiently extracted (e.g., a spreadsheet as opposed to a PDF), including all relevant meta-data listed in item 5. For any data inputs that cannot be shared due to ethical or legal reasons, such as third-party ownership, provide a contact name or the name of the institution that retains the right to the data.	Visualization of input data available through online model visualization tools at and for download via data query tools at	https://vizhub.healthdata.org and https://ghdx.healthdata.org
**Data analysis**			
9	Provide a conceptual overview of the data analysis method. A diagram may be helpful.	Description of the overall methodologicalprocesses, as well as of modelling processes, is provided in methods. Main text states that data analysis is followingthe GBD 2019 capstone papers	Main text (Methods)

10	Provide a detailed description of all steps of the analysis,including mathematical formulae, if necessary. This description should cover, as relevant, data cleaning, data pre-processing, data adjustments and weighting of data sources, and mathematical or statistical model(s).	Detailed methodological description of the analysed variables and statistical analysisis provided. Information on this models is following the GBD 2019 capstone papers andrelevant references have been provided.	Main text (Methods)
11	Describe how candidate models were evaluated and how the final model(s) were selected.	Information on this models is following theGBD 2019 capstone papers and relevant references have been provided	Main text (methods)
12	Provide the results of an evaluation of model performance, ifdone, as well as the results of any relevant sensitivity analysis.	Information on this models is following theGBD 2019 capstone papers and relevant references have been provided	Main text (methods)
13	Describe methods for calculating uncertainty of the estimates. State which sources of uncertainty were, and werenot, accounted for in the uncertainty analysis.	Brief Information on theuncertainty intervals is previded and is following the GBD 2019 capstone papers. Relevant referenceshave been provided	Main text (methods)
14	State how analytic or statistical source code used to generate estimates can be accessed.	Access statement provided.	Code is available upon request.
**Results and Discussion**			
15	Provide published estimates in a file format from which data can be efficiently extracted.	Results are availablethrough online data visualization tools, the Global Health Data Exchange, and the online data query tool (https://ghdx.healthdata.org/gbd-2019)	Main text, appendix, and online data tools (data visualization tools, dataquery tools, and the Global Health Data Exchange
16	Report a quantitative measure of the uncertainty of theestimates (e.g. uncertainty intervals).	Uncertainty intervals are provided with all results	Main text, appendix,and online data tools (data visualization tools, dataquery tools, and the Global Health Data Exchange, https://ghdx.healthdata.org/gbd-2019)
17	Interpret results in light of existing evidence. If updating a previous set of estimates, describe the reasons for changes in estimates.	Discussion of results is provided in the narrative of the article	Main text (results and Discussion)
18	Discuss limitations of the estimates. Include a discussion of any modelling assumptions or data limitations that affect interpretation of the estimates.	Discussion of limitations provided in the narrative of of the article	Main text (Limitations)

Supplementary Table 2. GBD location hierarchy, with SDI quintile

Geography	Level in hierarchy	SDI quintile
Global	Global	-
Central Europe, Eastern Europe, and Central Asia	Super region	-
Central Asia	Region	-
Armenia	Country	Middle SDI
Azerbaijan	Country	Middle SDI
Georgia	Country	High-middle SDI
Kazakhstan	Country	High-middle SDI
Kyrgyzstan	Country	Low-middle SDI
Mongolia	Country	Low-middle SDI
Tajikistan	Country	Low-middle SDI
Turkmenistan	Country	Middle SDI
Uzbekistan	Country	Middle SDI
Central Europe	Region	-
Albania	Country	Middle SDI
Bosnia and Herzegovina	Country	High-middle SDI
Bulgaria	Country	High-middle SDI
Croatia	Country	High-middle SDI
Czechia	Country	High SDI
Hungary	Country	High-middle SDI
Montenegro	Country	High-middle SDI
North Macedonia	Country	High-middle SDI
Poland	Country	High-middle SDI
Romania	Country	High-middle SDI
Serbia	Country	High-middle SDI
Slovakia	Country	High SDI
Slovenia	Country	High SDI
Eastern Europe	Region	-
Belarus	Country	High-middle SDI
Estonia	Country	High SDI
Latvia	Country	High SDI
Lithuania	Country	High SDI
Republic of Moldova	Country	High-middle SDI
Russian Federation	Country	High-middle SDI
Ukraine	Country	High-middle SDI
High-income	Super region	-
Australasia	Region	-
Australia	Country	High SDI
New Zealand	Country	High SDI
High-income Asia Pacific	Region	-
Brunei Darussalam	Country	High SDI
Japan	Country	High SDI
Aichi	Subnational Level 1	High SDI
Akita	Subnational Level 1	High SDI
Aomori	Subnational Level 1	High SDI
Chiba	Subnational Level 1	High SDI
Ehime	Subnational Level 1	High SDI
Fukui	Subnational Level 1	High SDI
Fukuoka	Subnational Level 1	High SDI
Fukushima	Subnational Level 1	High SDI
Gifu	Subnational Level 1	High SDI
Gunma	Subnational Level 1	High SDI
Hiroshima	Subnational Level 1	High SDI
Hokkaidō	Subnational Level 1	High SDI
Hyōgo	Subnational Level 1	High SDI
Ibaraki	Subnational Level 1	High SDI
Ishikawa	Subnational Level 1	High SDI
Iwate	Subnational Level 1	High SDI
Kagawa	Subnational Level 1	High SDI
Kagoshima	Subnational Level 1	High SDI
Kanagawa	Subnational Level 1	High SDI
Kōchi	Subnational Level 1	High SDI
Kumamoto	Subnational Level 1	High SDI
Kyōto	Subnational Level 1	High SDI
Mie	Subnational Level 1	High SDI
Miyagi	Subnational Level 1	High SDI
Miyazaki	Subnational Level 1	High SDI
Nagano	Subnational Level 1	High SDI
Nagasaki	Subnational Level 1	High SDI
Nara	Subnational Level 1	High SDI
Niigata	Subnational Level 1	High SDI
Ōita	Subnational Level 1	High SDI
Okayama	Subnational Level 1	High SDI

Geography	Level in hierarchy	SDI quintile
Haringey	Subnational Level 3	High SDI
Harrow	Subnational Level 3	High SDI
Havering	Subnational Level 3	High SDI
Hillingdon	Subnational Level 3	High SDI
Hounslow	Subnational Level 3	High SDI
Islington	Subnational Level 3	High SDI
Kensington and Chelsea	Subnational Level 3	High SDI
Kingston upon Thames	Subnational Level 3	High SDI
Lambeth	Subnational Level 3	High SDI
Lewisham	Subnational Level 3	High SDI
Merton	Subnational Level 3	High SDI
Newham	Subnational Level 3	High SDI
Redbridge	Subnational Level 3	High SDI
Richmond upon Thames	Subnational Level 3	High SDI
Southwark	Subnational Level 3	High SDI
Sutton	Subnational Level 3	High SDI
Tower Hamlets	Subnational Level 3	High SDI
Waltham Forest	Subnational Level 3	High SDI
Wandsworth	Subnational Level 3	High SDI
Westminster	Subnational Level 3	High SDI
North East England	Subnational Level 2	High SDI
County Durham	Subnational Level 3	High SDI
Darlington	Subnational Level 3	High SDI
Gateshead	Subnational Level 3	High SDI
Hartlepool	Subnational Level 3	High SDI
Middlesbrough	Subnational Level 3	High SDI
Newcastle upon Tyne	Subnational Level 3	High SDI
North Tyneside	Subnational Level 3	High SDI
Northumberland	Subnational Level 3	High SDI
Redcar and Cleveland	Subnational Level 3	High SDI
South Tyneside	Subnational Level 3	High SDI
Stockton-on-Tees	Subnational Level 3	High SDI
Sunderland	Subnational Level 3	High SDI
North West England	Subnational Level 2	High SDI
Blackburn with Darwen	Subnational Level 3	High SDI
Blackpool	Subnational Level 3	High SDI
Bolton	Subnational Level 3	High SDI
Bury	Subnational Level 3	High SDI
Cheshire East	Subnational Level 3	High SDI
Cheshire West and Chester	Subnational Level 3	High SDI
Cumbria	Subnational Level 3	High SDI
Halton	Subnational Level 3	High SDI
Knowsley	Subnational Level 3	High SDI
Lancashire	Subnational Level 3	High SDI
Liverpool	Subnational Level 3	High SDI
Manchester	Subnational Level 3	High SDI
Oldham	Subnational Level 3	High SDI
Rochdale	Subnational Level 3	High SDI
Salford	Subnational Level 3	High SDI
Sefton	Subnational Level 3	High SDI
St Helens	Subnational Level 3	High SDI
Stockport	Subnational Level 3	High SDI
Tameside	Subnational Level 3	High SDI
Trafford	Subnational Level 3	High SDI
Warrington	Subnational Level 3	High SDI
Wigan	Subnational Level 3	High SDI
Wirral	Subnational Level 3	High SDI
South East England	Subnational Level 2	High SDI
Bracknell Forest	Subnational Level 3	High SDI
Brighton and Hove	Subnational Level 3	High SDI
Buckinghamshire	Subnational Level 3	High SDI
East Sussex	Subnational Level 3	High SDI
Hampshire	Subnational Level 3	High SDI
Isle of Wight	Subnational Level 3	High SDI
Kent	Subnational Level 3	High SDI
Medway	Subnational Level 3	High SDI
Milton Keynes	Subnational Level 3	High SDI
Oxfordshire	Subnational Level 3	High SDI
Portsmouth	Subnational Level 3	High SDI
Reading	Subnational Level 3	High SDI
Slough	Subnational Level 3	High SDI
Southampton	Subnational Level 3	High SDI

Geography	Level in hierarchy	SDI quintile
Pernambuco	Subnational Level 1	Low-middle SDI
Piauí	Subnational Level 1	Low-middle SDI
Rio de Janeiro	Subnational Level 1	High-middle SDI
Rio Grande do Norte	Subnational Level 1	Low-middle SDI
Rio Grande do Sul	Subnational Level 1	Middle SDI
Rondônia	Subnational Level 1	Low-middle SDI
Roraima	Subnational Level 1	Middle SDI
Santa Catarina	Subnational Level 1	High-middle SDI
São Paulo	Subnational Level 1	High-middle SDI
Sergipe	Subnational Level 1	Low-middle SDI
Tocantins	Subnational Level 1	Low-middle SDI
Paraguay	Country	Middle SDI
North Africa and Middle East	Super region	-
North Africa and Middle East	Region	-
Afghanistan	Country	Low SDI
Algeria	Country	Middle SDI
Bahrain	Country	High-middle SDI
Egypt	Country	Middle SDI
Iran (Islamic Republic of)	Country	Middle SDI
Iraq	Country	Middle SDI
Jordan	Country	High-middle SDI
Kuwait	Country	High SDI
Lebanon	Country	High-middle SDI
Libya	Country	High-middle SDI
Morocco	Country	Low-middle SDI
Oman	Country	High-middle SDI
Palestine	Country	Low-middle SDI
Qatar	Country	High SDI
Saudi Arabia	Country	High SDI
Sudan	Country	Low-middle SDI
Syrian Arab Republic	Country	Middle SDI
Tunisia	Country	Middle SDI
Turkey	Country	High-middle SDI
United Arab Emirates	Country	High SDI
Yemen	Country	Low SDI
South Asia	Super region	-
South Asia	Region	-
Bangladesh	Country	Low-middle SDI
Bhutan	Country	Low-middle SDI
India	Country	Low-middle SDI
Nepal	Country	Low SDI
Pakistan	Country	Low SDI
Southeast Asia, East Asia, and Oceania	Super region	-
East Asia	Region	-
China	Country	Middle SDI
Democratic People's Republic of Korea	Country	Low-middle SDI
Taiwan (Province of China)	Country	High SDI
Oceania	Region	-
American Samoa	Country	High-middle SDI
Cook Islands	Country	High-middle SDI
Fiji	Country	Middle SDI
Guam	Country	High SDI
Kiribati	Country	Low-middle SDI
Marshall Islands	Country	Low-middle SDI
Micronesia (Federated States of)	Country	Low-middle SDI
Nauru	Country	Middle SDI
Niue	Country	High-middle SDI
Northern Mariana Islands	Country	High-middle SDI
Palau	Country	High-middle SDI
Papua New Guinea	Country	Low SDI
Samoa	Country	Middle SDI
Solomon Islands	Country	Low SDI
Tokelau	Country	Middle SDI
Tonga	Country	Middle SDI
Tuvalu	Country	Low-middle SDI
Vanuatu	Country	Low-middle SDI
Southeast Asia	Region	-
Cambodia	Country	Low-middle SDI
Indonesia	Country	Middle SDI
Aceh	Subnational Level 1	Middle SDI
Bali	Subnational Level 1	Middle SDI
Bangka-Belitung Islands	Subnational Level 1	Middle SDI

Supplementary Table 2. GBD location hierarchy, with SDI quintile

Geography	Level in hierarchy	SDI quintile
Okinawa	Subnational Level 1	High SDI
Ōsaka	Subnational Level 1	High SDI
Saga	Subnational Level 1	High SDI
Saitama	Subnational Level 1	High SDI
Shiga	Subnational Level 1	High SDI
Shimane	Subnational Level 1	High SDI
Shizuoka	Subnational Level 1	High SDI
Tochigi	Subnational Level 1	High SDI
Tokushima	Subnational Level 1	High SDI
Tōkyō	Subnational Level 1	High SDI
Tottori	Subnational Level 1	High SDI
Toyama	Subnational Level 1	High SDI
Wakayama	Subnational Level 1	High SDI
Yamagata	Subnational Level 1	High SDI
Yamaguchi	Subnational Level 1	High SDI
Yamanashi	Subnational Level 1	High SDI
Republic of Korea	Country	High SDI
Singapore	Country	High SDI
High-income North America	Region	-
Canada	Country	High SDI
Greenland	Country	High-middle SDI
United States of America	Country	High SDI
Alabama	Subnational Level 1	High SDI
Alaska	Subnational Level 1	High SDI
Arizona	Subnational Level 1	High SDI
Arkansas	Subnational Level 1	High SDI
California	Subnational Level 1	High SDI
Colorado	Subnational Level 1	High SDI
Connecticut	Subnational Level 1	High SDI
Delaware	Subnational Level 1	High SDI
District of Columbia	Subnational Level 1	High SDI
Florida	Subnational Level 1	High SDI
Georgia	Subnational Level 1	High SDI
Hawaii	Subnational Level 1	High SDI
Idaho	Subnational Level 1	High SDI
Illinois	Subnational Level 1	High SDI
Indiana	Subnational Level 1	High SDI
Iowa	Subnational Level 1	High SDI
Kansas	Subnational Level 1	High SDI
Kentucky	Subnational Level 1	High SDI
Louisiana	Subnational Level 1	High SDI
Maine	Subnational Level 1	High SDI
Maryland	Subnational Level 1	High SDI
Massachusetts	Subnational Level 1	High SDI
Michigan	Subnational Level 1	High SDI
Minnesota	Subnational Level 1	High SDI
Mississippi	Subnational Level 1	High-middle SDI
Missouri	Subnational Level 1	High SDI
Montana	Subnational Level 1	High SDI
Nebraska	Subnational Level 1	High SDI
Nevada	Subnational Level 1	High SDI
New Hampshire	Subnational Level 1	High SDI
New Jersey	Subnational Level 1	High SDI
New Mexico	Subnational Level 1	High SDI
New York	Subnational Level 1	High SDI
North Carolina	Subnational Level 1	High SDI
North Dakota	Subnational Level 1	High SDI
Ohio	Subnational Level 1	High SDI
Oklahoma	Subnational Level 1	High SDI
Oregon	Subnational Level 1	High SDI
Pennsylvania	Subnational Level 1	High SDI
Rhode Island	Subnational Level 1	High SDI
South Carolina	Subnational Level 1	High SDI
South Dakota	Subnational Level 1	High SDI
Tennessee	Subnational Level 1	High SDI
Texas	Subnational Level 1	High SDI
Utah	Subnational Level 1	High SDI
Vermont	Subnational Level 1	High SDI
Virginia	Subnational Level 1	High SDI
Washington	Subnational Level 1	High SDI
West Virginia	Subnational Level 1	High SDI
Wisconsin	Subnational Level 1	High SDI

Geography	Level in hierarchy	SDI quintile
Surrey	Subnational Level 3	High SDI
West Berkshire	Subnational Level 3	High SDI
West Sussex	Subnational Level 3	High SDI
Windsor and Maidenhead	Subnational Level 3	High SDI
Wokingham	Subnational Level 3	High SDI
South West England	Subnational Level 2	High SDI
Bath and North East Somerset	Subnational Level 3	High SDI
Bournemouth	Subnational Level 3	High SDI
Bristol, City of	Subnational Level 3	High SDI
Cornwall	Subnational Level 3	High SDI
Devon	Subnational Level 3	High SDI
Dorset	Subnational Level 3	High SDI
Gloucestershire	Subnational Level 3	High SDI
North Somerset	Subnational Level 3	High SDI
Plymouth	Subnational Level 3	High SDI
Poole	Subnational Level 3	High SDI
Somerset	Subnational Level 3	High SDI
South Gloucestershire	Subnational Level 3	High SDI
Swindon	Subnational Level 3	High SDI
Torbay	Subnational Level 3	High SDI
Wiltshire	Subnational Level 3	High SDI
West Midlands	Subnational Level 2	High SDI
Birmingham	Subnational Level 3	High SDI
Coventry	Subnational Level 3	High SDI
Dudley	Subnational Level 3	High SDI
Herefordshire, County of	Subnational Level 3	High SDI
Sandwell	Subnational Level 3	High SDI
Shropshire	Subnational Level 3	High SDI
Solihull	Subnational Level 3	High SDI
Staffordshire	Subnational Level 3	High SDI
Stoke-on-Trent	Subnational Level 3	High SDI
Telford and Wrekin	Subnational Level 3	High SDI
Walsall	Subnational Level 3	High SDI
Warwickshire	Subnational Level 3	High SDI
Wolverhampton	Subnational Level 3	High SDI
Worcestershire	Subnational Level 3	High SDI
Yorkshire and the Humber	Subnational Level 2	High SDI
Barnsley	Subnational Level 3	High SDI
Bradford	Subnational Level 3	High SDI
Calderdale	Subnational Level 3	High SDI
Doncaster	Subnational Level 3	High SDI
East Riding of Yorkshire	Subnational Level 3	High SDI
Kingston upon Hull, City of	Subnational Level 3	High SDI
Kirklees	Subnational Level 3	High SDI
Leeds	Subnational Level 3	High SDI
North East Lincolnshire	Subnational Level 3	High SDI
North Lincolnshire	Subnational Level 3	High SDI
North Yorkshire	Subnational Level 3	High SDI
Rotherham	Subnational Level 3	High SDI
Sheffield	Subnational Level 3	High SDI
Wakefield	Subnational Level 3	High SDI
York	Subnational Level 3	High SDI
Northern Ireland	Subnational Level 1	High SDI
Scotland	Subnational Level 1	High SDI
Wales	Subnational Level 1	High SDI
Latin America and Caribbean	Super region	-
Andean Latin America	Region	-
Bolivia (Plurinational State of)	Country	Low-middle SDI
Ecuador	Country	Middle SDI
Peru	Country	Middle SDI
Caribbean	Region	-
Antigua and Barbuda	Country	High-middle SDI
Bahamas	Country	High-middle SDI
Barbados	Country	High-middle SDI
Belize	Country	Low-middle SDI
Bermuda	Country	High SDI
Cuba	Country	Middle SDI
Dominica	Country	High-middle SDI
Dominican Republic	Country	Low-middle SDI
Grenada	Country	Middle SDI
Guyana	Country	Middle SDI
Haiti	Country	Low SDI

Geography	Level in hierarchy	SDI quintile
Banten	Subnational Level 1	Middle SDI
Bengkulu	Subnational Level 1	Middle SDI
Gorontalo	Subnational Level 1	Low-middle SDI
Jakarta	Subnational Level 1	High-middle SDI
Jambi	Subnational Level 1	Middle SDI
West Java	Subnational Level 1	Middle SDI
Central Java	Subnational Level 1	Middle SDI
East Java	Subnational Level 1	Middle SDI
West Kalimantan	Subnational Level 1	Low-middle SDI
South Kalimantan	Subnational Level 1	Middle SDI
Central Kalimantan	Subnational Level 1	Middle SDI
East Kalimantan	Subnational Level 1	High-middle SDI
North Kalimantan	Subnational Level 1	High-middle SDI
Riau Islands	Subnational Level 1	High-middle SDI
Lampung	Subnational Level 1	Middle SDI
Maluku	Subnational Level 1	Low-middle SDI
North Maluku	Subnational Level 1	Low-middle SDI
West Nusa Tenggara	Subnational Level 1	Low-middle SDI
East Nusa Tenggara	Subnational Level 1	Low-middle SDI
Papua	Subnational Level 1	Middle SDI
West Papua	Subnational Level 1	Middle SDI
Riau	Subnational Level 1	High-middle SDI
West Sulawesi	Subnational Level 1	Low-middle SDI
South Sulawesi	Subnational Level 1	Middle SDI
Central Sulawesi	Subnational Level 1	Middle SDI
Southeast Sulawesi	Subnational Level 1	Middle SDI
North Sulawesi	Subnational Level 1	Middle SDI
West Sumatra	Subnational Level 1	Middle SDI
South Sumatra	Subnational Level 1	Middle SDI
North Sumatra	Subnational Level 1	Middle SDI
Yogyakarta	Subnational Level 1	Middle SDI
Lao People's Democratic Republic	Country	Low-middle SDI
Malaysia	Country	High-middle SDI
Maldives	Country	Low-middle SDI
Mauritius	Country	High-middle SDI
Myanmar	Country	Low-middle SDI
Philippines	Country	Middle SDI
Seychelles	Country	High-middle SDI
Sri Lanka	Country	High-middle SDI
Thailand	Country	Middle SDI
Timor-Leste	Country	Low-middle SDI
Viet Nam	Country	Middle SDI
Sub-Saharan Africa	Super region	-
Central Sub-Saharan Africa	Region	-
Angola	Country	Low-middle SDI
Central African Republic	Country	Low SDI
Congo	Country	Low-middle SDI
Democratic Republic of the Congo	Country	Low SDI
Equatorial Guinea	Country	Middle SDI
Gabon	Country	Middle SDI
Eastern Sub-Saharan Africa	Region	-
Burundi	Country	Low SDI
Comoros	Country	Low SDI
Djibouti	Country	Low-middle SDI
Eritrea	Country	Low SDI
Ethiopia	Country	Low SDI
Kenya	Country	Low-middle SDI
Baringo	Subnational Level 1	Low-middle SDI
Bomet	Subnational Level 1	Low-middle SDI
Bungoma	Subnational Level 1	Low-middle SDI
Busia	Subnational Level 1	Low-middle SDI
Elgeyo-Marakwet	Subnational Level 1	Low-middle SDI
Embu	Subnational Level 1	Low-middle SDI
Garissa	Subnational Level 1	Low-middle SDI
HomaBay	Subnational Level 1	Low-middle SDI
Isiolo	Subnational Level 1	Low-middle SDI
Kajiado	Subnational Level 1	Low-middle SDI
Kakamega	Subnational Level 1	Low-middle SDI
Kericho	Subnational Level 1	Low-middle SDI
Kiambu	Subnational Level 1	Low-middle SDI
Kilifi	Subnational Level 1	Low-middle SDI
Kirinyaga	Subnational Level 1	Low-middle SDI

Supplementary Table 2. GBD location hierarchy, with SDI quintile

Geography	Level in hierarchy	SDI quintile
Wyoming	Subnational Level 1	High SDI
Southern Latin America	Region	-
Argentina	Country	High-middle SDI
Chile	Country	High-middle SDI
Uruguay	Country	High-middle SDI
Western Europe	Region	-
Andorra	Country	High SDI
Austria	Country	High SDI
Belgium	Country	High SDI
Cyprus	Country	High SDI
Denmark	Country	High SDI
Finland	Country	High SDI
France	Country	High SDI
Germany	Country	High SDI
Greece	Country	High-middle SDI
Iceland	Country	High SDI
Ireland	Country	High SDI
Israel	Country	High-middle SDI
Italy	Country	High-middle SDI
Luxembourg	Country	High SDI
Malta	Country	High-middle SDI
Monaco	Country	High SDI
Netherlands	Country	High SDI
Norway	Country	High SDI
Portugal	Country	High-middle SDI
San Marino	Country	High SDI
Spain	Country	High-middle SDI
Sweden	Country	High SDI
Stockholm	Subnational Level 1	High SDI
Sweden except Stockholm	Subnational Level 1	High SDI
Switzerland	Country	High SDI
United Kingdom	Country	High SDI
England	Subnational Level 1	High SDI
East Midlands	Subnational Level 2	High SDI
Derby	Subnational Level 3	High SDI
Derbyshire	Subnational Level 3	High SDI
Leicester	Subnational Level 3	High SDI
Leicestershire	Subnational Level 3	High SDI
Lincolnshire	Subnational Level 3	High SDI
Northamptonshire	Subnational Level 3	High SDI
Nottingham	Subnational Level 3	High SDI
Nottinghamshire	Subnational Level 3	High SDI
Rutland	Subnational Level 3	High SDI
East of England	Subnational Level 2	High SDI
Bedford	Subnational Level 3	High SDI
Cambridgeshire	Subnational Level 3	High SDI
Central Bedfordshire	Subnational Level 3	High SDI
Essex	Subnational Level 3	High SDI
Hertfordshire	Subnational Level 3	High SDI
Luton	Subnational Level 3	High SDI
Norfolk	Subnational Level 3	High SDI
Peterborough	Subnational Level 3	High SDI
Southend-on-Sea	Subnational Level 3	High SDI
Suffolk	Subnational Level 3	High SDI
Thurrock	Subnational Level 3	High SDI
Greater London	Subnational Level 2	High SDI
Barking and Dagenham	Subnational Level 3	High SDI
Barnet	Subnational Level 3	High SDI
Bexley	Subnational Level 3	High SDI
Brent	Subnational Level 3	High SDI
Bromley	Subnational Level 3	High SDI
Camden	Subnational Level 3	High SDI
Croydon	Subnational Level 3	High SDI
Ealing	Subnational Level 3	High SDI
Enfield	Subnational Level 3	High SDI
Greenwich	Subnational Level 3	High SDI
Hackney	Subnational Level 3	High SDI
Hammersmith and Fulham	Subnational Level 3	High SDI

Geography	Level in hierarchy	SDI quintile
Jamaica	Country	Middle SDI
Puerto Rico	Country	High SDI
Saint Kitts and Nevis	Country	High-middle SDI
Saint Lucia	Country	Middle SDI
Saint Vincent and the Grenadines	Country	Middle SDI
Suriname	Country	Middle SDI
Trinidad and Tobago	Country	High-middle SDI
United States Virgin Islands	Country	High-middle SDI
Central Latin America	Region	-
Colombia	Country	Middle SDI
Costa Rica	Country	Middle SDI
El Salvador	Country	Low-middle SDI
Guatemala	Country	Low-middle SDI
Honduras	Country	Low-middle SDI
Mexico	Country	Middle SDI
Aguascalientes	Subnational Level 1	Middle SDI
Baja California	Subnational Level 1	Middle SDI
Baja California Sur	Subnational Level 1	Middle SDI
Campeche	Subnational Level 1	Middle SDI
Chiapas	Subnational Level 1	Middle SDI
Chihuahua	Subnational Level 1	Middle SDI
Coahuila	Subnational Level 1	Middle SDI
Colima	Subnational Level 1	Middle SDI
Durango	Subnational Level 1	Middle SDI
Guanajuato	Subnational Level 1	Middle SDI
Guerrero	Subnational Level 1	Middle SDI
Hidalgo	Subnational Level 1	Middle SDI
Jalisco	Subnational Level 1	Middle SDI
México	Subnational Level 1	Middle SDI
Mexico City	Subnational Level 1	Middle SDI
Michoacán de Ocampo	Subnational Level 1	Middle SDI
Morelos	Subnational Level 1	Middle SDI
Nayarit	Subnational Level 1	Middle SDI
Nuevo León	Subnational Level 1	Middle SDI
Oaxaca	Subnational Level 1	Middle SDI
Puebla	Subnational Level 1	Middle SDI
Querétaro	Subnational Level 1	Middle SDI
Quintana Roo	Subnational Level 1	Middle SDI
San Luis Potosí	Subnational Level 1	Middle SDI
Sinaloa	Subnational Level 1	Middle SDI
Sonora	Subnational Level 1	Middle SDI
Tabasco	Subnational Level 1	Middle SDI
Tamaulipas	Subnational Level 1	Middle SDI
Tlaxcala	Subnational Level 1	Middle SDI
Veracruz de Ignacio de la Llave	Subnational Level 1	Middle SDI
Yucatán	Subnational Level 1	Middle SDI
Zacatecas	Subnational Level 1	Middle SDI
Nicaragua	Country	Low-middle SDI
Panama	Country	Middle SDI
Venezuela (Bolivarian Republic of)	Country	Low-middle SDI
Tropical Latin America	Region	-
Brazil	Country	Middle SDI
Acre	Subnational Level 1	Low-middle SDI
Alagoas	Subnational Level 1	Low-middle SDI
Amapá	Subnational Level 1	Middle SDI
Amazonas	Subnational Level 1	Low-middle SDI
Bahia	Subnational Level 1	Low-middle SDI
Ceará	Subnational Level 1	Low-middle SDI
Distrito Federal	Subnational Level 1	High-middle SDI
Espírito Santo	Subnational Level 1	Middle SDI
Goiás	Subnational Level 1	Middle SDI
Maranhão	Subnational Level 1	Low SDI
Mato Grosso	Subnational Level 1	Middle SDI
Mato Grosso do Sul	Subnational Level 1	Middle SDI
Minas Gerais	Subnational Level 1	Middle SDI
Pará	Subnational Level 1	Low-middle SDI
Paraíba	Subnational Level 1	Low-middle SDI
Paraná	Subnational Level 1	Middle SDI

Geography	Level in hierarchy	SDI quintile
Kisii	Subnational Level 1	Low-middle SDI
Kisumu	Subnational Level 1	Low-middle SDI
Kitui	Subnational Level 1	Low-middle SDI
Kwale	Subnational Level 1	Low-middle SDI
Laikipia	Subnational Level 1	Low-middle SDI
Lamu	Subnational Level 1	Low-middle SDI
Machakos	Subnational Level 1	Low-middle SDI
Makueni	Subnational Level 1	Low-middle SDI
Mandera	Subnational Level 1	Low-middle SDI
Marsabit	Subnational Level 1	Low-middle SDI
Meru	Subnational Level 1	Low-middle SDI
Migori	Subnational Level 1	Low-middle SDI
Mombasa	Subnational Level 1	Low-middle SDI
Murang'a	Subnational Level 1	Low-middle SDI
Nairobi	Subnational Level 1	Low-middle SDI
Nakuru	Subnational Level 1	Low-middle SDI
Nandi	Subnational Level 1	Low-middle SDI
Narok	Subnational Level 1	Low-middle SDI
Nyamira	Subnational Level 1	Low-middle SDI
Nyandarua	Subnational Level 1	Low-middle SDI
Nyeri	Subnational Level 1	Low-middle SDI
Samburu	Subnational Level 1	Low-middle SDI
Siaya	Subnational Level 1	Low-middle SDI
TaitaTaveta	Subnational Level 1	Low-middle SDI
TanaRiver	Subnational Level 1	Low-middle SDI
TharakaNithi	Subnational Level 1	Low-middle SDI
TransNzoia	Subnational Level 1	Low-middle SDI
Turkana	Subnational Level 1	Low-middle SDI
UasinGishu	Subnational Level 1	Low-middle SDI
Vihiga	Subnational Level 1	Low-middle SDI
Wajir	Subnational Level 1	Low-middle SDI
WestPokot	Subnational Level 1	Low-middle SDI
Madagascar	Country	Low SDI
Malawi	Country	Low SDI
Mozambique	Country	Low SDI
Rwanda	Country	Low SDI
Somalia	Country	Low SDI
South Sudan	Country	Low SDI
Uganda	Country	Low SDI
United Republic of Tanzania	Country	Low SDI
Zambia	Country	Low-middle SDI
Southern Sub-Saharan Africa	Region	-
Botswana	Country	Middle SDI
Eswatini	Country	Low-middle SDI
Lesotho	Country	Low-middle SDI
Namibia	Country	Middle SDI
South Africa	Country	Middle SDI
Zimbabwe	Country	Low-middle SDI
Western Sub-Saharan Africa	Region	-
Benin	Country	Low SDI
Burkina Faso	Country	Low SDI
Cabo Verde	Country	Low-middle SDI
Cameroon	Country	Low-middle SDI
Chad	Country	Low SDI
Côte d'Ivoire	Country	Low SDI
Gambia	Country	Low SDI
Ghana	Country	Low-middle SDI
Guinea	Country	Low SDI
Guinea-Bissau	Country	Low SDI
Liberia	Country	Low SDI
Mali	Country	Low SDI
Mauritania	Country	Low-middle SDI
Niger	Country	Low SDI
Nigeria	Country	Low-middle SDI
Sao Tome and Principe	Country	Low-middle SDI
Senegal	Country	Low SDI
Sierra Leone	Country	Low SDI
Togo	Country	Low SDI

Supplementary Table 3. List of GBD causes, with levels

Cause name	Level in hierarchy	Cause name	Level in hierarchy	Cause name	Level in hierarchy
All causes	0	Testicular cancer	3	Bacterial skin diseases	3
Communicable, maternal, neonatal, and nutritional	1	Kidney cancer	3	Cellulitis	4
HIV/AIDS and sexually transmitted infections	2	Bladder cancer	3	Pyoderma	4
HIV/AIDS	3	Brain and central nervous system cancer	3	Scabies	3
HIV/AIDS - Drug-susceptible	4	Thyroid cancer	3	Fungal skin diseases	3
HIV/AIDS - Multidrug-resistant Tuberculosis	4	Mesothelioma	3	Viral skin diseases	3
HIV/AIDS - Extensively drug-resistant	4	Hodgkin lymphoma	3	Acne vulgaris	3
HIV/AIDS resulting in other diseases	4	Non-Hodgkin lymphoma	3	Alopecia areata	3
Sexually transmitted infections excluding HIV	3	Multiple myeloma	3	Pruritus	3
Syphilis	4	Leukemia	3	Urticaria	3
Chlamydial infection	4	Acute lymphoid leukemia	4	Decubitus ulcer	3
Gonococcal infection	4	Chronic lymphoid leukemia	4	Other skin and subcutaneous diseases	3
Trichomoniasis	4	Acute myeloid leukemia	4	Sense organ diseases	2
Genital herpes	4	Chronic myeloid leukemia	4	Blindness and vision loss	3
Other sexually transmitted infections	4	Other leukemia	4	Glaucoma	4
Respiratory infections and tuberculosis	2	Other malignant neoplasms	3	Cataract	4
Tuberculosis	3	Other neoplasms	3	Age-related macular degeneration	4
Latent tuberculosis infection	4	Myelodysplastic, myeloproliferative,	4	Refraction disorders	4
Drug-susceptible tuberculosis	4	Benign and in situ intestinal neoplasms	4	Near vision loss	4
Multidrug-resistant tuberculosis without	4	Benign and in situ cervical and uterine	4	Other vision loss	4
Extensively drug-resistant tuberculosis	4	Other benign and in situ neoplasms	4	Age-related and other hearing loss	3
Lower respiratory infections	3	Cardiovascular diseases	2	Other sense organ diseases	3
Upper respiratory infections	3	Rheumatic heart disease	3	Musculoskeletal disorders	2
Otitis media	3	Ischemic heart disease	3	Rheumatoid arthritis	3
Enteric infections	2	Stroke	3	Osteoarthritis	3
Diarrheal diseases	3	Ischemic stroke	4	Osteoarthritis hip	4
Typhoid and paratyphoid	3	Intracerebral hemorrhage	4	Osteoarthritis knee	4
Typhoid fever	4	Subarachnoid hemorrhage	4	Osteoarthritis hand	4
Paratyphoid fever	4	Hypertensive heart disease	3	Osteoarthritis other	4
Invasive Non-typhoidal Salmonella (iNTS)	3	Non-rheumatic valvular heart disease	3	Low back pain	3
Other intestinal infectious diseases	3	Non-rheumatic calcific aortic valve disease	4	Neck pain	3
Neglected tropical diseases and malaria	2	Non-rheumatic degenerative mitral	4	Gout	3
Malaria	3	Other non-rheumatic valve diseases	4	Other musculoskeletal disorders	3
Chagas disease	3	Cardiomyopathy and myocarditis	3	Other non-communicable diseases	2
Leishmaniasis	3	Myocarditis	4	Congenital birth defects	3
Visceral leishmaniasis	4	Alcoholic cardiomyopathy	4	Neural tube defects	4
Cutaneous and mucocutaneous	4	Other cardiomyopathy	4	Congenital heart anomalies	4
African trypanosomiasis	3	Atrial fibrillation and flutter	3	Orofacial clefts	4
Schistosomiasis	3	Aortic aneurysm	3	Down syndrome	4
Cysticercosis	3	Peripheral artery disease	3	Turner syndrome	4
Cystic echinococcosis	3	Endocarditis	3	Klinefelter syndrome	4
Lymphatic filariasis	3	Other cardiovascular and circulatory diseases	3	Other chromosomal abnormalities	4
Onchocerciasis	3	Chronic respiratory diseases	2	Congenital musculoskeletal and	4
Trachoma	3	Chronic obstructive pulmonary disease	3	Urogenital congenital anomalies	4
Dengue	3	Pneumoconiosis	3	Digestive congenital anomalies	4

Supplementary Table 3. List of GBD causes, with levels

Cause name	Level in hierarchy
Yellow fever	3
Rabies	3
Intestinal nematode infections	3
Ascariasis	4
Trichuriasis	4
Hookworm disease	4
Food-borne trematodiases	3
Leprosy	3
Ebola	3
Zika virus	3
Guinea worm disease	3
Other neglected tropical diseases	3
Other infectious diseases	2
Meningitis	3
Encephalitis	3
Diphtheria	3
Whooping cough	3
Tetanus	3
Measles	3
Varicella and herpes zoster	3
Acute hepatitis	3
Acute hepatitis A	4
Acute hepatitis B	4
Acute hepatitis C	4
Acute hepatitis E	4
Other unspecified infectious diseases	3
Maternal and neonatal disorders	2
Maternal disorders	3
Maternal hemorrhage	4
Maternal sepsis and other maternal	4
Maternal hypertensive disorders	4
Maternal obstructed labor and uterine	4
Maternal abortion and miscarriage	4
Ectopic pregnancy	4
Indirect maternal deaths	4
Late maternal deaths	4
Maternal deaths aggravated by	4
Other maternal disorders	4
Neonatal disorders	3
Neonatal preterm birth	4
Neonatal encephalopathy due to	4
Neonatal sepsis and other neonatal	4
Hemolytic disease and other neonatal	4
Other neonatal disorders	4
Nutritional deficiencies	2

Cause name	Level in hierarchy
Silicosis	4
Asbestosis	4
Coal workers pneumoconiosis	4
Other pneumoconiosis	4
Asthma	3
Interstitial lung disease and pulmonary	3
Other chronic respiratory diseases	3
Digestive diseases	2
Cirrhosis and other chronic liver diseases	3
Cirrhosis and other chronic liver diseases	4
Cirrhosis and other chronic liver diseases	4
Cirrhosis and other chronic liver diseases	4
Cirrhosis and other chronic liver diseases	4
Cirrhosis and other chronic liver diseases	4
Upper digestive system diseases	3
Peptic ulcer disease	4
Gastritis and duodenitis	4
Gastroesophageal reflux disease	4
Appendicitis	3
Paralytic ileus and intestinal obstruction	3
Inguinal, femoral, and abdominal hernia	3
Inflammatory bowel disease	3
Vascular intestinal disorders	3
Gallbladder and biliary diseases	3
Pancreatitis	3
Other digestive diseases	3
Neurological disorders	2
Alzheimer's disease and other dementias	3
Parkinson's disease	3
Idiopathic epilepsy	3
Multiple sclerosis	3
Motor neuron disease	3
Headache disorders	3
Migraine	4
Tension-type headache	4
Other neurological disorders	3
Mental disorders	2
Schizophrenia	3
Depressive disorders	3
Major depressive disorder	4
Dysthymia	4
Bipolar disorder	3
Anxiety disorders	3
Eating disorders	3
Anorexia nervosa	4

Cause name	Level in hierarchy
Other congenital birth defects	4
Urinary diseases and male infertility	3
Urinary tract infections and interstitial	4
Urolithiasis	4
Benign prostatic hyperplasia	4
Male infertility	4
Other urinary diseases	4
Gynecological diseases	3
Uterine fibroids	4
Polycystic ovarian syndrome	4
Female infertility	4
Endometriosis	4
Genital prolapse	4
Premenstrual syndrome	4
Other gynecological diseases	4
Hemoglobinopathies and hemolytic anemias	3
Thalassemias	4
Thalassemias trait	4
Sickle cell disorders	4
Sickle cell trait	4
G6PD deficiency	4
G6PD trait	4
Other hemoglobinopathies	4
Endocrine, metabolic, blood, and immune	3
Oral disorders	3
Caries of deciduous teeth	4
Caries of permanent teeth	4
Periodontal diseases	4
Edentulism	4
Other oral disorders	4
Sudden infant death syndrome	3
Injuries	1
Transport injuries	2
Road injuries	3
Pedestrian road injuries	4
Cyclist road injuries	4
Motorcyclist road injuries	4
Motor vehicle road injuries	4
Other road injuries	4
Other transport injuries	3
Unintentional injuries	2
Falls	3
Drowning	3
Fire, heat, and hot substances	3
Poisonings	3

Supplementary Table 3. List of GBD causes, with levels

Cause name	Level in hierarchy		Cause name	Level in hierarchy		Cause name	Level in hierarchy
Protein-energy malnutrition	3		Bulimia nervosa	4		Poisoning by carbon monoxide	4
Iodine deficiency	3		Autism spectrum disorders	3		Poisoning by other means	4
Vitamin A deficiency	3		Attention-deficit/hyperactivity	3		Exposure to mechanical forces	3
Dietary iron deficiency	3		Conduct disorder	3		Unintentional firearm injuries	4
Other nutritional deficiencies	3		Idiopathic developmental	3		Other exposure to mechanical forces	4
Non-communicable diseases	1		Other mental disorders	3		Adverse effects of medical treatment	3
Neoplasms	2		Substance use disorders	2		Animal contact	3
Lip and oral cavity cancer	3		Alcohol use disorders	3		Venomous animal contact	4
Nasopharynx cancer	3		Drug use disorders	3		Non-venomous animal contact	4
Other pharynx cancer	3		Opioid use disorders	4		Foreign body	3
Esophageal cancer	3		Cocaine use disorders	4		Pulmonary aspiration and foreign body in	4
Stomach cancer	3		Amphetamine use disorders	4		Foreign body in eyes	4
Colon and rectum cancer	3		Cannabis use disorders	4		Foreign body in other body part	4
Liver cancer	3		Other drug use disorders	4		Environmental heat and cold exposure	3
Liver cancer due to hepatitis B	4		Diabetes and kidney diseases	2		Exposure to forces of nature	3
Liver cancer due to hepatitis C	4		Diabetes mellitus	3		Other unintentional injuries	3
Liver cancer due to alcohol use	4		Diabetes mellitus type 1	4		Self-harm and interpersonal violence	2
Liver cancer due to NASH	4		Diabetes mellitus type 2	4		Self-harm	3
Liver cancer due to other causes	4		Chronic kidney disease	3		Self-harm by firearm	4
Gallbladder and biliary tract cancer	3		Chronic kidney disease due to diabetes	4		Self-harm by other specified means	4
Pancreatic cancer	3		Chronic kidney disease due to diabetes	4		Interpersonal violence	3
Larynx cancer	3		Chronic kidney disease due to hypertension	4		Physical violence by firearm	4
Tracheal, bronchus, and lung cancer	3		Chronic kidney disease due to	4		Physical violence by sharp object	4
Malignant skin melanoma	3		Chronic kidney disease due to other and	4		Sexual violence	4
Non-melanoma skin cancer	3		Acute glomerulonephritis	3		Physical violence by other means	4
Non-melanoma skin cancer (squamous-cell	4		Skin and subcutaneous diseases	2		Conflict and terrorism	3
Non-melanoma skin cancer (basal-cell	4		Dermatitis	3		Executions and police conflict	3
Breast cancer	3		Atopic dermatitis	4		Total cancers	1
Cervical cancer	3		Contact dermatitis	4		Total burden related to hepatitis B	1
Uterine cancer	3		Seborrhoeic dermatitis	4		Total burden related to hepatitis C	1
Ovarian cancer	3		Psoriasis	3		Total burden related to Non-alcoholic fatty liver	1
Prostate cancer	3						

Supplementary Table 4. Cause-specific mortality rate for 70-79 years, 80-94 years, and 95+ years in 2019, and percentage change from 1990 to 2019 in death number and rate in 70+ years, global, for all GBD causes

Causes	Males					Females				
	Deaths per 100,000, 2019			Percentage change in 70+, 1990-2019		Deaths per 100,000, 2019			Percentage change in 70+, 1990-2019	
	70 to 79	80 to 94	95 plus	Number	Rate	70 to 79	80 to 94	95 plus	Number	Rate
All causes	4510 (4220 to 4790)	12000 (11500 to 12500)	29500 (29100 to 29900)	91.8 (81.1 to 103)	-22.4 (-26.8 to -18.1)	3110 (2930 to 3290)	9560 (9190 to 9950)	28200 (27800 to 28700)	75.1 (67.2 to 83.9)	-19.7 (-23.4 to -15.7)
Communicable, maternal, neonatal, and nutritional diseases	358 (321 to 424)	1030 (903 to 1190)	3520 (2750 to 4050)	31.7 (16.5 to 46)	-46.8 (-52.9 to -41)	283 (229 to 370)	810 (648 to 1030)	2640 (1970 to 3090)	37.5 (17.8 to 65.8)	-37 (-46 to -24)
HIV/AIDS and sexually transmitted infections	5.72 (5.02 to 6.67)	2.65 (2.17 to 3.15)	3.62 (2.15 to 5.56)	250 (141 to 438)	41.3 (-2.71 to 118)	4.35 (3.79 to 5.07)	1.83 (1.56 to 2.09)	2.84 (2.21 to 3.31)	227 (137 to 376)	49.9 (8.46 to 118)
HIV/AIDS	5.59 (4.89 to 6.55)	2.3 (1.84 to 2.78)	1.29 (1.17 to 1.45)	331 (175 to 653)	74.4 (11.2 to 205)	3.93 (3.37 to 4.66)	0.976 (0.795 to 1.23)	0.586 (0.504 to 0.696)	326 (170 to 644)	95.1 (23.9 to 241)
HIV/AIDS - Drug-susceptible Tuberculosis	1.01 (0.707 to 1.37)	0.336 (0.23 to 0.462)	0.202 (0.138 to 0.275)	105 (43.6 to 243)	-17.3 (-41.9 to 38.6)	0.88 (0.606 to 1.21)	0.2 (0.137 to 0.274)	0.108 (0.0742 to 0.146)	142 (68.8 to 297)	11 (-22.6 to 82.1)
HIV/AIDS - Multidrug-resistant Tuberculosis without extensive drug resistance	0.0964 (0.0401 to 0.186)	0.0325 (0.013 to 0.0635)	0.0176 (0.00716 to 0.0343)	2380 (1000 to 5520)	903 (346 to 2170)	0.0796 (0.032 to 0.152)	0.0188 (0.00747 to 0.0363)	0.0095 (0.00385 to 0.0183)	3830 (1820 to 8210)	1700 (779 to 3710)
HIV/AIDS - Extensively drug-resistant Tuberculosis	0.00421 (0.00148 to 0.00924)	0.002 (0.000603 to 0.00502)	0.00138 (0.000541 to 0.00279)			0.0023 (0.000891 to 0.00462)	0.000641 (0.000241 to 0.0014)	0.000458 (0.000172 to 0.00096)		
HIV/AIDS resulting in other diseases	4.47 (3.75 to 5.42)	1.93 (1.45 to 2.41)	1.07 (0.938 to 1.23)	457 (235 to 903)	125 (35.6 to 306)	2.97 (2.38 to 3.7)	0.757 (0.585 to 0.992)	0.468 (0.391 to 0.575)	430 (216 to 867)	143 (45.1 to 343)
Sexually transmitted infections excluding HIV	0.129 (0.0999 to 0.202)	0.358 (0.177 to 0.51)	2.33 (0.86 to 4.32)	-33.5 (-52.6 to -7.46)	-73.1 (-80.8 to -62.6)	0.424 (0.352 to 0.489)	0.854 (0.654 to 0.977)	2.26 (1.62 to 2.71)	53.7 (32.6 to 77.2)	-29.5 (-39.2 to -18.8)
Syphilis	0.0924 (0.0724 to 0.143)	0.151 (0.0901 to 0.199)	0.688 (0.36 to 1.1)	-43.2 (-56.3 to -19.7)	-77 (-82.4 to -67.5)	0.0373 (0.0297 to 0.0441)	0.121 (0.0823 to 0.144)	0.308 (0.18 to 0.419)	85.8 (50.2 to 128)	-14.8 (-31.1 to 4.67)
Chlamydial infection						0.064 (0.0535 to 0.0735)	0.162 (0.127 to 0.184)	0.455 (0.329 to 0.545)	55.3 (34.7 to 77.3)	-28.8 (-38.3 to -18.7)
Gonococcal infection	0.0097 (0.00702 to 0.0159)	0.0206 (0.0098 to 0.0295)	0.0234 (0.0125 to 0.037)	-32.8 (-55.4 to 8.21)	-72.8 (-82 to -56.3)	0.226 (0.19 to 0.259)	0.41 (0.323 to 0.466)	1.22 (0.88 to 1.43)	48.8 (29.1 to 70.2)	-31.8 (-40.8 to -22)
Trichomoniasis										
Genital herpes										
Other sexually transmitted infections	0.027 (0.0195 to 0.0446)	0.186 (0.0769 to 0.291)	1.62 (0.482 to 3.19)	-13 (-47.1 to 34.1)	-64.8 (-78.6 to -45.8)	0.0964 (0.0798 to 0.111)	0.161 (0.124 to 0.185)	0.276 (0.198 to 0.337)	50 (28.8 to 73.3)	-31.2 (-41 to -20.5)
Respiratory infections and tuberculosis	235 (218 to 254)	736 (648 to 799)	2650 (2070 to 2960)	50.6 (38.2 to 64)	-39.1 (-44.1 to -33.7)	151 (135 to 169)	494 (405 to 550)	1960 (1430 to 2230)	57.1 (39.8 to 78.1)	-28 (-35.9 to -18.3)
Tuberculosis	80.3 (72.4 to 89.1)	130 (115 to 142)	161 (128 to 181)	-14.1 (-25.4 to -3.86)	-65.3 (-69.8 to -61.1)	42.6 (36.8 to 53.3)	62.7 (52 to 79.7)	70.8 (52 to 94.6)	-12.9 (-32.8 to 37.8)	-60 (-69.2 to -36.8)
Latent tuberculosis infection										
Drug-susceptible tuberculosis	72.4 (62.4 to 81.5)	118 (102 to 132)	149 (118 to 169)	-21.4 (-33.3 to -9.83)	-68.2 (-73 to -63.5)	38.5 (32.5 to 48)	56.8 (46.3 to 73.1)	65.7 (48.4 to 87.5)	-20.2 (-39.2 to 26.3)	-63.4 (-72.1 to -42.1)
Multidrug-resistant tuberculosis without extensive drug resistance	7.43 (2.76 to 14.3)	11.1 (4.4 to 21.2)	10.8 (4.64 to 20.4)	580 (165 to 1710)	175 (7.22 to 631)	3.88 (1.43 to 7.77)	5.41 (2.03 to 11)	4.72 (2.04 to 8.92)	559 (162 to 1740)	202 (20.1 to 741)
Extensively drug-resistant tuberculosis	0.534 (0.224 to 1.06)	0.831 (0.358 to 1.58)	0.946 (0.419 to 1.78)			0.261 (0.105 to 0.518)	0.42 (0.178 to 0.823)	0.393 (0.175 to 0.76)		
Lower respiratory infections	154 (143 to 166)	605 (529 to 656)	2490 (1930 to 2780)	100 (83.4 to 116)	-19.1 (-25.8 to -12.7)	109 (94 to 121)	431 (351 to 479)	1880 (1380 to 2150)	90.5 (73.3 to 106)	-12.7 (-20.6 to -5.64)
Upper respiratory infections	0.356 (0.253 to 0.665)	1.49 (0.944 to 3.48)	2.57 (1.88 to 3.83)	-53.5 (-77.5 to 134)	-81.2 (-90.9 to -5.23)	0.236 (0.15 to 0.42)	1.24 (0.717 to 3.03)	4.74 (2.7 to 10.9)	-64.3 (-84.6 to 107)	-83.6 (-92.9 to -5.22)
Otitis media	0.0214 (0.0158 to 0.0319)	0.0425 (0.0323 to 0.0618)	0.0899 (0.0649 to 0.122)	-47.5 (-62.8 to -21.6)	-78.8 (-85 to -68.3)	0.0162 (0.0122 to 0.0254)	0.0331 (0.0252 to 0.0457)	0.0935 (0.0674 to 0.119)	-42.5 (-58.6 to 2.5)	-73.7 (-81 to -53)
Enteric infections	73.9 (43.6 to 142)	211 (133 to 387)	592 (380 to 1000)	-4.38 (-39.3 to 34.4)	-61.3 (-75.5 to -45.7)	89.7 (34.9 to 182)	243 (106 to 467)	436 (235 to 729)	11.5 (-32.3 to 90)	-48.9 (-69 to -12.9)
Diarrheal diseases	73 (42.6 to 141)	210 (132 to 386)	590 (379 to 998)	-4.45 (-39.7 to 34.8)	-61.4 (-75.6 to -45.5)	89 (34 to 181)	243 (105 to 466)	435 (234 to 728)	11.4 (-32.5 to 90.4)	-48.9 (-69.1 to -12.7)
Typhoid and paratyphoid	0.424 (0.171 to 0.843)	0.427 (0.185 to 0.798)	0.589 (0.236 to 1.34)	-16.5 (-29.6 to -0.268)	-66.2 (-71.5 to -59.7)	0.363 (0.15 to 0.704)	0.316 (0.138 to 0.605)	0.279 (0.119 to 0.636)	3.42 (-14.3 to 23)	-52.6 (-60.7 to -43.6)
Typhoid fever	0.328 (0.128 to 0.664)	0.336 (0.139 to 0.631)	0.451 (0.168 to 1.08)	-22.7 (-34.9 to -7.33)	-68.7 (-73.7 to -62.5)	0.281 (0.113 to 0.567)	0.251 (0.105 to 0.484)	0.219 (0.0851 to 0.52)	-1.57 (-19.3 to 17.8)	-54.9 (-63 to -46)
Paratyphoid fever	0.0954 (0.0312 to 0.211)	0.091 (0.0348 to 0.185)	0.138 (0.0579 to 0.307)	15.8 (-5.31 to 42.3)	-53.2 (-61.7 to -42.5)	0.0821 (0.0277 to 0.183)	0.0654 (0.0249 to 0.134)	0.0606 (0.0276 to 0.132)	26.1 (-0.0841 to 56)	-42.2 (-54.2 to -28.5)
Invasive Non-typhoidal Salmonella (iNTS)	0.47 (0.247 to 0.769)	0.55 (0.271 to 0.931)	0.96 (0.406 to 1.97)	31.8 (9.92 to 57.5)	-46.7 (-55.6 to -36.3)	0.341 (0.176 to 0.569)	0.352 (0.162 to 0.61)	0.458 (0.172 to 0.963)	22.8 (-0.792 to 45.8)	-43.7 (-54.5 to -33.1)
Other intestinal infectious diseases	0.0124 (0.000192 to 0.0309)	0.0469 (0.0035 to 0.106)	0.175 (0.000118 to 0.681)	51 (-63.5 to 613)	-39 (-85.3 to 188)	0.0132 (0.000315 to 0.0346)	0.0582 (0.00616 to 0.136)	0.254 (0.000782 to 0.806)	47.3 (-66.8 to 517)	-32.5 (-84.8 to 183)
Neglected tropical diseases and malaria	16.9 (9.38 to 27.7)	10.9 (7.67 to 15.5)	16.6 (9.97 to 26.3)	44.4 (-3.74 to 103)	-41.6 (-61.1 to -17.8)	14 (7.67 to 23.9)	8.08 (5.42 to 11.5)	7.86 (4.96 to 13)	60.8 (7.77 to 125)	-26.3 (-50.6 to 3.17)
Malaria	13.2 (5.82 to 24.6)	4.23 (1.82 to 8.03)	0.0334 (0.0125 to 0.0789)	52 (-16.7 to 146)	-38.6 (-66.3 to -0.389)	11.1 (4.8 to 21.2)	2.92 (1.24 to 5.76)	0.0427 (0.0163 to 0.101)	68.3 (-6.4 to 166)	-22.8 (-57.1 to 21.8)
Chagas disease	0.914 (0.348 to 1.69)	1.74 (0.758 to 3.44)	6.38 (2.48 to 14.8)	15.5 (-19.2 to 133)	-53.3 (-67.3 to -5.9)	0.647 (0.235 to 1.18)	1.34 (0.574 to 2.81)	3.13 (1.39 to 7.8)	8.15 (-21.5 to 114)	-50.4 (-64 to -1.97)
Leishmaniasis	0.038 (0.00413 to 0.105)	0.0388 (0.00369 to 0.109)	0.0901 (0.00484 to 0.25)	-84.8 (-93 to -80.5)	-93.8 (-97.2 to -92.1)	0.0195 (0.00199 to 0.0517)	0.0167 (0.00141 to 0.0456)	0.0217 (0.000953 to 0.0599)	-82 (-91.8 to -75.7)	-91.8 (-96.3 to -88.8)
Visceral leishmaniasis	0.038 (0.00413 to 0.105)	0.0388 (0.00369 to 0.109)	0.0901 (0.00484 to 0.25)	-84.8 (-93 to -80.5)	-93.8 (-97.2 to -92.1)	0.0195 (0.00199 to 0.0517)	0.0167 (0.00141 to 0.0456)	0.0217 (0.000953 to 0.0599)	-82 (-91.8 to -75.7)	-91.8 (-96.3 to -88.8)
Cutaneous and mucocutaneous leishmaniasis										
African trypanosomiasis	0.0102 (0.00177 to 0.0383)	0.0093 (0.00172 to 0.036)	0.00658 (0.000684 to 0.0375)	-93.4 (-98.6 to -71.1)	-97.3 (-99.4 to -88.3)	0.00932 (0.00168 to 0.0351)	0.00712 (0.00144 to 0.0242)	0.00295 (0.000373 to 0.0121)	-92.6 (-98.3 to -68.6)	-96.6 (-99.2 to -85.6)
Schistosomiasis	0.684 (0.575 to 0.817)	0.759 (0.626 to 0.893)	0.53 (0.325 to 0.785)	-26.3 (-39.3 to -10.3)	-70.2 (-75.5 to -63.7)	0.549 (0.457 to 0.656)	0.608 (0.489 to 0.724)	0.338 (0.212 to 0.501)	-23.9 (-36.6 to -7.4)	-65.1 (-70.9 to -57.5)
Cysticercosis	0.0166 (0.000339 to 0.0434)	0.0257 (0.000876 to 0.0684)	0.0474 (0.0000135 to 0.168)	11.2 (-87.7 to 910)	-55 (-95 to 308)	0.0176 (0.000774 to 0.0494)	0.0268 (0.0012 to 0.0664)	0.0371 (0.0000123 to 0.139)	16.1 (-83 to 713)	-46.8 (-92.2 to 273)
Cystic echinococcosis	0.0587 (0.013 to 0.127)	0.101 (0.0176 to 0.224)	0.202 (0.00741 to 0.742)	1.09 (-68.7 to 212)	-59.1 (-87.3 to 26.2)	0.0513 (0.00926 to 0.115)	0.0896 (0.0159 to 0.199)	0.156 (0.00852 to 0.489)	10.3 (-70.1 to 274)	-49.5 (-86.3 to 71.3)
Lymphatic filariasis										
Onchocerciasis										
Trachoma										
Dengue	1.09 (0.24 to 1.54)	2.99 (0.641 to 3.99)	6.51 (1.83 to 9.16)	336 (215 to 499)	76.3 (27.3 to 142)	1.11 (0.21 to 1.55)	2.52 (0.533 to 3.55)	2.65 (0.909 to 3.91)	435 (209 to 725)	145 (41.7 to 278)
Yellow fever	0.0198 (0.00726 to 0.0445)	0.0168 (0.00621 to 0.0389)	0.0124 (0.00414 to 0.0298)	-75 (-81.7 to -64.8)	-89.9 (-92.6 to -85.8)	0.00718 (0.00255 to 0.0162)	0.00502 (0.00176 to 0.0109)	0.00247 (0.000802 to 0.0063)	-76.9 (-83.2 to -68.2)	-89.4 (-92.3 to -85.4)
Rabies	0.343 (0.126 to 0.534)	0.246 (0.0969 to 0.355)	0.355 (0.171 to 0.606)	-6.51 (-49.7 to 49.3)	-62.2 (-79.7 to -39.6)	0.201 (0.0984 to 0.34)	0.126 (0.0515 to 0.206)	0.25 (0.0917 to 0.438)	3.39 (-30.5 to 48.1)	-52.6 (-68.2 to -32.1)
Intestinal nematode infections	0.0167 (0.0136 to 0.0202)	0.0273 (0.0219 to 0.033)	0.0856 (0.0575 to 0.117)	2.62 (-16.6 to 24.5)	-58.5 (-66.3 to -49.7)	0.0154 (0.0124 to 0.0186)	0.0258 (0.0204 to 0.031)	0.0615 (0.0405 to 0.0869)	8.3 (-12.9 to 32.4)	-50.4 (-60.1 to -39.3)
Ascariasis	0.0167 (0.0136 to 0.0202)	0.0273 (0.0219 to 0.033)	0.0856 (0.0575 to 0.117)	2.62 (-16.6 to 24.5)	-58.5 (-66.3 to -49.7)	0.0154 (0.0124 to 0.0186)	0.0258 (0.0204 to 0.031)	0.0615 (0.0405 to 0.0869)	8.3 (-12.9 to 32.4)	-50.4 (-60.1 to -39.3)
Trichuriasis										
Hookworm disease										
Food-borne trematodiases										
Leprosy										
Ebola	0.0153 (0.0125 to 0.018)	0.00733 (0.00601 to 0.00865)	0.109 (0.0892 to 0.128)			0.00411 (0.00337 to 0.00485)	0.0118 (0.00971 to 0.014)	0.0992 (0.0814 to 0.117)		
Zika virus	0.0000346 (0.0000207 to 0.0000523)	0.0000303 (0.0000179 to 0.0000461)	0.0000354 (0.0000197 to 0.0000543)			0.0000146 (0.00000631 to 0.0000252)	0.00000963 (0.00000417 to 0.000017)	0.00000644 (0.00000266 to 0.0000116)		
Guinea worm disease										
Other neglected tropical diseases	0.425 (0.286 to 0.954)	0.713 (0.481 to 1.51)	2.23 (1.25 to 3.81)	111 (72.6 to 187)	-14.7 (-30.2 to 16)	0.258 (0.142 to 0.434)	0.388 (0.228 to 0.66)	1.07 (0.568 to 1.74)	117 (57.6 to 187)	-0.416 (-27.8 to 31.4)
Other infectious diseases	16.5 (14.7 to 18.8)	30.6 (26.1 to 34.2)	59.3 (45.3 to 68.4)	19.3 (-1.56 to 44.6)	-51.8 (-60.2 to -41.5)	13.9 (12 to 15.7)	23.8 (19.5 to 27.3)	44.4 (32.2 to 53)	25.8 (8.56 to 44.4)	-42.3 (-50.2 to -33.8)

Supplementary Table 4. Cause-specific mortality rate for 70-79 years, 80-94 years, and 95+ years in 2019, and percentage change from 1990 to 2019 in death number and rate in 70+ years, global, for all GBD causes

	Males					Females				
	Deaths per 100,000, 2019			Percentage change in 70+, 1990-2019		Deaths per 100,000, 2019			Percentage change in 70+, 1990-2019	
Causes	70 to 79	80 to 94	95 plus	Number	Rate	70 to 79	80 to 94	95 plus	Number	Rate
Meningitis	4.25 (3.84 to 4.82)	7.56 (6.63 to 8.63)	11.1 (8.63 to 13.3)	19.5 (4.38 to 40.8)	-51.7 (-57.8 to -43.1)	4 (3.56 to 4.43)	5.95 (4.99 to 6.68)	9.7 (7.33 to 11.4)	18.3 (-0.812 to 38)	-45.8 (-54.5 to -36.7)
Encephalitis	4.42 (3.54 to 6.75)	8.52 (6.85 to 11.3)	10.5 (8.04 to 14.1)	73.1 (28.2 to 201)	-30 (-48.2 to 21.7)	4.05 (3.13 to 5.87)	6.91 (5.25 to 8.87)	6.52 (4.66 to 8.1)	129 (64.1 to 233)	4.98 (-24.8 to 52.9)
Diphtheria										
Whooping cough										
Tetanus	0.62 (0.31 to 1.01)	0.815 (0.434 to 1.38)	1.36 (0.802 to 2.69)	-64.7 (-76.4 to -36.7)	-85.7 (-90.4 to -74.4)	0.614 (0.33 to 0.861)	0.566 (0.255 to 0.792)	0.571 (0.289 to 0.827)	-66.1 (-77.8 to -41.4)	-84.5 (-89.8 to -73.1)
Measles	0.152 (0.138 to 0.167)	0.257 (0.233 to 0.282)	0.386 (0.351 to 0.425)			0.133 (0.121 to 0.146)	0.246 (0.224 to 0.271)	0.295 (0.268 to 0.324)		
Varicella and herpes zoster	0.413 (0.348 to 0.515)	2.57 (2.14 to 3.36)	13.7 (9.58 to 18.6)	23.5 (6.99 to 44.8)	-50.1 (-56.7 to -41.4)	0.385 (0.323 to 0.473)	2.47 (1.96 to 3.27)	13.9 (9.01 to 20.6)	30.3 (12.5 to 49)	-40.3 (-48.4 to -31.7)
Acute hepatitis	2.99 (2.42 to 3.8)	3.76 (3 to 4.67)	4.52 (3.12 to 6.16)	-24.8 (-47.7 to 17.9)	-69.6 (-78.9 to -52.3)	1.95 (1.61 to 2.5)	2.4 (1.96 to 3.12)	1.94 (1.42 to 2.55)	-34.6 (-53.8 to 24.2)	-70 (-78.8 to -43.1)
Acute hepatitis A	1.16 (0.759 to 1.65)	1.77 (1.2 to 2.42)	2.05 (1.18 to 3.28)	-42.3 (-60.8 to -3.29)	-76.7 (-84.2 to -60.9)	0.911 (0.498 to 1.33)	1.32 (0.789 to 1.86)	1.09 (0.652 to 1.6)	-41.8 (-61.5 to 23.9)	-73.3 (-82.3 to -43.2)
Acute hepatitis B	1.6 (1.16 to 2.27)	1.66 (1.18 to 2.31)	1.9 (1.13 to 3.02)	-0.282 (-41.4 to 81.3)	-59.7 (-76.3 to -26.7)	0.823 (0.545 to 1.26)	0.778 (0.467 to 1.29)	0.648 (0.397 to 1.05)	-19.3 (-51.6 to 60.7)	-63 (-77.8 to -26.3)
Acute hepatitis C	0.147 (0.0852 to 0.308)	0.201 (0.126 to 0.425)	0.256 (0.125 to 0.54)	-31.1 (-65.9 to 47.3)	-72.2 (-86.2 to -40.4)	0.179 (0.0965 to 0.317)	0.258 (0.16 to 0.402)	0.119 (0.0715 to 0.193)	-45.7 (-69.6 to 15.1)	-75.1 (-86.1 to -47.3)
Acute hepatitis E	0.0864 (0.0444 to 0.142)	0.126 (0.07 to 0.195)	0.31 (0.123 to 0.722)	-5.08 (-47.6 to 79.2)	-61.6 (-78.8 to -27.5)	0.036 (0.0156 to 0.0547)	0.0514 (0.0226 to 0.0801)	0.0796 (0.0363 to 0.147)	17.3 (-32.3 to 144)	-46.2 (-69 to 12.1)
Other unspecified infectious diseases	3.62 (2.41 to 4.39)	7.17 (4.38 to 8.71)	17.8 (10.4 to 23)	76.7 (18.7 to 120)	-28.6 (-52 to -11.2)	2.73 (1.78 to 3.24)	5.29 (3.34 to 6.52)	11.5 (7.51 to 14.5)	99 (58.3 to 143)	-8.76 (-27.4 to 11.3)
Maternal and neonatal disorders										
Maternal disorders										
Maternal hemorrhage										
Maternal sepsis and other maternal infections										
Maternal hypertensive disorders										
Maternal obstructed labor and uterine rupture										
Maternal abortion and miscarriage										
Ectopic pregnancy										
Indirect maternal deaths										
Late maternal deaths										
Maternal deaths aggravated by HIV/AIDS										
Other maternal disorders										
Neonatal disorders										
Neonatal preterm birth										
Neonatal encephalopathy due to birth asphyxia and trauma										
Neonatal sepsis and other neonatal infections										
Hemolytic disease and other neonatal jaundice										
Other neonatal disorders										
Nutritional deficiencies	10.1 (9.32 to 10.9)	40.8 (36.3 to 44)	196 (155 to 220)	-4.78 (-19.1 to 39.5)	-61.5 (-67.3 to -43.6)	9.4 (8.24 to 11.1)	38.6 (32.1 to 44.1)	196 (147 to 226)	14.9 (-18.9 to 87.4)	-47.3 (-62.8 to -14.1)
Protein-energy malnutrition	7.88 (7.23 to 8.58)	33 (28.8 to 35.8)	167 (129 to 189)	9.45 (-3.55 to 41.1)	-55.8 (-61 to -42.9)	6.86 (6.06 to 7.97)	31.2 (25.7 to 35.3)	169 (125 to 196)	31.5 (2.67 to 83.7)	-39.7 (-52.9 to -15.8)
Iodine deficiency										
Vitamin A deficiency										
Dietary iron deficiency										
Other nutritional deficiencies	2.19 (1.88 to 2.71)	7.82 (6.64 to 9.93)	29.1 (21.9 to 44.8)	-37.6 (-52.8 to 31.9)	-74.8 (-80.9 to -46.7)	2.55 (2.02 to 3.28)	7.43 (5.99 to 9.24)	27 (19.5 to 39.8)	-22.5 (-63.8 to 117)	-64.5 (-83.4 to -0.395)
Non-communicable diseases	3980 (3710 to 4240)	10600 (10100 to 11000)	25000 (24300 to 26000)	99.7 (87.5 to 112)	-19.3 (-24.2 to -14.1)	2720 (2550 to 2890)	8450 (8050 to 8840)	24700 (24000 to 25600)	79.1 (69.8 to 88.8)	-17.9 (-22.2 to -13.4)
Neoplasms	1090 (1010 to 1170)	2040 (1820 to 2180)	3270 (2560 to 3660)	133 (117 to 150)	-5.74 (-12.4 to 1.1)	624 (573 to 670)	1130 (936 to 1240)	2170 (1580 to 2470)	110 (94.5 to 124)	-3.7 (-10.8 to 2.68)
Lip and oral cavity cancer	18 (16.2 to 19.9)	26.4 (23.3 to 29.1)	43.8 (34.2 to 49.5)	146 (113 to 183)	-0.46 (-14 to 14.3)	8.64 (7.75 to 9.56)	16.6 (13.6 to 18.5)	35.8 (26.2 to 41.5)	164 (140 to 190)	21.2 (9.96 to 32.7)
Nasopharynx cancer	5.68 (5.04 to 6.33)	5.77 (5.12 to 6.37)	5.5 (4.26 to 6.3)	105 (79.4 to 135)	-17 (-27.5 to -5.15)	2.15 (1.9 to 2.43)	2.4 (1.99 to 2.73)	3.1 (2.31 to 3.6)	48.2 (26.8 to 80.5)	-32 (-41.9 to -17.3)
Other pharynx cancer	11.8 (10.4 to 13.3)	12.8 (11.2 to 14.4)	10.9 (8.62 to 12.5)	204 (157 to 253)	23 (3.95 to 42.5)	3.18 (2.7 to 3.7)	3.93 (3.18 to 4.62)	4.3 (3.22 to 5.05)	171 (125 to 221)	24.1 (3.35 to 47)
Esophageal cancer	71 (59.5 to 81.3)	97.9 (82.5 to 108)	75.7 (59.4 to 84.3)	129 (96.2 to 169)	-7.62 (-20.7 to 8.77)	23.7 (19.1 to 27.4)	38.3 (29.4 to 43.9)	47.6 (35.6 to 55.2)	51.7 (31.7 to 78.1)	-30.5 (-39.6 to -18.3)
Stomach cancer	121 (108 to 135)	204 (181 to 221)	247 (189 to 277)	68.9 (50.4 to 89.4)	-31.7 (-39.2 to -23.4)	54 (48.3 to 60)	107 (87.5 to 120)	202 (146 to 233)	34.5 (21.7 to 48.9)	-38.4 (-44.2 to -31.7)
Colon and rectum cancer	115 (107 to 123)	246 (219 to 264)	452 (352 to 506)	166 (146 to 184)	7.54 (-0.392 to 14.7)	74.7 (68.3 to 80.4)	183 (150 to 201)	441 (316 to 506)	108 (92.4 to 121)	-4.66 (-11.8 to 1.51)
Liver cancer	54 (49.4 to 58.8)	76.9 (67.9 to 83.4)	78.7 (60.4 to 89)	123 (95.3 to 155)	-10 (-21 to 3.15)	24.7 (22.2 to 27.1)	41.4 (33.5 to 46.1)	58.9 (42.6 to 68.5)	84.7 (55.8 to 112)	-15.3 (-28.6 to -2.7)
Liver cancer due to hepatitis B	18.7 (14.8 to 23.1)	20 (15.1 to 24.8)	11.9 (7.02 to 17.8)	68.1 (38.9 to 107)	-32 (-43.8 to -16.4)	4.63 (3.36 to 6.21)	5.12 (3.45 to 6.91)	4.42 (2.34 to 7.44)	39.1 (9.72 to 68.8)	-36.3 (-49.7 to -22.6)
Liver cancer due to hepatitis C	15.4 (12.3 to 18.7)	32.4 (27.1 to 38.1)	47.8 (34.1 to 58.7)	158 (133 to 188)	4.23 (-5.78 to 16.5)	11.9 (9.66 to 13.9)	25 (19.4 to 29.1)	43.1 (29.1 to 52.3)	91.7 (62.8 to 119)	-12.1 (-25.4 to 0.199)
Liver cancer due to alcohol use	14.7 (11.3 to 18.4)	16.6 (12.5 to 20.7)	11.7 (6.86 to 17.1)	169 (142 to 199)	8.57 (-2.22 to 20.7)	3.54 (2.53 to 4.89)	3.92 (2.76 to 5.26)	3.16 (1.68 to 4.88)	101 (71.4 to 127)	-8.07 (-21.4 to 4.2)
Liver cancer due to NASH	3.49 (2.6 to 4.74)	5.7 (4.08 to 7.8)	5.4 (3.03 to 8.65)	193 (155 to 236)	18.5 (3.03 to 36)	3.12 (2.34 to 4.16)	5.21 (3.69 to 6.97)	5.83 (3.29 to 9.22)	139 (101 to 177)	9.78 (-7.67 to 27.2)
Liver cancer due to other causes	1.7 (1.22 to 2.27)	2.34 (1.68 to 3.14)	1.94 (1.14 to 3.08)	100 (69.3 to 137)	-19.1 (-31.5 to -4)	1.55 (1.12 to 2.03)	2.15 (1.52 to 2.9)	2.39 (1.41 to 3.75)	65.3 (36.2 to 95.1)	-24.2 (-37.6 to -10.5)
Gallbladder and biliary tract cancer	15.1 (12.4 to 16.7)	32 (24.2 to 35.4)	54.1 (35.5 to 63.8)	150 (96.9 to 179)	1.13 (-20.4 to 12.9)	16.4 (13.8 to 19.3)	32.5 (24.5 to 37.1)	72.5 (45.1 to 87.6)	79.8 (49.3 to 104)	-17.6 (-31.6 to -6.27)
Pancreatic cancer	55.1 (51.4 to 58.9)	91.3 (81.1 to 98.1)	125 (97 to 142)	226 (202 to 252)	31.9 (22.1 to 42.2)	44.7 (40.5 to 48.1)	83.5 (68 to 92.6)	148 (105 to 172)	188 (166 to 209)	32.2 (22 to 41.6)
Larynx cancer	17.2 (16 to 18.7)	22.4 (20 to 24.3)	25.1 (19.7 to 28.4)	81 (66.7 to 96.5)	-26.8 (-32.6 to -20.5)	2.61 (2.38 to 2.88)	3.34 (2.8 to 3.73)	3.87 (2.82 to 4.46)	78 (63.4 to 97.5)	-18.4 (-25.1 to -9.48)
Tracheal, bronchus, and lung cancer	296 (269 to 324)	434 (385 to 469)	428 (331 to 482)	140 (115 to 164)	-3.14 (-13.2 to 6.62)	115 (104 to 126)	176 (144 to 196)	259 (191 to 300)	199 (173 to 225)	36.9 (25.1 to 48.9)
Malignant skin melanoma	5.74 (3.24 to 6.78)	12.7 (6.91 to 14.8)	29.7 (15 to 36.3)	190 (56.7 to 233)	17.4 (-36.7 to 34.5)	3.47 (2.27 to 4.03)	8.07 (4.98 to 9.33)	20.3 (11.5 to 24.7)	123 (48.1 to 147)	2.28 (-32.1 to 13.3)
Non-melanoma skin cancer	6.11 (5.58 to 6.57)	19.1 (16.7 to 20.5)	75.1 (58 to 84.4)	197 (175 to 225)	20.2 (11.2 to 31.3)	2.74 (2.41 to 3.06)	11 (8.77 to 12.2)	54.9 (39.1 to 63.2)	170 (141 to 194)	23.8 (10.6 to 35)
Non-melanoma skin cancer (squamous-cell carcinoma)	6.11 (5.58 to 6.57)	19.1 (16.7 to 20.5)	75.1 (58 to 84.4)	197 (175 to 225)	20.2 (11.2 to 31.3)	2.74 (2.41 to 3.06)	11 (8.77 to 12.2)	54.9 (39.1 to 63.2)	170 (141 to 194)	23.8 (10.6 to 35)

Supplementary Table 4. Cause-specific mortality rate for 70-79 years, 80-94 years, and 95+ years in 2019, and percentage change from 1990 to 2019 in death number and rate in 70+ years, global, for all GBD causes

Causes	Males					Females				
	Deaths per 100,000, 2019			Percentage change in 70+, 1990-2019		Deaths per 100,000, 2019			Percentage change in 70+, 1990-2019	
	70 to 79	80 to 94	95 plus	Number	Rate	70 to 79	80 to 94	95 plus	Number	Rate
Non-melanoma skin cancer (basal-cell carcinoma)										
Breast cancer	2.04 (1.79 to 2.29)	3.74 (3.24 to 4.21)	6.99 (5.48 to 8.19)	108 (78 to 140)	-15.9 (-28.1 to -2.86)	73.9 (68.2 to 79)	127 (105 to 139)	292 (218 to 335)	109 (94.4 to 121)	-4.32 (-10.9 to 1.46)
Cervical cancer						26 (22.6 to 29.3)	31.7 (26.1 to 35.7)	40.5 (30.1 to 46.9)	74.9 (52.6 to 93.8)	-19.8 (-30 to -11.2)
Uterine cancer						14.2 (13 to 16)	20.5 (16.9 to 23.3)	32.8 (24.5 to 37.7)	84.5 (73.4 to 98.9)	-15.4 (-20.5 to -8.8)
Ovarian cancer						26.3 (23.3 to 28.8)	35.1 (28.9 to 39)	46.5 (33.7 to 54.2)	121 (95.7 to 140)	1.44 (-10.3 to 10.2)
Prostate cancer	112 (98.1 to 138)	362 (301 to 447)	896 (626 to 1070)	121 (107 to 145)	-10.5 (-16.5 to -0.925)					
Testicular cancer	0.58 (0.529 to 0.635)	1.38 (1.19 to 1.5)	4.09 (3.16 to 4.71)	127 (106 to 152)	-8.33 (-16.5 to 1.72)					
Kidney cancer	19.4 (18.2 to 20.6)	38.5 (33.6 to 41.3)	60.7 (47.5 to 68.7)	225 (203 to 252)	31.6 (22.5 to 42.2)	9.11 (8.4 to 9.71)	18.9 (15.3 to 20.9)	34.2 (24.7 to 40)	150 (130 to 170)	14.4 (5.45 to 23.6)
Bladder cancer	35.4 (33.1 to 37.6)	105 (92.6 to 113)	246 (193 to 278)	120 (105 to 139)	-11.1 (-17.2 to -3.46)	9.43 (8.63 to 10.1)	28.5 (23.3 to 31.6)	75.4 (55.4 to 87.3)	95.3 (77.7 to 112)	-10.5 (-18.5 to -2.71)
Brain and central nervous system cancer	16.7 (11.4 to 19)	19.7 (12.5 to 22.9)	16.7 (9.68 to 20.3)	205 (82.6 to 266)	23.4 (-26.2 to 48.1)	12.5 (7.98 to 14.4)	14.6 (8.37 to 17.7)	16.4 (9.16 to 20.7)	174 (45.8 to 249)	25.5 (-33.2 to 60)
Thyroid cancer	3.27 (2.94 to 3.55)	6.43 (5.37 to 7.08)	8.31 (5.85 to 9.66)	217 (162 to 257)	28.3 (5.72 to 44.5)	4.11 (3.66 to 4.47)	7.27 (5.87 to 8.09)	12.8 (8.85 to 14.9)	102 (78.1 to 119)	-7.41 (-18.4 to 0.363)
Mesothelioma	4.73 (4.47 to 4.98)	9.03 (7.95 to 9.64)	10.6 (8.44 to 12.1)	158 (134 to 180)	4.45 (-5.46 to 13)	1.27 (0.9 to 1.42)	1.99 (1.41 to 2.3)	2.58 (1.62 to 3.21)	83.1 (33.7 to 124)	-16 (-38.7 to 2.79)
Hodgkin lymphoma	1.67 (1.38 to 1.99)	2.17 (1.73 to 2.44)	1.68 (1.01 to 1.98)	35.9 (20.4 to 57.7)	-45.1 (-51.3 to -36.3)	1.04 (0.822 to 1.29)	1.35 (0.965 to 1.61)	1.05 (0.548 to 1.39)	22 (12 to 36.2)	-44.1 (-48.6 to -37.6)
Non-Hodgkin lymphoma	24.1 (22.6 to 25.5)	47.4 (41.8 to 50.7)	75.3 (58.9 to 84.5)	186 (167 to 205)	15.6 (8.09 to 23.2)	16.1 (14.8 to 17.2)	32.9 (26.7 to 36.3)	57.4 (41.1 to 66.1)	139 (122 to 154)	9.47 (1.81 to 16.5)
Multiple myeloma	12.5 (10.3 to 13.7)	24.1 (17.7 to 26.3)	38.8 (28.1 to 44.6)	180 (132 to 201)	13.1 (-6.05 to 21.7)	9.62 (8.4 to 10.9)	16.4 (12.1 to 18.3)	20.9 (12.8 to 25)	128 (88.4 to 144)	4.5 (-13.6 to 11.7)
Leukemia	27.4 (24.2 to 29.9)	56.7 (49.8 to 61.6)	107 (81.5 to 123)	134 (117 to 153)	-5.2 (-12.2 to 2.36)	17.2 (15.4 to 18.4)	32.8 (27.1 to 36.3)	64.5 (47.2 to 74.6)	105 (85.1 to 120)	-6.05 (-15.1 to 0.836)
Acute lymphoid leukemia	1.68 (1.27 to 1.91)	2.38 (1.86 to 2.69)	2.85 (1.94 to 3.41)	166 (124 to 198)	7.6 (-9.52 to 20.6)	1.13 (0.821 to 1.32)	1.5 (1.11 to 1.75)	1.65 (1.1 to 1.99)	132 (86.7 to 163)	6.17 (-14.4 to 20.4)
Chronic lymphoid leukemia	4.46 (4.04 to 5.34)	12.6 (10.7 to 15)	35.5 (26.4 to 44.4)	138 (114 to 181)	-3.74 (-13.4 to 13.7)	3.6 (3.21 to 4.14)	8.54 (7.1 to 9.68)	20.6 (15 to 24.6)	127 (107 to 150)	3.92 (-4.9 to 14.8)
Acute myeloid leukemia	8.79 (6.9 to 9.66)	15.4 (11.2 to 17.3)	16.9 (9.46 to 22.2)	230 (152 to 271)	33.3 (1.82 to 50.1)	5.33 (4.43 to 5.82)	8.38 (5.95 to 9.55)	10.6 (5.8 to 13.7)	170 (109 to 199)	23.6 (-4.17 to 37)
Chronic myeloid leukemia	2.36 (2.05 to 2.76)	4.72 (4.04 to 5.55)	7.09 (5.26 to 8.71)	48 (30.6 to 73.4)	-40.2 (-47.2 to -29.9)	1.1 (0.981 to 1.29)	2.17 (1.78 to 2.51)	4.51 (3.13 to 5.41)	20.4 (9.01 to 37.1)	-44.8 (-50 to -37.2)
Other leukemia	10.1 (8.6 to 11.6)	21.6 (18.5 to 24.7)	44.4 (33.1 to 53.3)	109 (84.2 to 144)	-15.4 (-25.5 to -1.24)	5.99 (5.12 to 6.82)	12.2 (9.76 to 13.9)	27.1 (20.1 to 32.1)	80 (62.5 to 102)	-17.5 (-25.5 to -7.22)
Other malignant neoplasms	31.6 (24.4 to 36.3)	55.5 (42 to 63.6)	83.1 (60.5 to 95.6)	143 (107 to 179)	-1.65 (-16.4 to 12.7)	23.6 (21 to 25.9)	43.5 (35.4 to 48.4)	77.2 (54.6 to 89.6)	121 (97 to 143)	1.25 (-9.71 to 11.6)
Other neoplasms	6.57 (4.7 to 9.57)	23.4 (15.1 to 33.7)	62.8 (32.8 to 91.8)	183 (104 to 246)	14.3 (-17.3 to 40)	3.6 (3.21 to 3.89)	13.8 (10.5 to 15.4)	41.8 (27.7 to 49.4)	165 (110 to 198)	21.5 (-3.77 to 36.8)
Myelodysplastic, myeloproliferative, and other hematopoietic neoplasms	6.57 (4.7 to 9.57)	23.4 (15.1 to 33.7)	62.8 (32.8 to 91.8)	183 (104 to 246)	14.3 (-17.3 to 40)	3.6 (3.21 to 3.89)	13.8 (10.5 to 15.4)	41.8 (27.7 to 49.4)	165 (110 to 198)	21.5 (-3.77 to 36.8)
Benign and in situ intestinal neoplasms										
Benign and in situ cervical and uterine neoplasms										
Other benign and in situ neoplasms										
Cardiovascular diseases	1740 (1610 to 1870)	5070 (4540 to 5420)	12300 (9780 to 13500)	85 (72.4 to 98)	-25.2 (-30.3 to -19.9)	1280 (1160 to 1380)	4510 (3780 to 4960)	13500 (10100 to 15300)	60.1 (47.6 to 72)	-26.6 (-32.3 to -21.1)
Rheumatic heart disease	18.8 (15.9 to 23.3)	45.3 (38.6 to 53.3)	80.9 (64.8 to 94.4)	21.7 (-7.8 to 54.3)	-50.8 (-62.7 to -37.6)	20.8 (17.2 to 24.4)	48.2 (39 to 55.6)	116 (86.5 to 136)	11.4 (-14.1 to 40.5)	-48.9 (-60.6 to -35.6)
Ischemic heart disease	857 (795 to 920)	2560 (2280 to 2740)	6730 (5340 to 7470)	85.3 (72.1 to 96.7)	-25.1 (-30.4 to -20.5)	586 (531 to 635)	2150 (1800 to 2360)	6710 (5090 to 7600)	62 (50.9 to 72.3)	-25.7 (-30.8 to -21)
Stroke	668 (604 to 732)	1750 (1560 to 1880)	3060 (2430 to 3410)	78.4 (59.8 to 97.1)	-27.9 (-35.4 to -20.3)	500 (453 to 550)	1550 (1300 to 1710)	3810 (2830 to 4340)	46 (31.7 to 63.5)	-33.1 (-39.6 to -25)
Ischemic stroke	345 (311 to 381)	1140 (1020 to 1230)	2320 (1820 to 2600)	90.7 (67.8 to 108)	-22.9 (-32.2 to -16)	261 (236 to 286)	1070 (893 to 1180)	2930 (2180 to 3350)	52.4 (38.5 to 68.9)	-30.2 (-36.5 to -22.5)
Intracerebral hemorrhage	297 (261 to 328)	552 (490 to 605)	624 (499 to 705)	72.8 (51.4 to 95.8)	-30.1 (-38.8 to -20.9)	215 (190 to 238)	431 (360 to 482)	751 (571 to 860)	44.7 (24.5 to 67.1)	-33.7 (-42.9 to -23.4)
Subarachnoid hemorrhage	26.2 (21.7 to 31.2)	57 (44.7 to 65.5)	123 (75.9 to 150)	4.21 (-21.8 to 149)	-57.9 (-68.4 to 0.575)	24.3 (21.4 to 27.5)	52.7 (42.8 to 58.7)	123 (84.3 to 143)	-8.66 (-23.8 to 11.8)	-58.1 (-65.1 to -48.7)
Hypertensive heart disease	92.6 (65.6 to 105)	315 (211 to 352)	772 (500 to 880)	111 (70.7 to 145)	-14.8 (-31 to -1.03)	92.9 (75.2 to 106)	356 (257 to 403)	1190 (734 to 1390)	100 (57.1 to 137)	-8.13 (-28 to 8.78)
Non-rheumatic valvular heart disease	10.1 (9.46 to 10.8)	55 (46.1 to 59.4)	273 (201 to 343)	158 (136 to 176)	4.47 (-4.47 to 11.7)	8.83 (7.99 to 9.75)	62.7 (48.8 to 71.3)	358 (249 to 423)	142 (115 to 162)	10.9 (-1.31 to 20)
Non-rheumatic calcific aortic valve disease	7.87 (7.14 to 8.48)	46.7 (38.9 to 51.2)	245 (179 to 309)	176 (152 to 201)	11.5 (1.87 to 21.5)	6 (5.25 to 6.89)	51 (39.1 to 59.5)	309 (217 to 372)	175 (147 to 199)	26.1 (13 to 37.1)
Non-rheumatic degenerative mitral valve disease	2.03 (1.59 to 2.51)	7.52 (5.19 to 9.17)	27.6 (15.9 to 35.9)	100 (80.5 to 149)	-19.1 (-27 to 0.587)	2.58 (2.16 to 3.47)	10.8 (7.18 to 15.1)	48.1 (23.4 to 87.2)	65 (44.7 to 124)	-24.4 (-33.7 to 2.48)
Other non-rheumatic valve diseases	0.205 (0.138 to 0.271)	0.768 (0.551 to 1.06)	0.648 (0.396 to 0.853)	68.4 (16.8 to 174)	-31.9 (-52.8 to 10.9)	0.251 (0.182 to 0.302)	0.877 (0.617 to 1.07)	1.76 (1.09 to 2.35)	53.5 (17.6 to 107)	-29.6 (-46.1 to -4.93)
Cardiomyopathy and myocarditis	22.7 (18.3 to 26.3)	74.1 (54.5 to 83.1)	283 (185 to 342)	69.1 (54.5 to 116)	-31.6 (-37.5 to -12.6)	13.3 (11.2 to 15.2)	69 (51.1 to 77.9)	302 (206 to 356)	34.8 (18.6 to 75.1)	-38.2 (-45.6 to -19.7)
Myocarditis	1.79 (1.07 to 2.19)	7.61 (4.16 to 9.67)	21.5 (10.2 to 29.3)	248 (116 to 390)	40.8 (-12.5 to 98.1)	1.24 (0.776 to 1.53)	8.1 (4.48 to 10.4)	35.4 (16.4 to 49.5)	200 (79.4 to 345)	37.5 (-17.8 to 104)
Alcoholic cardiomyopathy	4.8 (3.69 to 5.76)	5.23 (3.7 to 6.76)	10.9 (6.92 to 17.1)	51.2 (18.7 to 75.2)	-38.9 (-52 to -29.2)	0.985 (0.742 to 1.24)	1.3 (0.814 to 1.82)	2.64 (1.65 to 3.6)	-53.2 (-64.4 to -18)	-78.6 (-83.7 to -62.4)
Other cardiomyopathy	16.1 (13.4 to 19.2)	61.3 (46 to 68.5)	251 (165 to 302)	62.5 (46.3 to 126)	-34.3 (-40.9 to -8.48)	11 (9.46 to 12.8)	59.6 (44 to 68)	264 (177 to 318)	34.4 (18.2 to 82.6)	-38.4 (-45.8 to -16.3)
Atrial fibrillation and flutter	18.4 (15.1 to 22.6)	127 (100 to 158)	577 (416 to 752)	206 (163 to 241)	23.7 (6.13 to 37.9)	19.4 (17.1 to 22)	143 (115 to 166)	583 (423 to 742)	168 (141 to 192)	23 (10.4 to 33.9)
Aortic aneurysm	21.6 (20.2 to 23)	56.4 (49.1 to 60.4)	119 (92.1 to 138)	85.8 (70 to 102)	-24.9 (-31.3 to -18.5)	9.81 (8.93 to 10.5)	32.2 (25.6 to 35.8)	90.2 (64.2 to 106)	119 (99.2 to 132)	0.298 (-8.68 to 6.24)
Peripheral artery disease	7.18 (3.45 to 14.6)	28.8 (12.6 to 63.5)	134 (49.8 to 317)	171 (124 to 215)	9.7 (-9.36 to 27.2)	3.78 (1.76 to 7.5)	24.5 (10.2 to 51.3)	129 (47 to 266)	150 (65.7 to 187)	14.7 (-24 to 31.8)
Endocarditis	4.16 (2.58 to 4.86)	13.3 (6.75 to 16.3)	57.1 (20.3 to 82.8)	285 (106 to 404)	55.7 (-16.5 to 104)	3.51 (2.39 to 3.98)	15.3 (8.23 to 19.1)	76.4 (28.9 to 104)	250 (87.3 to 353)	60.4 (-14.1 to 108)
Other cardiovascular and circulatory diseases	21.1 (18.9 to 24.2)	57.5 (50.1 to 64.6)	162 (126 to 193)	69.4 (46.4 to 86.6)	-31.5 (-40.8 to -24.6)	19.1 (17.1 to 21)	57.2 (46.9 to 63.6)	178 (130 to 206)	67.6 (45.3 to 82.9)	-23.2 (-33.4 to -16.1)
Chronic respiratory diseases	500 (452 to 552)	1420 (1250 to 1550)	2370 (1920 to 2620)	54.1 (35.6 to 76.8)	-37.7 (-45.2 to -28.5)	296 (247 to 334)	853 (682 to 971)	1740 (1260 to 2000)	44 (21.4 to 80.2)	-34 (-44.4 to -17.4)
Chronic obstructive pulmonary disease	434 (391 to 482)	1270 (1130 to 1390)	2110 (1680 to 2350)	55.5 (35.7 to 79.2)	-37.1 (-45.1 to -27.6)	243 (201 to 278)	739 (591 to 848)	1540 (1140 to 1790)	41.4 (18.8 to 82.5)	-35.2 (-45.5 to -16.3)
Pneumoconiosis	4.05 (3.59 to 4.6)	11.4 (10 to 12.9)	14.3 (11.3 to 16.1)	21.5 (2.86 to 56.4)	-50.9 (-58.4 to -36.8)	0.33 (0.271 to 0.39)	0.879 (0.679 to 1.03)	1.86 (1.24 to 2.25)	84.1 (34.5 to 125)	-15.6 (-38.3 to 3.16)
Silicosis	2.29 (1.91 to 2.91)	6.11 (5.26 to 8.01)	5.29 (4.04 to 6.45)	5.21 (-16.8 to 53.7)	-57.5 (-66.4 to -37.9)	0.0911 (0.0649 to 0.121)	0.248 (0.177 to 0.325)	0.441 (0.289 to 0.638)	62.3 (-2.47 to 117)	-25.6 (-55.3 to -0.493)
Asbestosis	0.673 (0.441 to 0.778)	2.44 (1.6 to 2.79)	4.58 (2.92 to 5.42)	270 (145 to 337)	49.7 (-0.918 to 76.6)	0.0727 (0.0463 to 0.093)	0.211 (0.129 to 0.273)	0.485 (0.257 to 0.67)	114 (61.5 to 169)	-1.75 (-26 to 23.5)
Coal workers pneumoconiosis	0.595 (0.385 to 0.755)	1.7 (1.32 to 2.09)	2.38 (1.77 to 2.94)	-21.9 (-37.1 to 11.8)	-68.4 (-74.6 to -54.8)	0.0361 (0.0202 to 0.0543)	0.096 (0.06 to 0.134)	0.285 (0.168 to 0.447)	43.8 (0.99 to 108)	-34.1 (-53.7 to -4.65)
Other pneumoconiosis	0.484 (0.355 to 0.672)	1.12 (0.893 to 1.45)	2.08 (1.49 to 2.55)	84.5 (39.4 to 178)	-25.4 (-43.6 to 12.3)	0.13 (0.0978 to 0.169)	0.324 (0.242 to 0.413)	0.645 (0.405 to 0.861)	103 (49.3 to 148)	-6.82 (-31.5 to 13.8)
Asthma	38.9 (30.9 to 49.7)	82.7 (67.5 to 101)	126 (96.8 to 161)	11 (-14.8 to 38.7)	-55.1 (-65.5 to -43.9)	37.6 (25.7 to 51.4)	78.6 (55 to 100)	118 (79.9 to 160)	33.3 (1.56 to 69.1)	-38.9 (-53.4 to -22.5)
Interstitial lung disease and pulmonary sarcoidosis	21.1 (12.7 to 27.4)	49.2 (27.8 to 65.2)	108 (48.8 to 161)	222 (107 to 391)	30.4 (-16.3 to 98.4)	13.8 (8.7 to 17)	30.9 (17.5 to 39.8)	68.1 (30.2 to 98.8)	229 (100 to 350)	51 (-8.11 to 106)
Other chronic respiratory diseases	1.98 (1.42 to 2.43)	5.72 (4.17 to 6.71)	18.1 (10.3 to 22.4)	81 (51.9 to 156)	-26.8 (-38.6 to 3.52)	1.16 (0.722 to 1.46)	3.5 (2.15 to 4.41)	9.68 (5.34 to 12.4)	96.8 (59.7 to 146)	-9.78 (-26.8 to 12.9)

Supplementary Table 4. Cause-specific mortality rate for 70-79 years, 80-94 years, and 95+ years in 2019, and percentage change from 1990 to 2019 in death number and rate in 70+ years, global, for all GBD causes

Causes	Males					Females				
	Deaths per 100,000, 2019			Percentage change in 70+, 1990-2019		Deaths per 100,000, 2019			Percentage change in 70+, 1990-2019	
	70 to 79	80 to 94	95 plus	Number	Rate	70 to 79	80 to 94	95 plus	Number	Rate
Digestive diseases	186 (175 to 200)	401 (356 to 432)	1000 (788 to 1120)	78.8 (63.5 to 95.2)	-27.7 (-33.9 to -21.1)	130 (119 to 140)	337 (279 to 369)	961 (706 to 1100)	72.8 (55.7 to 87.3)	-20.8 (-28.6 to -14.1)
Cirrhosis and other chronic liver diseases	102 (95 to 111)	153 (135 to 169)	284 (221 to 327)	87.8 (69.6 to 109)	-24.1 (-31.4 to -15.6)	62.7 (56.9 to 69.6)	101 (83.6 to 113)	185 (134 to 227)	72.6 (50.3 to 92.9)	-20.9 (-31.1 to -11.6)
Cirrhosis and other chronic liver diseases due to hepatitis B	22.1 (16 to 29.1)	33.3 (24.4 to 43.1)	51.8 (27.8 to 83)	43.6 (25 to 64.9)	-42 (-49.5 to -33.3)	11 (7.66 to 14.6)	17 (12 to 22.4)	24.1 (12.5 to 39.3)	24 (4.79 to 42.6)	-43.1 (-52 to -34.6)
Cirrhosis and other chronic liver diseases due to hepatitis C	28.5 (22.3 to 35.6)	43.7 (33.3 to 54.7)	88.9 (54.1 to 128)	107 (85.2 to 131)	-16.3 (-25.1 to -6.59)	15.5 (11.5 to 20.2)	24.8 (17.9 to 32.2)	53.3 (31.3 to 79)	87.3 (61.7 to 113)	-14.2 (-25.9 to -2.52)
Cirrhosis and other chronic liver diseases due to alcohol use	29 (22.4 to 36.2)	34.6 (25.9 to 44.6)	56.5 (32.3 to 92.5)	97.1 (79.8 to 118)	-20.3 (-27.3 to -11.8)	11.8 (8.34 to 15.9)	15 (10.8 to 20.7)	25.9 (13.8 to 45.2)	80.6 (61.3 to 101)	-17.2 (-26.1 to -7.93)
Cirrhosis and other chronic liver diseases due to NAFLD	9.63 (5.74 to 14.6)	17.2 (10.5 to 25.8)	37 (15.6 to 67.8)	125 (102 to 153)	-9.09 (-18.5 to 2.28)	8.98 (5.39 to 13.4)	16.3 (9.97 to 23.8)	30.3 (13.5 to 52.8)	104 (77.2 to 130)	-6.49 (-18.8 to 5.27)
Cirrhosis and other chronic liver diseases due to other causes	13.1 (7.68 to 20.6)	23.8 (14.3 to 36.2)	49.3 (21.3 to 92.1)	104 (81.7 to 130)	-17.5 (-26.5 to -6.82)	15.5 (9.34 to 22.6)	27.5 (17.4 to 39)	51.3 (24.4 to 83.9)	84.3 (59.9 to 108)	-15.5 (-26.7 to -4.46)
Upper digestive system diseases	24.5 (22.3 to 27.3)	59.9 (53.4 to 66.5)	125 (98.2 to 143)	10.1 (-2.09 to 25)	-55.5 (-60.4 to -49.5)	17.5 (15.6 to 19.3)	51.5 (42.8 to 57.6)	125 (93.6 to 148)	20.2 (7.57 to 33.7)	-44.9 (-50.7 to -38.7)
Peptic ulcer disease	21.1 (19.1 to 23.7)	50.9 (45.2 to 57.2)	110 (86.2 to 127)	4.58 (-8.49 to 22)	-57.7 (-63 to -50.7)	15.2 (13.5 to 16.8)	43.8 (36.5 to 49.1)	105 (77.8 to 125)	14.6 (1.78 to 29.7)	-47.5 (-53.3 to -40.5)
Gastritis and duodenitis	3.44 (2.24 to 4.04)	8.95 (6.66 to 10.2)	14.4 (10.9 to 17.2)	59.7 (4.28 to 138)	-35.4 (-57.8 to -3.95)	2.32 (1.92 to 2.68)	7.69 (6.25 to 8.85)	20.2 (15.2 to 24.4)	69.7 (29 to 121)	-22.2 (-40.9 to 1.15)
Gastroesophageal reflux disease										
Appendicitis	1.83 (1.36 to 2.23)	4.01 (3.11 to 4.98)	8.28 (5.99 to 11.1)	25.3 (3.92 to 65)	-49.3 (-58 to -33.3)	1.46 (1.13 to 1.68)	3.06 (2.46 to 3.53)	5.92 (4.44 to 7.05)	28.9 (-4.58 to 63)	-40.9 (-56.3 to -25.3)
Paralytic ileus and intestinal obstruction	16.3 (13.3 to 19)	55.2 (44.8 to 64.6)	180 (120 to 213)	137 (97.2 to 176)	-4.11 (-20.3 to 11.7)	12.5 (10.3 to 14.3)	46.7 (36.6 to 53.4)	172 (109 to 211)	120 (85.5 to 155)	0.743 (-15 to 17)
Inguinal, femoral, and abdominal hernia	4.69 (4.05 to 5.46)	15.3 (13.2 to 17.8)	47.8 (37.2 to 57.6)	63.4 (25.1 to 95.5)	-33.9 (-49.4 to -21)	2.42 (1.82 to 2.97)	9.49 (7.35 to 11.5)	30.5 (20.7 to 38.9)	59.3 (35 to 98.8)	-27 (-38.1 to -8.85)
Inflammatory bowel disease	2.92 (2.31 to 3.33)	10.2 (7.94 to 11.4)	32.6 (21.5 to 39.7)	130 (88.6 to 180)	-6.88 (-23.7 to 13.1)	2.59 (2.26 to 2.81)	10.9 (8.41 to 12.3)	45.9 (31.8 to 56.9)	121 (77.8 to 160)	1.54 (-18.5 to 19)
Vascular intestinal disorders	9.16 (8.48 to 9.82)	26.9 (23.4 to 29.7)	70.2 (55.5 to 83.5)	121 (100 to 140)	-10.6 (-19.1 to -2.78)	9.59 (8.65 to 10.7)	34.7 (28.8 to 39.3)	89.2 (65.7 to 110)	109 (91.3 to 129)	-4.23 (-12.3 to 5.06)
Gallbladder and biliary diseases	9.54 (8.08 to 11.3)	35.9 (29.6 to 41.1)	125 (89.6 to 151)	108 (87.4 to 147)	-15.9 (-24.2 to -0.156)	8.95 (7.86 to 10.7)	37.1 (29.1 to 43.2)	155 (103 to 192)	73.5 (54.1 to 104)	-20.5 (-29.3 to -6.59)
Pancreatitis	7.49 (6.44 to 8.6)	14.7 (12.8 to 16.5)	28.3 (22.1 to 33.1)	123 (90.6 to 155)	-10 (-22.9 to 3.19)	5.51 (4.53 to 6.46)	13.5 (11 to 15.7)	31.6 (22.9 to 39.1)	92.8 (61.9 to 124)	-11.6 (-25.8 to 2.69)
Other digestive diseases	7.44 (5.66 to 8.89)	26.2 (19.5 to 31.8)	103 (69.4 to 133)	127 (76 to 168)	-8.41 (-28.8 to 8.27)	6.82 (5.33 to 8.22)	29.5 (21.7 to 36.7)	121 (79.7 to 161)	118 (67.4 to 158)	-0.0414 (-23.2 to 18.3)
Neurological disorders	135 (79.3 to 269)	848 (373 to 1900)	3620 (1140 to 8930)	209 (189 to 231)	25.1 (16.8 to 33.7)	123 (59.1 to 277)	934 (333 to 2150)	4200 (1220 to 10300)	178 (161 to 198)	27.5 (19.7 to 36.7)
Alzheimer's disease and other dementias	72.2 (16.3 to 208)	635 (151 to 1710)	3330 (816 to 8710)	224 (206 to 250)	30.8 (23.7 to 41.3)	84.7 (19.4 to 240)	818 (208 to 2060)	4030 (1030 to 10200)	186 (168 to 213)	31 (22.6 to 43.7)
Parkinson's disease	49.9 (46 to 54.1)	190 (168 to 206)	255 (197 to 300)	180 (156 to 204)	13.1 (3.31 to 22.7)	27.9 (25 to 30)	98.6 (81 to 109)	134 (99.6 to 152)	136 (116 to 154)	8.16 (-1.1 to 16.6)
Idiopathic epilepsy	3.6 (3.23 to 4.04)	8.05 (6.92 to 8.93)	15.4 (12.2 to 17.7)	161 (137 to 205)	5.55 (-4.12 to 23.2)	3.01 (2.13 to 3.55)	6.68 (4.04 to 7.82)	13.6 (6.96 to 16.6)	182 (96.2 to 253)	29.1 (-10 to 61.9)
Multiple sclerosis	0.956 (0.664 to 1.19)	1.1 (0.777 to 1.36)	1.1 (0.571 to 1.41)	136 (44.6 to 173)	-4.63 (-41.5 to 10.5)	1.3 (0.802 to 1.62)	1.4 (0.874 to 1.71)	1.32 (0.739 to 1.68)	123 (24.4 to 166)	2.05 (-43 to 21.8)
Motor neuron disease	4.32 (4.01 to 4.57)	5.19 (4.48 to 5.63)	4.83 (3.79 to 5.52)	236 (212 to 258)	36 (25.9 to 44.6)	3.22 (2.92 to 3.48)	3.4 (2.72 to 3.8)	2.66 (1.92 to 3.08)	180 (157 to 201)	28.5 (17.8 to 38)
Headache disorders										
Migraine										
Tension-type headache										
Other neurological disorders	3.92 (3.68 to 4.18)	8.34 (7.32 to 8.98)	17.3 (13.4 to 20.7)	186 (171 to 202)	15.6 (9.51 to 22.2)	2.99 (2.76 to 3.17)	5.86 (4.75 to 6.45)	12.8 (9.47 to 14.6)	144 (129 to 160)	11.9 (4.76 to 19.1)
Mental disorders										
Schizophrenia										
Depressive disorders										
Major depressive disorder										
Dysthymia										
Bipolar disorder										
Anxiety disorders										
Eating disorders										
Anorexia nervosa										
Bulimia nervosa										
Autism spectrum disorders										
Attention-deficit/hyperactivity disorder										
Conduct disorder										
Idiopathic developmental intellectual disability										
Other mental disorders										
Substance use disorders	9.65 (8.41 to 10.3)	9.69 (8.47 to 10.4)	16.2 (12.8 to 18.2)	147 (125 to 167)	-0.234 (-9.08 to 7.82)	2.76 (2.56 to 2.93)	4.1 (3.4 to 4.51)	9.44 (6.94 to 10.9)	85.2 (70 to 101)	-15.1 (-22.1 to -7.9)
Alcohol use disorders	7.58 (6.33 to 8.19)	6.32 (5.24 to 6.86)	8.15 (6.03 to 9.28)	141 (113 to 166)	-2.61 (-13.8 to 7.43)	1.43 (1.31 to 1.53)	1.65 (1.4 to 1.83)	2.96 (2.18 to 3.46)	71.1 (57.1 to 82.6)	-21.6 (-28 to -16.3)
Drug use disorders	2.07 (1.91 to 2.22)	3.37 (2.99 to 3.66)	8.03 (6.38 to 9.16)	166 (133 to 200)	7.43 (-5.67 to 21.5)	1.33 (1.21 to 1.44)	2.45 (2 to 2.74)	6.47 (4.69 to 7.56)	99.4 (78.6 to 125)	-8.6 (-18.1 to 2.95)
Opioid use disorders	1.41 (1.29 to 1.52)	2.17 (1.92 to 2.37)	4.85 (3.94 to 5.56)	123 (91.2 to 152)	-9.88 (-22.7 to 1.88)	0.846 (0.774 to 0.922)	1.69 (1.37 to 1.9)	4.78 (3.46 to 5.68)	84.1 (62.6 to 108)	-15.6 (-25.5 to -4.78)
Cocaine use disorders	0.156 (0.131 to 0.174)	0.048 (0.0411 to 0.0538)	0.159 (0.121 to 0.183)	393 (203 to 491)	99.5 (22.3 to 139)	0.0253 (0.0228 to 0.0276)	0.0136 (0.0113 to 0.0152)	0.0292 (0.0215 to 0.0345)	143 (107 to 185)	11.6 (-5 to 30.8)
Amphetamine use disorders	0.0375 (0.0266 to 0.0438)	0.161 (0.13 to 0.204)	0.67 (0.46 to 0.93)	688 (521 to 865)	218 (151 to 290)	0.0385 (0.0315 to 0.047)	0.0854 (0.0685 to 0.102)	0.0731 (0.055 to 0.0882)	-26.3 (-48.1 to 8.01)	-66.2 (-76.2 to -50.5)
Cannabis use disorders										
Other drug use disorders	0.471 (0.429 to 0.518)	0.986 (0.878 to 1.08)	2.34 (1.81 to 2.85)	297 (218 to 395)	60.5 (28.5 to 100)	0.416 (0.376 to 0.459)	0.662 (0.543 to 0.738)	1.59 (1.16 to 1.87)	214 (149 to 298)	44.1 (14 to 82.4)
Diabetes and kidney diseases	262 (245 to 281)	640 (566 to 686)	1790 (1410 to 1980)	199 (174 to 222)	20.8 (10.6 to 30.3)	219 (201 to 237)	523 (434 to 576)	1450 (1060 to 1650)	171 (149 to 189)	24 (14.3 to 32.4)
Diabetes mellitus	141 (132 to 151)	290 (257 to 311)	564 (451 to 625)	199 (178 to 221)	20.7 (12.5 to 29.6)	127 (116 to 138)	265 (222 to 292)	525 (390 to 597)	147 (127 to 166)	13.3 (3.87 to 22.1)
Diabetes mellitus type 1	3.62 (3.03 to 5.12)	3.94 (3.22 to 5.25)	10.2 (6.97 to 14.4)	111 (64 to 168)	-14.7 (-33.7 to 8.48)	3.58 (2.76 to 4.92)	4.24 (3.25 to 5.68)	10.6 (6.95 to 14.6)	56 (36.2 to 102)	-28.5 (-37.6 to -7.24)
Diabetes mellitus type 2	138 (129 to 147)	286 (254 to 306)	554 (441 to 614)	201 (181 to 223)	21.7 (13.5 to 30.7)	124 (113 to 134)	261 (218 to 288)	515 (382 to 586)	150 (129 to 170)	14.8 (4.89 to 23.9)
Chronic kidney disease	120 (112 to 130)	349 (307 to 377)	1220 (956 to 1370)	202 (168 to 229)	21.9 (8.28 to 33)	90.9 (82.7 to 98.3)	257 (210 to 283)	918 (668 to 1050)	206 (172 to 229)	40.1 (24.7 to 50.6)
Chronic kidney disease due to diabetes mellitus type 1	3.57 (1.62 to 6.72)	2.51 (1.08 to 5.27)	0.425 (0.12 to 1.11)	177 (143 to 210)	11.8 (-1.83 to 25.4)	2.83 (1.32 to 5.38)	1.99 (0.868 to 4.23)	0.35 (0.101 to 0.9)	165 (140 to 195)	21.5 (9.81 to 35.2)

Supplementary Table 4. Cause-specific mortality rate for 70-79 years, 80-94 years, and 95+ years in 2019, and percentage change from 1990 to 2019 in death number and rate in 70+ years, global, for all GBD causes

	Males					Females				
	Deaths per 100,000, 2019			Percentage change in 70+, 1990-2019		Deaths per 100,000, 2019			Percentage change in 70+, 1990-2019	
Causes	70 to 79	80 to 94	95 plus	Number	Rate	70 to 79	80 to 94	95 plus	Number	Rate
Chronic kidney disease due to diabetes mellitus type 2	39.3 (27.6 to 50.5)	94.4 (65.4 to 129)	277 (148 to 428)	220 (178 to 260)	29.4 (12.2 to 45.4)	31.1 (21.9 to 39.8)	76.2 (53 to 101)	231 (125 to 349)	223 (185 to 260)	48.2 (30.9 to 64.9)
Chronic kidney disease due to hypertension	41 (32.3 to 51.4)	144 (116 to 174)	582 (419 to 768)	204 (169 to 233)	22.7 (8.93 to 34.5)	30.1 (23.6 to 38.5)	102 (77.8 to 125)	418 (279 to 558)	208 (174 to 236)	41.2 (25.6 to 54.2)
Chronic kidney disease due to glomerulonephritis	13.6 (7.69 to 20.8)	33.7 (18.7 to 53.8)	87.2 (38.6 to 174)	176 (144 to 208)	11.7 (-1.46 to 24.6)	9 (5.16 to 14.1)	20.5 (11.3 to 32.8)	54.3 (23.5 to 112)	178 (150 to 210)	27.7 (14.4 to 42)
Chronic kidney disease due to other and unspecified causes	22.9 (13.9 to 35.3)	74.3 (46.6 to 106)	275 (142 to 426)	189 (155 to 222)	16.9 (3.1 to 30.1)	17.8 (11 to 26.4)	56 (36.4 to 78.4)	215 (113 to 325)	193 (157 to 226)	34.2 (17.9 to 49.4)
Acute glomerulonephritis	0.88 (0.454 to 1.09)	2.07 (1.12 to 2.49)	2.2 (1.64 to 2.66)	33.9 (-41.6 to 78.9)	-45.9 (-76.4 to -27.7)	0.644 (0.469 to 0.802)	1.42 (1.08 to 1.74)	2.38 (1.75 to 2.9)	29.4 (-8.17 to 64.7)	-40.7 (-57.9 to -24.5)
Skin and subcutaneous diseases	6.97 (4.48 to 10.1)	22.5 (14.4 to 34.5)	99.9 (52.8 to 163)	153 (103 to 207)	2.16 (-17.8 to 24.2)	6.65 (5.06 to 8.47)	25.3 (17.8 to 34.7)	112 (66.4 to 162)	162 (132 to 194)	19.9 (6.27 to 34.6)
Dermatitis										
Atopic dermatitis										
Contact dermatitis										
Seborrhoeic dermatitis										
Psoriasis										
Bacterial skin diseases	5.13 (3.19 to 7.33)	14 (7.73 to 22)	60 (26.3 to 102)	182 (129 to 268)	14 (-7.42 to 48.9)	4.74 (3.66 to 6.2)	14.4 (9.94 to 20.9)	58.8 (33.2 to 89)	232 (180 to 291)	52.4 (28.2 to 79.3)
Cellulitis	1.23 (0.544 to 2.01)	3.53 (1.38 to 6.53)	16.4 (5.16 to 30.5)	222 (94.7 to 356)	30 (-21.3 to 84.5)	1.2 (0.664 to 1.71)	3.72 (2.02 to 5.84)	16.2 (7.31 to 27.3)	242 (166 to 295)	56.7 (21.9 to 81)
Pyoderma	3.91 (2.25 to 5.38)	10.5 (5.76 to 15.8)	43.6 (19.2 to 72.2)	171 (120 to 269)	9.58 (-11.2 to 49.1)	3.54 (2.9 to 4.62)	10.7 (7.59 to 15)	42.6 (24.8 to 61.1)	229 (180 to 299)	50.9 (28.1 to 82.8)
Scabies										
Fungal skin diseases										
Viral skin diseases										
Acne vulgaris										
Alopecia areata										
Pruritus										
Urticaria										
Decubitus ulcer	1.63 (0.949 to 2.68)	7.61 (4.56 to 12.5)	36.3 (17.2 to 65.2)	111 (40.2 to 204)	-14.9 (-43.3 to 23)	1.68 (1.08 to 2.05)	9.77 (6.39 to 13)	49.8 (28.2 to 65.8)	95.5 (59.3 to 131)	-10.4 (-27 to 5.81)
Other skin and subcutaneous diseases	0.208 (0.135 to 0.323)	0.918 (0.556 to 1.53)	3.67 (1.48 to 7.15)	96.5 (23.7 to 148)	-20.6 (-50 to 0.429)	0.233 (0.18 to 0.378)	1.09 (0.731 to 1.89)	3.85 (1.8 to 6.46)	105 (46 to 146)	-5.98 (-33.1 to 12.9)
Sense organ diseases										
Blindness and vision loss										
Glaucoma										
Cataract										
Age-related macular degeneration										
Refraction disorders										
Near vision loss										
Other vision loss										
Age-related and other hearing loss										
Other sense organ diseases										
Musculoskeletal disorders	6.96 (6.15 to 7.86)	19.1 (15.6 to 21.4)	48.4 (33.5 to 57.6)	162 (137 to 203)	5.96 (-4.14 to 22.5)	10.9 (7.76 to 13.4)	27.8 (18 to 32.8)	77.1 (41.6 to 99.4)	158 (109 to 199)	18.3 (-4.29 to 37.2)
Rheumatoid arthritis	3.31 (2.84 to 3.76)	7.65 (6.44 to 8.6)	11.3 (8.22 to 13.9)	147 (119 to 193)	-0.00665 (-11.5 to 18.4)	5.79 (3.94 to 7.13)	12 (7.52 to 14.6)	17.8 (10.8 to 23.6)	124 (70.1 to 176)	2.59 (-22 to 26.5)
Osteoarthritis										
Osteoarthritis hip										
Osteoarthritis knee										
Osteoarthritis hand										
Osteoarthritis other										
Low back pain										
Neck pain										
Gout										
Other musculoskeletal disorders	3.65 (3.15 to 4.21)	11.4 (9 to 12.9)	37.1 (25.1 to 44.5)	175 (147 to 213)	11 (-0.0193 to 26.5)	5.07 (3.51 to 6.41)	15.8 (9.67 to 19.1)	59.3 (30.6 to 75.9)	197 (152 to 247)	36.1 (15.6 to 58.9)
Other non-communicable diseases	36.6 (30.3 to 39.9)	120 (95.8 to 132)	494 (338 to 565)	178 (149 to 203)	12.5 (0.8 to 22.5)	30.9 (26.8 to 33.4)	105 (82.6 to 117)	456 (312 to 525)	203 (149 to 227)	38.7 (14 to 50.1)
Congenital birth defects										
Neural tube defects										
Congenital heart anomalies										
Orofacial clefts										
Down syndrome										
Turner syndrome										
Klinefelter syndrome										
Other chromosomal abnormalities										
Congenital musculoskeletal and limb anomalies										
Urogenital congenital anomalies										
Digestive congenital anomalies										
Other congenital birth defects										
Urinary diseases and male infertility	23.5 (18.8 to 25.8)	85.7 (67.1 to 94.4)	356 (240 to 409)	172 (138 to 202)	10.1 (-3.59 to 22.1)	18.1 (15.1 to 19.8)	68.5 (52.4 to 76.3)	317 (216 to 365)	225 (158 to 254)	49.2 (18.1 to 62.2)

Supplementary Table 4. Cause-specific mortality rate for 70-79 years, 80-94 years, and 95+ years in 2019, and percentage change from 1990 to 2019 in death number and rate in 70+ years, global, for all GBD causes

	Males					Females				
	Deaths per 100,000, 2019			Percentage change in 70+, 1990-2019		Deaths per 100,000, 2019			Percentage change in 70+, 1990-2019	
Causes	70 to 79	80 to 94	95 plus	Number	Rate	70 to 79	80 to 94	95 plus	Number	Rate
Urinary tract infections and interstitial nephritis	16.7 (12.9 to 18.9)	65.4 (47.9 to 73)	302 (204 to 350)	196 (145 to 229)	19.8 (-0.834 to 33)	15.6 (13.2 to 17.4)	61.7 (47.3 to 68.8)	291 (199 to 335)	239 (164 to 269)	55.3 (21.2 to 69.2)
Urolithiasis	1.31 (0.848 to 1.83)	3.25 (2.18 to 4.44)	7.42 (4.97 to 11.3)	40.1 (2.65 to 105)	-43.3 (-58.5 to -17.2)	0.92 (0.726 to 1.16)	2.29 (1.69 to 3.27)	8.65 (5.58 to 15.7)	64.2 (33.6 to 133)	-24.7 (-38.7 to 6.97)
Benign prostatic hyperplasia										
Male infertility										
Other urinary diseases	5.41 (3.84 to 7.31)	17.1 (12.9 to 22.4)	47 (30.9 to 60.9)	150 (118 to 186)	1.04 (-11.9 to 15.8)	1.57 (0.705 to 2.13)	4.55 (2.08 to 6.22)	16.8 (6.45 to 25.9)	237 (107 to 318)	54.3 (-5.29 to 91.6)
Gynecological diseases						0.773 (0.364 to 1.05)	1.7 (0.884 to 2.3)	2.59 (1.71 to 3.56)	98.3 (22 to 200)	-9.07 (-44.1 to 37.5)
Uterine fibroids						0.43 (0.187 to 0.638)	0.742 (0.342 to 1.12)	0.675 (0.4 to 1.07)	105 (3.34 to 261)	-5.95 (-52.6 to 65.6)
Polycystic ovarian syndrome										
Female infertility										
Endometriosis										
Genital prolapse						0.193 (0.0808 to 0.308)	0.406 (0.199 to 0.608)	0.575 (0.337 to 0.869)	70.3 (10.5 to 162)	-21.9 (-49.3 to 20.2)
Premenstrual syndrome										
Other gynecological diseases						0.149 (0.0773 to 0.18)	0.551 (0.31 to 0.679)	1.34 (0.866 to 1.72)	118 (61.3 to 196)	-0.256 (-26.1 to 35.7)
Hemoglobinopathies and hemolytic anemias	3.03 (2.58 to 4.06)	7.11 (6.11 to 8.95)	17.4 (13.5 to 20.3)	64.2 (42 to 81)	-33.6 (-42.6 to -26.8)	2.74 (2.38 to 3.46)	6.48 (5.18 to 7.99)	16.7 (11.6 to 21.4)	70.1 (48 to 102)	-22 (-32.1 to -7.22)
Thalassemias	0.0036 (0.00177 to 0.00641)	0.000218 (0.0000751 to 0.000512)	0.00000231 (0.000000366 to 0.00000713)	619 (392 to 899)	191 (98.7 to 304)	0.00479 (0.00246 to 0.00823)	0.000264 (0.0001 to 0.000603)	0.00000489 (0.000000995 to 0.0000148)	442 (309 to 683)	149 (87.4 to 259)
Thalassemias trait										
Sickle cell disorders	0.00458 (0.00278 to 0.00733)	0.000479 (0.000198 to 0.000917)	0.0000595 (0.0000153 to 0.000152)	473 (340 to 686)	132 (78 to 218)	0.0125 (0.0082 to 0.0184)	0.00164 (0.000783 to 0.00284)	0.000164 (0.0000525 to 0.000386)	384 (284 to 569)	122 (76.1 to 207)
Sickle cell trait										
G6PD deficiency	0.586 (0.339 to 0.986)	0.365 (0.197 to 0.639)	0.146 (0.06 to 0.261)	244 (184 to 306)	39 (14.7 to 64.1)	0.223 (0.126 to 0.357)	0.139 (0.0767 to 0.227)	0.0468 (0.018 to 0.0854)	292 (239 to 383)	79.9 (55.4 to 121)
G6PD trait										
Other hemoglobinopathies and hemolytic anemias	2.44 (2.07 to 3.17)	6.75 (5.8 to 8.33)	17.2 (13.4 to 20.1)	52.9 (32.2 to 69.7)	-38.2 (-46.6 to -31.4)	2.5 (2.16 to 3.14)	6.34 (5.06 to 7.81)	16.7 (11.6 to 21.3)	65.2 (43.5 to 96.5)	-24.3 (-34.2 to -9.92)
Endocrine, metabolic, blood, and immune disorders	10.1 (7.33 to 11.8)	27.6 (18.5 to 31.6)	120 (61.2 to 147)	269 (225 to 307)	49.3 (31.6 to 64.4)	9.3 (7.34 to 10.3)	28.6 (20.3 to 32.4)	120 (70 to 141)	224 (171 to 254)	48.3 (24.2 to 62.3)
Oral disorders										
Caries of deciduous teeth										
Caries of permanent teeth										
Periodontal diseases										
Edentulism										
Other oral disorders										
Sudden infant death syndrome										
Injuries	175 (153 to 191)	400 (344 to 437)	1020 (810 to 1160)	114 (97 to 131)	-13.5 (-20.4 to -6.59)	107 (93.2 to 119)	299 (241 to 336)	881 (653 to 1010)	103 (78.5 to 125)	-7.02 (-18.2 to 3.11)
Transport injuries	44.7 (36.3 to 49.3)	61.1 (51.9 to 67)	73.2 (58 to 85.4)	90.9 (66.3 to 110)	-22.8 (-32.8 to -15.3)	19.7 (17.9 to 21.6)	25.9 (21.7 to 28.6)	31.7 (24.1 to 36.2)	69 (56.5 to 83.8)	-22.5 (-28.3 to -15.7)
Road injuries	41.9 (34 to 46.1)	57.4 (48.9 to 62.9)	67.4 (53.3 to 78.1)	90 (65.5 to 109)	-23.2 (-33.1 to -15.6)	18.8 (17.1 to 20.6)	24.5 (20.5 to 27.1)	29.7 (22.6 to 33.8)	68.7 (56.4 to 84)	-22.6 (-28.3 to -15.6)
Pedestrian road injuries	22 (17.2 to 25.4)	32.3 (26.4 to 36.6)	34.8 (27.6 to 40.8)	77 (44.5 to 117)	-28.4 (-41.6 to -12.2)	11 (9.82 to 12.2)	15.3 (12.7 to 17)	15.8 (12 to 18.3)	52.3 (38.8 to 66.9)	-30.2 (-36.3 to -23.5)
Cyclist road injuries	3.73 (2.9 to 4.41)	3.83 (3.04 to 4.53)	0.112 (0.0852 to 0.136)	148 (75.8 to 244)	0.105 (-28.9 to 39)	0.956 (0.833 to 1.1)	0.741 (0.607 to 0.852)	0.0197 (0.0144 to 0.0235)	155 (116 to 209)	17 (-0.91 to 41.5)
Motorcyclist road injuries	3.46 (2.44 to 4.06)	3.9 (2.75 to 4.79)	3.87 (2.81 to 4.74)	185 (99.6 to 296)	15.4 (-19.3 to 60.1)	0.967 (0.677 to 1.13)	0.83 (0.599 to 0.976)	1.14 (0.815 to 1.37)	201 (141 to 289)	38.1 (10.7 to 78.5)
Motor vehicle road injuries	12.2 (10.3 to 14.2)	16.4 (13.7 to 19.5)	27.2 (21.3 to 33.1)	85.5 (49.9 to 115)	-25 (-39.4 to -12.9)	5.78 (5.14 to 6.51)	7.44 (6.15 to 8.39)	12.4 (9.16 to 14.5)	86.6 (60.4 to 112)	-14.5 (-26.5 to -2.64)
Other road injuries	0.592 (0.481 to 0.698)	0.885 (0.71 to 1.07)	1.3 (0.958 to 1.61)	121 (62.4 to 217)	-10.8 (-34.4 to 28.2)	0.127 (0.106 to 0.153)	0.23 (0.187 to 0.289)	0.393 (0.288 to 0.517)	116 (78.6 to 181)	-1.03 (-18.1 to 28.9)
Other transport injuries	2.82 (2.22 to 3.19)	3.73 (3.12 to 4.23)	5.84 (4.6 to 7.18)	106 (76.8 to 137)	-16.8 (-28.5 to -4.12)	0.87 (0.774 to 0.986)	1.37 (1.16 to 1.57)	2.02 (1.54 to 2.4)	74.5 (46.8 to 98.9)	-20 (-32.7 to -8.82)
Unintentional injuries	92.7 (78.1 to 103)	281 (237 to 310)	875 (696 to 1010)	141 (118 to 162)	-2.59 (-11.7 to 6.11)	70.4 (57.6 to 79.6)	251 (198 to 283)	830 (615 to 955)	129 (101 to 158)	5.2 (-7.94 to 18.3)
Falls	53.2 (42.2 to 60.4)	195 (159 to 220)	662 (526 to 779)	189 (153 to 226)	16.8 (2.31 to 31.6)	43.6 (33 to 50)	188 (145 to 214)	652 (477 to 753)	150 (116 to 187)	14.5 (-0.773 to 31.8)
Drowning	7.28 (6.04 to 8.12)	13.7 (11.3 to 15.3)	15.3 (11.7 to 17.6)	122 (76.9 to 157)	-10.3 (-28.5 to 3.71)	4.74 (4.12 to 5.34)	9.26 (7.38 to 10.7)	9.38 (6.74 to 11)	109 (72.6 to 153)	-4.02 (-20.9 to 15.9)
Fire, heat, and hot substances	4.25 (3.45 to 4.97)	11 (8.86 to 13.1)	24.7 (19 to 30)	57.6 (43.1 to 84.7)	-36.3 (-42.1 to -25.3)	3.66 (2.88 to 4.55)	9.67 (7.68 to 12)	22 (16.1 to 27.1)	61.6 (38.7 to 103)	-25.9 (-36.4 to -6.73)
Poisonings	3.07 (2.05 to 3.57)	4.86 (3.48 to 5.56)	3.52 (2.78 to 4.02)	105 (40.2 to 146)	-17.3 (-43.3 to -0.374)	1.96 (1.65 to 2.27)	3.28 (2.7 to 3.74)	2.92 (2.22 to 3.38)	106 (71.6 to 137)	-5.73 (-21.3 to 8.86)
Poisoning by carbon monoxide	1.79 (1.14 to 2.15)	2.98 (1.98 to 3.51)	2 (1.53 to 2.31)	112 (43 to 162)	-14.2 (-42.2 to 6.02)	1.15 (0.867 to 1.38)	2.07 (1.58 to 2.43)	1.76 (1.28 to 2.09)	93.3 (49.5 to 129)	-11.4 (-31.5 to 4.97)
Poisoning by other means	1.29 (0.733 to 1.77)	1.89 (1.16 to 2.65)	1.52 (1.18 to 1.83)	94.5 (19.1 to 154)	-21.4 (-51.8 to 2.71)	0.813 (0.549 to 1.08)	1.21 (0.876 to 1.58)	1.16 (0.85 to 1.45)	128 (62.2 to 191)	4.62 (-25.7 to 33.6)
Exposure to mechanical forces	3.74 (2.86 to 4.27)	5.79 (4.6 to 6.5)	11.9 (9.27 to 14.8)	81 (34.7 to 117)	-26.8 (-45.6 to -12.1)	1.78 (1.3 to 2.04)	4.01 (3.1 to 4.55)	9.29 (6.83 to 10.9)	127 (70.8 to 164)	3.84 (-21.7 to 21.1)
Unintentional firearm injuries	0.43 (0.283 to 0.558)	0.698 (0.457 to 0.891)	1.65 (1.11 to 2.44)	12 (-16.1 to 62.2)	-54.7 (-66.1 to -34.4)	0.229 (0.175 to 0.297)	0.525 (0.399 to 0.675)	1.64 (1.16 to 2.11)	41.8 (-1.51 to 99.3)	-35 (-54.8 to -8.64)
Other exposure to mechanical forces	3.31 (2.5 to 3.81)	5.09 (4.05 to 5.76)	10.2 (7.96 to 12.5)	97.1 (44.8 to 144)	-20.3 (-41.5 to -1.43)	1.55 (1.12 to 1.79)	3.49 (2.64 to 4)	7.65 (5.61 to 8.95)	149 (86.1 to 197)	14.2 (-14.7 to 35.9)
Adverse effects of medical treatment	6.85 (5.51 to 7.88)	15.3 (12.6 to 17.3)	37.9 (29 to 43.5)	73.8 (33.9 to 115)	-29.8 (-45.9 to -13)	6.01 (4.71 to 6.88)	13.5 (10.5 to 15.5)	31.3 (22.6 to 37.1)	72.5 (32.6 to 101)	-20.9 (-39.2 to -7.72)
Animal contact	3.68 (2.55 to 4.46)	5.04 (3.25 to 6.03)	6.49 (4.6 to 8.14)	57.9 (12.1 to 124)	-36.2 (-54.7 to -9.4)	2.81 (1.49 to 3.72)	2.69 (1.63 to 3.42)	2.7 (1.93 to 3.59)	105 (38.9 to 215)	-5.85 (-36.3 to 44.6)
Venomous animal contact	3.01 (1.97 to 3.71)	4.08 (2.45 to 5)	4.74 (3.19 to 6.02)	63.7 (13.7 to 139)	-33.8 (-54 to -3.24)	2.63 (1.33 to 3.5)	2.34 (1.35 to 3)	2.04 (1.43 to 2.78)	110 (37.1 to 229)	-3.94 (-37.1 to 50.9)
Non-venomous animal contact	0.676 (0.541 to 0.902)	0.96 (0.771 to 1.22)	1.76 (1.33 to 2.15)	36.8 (-0.148 to 80.1)	-44.7 (-59.6 to -27.2)	0.178 (0.136 to 0.244)	0.348 (0.265 to 0.449)	0.663 (0.478 to 0.848)	70.1 (34.3 to 130)	-22 (-38.4 to 5.41)
Foreign body	5.37 (4.99 to 5.69)	20 (17.3 to 21.6)	92.4 (71.5 to 104)	187 (168 to 203)	16.1 (8.39 to 22.5)	3.26 (3.01 to 3.49)	14.1 (11.2 to 15.6)	85.8 (61.4 to 99.4)	184 (158 to 203)	30.4 (18.1 to 38.8)
Pulmonary aspiration and foreign body in airway	5.17 (4.78 to 5.47)	19.5 (16.9 to 21)	91.3 (70.5 to 103)	196 (176 to 211)	19.6 (11.7 to 25.7)	3.14 (2.9 to 3.35)	13.7 (10.9 to 15.2)	84.8 (60.6 to 98.2)	192 (164 to 211)	33.7 (21.1 to 42.5)
Foreign body in eyes										
Foreign body in other body part	0.21 (0.152 to 0.275)	0.504 (0.376 to 0.677)	1.12 (0.789 to 1.55)	47 (14.9 to 96.6)	-40.6 (-53.6 to -20.5)	0.126 (0.0966 to 0.167)	0.326 (0.244 to 0.442)	1.08 (0.742 to 1.67)	45.6 (22.6 to 85.3)	-33.2 (-43.8 to -15)
Environmental heat and cold exposure	2.88 (1.59 to 3.54)	5.91 (3.47 to 6.99)	11.2 (8.37 to 12.8)	42.9 (18 to 66.5)	-42.2 (-52.3 to -32.7)	1.61 (0.724 to 2.26)	4.39 (2.37 to 5.75)	10 (6.79 to 12.3)	51 (4.27 to 87.2)	-30.8 (-52.2 to -14.2)
Exposure to forces of nature	0.116 (0.106 to 0.128)	0.15 (0.136 to 0.165)	0.156 (0.142 to 0.172)	-80.7 (-80.7 to -80.7)	-92.2 (-92.2 to -92.2)	0.0968 (0.0879 to 0.107)	0.119 (0.108 to 0.131)	0.107 (0.0974 to 0.118)	-74.8 (-74.8 to -74.8)	-88.4 (-88.4 to -88.4)

Supplementary Table 4. Cause-specific mortality rate for 70-79 years, 80-94 years, and 95+ years in 2019, and percentage change from 1990 to 2019 in death number and rate in 70+ years, global, for all GBD causes

	Males					Females				
	Deaths per 100,000, 2019			Percentage change in 70+, 1990-2019		Deaths per 100,000, 2019			Percentage change in 70+, 1990-2019	
Causes	70 to 79	80 to 94	95 plus	Number	Rate	70 to 79	80 to 94	95 plus	Number	Rate
Other unintentional injuries	2.29 (1.61 to 2.56)	3.73 (2.67 to 4.18)	9.07 (6.74 to 10.8)	49.2 (13 to 74.8)	-39.7 (-54.3 to -29.3)	0.903 (0.808 to 1.01)	2.12 (1.75 to 2.4)	5.09 (3.79 to 5.87)	45.6 (27.2 to 68.7)	-33.2 (-41.7 to -22.7)
Self-harm and interpersonal violence	37.6 (32.5 to 41.7)	57.6 (49 to 64.2)	70.4 (56.2 to 79.7)	71 (57.8 to 95.6)	-30.9 (-36.2 to -20.9)	17.3 (15.6 to 19.1)	22.6 (18.9 to 25.5)	18.9 (14.4 to 21.6)	24.3 (11 to 37.6)	-43 (-49.1 to -36.9)
Self-harm	30.3 (25.3 to 34)	51 (42.8 to 57.3)	52.6 (41.4 to 61.3)	67.6 (52.9 to 93.8)	-32.2 (-38.2 to -21.6)	13.4 (11.9 to 15.1)	19.2 (15.7 to 21.9)	16.1 (12 to 18.6)	14.4 (0.0522 to 29.2)	-47.5 (-54.1 to -40.8)
Self-harm by firearm	3.16 (2.71 to 3.48)	5.54 (4.69 to 6.35)	8.5 (6.73 to 11)	36.2 (14.5 to 47.9)	-44.9 (-53.7 to -40.2)	0.239 (0.218 to 0.262)	0.264 (0.219 to 0.298)	0.521 (0.381 to 0.616)	37.3 (26.3 to 46.5)	-37.1 (-42.1 to -32.8)
Self-harm by other specified means	27.2 (21.9 to 30.9)	45.4 (37.6 to 51.4)	44.1 (34 to 51.3)	72.3 (55.5 to 104)	-30.3 (-37.1 to -17.6)	13.1 (11.6 to 14.9)	18.9 (15.5 to 21.6)	15.6 (11.6 to 18.1)	14.1 (-0.353 to 29)	-47.7 (-54.3 to -40.8)
Interpersonal violence	5.55 (5.17 to 6.06)	2.95 (2.59 to 3.26)	11.7 (8.86 to 13.7)	60.3 (45.3 to 86.9)	-35.2 (-41.3 to -24.4)	2.46 (2.27 to 2.65)	1.46 (1.23 to 1.59)	1.55 (1.17 to 1.78)	34.7 (25.9 to 45.1)	-38.2 (-42.3 to -33.5)
Physical violence by firearm	1.4 (1.28 to 1.53)	0.793 (0.676 to 0.89)	4.32 (3.14 to 5.27)	85.4 (64.4 to 111)	-25.1 (-33.5 to -14.8)	0.355 (0.329 to 0.382)	0.193 (0.16 to 0.213)	0.298 (0.217 to 0.359)	55.6 (42.2 to 70)	-28.7 (-34.8 to -22.1)
Physical violence by sharp object	1.29 (1.08 to 1.48)	0.835 (0.714 to 0.965)	3.34 (2.52 to 3.93)	56.7 (36.1 to 86.7)	-36.7 (-45 to -24.5)	0.625 (0.577 to 0.672)	0.436 (0.373 to 0.479)	0.411 (0.312 to 0.483)	38.8 (24.1 to 51.5)	-36.4 (-43.1 to -30.5)
Sexual violence										
Physical violence by other means	2.86 (2.6 to 3.19)	1.33 (1.15 to 1.51)	4.03 (3.09 to 4.66)	51.4 (34.1 to 80.1)	-38.8 (-45.8 to -27.2)	1.48 (1.35 to 1.61)	0.828 (0.706 to 0.904)	0.841 (0.636 to 0.952)	28.9 (19.3 to 38.9)	-40.9 (-45.3 to -36.3)
Conflict and terrorism	1.61 (1.46 to 1.77)	3.51 (3.19 to 3.87)	5.62 (5.1 to 6.18)	225 (220 to 226)	31.2 (29.2 to 32)	1.39 (1.26 to 1.53)	1.9 (1.73 to 2.09)	1.15 (1.04 to 1.27)	344 (339 to 346)	104 (101 to 104)
Executions and police conflict	0.0776 (0.0612 to 0.0969)	0.117 (0.0986 to 0.139)	0.524 (0.356 to 0.828)	217 (147 to 408)	27.9 (-0.136 to 105)	0.045 (0.0398 to 0.0547)	0.0572 (0.0513 to 0.0655)	0.0587 (0.0413 to 0.0937)	347 (239 to 446)	105 (55.4 to 150)
Total cancers	1080 (1000 to 1170)	2010 (1800 to 2160)	3210 (2500 to 3580)	133 (116 to 150)	-5.88 (-12.7 to 1.14)	621 (569 to 666)	1120 (925 to 1230)	2130 (1540 to 2420)	110 (94.1 to 124)	-3.89 (-11 to 2.5)
Total burden related to hepatitis B	42.4 (34.4 to 51.1)	54.9 (44.2 to 66.3)	65.6 (41.1 to 96.9)	50.3 (32.1 to 73.1)	-39.2 (-46.6 to -30)	16.5 (12.6 to 20.5)	22.9 (17.1 to 28.8)	29.2 (17.2 to 44.3)	24.6 (8.1 to 44.3)	-42.9 (-50.4 to -33.9)
Total burden related to hepatitis C	44 (36.9 to 52.1)	76.3 (63.2 to 90.1)	137 (93.9 to 180)	122 (103 to 147)	-10.1 (-18.1 to -0.227)	27.5 (22.8 to 32.8)	50 (39.1 to 58.9)	96.5 (64.3 to 127)	86.7 (64.8 to 108)	-14.4 (-24.4 to -4.82)
Total burden related to Non-alcoholic fatty liver disease (NAFLD)	13.1 (9.11 to 18.1)	22.9 (15.8 to 31.6)	42.4 (20.7 to 73.3)	139 (114 to 168)	-3.3 (-13.3 to 8.2)	12.1 (8.49 to 16.5)	21.5 (14.7 to 29.3)	36.1 (18.4 to 58.4)	112 (86.5 to 136)	-2.93 (-14.5 to 8.19)

Supplementary Table 5. Cause-specific rate of YLDs for 70-79 years, 80-94 years, and 95+ years in 2019, and percentage change from 1990 to 2019 in YLD number and rate in 70+ years, global, for all GBD causes

	Males					Females				
	YLDs per 100,000, 2019			Percentage change in 70+, 1990-2019		YLDs per 100,000, 2019			Percentage change in 70+, 1990-2019	
Causes	70 to 79	80 to 94	95 plus	Number	Rate	70 to 79	80 to 94	95 plus	Number	Rate
All causes	25000 (18900 to 31800)	31000 (23400 to 39100)	37900 (29200 to 46900)	147 (145 to 149)	-0.0825 (-0.876 to 0.694)	26600 (20200 to 33500)	33100 (25200 to 41500)	41100 (31800 to 50500)	121 (120 to 123)	1.54 (0.84 to 2.32)
Communicable, maternal, neonatal, and nutritional diseases	1150 (845 to 1510)	1110 (810 to 1440)	988 (720 to 1280)	77.3 (70.8 to 84)	-28.3 (-30.9 to -25.6)	1150 (856 to 1500)	991 (733 to 1280)	776 (565 to 1010)	88.2 (82 to 94.9)	-13.7 (-16.6 to -10.6)
HIV/AIDS and sexually transmitted infections	29.4 (20.2 to 41.1)	13.8 (9.15 to 20.2)	11.7 (7.9 to 16.6)	411 (316 to 529)	107 (68.1 to 154)	28.1 (18.8 to 41.2)	12.9 (7.71 to 21.7)	9.2 (5.61 to 15.3)	292 (210 to 415)	79.8 (42 to 136)
HIV/AIDS	24.8 (16.8 to 35)	9.56 (6.11 to 14.4)	7.23 (4.83 to 10.2)	614 (442 to 803)	189 (119 to 265)	18.4 (12.8 to 25.4)	5.64 (3.71 to 7.99)	3.87 (2.65 to 5.42)	767 (563 to 991)	297 (204 to 400)
HIV/AIDS - Drug-susceptible Tuberculosis	3.36 (2.3 to 4.52)	1.46 (1 to 1.95)	0.703 (0.489 to 0.932)	194 (170 to 223)	18.9 (9.12 to 30.6)	3.01 (2.08 to 4)	0.951 (0.655 to 1.26)	0.547 (0.382 to 0.722)	312 (279 to 350)	88.9 (73.7 to 106)
HIV/AIDS - Multidrug-resistant Tuberculosis without extensive drug resistance	0.157 (0.086 to 0.276)	0.0696 (0.0334 to 0.14)	0.0308 (0.0176 to 0.0511)	1470 (496 to 4610)	535 (141 to 1800)	0.125 (0.0726 to 0.198)	0.0455 (0.0255 to 0.0776)	0.0269 (0.015 to 0.0455)	3140 (1410 to 7330)	1390 (592 to 3310)
HIV/AIDS - Extensively drug-resistant Tuberculosis	0.00636 (0.00263 to 0.0145)	0.00336 (0.00106 to 0.00927)	0.000944 (0.000514 to 0.00168)	0 (0 to 0)	0 (0 to 0)	0.00314 (0.00158 to 0.00617)	0.00105 (0.000473 to 0.00234)	0.000632 (0.000311 to 0.00129)	0 (0 to 0)	0 (0 to 0)
HIV/AIDS resulting in other diseases	21.3 (14 to 30.8)	8.03 (4.89 to 12.6)	6.5 (4.21 to 9.37)	822 (544 to 1150)	273 (160 to 406)	15.3 (10.3 to 21.6)	4.64 (2.98 to 6.9)	3.3 (2.19 to 4.78)	1000 (674 to 1420)	404 (255 to 597)
Sexually transmitted infections excluding HIV	4.62 (2.24 to 9.71)	4.22 (2.19 to 8.43)	4.46 (2.6 to 8.05)	123 (107 to 133)	-9.65 (-16.1 to -5.74)	9.64 (3.8 to 21.1)	7.3 (2.97 to 15.3)	5.33 (2.44 to 11.4)	110 (103 to 114)	-3.65 (-6.82 to -1.82)
Syphilis	1.16 (0.786 to 1.63)	1.34 (0.91 to 1.87)	2.03 (1.3 to 2.93)	91.1 (76.5 to 106)	-22.7 (-28.6 to -16.7)	0.391 (0.253 to 0.55)	0.51 (0.333 to 0.721)	0.907 (0.57 to 1.3)	58.6 (46.1 to 72.3)	-27.3 (-33 to -21)
Chlamydial infection	0.000278 (0.00013 to 0.000519)	0.000000000000000259 (0.0000000000000000655 to 0.000000000000000762)	0.0000000000000000000000000000000000378 (0.00000000000000000000000	145 (141 to 150)	-0.906 (-2.71 to 1.09)	0.0000784 (0.0000253 to 0.000188)	0.0000000373 (0.000000012 to 0.0000000938)	0.000000000000317 (0.00000000000009 to 0.000000000000891)	115 (111 to 119)	-1.47 (-3.34 to 0.47)
Gonococcal infection	0.000643 (0.000186 to 0.0017)	0.000000000948 (0.000000000000000305 to 0.00000000399)	0.0000000000000000181 (0.000000000000000000000000000000000444 to	106 (82.8 to 130)	-16.8 (-26.1 to -6.95)	0.000046 (0.00000812 to 0.000144)	0.0000000101 (0.00000000000765 to 0.0000000773)	0.000000000000266 (0.00000000000000000000000817 to 0.00000000000118)	102 (74.3 to 131)	-7.43 (-20.1 to 6)
Trichomoniasis	0.113 (0.0446 to 0.26)	0.0659 (0.0261 to 0.149)	0.0133 (0.005 to 0.0305)	141 (124 to 158)	-2.67 (-9.54 to 4.36)	4.02 (1.51 to 9.15)	2.43 (0.951 to 5.42)	0.764 (0.249 to 1.85)	115 (110 to 121)	-1.28 (-3.89 to 1.53)
Genital herpes	3.34 (1.01 to 8.36)	2.81 (0.838 to 7)	2.42 (0.739 to 5.87)	138 (134 to 143)	-3.62 (-5.37 to -1.81)	5.23 (1.58 to 12.8)	4.36 (1.3 to 10.8)	3.65 (1.11 to 8.9)	113 (109 to 117)	-2.46 (-4.29 to -0.691)
Other sexually transmitted infections	0.000384 (0.000158 to 0.00086)	0.000000000371 (0.000000000000000232 to 0.00000000156)	0.00000000000000000975 (0.000000000000000000000000000000000298 to	105 (87.6 to 122)	-17.3 (-24.1 to -10.2)	0.0000519 (0.000016 to 0.000129)	0.0000000186 (0.00000000495 to 0.0000000623)	0.000000000000314 (0.0000000000000488 to 0.00000000000101)	98.3 (87.7 to 109)	-9.11 (-13.9 to -4.29)
Respiratory infections and tuberculosis	238 (162 to 326)	221 (158 to 297)	200 (143 to 277)	68.9 (56.9 to 83.2)	-31.7 (-36.6 to -25.9)	167 (115 to 231)	153 (109 to 206)	132 (93.6 to 183)	64.2 (53.4 to 76.5)	-24.7 (-29.7 to -19.1)
Tuberculosis	146 (90 to 216)	118 (77.1 to 169)	62.8 (38.6 to 97.2)	42.5 (33 to 52.5)	-42.4 (-46.2 to -38.3)	87.7 (53.7 to 132)	69.7 (46 to 100)	30.9 (18.8 to 46.8)	37.4 (28.3 to 46.9)	-37 (-41.2 to -32.7)
Latent tuberculosis infection				0 (0 to 0)	0 (0 to 0)				0 (0 to 0)	0 (0 to 0)
Drug-susceptible tuberculosis	139 (84.9 to 205)	113 (73.8 to 162)	60.3 (36.8 to 92.7)	37.1 (26.5 to 47.4)	-44.6 (-48.9 to -40.4)	83.5 (51.1 to 124)	66.5 (43.8 to 95.4)	29.6 (18.1 to 45)	31.8 (21.9 to 42)	-39.6 (-44.1 to -34.9)
Multidrug-resistant tuberculosis without extensive drug resistance	6.54 (2.52 to 13.7)	5.18 (2.23 to 10.3)	2.41 (1.07 to 4.66)	573 (115 to 2200)	172 (-13.1 to 828)	4.03 (1.54 to 8.32)	3.11 (1.34 to 6.28)	1.2 (0.589 to 2.2)	690 (168 to 2250)	262 (22.9 to 978)
Extensively drug-resistant tuberculosis	0.393 (0.155 to 0.886)	0.299 (0.127 to 0.631)	0.132 (0.0657 to 0.236)	0 (0 to 0)	0 (0 to 0)	0.223 (0.0991 to 0.447)	0.171 (0.0828 to 0.317)	0.0749 (0.0373 to 0.135)	0 (0 to 0)	0 (0 to 0)
Lower respiratory infections	25.8 (17.3 to 35.9)	43.2 (29.5 to 60.3)	73.3 (50.3 to 105)	123 (118 to 129)	-9.68 (-12 to -7.6)	19 (12.8 to 26.5)	31.2 (21.5 to 43.5)	48.9 (33.6 to 69.1)	92.7 (87.8 to 97.9)	-11.7 (-13.9 to -9.26)
Upper respiratory infections	52.9 (29.7 to 87.1)	45.9 (25.8 to 76)	49 (26.6 to 84.4)	142 (138 to 147)	-2.14 (-3.95 to -0.034)	49.9 (28.1 to 82.8)	41.5 (23.5 to 69.9)	41.7 (22.5 to 71.9)	113 (109 to 118)	-2.32 (-4.21 to -0.182)
Otitis media	13.1 (6.44 to 23.7)	13.5 (6.63 to 23.7)	14.7 (7.06 to 26.2)	111 (94.2 to 131)	-14.7 (-21.5 to -6.51)	10.7 (5.24 to 19.6)	10.5 (5.1 to 18.6)	11 (5.16 to 20.4)	91.7 (77.4 to 108)	-12.1 (-18.7 to -4.48)
Enteric infections	253 (178 to 341)	308 (220 to 409)	309 (217 to 410)	172 (166 to 179)	9.9 (7.49 to 12.7)	235 (166 to 316)	273 (196 to 361)	266 (189 to 352)	180 (174 to 187)	28.5 (25.5 to 31.4)
Diarrheal diseases	253 (178 to 341)	308 (220 to 409)	308 (217 to 410)	173 (167 to 180)	10.2 (7.8 to 13)	235 (166 to 316)	273 (196 to 361)	265 (189 to 352)	181 (174 to 187)	28.8 (25.8 to 31.6)
Typhoid and paratyphoid	0.357 (0.164 to 0.643)	0.338 (0.163 to 0.576)	0.372 (0.185 to 0.717)	-10.3 (-25.9 to 6.74)	-63.7 (-70 to -56.8)	0.292 (0.138 to 0.524)	0.243 (0.118 to 0.426)	0.156 (0.0742 to 0.321)	5.18 (-11 to 23.8)	-51.8 (-59.2 to -43.3)
Typhoid fever	0.311 (0.143 to 0.559)	0.299 (0.141 to 0.505)	0.337 (0.165 to 0.66)	-13.2 (-29.5 to 5.29)	-64.9 (-71.5 to -57.4)	0.255 (0.12 to 0.466)	0.215 (0.103 to 0.374)	0.14 (0.0654 to 0.288)	3.71 (-14.3 to 24.1)	-52.5 (-60.7 to -43.1)
Paratyphoid fever	0.046 (0.0188 to 0.0913)	0.0389 (0.0176 to 0.0699)	0.0346 (0.0139 to 0.0759)	17.3 (-3.04 to 41.5)	-52.6 (-60.8 to -42.8)	0.0373 (0.0152 to 0.0741)	0.0281 (0.0125 to 0.051)	0.016 (0.00639 to 0.0352)	16.9 (-2.56 to 40.3)	-46.4 (-55.3 to -35.7)
Invasive Non-typhoidal Salmonella (iNTS)	0.00385 (0.00221 to 0.00654)	0.00392 (0.00229 to 0.00643)	0.00501 (0.0025 to 0.00843)	72.1 (53.7 to 89.4)	-30.4 (-37.9 to -23.5)	0.003 (0.00169 to 0.00508)	0.00275 (0.00157 to 0.00458)	0.00283 (0.00138 to 0.00484)	67.6 (50.1 to 85.8)	-23.2 (-31.2 to -14.8)
Other intestinal infectious diseases	0.0406 (0.0287 to 0.0548)	0.0655 (0.0469 to 0.0871)	0.147 (0.103 to 0.195)	266 (258 to 275)	47.8 (44.6 to 51.4)	0.0376 (0.0265 to 0.0504)	0.0602 (0.0433 to 0.0797)	0.127 (0.0902 to 0.168)	276 (267 to 284)	72.2 (68.1 to 75.8)
Neglected tropical diseases and malaria	195 (139 to 268)	182 (126 to 254)	168 (99.2 to 262)	4.38 (-6.73 to 18.4)	-57.8 (-62.3 to -52.1)	168 (118 to 226)	153 (101 to 215)	120 (68 to 188)	29.4 (16.1 to 44.8)	-40.7 (-46.8 to -33.6)
Malaria	2.95 (2.02 to 4.2)	2.6 (1.75 to 3.76)	2.01 (1.37 to 2.87)	-2.09 (-20.8 to 17.1)	-60.4 (-68 to -52.7)	4.21 (2.96 to 5.84)	3 (2.06 to 4.21)	1.96 (1.31 to 2.75)	9.67 (-7.3 to 28.4)	-49.7 (-57.5 to -41.2)
Chagas disease	3.07 (1.99 to 4.41)	4.33 (2.49 to 6.65)	9.84 (2.01 to 24.6)	61.8 (52.7 to 71.6)	-34.6 (-38.3 to -30.6)	2.05 (1.33 to 2.94)	2.65 (1.67 to 3.91)	3.93 (0.864 to 9.31)	59 (48.3 to 70.6)	-27.1 (-32 to -21.8)
Leishmaniasis	2.98 (1.81 to 4.73)	2.92 (1.79 to 4.54)	2.93 (1.86 to 4.36)	55.1 (33.4 to 94.6)	-37.3 (-46.1 to -21.3)	4.35 (2.7 to 6.66)	3.06 (1.92 to 4.66)	2.19 (1.36 to 3.35)	75.7 (55 to 123)	-19.5 (-28.9 to 2.31)
Visceral leishmaniasis	0.00227 (0.0014 to 0.00339)	0.00222 (0.00139 to 0.0033)	0.00336 (0.00206 to 0.00518)	-84.8 (-89 to -78.8)	-93.9 (-95.6 to -91.4)	0.0011 (0.000674 to 0.00162)	0.000926 (0.000574 to 0.00139)	0.00113 (0.000689 to 0.00174)	-82.6 (-87.2 to -76.1)	-92 (-94.1 to -89)
Cutaneous and mucocutaneous leishmaniasis	2.98 (1.81 to 4.73)	2.91 (1.79 to 4.54)	2.93 (1.86 to 4.35)	56.2 (33.8 to 97.3)	-36.9 (-45.9 to -20.2)	4.35 (2.7 to 6.66)	3.06 (1.92 to 4.66)	2.19 (1.36 to 3.35)	76.1 (55.3 to 124)	-19.3 (-28.8 to 2.81)
African trypanosomiasis	0.00591 (0.000662 to 0.0215)	0.00478 (0.000479 to 0.0193)	0.00323 (0.00012 to 0.0164)	-92.6 (-98.5 to -70)	-97 (-99.4 to -87.9)	0.00521 (0.000704 to 0.0165)	0.0042 (0.000474 to 0.0157)	0.00276 (0.000134 to 0.014)	-92.8 (-98.4 to -68.4)	-96.7 (-99.2 to -85.5)
Schistosomiasis	5.05 (2.46 to 9.85)	3.83 (1.9 to 7.19)	2.99 (1.38 to 5.78)	15.8 (6.65 to 25.4)	-53.2 (-56.9 to -49.3)	4.01 (2.01 to 7.66)	2.81 (1.42 to 5.2)	2.2 (1.01 to 4.24)	14.8 (5.45 to 24.9)	-47.4 (-51.7 to -42.8)
Cysticercosis	75 (39.8 to 119)	83 (42.9 to 135)	92.7 (36.6 to 172)	104 (78 to 136)	-17.7 (-28 to -4.48)	79.3 (43.6 to 124)	83.6 (44.9 to 132)	76.5 (31.2 to 137)	103 (80.9 to 130)	-6.88 (-17.1 to 5.52)
Cystic echinococcosis	0.518 (0.264 to 0.996)	0.434 (0.211 to 0.905)	0.268 (0.0981 to 0.81)	75.7 (54 to 98.4)	-29 (-37.8 to -19.8)	0.951 (0.551 to 1.56)	0.651 (0.367 to 1.13)	0.408 (0.193 to 0.942)	48.5 (37.4 to 60.5)	-31.9 (-37 to -26.4)
Lymphatic filariasis	29.6 (17.8 to 48)	21.1 (12.4 to 35.2)	12 (6.91 to 20.3)	-52.7 (-65 to -36.4)	-80.9 (-85.8 to -74.3)	5.15 (3.53 to 7.11)	3.58 (2.48 to 4.85)	1.64 (1.14 to 2.23)	-58.5 (-64.7 to -51.2)	-81 (-83.8 to -77.6)
Onchocerciasis	14.3 (9.64 to 20.2)	7.21 (4.8 to 10.4)	4.69 (3.06 to 6.81)	-11.7 (-15.3 to -7.12)	-64.3 (-65.8 to -62.5)	13.1 (8.79 to 18.7)	6.28 (4.19 to 8.96)	6.58 (4.33 to 9.46)	-17.8 (-21.9 to -12.4)	-62.3 (-64.2 to -59.9)
Trachoma	15.8 (9.32 to 25.3)	18.6 (10.9 to 29.9)	12.6 (6.24 to 22.1)	-38.8 (-43.5 to -33.4)	-75.2 (-77.1 to -73.1)	14.5 (8.39 to 23.5)	14.4 (8.3 to 23.6)	8.53 (4.07 to 15.2)	-21.9 (-28.1 to -15.4)	-64.2 (-67.1 to -61.2)
Dengue	5.07 (2.48 to 10)	5.48 (2.73 to 10.8)	18.1 (6.19 to 41.7)	252 (108 to 445)	42.5 (-15.9 to 120)	5.52 (2.63 to 10.9)	5.05 (2.47 to 9.8)	9.15 (2.51 to 20.5)	261 (123 to 453)	65.4 (2.08 to 153)
Yellow fever	0.000453 (0.000153 to 0.00109)	0.000356 (0.000116 to 0.000845)	0.000265 (0.0000845 to 0.000648)	-63.3 (-71.9 to -53.6)	-85.2 (-88.6 to -81.2)	0.000164 (0.0000547 to 0.00038)	0.000113 (0.0000388 to 0.000256)	0.0000778 (0.0000246 to 0.000197)	-66.9 (-74.1 to -57.4)	-84.8 (-88.1 to -80.5)
Rabies	0.00184 (0.000487 to 0.0036)	0.00128 (0.000432 to 0.00247)	0.00165 (0.000634 to 0.00356)	5.68 (-43.8 to 59.9)	-57.3 (-77.3 to -35.4)	0.000986 (0.000388 to 0.00208)	0.000604 (0.000208 to 0.00127)	0.00112 (0.000429 to 0.00217)	-11.5 (-48.8 to 49.6)	-59.4 (-76.5 to -31.4)
Intestinal nematode infections	6.11 (3.65 to 9.65)	5.08 (3.09 to 7.95)	3.23 (1.97 to 5.08)	-48.6 (-54.8 to -41.3)	-79.2 (-81.7 to -76.3)	5.9 (3.59 to 9.28)	3.85 (2.36 to 5.96)	1.21 (0.745 to 1.86)	-48.8 (-55.1 to -42)	-76.5 (-79.4 to -73.4)
Ascariasis	2.51 (1.4 to 4.17)	2.1 (1.19 to 3.44)	1.39 (0.791 to 2.25)	-42 (-52.9 to -28.1)	-76.6 (-81 to -70.9)	2.23 (1.24 to 3.71)	1.42 (0.792 to 2.35)	0.427 (0.237 to 0.692)	-46.4 (-56.9 to -33)	-75.4 (-80.2 to -69.3)
Trichuriasis	0.491 (0.266 to 0.822)	0.335 (0.186 to 0.554)	0.228 (0.127 to 0.376)	-32.2 (-43.7 to -19.3)	-72.6 (-77.2 to -67.4)	0.469 (0.252 to 0.775)	0.258 (0.142 to 0.42)	0.0683 (0.0369 to 0.114)	-33.3 (-44.7 to -20.8)	-69.4 (-74.6 to -63.7)
Hookworm disease	3.1 (1.9 to 4.79)	2.64 (1.66 to 4)	1.61 (1.03 to 2.39)	-54.4 (-62 to -45.8)	-81.6 (-84.6 to -78.1)	3.2 (2.02 to 4.82)	2.17 (1.36 to 3.24)	0.714 (0.459 to 1.06)	-51.8 (-59.8 to -43.1)	-77.9 (-81.6 to -73.9)
Food-borne trematodiases	26.7 (13.3 to 46)	19.9 (9.95 to 33.7)		40.2 (19.3 to 79.5)	-43.3 (-51.8 to -27.4)	19.3 (10.6 to 33)	16.4 (9.09 to 27)		18.7 (-1.56 to 53.5)	-45.6 (-54.9 to -29.6)
Leprosy	1.16 (0.732 to 1.68)	1.07 (0.679 to 1.52)	0.991 (0.65 to 1.43)	17 (4.85 to 30.1)	-52.7 (-57.6 to -47.4)	0.603 (0.375 to 0.877)	0.511 (0.33 to 0.734)	0.358 (0.232 to 0.517)	14.1 (0.523 to 30.5)	-47.7 (-53.9 to -40.2)
Ebola	0.00253 (0.00011 to 0.00636)	0.0000173 (0.000011 to 0.0000257)	0.000213 (0.000135 to 0.000315)	0 (0 to 0)	0 (0 to 0)	0.00263 (0.00105 to 0.00463)	0.0000361 (0.000023 to 0.0000535)	0.000433 (0.000276 to 0.000641)	0 (0 to 0)	0 (0 to 0)
Zika virus	0.000923 (0.000507 to 0.00157)	0.000949 (0.000515 to 0.00168)	0.00144 (0.000669 to 0.00287)	0 (0 to 0)	0 (0 to 0)	0.000574 (0.000324 to 0.00105)	0.000381 (0.000206 to 0.000707)	0.00021 (0.0000925 to 0.000485)	0 (0 to 0)	0 (0 to 0)
Guinea worm disease	0.00000965 (0.0000052 to 0.0000157)	0.00000715 (0.00000384 to 0.0000119)	0.00000409 (0.00000203 to 0.00000693)	-100 (-100 to -100)	-100 (-100 to -100)	0.00000713 (0.00000383 to 0.0000115)	0.00000481 (0.00000256 to 0.00000802)	0.00000313 (0.00000155 to 0.00000532)	-100 (-100 to -100)	-100 (-100 to -100)
Other neglected tropical diseases	6.73 (4.6 to 9.73)	6.68 (4.57 to 9.73)	5.85 (3.91 to 8.46)	68.1 (55.7 to 80.6)	-32 (-37.1 to -27)	9.11 (6.28 to 13)	7.28 (4.92 to 10.3)	5.06 (3.4 to 7.31)	70.8 (59.2 to 83.4)	-21.7 (-27 to -15.9)
Other infectious diseases	28.8 (20.2 to 39.9)	28.5 (19.9 to 40)	27.5 (18.5 to 40)	79.3 (67.8 to 91.4)	-27.5 (-32.2 to -22.6)	31.2 (22.1 to 43)	27.1 (18.6 to 38.1)	23.2 (15.2 to 35.7)	76.1 (68.5 to 84.1)	-19.3 (-22.8 to -15.6)

Supplementary Table 5. Cause-specific rate of YLDs for 70-79 years, 80-94 years, and 95+ years in 2019, and percentage change from 1990 to 2019 in YLD number and rate in 70+ years, global, for all GBD causes

	Males					Females				
	YLDs per 100,000, 2019			Percentage change in 70+, 1990-2019		YLDs per 100,000, 2019			Percentage change in 70+, 1990-2019	
Causes	70 to 79	80 to 94	95 plus	Number	Rate	70 to 79	80 to 94	95 plus	Number	Rate
Meningitis	4.46 (3.08 to 6.02)	5.15 (3.31 to 6.95)	6.12 (3.71 to 8.25)	7.12 (1.7 to 13.5)	-56.7 (-58.9 to -54.1)	5.01 (3.48 to 6.69)	4.8 (3.15 to 6.46)	4.71 (2.88 to 6.3)	15.2 (10.2 to 21.4)	-47.2 (-49.5 to -44.4)
Encephalitis	5.24 (3.83 to 6.88)	4.71 (3.42 to 6.19)	4.48 (3.27 to 5.83)	92.1 (78.7 to 108)	-22.3 (-27.8 to -16)	5.67 (4.15 to 7.47)	3.86 (2.8 to 5.05)	2.54 (1.8 to 3.39)	114 (99.9 to 128)	-2.12 (-8.35 to 4.69)
Diphtheria				0 (0 to 0)	0 (0 to 0)				0 (0 to 0)	0 (0 to 0)
Whooping cough				0 (0 to 0)	0 (0 to 0)				0 (0 to 0)	0 (0 to 0)
Tetanus	0.0178 (0.00959 to 0.0279)	0.0209 (0.0119 to 0.0349)	0.0316 (0.0148 to 0.0639)	-57.6 (-68.1 to -39.4)	-82.8 (-87.1 to -75.5)	0.0174 (0.00953 to 0.0274)	0.0165 (0.0088 to 0.0267)	0.0155 (0.00791 to 0.0268)	-55.8 (-69.1 to -37.6)	-79.8 (-85.8 to -71.4)
Measles				0 (0 to 0)	0 (0 to 0)				0 (0 to 0)	0 (0 to 0)
Varicella and herpes zoster	7.63 (4.1 to 12.3)	8.54 (4.48 to 14.4)	9.18 (4.34 to 17.6)	153 (145 to 163)	2.41 (-1.13 to 6.12)	7.94 (4.31 to 12.8)	8.8 (4.57 to 14.6)	9.67 (4.62 to 18.7)	120 (111 to 129)	0.827 (-3.06 to 4.8)
Acute hepatitis	3.02 (1.66 to 5.14)	2.43 (1.28 to 4.18)	1.89 (0.953 to 3.35)	130 (52.7 to 243)	-7.19 (-38.3 to 38.8)	1.95 (1.05 to 3.23)	1.64 (0.901 to 2.83)	1.54 (0.737 to 2.65)	99.1 (43.4 to 182)	-8.73 (-34.3 to 29.3)
Acute hepatitis A	0.206 (0.113 to 0.328)	0.0562 (0.0236 to 0.116)	0.00255 (0.000399 to 0.00834)	150 (106 to 203)	0.915 (-16.6 to 22.6)	0.18 (0.096 to 0.288)	0.042 (0.0163 to 0.0926)	0.00211 (0.000263 to 0.00753)	91.1 (54.8 to 136)	-12.4 (-29 to 8.29)
Acute hepatitis B	2.41 (1.17 to 4.48)	1.76 (0.793 to 3.25)	0.947 (0.343 to 2.07)	124 (36.8 to 270)	-9.54 (-44.7 to 49.7)	1.37 (0.636 to 2.55)	0.984 (0.417 to 1.92)	0.617 (0.19 to 1.4)	90.6 (19.5 to 214)	-12.6 (-45.2 to 44.1)
Acute hepatitis C	0.143 (0.0585 to 0.3)	0.215 (0.0882 to 0.44)	0.334 (0.118 to 0.744)	147 (132 to 166)	-0.191 (-6.23 to 7.58)	0.144 (0.0592 to 0.3)	0.2 (0.0814 to 0.408)	0.308 (0.108 to 0.705)	129 (118 to 147)	5.14 (-0.25 to 13)
Acute hepatitis E	0.255 (0.115 to 0.468)	0.401 (0.186 to 0.689)	0.605 (0.202 to 1.29)	158 (122 to 198)	4.4 (-10.4 to 20.3)	0.256 (0.118 to 0.479)	0.416 (0.201 to 0.739)	0.618 (0.208 to 1.26)	127 (95 to 165)	3.91 (-10.6 to 21.4)
Other unspecified infectious diseases	8.47 (5.76 to 12.3)	7.67 (5.18 to 11)	5.8 (3.85 to 8.49)	78 (66.2 to 89)	-28 (-32.8 to -23.6)	10.6 (7.25 to 14.9)	8.01 (5.5 to 11.4)	4.73 (3.09 to 6.83)	74.8 (63.6 to 86.9)	-19.9 (-25 to -14.3)
Maternal and neonatal disorders	42.4 (32.4 to 54.4)	9.42 (6.82 to 12.5)	5.07 (3.13 to 7.76)	436 (343 to 573)	117 (79.2 to 172)	74 (57.4 to 93.5)	17.6 (13.3 to 23.3)	5.14 (3.2 to 7.55)	334 (276 to 415)	99.1 (72.3 to 136)
Maternal disorders				0 (0 to 0)	0 (0 to 0)	1.33 (0.805 to 2.07)	0.628 (0.357 to 1)	0.208 (0.122 to 0.331)	-5.99 (-16.2 to 5.59)	-56.9 (-61.6 to -51.6)
Maternal hemorrhage				0 (0 to 0)	0 (0 to 0)				0 (0 to 0)	0 (0 to 0)
Maternal sepsis and other maternal infections				0 (0 to 0)	0 (0 to 0)				0 (0 to 0)	0 (0 to 0)
Maternal hypertensive disorders				0 (0 to 0)	0 (0 to 0)	0.0000000000000000000000<br>0000000000000000000000<br>0000000000000000000000083	2.92e-109 (5.89e-121 to 8.28e-111)	7.03e-177 (1.99e-191 to 1.26e-178)	88.6 (-86.5 to 2940)	-13.5 (-93.8 to 1290)
Maternal obstructed labor and uterine rupture				0 (0 to 0)	0 (0 to 0)	1.33 (0.805 to 2.07)	0.628 (0.357 to 1)	0.208 (0.122 to 0.331)	-5.99 (-16.2 to 5.59)	-56.9 (-61.6 to -51.6)
Maternal abortion and miscarriage				0 (0 to 0)	0 (0 to 0)				0 (0 to 0)	0 (0 to 0)
Ectopic pregnancy				0 (0 to 0)	0 (0 to 0)				0 (0 to 0)	0 (0 to 0)
Indirect maternal deaths										
Late maternal deaths										
Maternal deaths aggravated by HIV/AIDS										
Other maternal disorders				0 (0 to 0)	0 (0 to 0)				0 (0 to 0)	0 (0 to 0)
Neonatal disorders	42.4 (32.4 to 54.4)	9.42 (6.82 to 12.5)	5.07 (3.13 to 7.76)	436 (343 to 573)	117 (79.2 to 172)	72.7 (56.1 to 92)	16.9 (12.7 to 22.7)	4.93 (3.05 to 7.23)	369 (302 to 458)	115 (84.1 to 156)
Neonatal preterm birth	20 (15.5 to 26.2)	6.54 (4.48 to 9.12)	5.05 (3.1 to 7.75)	235 (201 to 276)	35.3 (21.5 to 52)	35.1 (27.6 to 44.2)	9.02 (6.61 to 11.9)	4.83 (2.98 to 7.14)	206 (184 to 229)	40.3 (30 to 50.7)
Neonatal encephalopathy due to birth asphyxia and trauma	4.77 (2.93 to 7.22)	0.309 (0.115 to 0.648)	0.00247 (0.000208 to 0.00966)	688 (384 to 1290)	219 (95.7 to 462)	7.16 (4.48 to 10.7)	0.897 (0.384 to 1.74)	0.0421 (0.00708 to 0.129)	404 (223 to 756)	131 (48.2 to 293)
Neonatal sepsis and other neonatal infections	15.9 (9.96 to 24.8)	2.29 (1.21 to 4.11)	0.0159 (0.00497 to 0.0386)	2280 (1270 to 4350)	861 (454 to 1700)	27.4 (17.8 to 41.4)	6.31 (3.78 to 10.6)	0.0573 (0.0179 to 0.148)	1730 (938 to 3990)	741 (376 to 1780)
Hemolytic disease and other neonatal jaundice	1.74 (1.29 to 2.2)	0.279 (0.198 to 0.381)	0.000513 (0.000202 to 0.00105)	254 (235 to 274)	43.2 (35.5 to 51.3)	3.02 (2.3 to 3.79)	0.713 (0.529 to 0.945)	0.00306 (0.00147 to 0.00548)	187 (173 to 202)	31.7 (25.3 to 38.3)
Other neonatal disorders				0 (0 to 0)	0 (0 to 0)				0 (0 to 0)	0 (0 to 0)
Nutritional deficiencies	366 (255 to 512)	349 (240 to 492)	268 (185 to 378)	84 (72.6 to 96.3)	-25.6 (-30.2 to -20.6)	449 (312 to 624)	354 (249 to 498)	221 (151 to 310)	77.6 (66.8 to 89)	-18.6 (-23.5 to -13.3)
Protein-energy malnutrition	57.1 (36.2 to 82.4)	60.2 (38.7 to 85)	56.3 (36.7 to 79.5)	263 (232 to 298)	46.8 (34.3 to 60.8)	38.7 (24.9 to 54.7)	41.1 (26.7 to 57.9)	43.8 (28.8 to 61.1)	197 (179 to 218)	36.3 (28.1 to 45.8)
Iodine deficiency	15.7 (9.1 to 26.1)	10.2 (5.79 to 16.9)	4.8 (2.56 to 8.2)	51.6 (33.5 to 67.3)	-38.7 (-46 to -32.3)	24.3 (13.3 to 42.9)	14.9 (8.16 to 25.9)	6.6 (3.44 to 11.6)	80.5 (57.1 to 98.5)	-17.2 (-28 to -8.98)
Vitamin A deficiency	2.27 (1.32 to 3.49)	1.5 (0.888 to 2.32)	0.584 (0.252 to 1.07)	174 (151 to 197)	10.6 (1.33 to 19.9)	1.89 (1.12 to 2.97)	1.15 (0.667 to 1.83)	0.388 (0.159 to 0.717)	176 (155 to 199)	26.6 (17 to 37)
Dietary iron deficiency	272 (185 to 392)	262 (179 to 380)	197 (127 to 288)	66.4 (53.7 to 78.8)	-32.7 (-37.9 to -27.7)	372 (257 to 523)	287 (196 to 409)	163 (106 to 240)	69.3 (57.5 to 81.8)	-22.4 (-27.8 to -16.7)
Other nutritional deficiencies	18.9 (11.9 to 27.2)	15.4 (9.9 to 21.7)	9.31 (6.07 to 13.1)	111 (92.4 to 131)	-14.7 (-22.2 to -6.43)	12.8 (8.22 to 18)	10.3 (6.69 to 14.6)	7.23 (4.76 to 10.1)	75.1 (64.6 to 87.1)	-19.7 (-24.5 to -14.2)
Non-communicable diseases	21600 (16400 to 27200)	27000 (20500 to 33900)	32700 (25100 to 40100)	151 (149 to 153)	1.62 (0.783 to 2.44)	23500 (17800 to 29300)	29000 (22100 to 36400)	34900 (26900 to 42500)	122 (121 to 124)	1.99 (1.21 to 2.88)
Neoplasms	747 (551 to 946)	950 (706 to 1210)	746 (540 to 938)	191 (169 to 215)	17.7 (8.7 to 27.5)	441 (330 to 567)	541 (391 to 687)	531 (384 to 673)	141 (125 to 158)	10.6 (2.93 to 18.1)
Lip and oral cavity cancer	11.8 (8.57 to 15.2)	9.91 (7.19 to 12.7)	5.3 (3.42 to 7.1)	154 (124 to 186)	2.88 (-9.28 to 15.4)	6.12 (4.47 to 7.91)	6.34 (4.36 to 8.38)	4.48 (2.81 to 6.17)	157 (135 to 182)	17.9 (7.73 to 29.1)
Nasopharynx cancer	4.48 (3.11 to 6.12)	5.18 (3.72 to 6.82)	1.32 (0.832 to 1.83)	312 (248 to 387)	66.4 (40.7 to 96.8)	1.46 (1.03 to 1.98)	2.12 (1.42 to 2.83)	0.864 (0.525 to 1.21)	158 (122 to 205)	18.1 (1.76 to 39.7)
Other pharynx cancer	4.1 (2.94 to 5.35)	3.17 (2.22 to 4.2)	1.86 (1.2 to 2.53)	253 (212 to 301)	42.7 (26.2 to 62.2)	1.18 (0.808 to 1.59)	1.02 (0.682 to 1.38)	0.825 (0.519 to 1.16)	214 (172 to 257)	43.8 (24.6 to 63.8)
Esophageal cancer	18.4 (12.7 to 24)	20.6 (14.7 to 26.5)	8.47 (5.45 to 11.2)	165 (127 to 211)	7.03 (-8.16 to 25.8)	7.09 (4.81 to 9.54)	8.74 (5.83 to 11.7)	5.58 (3.49 to 7.65)	88.3 (61.2 to 123)	-13.7 (-26.1 to 2.21)
Stomach cancer	41.4 (29.9 to 54.1)	50.8 (37.1 to 64.5)	28.8 (18.7 to 38.3)	125 (100 to 156)	-8.98 (-19.1 to 3.49)	15.9 (11.5 to 20.7)	23.9 (16.5 to 31.3)	24.4 (15.5 to 33.2)	59.9 (43.3 to 77.5)	-26.7 (-34.3 to -18.6)
Colon and rectum cancer	112 (82.3 to 143)	145 (106 to 185)	128 (91.8 to 167)	233 (207 to 263)	34.6 (24 to 46.7)	69.3 (51.4 to 90.1)	96.4 (68.5 to 124)	108 (75.1 to 142)	138 (118 to 158)	9.3 (-0.0718 to 18.5)
Liver cancer	12.8 (9.08 to 16.9)	16.6 (11.6 to 22)	9.14 (5.74 to 12.6)	175 (139 to 219)	11 (-3.3 to 28.9)	5.82 (4.07 to 7.82)	8.44 (5.61 to 11.5)	6.83 (4.27 to 9.5)	114 (81.1 to 146)	-2.06 (-17 to 12.8)
Liver cancer due to hepatitis B	4.19 (2.74 to 5.9)	3.92 (2.5 to 5.6)	1.35 (0.713 to 2.24)	110 (68.6 to 160)	-15.2 (-31.9 to 5.23)	1.07 (0.654 to 1.6)	1.04 (0.594 to 1.6)	0.534 (0.249 to 0.957)	59.4 (26.9 to 95)	-26.9 (-41.8 to -10.6)
Liver cancer due to hepatitis C	3.93 (2.66 to 5.41)	7.41 (5.13 to 10)	5.45 (3.3 to 7.77)	223 (185 to 272)	30.7 (15.3 to 50.5)	2.85 (1.91 to 3.96)	5.1 (3.34 to 7.14)	4.91 (2.94 to 6.91)	125 (91.9 to 161)	3.36 (-12 to 19.7)
Liver cancer due to alcohol use	3.48 (2.25 to 5.06)	3.55 (2.25 to 5.18)	1.44 (0.768 to 2.41)	211 (179 to 249)	25.9 (12.8 to 41.2)	0.813 (0.506 to 1.24)	0.796 (0.481 to 1.22)	0.382 (0.186 to 0.668)	125 (93.6 to 158)	3.15 (-11.2 to 18.2)
Liver cancer due to NASH	0.831 (0.511 to 1.26)	1.22 (0.747 to 1.83)	0.663 (0.328 to 1.15)	240 (197 to 290)	37.4 (20.2 to 57.6)	0.724 (0.451 to 1.07)	1.07 (0.627 to 1.61)	0.716 (0.343 to 1.2)	170 (127 to 214)	23.6 (3.92 to 43.8)
Liver cancer due to other causes	0.416 (0.258 to 0.622)	0.521 (0.321 to 0.793)	0.241 (0.123 to 0.421)	141 (102 to 185)	-2.56 (-18.1 to 15.4)	0.363 (0.229 to 0.542)	0.446 (0.264 to 0.691)	0.292 (0.146 to 0.503)	89.8 (55.7 to 125)	-13 (-28.6 to 2.99)
Gallbladder and biliary tract cancer	4.27 (2.89 to 5.7)	8.07 (5.37 to 10.7)	6.61 (3.85 to 9.22)	161 (108 to 193)	5.44 (-16.1 to 18.5)	4.4 (2.99 to 5.94)	7.78 (5.11 to 10.6)	9.71 (5.3 to 13.8)	87.1 (58.3 to 115)	-14.2 (-27.4 to -1.58)
Pancreatic cancer	10.2 (7.15 to 13.3)	15.1 (10.7 to 19.6)	12.5 (8.05 to 17)	232 (200 to 267)	34.3 (21.4 to 48.5)	8.47 (5.93 to 11.2)	14 (9.39 to 18.6)	14.8 (9.32 to 20.2)	192 (164 to 219)	33.7 (21.2 to 46.3)
Larynx cancer	13.6 (9.97 to 17.5)	12.2 (9.1 to 15.7)	6.08 (4.14 to 8.06)	131 (113 to 152)	-6.66 (-13.9 to 1.97)	2.25 (1.62 to 3.03)	1.98 (1.38 to 2.67)	0.944 (0.626 to 1.29)	118 (101 to 140)	-0.086 (-7.66 to 9.86)
Tracheal, bronchus, and lung cancer	71.8 (52.7 to 91.4)	85.1 (61.1 to 108)	50.2 (33.2 to 65.6)	169 (139 to 198)	8.74 (-3.23 to 20.6)	29.1 (20.7 to 37.1)	33.6 (23.2 to 44)	28.9 (18.2 to 39.2)	221 (187 to 255)	47.3 (31.6 to 62.7)
Malignant skin melanoma	10.1 (4.72 to 14.5)	14.9 (7.09 to 20.1)	11.6 (5.66 to 16.4)	400 (143 to 515)	102 (-1.82 to 149)	6.23 (3.53 to 8.99)	8.63 (4.8 to 12)	8.03 (4.11 to 11.4)	248 (126 to 302)	59.5 (3.79 to 84.4)
Non-melanoma skin cancer	18.5 (11.7 to 27.2)	32.9 (21.9 to 48.4)	82.2 (53.5 to 120)	224 (168 to 296)	31.2 (8.54 to 60)	7.79 (4.97 to 11.3)	14.3 (9.46 to 20.8)	42.9 (28.5 to 63.2)	113 (80.6 to 158)	-2.29 (-17.2 to 18.1)
Non-melanoma skin cancer (squamous-cell carcinoma)	18.3 (11.6 to 27.1)	32.6 (21.7 to 48)	81.5 (53.1 to 119)	224 (168 to 296)	31 (8.35 to 60.1)	7.68 (4.88 to 11.2)	14.1 (9.36 to 20.7)	42.6 (28.3 to 62.8)	113 (79.7 to 157)	-2.56 (-17.6 to 18)

Supplementary Table 5. Cause-specific rate of YLDs for 70-79 years, 80-94 years, and 95+ years in 2019, and percentage change from 1990 to 2019 in YLD number and rate in 70+ years, global, for all GBD causes

	Males					Females				
	YLDs per 100,000, 2019			Percentage change in 70+, 1990-2019		YLDs per 100,000, 2019			Percentage change in 70+, 1990-2019	
Causes	70 to 79	80 to 94	95 plus	Number	Rate	70 to 79	80 to 94	95 plus	Number	Rate
Non-melanoma skin cancer (basal-cell carcinoma)	0.189 (0.0857 to 0.353)	0.298 (0.14 to 0.552)	0.677 (0.301 to 1.33)	290 (251 to 341)	57.5 (41.9 to 78.3)	0.108 (0.0496 to 0.206)	0.153 (0.0707 to 0.288)	0.282 (0.12 to 0.563)	176 (150 to 208)	26.4 (14.4 to 41)
Breast cancer	2.89 (2.07 to 3.87)	3.73 (2.69 to 4.94)	2.83 (1.96 to 3.82)	197 (162 to 235)	20 (5.89 to 35.3)	128 (90.2 to 172)	143 (104 to 191)	134 (97.1 to 180)	125 (108 to 144)	3.33 (-4.53 to 11.9)
Cervical cancer				0 (0 to 0)	0 (0 to 0)	9.16 (6.51 to 12)	6.87 (4.63 to 9.14)	5.06 (3.12 to 6.94)	86.7 (63.6 to 107)	-14.4 (-25 to -5.03)
Uterine cancer				0 (0 to 0)	0 (0 to 0)	23 (16.7 to 30.1)	15.8 (11.2 to 20.3)	6.32 (4.02 to 8.71)	150 (128 to 174)	14.4 (4.48 to 25.7)
Ovarian cancer				0 (0 to 0)	0 (0 to 0)	12.3 (8.84 to 16)	12.5 (8.62 to 16.6)	6.49 (3.96 to 8.85)	117 (90.3 to 143)	-0.401 (-12.7 to 11.3)
Prostate cancer	224 (159 to 315)	276 (199 to 363)	189 (135 to 244)	160 (133 to 220)	5.28 (-5.91 to 29.2)				0 (0 to 0)	0 (0 to 0)
Testicular cancer	0.91 (0.635 to 1.22)	0.629 (0.419 to 0.855)	0.535 (0.339 to 0.752)	244 (197 to 318)	39.2 (20 to 68.8)				0 (0 to 0)	0 (0 to 0)
Kidney cancer	13.3 (9.74 to 17.1)	12.5 (8.98 to 16.1)	7.91 (5.17 to 10.7)	275 (239 to 313)	51.7 (36.9 to 67.1)	6.84 (4.97 to 8.82)	6.3 (4.38 to 8.27)	4.43 (2.72 to 6.11)	175 (150 to 205)	26 (14.4 to 39.7)
Bladder cancer	41.2 (30.2 to 54)	60.6 (43.9 to 77.9)	55.5 (38.3 to 72.7)	194 (169 to 219)	18.7 (8.85 to 29.1)	9.68 (7.02 to 12.8)	14.7 (10.3 to 19.4)	16.5 (11 to 22.1)	134 (113 to 156)	7.19 (-2.4 to 17.5)
Brain and central nervous system cancer	5.8 (3.42 to 7.87)	7.06 (3.88 to 9.83)	2.21 (1.19 to 3.18)	274 (110 to 359)	51.3 (-15.1 to 85.5)	4.81 (2.77 to 6.7)	6 (3.12 to 8.81)	2.18 (1.07 to 3.3)	246 (81.5 to 349)	58.4 (-16.8 to 106)
Thyroid cancer	3.12 (2.2 to 4.09)	2.5 (1.67 to 3.3)	2.52 (1.5 to 3.46)	291 (236 to 338)	58 (35.8 to 76.9)	4.77 (3.41 to 6.29)	3.12 (2.13 to 4.15)	3.99 (2.42 to 5.5)	167 (141 to 195)	22.4 (10.4 to 35.3)
Mesothelioma	2.15 (1.5 to 2.85)	2.89 (2.01 to 3.83)	1.27 (0.81 to 1.73)	145 (113 to 180)	-0.968 (-13.7 to 13.2)	0.451 (0.285 to 0.613)	0.527 (0.314 to 0.733)	0.319 (0.176 to 0.458)	74.9 (26.3 to 117)	-19.8 (-42.1 to -0.488)
Hodgkin lymphoma	2.15 (1.43 to 2.86)	6.08 (3.92 to 8.16)	3.58 (2.09 to 4.94)	217 (155 to 259)	28.2 (3.2 to 45.2)	1.25 (0.836 to 1.73)	3.03 (1.79 to 4.26)	2.36 (1.14 to 3.67)	204 (163 to 241)	39.2 (20.7 to 56.3)
Non-Hodgkin lymphoma	33.7 (23 to 44.8)	46.4 (30.9 to 63.4)	29.6 (18.5 to 40.6)	340 (296 to 387)	78 (60.1 to 97)	19.7 (13.5 to 26.9)	29.4 (19.3 to 41.7)	23.7 (14.6 to 33.3)	235 (197 to 273)	53.4 (36.4 to 71.2)
Multiple myeloma	10.5 (7.23 to 13.9)	14.2 (9.24 to 18.8)	6.43 (4.12 to 8.68)	243 (178 to 289)	38.7 (12.4 to 57.1)	7.39 (5.06 to 9.85)	9.12 (5.76 to 12.4)	3.63 (1.95 to 5.15)	169 (121 to 207)	23.2 (1.53 to 40.8)
Leukemia	24.5 (17.3 to 31.9)	36.1 (25.6 to 46.7)	35 (22.3 to 48)	206 (175 to 244)	23.8 (11.3 to 39)	16 (11.5 to 21)	22.6 (15.5 to 29.7)	23.6 (14.3 to 32.4)	162 (136 to 191)	20.3 (8.07 to 33.4)
Acute lymphoid leukemia	3.52 (2.28 to 4.87)	2.88 (1.89 to 3.95)	1.1 (0.646 to 1.58)	620 (487 to 755)	191 (137 to 246)	2.62 (1.66 to 3.66)	1.96 (1.25 to 2.73)	0.693 (0.384 to 1)	482 (370 to 599)	167 (116 to 220)
Chronic lymphoid leukemia	10.5 (7.36 to 14)	11.9 (8.18 to 15.7)	7.83 (4.96 to 10.9)	214 (174 to 291)	26.8 (10.7 to 58)	6.07 (4.31 to 8.06)	7.31 (5.04 to 9.78)	5.01 (3.1 to 6.86)	157 (127 to 199)	18 (3.95 to 37.3)
Acute myeloid leukemia	2.54 (1.69 to 3.47)	4.47 (2.87 to 6.15)	16.1 (8.65 to 23.4)	245 (158 to 311)	39.3 (4.29 to 66.1)	1.65 (1.09 to 2.24)	2.64 (1.65 to 3.63)	10.7 (5.47 to 15.7)	198 (125 to 246)	36.7 (3.09 to 58.8)
Chronic myeloid leukemia	2.1 (1.42 to 2.9)	4.85 (3.27 to 6.74)	3.16 (1.9 to 4.49)	273 (209 to 378)	50.9 (25 to 93.3)	1.44 (0.987 to 1.98)	3.58 (2.43 to 4.91)	2.7 (1.59 to 3.9)	277 (210 to 367)	72.9 (42.1 to 114)
Other leukemia	5.77 (4.04 to 7.55)	12.1 (8.51 to 15.5)	6.78 (4.34 to 9.27)	118 (91.3 to 156)	-11.7 (-22.7 to 3.41)	4.24 (2.91 to 5.72)	7.07 (4.8 to 9.38)	4.47 (2.69 to 6.22)	87.1 (63.9 to 113)	-14.2 (-24.9 to -2.43)
Other malignant neoplasms	26 (18 to 34.6)	33.6 (22.8 to 44.5)	34.1 (21.5 to 45.7)	296 (237 to 353)	60.2 (36.3 to 83.2)	16.8 (12.2 to 22.1)	22.5 (15.3 to 29.9)	29 (18 to 39.5)	228 (186 to 265)	50.2 (31.2 to 67.1)
Other neoplasms	24.7 (15.3 to 36.8)	29.3 (18.6 to 43.6)	23.5 (14.6 to 36.6)	202 (187 to 222)	22 (16.1 to 30.2)	15.7 (9.88 to 23.3)	17.9 (11.6 to 26.5)	12.7 (7.92 to 19.8)	147 (135 to 163)	13.5 (7.92 to 20.5)
Myelodysplastic, myeloproliferative, and other hematopoietic neoplasms	24.7 (15.3 to 36.8)	29.3 (18.6 to 43.6)	23.5 (14.6 to 36.6)	202 (187 to 222)	22 (16.1 to 30.2)	15.7 (9.88 to 23.3)	17.9 (11.6 to 26.5)	12.7 (7.92 to 19.8)	147 (135 to 163)	13.5 (7.92 to 20.5)
Benign and in situ intestinal neoplasms				0 (0 to 0)	0 (0 to 0)				0 (0 to 0)	0 (0 to 0)
Benign and in situ cervical and uterine neoplasms				0 (0 to 0)	0 (0 to 0)				0 (0 to 0)	0 (0 to 0)
Other benign and in situ neoplasms				0 (0 to 0)	0 (0 to 0)				0 (0 to 0)	0 (0 to 0)
Cardiovascular diseases	2520 (1810 to 3260)	3490 (2550 to 4540)	3900 (2790 to 5170)	140 (135 to 145)	-2.98 (-4.91 to -1.01)	2640 (1920 to 3370)	3890 (2900 to 4970)	4590 (3340 to 5950)	113 (109 to 117)	-2.3 (-4.25 to -0.353)
Rheumatic heart disease	17.5 (11.7 to 24.4)	19.9 (13.7 to 27.5)	31 (20.5 to 43.8)	139 (127 to 153)	-3.2 (-8.31 to 2.46)	24.1 (16.2 to 33.5)	25.6 (17.4 to 35.1)	28.5 (18.9 to 40)	94.1 (82.3 to 108)	-11 (-16.4 to -4.61)
Ischemic heart disease	554 (357 to 788)	813 (530 to 1190)	1060 (589 to 1670)	125 (117 to 133)	-9.18 (-12.3 to -5.81)	413 (267 to 588)	661 (434 to 961)	886 (493 to 1430)	105 (97.1 to 115)	-5.81 (-9.65 to -1.53)
Stroke	1100 (780 to 1440)	1330 (944 to 1770)	1050 (710 to 1450)	151 (143 to 159)	1.48 (-1.96 to 4.88)	1440 (1020 to 1860)	1920 (1370 to 2480)	1970 (1360 to 2680)	115 (108 to 122)	-1.65 (-4.75 to 1.65)
Ischemic stroke	937 (652 to 1260)	1210 (849 to 1620)	896 (592 to 1280)	160 (150 to 170)	5.12 (1.21 to 9.23)	1240 (859 to 1620)	1710 (1220 to 2240)	1670 (1120 to 2320)	119 (111 to 127)	0.241 (-3.3 to 4.05)
Intracerebral hemorrhage	129 (87.5 to 179)	88.9 (60.1 to 120)	77.5 (49 to 113)	95.4 (88.7 to 103)	-21 (-23.7 to -18.1)	122 (83.8 to 166)	112 (76.2 to 150)	128 (79.6 to 190)	68.6 (62.9 to 74.7)	-22.7 (-25.3 to -19.9)
Subarachnoid hemorrhage	31.5 (19.3 to 45.8)	39.2 (24.9 to 57)	74.1 (44.4 to 111)	140 (129 to 152)	-3.14 (-7.32 to 2.07)	76.3 (49.1 to 109)	93.9 (60.8 to 134)	169 (105 to 248)	131 (120 to 143)	5.87 (1.03 to 11.4)
Hypertensive heart disease	163 (86.2 to 277)	257 (142 to 423)	331 (141 to 679)	150 (139 to 164)	1.14 (-3.28 to 6.77)	148 (78.4 to 245)	280 (159 to 449)	439 (187 to 866)	154 (141 to 170)	16.5 (10.6 to 24)
Non-rheumatic valvular heart disease	28.7 (16 to 48.1)	63.2 (36.6 to 103)	167 (106 to 234)	191 (169 to 209)	17.7 (8.87 to 25.1)	36.7 (20.6 to 61.3)	85.4 (49.5 to 138)	219 (139 to 310)	132 (115 to 148)	6.57 (-1.62 to 13.9)
Non-rheumatic calcific aortic valve disease	11.1 (6.14 to 19)	27.1 (15.2 to 45.3)	61.2 (35.5 to 92.3)	498 (448 to 564)	142 (121 to 168)	10.3 (5.64 to 17.4)	27.8 (15.8 to 47.1)	58.4 (33.9 to 88.9)	458 (405 to 529)	156 (131 to 188)
Non-rheumatic degenerative mitral valve disease	17.4 (9.58 to 29.1)	35.7 (20.3 to 57.1)	105 (65.8 to 150)	115 (97.4 to 130)	-13 (-20.2 to -7.06)	26.2 (14.5 to 44.3)	57 (32.3 to 91.8)	160 (99.3 to 231)	85 (70 to 98)	-15.2 (-22.1 to -9.21)
Other non-rheumatic valve diseases	0.192 (0.102 to 0.324)	0.379 (0.21 to 0.621)	0.104 (0.0447 to 0.204)	170 (156 to 187)	9.18 (3.57 to 15.8)	0.28 (0.148 to 0.472)	0.592 (0.34 to 0.949)	0.383 (0.162 to 0.761)	161 (144 to 179)	19.8 (11.7 to 28.1)
Cardiomyopathy and myocarditis	40 (22.3 to 66.3)	77.4 (47.4 to 121)	155 (74.3 to 293)	116 (102 to 137)	-12.5 (-18.4 to -4.31)	26.1 (14.2 to 43.2)	59.1 (35.5 to 91.9)	122 (56.6 to 235)	89.7 (75.4 to 108)	-13 (-19.6 to -4.78)
Myocarditis	2.69 (1.55 to 4.24)	5.36 (3.37 to 8.05)	11.1 (5.51 to 19.9)	155 (142 to 172)	3.23 (-2.27 to 10)	2.07 (1.18 to 3.35)	5.79 (3.63 to 8.89)	13.7 (6.56 to 25.5)	137 (121 to 155)	8.5 (1.1 to 17.1)
Alcoholic cardiomyopathy	6.74 (3.85 to 11.3)	4.2 (2.6 to 6.73)	4.25 (1.96 to 8.23)	78.1 (61.8 to 101)	-28 (-34.6 to -18.8)	1.87 (1.03 to 3.22)	1.6 (0.937 to 2.58)	1.76 (0.764 to 3.48)	36.8 (24.2 to 54.3)	-37.3 (-43.1 to -29.3)
Other cardiomyopathy	30.6 (16.8 to 50.9)	67.9 (41.2 to 106)	140 (66.3 to 265)	121 (106 to 141)	-10.8 (-16.8 to -2.59)	22.1 (12 to 36.7)	51.7 (30.9 to 80.4)	106 (49 to 205)	89.7 (75.5 to 108)	-13 (-19.5 to -4.58)
Atrial fibrillation and flutter	513 (320 to 773)	794 (504 to 1160)	933 (615 to 1340)	139 (134 to 147)	-3.3 (-5.37 to -0.309)	433 (270 to 661)	695 (439 to 1020)	720 (471 to 1040)	115 (111 to 122)	-1.25 (-3.5 to 1.84)
Aortic aneurysm										
Peripheral artery disease	42 (19.2 to 77)	65.3 (29.2 to 123)	106 (41.9 to 224)	82.6 (77.6 to 88.7)	-26.2 (-28.2 to -23.7)	73.3 (33.6 to 135)	111 (50.2 to 210)	139 (54.9 to 295)	68.2 (63.1 to 73.9)	-22.9 (-25.2 to -20.3)
Endocarditis	2.48 (1.49 to 3.8)	7.26 (4.65 to 10.7)	29 (13.8 to 53.5)	157 (133 to 186)	3.71 (-5.94 to 15.8)	2.58 (1.56 to 4)	8.7 (5.47 to 12.8)	33.8 (16.1 to 63.1)	113 (91.4 to 140)	-2.56 (-12.2 to 9.84)
Other cardiovascular and circulatory diseases	62.9 (36.4 to 96.9)	62.4 (36.3 to 97.6)	46.4 (22.7 to 85.1)	140 (126 to 157)	-3.17 (-8.44 to 3.92)	50.1 (28.4 to 78.9)	44 (26.5 to 68.1)	35.4 (18 to 64.7)	99.3 (86.4 to 116)	-8.65 (-14.5 to -1.06)
Chronic respiratory diseases	1770 (1410 to 2090)	2660 (2160 to 3100)	3970 (3210 to 4620)	125 (116 to 133)	-9.05 (-12.6 to -5.61)	1670 (1360 to 1930)	2440 (1990 to 2820)	3480 (2810 to 4100)	116 (107 to 125)	-0.953 (-5.3 to 3)
Chronic obstructive pulmonary disease	1480 (1190 to 1730)	2350 (1880 to 2750)	3600 (2910 to 4230)	145 (139 to 151)	-1.03 (-3.57 to 1.6)	1410 (1150 to 1650)	2190 (1760 to 2560)	3100 (2450 to 3710)	138 (132 to 144)	9.09 (6.41 to 11.7)
Pneumoconiosis	50.9 (34.1 to 71.8)	48.2 (33.4 to 67)	22.7 (15.5 to 31.2)	145 (127 to 163)	-0.944 (-8.42 to 6.44)	4.61 (3.06 to 6.75)	6.23 (4.1 to 9.09)	6.52 (4.15 to 9.64)	111 (97.1 to 126)	-3.41 (-9.63 to 3.59)
Silicosis	42.8 (28.1 to 61.2)	37.2 (25.6 to 52.6)	12.7 (8.54 to 17.7)	154 (131 to 178)	2.88 (-6.61 to 12.3)	2.42 (1.45 to 3.79)	3.12 (1.96 to 4.81)	2.94 (1.75 to 4.59)	120 (94.5 to 147)	0.982 (-10.8 to 13.1)
Asbestosis	1.67 (1.06 to 2.49)	2.69 (1.74 to 3.92)	2.38 (1.16 to 4.15)	192 (151 to 244)	18.2 (1.37 to 39.1)	0.47 (0.251 to 0.788)	0.527 (0.293 to 0.845)	0.52 (0.214 to 1.04)	140 (108 to 178)	10.1 (-4.5 to 27.3)
Coal workers pneumoconiosis	1.87 (1.16 to 2.81)	1.64 (1.03 to 2.42)	0.163 (0.0837 to 0.288)	47.4 (34.7 to 62.3)	-40.4 (-45.6 to -34.4)	0.347 (0.197 to 0.539)	0.395 (0.231 to 0.623)	0.368 (0.195 to 0.692)	71.6 (40.6 to 117)	-21.3 (-35.6 to -0.426)
Other pneumoconiosis	4.55 (2.84 to 6.72)	6.69 (4.21 to 9.69)	7.49 (4.71 to 11.2)	118 (98.7 to 138)	-12 (-19.7 to -3.7)	1.37 (0.828 to 2.15)	2.19 (1.32 to 3.44)	2.7 (1.54 to 4.51)	99.6 (81.7 to 120)	-8.51 (-16.7 to 0.779)
Asthma	198 (120 to 298)	205 (133 to 295)	273 (172 to 410)	25.3 (16.4 to 34.2)	-49.3 (-52.9 to -45.7)	210 (132 to 310)	208 (135 to 294)	320 (202 to 474)	21 (16.3 to 26.8)	-44.5 (-46.7 to -41.9)
Interstitial lung disease and pulmonary sarcoidosis	40.1 (24.9 to 61.5)	45 (28.9 to 66.8)	46.6 (29.3 to 72.2)	220 (200 to 241)	29.4 (21.4 to 37.9)	33.1 (20.5 to 50.7)	32.2 (21 to 47)	32.6 (20.5 to 48.9)	177 (162 to 195)	27 (19.9 to 35.3)
Other chronic respiratory diseases	6.96 (5.59 to 8.15)	10.6 (8.59 to 12.4)	24.2 (19.6 to 28.5)	219 (212 to 227)	29.1 (26.2 to 32.1)	6.43 (5.2 to 7.52)	9.82 (7.94 to 11.5)	20.7 (16.4 to 24.8)	202 (196 to 209)	38.6 (35.6 to 41.6)

Supplementary Table 5. Cause-specific rate of YLDs for 70-79 years, 80-94 years, and 95+ years in 2019, and percentage change from 1990 to 2019 in YLD number and rate in 70+ years, global, for all GBD causes

Causes	Males					Females				
	YLDs per 100,000, 2019			Percentage change in 70+, 1990-2019		YLDs per 100,000, 2019			Percentage change in 70+, 1990-2019	
	70 to 79	80 to 94	95 plus	Number	Rate	70 to 79	80 to 94	95 plus	Number	Rate
Digestive diseases	464 (323 to 629)	436 (308 to 597)	421 (297 to 576)	111 (105 to 117)	-14.7 (-17.1 to -12.2)	457 (314 to 634)	421 (293 to 595)	424 (294 to 594)	88.7 (83 to 94.3)	-13.5 (-16.1 to -10.9)
Cirrhosis and other chronic liver diseases	24.5 (15.7 to 35.8)	20.7 (13.6 to 31.1)	10.4 (6.31 to 15.8)	129 (121 to 137)	-7.6 (-10.8 to -4.21)	18.7 (12.1 to 27.4)	15 (9.8 to 22.5)	8.09 (4.98 to 12.1)	95.5 (88.5 to 103)	-10.4 (-13.6 to -6.98)
Cirrhosis and other chronic liver diseases due to hepatitis B	5.25 (3.06 to 8.14)	4.34 (2.55 to 6.85)	1.56 (0.734 to 2.84)	77.7 (63.7 to 92.4)	-28.2 (-33.8 to -22.2)	3.33 (1.98 to 5.28)	2.5 (1.45 to 4.15)	1.12 (0.518 to 2.1)	46.7 (33.3 to 59.5)	-32.8 (-38.9 to -26.9)
Cirrhosis and other chronic liver diseases due to hepatitis C	6.67 (4.05 to 10.3)	5.9 (3.52 to 9.04)	3.51 (1.89 to 5.58)	150 (135 to 166)	0.941 (-4.89 to 7.34)	4.62 (2.76 to 7.33)	3.83 (2.24 to 6.06)	2.35 (1.2 to 3.84)	112 (98.6 to 125)	-3.01 (-8.95 to 3.08)
Cirrhosis and other chronic liver diseases due to alcohol use	7.57 (4.55 to 11.5)	5.68 (3.45 to 8.89)	2.65 (1.27 to 4.82)	151 (139 to 165)	1.58 (-3.53 to 7.04)	3.68 (2.06 to 5.79)	2.6 (1.47 to 4.13)	1.33 (0.579 to 2.62)	117 (103 to 132)	-0.615 (-7.03 to 6.42)
Cirrhosis and other chronic liver diseases due to NAFLD	2.08 (0.959 to 3.75)	1.88 (0.932 to 3.39)	1.04 (0.349 to 2.34)	144 (126 to 164)	-1.37 (-8.56 to 6.87)	2.62 (1.26 to 4.51)	2.13 (1.08 to 3.69)	1.16 (0.408 to 2.5)	114 (99.9 to 131)	-1.78 (-8.38 to 5.71)
Cirrhosis and other chronic liver diseases due to other causes	2.92 (1.45 to 5.34)	2.9 (1.42 to 5.1)	1.67 (0.633 to 3.41)	138 (120 to 157)	-3.72 (-11.1 to 3.72)	4.44 (2.37 to 7.7)	3.92 (1.99 to 6.49)	2.12 (0.899 to 3.91)	102 (90 to 117)	-7.3 (-12.9 to -0.723)
Upper digestive system diseases	193 (116 to 309)	175 (102 to 283)	149 (81.3 to 240)	122 (112 to 129)	-10.2 (-14.3 to -7.26)	219 (132 to 346)	183 (109 to 292)	142 (80.8 to 227)	103 (97 to 108)	-7 (-9.67 to -4.82)
Peptic ulcer disease	16.7 (10.2 to 26.1)	18.5 (11.8 to 27.1)	14.6 (8.95 to 22.1)	48.5 (41.5 to 56.4)	-40 (-42.8 to -36.8)	16.3 (9.95 to 25.2)	18.5 (11.9 to 27.3)	14.8 (9.11 to 23.2)	53 (47.4 to 58.9)	-29.8 (-32.4 to -27.1)
Gastritis and duodenitis	41.1 (24 to 64.6)	31.7 (18.3 to 49.6)	20.2 (12.2 to 32)	108 (93.3 to 126)	-16.1 (-21.9 to -8.66)	59.2 (34.1 to 93.6)	39.4 (23 to 60.5)	24.4 (14.7 to 37.7)	99.9 (87.7 to 113)	-8.34 (-14 to -2.41)
Gastroesophageal reflux disease	135 (70.4 to 244)	125 (62.8 to 220)	115 (55.7 to 202)	143 (138 to 148)	-1.95 (-3.62 to 0.0802)	143 (74.2 to 256)	125 (63.2 to 221)	103 (50.2 to 181)	113 (109 to 117)	-2.49 (-4.27 to -0.318)
Appendicitis	0.802 (0.476 to 1.22)	0.471 (0.311 to 0.67)	0.404 (0.248 to 0.584)	250 (218 to 286)	41.3 (28.7 to 56.1)	0.758 (0.451 to 1.18)	0.408 (0.268 to 0.586)	0.327 (0.203 to 0.473)	239 (212 to 270)	55.5 (42.9 to 69.7)
Paralytic ileus and intestinal obstruction	5.58 (3.7 to 7.51)	7.96 (5.48 to 10.5)	8.96 (6.41 to 11.9)	187 (169 to 208)	16.2 (8.81 to 24.4)	4.61 (3.12 to 6.17)	6.64 (4.64 to 8.71)	7.8 (5.48 to 10.3)	131 (116 to 148)	6 (-0.992 to 13.7)
Inguinal, femoral, and abdominal hernia	91.5 (59.7 to 134)	56 (38.5 to 77.8)	30.7 (21.3 to 42.1)	89.6 (79.7 to 99.9)	-23.4 (-27.3 to -19.2)	16.6 (10.7 to 24.1)	10.4 (7.11 to 14.5)	5.88 (4 to 8.32)	50.9 (41.9 to 60.5)	-30.8 (-34.9 to -26.4)
Inflammatory bowel disease	18.2 (12 to 25.6)	14.2 (9.42 to 20.1)	13.2 (8.61 to 19.5)	77.4 (69.5 to 85.6)	-28.3 (-31.5 to -25)	17.1 (11.4 to 23.8)	12.8 (8.37 to 18)	10.9 (7.17 to 16.2)	43.7 (37.3 to 50.7)	-34.1 (-37.1 to -30.9)
Vascular intestinal disorders	3.78 (2.54 to 5.24)	5.73 (3.86 to 7.64)	4.69 (3.06 to 6.5)	119 (105 to 134)	-11.4 (-17 to -5.4)	3.76 (2.47 to 5.17)	6.26 (4.31 to 8.29)	5.01 (3.28 to 6.83)	86.3 (74.5 to 99.4)	-14.6 (-20 to -8.6)
Gallbladder and biliary diseases	104 (61.6 to 161)	125 (79.9 to 196)	160 (100 to 248)	103 (94.1 to 115)	-17.7 (-21.5 to -13.2)	156 (92.4 to 241)	156 (99.9 to 241)	198 (122 to 307)	76.7 (69.6 to 84.1)	-19 (-22.2 to -15.6)
Pancreatitis	7.91 (4.36 to 12.8)	7.82 (4.5 to 12.2)	7.16 (4.23 to 11.4)	160 (145 to 175)	5.27 (-0.871 to 11.1)	7.6 (4.19 to 12.1)	8.35 (4.85 to 13)	8.1 (4.87 to 12.8)	113 (100 to 125)	-2.17 (-8.2 to 3.09)
Other digestive diseases	14.6 (10.2 to 19.8)	22.5 (15.9 to 30.3)	36.3 (25.4 to 50.6)	136 (130 to 142)	-4.78 (-7.1 to -2.17)	14 (9.67 to 19.6)	22.1 (15.2 to 30.7)	38 (25.9 to 54.5)	114 (108 to 120)	-1.87 (-4.61 to 0.955)
Neurological disorders	1180 (796 to 1610)	2890 (1990 to 3910)	6370 (4340 to 8550)	186 (177 to 195)	15.6 (11.9 to 19.4)	1460 (966 to 2130)	3700 (2570 to 5030)	8020 (5610 to 10600)	148 (141 to 155)	13.5 (10.3 to 16.8)
Alzheimer's disease and other dementias	545 (363 to 773)	2220 (1490 to 3080)	5790 (3920 to 7910)	200 (191 to 208)	21.2 (17.7 to 24.6)	701 (479 to 995)	3020 (2070 to 4150)	7490 (5140 to 10100)	161 (155 to 168)	19.9 (16.8 to 23)
Parkinson's disease	156 (106 to 214)	257 (178 to 343)	217 (139 to 324)	199 (188 to 211)	20.7 (16.4 to 25.7)	106 (71.5 to 146)	171 (119 to 228)	148 (94.7 to 216)	136 (128 to 144)	8.05 (4.65 to 11.9)
Idiopathic epilepsy	138 (82.7 to 210)	177 (104 to 273)	221 (123 to 358)	168 (119 to 230)	8.34 (-11.5 to 33.2)	126 (75.8 to 192)	148 (86.9 to 232)	172 (94.6 to 283)	138 (97.3 to 187)	9.09 (-9.57 to 31.6)
Multiple sclerosis	7.46 (5.3 to 9.78)	6.81 (4.84 to 8.98)	6.36 (4.39 to 8.53)	141 (127 to 157)	-2.62 (-8.4 to 3.95)	14.8 (10.7 to 19.1)	13.6 (9.85 to 17.7)	14.5 (10.4 to 18.9)	97.4 (85.9 to 110)	-9.52 (-14.8 to -3.64)
Motor neuron disease	2.88 (1.87 to 4.11)	3.15 (2.08 to 4.45)	2.79 (1.61 to 4.45)	185 (173 to 197)	15.1 (10.5 to 20.2)	2.17 (1.42 to 3.1)	2.25 (1.47 to 3.22)	2.03 (1.17 to 3.33)	132 (121 to 142)	6.24 (1.54 to 11.1)
Headache disorders	303 (108 to 611)	210 (70.6 to 426)	113 (28.2 to 290)	145 (137 to 153)	-0.76 (-4.16 to 2.11)	483 (148 to 1000)	323 (102 to 682)	166 (38.6 to 368)	113 (99.7 to 121)	-2.33 (-8.44 to 1.42)
Migraine	243 (76 to 527)	167 (50.2 to 359)	80.9 (19.2 to 182)	147 (137 to 154)	-0.309 (-4.03 to 2.49)	408 (107 to 904)	270 (71.2 to 595)	129 (24.8 to 302)	115 (99.1 to 123)	-1.56 (-8.74 to 2.24)
Tension-type headache	59.2 (14.9 to 206)	42.7 (9.87 to 161)	32.6 (2.85 to 187)	141 (129 to 150)	-2.55 (-7.54 to 1.2)	75.3 (20.5 to 254)	53 (14 to 188)	36.1 (3.77 to 176)	104 (92.1 to 114)	-6.26 (-11.9 to -1.91)
Other neurological disorders	24.8 (17.7 to 33.6)	23.5 (16.1 to 31.7)	21.9 (15 to 29.5)	195 (186 to 206)	19.4 (15.7 to 23.8)	27.2 (19.2 to 37.2)	27.7 (19 to 37.7)	27 (18.7 to 36.1)	152 (146 to 159)	15.4 (12.9 to 18.6)
Mental disorders	1540 (1150 to 1980)	1330 (964 to 1720)	1080 (744 to 1480)	149 (146 to 152)	0.593 (-0.468 to 1.69)	1940 (1430 to 2510)	1660 (1220 to 2140)	1390 (932 to 1910)	115 (112 to 118)	-1.4 (-2.62 to -0.0042)
Schizophrenia	145 (105 to 187)	72.5 (51.6 to 95.3)	24.3 (14.7 to 35.1)	152 (146 to 159)	1.89 (-0.69 to 4.68)	142 (102 to 183)	76.9 (55.4 to 99.7)	32.2 (20.7 to 44.6)	116 (110 to 121)	-1.03 (-3.54 to 1.34)
Depressive disorders	710 (484 to 966)	662 (449 to 918)	599 (357 to 905)	153 (148 to 157)	2.13 (0.345 to 3.94)	1020 (695 to 1400)	910 (610 to 1240)	827 (496 to 1260)	119 (115 to 123)	0.32 (-1.54 to 2.35)
Major depressive disorder	572 (381 to 812)	559 (362 to 784)	508 (286 to 801)	153 (148 to 159)	2.47 (0.404 to 4.59)	848 (570 to 1200)	789 (515 to 1100)	733 (423 to 1140)	117 (113 to 123)	-0.381 (-2.57 to 2.01)
Dysthymia	138 (86.5 to 211)	103 (61.7 to 159)	91.1 (51 to 151)	149 (140 to 158)	0.664 (-2.99 to 4.29)	176 (112 to 267)	120 (73.1 to 183)	94.4 (52.8 to 157)	127 (119 to 136)	4.19 (0.393 to 8.08)
Bipolar disorder	100 (60.1 to 157)	67.8 (40.2 to 104)	42.3 (24.4 to 67.6)	133 (128 to 138)	-5.86 (-7.95 to -3.71)	103 (62.8 to 159)	68.1 (40.9 to 104)	45.9 (27 to 73.4)	96.3 (91.7 to 101)	-10 (-12.1 to -7.73)
Anxiety disorders	291 (193 to 419)	236 (150 to 341)	122 (70.8 to 189)	148 (143 to 154)	0.384 (-1.65 to 2.69)	474 (314 to 687)	415 (273 to 600)	285 (175 to 417)	111 (106 to 116)	-3.35 (-5.5 to -1.06)
Eating disorders				0 (0 to 0)	0 (0 to 0)				0 (0 to 0)	0 (0 to 0)
Anorexia nervosa				0 (0 to 0)	0 (0 to 0)				0 (0 to 0)	0 (0 to 0)
Bulimia nervosa				0 (0 to 0)	0 (0 to 0)				0 (0 to 0)	0 (0 to 0)
Autism spectrum disorders	66 (44.5 to 94.1)	63.5 (43.2 to 90.3)	62.8 (42.9 to 88.6)	147 (143 to 151)	-0.142 (-1.7 to 1.34)	18.3 (12.2 to 26.2)	17 (11.5 to 24.5)	16.2 (11 to 23.3)	115 (110 to 121)	-1.24 (-3.62 to 1.14)
Attention-deficit/hyperactivity disorder	0.531 (0.163 to 1.28)	0.0172 (0.00188 to 0.0616)	0.00000143 (0.00000000000877 to 0.0000137)	130 (98.8 to 162)	-6.86 (-19.6 to 5.95)	0.253 (0.0634 to 0.656)	0.0119 (0.00103 to 0.0461)	0.00000326 (0.00000000053 to 0.0000238)	96.2 (64 to 131)	-10 (-24.8 to 5.81)
Conduct disorder				0 (0 to 0)	0 (0 to 0)				0 (0 to 0)	0 (0 to 0)
Idiopathic developmental intellectual disability	19 (9.41 to 31.7)	11.2 (5.39 to 19.2)	3.78 (1.51 to 7.37)	106 (93 to 121)	-16.8 (-22 to -10.5)	20.3 (11.1 to 32.2)	13.9 (7.98 to 21.6)	6.44 (3.52 to 10.1)	85.2 (75.3 to 93.6)	-15.1 (-19.6 to -11.2)
Other mental disorders	211 (140 to 307)	212 (143 to 302)	229 (153 to 322)	147 (144 to 150)	-0.00705 (-1.27 to 1.2)	153 (102 to 222)	158 (108 to 227)	176 (117 to 249)	120 (117 to 123)	0.741 (-0.723 to 2.27)
Substance use disorders	256 (181 to 350)	208 (150 to 282)	258 (174 to 377)	170 (161 to 180)	9.15 (5.69 to 13.4)	125 (87.5 to 172)	136 (98.3 to 181)	215 (144 to 311)	157 (146 to 169)	17.7 (12.6 to 23.2)
Alcohol use disorders	191 (128 to 277)	143 (98.3 to 205)	193 (122 to 299)	151 (144 to 158)	1.36 (-1.46 to 4.47)	51.8 (33.8 to 77.5)	59.2 (39.2 to 86.3)	117 (71.2 to 188)	129 (122 to 138)	5.21 (1.96 to 9.16)
Drug use disorders	65.7 (42.4 to 94.6)	64.9 (42.6 to 88.8)	65.4 (41.5 to 98.3)	241 (221 to 262)	37.9 (29.6 to 46.2)	73.3 (46.9 to 106)	77.3 (51.5 to 108)	98.5 (61.6 to 150)	181 (163 to 203)	28.9 (20.7 to 38.8)
Opioid use disorders	55.4 (34.2 to 81.8)	56.6 (35.9 to 79.8)	54.1 (32.3 to 86.3)	265 (242 to 293)	47.7 (38.4 to 58.8)	66.5 (41.1 to 98.3)	71.1 (45.4 to 101)	88.8 (53.9 to 138)	192 (172 to 218)	34 (24.8 to 46)
Cocaine use disorders	3.75 (2.27 to 5.63)	3.34 (2.04 to 5.07)	4.15 (2.41 to 6.42)	147 (126 to 173)	-0.33 (-8.63 to 10.4)	2.46 (1.44 to 3.74)	2.42 (1.4 to 3.73)	3.07 (1.72 to 4.66)	95.4 (81 to 112)	-10.4 (-17 to -2.74)
Amphetamine use disorders	1.6 (0.846 to 2.67)	1.15 (0.593 to 2.08)	2.26 (0.96 to 4.4)	144 (135 to 156)	-1.39 (-5.11 to 3.37)	1.15 (0.578 to 1.95)	1.03 (0.552 to 1.86)	2.46 (1.14 to 4.65)	113 (105 to 125)	-2.14 (-6.02 to 3.33)
Cannabis use disorders	1.23 (0.666 to 2.07)	0.624 (0.309 to 1.1)	0.298 (0.129 to 0.59)	147 (134 to 161)	-0.342 (-5.57 to 5.51)	0.608 (0.309 to 1.06)	0.265 (0.116 to 0.51)	0.115 (0.0432 to 0.242)	106 (95.2 to 117)	-5.65 (-10.5 to -0.4)
Other drug use disorders	3.71 (2.38 to 5.34)	3.16 (2.06 to 4.54)	4.56 (2.86 to 6.74)	148 (133 to 167)	0.442 (-5.91 to 8.06)	2.61 (1.64 to 3.82)	2.52 (1.64 to 3.63)	4.07 (2.47 to 6.03)	105 (92.9 to 118)	-6.08 (-11.5 to 0.146)
Diabetes and kidney diseases	2640 (1860 to 3550)	2880 (2090 to 3810)	3050 (2190 to 4060)	250 (244 to 257)	41.4 (38.9 to 44.3)	2410 (1680 to 3250)	2540 (1830 to 3360)	2390 (1710 to 3180)	199 (193 to 204)	36.9 (34.3 to 39.5)
Diabetes mellitus	2240 (1560 to 3060)	2250 (1600 to 3020)	2080 (1460 to 2830)	258 (251 to 266)	44.6 (41.7 to 47.8)	2000 (1390 to 2740)	1940 (1370 to 2580)	1560 (1100 to 2120)	203 (197 to 209)	39 (36.2 to 41.8)
Diabetes mellitus type 1	45.9 (30.1 to 66)	50.5 (33.5 to 71.3)	59.9 (39.2 to 85.1)	236 (222 to 250)	35.8 (30 to 41.6)	43 (28.3 to 61.3)	47.8 (31.9 to 67.2)	55.6 (36.5 to 78.8)	179 (168 to 189)	27.9 (22.8 to 32.5)
Diabetes mellitus type 2	2190 (1520 to 2990)	2200 (1560 to 2960)	2020 (1420 to 2750)	258 (251 to 266)	44.8 (41.9 to 48)	1950 (1350 to 2690)	1890 (1340 to 2520)	1500 (1060 to 2050)	204 (198 to 210)	39.3 (36.5 to 42.1)
Chronic kidney disease	403 (264 to 612)	630 (436 to 911)	966 (626 to 1490)	217 (203 to 231)	28 (22.6 to 33.7)	411 (273 to 618)	602 (419 to 853)	829 (531 to 1290)	181 (168 to 193)	28.8 (23 to 34.5)
Chronic kidney disease due to diabetes mellitus type 1	4.6 (2.55 to 7.81)	1.64 (0.919 to 2.8)	0.217 (0.121 to 0.363)	248 (226 to 273)	40.7 (31.8 to 50.7)	3.48 (1.91 to 6.04)	1.15 (0.61 to 2.04)	0.123 (0.0662 to 0.216)	181 (164 to 204)	28.9 (20.9 to 39.5)

Supplementary Table 5. Cause-specific rate of YLDs for 70-79 years, 80-94 years, and 95+ years in 2019, and percentage change from 1990 to 2019 in YLD number and rate in 70+ years, global, for all GBD causes

	Males					Females				
	YLDs per 100,000, 2019			Percentage change in 70+, 1990-2019		YLDs per 100,000, 2019			Percentage change in 70+, 1990-2019	
Causes	70 to 79	80 to 94	95 plus	Number	Rate	70 to 79	80 to 94	95 plus	Number	Rate
Chronic kidney disease due to diabetes mellitus type 2	126 (78.6 to 196)	136 (91.2 to 204)	84.3 (55.9 to 128)	220 (205 to 235)	29.3 (23.2 to 35.6)	123 (77.7 to 194)	120 (81.4 to 179)	67 (43.3 to 102)	184 (171 to 198)	30.3 (24.5 to 36.4)
Chronic kidney disease due to hypertension	98.8 (62.3 to 153)	169 (113 to 252)	270 (172 to 427)	229 (215 to 246)	33.1 (27.2 to 39.7)	80.4 (51.2 to 124)	127 (87 to 187)	180 (115 to 287)	190 (176 to 204)	32.8 (26.7 to 39.3)
Chronic kidney disease due to glomerulonephritis	20.9 (12.5 to 33.1)	15.7 (9.8 to 24.4)	10.2 (5.14 to 20.9)	194 (178 to 210)	18.8 (12.4 to 25.3)	16.1 (9.51 to 25.8)	11 (6.94 to 17.3)	6.86 (3.35 to 14.4)	163 (148 to 180)	20.4 (13.6 to 28.2)
Chronic kidney disease due to other and unspecified causes	153 (102 to 226)	308 (215 to 431)	601 (387 to 917)	209 (195 to 224)	24.9 (19.3 to 30.9)	188 (129 to 275)	343 (240 to 476)	575 (359 to 901)	177 (164 to 191)	27 (20.8 to 33.2)
Acute glomerulonephritis	0.0321 (0.0164 to 0.0534)	0.0301 (0.0161 to 0.0494)	0.0297 (0.0146 to 0.0517)	39.7 (27.9 to 56)	-43.5 (-48.3 to -36.9)	0.0288 (0.0149 to 0.0473)	0.0237 (0.0127 to 0.0387)	0.0205 (0.0103 to 0.0356)	44.4 (34.5 to 58.6)	-33.8 (-38.4 to -27.3)
Skin and subcutaneous diseases	585 (380 to 893)	658 (415 to 1050)	766 (477 to 1190)	141 (135 to 145)	-2.61 (-4.92 to -0.888)	616 (401 to 931)	659 (419 to 1040)	776 (500 to 1200)	112 (106 to 116)	-2.98 (-5.49 to -1.08)
Dermatitis	127 (76.6 to 188)	131 (81.2 to 198)	139 (82.8 to 214)	146 (142 to 151)	-0.419 (-2.12 to 1.59)	151 (91.5 to 224)	139 (87.8 to 210)	136 (80.8 to 210)	118 (114 to 122)	-0.0954 (-1.74 to 1.59)
Atopic dermatitis	66.5 (37 to 107)	78.7 (43.4 to 130)	91.2 (50.3 to 151)	151 (146 to 157)	1.64 (-0.522 to 4.05)	85.3 (47.6 to 139)	88.7 (49.3 to 146)	95.5 (52.9 to 157)	119 (114 to 123)	0.178 (-1.95 to 2.32)
Contact dermatitis	51.6 (26.7 to 90.7)	42.7 (20.9 to 71.7)	39.8 (18.5 to 75.6)	140 (134 to 146)	-2.91 (-5.32 to -0.544)	58.4 (30.8 to 104)	43.5 (21.5 to 72.8)	34.7 (15.8 to 66.2)	117 (112 to 123)	-0.527 (-2.99 to 2.03)
Seborrhoeic dermatitis	8.62 (4.71 to 13.7)	9.4 (5.35 to 15)	7.85 (4.34 to 12.7)	142 (137 to 146)	-2.22 (-4.03 to -0.387)	7.23 (3.97 to 11.5)	7.32 (4.15 to 11.6)	6.09 (3.36 to 9.72)	118 (113 to 122)	-0.177 (-2.13 to 1.98)
Psoriasis	92.9 (67.3 to 120)	74.6 (54.2 to 97.1)	45.1 (33.2 to 57.6)	92.3 (88.9 to 96.2)	-22.3 (-23.6 to -20.7)	89.6 (65.5 to 117)	68.7 (49.8 to 89.2)	43.4 (32 to 55.4)	62 (58.9 to 65.3)	-25.7 (-27.2 to -24.2)
Bacterial skin diseases	6.76 (3.96 to 11.4)	6.85 (4.14 to 10.8)	7.48 (4.88 to 11.2)	170 (162 to 177)	9.02 (5.75 to 11.9)	5.76 (3.44 to 9.57)	6.19 (3.86 to 9.61)	7.22 (4.83 to 10.6)	123 (115 to 131)	2.35 (-1.47 to 5.87)
Cellulitis	2.97 (2.02 to 4.19)	3.69 (2.45 to 5.18)	5.39 (3.66 to 7.67)	162 (150 to 175)	5.98 (1.16 to 11.3)	2.86 (1.91 to 4.01)	3.61 (2.43 to 5.07)	5.43 (3.73 to 7.61)	110 (100 to 120)	-3.69 (-8.21 to 0.85)
Pyoderma	3.78 (1.54 to 7.98)	3.16 (1.3 to 6.49)	2.09 (0.851 to 4.29)	177 (171 to 183)	11.9 (9.75 to 14.3)	2.91 (1.17 to 6.12)	2.58 (1.06 to 5.3)	1.79 (0.728 to 3.71)	140 (135 to 145)	10.2 (7.93 to 12.3)
Scabies	39.9 (20.8 to 68)	43.1 (23.3 to 71.1)	34.8 (17.6 to 59.9)	178 (171 to 185)	12.4 (9.61 to 15.4)	38.9 (20.3 to 66.3)	42.6 (23.6 to 70.4)	35.6 (18.3 to 61.5)	175 (168 to 184)	26.3 (22.7 to 30.3)
Fungal skin diseases	91.6 (35.6 to 192)	159 (63.4 to 341)	245 (97.6 to 505)	151 (147 to 156)	1.55 (0.0194 to 3.3)	86.9 (33.9 to 183)	157 (63.3 to 337)	241 (96.3 to 496)	123 (119 to 128)	2.3 (0.288 to 4.67)
Viral skin diseases	41.1 (26.6 to 59.8)	32.5 (21.4 to 47.3)	22.1 (14.7 to 32.2)	145 (142 to 148)	-1.11 (-2.28 to 0.0559)	36.5 (23.4 to 52.9)	28.9 (19 to 41.8)	25.3 (16.9 to 36.5)	103 (101 to 106)	-6.74 (-7.9 to -5.51)
Acne vulgaris	5.54 (3.41 to 8.68)	3.81 (2.34 to 5.97)	1.25 (0.752 to 1.97)	187 (180 to 195)	16.1 (13.3 to 19.1)	6.1 (3.72 to 9.51)	3.98 (2.45 to 6.23)	1.41 (0.863 to 2.24)	141 (136 to 148)	10.7 (8.32 to 13.6)
Alopecia areata	3.6 (2.29 to 5.34)	2.12 (1.37 to 3.1)	0.809 (0.507 to 1.18)	147 (138 to 157)	-0.0984 (-3.94 to 3.73)	9.29 (5.94 to 13.7)	5.43 (3.58 to 7.91)	1.6 (1.03 to 2.32)	108 (103 to 112)	-4.86 (-7.09 to -2.62)
Pruritus	18.2 (8.3 to 34.2)	18.8 (8.52 to 37)	19.6 (8.79 to 37.2)	169 (164 to 175)	8.74 (6.73 to 11.3)	23.1 (10.4 to 43.4)	22 (10.1 to 42.6)	19.4 (8.61 to 36.5)	138 (133 to 143)	8.95 (6.74 to 11.4)
Urticaria	26.7 (17 to 40)	22 (14.6 to 32.3)	16.7 (9.5 to 27.2)	143 (138 to 149)	-1.78 (-3.89 to 0.636)	41.9 (26.6 to 62.8)	31 (20.6 to 45.3)	18.9 (10.7 to 30.4)	111 (107 to 116)	-3.13 (-5.08 to -0.998)
Decubitus ulcer	10.3 (6.91 to 14.7)	30.4 (21.2 to 41.1)	104 (68.1 to 154)	149 (137 to 164)	0.629 (-4.08 to 6.66)	9.53 (6.46 to 13.4)	34.5 (24.1 to 46.9)	123 (82.1 to 176)	116 (105 to 130)	-0.817 (-5.86 to 5.38)
Other skin and subcutaneous diseases	122 (58.6 to 221)	134 (64.8 to 241)	130 (64.3 to 233)	150 (148 to 151)	0.929 (0.352 to 1.48)	118 (56.6 to 214)	118 (57.2 to 213)	123 (61.1 to 220)	118 (117 to 119)	-0.0577 (-0.628 to 0.485)
Sense organ diseases	4000 (2790 to 5520)	5690 (4080 to 7560)	7130 (5240 to 9340)	136 (130 to 140)	-4.79 (-6.85 to -2.84)	4000 (2800 to 5490)	5760 (4140 to 7600)	7160 (5200 to 9360)	117 (113 to 121)	-0.502 (-2.39 to 1.32)
Blindness and vision loss	1480 (1040 to 2050)	2060 (1470 to 2760)	2820 (1990 to 3790)	122 (115 to 129)	-10.2 (-13 to -7.34)	1670 (1180 to 2300)	2250 (1610 to 2990)	2800 (1980 to 3780)	112 (106 to 117)	-3.04 (-5.34 to -0.487)
Glaucoma	92 (61.5 to 135)	199 (132 to 279)	408 (267 to 600)	84.8 (78.6 to 92.6)	-25.3 (-27.8 to -22.2)	68.4 (45.3 to 100)	144 (94.6 to 201)	284 (185 to 423)	77.4 (71.3 to 84.6)	-18.7 (-21.5 to -15.4)
Cataract	575 (399 to 796)	936 (656 to 1250)	1180 (777 to 1620)	122 (112 to 133)	-10.4 (-14.2 to -5.96)	718 (505 to 985)	1090 (772 to 1440)	1260 (849 to 1730)	118 (111 to 127)	-0.0255 (-3.48 to 4.16)
Age-related macular degeneration	51.6 (34.6 to 73.5)	77.9 (53.2 to 109)	129 (79.8 to 196)	124 (116 to 133)	-9.55 (-12.6 to -5.83)	61.9 (41.7 to 87.9)	104 (72 to 145)	202 (124 to 306)	93 (86.3 to 101)	-11.5 (-14.6 to -7.7)
Refraction disorders	290 (198 to 410)	305 (205 to 429)	208 (130 to 315)	121 (117 to 126)	-10.5 (-12.4 to -8.51)	306 (209 to 428)	314 (212 to 439)	220 (138 to 333)	102 (97.5 to 105)	-7.53 (-9.44 to -5.89)
Near vision loss	278 (121 to 557)	285 (125 to 553)	235 (92.3 to 487)	141 (136 to 148)	-2.56 (-4.74 to 0.215)	306 (133 to 613)	310 (138 to 607)	226 (90.7 to 467)	125 (119 to 133)	3.34 (0.602 to 6.67)
Other vision loss	196 (137 to 275)	257 (179 to 354)	661 (438 to 929)	128 (122 to 133)	-8.02 (-10.2 to -5.76)	213 (149 to 298)	290 (204 to 394)	612 (410 to 859)	108 (104 to 113)	-4.43 (-6.35 to -2.22)
Age-related and other hearing loss	2360 (1620 to 3370)	3460 (2410 to 4670)	4110 (2940 to 5550)	143 (136 to 150)	-1.75 (-4.54 to 0.883)	2140 (1470 to 3070)	3320 (2310 to 4460)	4140 (2960 to 5470)	120 (115 to 126)	1.07 (-1.62 to 3.81)
Other sense organ diseases	157 (79.1 to 270)	174 (93.7 to 297)	205 (101 to 355)	162 (156 to 170)	6.06 (3.47 to 9.03)	191 (96.4 to 326)	193 (105 to 330)	218 (108 to 381)	128 (123 to 135)	4.64 (2.3 to 7.72)
Musculoskeletal disorders	4050 (2800 to 5520)	3880 (2710 to 5380)	3280 (2240 to 4690)	144 (137 to 151)	-1.33 (-4.25 to 1.47)	5620 (3910 to 7670)	5220 (3660 to 7230)	4100 (2790 to 5930)	118 (113 to 123)	0.111 (-2.2 to 2.39)
Rheumatoid arthritis	70.6 (48.7 to 95.2)	57.6 (40.5 to 75.4)	29.9 (20.7 to 40)	165 (159 to 170)	7.13 (4.65 to 9.12)	144 (100 to 194)	103 (72.6 to 134)	57.4 (40.7 to 76)	134 (130 to 137)	7.16 (5.56 to 8.65)
Osteoarthritis	967 (482 to 1980)	1060 (539 to 2120)	1100 (564 to 2160)	157 (154 to 160)	3.87 (2.7 to 5.02)	1370 (696 to 2820)	1480 (752 to 2960)	1520 (781 to 2990)	124 (121 to 127)	2.84 (1.54 to 4.19)
Osteoarthritis hip	71.7 (33.8 to 151)	82.3 (39.1 to 171)	97.7 (47.3 to 201)	173 (169 to 178)	10.6 (8.94 to 12.5)	76.4 (36.6 to 159)	88.5 (42.5 to 181)	107 (52.3 to 218)	128 (124 to 133)	4.45 (2.72 to 6.59)
Osteoarthritis knee	543 (264 to 1100)	550 (268 to 1100)	471 (236 to 925)	163 (160 to 166)	6.28 (5.17 to 7.59)	800 (394 to 1620)	781 (385 to 1560)	628 (318 to 1240)	133 (131 to 136)	6.89 (5.69 to 8.21)
Osteoarthritis hand	209 (101 to 422)	246 (123 to 498)	316 (159 to 642)	135 (128 to 142)	-5.08 (-7.88 to -2.26)	368 (184 to 753)	444 (228 to 912)	584 (301 to 1180)	107 (101 to 113)	-5.21 (-7.78 to -2.18)
Osteoarthritis other	144 (71.2 to 295)	177 (90.8 to 364)	212 (110 to 414)	164 (160 to 168)	6.57 (5.04 to 8.16)	130 (64.9 to 267)	166 (84.2 to 334)	201 (104 to 390)	128 (125 to 132)	4.67 (3.26 to 6.16)
Low back pain	1680 (1120 to 2380)	1820 (1220 to 2550)	1480 (982 to 2150)	108 (103 to 114)	-15.9 (-17.7 to -13.7)	2300 (1530 to 3230)	2460 (1680 to 3380)	1910 (1270 to 2770)	92.7 (89 to 97.2)	-11.7 (-13.4 to -9.6)
Neck pain	519 (301 to 851)	433 (244 to 719)	423 (238 to 732)	146 (135 to 158)	-0.579 (-4.86 to 4.22)	644 (374 to 1050)	498 (278 to 827)	412 (233 to 686)	117 (107 to 127)	-0.734 (-4.9 to 4.04)
Gout	146 (83.7 to 219)	159 (96.7 to 249)	178 (104 to 277)	205 (196 to 218)	23.5 (19.7 to 28.4)	52.3 (30.6 to 78.4)	65.3 (40 to 100)	79.3 (45.9 to 123)	173 (162 to 188)	25 (20.1 to 31.8)
Other musculoskeletal disorders	661 (364 to 1080)	353 (137 to 651)	70.8 (6.92 to 227)	286 (240 to 388)	55.9 (37.6 to 97.2)	1110 (675 to 1680)	611 (321 to 991)	124 (23.8 to 370)	204 (182 to 239)	39.2 (29.2 to 55.3)
Other non-communicable diseases	1840 (1230 to 2630)	1880 (1280 to 2640)	1680 (1110 to 2360)	130 (126 to 134)	-7.01 (-8.51 to -5.37)	2110 (1450 to 2990)	2010 (1400 to 2770)	1810 (1220 to 2510)	92.4 (88.6 to 96.2)	-11.8 (-13.5 to -10)
Congenital birth defects	34.9 (22.4 to 53.3)	27.5 (17.4 to 42.9)	19.6 (12.2 to 31.3)	115 (99.4 to 131)	-13.1 (-19.4 to -6.51)	34.1 (22.6 to 50.5)	25.6 (16.8 to 39.6)	19.3 (12.5 to 29.7)	100 (90.6 to 110)	-8.15 (-12.6 to -3.65)
Neural tube defects	0.562 (0.344 to 0.815)	0.44 (0.274 to 0.654)	0.321 (0.183 to 0.487)	315 (284 to 347)	67.8 (55.3 to 80.8)	0.669 (0.419 to 0.948)	0.517 (0.325 to 0.756)	0.424 (0.244 to 0.631)	250 (229 to 273)	60.5 (50.8 to 71.1)
Congenital heart anomalies	3.51 (1.39 to 6.17)	3.42 (1.38 to 6.03)	3.18 (1.26 to 5.56)	171 (158 to 183)	9.49 (4.49 to 14.5)	3.5 (1.43 to 6.1)	3.13 (1.28 to 5.53)	3.23 (1.32 to 5.67)	130 (120 to 140)	5.39 (0.762 to 9.85)
Orofacial clefts	2.77 (1.74 to 4.11)	2.5 (1.57 to 3.66)	2.01 (1.24 to 2.95)	157 (138 to 180)	3.93 (-3.66 to 13)	2.6 (1.61 to 3.88)	2.27 (1.41 to 3.39)	1.79 (1.12 to 2.62)	150 (128 to 175)	14.5 (4.54 to 26.1)
Down syndrome				0 (0 to 0)	0 (0 to 0)				0 (0 to 0)	0 (0 to 0)
Turner syndrome				0 (0 to 0)	0 (0 to 0)	0.0554 (0.0203 to 0.106)	0.0385 (0.0147 to 0.0751)	0.0282 (0.0102 to 0.0576)	98.1 (93.5 to 103)	-9.18 (-11.3 to -7.01)
Klinefelter syndrome	0.00229 (0.000981 to 0.00444)	0.00127 (0.000511 to 0.00251)	0.000661 (0.00023 to 0.00143)	91 (84.1 to 97.6)	-22.8 (-25.6 to -20.1)				0 (0 to 0)	0 (0 to 0)
Other chromosomal abnormalities				0 (0 to 0)	0 (0 to 0)				0 (0 to 0)	0 (0 to 0)
Congenital musculoskeletal and limb anomalies	12.7 (7.93 to 18.3)	7.84 (4.71 to 11.8)	4.91 (2.78 to 8.01)	161 (149 to 173)	5.37 (0.487 to 10.4)	15.4 (9.81 to 21.8)	9.43 (5.81 to 14.1)	6.22 (3.62 to 10.1)	112 (104 to 121)	-2.75 (-6.4 to 1.37)
Urogenital congenital anomalies	0.000946 (0.0000258 to 0.00477)	0.00000234 (0.000000000298 to 0.0000206)	0.0000000204 (0.0000000000000128 to 0.000000197)	140 (120 to 204)	-3.09 (-11.2 to 22.9)	0.00182 (0.00016 to 0.00673)	0.0000139 (0.00000000838 to 0.000119)	0.000000122 (0.000000000000306 to 0.00000115)	104 (82.1 to 124)	-6.69 (-16.5 to 2.73)
Digestive congenital anomalies	0.291 (0.177 to 0.444)	0.304 (0.187 to 0.467)	0.339 (0.204 to 0.529)	90.9 (70.3 to 111)	-22.8 (-31.2 to -14.7)	0.381 (0.235 to 0.575)	0.418 (0.25 to 0.644)	0.521 (0.309 to 0.814)	58.1 (44.5 to 73.5)	-27.5 (-33.7 to -20.5)
Other congenital birth defects	15.1 (6.67 to 31)	13 (5.68 to 27.1)	8.83 (3.69 to 19.6)	74.5 (52.2 to 92.5)	-29.5 (-38.5 to -22.2)	11.6 (4.91 to 25.1)	9.78 (4.14 to 21.5)	7.06 (2.89 to 16.5)	71.8 (50.4 to 87.2)	-21.3 (-31.1 to -14.2)
Urinary diseases and male infertility	463 (273 to 715)	417 (249 to 638)	203 (123 to 313)	146 (138 to 156)	-0.696 (-3.7 to 3.38)	10.9 (6.93 to 15.6)	8.13 (5.22 to 11.4)	8.3 (5.22 to 12.7)	99.5 (91.5 to 108)	-8.56 (-12.2 to -4.78)

Supplementary Table 5. Cause-specific rate of YLDs for 70-79 years, 80-94 years, and 95+ years in 2019, and percentage change from 1990 to 2019 in YLD number and rate in 70+ years, global, for all GBD causes

	Males					Females				
	YLDs per 100,000, 2019			Percentage change in 70+, 1990-2019		YLDs per 100,000, 2019			Percentage change in 70+, 1990-2019	
Causes	70 to 79	80 to 94	95 plus	Number	Rate	70 to 79	80 to 94	95 plus	Number	Rate
Urinary tract infections and interstitial nephritis	2.02 (1.21 to 3.09)	2.47 (1.5 to 3.59)	4.62 (2.79 to 7.04)	169 (158 to 181)	8.69 (4.1 to 13.8)	5.08 (2.98 to 7.7)	5.06 (3.09 to 7.36)	6.77 (4.04 to 10.6)	112 (105 to 120)	-2.84 (-5.82 to 0.763)
Urolithiasis	9.27 (5.33 to 14.9)	5.19 (3.1 to 8.12)	2.45 (1.39 to 3.91)	73.7 (64 to 86.8)	-29.8 (-33.7 to -24.5)	3.96 (2.23 to 6.63)	1.97 (1.12 to 3.1)	0.914 (0.504 to 1.51)	86.6 (75.8 to 101)	-14.5 (-19.4 to -7.91)
Benign prostatic hyperplasia	450 (260 to 696)	408 (242 to 628)	196 (116 to 304)	148 (140 to 160)	0.208 (-2.98 to 5.02)				0 (0 to 0)	0 (0 to 0)
Male infertility				0 (0 to 0)	0 (0 to 0)				0 (0 to 0)	0 (0 to 0)
Other urinary diseases	2.28 (1.38 to 3.52)	1.25 (0.796 to 1.8)	0.568 (0.376 to 0.815)	76.5 (65.2 to 90.6)	-28.6 (-33.2 to -23)	1.81 (1.16 to 2.63)	1.11 (0.709 to 1.57)	0.618 (0.389 to 0.947)	90.2 (82.2 to 98.1)	-12.8 (-16.5 to -9.21)
Gynecological diseases				0 (0 to 0)	0 (0 to 0)	327 (208 to 471)	265 (166 to 389)	227 (124 to 377)	71.8 (65.9 to 78.6)	-21.2 (-23.9 to -18.1)
Uterine fibroids				0 (0 to 0)	0 (0 to 0)	13.1 (5.82 to 26.3)	5.88 (2.64 to 11.5)	2.57 (1.15 to 4.92)	89.5 (82.5 to 97.5)	-13.1 (-16.4 to -9.44)
Polycystic ovarian syndrome				0 (0 to 0)	0 (0 to 0)				0 (0 to 0)	0 (0 to 0)
Female infertility				0 (0 to 0)	0 (0 to 0)				0 (0 to 0)	0 (0 to 0)
Endometriosis				0 (0 to 0)	0 (0 to 0)				0 (0 to 0)	0 (0 to 0)
Genital prolapse				0 (0 to 0)	0 (0 to 0)	31.1 (14.6 to 57.2)	31 (14.1 to 57.6)	31.2 (14.7 to 60.5)	88.7 (84 to 94.3)	-13.5 (-15.7 to -10.9)
Premenstrual syndrome				0 (0 to 0)	0 (0 to 0)				0 (0 to 0)	0 (0 to 0)
Other gynecological diseases				0 (0 to 0)	0 (0 to 0)	283 (178 to 415)	228 (139 to 342)	193 (94.6 to 343)	69.4 (63.3 to 76.7)	-22.3 (-25.2 to -19)
Hemoglobinopathies and hemolytic anemias	76.9 (52 to 111)	85 (58.6 to 121)	117 (77.4 to 167)	83.7 (72.9 to 95)	-25.7 (-30.1 to -21.2)	96.1 (65.4 to 135)	82.5 (56.7 to 119)	63.7 (43.1 to 91)	85.9 (75.2 to 96.7)	-14.8 (-19.7 to -9.84)
Thalassemias	0.000788 (0.000419 to 0.00133)	0.0000453 (0.0000217 to 0.0000921)	0.000000976 (0.000000405 to 0.00000218)	178 (144 to 229)	12.2 (-1.36 to 33)	0.00144 (0.000781 to 0.00244)	0.0000854 (0.0000392 to 0.000176)	0.00000319 (0.00000112 to 0.00000857)	171 (135 to 216)	24 (7.52 to 44.7)
Thalassemias trait	30 (19.6 to 45.1)	31.3 (21 to 45.8)	20.7 (13.4 to 30.4)	102 (87.3 to 118)	-18.3 (-24.3 to -11.9)	34.9 (22.9 to 51.8)	32.6 (21.8 to 48.4)	21.3 (14.2 to 31.5)	92.1 (78.8 to 106)	-11.9 (-18 to -5.61)
Sickle cell disorders	0.00158 (0.00077 to 0.00289)	0.000213 (0.0000715 to 0.000459)	0.0000535 (0.0000116 to 0.000149)	391 (290 to 550)	98.4 (57.8 to 163)	0.00521 (0.00278 to 0.00889)	0.000915 (0.000371 to 0.00169)	0.000205 (0.0000564 to 0.000493)	214 (171 to 276)	44 (24.3 to 72.4)
Sickle cell trait	11.2 (7.36 to 16)	10.3 (6.83 to 14.6)	6.89 (4.47 to 9.67)	144 (129 to 160)	-1.32 (-7.37 to 5.06)	15 (9.98 to 20.9)	11.1 (7.47 to 15.5)	6.01 (3.97 to 8.43)	151 (140 to 162)	15 (9.82 to 20.2)
G6PD deficiency	0.331 (0.219 to 0.469)	0.328 (0.221 to 0.471)	0.272 (0.174 to 0.394)	150 (134 to 168)	1.03 (-5.56 to 8.32)	0.118 (0.0785 to 0.167)	0.0929 (0.0625 to 0.132)	0.0595 (0.0395 to 0.0861)	169 (160 to 179)	23.5 (19.3 to 27.8)
G6PD trait				0 (0 to 0)	0 (0 to 0)	0.00374 (0.00251 to 0.00538)	0.00355 (0.00233 to 0.00526)	0.00264 (0.00173 to 0.00389)	127 (118 to 137)	3.98 (-0.119 to 8.47)
Other hemoglobinopathies and hemolytic anemias	35.3 (24 to 50.7)	43.1 (29.4 to 61.4)	89 (58.2 to 129)	60.3 (49.7 to 70.8)	-35.2 (-39.5 to -31)	46 (31.7 to 64.9)	38.7 (26 to 55.3)	36.3 (24.5 to 53.2)	68 (57 to 80)	-23 (-28 to -17.5)
Endocrine, metabolic, blood, and immune disorders	398 (247 to 607)	322 (200 to 494)	247 (143 to 398)	137 (126 to 147)	-4.37 (-8.46 to -0.222)	584 (363 to 899)	392 (243 to 605)	270 (155 to 439)	98.7 (90.4 to 106)	-8.9 (-12.7 to -5.36)
Oral disorders	867 (535 to 1330)	1030 (644 to 1530)	1100 (684 to 1610)	126 (121 to 131)	-8.66 (-10.6 to -6.67)	1060 (665 to 1590)	1240 (795 to 1780)	1220 (774 to 1760)	96.2 (91.5 to 101)	-10.1 (-12.2 to -7.91)
Caries of deciduous teeth				0 (0 to 0)	0 (0 to 0)				0 (0 to 0)	0 (0 to 0)
Caries of permanent teeth	24.7 (10.4 to 49.3)	19.8 (8.12 to 39.2)	12.2 (4.29 to 27.8)	129 (124 to 136)	-7.3 (-9.47 to -4.77)	26.2 (11.2 to 52.4)	20.4 (8.34 to 40)	12 (4.18 to 27.3)	106 (101 to 112)	-5.59 (-7.94 to -3)
Periodontal diseases	171 (68.7 to 347)	145 (56 to 297)	122 (44.6 to 263)	161 (156 to 167)	5.41 (3.37 to 8.07)	153 (61.6 to 312)	126 (47.7 to 257)	109 (40.1 to 234)	139 (132 to 148)	9.43 (6.18 to 13.7)
Edentulism	621 (376 to 945)	837 (523 to 1260)	941 (564 to 1390)	118 (114 to 123)	-11.9 (-13.6 to -9.86)	823 (502 to 1230)	1060 (674 to 1560)	1070 (662 to 1570)	89.7 (85.2 to 94.9)	-13 (-15.1 to -10.7)
Other oral disorders	50.8 (31.8 to 75)	30.6 (19.2 to 44.1)	22.3 (13.7 to 33.7)	142 (140 to 145)	-2 (-3.03 to -0.936)	57.9 (35.9 to 85.3)	35.8 (22.4 to 50.8)	27.2 (16.7 to 39.8)	113 (111 to 116)	-2.23 (-3.21 to -1.17)
Sudden infant death syndrome										
Injuries	2240 (1600 to 3030)	2890 (2020 to 3930)	4280 (2990 to 5880)	154 (149 to 158)	2.52 (0.741 to 4.3)	1940 (1410 to 2600)	3140 (2260 to 4240)	5450 (3820 to 7450)	129 (126 to 132)	5.1 (3.77 to 6.55)
Transport injuries	690 (495 to 920)	659 (464 to 882)	587 (416 to 790)	173 (169 to 178)	10.4 (8.64 to 12.2)	454 (332 to 605)	431 (308 to 574)	362 (255 to 486)	107 (104 to 110)	-5.13 (-6.52 to -3.83)
Road injuries	661 (474 to 882)	626 (440 to 839)	526 (371 to 710)	179 (175 to 184)	13 (11.1 to 14.9)	427 (312 to 569)	396 (280 to 531)	290 (204 to 396)	110 (106 to 113)	-3.87 (-5.43 to -2.47)
Pedestrian road injuries	175 (122 to 235)	177 (125 to 239)	138 (95.1 to 188)	146 (142 to 150)	-0.674 (-2.22 to 1.07)	144 (102 to 193)	150 (107 to 201)	105 (72.5 to 142)	94.1 (90.6 to 97.3)	-11 (-12.6 to -9.54)
Cyclist road injuries	140 (99 to 189)	127 (88.1 to 172)	95 (65.7 to 130)	200 (195 to 206)	21.2 (19.1 to 23.6)	78.4 (56 to 105)	69.1 (48.5 to 93)	46.9 (32.4 to 64.3)	118 (114 to 121)	-0.179 (-1.94 to 1.39)
Motorcyclist road injuries	193 (136 to 263)	176 (121 to 242)	148 (102 to 205)	284 (274 to 294)	55.1 (51.3 to 59.3)	85.5 (60.3 to 115)	68.2 (47.9 to 93.4)	48 (33.4 to 67)	235 (227 to 245)	53.7 (50.1 to 58.2)
Motor vehicle road injuries	143 (102 to 196)	136 (96 to 187)	136 (94.4 to 187)	122 (118 to 126)	-10.2 (-11.8 to -8.68)	112 (79.9 to 152)	102 (72.1 to 139)	85.5 (59.3 to 118)	76.1 (73.4 to 78.6)	-19.3 (-20.5 to -18.1)
Other road injuries	10.6 (7.35 to 14.6)	10.7 (7.33 to 14.8)	9.96 (6.81 to 14)	185 (181 to 189)	15.1 (13.7 to 16.7)	7.43 (5.12 to 10.2)	6.9 (4.72 to 9.55)	5.42 (3.68 to 7.66)	106 (103 to 110)	-5.37 (-7.16 to -3.62)
Other transport injuries	28.4 (20.5 to 38.1)	33.4 (24.3 to 45.1)	61.3 (43.1 to 83.6)	83.2 (80.3 to 86.4)	-26 (-27.1 to -24.6)	26.8 (19.3 to 36.1)	34.9 (25.2 to 47)	71.5 (49.4 to 97.9)	75.4 (72.8 to 78.3)	-19.6 (-20.8 to -18.3)
Unintentional injuries	1420 (997 to 1940)	2100 (1470 to 2900)	3550 (2470 to 4910)	149 (144 to 156)	0.856 (-1.45 to 3.3)	1400 (1000 to 1900)	2630 (1870 to 3590)	5030 (3510 to 6870)	138 (135 to 142)	9.21 (7.56 to 11.1)
Falls	913 (655 to 1250)	1510 (1070 to 2090)	2700 (1880 to 3730)	186 (179 to 193)	15.5 (12.8 to 18.3)	1040 (748 to 1410)	2200 (1560 to 2980)	4410 (3100 to 6030)	156 (152 to 160)	17.3 (15.5 to 19.2)
Drowning	4.74 (3.32 to 6.43)	7.76 (5.44 to 10.6)	12.5 (8.55 to 17.4)	126 (116 to 137)	-8.55 (-12.8 to -4.2)	7.01 (4.85 to 9.55)	14.5 (10.1 to 20)	29.8 (19.9 to 41.3)	254 (239 to 269)	62.1 (55.4 to 69.2)
Fire, heat, and hot substances	50.9 (29 to 83)	56.3 (32.3 to 93.2)	76.2 (44.7 to 122)	85 (81.5 to 88)	-25.2 (-26.6 to -24)	54.1 (32.2 to 86.9)	56.8 (32.8 to 93.3)	66.1 (38.3 to 107)	76.4 (72.3 to 79.6)	-19.1 (-21 to -17.7)
Poisonings	6.26 (4.21 to 8.59)	6.94 (4.77 to 9.44)	8.84 (6.17 to 12)	90.4 (84.5 to 96.8)	-23 (-25.4 to -20.5)	7.28 (4.97 to 9.99)	8.34 (5.8 to 11.4)	10.6 (7.39 to 14.4)	60.7 (56.1 to 65)	-26.3 (-28.5 to -24.4)
Poisoning by carbon monoxide	1.33 (0.835 to 1.98)	1.35 (0.883 to 1.97)	1.27 (0.85 to 1.8)	155 (144 to 166)	2.95 (-1.36 to 7.48)	1.5 (0.953 to 2.22)	1.66 (1.08 to 2.41)	1.87 (1.24 to 2.68)	93.3 (87.7 to 98.6)	-11.4 (-14 to -8.96)
Poisoning by other means	4.93 (3.23 to 6.88)	5.59 (3.69 to 7.83)	7.57 (5.13 to 10.5)	78.6 (73.6 to 83.6)	-27.8 (-29.8 to -25.8)	5.78 (3.79 to 8.1)	6.68 (4.42 to 9.29)	8.77 (5.91 to 12.3)	54.1 (49.9 to 58.1)	-29.4 (-31.3 to -27.5)
Exposure to mechanical forces	239 (155 to 351)	280 (182 to 410)	371 (241 to 546)	93.1 (90.9 to 95.2)	-22 (-22.8 to -21.1)	112 (75.3 to 161)	137 (91.5 to 196)	191 (126 to 275)	93.2 (91 to 95.2)	-11.4 (-12.4 to -10.5)
Unintentional firearm injuries	8.73 (5.9 to 12.6)	9.94 (6.74 to 14.4)	17.8 (11.9 to 26.1)	84.5 (80.6 to 88.5)	-25.4 (-27 to -23.8)	6 (4.15 to 8.32)	6.55 (4.53 to 9.19)	8.94 (5.98 to 13)	101 (97.7 to 104)	-7.99 (-9.34 to -6.66)
Other exposure to mechanical forces	231 (149 to 339)	270 (174 to 396)	354 (229 to 519)	93.4 (91.1 to 95.6)	-21.8 (-22.7 to -20.9)	106 (70.6 to 153)	131 (87.2 to 186)	182 (120 to 263)	92.8 (90.6 to 94.8)	-11.6 (-12.6 to -10.7)
Adverse effects of medical treatment	8.22 (4.4 to 13.9)	8.53 (4.84 to 13.9)	12.6 (6.21 to 23)	224 (210 to 242)	31.1 (25.2 to 38.1)	6.98 (3.76 to 11.8)	6.55 (3.74 to 10.6)	8.16 (4 to 15.1)	132 (121 to 143)	6.17 (1.21 to 11.2)
Animal contact	16.2 (10.7 to 23.9)	17.4 (11.6 to 25.8)	23 (15.3 to 34.2)	76.3 (70.4 to 81.3)	-28.7 (-31.1 to -26.7)	15.1 (10 to 22.2)	15.6 (10.3 to 23)	18.7 (12.3 to 27.8)	82.2 (77.8 to 85.9)	-16.5 (-18.5 to -14.8)
Venomous animal contact	4.98 (3.42 to 6.89)	5.01 (3.48 to 6.94)	6.16 (4.31 to 8.6)	64.2 (59.4 to 68.6)	-33.6 (-35.6 to -31.8)	4.57 (3.11 to 6.35)	4.34 (3.01 to 6)	4.55 (3.17 to 6.35)	71.2 (67.3 to 75)	-21.5 (-23.3 to -19.8)
Non-venomous animal contact	11.2 (6.88 to 17.3)	12.4 (7.8 to 19.2)	16.9 (10.7 to 25.7)	82.1 (75.6 to 87)	-26.4 (-29 to -24.4)	10.5 (6.46 to 16.2)	11.2 (7.01 to 17.4)	14.1 (8.8 to 21.9)	87.2 (83 to 90.7)	-14.2 (-16.1 to -12.6)
Foreign body	41.6 (29 to 57.4)	48.2 (34.5 to 66.9)	74.8 (52.6 to 104)	124 (121 to 127)	-9.47 (-10.7 to -8.16)	29 (20.7 to 39.2)	34.2 (24.6 to 46.6)	52 (36.9 to 71.6)	94.2 (91.6 to 96.6)	-11 (-12.1 to -9.85)
Pulmonary aspiration and foreign body in airway	1.87 (1.33 to 2.53)	3.05 (2.14 to 4.14)	6.52 (4.42 to 9.45)	95.2 (89.6 to 102)	-21.1 (-23.3 to -18.5)	2.5 (1.81 to 3.35)	3.46 (2.44 to 4.63)	6.17 (4.23 to 8.77)	73.1 (69.7 to 77.2)	-20.6 (-22.2 to -18.8)
Foreign body in eyes	15.2 (9.8 to 22.2)	14.5 (9.74 to 21.1)	12.6 (8.68 to 17.7)	122 (118 to 126)	-10.2 (-11.7 to -8.67)	8.41 (5.42 to 12.4)	8.63 (5.78 to 12.5)	9.25 (6.41 to 13)	100 (97.2 to 103)	-8.15 (-9.59 to -6.76)
Foreign body in other body part	24.5 (17.1 to 33.4)	30.6 (21.6 to 42.5)	55.8 (38.9 to 78.2)	128 (123 to 132)	-7.93 (-9.77 to -6.07)	18.1 (12.9 to 24.2)	22.1 (16 to 30.2)	36.6 (25.7 to 51.1)	95.1 (91.2 to 98.8)	-10.6 (-12.3 to -8.86)
Environmental heat and cold exposure	25.8 (17 to 36.4)	34.3 (23.2 to 48.1)	67.6 (45.3 to 94.7)	104 (99.9 to 108)	-17.6 (-19.2 to -16.1)	19.2 (12.8 to 27.2)	25.6 (17.5 to 36)	44.4 (29.9 to 61.8)	88.7 (85.5 to 91.4)	-13.5 (-15 to -12.2)
Exposure to forces of nature	9.45 (6.92 to 12.4)	8.04 (5.86 to 10.6)	7.22 (5.13 to 9.91)	721 (552 to 950)	232 (164 to 324)	6.28 (4.41 to 8.78)	5.09 (3.59 to 7.02)	4.04 (2.77 to 5.68)	612 (469 to 794)	226 (161 to 310)

Supplementary Table 5. Cause-specific rate of YLDs for 70-79 years, 80-94 years, and 95+ years in 2019, and percentage change from 1990 to 2019 in YLD number and rate in 70+ years, global, for all GBD causes

	Males					Females				
	YLDs per 100,000, 2019			Percentage change in 70+, 1990-2019		YLDs per 100,000, 2019			Percentage change in 70+, 1990-2019	
Causes	70 to 79	80 to 94	95 plus	Number	Rate	70 to 79	80 to 94	95 plus	Number	Rate
Other unintentional injuries	99.9 (67 to 145)	123 (82.1 to 177)	190 (132 to 267)	88.9 (87.2 to 90.8)	-23.6 (-24.3 to -22.9)	96.6 (64.8 to 138)	126 (85.8 to 177)	196 (137 to 271)	74.4 (71.3 to 76.5)	-20 (-21.5 to -19.1)
Self-harm and interpersonal violence	134 (98.5 to 177)	127 (93.3 to 167)	148 (109 to 197)	118 (109 to 126)	-12.1 (-15.5 to -8.53)	88.7 (68.6 to 112)	81.3 (62.2 to 103)	64.7 (47.5 to 86.1)	82.4 (75.8 to 89.6)	-16.4 (-19.4 to -13.1)
Self-harm	18 (12.8 to 24.3)	18.7 (13.2 to 25.3)	19.1 (13.3 to 26.2)	104 (99.7 to 108)	-17.6 (-19.3 to -15.8)	24.1 (17.3 to 31.8)	23.2 (16.6 to 31.1)	20.9 (14.7 to 28.8)	60.1 (56.8 to 63.5)	-26.6 (-28.1 to -25.1)
Self-harm by firearm	2.65 (1.9 to 3.61)	2.96 (2.06 to 4.12)	2.67 (1.77 to 3.81)	105 (99.7 to 109)	-17 (-19.3 to -15.3)	2.36 (1.72 to 3.16)	2.22 (1.59 to 3.03)	1.3 (0.88 to 1.83)	111 (104 to 116)	-3.41 (-6.33 to -0.954)
Self-harm by other specified means	15.4 (10.7 to 20.7)	15.7 (10.9 to 21.4)	16.4 (11.3 to 22.8)	104 (99.1 to 108)	-17.7 (-19.5 to -15.8)	21.7 (15.3 to 28.8)	21 (14.9 to 28.2)	19.6 (13.8 to 27)	56.1 (53 to 59.2)	-28.4 (-29.9 to -27)
Interpersonal violence	92.2 (67.8 to 124)	85.1 (61.9 to 117)	103 (72.9 to 143)	115 (112 to 119)	-12.9 (-14.3 to -11.5)	52.3 (40.1 to 66.8)	46.7 (35.4 to 60.3)	35.3 (25.4 to 49.2)	94.8 (89.2 to 102)	-10.7 (-13.2 to -7.41)
Physical violence by firearm	5.16 (3.61 to 7.12)	5.11 (3.5 to 7.23)	9.72 (6.54 to 13.7)	195 (190 to 201)	19.4 (17.3 to 21.7)	1.68 (1.18 to 2.25)	1.74 (1.21 to 2.42)	2.38 (1.59 to 3.4)	141 (137 to 145)	10.5 (8.64 to 12.5)
Physical violence by sharp object	13.9 (9.65 to 19.7)	14.2 (9.7 to 20.3)	24.4 (16.5 to 34.9)	86.3 (82 to 90.2)	-24.7 (-26.4 to -23.1)	4.66 (3.26 to 6.45)	5.05 (3.48 to 7.05)	6.1 (4.13 to 8.69)	57.1 (53.4 to 60.8)	-28 (-29.7 to -26.3)
Sexual violence	7.64 (4.87 to 11.3)	5.46 (3.5 to 8.12)	0.272 (0.148 to 0.47)	161 (159 to 165)	5.63 (4.51 to 6.94)	16 (10.3 to 23.6)	11.3 (7.36 to 16.6)	0.753 (0.419 to 1.29)	147 (144 to 150)	13.1 (11.9 to 14.4)
Physical violence by other means	65.4 (45.6 to 90.5)	60.3 (41.8 to 84.1)	68.7 (48.3 to 96.5)	114 (111 to 117)	-13.5 (-14.9 to -12.1)	29.9 (21.3 to 40.7)	28.6 (20.1 to 39.3)	26.1 (18.4 to 36.2)	81.5 (78 to 85)	-16.8 (-18.4 to -15.2)
Conflict and terrorism	23.8 (15 to 39.7)	23.1 (15.5 to 34.7)	25.2 (17.3 to 35.2)	135 (91.7 to 189)	-4.91 (-22.5 to 16.9)	12.1 (7.45 to 20.4)	11.1 (7.07 to 16.8)	8.15 (5.35 to 11.9)	81.5 (50.1 to 120)	-16.8 (-31.2 to 1.01)
Executions and police conflict	0.448 (0.258 to 0.803)	0.493 (0.314 to 0.785)	0.642 (0.4 to 1)	720 (526 to 994)	231 (153 to 342)	0.221 (0.136 to 0.36)	0.291 (0.181 to 0.455)	0.288 (0.176 to 0.46)	388 (221 to 593)	124 (47.2 to 218)
Total cancers	723 (530 to 913)	921 (681 to 1170)	723 (521 to 913)	191 (168 to 216)	17.5 (8.4 to 27.8)	425 (318 to 546)	523 (377 to 665)	518 (373 to 658)	141 (124 to 158)	10.5 (2.55 to 18.2)
Total burden related to hepatitis B	11.8 (7.97 to 16.8)	10 (6.81 to 14.6)	3.86 (2.32 to 5.97)	96.5 (72.4 to 125)	-20.6 (-30.3 to -9.16)	5.76 (3.73 to 8.45)	4.53 (2.92 to 6.73)	2.27 (1.28 to 3.64)	57.6 (39.2 to 80)	-27.8 (-36.2 to -17.5)
Total burden related to hepatitis C	10.7 (7.13 to 15.2)	13.5 (9.39 to 18.3)	9.3 (6.2 to 13.1)	176 (159 to 198)	11.8 (4.7 to 20.6)	7.62 (5.1 to 11)	9.13 (6.2 to 12.8)	7.57 (5.12 to 10.6)	118 (102 to 135)	-0.0976 (-7.59 to 7.95)
Total burden related to Non-alcoholic fatty liver disease (NAFLD)	2.91 (1.65 to 4.68)	3.1 (1.8 to 4.9)	1.7 (0.862 to 3.18)	168 (148 to 195)	8.36 (0.441 to 19.1)	3.34 (1.84 to 5.33)	3.2 (1.85 to 5.09)	1.88 (0.96 to 3.43)	126 (110 to 144)	3.69 (-3.6 to 12)

Supplementary Table 6: Life expectancy, Health-adjusted life expectancy, and proportion of years spent in ill health in all reporting locations, in 1990, 2005, 2019

location	LE-70			HALE-70			PYIH-70		
	1990	2005	2019	1990	2005	2019	1990	2005	2019
**Global**	11.8 (11.7 to 12)	12.8 (12.7 to 12.9)	14.1 (13.9 to 14.4)	8.48 (7.57 to 9.29)	9.18 (8.24 to 10)	10.1 (9.01 to 11)	0.28 (0.22 to 0.35)	0.28 (0.22 to 0.35)	0.29 (0.23 to 0.35)
**Low SDI**	9.53 (9.34 to 9.74)	10.3 (10.1 to 10.5)	11.5 (11.2 to 11.8)	6.79 (6.07 to 7.48)	7.32 (6.55 to 8.07)	8.2 (7.31 to 9.04)	0.29 (0.23 to 0.35)	0.29 (0.23 to 0.35)	0.29 (0.24 to 0.35)
**Low-middle SDI**	10 (9.88 to 10.2)	11.1 (10.9 to 11.2)	12.3 (11.9 to 12.7)	7.09 (6.33 to 7.81)	7.8 (6.94 to 8.57)	8.62 (7.66 to 9.55)	0.29 (0.24 to 0.36)	0.3 (0.24 to 0.36)	0.3 (0.25 to 0.36)
**Middle SDI**	11.2 (10.9 to 11.5)	11.9 (11.7 to 12.1)	13.3 (12.8 to 13.7)	8.06 (7.19 to 8.83)	8.59 (7.72 to 9.39)	9.49 (8.54 to 10.4)	0.28 (0.23 to 0.34)	0.28 (0.22 to 0.34)	0.28 (0.24 to 0.34)
**High-middle SDI**	11.8 (11.7 to 12)	12.6 (12.5 to 12.7)	14.5 (14.1 to 14.8)	8.55 (7.66 to 9.35)	9.14 (8.21 to 9.97)	10.4 (9.38 to 11.4)	0.28 (0.22 to 0.34)	0.28 (0.22 to 0.34)	0.28 (0.23 to 0.34)
**High SDI**	13.8 (13.7 to 13.8)	15.5 (15.5 to 15.5)	16.7 (16.6 to 16.8)	9.81 (8.76 to 10.8)	11.1 (9.87 to 12.1)	11.8 (10.5 to 13)	0.29 (0.22 to 0.36)	0.29 (0.22 to 0.36)	0.29 (0.23 to 0.37)
**Central Europe, Eastern Europe, and Central Asia**	11.6 (11.6 to 11.6)	11.4 (11.4 to 11.4)	13.2 (12.8 to 13.6)	8.43 (7.55 to 9.22)	8.34 (7.48 to 9.1)	9.62 (8.59 to 10.5)	0.27 (0.21 to 0.35)	0.27 (0.2 to 0.34)	0.27 (0.23 to 0.33)
Central Asia	12.1 (12 to 12.2)	9.84 (9.76 to 9.93)	11 (10.6 to 11.4)	9.1 (8.27 to 9.86)	7.46 (6.8 to 8.07)	8.26 (7.49 to 9.04)	0.25 (0.19 to 0.31)	0.24 (0.19 to 0.3)	0.25 (0.21 to 0.29)
Armenia	12 (11.9 to 12.1)	11.9 (11.8 to 12)	12.9 (12.1 to 13.8)	8.92 (8.1 to 9.68)	8.85 (8.02 to 9.6)	9.61 (8.58 to 10.6)	0.26 (0.2 to 0.32)	0.25 (0.2 to 0.32)	0.26 (0.23 to 0.29)
Azerbaijan	12.5 (12.1 to 12.8)	9.89 (9.65 to 10.1)	10.4 (9.83 to 10.9)	9.47 (8.63 to 10.3)	7.54 (6.85 to 8.17)	7.89 (7.07 to 8.66)	0.24 (0.2 to 0.29)	0.24 (0.19 to 0.29)	0.24 (0.21 to 0.28)
Georgia	11.1 (10.8 to 11.5)	11.4 (11.1 to 11.7)	12.4 (11.5 to 13.3)	8.47 (7.68 to 9.18)	8.62 (7.83 to 9.37)	9.18 (8.17 to 10.2)	0.24 (0.2 to 0.29)	0.25 (0.2 to 0.29)	0.26 (0.23 to 0.29)
Kazakhstan	11.8 (11.7 to 12)	10.4 (10.3 to 10.5)	11.9 (11.3 to 12.5)	8.75 (7.88 to 9.53)	7.73 (6.97 to 8.42)	8.77 (7.86 to 9.72)	0.26 (0.21 to 0.33)	0.26 (0.2 to 0.32)	0.26 (0.22 to 0.3)
Kyrgyzstan	12.7 (12.5 to 12.9)	10.9 (10.9 to 11)	12.9 (12.3 to 13.5)	9.48 (8.57 to 10.3)	8.31 (7.58 to 8.96)	9.79 (8.85 to 10.7)	0.25 (0.2 to 0.31)	0.24 (0.18 to 0.3)	0.24 (0.2 to 0.28)
Mongolia	9.85 (9.19 to 10.6)	9.28 (8.72 to 9.93)	10.8 (9.74 to 11.9)	7.48 (6.66 to 8.29)	7.08 (6.32 to 7.87)	8.28 (7.19 to 9.38)	0.24 (0.22 to 0.28)	0.24 (0.21 to 0.27)	0.24 (0.21 to 0.26)
Tajikistan	13.2 (12.9 to 13.6)	9.96 (9.64 to 10.3)	9.61 (8.7 to 10.5)	10 (9.11 to 10.9)	7.65 (6.95 to 8.33)	7.36 (6.44 to 8.3)	0.24 (0.2 to 0.29)	0.23 (0.19 to 0.28)	0.23 (0.21 to 0.26)
Turkmenistan	11.7 (11.6 to 11.8)	10.8 (10.7 to 10.8)	12.4 (11.2 to 13.7)	8.87 (8.07 to 9.57)	8.22 (7.5 to 8.87)	9.48 (8.25 to 10.7)	0.24 (0.19 to 0.3)	0.24 (0.18 to 0.3)	0.24 (0.22 to 0.26)
Uzbekistan	12.7 (12.7 to 12.8)	7.75 (7.61 to 7.9)	8.73 (8.18 to 9.33)	9.71 (8.84 to 10.5)	5.98 (5.47 to 6.44)	6.69 (5.98 to 7.39)	0.24 (0.18 to 0.3)	0.23 (0.18 to 0.28)	0.23 (0.21 to 0.27)
Central Europe	11.3 (11.2 to 11.3)	12.4 (12.4 to 12.4)	13.9 (13.2 to 14.6)	8.15 (7.3 to 8.9)	9.03 (8.1 to 9.85)	10.1 (8.97 to 11.2)	0.28 (0.21 to 0.35)	0.27 (0.21 to 0.34)	0.27 (0.23 to 0.32)
Albania	12.1 (12 to 12.2)	13.2 (13.1 to 13.3)	14.2 (12.7 to 15.8)	9.11 (8.28 to 9.85)	9.96 (9.06 to 10.8)	10.7 (9.3 to 12.2)	0.25 (0.19 to 0.31)	0.25 (0.19 to 0.31)	0.25 (0.23 to 0.27)
Bosnia and Herzegovina	11.9 (11.8 to 12.1)	11.9 (11.8 to 12)	12.8 (11.7 to 14.1)	8.77 (7.91 to 9.54)	8.69 (7.81 to 9.49)	9.28 (8.06 to 10.5)	0.27 (0.21 to 0.33)	0.27 (0.21 to 0.34)	0.28 (0.25 to 0.31)
Bulgaria	10.3 (10.2 to 10.5)	10.7 (10.6 to 10.9)	11.9 (10.9 to 13)	7.67 (6.93 to 8.35)	7.99 (7.23 to 8.69)	8.86 (7.75 to 10)	0.26 (0.2 to 0.32)	0.26 (0.2 to 0.32)	0.26 (0.23 to 0.29)
Croatia	11.3 (11 to 11.5)	12.5 (12.4 to 12.6)	14.2 (13 to 15.4)	8.07 (7.2 to 8.87)	8.96 (7.99 to 9.85)	10 (8.71 to 11.4)	0.28 (0.23 to 0.35)	0.28 (0.22 to 0.36)	0.29 (0.26 to 0.33)
Czechia	10.8 (10.8 to 10.9)	13.1 (13 to 13.2)	14.8 (13.8 to 15.9)	7.69 (6.83 to 8.46)	9.28 (8.23 to 10.2)	10.4 (9.09 to 11.8)	0.29 (0.22 to 0.37)	0.29 (0.22 to 0.37)	0.3 (0.26 to 0.34)
Hungary	11.1 (11 to 11.1)	12.5 (12.4 to 12.5)	13.9 (12.9 to 15)	7.81 (6.94 to 8.6)	8.87 (7.9 to 9.76)	9.98 (8.74 to 11.2)	0.29 (0.22 to 0.37)	0.29 (0.22 to 0.36)	0.28 (0.25 to 0.32)
Montenegro	13.2 (12.9 to 13.4)	12.1 (11.9 to 12.3)	12.5 (11.7 to 13.3)	9.73 (8.77 to 10.7)	8.97 (8.08 to 9.77)	9.16 (8.08 to 10.2)	0.26 (0.21 to 0.32)	0.26 (0.21 to 0.32)	0.27 (0.23 to 0.31)
North Macedonia	10.6 (10.4 to 10.7)	10.2 (10 to 10.3)	10.9 (9.99 to 11.9)	7.78 (7 to 8.5)	7.44 (6.68 to 8.13)	7.99 (6.96 to 9.05)	0.27 (0.21 to 0.33)	0.27 (0.21 to 0.33)	0.27 (0.24 to 0.3)
Poland	11.7 (11.6 to 11.7)	13.5 (13.4 to 13.5)	14.9 (14 to 15.9)	8.4 (7.5 to 9.21)	9.78 (8.75 to 10.7)	10.9 (9.54 to 12.1)	0.28 (0.21 to 0.36)	0.27 (0.21 to 0.35)	0.27 (0.24 to 0.32)
Romania	11.1 (11.1 to 11.2)	11.9 (11.9 to 12)	13.4 (12.5 to 14.5)	8.2 (7.4 to 8.92)	8.89 (8.06 to 9.65)	9.97 (8.78 to 11.1)	0.26 (0.2 to 0.33)	0.25 (0.19 to 0.32)	0.26 (0.23 to 0.3)
Serbia	10.9 (10.8 to 10.9)	10.7 (10.6 to 10.7)	12.2 (11.2 to 13.1)	7.98 (7.22 to 8.68)	7.86 (7.09 to 8.55)	8.85 (7.75 to 9.96)	0.26 (0.2 to 0.33)	0.26 (0.2 to 0.33)	0.27 (0.24 to 0.31)
Slovakia	11.4 (11.3 to 11.5)	12.6 (12.5 to 12.7)	14.1 (12.9 to 15.4)	8.33 (7.49 to 9.09)	9.2 (8.24 to 10)	10.3 (8.92 to 11.6)	0.27 (0.21 to 0.34)	0.27 (0.21 to 0.34)	0.27 (0.24 to 0.31)
Slovenia	12.4 (11 to 13.8)	14.5 (14.4 to 14.7)	16.3 (14.7 to 17.7)	8.8 (7.53 to 10.2)	10.3 (9.07 to 11.3)	11.6 (9.95 to 13.2)	0.29 (0.26 to 0.32)	0.29 (0.23 to 0.37)	0.29 (0.25 to 0.33)
Eastern Europe	11.8 (11.7 to 11.8)	11.1 (11.1 to 11.1)	13.3 (12.8 to 13.9)	8.49 (7.59 to 9.29)	8.09 (7.24 to 8.83)	9.73 (8.62 to 10.7)	0.28 (0.21 to 0.35)	0.27 (0.21 to 0.35)	0.27 (0.22 to 0.33)
Belarus	12.4 (12.3 to 12.4)	11.5 (11.5 to 11.6)	13 (11.7 to 14.3)	9.07 (8.15 to 9.89)	8.47 (7.63 to 9.21)	9.63 (8.27 to 11)	0.27 (0.2 to 0.34)	0.26 (0.2 to 0.33)	0.26 (0.23 to 0.29)
Estonia	11.5 (11.4 to 11.7)	13 (12.9 to 13.2)	15 (13.8 to 16.4)	8.5 (7.67 to 9.24)	9.63 (8.65 to 10.5)	11.1 (9.73 to 12.5)	0.26 (0.21 to 0.33)	0.26 (0.21 to 0.33)	0.26 (0.23 to 0.3)
Latvia	11.8 (11.7 to 11.9)	12.4 (12.3 to 12.5)	14.2 (13.2 to 15.1)	8.55 (7.65 to 9.36)	9.07 (8.12 to 9.9)	10.4 (9.17 to 11.6)	0.27 (0.21 to 0.35)	0.27 (0.21 to 0.34)	0.27 (0.23 to 0.3)
Lithuania	12.5 (12.4 to 12.6)	12.9 (12.8 to 13)	14.5 (13.6 to 15.6)	9.12 (8.16 to 9.97)	9.33 (8.38 to 10.2)	10.7 (9.38 to 11.9)	0.27 (0.21 to 0.34)	0.28 (0.21 to 0.34)	0.27 (0.24 to 0.31)
Republic of Moldova	11.1 (11 to 11.1)	11.2 (11.1 to 11.3)	13.7 (13 to 14.4)	8.13 (7.32 to 8.87)	8.29 (7.49 to 9.01)	10.2 (9.07 to 11.3)	0.26 (0.2 to 0.33)	0.26 (0.2 to 0.33)	0.26 (0.22 to 0.3)
Russian Federation	11.7 (11.7 to 11.7)	11 (11 to 11)	13.3 (12.6 to 14.1)	8.44 (7.54 to 9.24)	7.99 (7.13 to 8.73)	9.69 (8.48 to 10.7)	0.28 (0.21 to 0.36)	0.28 (0.21 to 0.35)	0.27 (0.24 to 0.33)
Ukraine	11.7 (11.7 to 11.7)	11 (11 to 11)	13.1 (12.4 to 13.8)	8.49 (7.61 to 9.27)	8.12 (7.31 to 8.84)	9.67 (8.64 to 10.7)	0.28 (0.21 to 0.35)	0.26 (0.2 to 0.33)	0.26 (0.22 to 0.3)

Supplementary Table 6: Life expectancy, Health-adjusted life expectancy, and proportion of years spent in ill health in all reporting locations, in 1990, 2005, 2019									
	LE-70			HALE-70			PYIH-70		
location	1990	2005	2019	1990	2005	2019	1990	2005	2019
**High-income**	13.8 (13.8 to 13.8)	15.6 (15.6 to 15.6)	16.7 (16.7 to 16.7)	9.87 (8.82 to 10.8)	11.1 (9.92 to 12.2)	11.8 (10.6 to 13)	0.29 (0.22 to 0.36)	0.29 (0.22 to 0.36)	0.29 (0.22 to 0.37)
Australasia	13.8 (13.7 to 13.8)	16.1 (16 to 16.1)	17.1 (17 to 17.2)	9.8 (8.74 to 10.8)	11.3 (10.1 to 12.5)	12 (10.6 to 13.2)	0.29 (0.22 to 0.36)	0.3 (0.23 to 0.37)	0.3 (0.23 to 0.37)
Australia	13.8 (13.8 to 13.9)	16.2 (16.1 to 16.2)	17.2 (17.1 to 17.3)	9.86 (8.79 to 10.8)	11.4 (10.1 to 12.5)	12.1 (10.7 to 13.3)	0.29 (0.22 to 0.36)	0.3 (0.23 to 0.37)	0.3 (0.23 to 0.37)
New Zealand	13.4 (13.4 to 13.5)	15.4 (15.4 to 15.5)	16.5 (16.4 to 16.7)	9.53 (8.49 to 10.5)	11 (9.84 to 12.1)	11.7 (10.4 to 12.9)	0.29 (0.22 to 0.36)	0.29 (0.22 to 0.36)	0.29 (0.23 to 0.37)
High-income Asia Pacific	14.4 (14.4 to 14.4)	16.9 (16.9 to 16.9)	18.3 (18.3 to 18.4)	10.6 (9.53 to 11.5)	12.4 (11.2 to 13.5)	13.5 (12.2 to 14.7)	0.27 (0.2 to 0.34)	0.27 (0.2 to 0.34)	0.26 (0.2 to 0.33)
Brunei Darussalam	8.98 (8.66 to 9.35)	10.3 (9.94 to 10.7)	11.4 (10.8 to 11.8)	6.34 (5.65 to 7.03)	7.32 (6.47 to 8.13)	8.14 (7.25 to 9)	0.29 (0.25 to 0.35)	0.29 (0.24 to 0.35)	0.28 (0.24 to 0.33)
Japan	14.9 (14.9 to 14.9)	17.3 (17.2 to 17.3)	18.6 (18.5 to 18.7)	10.9 (9.84 to 11.9)	12.7 (11.4 to 13.8)	13.7 (12.4 to 14.9)	0.27 (0.2 to 0.34)	0.27 (0.2 to 0.34)	0.26 (0.2 to 0.33)
Republic of Korea	11.7 (11.7 to 11.7)	14.6 (14.6 to 14.6)	17 (16.7 to 17.2)	8.5 (7.66 to 9.29)	10.7 (9.6 to 11.6)	12.4 (11.2 to 13.5)	0.27 (0.21 to 0.34)	0.27 (0.21 to 0.34)	0.27 (0.21 to 0.33)
Singapore	12.9 (12.8 to 12.9)	16.1 (16 to 16.1)	18.2 (18.1 to 18.4)	9.46 (8.55 to 10.3)	11.9 (10.8 to 12.9)	13.7 (12.4 to 14.8)	0.26 (0.2 to 0.33)	0.26 (0.2 to 0.32)	0.25 (0.19 to 0.31)
High-income North America	14.3 (14.3 to 14.3)	15.2 (15.2 to 15.2)	16 (16 to 16.1)	9.72 (8.55 to 10.8)	10.3 (9.05 to 11.4)	10.5 (9.18 to 11.8)	0.32 (0.24 to 0.4)	0.32 (0.25 to 0.4)	0.34 (0.27 to 0.43)
Canada	14.3 (14.3 to 14.3)	15.9 (15.9 to 16)	17.1 (17 to 17.2)	10.3 (9.24 to 11.3)	11.5 (10.3 to 12.5)	12.1 (10.9 to 13.3)	0.28 (0.21 to 0.35)	0.28 (0.21 to 0.35)	0.29 (0.23 to 0.36)
Greenland	10.3 (9.89 to 10.7)	11.8 (11.3 to 12.1)	13 (12.2 to 14)	7.1 (6.3 to 7.9)	8.21 (7.26 to 9.03)	8.99 (7.85 to 10.2)	0.31 (0.26 to 0.36)	0.3 (0.25 to 0.36)	0.31 (0.27 to 0.36)
United States of America	14.3 (14.3 to 14.3)	15.1 (15.1 to 15.1)	15.9 (15.9 to 16)	9.66 (8.48 to 10.7)	10.1 (8.89 to 11.3)	10.4 (9 to 11.6)	0.32 (0.25 to 0.4)	0.33 (0.26 to 0.41)	0.35 (0.27 to 0.43)
Southern Latin America	12.8 (12.8 to 12.8)	14 (13.9 to 14)	14.6 (14.5 to 14.8)	9.56 (8.65 to 10.4)	10.4 (9.37 to 11.3)	10.8 (9.73 to 11.8)	0.25 (0.19 to 0.32)	0.26 (0.19 to 0.33)	0.26 (0.2 to 0.33)
Argentina	12.8 (12.7 to 12.8)	13.8 (13.7 to 13.8)	14.1 (14 to 14.2)	9.56 (8.66 to 10.4)	10.3 (9.27 to 11.1)	10.5 (9.48 to 11.4)	0.25 (0.19 to 0.32)	0.26 (0.19 to 0.33)	0.26 (0.2 to 0.32)
Chile	12.8 (12.8 to 12.8)	14.6 (14.6 to 14.7)	15.9 (15.8 to 16.1)	9.45 (8.53 to 10.3)	10.7 (9.61 to 11.7)	11.5 (10.3 to 12.6)	0.26 (0.2 to 0.33)	0.27 (0.2 to 0.34)	0.28 (0.22 to 0.34)
Uruguay	13 (12.9 to 13)	13.8 (13.7 to 13.9)	14.8 (14.6 to 15)	9.7 (8.81 to 10.5)	10.3 (9.35 to 11.2)	10.9 (9.85 to 11.9)	0.25 (0.19 to 0.32)	0.25 (0.19 to 0.32)	0.26 (0.21 to 0.33)
Western Europe	13.5 (13.4 to 13.5)	15.4 (15.4 to 15.4)	16.5 (16.5 to 16.6)	9.81 (8.82 to 10.7)	11.2 (10 to 12.2)	12 (10.8 to 13.1)	0.27 (0.21 to 0.34)	0.27 (0.21 to 0.35)	0.28 (0.21 to 0.35)
Andorra	14.8 (13.2 to 16.1)	16.5 (15.1 to 17.4)	16.7 (15.4 to 18.2)	10.9 (9.4 to 12.4)	12 (10.5 to 13.3)	12.2 (10.6 to 13.7)	0.26 (0.23 to 0.29)	0.27 (0.24 to 0.3)	0.27 (0.25 to 0.31)
Austria	13.2 (13.2 to 13.3)	15.2 (15.1 to 15.2)	16.6 (16.5 to 16.7)	9.64 (8.66 to 10.5)	10.9 (9.77 to 11.9)	12 (10.7 to 13.1)	0.27 (0.21 to 0.34)	0.28 (0.22 to 0.35)	0.28 (0.22 to 0.35)
Belgium	13.2 (13.2 to 13.3)	15 (15 to 15.1)	16.2 (16.1 to 16.4)	9.65 (8.67 to 10.5)	10.8 (9.64 to 11.8)	11.6 (10.4 to 12.8)	0.27 (0.21 to 0.34)	0.28 (0.22 to 0.36)	0.28 (0.22 to 0.36)
Cyprus	11.3 (11.2 to 11.4)	13 (12.9 to 13.2)	14.8 (14.4 to 15.3)	8.37 (7.54 to 9.11)	9.46 (8.49 to 10.3)	10.8 (9.67 to 11.8)	0.26 (0.2 to 0.33)	0.27 (0.21 to 0.34)	0.27 (0.23 to 0.33)
Denmark	13 (13 to 13.1)	14.2 (14.2 to 14.3)	15.8 (15.7 to 16)	9.5 (8.54 to 10.4)	10.4 (9.41 to 11.4)	11.6 (10.5 to 12.6)	0.27 (0.21 to 0.34)	0.27 (0.21 to 0.34)	0.27 (0.21 to 0.33)
Finland	13.1 (13 to 13.2)	15.4 (15.3 to 15.5)	16.6 (16.4 to 16.8)	9.51 (8.51 to 10.4)	11 (9.78 to 12.1)	12 (10.7 to 13.1)	0.27 (0.21 to 0.35)	0.29 (0.22 to 0.36)	0.28 (0.22 to 0.35)
France	14.4 (14.4 to 14.5)	16.3 (16.3 to 16.3)	17.7 (17.6 to 17.8)	10.6 (9.56 to 11.6)	12.1 (10.9 to 13.1)	13.1 (11.8 to 14.2)	0.26 (0.2 to 0.34)	0.26 (0.2 to 0.33)	0.26 (0.2 to 0.33)
Germany	13 (13 to 13)	15.1 (15.1 to 15.1)	15.9 (15.8 to 16)	9.5 (8.57 to 10.3)	11 (9.89 to 12)	11.5 (10.3 to 12.6)	0.27 (0.2 to 0.34)	0.27 (0.21 to 0.34)	0.28 (0.21 to 0.35)
Greece	13.3 (13.3 to 13.4)	14.5 (14.5 to 14.6)	15.8 (15.7 to 16)	9.82 (8.9 to 10.7)	10.6 (9.61 to 11.6)	11.6 (10.6 to 12.7)	0.26 (0.2 to 0.33)	0.27 (0.21 to 0.34)	0.26 (0.21 to 0.33)
Iceland	14.3 (14.1 to 14.5)	16 (15.6 to 16.3)	17.9 (17.4 to 18.4)	10.5 (9.46 to 11.4)	11.5 (10.4 to 12.7)	13 (11.7 to 14.3)	0.27 (0.21 to 0.33)	0.28 (0.22 to 0.34)	0.27 (0.23 to 0.33)
Ireland	12 (11.9 to 12.1)	14.4 (14.3 to 14.5)	16.1 (15.9 to 16.3)	8.87 (8.01 to 9.63)	10.5 (9.48 to 11.5)	11.7 (10.6 to 12.8)	0.26 (0.2 to 0.33)	0.27 (0.21 to 0.34)	0.27 (0.22 to 0.33)
Israel	13.6 (13.6 to 13.7)	15.2 (15.2 to 15.3)	16.9 (16.7 to 17)	10.2 (9.22 to 11)	11.2 (10.1 to 12.2)	12.5 (11.3 to 13.5)	0.25 (0.19 to 0.32)	0.27 (0.2 to 0.33)	0.26 (0.2 to 0.32)
Italy	13.8 (13.8 to 13.8)	15.7 (15.7 to 15.7)	16.9 (16.9 to 17)	9.81 (8.72 to 10.8)	11.2 (9.95 to 12.3)	12.1 (10.8 to 13.3)	0.29 (0.22 to 0.37)	0.29 (0.22 to 0.37)	0.28 (0.22 to 0.36)
Luxembourg	12.9 (12.7 to 13)	14.9 (14.7 to 15.2)	17.1 (16.4 to 17.7)	9.43 (8.49 to 10.3)	10.8 (9.67 to 11.8)	12.2 (10.8 to 13.5)	0.27 (0.21 to 0.33)	0.28 (0.22 to 0.34)	0.29 (0.24 to 0.34)
Malta	12.8 (12.6 to 12.9)	14.8 (14.6 to 15)	16.8 (16.3 to 17.4)	9.4 (8.48 to 10.2)	10.8 (9.75 to 11.8)	12.3 (11 to 13.5)	0.26 (0.21 to 0.33)	0.27 (0.21 to 0.33)	0.27 (0.23 to 0.33)
Monaco	14.3 (13.5 to 15.7)	14.8 (14.1 to 16.2)	15.5 (14.8 to 16.6)	10.7 (9.47 to 12.1)	11 (9.7 to 12.3)	11.4 (10.1 to 12.7)	0.26 (0.23 to 0.3)	0.26 (0.24 to 0.31)	0.27 (0.24 to 0.32)
Netherlands	13.5 (13.5 to 13.6)	14.7 (14.7 to 14.8)	16 (15.8 to 16.1)	9.98 (9.05 to 10.9)	10.8 (9.82 to 11.8)	11.5 (10.4 to 12.6)	0.26 (0.2 to 0.33)	0.26 (0.2 to 0.33)	0.28 (0.22 to 0.34)
Norway	13.6 (13.5 to 13.6)	15.3 (15.3 to 15.4)	16.7 (16.6 to 16.8)	9.7 (8.62 to 10.6)	10.9 (9.74 to 12)	11.8 (10.5 to 12.9)	0.29 (0.22 to 0.36)	0.29 (0.22 to 0.36)	0.3 (0.23 to 0.37)
Portugal	12.4 (12.4 to 12.4)	14.7 (14.6 to 14.7)	16.3 (16.2 to 16.5)	8.99 (8.09 to 9.83)	10.6 (9.51 to 11.6)	11.9 (10.7 to 13)	0.27 (0.21 to 0.34)	0.28 (0.21 to 0.35)	0.27 (0.21 to 0.34)
San Marino	15.1 (14.3 to 15.9)	16.1 (14.6 to 17.5)	16.6 (14.6 to 18.9)	11.1 (9.97 to 12.3)	11.9 (10.3 to 13.4)	12.2 (10.3 to 14.3)	0.26 (0.23 to 0.3)	0.26 (0.23 to 0.3)	0.26 (0.24 to 0.29)
Spain	13.9 (13.9 to 13.9)	15.7 (15.7 to 15.7)	17.2 (17 to 17.3)	10.1 (9.1 to 11)	11.4 (10.3 to 12.5)	12.5 (11.2 to 13.6)	0.27 (0.21 to 0.34)	0.27 (0.21 to 0.34)	0.27 (0.21 to 0.34)

Supplementary Table 6: Life expectancy, Health-adjusted life expectancy, and proportion of years spent in ill health in all reporting locations, in 1990, 2005, 2019									
location	LE-70			HALE-70			PYIH-70		
	1990	2005	2019	1990	2005	2019	1990	2005	2019
Sweden	14 (14 to 14.1)	15.6 (15.5 to 15.6)	16.7 (16.6 to 16.8)	10.3 (9.28 to 11.2)	11.3 (10.2 to 12.3)	12.1 (10.9 to 13.2)	0.26 (0.2 to 0.34)	0.27 (0.21 to 0.35)	0.27 (0.21 to 0.35)
Switzerland	14.4 (14.4 to 14.5)	16.4 (16.3 to 16.4)	17.6 (17.5 to 17.8)	10.4 (9.32 to 11.4)	11.9 (10.6 to 13)	12.8 (11.4 to 14)	0.28 (0.21 to 0.35)	0.28 (0.21 to 0.35)	0.27 (0.21 to 0.35)
United Kingdom	13 (12.9 to 13)	14.8 (14.8 to 14.8)	15.8 (15.7 to 15.9)	9.48 (8.53 to 10.3)	10.7 (9.62 to 11.7)	11.3 (10.1 to 12.4)	0.27 (0.2 to 0.34)	0.28 (0.21 to 0.35)	0.28 (0.22 to 0.36)
**Latin America and Caribbean**	13.1 (13 to 13.1)	14.6 (14.5 to 14.6)	15.2 (14.7 to 15.7)	9.44 (8.45 to 10.3)	10.6 (9.51 to 11.6)	11 (9.91 to 12.1)	0.28 (0.21 to 0.35)	0.27 (0.21 to 0.34)	0.27 (0.23 to 0.33)
Andean Latin America	13.5 (13.1 to 13.9)	14.8 (14.4 to 15.2)	15.4 (14.4 to 16.5)	10.1 (9.05 to 11)	11 (9.89 to 12)	11.4 (10.1 to 12.7)	0.25 (0.21 to 0.31)	0.26 (0.21 to 0.31)	0.26 (0.23 to 0.3)
Bolivia (Plurinational State of)	10.8 (10.3 to 11.2)	11.9 (11.4 to 12.2)	11.8 (11.1 to 12.6)	7.94 (7.11 to 8.74)	8.79 (7.84 to 9.62)	8.74 (7.7 to 9.83)	0.26 (0.22 to 0.31)	0.26 (0.21 to 0.31)	0.26 (0.22 to 0.31)
Ecuador	13.8 (13.7 to 14)	14.4 (14 to 14.8)	14.6 (13.4 to 15.8)	10.4 (9.39 to 11.2)	10.7 (9.59 to 11.6)	10.8 (9.37 to 12.2)	0.25 (0.2 to 0.32)	0.26 (0.21 to 0.31)	0.26 (0.23 to 0.3)
Peru	14.2 (13.5 to 15)	16.1 (15.3 to 16.9)	17.1 (15.4 to 18.8)	10.6 (9.49 to 11.8)	12 (10.7 to 13.2)	12.7 (11 to 14.4)	0.25 (0.21 to 0.3)	0.25 (0.22 to 0.3)	0.26 (0.23 to 0.29)
Caribbean	13.2 (13.1 to 13.3)	14.1 (13.9 to 14.3)	14.6 (13.8 to 15.4)	9.89 (8.95 to 10.7)	10.5 (9.48 to 11.4)	10.7 (9.54 to 11.8)	0.25 (0.19 to 0.32)	0.26 (0.2 to 0.32)	0.26 (0.23 to 0.31)
Antigua and Barbuda	13.2 (12.9 to 13.5)	13.7 (13.5 to 14)	13.5 (12.9 to 14.1)	10 (9.08 to 10.8)	10.3 (9.25 to 11.2)	10 (8.93 to 11.1)	0.24 (0.2 to 0.3)	0.25 (0.2 to 0.31)	0.26 (0.22 to 0.31)
Bahamas	13.1 (12.8 to 13.5)	14.2 (13.9 to 14.6)	14.3 (13.2 to 15.4)	9.97 (9.07 to 10.9)	10.7 (9.66 to 11.7)	10.7 (9.39 to 12)	0.24 (0.2 to 0.29)	0.25 (0.2 to 0.3)	0.25 (0.22 to 0.29)
Barbados	13.2 (13 to 13.4)	13.6 (13.4 to 13.8)	13.8 (13 to 14.7)	10 (9.12 to 10.8)	10.2 (9.25 to 11.1)	10.3 (9.16 to 11.5)	0.24 (0.19 to 0.3)	0.25 (0.19 to 0.31)	0.25 (0.22 to 0.29)
Belize	13.9 (13.6 to 14.2)	13.9 (13.7 to 14.1)	14.9 (14.2 to 15.7)	10.6 (9.6 to 11.5)	10.5 (9.47 to 11.3)	11.2 (10 to 12.4)	0.24 (0.19 to 0.29)	0.25 (0.19 to 0.31)	0.25 (0.21 to 0.29)
Bermuda	12.1 (11.9 to 12.3)	15.2 (14.5 to 15.9)	16.5 (15.4 to 17.3)	9.23 (8.41 to 9.96)	11.5 (10.3 to 12.6)	12.3 (11 to 13.6)	0.24 (0.19 to 0.29)	0.25 (0.21 to 0.29)	0.25 (0.21 to 0.29)
Cuba	13.7 (13.6 to 13.7)	14.6 (14.5 to 14.7)	15.1 (14 to 16.3)	10.2 (9.18 to 11)	10.8 (9.71 to 11.7)	11 (9.62 to 12.4)	0.26 (0.2 to 0.33)	0.26 (0.2 to 0.33)	0.27 (0.24 to 0.31)
Dominica	11.8 (11.5 to 12.2)	12.5 (12 to 13.1)	12.4 (11.5 to 13.1)	8.89 (8.05 to 9.67)	9.31 (8.36 to 10.2)	9.16 (8.06 to 10.3)	0.24 (0.2 to 0.3)	0.26 (0.22 to 0.3)	0.26 (0.22 to 0.3)
Dominican Republic	15.1 (14.5 to 15.7)	14.6 (13.9 to 15.4)	14 (12.8 to 15.1)	11.5 (10.4 to 12.5)	11.1 (9.99 to 12.1)	10.6 (9.32 to 11.9)	0.24 (0.2 to 0.28)	0.24 (0.21 to 0.28)	0.24 (0.21 to 0.27)
Grenada	11.3 (11 to 11.7)	11.5 (11.3 to 11.7)	12.4 (12.1 to 12.8)	8.55 (7.69 to 9.36)	8.57 (7.73 to 9.33)	9.16 (8.21 to 10)	0.25 (0.2 to 0.3)	0.25 (0.2 to 0.31)	0.26 (0.22 to 0.32)
Guyana	9.39 (8.88 to 10)	10.9 (10.4 to 11.4)	11.4 (10.3 to 12.7)	6.93 (6.15 to 7.67)	8 (7.06 to 8.84)	8.37 (7.19 to 9.64)	0.26 (0.23 to 0.31)	0.26 (0.23 to 0.32)	0.27 (0.24 to 0.3)
Haiti	9.48 (9.23 to 9.87)	10.4 (9.85 to 11)	10.9 (9.65 to 12.1)	7.12 (6.41 to 7.79)	7.76 (6.92 to 8.59)	8.11 (6.86 to 9.29)	0.25 (0.21 to 0.31)	0.25 (0.22 to 0.3)	0.26 (0.23 to 0.29)
Jamaica	14.4 (14.2 to 14.5)	15.7 (15.3 to 16)	14.6 (13.3 to 16)	10.9 (9.94 to 11.8)	11.7 (10.5 to 12.7)	10.8 (9.48 to 12.3)	0.24 (0.19 to 0.3)	0.25 (0.2 to 0.31)	0.26 (0.23 to 0.29)
Puerto Rico	14 (13.9 to 14.1)	15.8 (15.7 to 16)	17.4 (15.9 to 18.9)	10.4 (9.44 to 11.4)	11.6 (10.5 to 12.7)	12.7 (11.1 to 14.4)	0.25 (0.19 to 0.32)	0.27 (0.2 to 0.33)	0.27 (0.24 to 0.3)
Saint Kitts and Nevis	9.52 (9.25 to 9.8)	11.8 (11.5 to 12.1)	12 (11.4 to 12.4)	7.12 (6.43 to 7.78)	8.71 (7.81 to 9.54)	8.84 (7.84 to 9.73)	0.25 (0.21 to 0.3)	0.26 (0.21 to 0.32)	0.27 (0.21 to 0.31)
Saint Lucia	11.4 (11.2 to 11.7)	13.6 (13.4 to 13.8)	14 (13.2 to 14.8)	8.44 (7.59 to 9.24)	9.99 (8.98 to 10.9)	10.2 (8.96 to 11.3)	0.26 (0.21 to 0.32)	0.27 (0.21 to 0.33)	0.27 (0.23 to 0.32)
Saint Vincent and the Grenadines	11.6 (11.4 to 11.9)	13.5 (13.3 to 13.7)	12.6 (12.1 to 13.2)	8.74 (7.93 to 9.51)	10.1 (9.07 to 10.9)	9.35 (8.3 to 10.3)	0.25 (0.2 to 0.31)	0.26 (0.2 to 0.32)	0.26 (0.22 to 0.31)
Suriname	12.9 (12.7 to 13.1)	13.5 (13.3 to 13.7)	14 (13.1 to 15)	9.68 (8.76 to 10.5)	9.93 (8.95 to 10.9)	10.2 (8.99 to 11.4)	0.25 (0.2 to 0.31)	0.26 (0.21 to 0.33)	0.27 (0.24 to 0.31)
Trinidad and Tobago	11.6 (11.4 to 11.7)	13.4 (13.2 to 13.5)	14.5 (12.9 to 16.2)	8.66 (7.8 to 9.4)	9.94 (8.97 to 10.9)	10.7 (9.14 to 12.3)	0.25 (0.2 to 0.32)	0.26 (0.2 to 0.32)	0.26 (0.24 to 0.29)
United States Virgin Islands	12.9 (12.4 to 13.7)	13.4 (12.9 to 13.8)	12.9 (12.6 to 13.5)	9.87 (8.92 to 10.8)	10.1 (9.08 to 10.9)	9.64 (8.68 to 10.6)	0.24 (0.21 to 0.28)	0.25 (0.21 to 0.3)	0.25 (0.22 to 0.31)
Central Latin America	13.3 (13.3 to 13.4)	14.7 (14.7 to 14.8)	15.4 (14.6 to 16.1)	9.55 (8.52 to 10.5)	10.7 (9.58 to 11.7)	11.1 (9.82 to 12.3)	0.28 (0.22 to 0.36)	0.27 (0.21 to 0.35)	0.28 (0.24 to 0.33)
Colombia	13.7 (13.6 to 13.7)	15.8 (15.8 to 15.9)	17.5 (15.9 to 19.1)	9.93 (8.89 to 10.8)	11.5 (10.4 to 12.6)	12.8 (11.2 to 14.5)	0.27 (0.21 to 0.35)	0.27 (0.21 to 0.34)	0.27 (0.24 to 0.3)
Costa Rica	14.9 (14.6 to 15.1)	16.1 (16 to 16.3)	16.6 (15.2 to 18.1)	10.8 (9.69 to 11.8)	11.8 (10.5 to 12.9)	12.1 (10.5 to 13.7)	0.27 (0.22 to 0.34)	0.27 (0.21 to 0.34)	0.27 (0.24 to 0.31)
El Salvador	15.1 (15 to 15.2)	15.3 (15.2 to 15.4)	15.5 (14 to 17.2)	11 (9.87 to 12)	11.2 (9.99 to 12.2)	11.3 (9.73 to 12.9)	0.27 (0.21 to 0.34)	0.27 (0.21 to 0.34)	0.27 (0.25 to 0.3)
Guatemala	10.4 (9.92 to 10.9)	12.5 (12 to 13.1)	13.9 (12.7 to 15)	7.62 (6.78 to 8.45)	9.06 (8.07 to 10)	10 (8.75 to 11.3)	0.27 (0.22 to 0.32)	0.28 (0.23 to 0.33)	0.28 (0.24 to 0.31)
Honduras	14.1 (13.5 to 14.9)	12.9 (11.7 to 14.2)	11.9 (11.4 to 12.1)	10.3 (9.18 to 11.5)	9.53 (8.21 to 10.8)	8.75 (7.84 to 9.56)	0.27 (0.23 to 0.32)	0.26 (0.24 to 0.3)	0.27 (0.21 to 0.31)
Mexico	13.1 (13.1 to 13.2)	14.5 (14.4 to 14.5)	14.9 (14.4 to 15.4)	9.24 (8.19 to 10.2)	10.4 (9.32 to 11.4)	10.6 (9.43 to 11.8)	0.3 (0.23 to 0.38)	0.28 (0.21 to 0.35)	0.29 (0.24 to 0.35)
Nicaragua	15.3 (15.1 to 15.5)	12.2 (12.1 to 12.3)	13 (12.3 to 13.8)	11.1 (9.94 to 12.1)	9.05 (8.18 to 9.84)	9.54 (8.48 to 10.6)	0.28 (0.22 to 0.34)	0.26 (0.2 to 0.32)	0.26 (0.23 to 0.31)
Panama	15.6 (15.4 to 15.8)	16.3 (16.1 to 16.5)	17.3 (15.8 to 18.9)	11.4 (10.2 to 12.4)	11.9 (10.8 to 13)	12.6 (11 to 14.3)	0.27 (0.21 to 0.33)	0.27 (0.21 to 0.33)	0.27 (0.24 to 0.31)
Venezuela (Bolivarian Republic of)	13.8 (13.7 to 13.8)	15 (14.9 to 15)	14.8 (13.2 to 16.4)	10 (8.99 to 10.9)	10.9 (9.83 to 11.9)	10.9 (9.34 to 12.5)	0.27 (0.21 to 0.34)	0.27 (0.21 to 0.34)	0.27 (0.24 to 0.29)
Tropical Latin America	12.6 (12.5 to 12.6)	14.4 (14.4 to 14.5)	15.3 (15.1 to 15.4)	8.98 (8.01 to 9.86)	10.4 (9.32 to 11.4)	11.1 (9.94 to 12.1)	0.28 (0.22 to 0.36)	0.28 (0.21 to 0.35)	0.27 (0.22 to 0.34)

Supplementary Table 6: Life expectancy, Health-adjusted life expectancy, and proportion of years spent in ill health in all reporting locations, in 1990, 2005, 2019

location	LE-70			HALE-70			PYIH-70		
	1990	2005	2019	1990	2005	2019	1990	2005	2019
Brazil	12.5 (12.4 to 12.6)	14.4 (14.3 to 14.5)	15.3 (15.1 to 15.4)	8.92 (7.96 to 9.8)	10.4 (9.3 to 11.4)	11.1 (9.94 to 12.1)	0.29 (0.22 to 0.36)	0.28 (0.21 to 0.35)	0.27 (0.22 to 0.34)
Paraguay	15.3 (14.8 to 15.9)	15.2 (14.7 to 15.6)	15.1 (13.6 to 16.7)	11.3 (10.2 to 12.5)	11.2 (10.1 to 12.3)	11.2 (9.68 to 12.8)	0.26 (0.22 to 0.31)	0.26 (0.21 to 0.31)	0.26 (0.24 to 0.29)
**North Africa and Middle East**	11.3 (11.1 to 11.5)	12.4 (12.2 to 12.7)	13 (12.5 to 13.4)	8.23 (7.38 to 9.04)	9.01 (8.11 to 9.88)	9.33 (8.35 to 10.3)	0.27 (0.21 to 0.33)	0.28 (0.22 to 0.33)	0.28 (0.23 to 0.33)
North Africa and Middle East	11.3 (11.1 to 11.5)	12.4 (12.2 to 12.7)	13 (12.5 to 13.4)	8.23 (7.38 to 9.04)	9.01 (8.11 to 9.88)	9.33 (8.35 to 10.3)	0.27 (0.21 to 0.33)	0.28 (0.22 to 0.33)	0.28 (0.23 to 0.33)
Afghanistan	9.15 (8.5 to 9.88)	9.37 (8.55 to 10.2)	9.77 (9.11 to 10.8)	6.49 (5.63 to 7.29)	6.61 (5.68 to 7.52)	6.75 (5.82 to 7.76)	0.29 (0.26 to 0.34)	0.29 (0.26 to 0.34)	0.31 (0.28 to 0.36)
Algeria	10.6 (9.96 to 11.3)	12.3 (11.5 to 13.1)	13.3 (12.6 to 14.1)	7.9 (6.96 to 8.83)	9.06 (8.1 to 10.1)	9.71 (8.58 to 10.8)	0.26 (0.22 to 0.3)	0.26 (0.23 to 0.3)	0.27 (0.24 to 0.32)
Bahrain	9.1 (8.64 to 9.6)	9.54 (9.1 to 9.95)	12 (11.1 to 13)	6.48 (5.74 to 7.21)	6.7 (5.93 to 7.48)	8.36 (7.24 to 9.51)	0.29 (0.25 to 0.34)	0.3 (0.25 to 0.35)	0.3 (0.27 to 0.35)
Egypt	10.1 (10 to 10.1)	10.2 (10.1 to 10.2)	10.7 (9.62 to 11.9)	7.51 (6.81 to 8.14)	7.55 (6.84 to 8.2)	7.88 (6.81 to 9.02)	0.25 (0.19 to 0.32)	0.26 (0.2 to 0.33)	0.27 (0.24 to 0.29)
Iran (Islamic Republic of)	11.8 (11.7 to 12)	13.1 (13 to 13.2)	14.3 (14.2 to 14.5)	8.42 (7.52 to 9.26)	9.41 (8.44 to 10.3)	10.2 (9.12 to 11.2)	0.29 (0.23 to 0.36)	0.28 (0.22 to 0.35)	0.29 (0.22 to 0.36)
Iraq	11.8 (11 to 12.6)	11.5 (10.5 to 12.6)	12.5 (11.8 to 13.5)	8.21 (7.19 to 9.24)	8.25 (7.1 to 9.41)	9.01 (7.98 to 10.2)	0.3 (0.27 to 0.35)	0.29 (0.25 to 0.32)	0.28 (0.25 to 0.33)
Jordan	11.4 (10.8 to 12)	12.3 (11.8 to 12.8)	14.5 (13.6 to 15.4)	8.34 (7.47 to 9.24)	8.93 (7.93 to 9.85)	10.5 (9.32 to 11.7)	0.27 (0.23 to 0.31)	0.27 (0.23 to 0.33)	0.27 (0.24 to 0.32)
Kuwait	14.7 (14.5 to 14.9)	15.5 (15.3 to 15.6)	16.4 (15.5 to 17.4)	10.7 (9.65 to 11.8)	11.1 (9.96 to 12.2)	11.8 (10.4 to 13.2)	0.27 (0.21 to 0.33)	0.28 (0.22 to 0.35)	0.28 (0.24 to 0.33)
Lebanon	11.6 (10.9 to 12.1)	12.7 (12.3 to 13.2)	13.5 (13 to 14)	8.45 (7.51 to 9.33)	9.2 (8.24 to 10.1)	9.54 (8.43 to 10.6)	0.27 (0.23 to 0.31)	0.28 (0.23 to 0.33)	0.29 (0.24 to 0.35)
Libya	13.9 (12.9 to 14.8)	15.3 (15.1 to 15.5)	14.5 (13.3 to 15.8)	10 (8.89 to 11.2)	10.9 (9.72 to 12)	10.3 (8.9 to 11.7)	0.28 (0.24 to 0.31)	0.29 (0.22 to 0.36)	0.29 (0.26 to 0.33)
Morocco	11.5 (11.2 to 11.8)	12.2 (11.3 to 13)	11.9 (11.4 to 13)	8.49 (7.66 to 9.3)	8.92 (7.86 to 10)	8.69 (7.74 to 9.84)	0.26 (0.21 to 0.31)	0.27 (0.23 to 0.31)	0.27 (0.24 to 0.32)
Oman	9.5 (8.62 to 10.6)	10.6 (10.3 to 10.8)	10.7 (10.3 to 11.1)	6.87 (5.94 to 7.9)	7.55 (6.69 to 8.33)	7.6 (6.73 to 8.4)	0.28 (0.25 to 0.31)	0.28 (0.23 to 0.35)	0.29 (0.24 to 0.34)
Palestine	10.5 (9.47 to 11.6)	11.4 (11 to 11.7)	11.8 (11.1 to 12.5)	7.64 (6.61 to 8.71)	8.25 (7.37 to 9.08)	8.45 (7.44 to 9.45)	0.27 (0.25 to 0.3)	0.28 (0.23 to 0.33)	0.28 (0.25 to 0.33)
Qatar	9.56 (8.91 to 10.3)	9.91 (9.26 to 10.6)	10.9 (9.97 to 11.9)	6.82 (5.97 to 7.7)	6.87 (5.97 to 7.72)	7.65 (6.55 to 8.76)	0.29 (0.25 to 0.33)	0.31 (0.27 to 0.35)	0.3 (0.26 to 0.34)
Saudi Arabia	11.1 (10.4 to 11.9)	11.7 (11.4 to 12.1)	13.2 (12.5 to 14)	7.73 (6.78 to 8.75)	8.18 (7.17 to 9.1)	9.11 (7.94 to 10.2)	0.3 (0.27 to 0.35)	0.3 (0.24 to 0.37)	0.31 (0.27 to 0.37)
Sudan	10.4 (9.93 to 10.9)	11.3 (10.5 to 12.4)	11.9 (11.1 to 12.5)	7.66 (6.84 to 8.46)	8.28 (7.31 to 9.3)	8.65 (7.62 to 9.59)	0.27 (0.23 to 0.31)	0.27 (0.25 to 0.3)	0.27 (0.23 to 0.31)
Syrian Arab Republic	11.6 (10.7 to 12.6)	12.3 (11.7 to 12.9)	11.9 (10.7 to 13)	8.58 (7.54 to 9.59)	9.08 (8.03 to 10)	8.55 (7.31 to 9.77)	0.26 (0.24 to 0.3)	0.26 (0.23 to 0.32)	0.28 (0.25 to 0.32)
Tunisia	12.8 (12.3 to 13.4)	13.2 (12 to 14.5)	14.1 (12.6 to 15.6)	9.45 (8.38 to 10.4)	9.6 (8.37 to 11)	10.1 (8.69 to 11.6)	0.26 (0.23 to 0.32)	0.27 (0.24 to 0.31)	0.28 (0.26 to 0.31)
Turkey	12.6 (12.1 to 13.1)	15.1 (14.6 to 15.6)	14.6 (13.5 to 15.9)	9.25 (8.25 to 10.2)	10.8 (9.7 to 11.9)	10.5 (9.18 to 11.9)	0.27 (0.22 to 0.32)	0.28 (0.23 to 0.34)	0.28 (0.25 to 0.32)
United Arab Emirates	9.42 (8.96 to 9.92)	8.19 (7.76 to 8.68)	12.1 (11.2 to 12.9)	6.76 (6.01 to 7.51)	5.85 (5.15 to 6.52)	8.5 (7.37 to 9.59)	0.28 (0.24 to 0.33)	0.29 (0.25 to 0.34)	0.3 (0.26 to 0.34)
Yemen	10.4 (9.55 to 11.1)	11.1 (10.3 to 12)	11.4 (10.5 to 12)	7.57 (6.64 to 8.46)	8.16 (7.2 to 9.16)	8.35 (7.24 to 9.31)	0.27 (0.24 to 0.31)	0.27 (0.24 to 0.3)	0.27 (0.23 to 0.31)
**South Asia**	9.49 (9.29 to 9.69)	10.8 (10.5 to 11)	12.4 (11.8 to 13)	6.51 (5.77 to 7.24)	7.37 (6.5 to 8.18)	8.46 (7.41 to 9.5)	0.31 (0.25 to 0.38)	0.31 (0.26 to 0.38)	0.32 (0.27 to 0.37)
South Asia	9.49 (9.29 to 9.69)	10.8 (10.5 to 11)	12.4 (11.8 to 13)	6.51 (5.77 to 7.24)	7.37 (6.5 to 8.18)	8.46 (7.41 to 9.5)	0.31 (0.25 to 0.38)	0.31 (0.26 to 0.38)	0.32 (0.27 to 0.37)
Bangladesh	10.4 (10.1 to 10.8)	11.6 (11.3 to 11.9)	14 (12.9 to 15.1)	7.51 (6.73 to 8.26)	8.39 (7.49 to 9.22)	10.1 (8.88 to 11.3)	0.28 (0.24 to 0.33)	0.28 (0.23 to 0.34)	0.28 (0.25 to 0.31)
Bhutan	10.7 (10 to 11.3)	12 (11.5 to 12.7)	12.4 (11.9 to 13.3)	7.72 (6.87 to 8.58)	8.64 (7.75 to 9.53)	8.89 (7.93 to 9.92)	0.28 (0.24 to 0.32)	0.28 (0.25 to 0.33)	0.28 (0.25 to 0.33)
India	9.16 (8.92 to 9.4)	10.7 (10.4 to 11)	12.3 (11.6 to 13)	6.19 (5.45 to 6.94)	7.26 (6.37 to 8.09)	8.34 (7.23 to 9.43)	0.32 (0.26 to 0.39)	0.32 (0.26 to 0.39)	0.32 (0.28 to 0.38)
Nepal	10.1 (9.43 to 10.7)	11.6 (11.3 to 12)	11.4 (11 to 12.3)	7.11 (6.26 to 7.95)	8.16 (7.31 to 8.95)	8.11 (7.14 to 9.17)	0.3 (0.26 to 0.34)	0.29 (0.26 to 0.36)	0.29 (0.26 to 0.35)
Pakistan	10.6 (10.3 to 11)	10.2 (9.88 to 10.5)	11.2 (10.5 to 12)	7.55 (6.79 to 8.33)	7.16 (6.37 to 7.92)	7.92 (6.91 to 8.94)	0.29 (0.24 to 0.34)	0.3 (0.25 to 0.36)	0.3 (0.26 to 0.34)
**Southeast Asia, East Asia, and Oceania**	10.8 (10.5 to 11.2)	11.7 (11.5 to 12)	13.4 (12.8 to 14)	7.84 (7.02 to 8.62)	8.47 (7.63 to 9.26)	9.62 (8.62 to 10.6)	0.27 (0.23 to 0.33)	0.28 (0.23 to 0.33)	0.28 (0.24 to 0.33)
East Asia	10.6 (10.2 to 11.2)	11.6 (11.3 to 12)	13.5 (12.8 to 14.3)	7.76 (6.96 to 8.56)	8.45 (7.61 to 9.24)	9.76 (8.69 to 10.8)	0.27 (0.23 to 0.32)	0.27 (0.23 to 0.33)	0.28 (0.24 to 0.32)
China	10.6 (10.1 to 11.1)	11.6 (11.2 to 11.9)	13.5 (12.7 to 14.3)	7.72 (6.91 to 8.53)	8.4 (7.57 to 9.2)	9.72 (8.65 to 10.8)	0.27 (0.23 to 0.32)	0.27 (0.23 to 0.33)	0.28 (0.24 to 0.32)
Democratic People's Republic of Korea	11.6 (11 to 12.4)	11.5 (11.3 to 12.2)	12.8 (12.1 to 13.3)	8.53 (7.68 to 9.42)	8.47 (7.7 to 9.3)	9.37 (8.41 to 10.3)	0.27 (0.24 to 0.3)	0.27 (0.24 to 0.32)	0.27 (0.23 to 0.31)
Taiwan (Province of China)	13 (12.9 to 13)	15 (14.9 to 15)	16.1 (14.6 to 17.6)	9.53 (8.64 to 10.4)	11 (9.95 to 11.9)	11.9 (10.3 to 13.5)	0.27 (0.2 to 0.33)	0.27 (0.2 to 0.33)	0.26 (0.24 to 0.29)
Oceania	10.3 (9.83 to 10.8)	10.3 (9.7 to 10.9)	10.7 (9.82 to 11.5)	7.34 (6.51 to 8.14)	7.35 (6.47 to 8.19)	7.6 (6.58 to 8.6)	0.29 (0.24 to 0.34)	0.29 (0.25 to 0.33)	0.29 (0.25 to 0.33)
American Samoa	11.5 (11.3 to 11.8)	12 (11.6 to 12.3)	12.3 (11.6 to 12.9)	8.12 (7.21 to 8.96)	8.33 (7.34 to 9.21)	8.48 (7.37 to 9.5)	0.29 (0.24 to 0.36)	0.3 (0.25 to 0.37)	0.31 (0.26 to 0.37)

Supplementary Table 6: Life expectancy, Health-adjusted life expectancy, and proportion of years spent in ill health in all reporting locations, in 1990, 2005, 2019									
	LE-70			HALE-70			PYIH-70		
location	1990	2005	2019	1990	2005	2019	1990	2005	2019
Cook Islands	10.9 (10.7 to 11.2)	12.7 (12.2 to 13.1)	13.6 (12.7 to 14.4)	7.76 (6.94 to 8.53)	8.81 (7.8 to 9.79)	9.43 (8.17 to 10.6)	0.29 (0.23 to 0.35)	0.3 (0.25 to 0.36)	0.31 (0.27 to 0.36)
Fiji	10.1 (9.39 to 10.9)	10.2 (10.1 to 10.3)	10.5 (9.5 to 11.6)	7.19 (6.29 to 8.12)	7.09 (6.26 to 7.87)	7.29 (6.21 to 8.4)	0.29 (0.25 to 0.33)	0.31 (0.24 to 0.38)	0.31 (0.27 to 0.35)
Guam	12.3 (11.9 to 12.7)	16.1 (15.6 to 16.7)	16 (15 to 17)	8.96 (8.08 to 9.77)	11.6 (10.4 to 12.8)	11.5 (10.1 to 12.8)	0.27 (0.23 to 0.32)	0.28 (0.23 to 0.33)	0.28 (0.24 to 0.33)
Kiribati	8.01 (7.49 to 8.63)	8.37 (7.87 to 8.98)	8.84 (8.11 to 9.69)	5.8 (5.09 to 6.56)	6.03 (5.32 to 6.77)	6.33 (5.48 to 7.21)	0.28 (0.24 to 0.32)	0.28 (0.25 to 0.32)	0.28 (0.26 to 0.32)
Marshall Islands	9.22 (8.9 to 9.57)	9.58 (9.14 to 10.1)	10.1 (9.05 to 11.2)	6.6 (5.88 to 7.32)	6.81 (6.01 to 7.62)	7.14 (6.03 to 8.25)	0.28 (0.24 to 0.34)	0.29 (0.25 to 0.34)	0.29 (0.26 to 0.33)
Micronesia (Federated States of)	9.19 (8.45 to 9.97)	9.56 (9.07 to 10.1)	9.51 (8.69 to 10.5)	6.7 (5.86 to 7.56)	6.88 (6.09 to 7.63)	6.84 (5.91 to 7.83)	0.27 (0.24 to 0.31)	0.28 (0.25 to 0.33)	0.28 (0.25 to 0.32)
Nauru	9.18 (8.49 to 10)	8.32 (7.69 to 9.07)	9.44 (8.79 to 10.1)	6.65 (5.82 to 7.5)	6.04 (5.33 to 6.85)	6.72 (5.88 to 7.51)	0.28 (0.25 to 0.31)	0.27 (0.24 to 0.31)	0.29 (0.26 to 0.33)
Niue	11.1 (10.3 to 11.6)	11.1 (10.6 to 11.5)	11.7 (11.1 to 12.5)	7.89 (6.92 to 8.79)	7.76 (6.89 to 8.6)	8.12 (7.09 to 9.09)	0.29 (0.24 to 0.33)	0.3 (0.25 to 0.35)	0.31 (0.27 to 0.36)
Northern Mariana Islands	11.7 (11 to 12.1)	12.3 (11.7 to 12.9)	12.5 (12.1 to 13.2)	8.46 (7.53 to 9.33)	8.83 (7.88 to 9.75)	8.93 (7.91 to 9.9)	0.28 (0.23 to 0.31)	0.28 (0.24 to 0.33)	0.29 (0.25 to 0.34)
Palau	10.1 (9.16 to 10.9)	10.6 (9.76 to 11.5)	10.8 (9.78 to 11.9)	7.22 (6.28 to 8.18)	7.42 (6.48 to 8.4)	7.53 (6.45 to 8.66)	0.28 (0.25 to 0.31)	0.3 (0.27 to 0.34)	0.3 (0.27 to 0.34)
Papua New Guinea	10.5 (9.91 to 11.1)	10.3 (9.49 to 11.1)	10.7 (9.68 to 11.5)	7.45 (6.53 to 8.27)	7.31 (6.34 to 8.24)	7.56 (6.52 to 8.6)	0.29 (0.26 to 0.34)	0.29 (0.26 to 0.33)	0.29 (0.25 to 0.33)
Samoa	10.6 (9.94 to 11)	11.3 (10.6 to 11.9)	11.7 (10.8 to 12.4)	7.64 (6.69 to 8.49)	8.09 (7.14 to 9.02)	8.31 (7.26 to 9.33)	0.28 (0.23 to 0.33)	0.29 (0.24 to 0.33)	0.29 (0.25 to 0.33)
Solomon Islands	7.78 (7.19 to 8.52)	7.83 (7.33 to 8.43)	8.22 (7.59 to 8.95)	5.79 (5.1 to 6.55)	5.77 (5.1 to 6.45)	5.99 (5.23 to 6.77)	0.26 (0.23 to 0.29)	0.26 (0.24 to 0.3)	0.27 (0.24 to 0.31)
Tokelau	10.9 (10.2 to 11.4)	11.5 (10.6 to 12.3)	12.3 (11.2 to 13.2)	7.92 (7.02 to 8.75)	8.19 (7.16 to 9.2)	8.71 (7.56 to 9.77)	0.28 (0.23 to 0.31)	0.28 (0.25 to 0.32)	0.29 (0.26 to 0.33)
Tonga	12.3 (11.7 to 13)	12.6 (12.1 to 13.2)	12.7 (11.9 to 13.7)	8.91 (7.94 to 9.84)	9.09 (8.12 to 10)	9.14 (8.01 to 10.2)	0.28 (0.24 to 0.32)	0.28 (0.24 to 0.33)	0.28 (0.25 to 0.32)
Tuvalu	9.6 (9 to 10.3)	10.3 (9.6 to 10.9)	10.9 (9.62 to 12)	6.97 (6.19 to 7.77)	7.41 (6.56 to 8.26)	7.75 (6.53 to 8.95)	0.27 (0.24 to 0.31)	0.28 (0.24 to 0.32)	0.29 (0.26 to 0.32)
Vanuatu	9.57 (8.7 to 10.3)	9.72 (8.8 to 10.5)	10.1 (9.02 to 11)	6.98 (6.06 to 7.86)	7.09 (6.19 to 8.02)	7.29 (6.2 to 8.29)	0.27 (0.24 to 0.3)	0.27 (0.24 to 0.3)	0.28 (0.24 to 0.31)
Southeast Asia	11.5 (11.3 to 11.7)	12 (11.8 to 12.2)	12.8 (12.4 to 13.3)	8.14 (7.26 to 8.94)	8.57 (7.68 to 9.42)	9.1 (8.08 to 10)	0.29 (0.24 to 0.36)	0.29 (0.23 to 0.35)	0.29 (0.25 to 0.35)
Cambodia	9.86 (9.52 to 10.3)	10.8 (10.4 to 11.3)	11.4 (11 to 12.2)	7.04 (6.22 to 7.81)	7.73 (6.87 to 8.54)	8.05 (7.11 to 9.07)	0.29 (0.24 to 0.35)	0.28 (0.24 to 0.34)	0.29 (0.26 to 0.35)
Indonesia	11.1 (10.8 to 11.5)	11.2 (10.8 to 11.5)	11.4 (11 to 12.3)	7.69 (6.78 to 8.56)	7.78 (6.88 to 8.67)	7.98 (7.03 to 8.98)	0.31 (0.25 to 0.37)	0.3 (0.25 to 0.36)	0.3 (0.27 to 0.36)
Lao People's Democratic Republic	9.28 (8.69 to 9.98)	10.3 (9.89 to 10.9)	11.3 (10.7 to 12.1)	6.77 (6 to 7.61)	7.53 (6.74 to 8.29)	8.2 (7.29 to 9.17)	0.27 (0.24 to 0.31)	0.27 (0.24 to 0.32)	0.28 (0.24 to 0.32)
Malaysia	12.1 (12 to 12.2)	12.1 (12.1 to 12.2)	12.7 (11.5 to 13.9)	8.57 (7.65 to 9.43)	8.62 (7.7 to 9.47)	9.07 (7.85 to 10.3)	0.29 (0.23 to 0.37)	0.29 (0.22 to 0.36)	0.29 (0.26 to 0.32)
Maldives	10.5 (10.3 to 10.7)	13.2 (12.9 to 13.6)	14.9 (14 to 15.9)	7.63 (6.88 to 8.35)	9.61 (8.67 to 10.5)	10.7 (9.45 to 12)	0.27 (0.22 to 0.33)	0.27 (0.22 to 0.33)	0.28 (0.25 to 0.32)
Mauritius	11.6 (11.5 to 11.8)	12.9 (12.7 to 13)	14.3 (13.1 to 15.5)	8.27 (7.42 to 9.1)	9.01 (8 to 9.94)	9.96 (8.61 to 11.3)	0.29 (0.23 to 0.35)	0.3 (0.24 to 0.37)	0.3 (0.27 to 0.34)
Myanmar	9.59 (8.95 to 10.3)	10.4 (9.92 to 10.9)	11.6 (11.1 to 11.9)	6.86 (6 to 7.7)	7.41 (6.59 to 8.23)	8.19 (7.25 to 9.05)	0.28 (0.26 to 0.33)	0.28 (0.25 to 0.34)	0.29 (0.24 to 0.35)
Philippines	12.4 (12 to 12.7)	12.4 (12 to 12.7)	12.6 (11.7 to 13.6)	8.82 (7.83 to 9.71)	8.85 (7.89 to 9.71)	9.01 (7.99 to 10.1)	0.29 (0.24 to 0.35)	0.28 (0.24 to 0.34)	0.29 (0.26 to 0.32)
Seychelles	12.1 (11.8 to 12.3)	12.2 (11.9 to 12.5)	12.8 (12.3 to 13.3)	8.78 (7.87 to 9.59)	8.71 (7.76 to 9.62)	9.01 (7.98 to 9.99)	0.27 (0.22 to 0.34)	0.29 (0.23 to 0.35)	0.3 (0.25 to 0.35)
Sri Lanka	11.5 (11.3 to 11.8)	12.6 (12.1 to 13.1)	14.1 (12.7 to 15.7)	8.33 (7.46 to 9.15)	8.84 (7.78 to 9.88)	9.8 (8.33 to 11.4)	0.28 (0.23 to 0.34)	0.3 (0.25 to 0.35)	0.31 (0.28 to 0.34)
Thailand	13.2 (12.8 to 13.8)	15.2 (14.5 to 15.9)	16.7 (15 to 18.5)	9.47 (8.47 to 10.5)	11 (9.8 to 12.1)	12.1 (10.4 to 13.8)	0.28 (0.24 to 0.34)	0.28 (0.24 to 0.33)	0.27 (0.25 to 0.3)
Timor-Leste	11.1 (10.5 to 11.5)	11.7 (11.2 to 12.1)	11.4 (11 to 12.2)	7.91 (7.01 to 8.76)	8.29 (7.38 to 9.13)	8.04 (7.05 to 9.1)	0.29 (0.24 to 0.33)	0.29 (0.24 to 0.34)	0.29 (0.26 to 0.36)
Viet Nam	11.5 (11 to 12.2)	12.3 (11.6 to 12.6)	12.8 (12.4 to 13.6)	8.47 (7.61 to 9.36)	9.01 (8.08 to 9.87)	9.24 (8.22 to 10.2)	0.27 (0.23 to 0.31)	0.27 (0.21 to 0.3)	0.28 (0.25 to 0.34)
**Sub-Saharan Africa**	10.1 (9.88 to 10.3)	10.4 (10.1 to 10.6)	11.5 (11.1 to 11.8)	7.33 (6.59 to 8.04)	7.54 (6.79 to 8.24)	8.39 (7.55 to 9.2)	0.28 (0.22 to 0.33)	0.27 (0.22 to 0.33)	0.27 (0.22 to 0.32)
Central Sub-Saharan Africa	9.32 (8.83 to 9.79)	9.83 (9.4 to 10.3)	10.9 (10.1 to 11.6)	6.73 (6 to 7.47)	7.13 (6.4 to 7.86)	7.96 (7.01 to 8.89)	0.28 (0.24 to 0.32)	0.27 (0.24 to 0.32)	0.27 (0.23 to 0.3)
Angola	8.92 (8.39 to 9.55)	9.79 (9.3 to 10.3)	11.1 (10.4 to 11.6)	6.54 (5.81 to 7.29)	7.17 (6.41 to 7.92)	8.17 (7.23 to 9)	0.27 (0.24 to 0.31)	0.27 (0.23 to 0.31)	0.27 (0.22 to 0.3)
Central African Republic	8.29 (7.8 to 8.79)	8.15 (7.42 to 9.05)	8.8 (7.83 to 9.89)	5.97 (5.27 to 6.67)	5.9 (5.11 to 6.78)	6.42 (5.44 to 7.47)	0.28 (0.24 to 0.32)	0.28 (0.25 to 0.31)	0.27 (0.24 to 0.31)
Congo	8.81 (8.23 to 9.54)	9.81 (9.2 to 10.5)	10.6 (9.91 to 11.2)	6.45 (5.74 to 7.23)	7.15 (6.31 to 7.91)	7.8 (6.87 to 8.67)	0.27 (0.24 to 0.3)	0.27 (0.25 to 0.31)	0.27 (0.22 to 0.31)
Democratic Republic of the Congo	9.51 (8.92 to 10.1)	9.91 (9.46 to 10.4)	11 (10 to 11.9)	6.84 (6.05 to 7.64)	7.17 (6.41 to 7.95)	8 (7.01 to 8.99)	0.28 (0.24 to 0.32)	0.28 (0.24 to 0.32)	0.27 (0.24 to 0.3)
Equatorial Guinea	8.69 (7.94 to 9.42)	11.2 (10.1 to 12.7)	11.5 (10.4 to 12.5)	6.16 (5.36 to 6.95)	7.98 (6.86 to 9.23)	8.3 (7.16 to 9.43)	0.29 (0.26 to 0.33)	0.29 (0.27 to 0.32)	0.28 (0.25 to 0.31)
Gabon	9.93 (9.43 to 10.4)	10.4 (9.78 to 11)	11.2 (10.7 to 12.1)	7.27 (6.49 to 8.02)	7.57 (6.72 to 8.38)	8.21 (7.31 to 9.17)	0.27 (0.23 to 0.31)	0.27 (0.24 to 0.31)	0.27 (0.24 to 0.32)

Supplementary Table 6:Life expectancy, Health-adjusted life expectancy, and proportion of years spent in ill health in all reporting locations, in 1990, 2005, 2019									
	LE-70			HALE-70			PYIH-70		
location	1990	2005	2019	1990	2005	2019	1990	2005	2019
Eastern Sub-Saharan Africa	9.37 (9.16 to 9.59)	10.2 (10 to 10.4)	11.4 (11.1 to 11.7)	6.81 (6.11 to 7.45)	7.44 (6.68 to 8.12)	8.34 (7.51 to 9.1)	0.27 (0.22 to 0.33)	0.27 (0.22 to 0.33)	0.27 (0.22 to 0.32)
Burundi	8.51 (7.88 to 9.02)	9.35 (8.82 to 9.97)	10.7 (9.69 to 11.6)	6.3 (5.54 to 6.99)	6.89 (6.15 to 7.63)	7.94 (6.9 to 8.96)	0.26 (0.23 to 0.3)	0.26 (0.23 to 0.3)	0.26 (0.23 to 0.29)
Comoros	9.95 (9.35 to 10.7)	11.1 (10.5 to 11.5)	11.8 (10.9 to 12.2)	7.34 (6.53 to 8.22)	8.24 (7.4 to 9.05)	8.79 (7.76 to 9.65)	0.26 (0.23 to 0.3)	0.26 (0.22 to 0.3)	0.26 (0.21 to 0.29)
Djibouti	10.5 (9.65 to 11.3)	10.8 (9.82 to 12)	11.5 (10.5 to 12.1)	7.81 (6.88 to 8.7)	8.04 (7.05 to 9.15)	8.56 (7.53 to 9.4)	0.26 (0.23 to 0.29)	0.25 (0.23 to 0.28)	0.26 (0.22 to 0.28)
Eritrea	8.54 (8.04 to 9.06)	9.5 (8.66 to 10.5)	10.2 (9.22 to 11.1)	6.26 (5.57 to 6.97)	6.96 (6.12 to 7.88)	7.53 (6.51 to 8.5)	0.27 (0.23 to 0.31)	0.27 (0.25 to 0.29)	0.26 (0.23 to 0.29)
Ethiopia	8.2 (7.95 to 8.45)	10.1 (9.7 to 10.4)	11.8 (11.3 to 12.4)	5.93 (5.28 to 6.53)	7.34 (6.54 to 8.08)	8.7 (7.75 to 9.6)	0.28 (0.23 to 0.34)	0.27 (0.22 to 0.33)	0.26 (0.23 to 0.32)
Kenya	11.1 (10.7 to 11.4)	10.7 (10.5 to 11)	11.4 (11 to 12)	7.92 (7.1 to 8.73)	7.72 (6.94 to 8.46)	8.32 (7.43 to 9.19)	0.28 (0.24 to 0.34)	0.28 (0.23 to 0.34)	0.27 (0.24 to 0.32)
Madagascar	10.3 (9.82 to 10.8)	10.1 (9.61 to 10.7)	10.9 (9.84 to 12)	7.55 (6.77 to 8.32)	7.46 (6.66 to 8.21)	8.09 (7.02 to 9.18)	0.26 (0.23 to 0.31)	0.26 (0.23 to 0.31)	0.26 (0.24 to 0.29)
Malawi	9.48 (9.15 to 9.84)	9.95 (9.45 to 10.4)	11.3 (10.7 to 11.9)	6.9 (6.2 to 7.57)	7.26 (6.4 to 8.01)	8.32 (7.42 to 9.16)	0.27 (0.23 to 0.32)	0.27 (0.23 to 0.32)	0.26 (0.23 to 0.31)
Mozambique	9.54 (9.18 to 9.86)	10 (9.58 to 10.5)	10.4 (9.49 to 11.1)	6.87 (6.11 to 7.58)	7.2 (6.41 to 7.96)	7.54 (6.55 to 8.43)	0.28 (0.23 to 0.33)	0.28 (0.24 to 0.33)	0.27 (0.24 to 0.31)
Rwanda	8.54 (8.1 to 8.93)	10.5 (9.93 to 10.9)	11.7 (11 to 12.1)	6.29 (5.63 to 6.93)	7.66 (6.88 to 8.45)	8.65 (7.74 to 9.46)	0.26 (0.22 to 0.31)	0.27 (0.23 to 0.31)	0.26 (0.22 to 0.3)
Somalia	8.54 (7.85 to 9.26)	9.04 (8.12 to 9.87)	9.71 (8.53 to 10.7)	6.35 (5.62 to 7.09)	6.71 (5.82 to 7.62)	7.22 (6.15 to 8.23)	0.26 (0.23 to 0.28)	0.26 (0.23 to 0.28)	0.26 (0.23 to 0.28)
South Sudan	10 (9.29 to 10.9)	10.6 (9.71 to 12)	11.4 (10.5 to 12.6)	7 (6.08 to 7.94)	7.38 (6.36 to 8.59)	7.94 (6.82 to 9.18)	0.3 (0.27 to 0.35)	0.31 (0.28 to 0.34)	0.3 (0.27 to 0.35)
Uganda	9.81 (9.36 to 10.4)	10.2 (9.85 to 10.6)	11.5 (11.1 to 12.2)	7.11 (6.35 to 7.9)	7.47 (6.71 to 8.18)	8.47 (7.59 to 9.3)	0.28 (0.24 to 0.32)	0.27 (0.23 to 0.32)	0.26 (0.24 to 0.31)
United Republic of Tanzania	10.2 (9.79 to 10.6)	10.9 (10.6 to 11.3)	11.8 (11.3 to 12.4)	7.48 (6.73 to 8.2)	7.97 (7.14 to 8.75)	8.69 (7.81 to 9.48)	0.27 (0.23 to 0.31)	0.27 (0.23 to 0.32)	0.26 (0.23 to 0.31)
Zambia	9.24 (8.91 to 9.7)	9.22 (8.72 to 9.74)	10.7 (9.89 to 11.4)	6.84 (6.1 to 7.58)	6.81 (6.08 to 7.49)	7.99 (7.03 to 8.9)	0.26 (0.22 to 0.32)	0.26 (0.23 to 0.3)	0.26 (0.22 to 0.29)
Southern Sub-Saharan Africa	12.9 (12.7 to 13.1)	10.9 (10.8 to 11.1)	12.4 (12.2 to 12.6)	9.19 (8.24 to 10.1)	7.78 (6.99 to 8.55)	8.82 (7.92 to 9.71)	0.29 (0.23 to 0.35)	0.29 (0.23 to 0.35)	0.29 (0.23 to 0.35)
Botswana	10.2 (9.25 to 10.9)	9.52 (8.38 to 11.1)	10.8 (9.71 to 11.7)	7.38 (6.39 to 8.33)	6.85 (5.77 to 8.08)	7.66 (6.58 to 8.69)	0.28 (0.24 to 0.31)	0.28 (0.27 to 0.31)	0.29 (0.26 to 0.32)
Eswatini	10.2 (9.64 to 10.9)	8.42 (7.58 to 9.35)	10.2 (9.03 to 11.6)	7.36 (6.56 to 8.18)	6.01 (5.16 to 6.88)	7.31 (6.16 to 8.45)	0.28 (0.25 to 0.32)	0.29 (0.26 to 0.32)	0.29 (0.27 to 0.32)
Lesotho	10.5 (9.93 to 10.8)	8.61 (7.98 to 9.26)	9.06 (8.06 to 10.2)	7.6 (6.8 to 8.35)	6.21 (5.44 to 7)	6.49 (5.52 to 7.52)	0.27 (0.23 to 0.31)	0.28 (0.24 to 0.32)	0.28 (0.26 to 0.31)
Namibia	10.1 (9.72 to 10.5)	9.84 (9.25 to 10.4)	11.2 (10.4 to 12)	7.3 (6.5 to 8.08)	7.07 (6.28 to 7.87)	8.08 (7.04 to 9.14)	0.28 (0.23 to 0.33)	0.28 (0.24 to 0.32)	0.28 (0.24 to 0.32)
South Africa	13.7 (13.5 to 14)	11.4 (11.3 to 11.5)	13 (12.8 to 13.2)	9.75 (8.74 to 10.7)	8.12 (7.29 to 8.92)	9.22 (8.28 to 10.1)	0.29 (0.23 to 0.35)	0.29 (0.23 to 0.36)	0.29 (0.23 to 0.35)
Zimbabwe	10.7 (10.4 to 11.1)	9.42 (8.87 to 9.95)	10.2 (9.37 to 11.1)	7.81 (7.02 to 8.56)	6.83 (6.09 to 7.59)	7.39 (6.45 to 8.34)	0.27 (0.23 to 0.32)	0.28 (0.24 to 0.31)	0.27 (0.25 to 0.31)
Western Sub-Saharan Africa	10.2 (9.84 to 10.5)	10.4 (10 to 10.9)	11.5 (11 to 11.9)	7.4 (6.63 to 8.11)	7.65 (6.87 to 8.4)	8.42 (7.54 to 9.27)	0.27 (0.23 to 0.33)	0.27 (0.23 to 0.32)	0.27 (0.22 to 0.31)
Benin	10 (9.61 to 10.4)	10.5 (9.83 to 11)	11.3 (10.4 to 12)	7.29 (6.56 to 8)	7.72 (6.92 to 8.53)	8.32 (7.32 to 9.27)	0.27 (0.23 to 0.32)	0.26 (0.23 to 0.3)	0.26 (0.23 to 0.3)
Burkina Faso	9.77 (9.34 to 10.2)	10.1 (9.69 to 10.5)	10.8 (10.2 to 11.3)	7.11 (6.33 to 7.87)	7.45 (6.7 to 8.18)	8.03 (7.19 to 8.81)	0.27 (0.23 to 0.32)	0.26 (0.22 to 0.31)	0.26 (0.22 to 0.3)
Cabo Verde	14 (13.6 to 14.3)	13.8 (13.2 to 14.4)	12.5 (12.2 to 12.9)	10.4 (9.41 to 11.3)	10.2 (9.15 to 11.3)	9.24 (8.33 to 10.1)	0.25 (0.21 to 0.31)	0.26 (0.22 to 0.31)	0.26 (0.22 to 0.32)
Cameroon	10.3 (9.84 to 10.7)	9.89 (9.45 to 10.5)	11.2 (10.2 to 11.9)	7.44 (6.65 to 8.22)	7.12 (6.34 to 7.95)	8.16 (7.12 to 9.12)	0.28 (0.23 to 0.32)	0.28 (0.24 to 0.33)	0.27 (0.24 to 0.3)
Chad	9.93 (9.55 to 10.3)	9.71 (9.27 to 10.2)	10.6 (9.85 to 11.3)	7.29 (6.54 to 8.03)	7.07 (6.32 to 7.82)	7.79 (6.87 to 8.67)	0.27 (0.22 to 0.32)	0.27 (0.23 to 0.32)	0.27 (0.23 to 0.3)
Côte d'Ivoire	9.84 (9.4 to 10.2)	9.78 (9.23 to 10.2)	11.2 (10.5 to 11.8)	6.97 (6.16 to 7.78)	7.15 (6.37 to 7.91)	8.22 (7.26 to 9.08)	0.29 (0.24 to 0.34)	0.27 (0.23 to 0.31)	0.27 (0.23 to 0.31)
Gambia	10.4 (9.64 to 11.1)	10.6 (10.1 to 11.3)	10.9 (10.2 to 11.8)	7.69 (6.78 to 8.55)	7.78 (6.96 to 8.65)	8.05 (7.14 to 8.92)	0.26 (0.23 to 0.3)	0.26 (0.23 to 0.31)	0.26 (0.24 to 0.3)
Ghana	10.3 (9.63 to 10.9)	10.4 (10 to 10.9)	11.3 (10.7 to 11.6)	7.64 (6.81 to 8.48)	7.73 (6.93 to 8.5)	8.41 (7.48 to 9.22)	0.26 (0.22 to 0.29)	0.26 (0.22 to 0.31)	0.26 (0.21 to 0.3)
Guinea	10 (9.6 to 10.4)	10.1 (9.62 to 10.6)	10.7 (9.85 to 11.5)	7.43 (6.67 to 8.19)	7.49 (6.75 to 8.23)	7.9 (6.93 to 8.9)	0.26 (0.21 to 0.31)	0.26 (0.22 to 0.3)	0.26 (0.23 to 0.3)
Guinea-Bissau	8.51 (7.99 to 9.09)	9.21 (8.71 to 9.67)	10.1 (9.18 to 11)	6.27 (5.57 to 6.98)	6.8 (6.08 to 7.46)	7.47 (6.53 to 8.41)	0.26 (0.23 to 0.3)	0.26 (0.23 to 0.3)	0.26 (0.24 to 0.29)
Liberia	9.92 (9.42 to 10.3)	10.6 (10.1 to 11.2)	11.6 (10.6 to 12.3)	7.13 (6.32 to 7.9)	7.63 (6.74 to 8.48)	8.31 (7.21 to 9.35)	0.28 (0.24 to 0.33)	0.28 (0.24 to 0.33)	0.28 (0.24 to 0.32)
Mali	9.67 (9.33 to 10.1)	10.6 (10.2 to 11)	11.3 (10.4 to 12)	7.08 (6.33 to 7.81)	7.81 (7.03 to 8.56)	8.37 (7.37 to 9.28)	0.27 (0.22 to 0.32)	0.27 (0.22 to 0.31)	0.26 (0.23 to 0.29)
Mauritania	10 (9.61 to 10.5)	11.6 (10.9 to 12.1)	12.4 (11.6 to 12.7)	7.48 (6.72 to 8.21)	8.68 (7.76 to 9.52)	9.25 (8.33 to 10.1)	0.25 (0.22 to 0.3)	0.25 (0.21 to 0.29)	0.25 (0.2 to 0.28)
Niger	9.6 (9.29 to 9.95)	10.3 (9.97 to 10.7)	11.1 (10.2 to 11.8)	7.18 (6.47 to 7.83)	7.75 (7.06 to 8.46)	8.31 (7.36 to 9.2)	0.25 (0.21 to 0.3)	0.25 (0.21 to 0.29)	0.25 (0.22 to 0.28)
Nigeria	10.3 (9.8 to 10.9)	10.6 (9.87 to 11.5)	11.8 (11 to 12.7)	7.48 (6.64 to 8.31)	7.78 (6.84 to 8.73)	8.63 (7.64 to 9.69)	0.28 (0.24 to 0.32)	0.27 (0.24 to 0.31)	0.27 (0.24 to 0.3)

Supplementary Table 6: Life expectancy, Health-adjusted life expectancy, and proportion of years spent in ill health in all reporting locations, in 1990, 2005, 2019									
	LE-70			HALE-70			PYIH-70		
location	1990	2005	2019	1990	2005	2019	1990	2005	2019
Sao Tome and Principe	11.3 (10.8 to 11.7)	11.1 (10.7 to 11.4)	11.2 (11.1 to 11.9)	8.34 (7.5 to 9.12)	8.1 (7.34 to 8.89)	8.17 (7.36 to 9.03)	0.26 (0.22 to 0.31)	0.27 (0.22 to 0.32)	0.27 (0.24 to 0.33)
Senegal	10.2 (9.89 to 10.5)	11 (10.7 to 11.3)	11.7 (10.9 to 12.1)	7.52 (6.76 to 8.23)	8.02 (7.14 to 8.8)	8.6 (7.6 to 9.46)	0.27 (0.22 to 0.32)	0.27 (0.22 to 0.33)	0.27 (0.22 to 0.3)
Sierra Leone	9.67 (9.16 to 10.3)	9.78 (9.35 to 10.2)	11.1 (10.1 to 11.6)	7.16 (6.4 to 7.95)	7.2 (6.47 to 7.92)	8.16 (7.12 to 9.07)	0.26 (0.23 to 0.3)	0.26 (0.22 to 0.31)	0.26 (0.22 to 0.3)
Togo	10.2 (9.74 to 10.7)	10.3 (9.72 to 10.8)	11.4 (10.5 to 11.8)	7.57 (6.79 to 8.31)	7.64 (6.83 to 8.42)	8.42 (7.4 to 9.29)	0.26 (0.22 to 0.3)	0.26 (0.22 to 0.3)	0.26 (0.22 to 0.3)

Supplementary Table 7: Life expectancy, Health-adjusted life expectancy, and proportion of years spent in ill health in all reporting locations, in 1990, 2005, 2019, Males									
	LE-70			HALE-70			PYIH-70		
location	1990	2005	2019	1990	2005	2019	1990	2005	2019
**Global**	10.6 (10.4 to 10.8)	11.5 (11.4 to 11.7)	12.9 (12.5 to 13.3)	7.72 (6.92 to 8.42)	8.4 (7.58 to 9.18)	9.35 (8.43 to 10.3)	0.27 (0.22 to 0.34)	0.27 (0.22 to 0.33)	0.27 (0.23 to 0.33)
**Low SDI**	8.99 (8.74 to 9.26)	9.66 (9.42 to 9.92)	11 (10.6 to 11.4)	6.48 (5.81 to 7.11)	6.95 (6.22 to 7.65)	7.92 (7.06 to 8.76)	0.28 (0.23 to 0.34)	0.28 (0.23 to 0.34)	0.28 (0.23 to 0.33)
**Low-middle SDI**	9.56 (9.34 to 9.81)	10.4 (10.2 to 10.7)	11.6 (11 to 12.1)	6.86 (6.15 to 7.54)	7.46 (6.67 to 8.2)	8.28 (7.36 to 9.21)	0.28 (0.23 to 0.34)	0.28 (0.23 to 0.35)	0.28 (0.24 to 0.33)
**Middle SDI**	10.5 (10 to 10.9)	11 (10.7 to 11.3)	12.1 (11.6 to 12.7)	7.7 (6.9 to 8.45)	8.11 (7.33 to 8.83)	8.89 (8.01 to 9.82)	0.26 (0.22 to 0.31)	0.26 (0.22 to 0.32)	0.27 (0.23 to 0.31)
**High-middle SDI**	10.5 (10.2 to 10.7)	11.1 (11 to 11.3)	13 (12.5 to 13.5)	7.7 (6.93 to 8.38)	8.19 (7.39 to 8.92)	9.57 (8.61 to 10.5)	0.26 (0.22 to 0.32)	0.26 (0.21 to 0.33)	0.26 (0.22 to 0.31)
**High SDI**	11.9 (11.9 to 11.9)	13.9 (13.9 to 13.9)	15.2 (15.1 to 15.3)	8.62 (7.74 to 9.42)	10 (8.98 to 10.9)	10.9 (9.78 to 11.9)	0.28 (0.21 to 0.35)	0.28 (0.21 to 0.35)	0.28 (0.22 to 0.35)
**Central Europe, Eastern Europe, and Central Asia**	9.99 (9.97 to 10)	9.82 (9.8 to 9.84)	11.5 (11.1 to 12.1)	7.26 (6.49 to 7.93)	7.18 (6.45 to 7.83)	8.44 (7.48 to 9.27)	0.27 (0.21 to 0.35)	0.27 (0.2 to 0.34)	0.27 (0.23 to 0.32)
Central Asia	10.6 (10.4 to 10.7)	8.71 (8.61 to 8.8)	9.68 (9.26 to 10.1)	8.02 (7.3 to 8.68)	6.66 (6.09 to 7.17)	7.34 (6.66 to 8.05)	0.24 (0.19 to 0.3)	0.24 (0.19 to 0.29)	0.24 (0.2 to 0.28)
Armenia	10.9 (10.8 to 11)	10.8 (10.7 to 10.9)	11.8 (10.9 to 12.7)	8.13 (7.37 to 8.82)	8.13 (7.38 to 8.8)	8.83 (7.86 to 9.82)	0.25 (0.2 to 0.32)	0.25 (0.19 to 0.31)	0.25 (0.22 to 0.28)
Azerbaijan	10.7 (10.3 to 11.2)	9.28 (8.94 to 9.62)	9.66 (8.79 to 10.5)	8.23 (7.45 to 8.98)	7.15 (6.51 to 7.79)	7.41 (6.49 to 8.32)	0.23 (0.2 to 0.28)	0.23 (0.19 to 0.27)	0.23 (0.21 to 0.26)
Georgia	9.76 (9.35 to 10.2)	9.89 (9.47 to 10.3)	10.5 (9.73 to 11.4)	7.47 (6.74 to 8.17)	7.51 (6.77 to 8.19)	7.8 (6.93 to 8.72)	0.23 (0.2 to 0.28)	0.24 (0.21 to 0.28)	0.26 (0.23 to 0.29)
Kazakhstan	10 (9.83 to 10.2)	8.85 (8.65 to 9.04)	10.2 (9.57 to 10.8)	7.48 (6.77 to 8.13)	6.64 (6.03 to 7.24)	7.58 (6.75 to 8.41)	0.25 (0.2 to 0.31)	0.25 (0.2 to 0.3)	0.26 (0.22 to 0.29)
Kyrgyzstan	10.8 (10.5 to 11)	9.76 (9.69 to 9.83)	11.6 (11 to 12.2)	8.14 (7.37 to 8.85)	7.49 (6.85 to 8.06)	8.91 (8.04 to 9.78)	0.24 (0.2 to 0.3)	0.23 (0.18 to 0.29)	0.23 (0.2 to 0.27)
Mongolia	8.38 (7.69 to 9.17)	7.88 (7.29 to 8.57)	9.31 (8.29 to 10.3)	6.41 (5.63 to 7.17)	6.03 (5.37 to 6.76)	7.14 (6.13 to 8.14)	0.24 (0.22 to 0.27)	0.23 (0.21 to 0.26)	0.23 (0.21 to 0.26)
Tajikistan	11.8 (11.3 to 12.3)	9.42 (9.05 to 9.81)	8.8 (7.93 to 9.67)	8.97 (8.09 to 9.81)	7.3 (6.61 to 7.97)	6.82 (5.93 to 7.7)	0.24 (0.2 to 0.28)	0.22 (0.19 to 0.27)	0.23 (0.2 to 0.25)
Turkmenistan	10.4 (10.3 to 10.5)	9.34 (9.26 to 9.41)	11 (9.91 to 12.2)	7.98 (7.29 to 8.59)	7.22 (6.6 to 7.74)	8.52 (7.4 to 9.64)	0.23 (0.18 to 0.29)	0.23 (0.18 to 0.29)	0.23 (0.21 to 0.25)
Uzbekistan	11.4 (11.3 to 11.4)	6.9 (6.76 to 7.05)	7.88 (7.32 to 8.5)	8.73 (7.96 to 9.4)	5.36 (4.91 to 5.78)	6.09 (5.43 to 6.78)	0.23 (0.18 to 0.3)	0.22 (0.18 to 0.27)	0.23 (0.2 to 0.26)
Central Europe	9.97 (9.93 to 10)	10.9 (10.9 to 11)	12.3 (11.7 to 13.1)	7.2 (6.43 to 7.88)	7.91 (7.09 to 8.65)	8.9 (7.86 to 9.93)	0.28 (0.21 to 0.35)	0.28 (0.21 to 0.35)	0.28 (0.24 to 0.33)
Albania	10.1 (10 to 10.3)	11.9 (11.8 to 12.1)	12.8 (11.3 to 14.3)	7.56 (6.85 to 8.21)	8.93 (8.1 to 9.69)	9.57 (8.22 to 11)	0.25 (0.2 to 0.32)	0.25 (0.2 to 0.31)	0.25 (0.23 to 0.27)
Bosnia and Herzegovina	11.1 (11 to 11.2)	10.8 (10.7 to 10.9)	11.9 (10.7 to 13.1)	8.09 (7.28 to 8.83)	7.82 (7 to 8.54)	8.51 (7.32 to 9.76)	0.27 (0.21 to 0.34)	0.28 (0.22 to 0.34)	0.28 (0.26 to 0.31)
Bulgaria	9.42 (9.19 to 9.67)	9.56 (9.33 to 9.8)	10.6 (9.54 to 11.7)	6.93 (6.2 to 7.58)	7.06 (6.37 to 7.71)	7.8 (6.77 to 8.91)	0.26 (0.22 to 0.33)	0.26 (0.21 to 0.32)	0.26 (0.24 to 0.29)
Croatia	9.79 (9.45 to 10.1)	10.9 (10.8 to 11)	12.7 (11.5 to 13.9)	6.99 (6.22 to 7.75)	7.74 (6.86 to 8.52)	8.95 (7.72 to 10.2)	0.29 (0.23 to 0.34)	0.29 (0.23 to 0.36)	0.29 (0.26 to 0.33)
Czechia	9.17 (9.11 to 9.22)	11.6 (11.5 to 11.7)	13.3 (12.2 to 14.4)	6.56 (5.86 to 7.2)	8.14 (7.18 to 8.98)	9.29 (8.01 to 10.5)	0.28 (0.22 to 0.36)	0.3 (0.23 to 0.38)	0.3 (0.27 to 0.35)
Hungary	9.61 (9.56 to 9.66)	10.7 (10.7 to 10.8)	12.3 (11.2 to 13.3)	6.8 (6.03 to 7.48)	7.57 (6.74 to 8.34)	8.7 (7.54 to 9.85)	0.29 (0.23 to 0.37)	0.29 (0.23 to 0.37)	0.29 (0.26 to 0.33)
Montenegro	11.7 (11.4 to 12)	10.8 (10.6 to 11.1)	11.1 (10.3 to 12)	8.6 (7.73 to 9.43)	7.98 (7.14 to 8.74)	8.14 (7.11 to 9.17)	0.27 (0.22 to 0.32)	0.26 (0.21 to 0.32)	0.27 (0.24 to 0.31)
North Macedonia	10.2 (10 to 10.4)	9.5 (9.31 to 9.69)	10.3 (9.31 to 11.3)	7.5 (6.74 to 8.2)	6.97 (6.23 to 7.63)	7.51 (6.49 to 8.59)	0.27 (0.21 to 0.33)	0.27 (0.21 to 0.33)	0.27 (0.24 to 0.3)
Poland	10 (9.98 to 10)	11.6 (11.6 to 11.6)	13 (11.8 to 14.4)	7.18 (6.42 to 7.86)	8.39 (7.52 to 9.19)	9.43 (8.14 to 10.8)	0.28 (0.22 to 0.36)	0.28 (0.21 to 0.35)	0.28 (0.25 to 0.31)
Romania	10.3 (10.3 to 10.4)	10.9 (10.8 to 10.9)	12 (11 to 13)	7.51 (6.74 to 8.19)	8.01 (7.22 to 8.71)	8.81 (7.7 to 9.91)	0.27 (0.21 to 0.34)	0.26 (0.2 to 0.33)	0.27 (0.24 to 0.3)
Serbia	10.8 (10.7 to 10.8)	10.1 (10 to 10.1)	11.5 (10.5 to 12.5)	7.86 (7.07 to 8.6)	7.38 (6.64 to 8.05)	8.31 (7.22 to 9.46)	0.27 (0.21 to 0.34)	0.27 (0.21 to 0.34)	0.28 (0.25 to 0.31)
Slovakia	9.95 (9.87 to 10)	10.8 (10.7 to 10.9)	12.5 (11.3 to 13.8)	7.19 (6.42 to 7.87)	7.76 (6.91 to 8.52)	8.99 (7.75 to 10.3)	0.28 (0.22 to 0.35)	0.28 (0.22 to 0.35)	0.28 (0.25 to 0.31)
Slovenia	10.8 (9.54 to 12.3)	12.5 (12.3 to 12.7)	14.3 (12.8 to 15.6)	7.63 (6.43 to 8.91)	8.74 (7.72 to 9.68)	10.2 (8.66 to 11.6)	0.3 (0.27 to 0.33)	0.3 (0.24 to 0.37)	0.29 (0.25 to 0.33)
Eastern Europe	9.9 (9.88 to 9.91)	9.29 (9.28 to 9.3)	11.6 (10.8 to 12.3)	7.17 (6.4 to 7.83)	6.78 (6.07 to 7.39)	8.49 (7.41 to 9.42)	0.28 (0.21 to 0.35)	0.27 (0.21 to 0.35)	0.26 (0.24 to 0.32)
Belarus	10.6 (10.6 to 10.7)	9.31 (9.26 to 9.36)	10.8 (9.53 to 12.1)	7.81 (7.01 to 8.52)	6.8 (6.1 to 7.42)	7.95 (6.72 to 9.21)	0.27 (0.2 to 0.34)	0.27 (0.21 to 0.34)	0.26 (0.24 to 0.3)
Estonia	9.76 (9.63 to 9.89)	10.7 (10.6 to 10.9)	13.1 (11.9 to 14.5)	7.15 (6.39 to 7.81)	7.85 (7.04 to 8.57)	9.66 (8.36 to 11.1)	0.27 (0.21 to 0.34)	0.27 (0.21 to 0.33)	0.26 (0.24 to 0.3)
Latvia	10.1 (9.94 to 10.2)	10.1 (10 to 10.2)	12.3 (11.1 to 13.6)	7.27 (6.49 to 7.97)	7.36 (6.56 to 8.06)	9.03 (7.79 to 10.3)	0.28 (0.22 to 0.35)	0.27 (0.21 to 0.34)	0.27 (0.24 to 0.3)
Lithuania	10.9 (10.8 to 11)	10.8 (10.7 to 10.9)	12.5 (11.5 to 13.6)	7.86 (7 to 8.62)	7.78 (6.96 to 8.53)	9.13 (7.95 to 10.3)	0.28 (0.22 to 0.35)	0.28 (0.22 to 0.35)	0.27 (0.24 to 0.31)
Republic of Moldova	10.1 (9.97 to 10.1)	9.88 (9.8 to 9.96)	12.4 (11.7 to 13.1)	7.38 (6.62 to 8.03)	7.33 (6.62 to 7.97)	9.17 (8.16 to 10.2)	0.27 (0.21 to 0.34)	0.26 (0.2 to 0.32)	0.26 (0.22 to 0.3)
Russian Federation	9.7 (9.69 to 9.72)	9.15 (9.14 to 9.16)	11.6 (10.6 to 12.7)	7 (6.24 to 7.66)	6.65 (5.92 to 7.27)	8.52 (7.32 to 9.62)	0.28 (0.21 to 0.36)	0.27 (0.21 to 0.35)	0.27 (0.24 to 0.31)
Ukraine	10.1 (10.1 to 10.1)	9.46 (9.44 to 9.48)	11.3 (10.3 to 12.3)	7.34 (6.58 to 8)	6.97 (6.27 to 7.57)	8.37 (7.33 to 9.46)	0.27 (0.21 to 0.35)	0.26 (0.2 to 0.34)	0.26 (0.23 to 0.29)
**High-income**	12 (12 to 12)	13.9 (13.9 to 13.9)	15.2 (15.2 to 15.3)	8.7 (7.82 to 9.5)	10 (9.01 to 11)	10.9 (9.81 to 12)	0.28 (0.21 to 0.35)	0.28 (0.21 to 0.35)	0.28 (0.22 to 0.35)
Australasia	12 (12 to 12)	14.6 (14.6 to 14.7)	15.9 (15.8 to 16)	8.6 (7.69 to 9.43)	10.4 (9.25 to 11.4)	11.3 (10 to 12.4)	0.28 (0.22 to 0.36)	0.29 (0.22 to 0.37)	0.29 (0.23 to 0.36)
Australia	12.1 (12 to 12.1)	14.7 (14.7 to 14.8)	16 (15.8 to 16.1)	8.64 (7.71 to 9.46)	10.4 (9.27 to 11.4)	11.3 (10.1 to 12.4)	0.28 (0.22 to 0.36)	0.29 (0.23 to 0.37)	0.29 (0.23 to 0.36)

Supplementary Table 7: Life expectancy, Health-adjusted life expectancy, and proportion of years spent in ill health in all reporting locations, in 1990, 2005, 2019, Males

location	LE-70			HALE-70			PYIH-70		
	1990	2005	2019	1990	2005	2019	1990	2005	2019
New Zealand	11.7 (11.6 to 11.8)	14.2 (14.1 to 14.3)	15.5 (15.3 to 15.6)	8.42 (7.52 to 9.23)	10.2 (9.12 to 11.2)	11 (9.85 to 12.1)	0.28 (0.22 to 0.35)	0.28 (0.22 to 0.35)	0.29 (0.22 to 0.36)
High-income Asia Pacific	12.6 (12.6 to 12.6)	14.6 (14.5 to 14.6)	16.3 (16.2 to 16.4)	9.36 (8.49 to 10.1)	10.8 (9.82 to 11.7)	12.2 (11.1 to 13.2)	0.26 (0.2 to 0.32)	0.26 (0.2 to 0.32)	0.25 (0.2 to 0.32)
Brunei Darussalam	7.1 (6.82 to 7.41)	9.2 (8.79 to 9.59)	9.72 (9.16 to 10.3)	5.06 (4.51 to 5.61)	6.52 (5.76 to 7.27)	7.01 (6.17 to 7.81)	0.29 (0.24 to 0.34)	0.29 (0.24 to 0.34)	0.28 (0.24 to 0.33)
Japan	13 (13 to 13)	14.8 (14.8 to 14.8)	16.5 (16.4 to 16.6)	9.66 (8.76 to 10.5)	11 (10 to 11.9)	12.3 (11.2 to 13.3)	0.26 (0.2 to 0.32)	0.26 (0.2 to 0.32)	0.25 (0.2 to 0.31)
Republic of Korea	9.89 (9.87 to 9.92)	12.8 (12.8 to 12.9)	15.1 (14.9 to 15.4)	7.35 (6.66 to 7.97)	9.48 (8.6 to 10.3)	11.2 (10.2 to 12.2)	0.26 (0.2 to 0.32)	0.26 (0.2 to 0.33)	0.26 (0.21 to 0.32)
Singapore	11.3 (11.2 to 11.4)	14.5 (14.4 to 14.5)	16.7 (16.6 to 16.9)	8.42 (7.65 to 9.12)	10.8 (9.81 to 11.7)	12.7 (11.6 to 13.7)	0.25 (0.2 to 0.32)	0.25 (0.2 to 0.32)	0.24 (0.19 to 0.3)
High-income North America	12.4 (12.4 to 12.4)	13.9 (13.9 to 13.9)	14.8 (14.7 to 14.8)	8.5 (7.51 to 9.4)	9.47 (8.39 to 10.5)	9.81 (8.59 to 10.9)	0.31 (0.24 to 0.39)	0.32 (0.25 to 0.39)	0.34 (0.26 to 0.42)
Canada	12.4 (12.3 to 12.4)	14.4 (14.4 to 14.4)	15.8 (15.7 to 15.9)	8.98 (8.06 to 9.81)	10.5 (9.46 to 11.4)	11.3 (10.1 to 12.4)	0.27 (0.21 to 0.35)	0.27 (0.21 to 0.34)	0.28 (0.22 to 0.35)
Greenland	9.78 (9.46 to 10.2)	11.2 (10.9 to 11.5)	12 (11.4 to 13)	6.76 (6 to 7.53)	7.91 (7.03 to 8.74)	8.39 (7.38 to 9.53)	0.31 (0.26 to 0.37)	0.29 (0.24 to 0.36)	0.3 (0.27 to 0.35)
United States of America	12.4 (12.4 to 12.4)	13.8 (13.8 to 13.8)	14.6 (14.6 to 14.7)	8.45 (7.46 to 9.36)	9.35 (8.27 to 10.3)	9.63 (8.42 to 10.8)	0.32 (0.25 to 0.4)	0.32 (0.25 to 0.4)	0.34 (0.27 to 0.42)
Southern Latin America	11.3 (11.3 to 11.3)	12.3 (12.3 to 12.3)	13.1 (13 to 13.3)	8.57 (7.81 to 9.25)	9.22 (8.35 to 9.99)	9.79 (8.84 to 10.6)	0.24 (0.18 to 0.31)	0.25 (0.19 to 0.32)	0.25 (0.2 to 0.32)
Argentina	11.2 (11.2 to 11.2)	12 (11.9 to 12)	12.5 (12.4 to 12.6)	8.51 (7.76 to 9.18)	9.01 (8.18 to 9.76)	9.39 (8.53 to 10.2)	0.24 (0.18 to 0.3)	0.25 (0.19 to 0.31)	0.25 (0.19 to 0.31)
Chile	11.8 (11.7 to 11.8)	13.4 (13.3 to 13.4)	14.8 (14.6 to 14.9)	8.82 (7.99 to 9.55)	9.9 (8.94 to 10.8)	10.9 (9.78 to 11.8)	0.25 (0.19 to 0.32)	0.26 (0.2 to 0.33)	0.27 (0.21 to 0.33)
Uruguay	11.2 (11.2 to 11.3)	11.9 (11.8 to 12)	13 (12.8 to 13.2)	8.5 (7.73 to 9.19)	9 (8.19 to 9.71)	9.67 (8.76 to 10.5)	0.24 (0.19 to 0.31)	0.25 (0.19 to 0.31)	0.26 (0.2 to 0.32)
Western Europe	11.7 (11.7 to 11.7)	13.7 (13.7 to 13.7)	15.1 (15.1 to 15.2)	8.65 (7.83 to 9.4)	10.1 (9.12 to 11)	11.2 (10.1 to 12.1)	0.26 (0.19 to 0.33)	0.26 (0.2 to 0.33)	0.26 (0.2 to 0.33)
Andorra	13.1 (11.7 to 14.3)	14.5 (13.5 to 15.4)	15.2 (14.1 to 16.5)	9.85 (8.49 to 11.1)	10.8 (9.5 to 11.9)	11.3 (10 to 12.7)	0.25 (0.22 to 0.27)	0.26 (0.22 to 0.3)	0.26 (0.23 to 0.29)
Austria	11.5 (11.4 to 11.5)	13.6 (13.5 to 13.6)	15.2 (15.1 to 15.3)	8.41 (7.55 to 9.17)	9.79 (8.77 to 10.7)	11.1 (9.88 to 12.1)	0.27 (0.21 to 0.34)	0.28 (0.21 to 0.35)	0.27 (0.21 to 0.35)
Belgium	11.2 (11.2 to 11.3)	13.2 (13.2 to 13.3)	14.7 (14.6 to 14.9)	8.27 (7.47 to 9)	9.66 (8.67 to 10.5)	10.7 (9.62 to 11.7)	0.26 (0.2 to 0.33)	0.27 (0.21 to 0.34)	0.27 (0.21 to 0.34)
Cyprus	11.5 (11.3 to 11.6)	11.9 (11.7 to 12.1)	14.1 (13.5 to 14.6)	8.58 (7.79 to 9.3)	8.79 (7.92 to 9.57)	10.4 (9.33 to 11.4)	0.25 (0.2 to 0.31)	0.26 (0.21 to 0.32)	0.26 (0.22 to 0.31)
Denmark	11.2 (11.2 to 11.3)	12.8 (12.7 to 12.9)	14.6 (14.4 to 14.7)	8.41 (7.64 to 9.11)	9.62 (8.75 to 10.4)	10.9 (9.91 to 11.8)	0.25 (0.19 to 0.32)	0.25 (0.19 to 0.31)	0.25 (0.2 to 0.31)
Finland	11.1 (11 to 11.2)	13.5 (13.5 to 13.6)	15.1 (14.9 to 15.3)	8.17 (7.36 to 8.89)	9.75 (8.71 to 10.7)	11.1 (9.92 to 12.1)	0.26 (0.2 to 0.33)	0.28 (0.21 to 0.35)	0.27 (0.21 to 0.33)
France	12.3 (12.3 to 12.3)	14.3 (14.2 to 14.3)	16 (15.8 to 16.1)	9.29 (8.43 to 10.1)	10.8 (9.81 to 11.7)	12.1 (11 to 13)	0.25 (0.18 to 0.31)	0.24 (0.18 to 0.31)	0.24 (0.19 to 0.31)
Germany	11.1 (11.1 to 11.1)	13.6 (13.6 to 13.7)	14.6 (14.5 to 14.7)	8.28 (7.51 to 8.97)	10.1 (9.09 to 11)	10.7 (9.7 to 11.7)	0.25 (0.19 to 0.32)	0.26 (0.2 to 0.33)	0.26 (0.2 to 0.33)
Greece	12.5 (12.4 to 12.6)	13.8 (13.7 to 13.8)	14.9 (14.7 to 15)	9.38 (8.53 to 10.1)	10.2 (9.22 to 11)	11.1 (10.1 to 12)	0.25 (0.19 to 0.31)	0.26 (0.2 to 0.33)	0.26 (0.2 to 0.32)
Iceland	12.9 (12.6 to 13.2)	14.5 (14.2 to 14.8)	16.1 (15.6 to 16.6)	9.69 (8.83 to 10.5)	10.7 (9.59 to 11.6)	12 (10.8 to 13.1)	0.25 (0.2 to 0.3)	0.26 (0.21 to 0.32)	0.26 (0.21 to 0.31)
Ireland	10.5 (10.4 to 10.5)	12.9 (12.8 to 13)	14.9 (14.7 to 15.1)	7.89 (7.2 to 8.54)	9.59 (8.69 to 10.4)	11.1 (10 to 12)	0.25 (0.19 to 0.31)	0.26 (0.2 to 0.32)	0.26 (0.21 to 0.32)
Israel	13 (12.9 to 13.1)	14.5 (14.4 to 14.6)	16 (15.8 to 16.1)	9.93 (9.05 to 10.7)	10.8 (9.82 to 11.7)	12.1 (11 to 13)	0.24 (0.18 to 0.3)	0.25 (0.19 to 0.32)	0.24 (0.19 to 0.3)
Italy	12.1 (12.1 to 12.1)	13.8 (13.8 to 13.9)	15.4 (15.3 to 15.5)	8.74 (7.83 to 9.57)	9.93 (8.87 to 10.9)	11.2 (10 to 12.2)	0.28 (0.21 to 0.35)	0.28 (0.21 to 0.36)	0.28 (0.21 to 0.35)
Luxembourg	10.9 (10.7 to 11.1)	13.2 (13 to 13.4)	15.7 (15 to 16.4)	8.12 (7.35 to 8.82)	9.7 (8.74 to 10.6)	11.5 (10.2 to 12.7)	0.25 (0.2 to 0.31)	0.27 (0.22 to 0.33)	0.27 (0.23 to 0.32)
Malta	11.3 (11.1 to 11.5)	13 (12.7 to 13.2)	15.3 (14.8 to 15.8)	8.46 (7.69 to 9.16)	9.65 (8.75 to 10.5)	11.3 (10.2 to 12.4)	0.25 (0.2 to 0.31)	0.26 (0.21 to 0.31)	0.26 (0.21 to 0.31)
Monaco	12.5 (11.8 to 13.6)	13.4 (12.8 to 14.5)	14.3 (13.8 to 15)	9.43 (8.44 to 10.5)	10.1 (8.99 to 11.2)	10.6 (9.6 to 11.7)	0.25 (0.23 to 0.28)	0.25 (0.22 to 0.3)	0.26 (0.22 to 0.3)
Netherlands	11.3 (11.3 to 11.4)	12.9 (12.9 to 13)	14.7 (14.5 to 14.8)	8.48 (7.73 to 9.18)	9.62 (8.77 to 10.4)	10.8 (9.86 to 11.7)	0.25 (0.19 to 0.31)	0.26 (0.2 to 0.32)	0.26 (0.21 to 0.32)
Norway	11.7 (11.6 to 11.8)	13.6 (13.5 to 13.7)	15.5 (15.3 to 15.7)	8.41 (7.5 to 9.22)	9.78 (8.73 to 10.7)	11.1 (9.88 to 12.1)	0.28 (0.22 to 0.35)	0.28 (0.22 to 0.35)	0.29 (0.22 to 0.36)
Portugal	11 (11 to 11.1)	13.1 (13 to 13.2)	14.8 (14.6 to 14.9)	8.15 (7.37 to 8.86)	9.66 (8.73 to 10.5)	11 (9.95 to 11.9)	0.26 (0.2 to 0.33)	0.26 (0.2 to 0.33)	0.25 (0.2 to 0.32)
San Marino	13.4 (12.8 to 14.2)	14.7 (13.4 to 16.2)	15.2 (13.5 to 17.6)	10.1 (9.03 to 11)	11 (9.58 to 12.4)	11.4 (9.67 to 13.4)	0.25 (0.22 to 0.29)	0.25 (0.23 to 0.29)	0.25 (0.24 to 0.28)
Spain	12.2 (12.2 to 12.3)	13.9 (13.8 to 13.9)	15.5 (15.4 to 15.6)	9.01 (8.15 to 9.81)	10.2 (9.28 to 11.1)	11.5 (10.4 to 12.4)	0.26 (0.2 to 0.33)	0.26 (0.2 to 0.33)	0.26 (0.2 to 0.32)
Sweden	12.2 (12.2 to 12.3)	14 (13.9 to 14.1)	15.6 (15.4 to 15.7)	9.17 (8.3 to 9.93)	10.4 (9.42 to 11.3)	11.6 (10.5 to 12.5)	0.25 (0.19 to 0.32)	0.26 (0.2 to 0.32)	0.26 (0.2 to 0.32)
Switzerland	12.4 (12.4 to 12.5)	14.7 (14.6 to 14.7)	16.3 (16.2 to 16.5)	9.09 (8.19 to 9.91)	10.8 (9.72 to 11.7)	12.1 (10.9 to 13.2)	0.27 (0.21 to 0.34)	0.26 (0.2 to 0.33)	0.26 (0.2 to 0.33)
United Kingdom	11.2 (11.1 to 11.2)	13.5 (13.4 to 13.5)	14.7 (14.6 to 14.8)	8.29 (7.52 to 8.99)	9.86 (8.88 to 10.7)	10.7 (9.64 to 11.7)	0.26 (0.2 to 0.33)	0.27 (0.2 to 0.34)	0.27 (0.21 to 0.34)
**Latin America and Caribbean**	12.3 (12.2 to 12.4)	13.6 (13.5 to 13.7)	14.1 (13.6 to 14.7)	8.98 (8.05 to 9.81)	9.98 (8.97 to 10.9)	10.3 (9.24 to 11.4)	0.27 (0.21 to 0.34)	0.27 (0.21 to 0.34)	0.27 (0.23 to 0.32)
Andean Latin America	12.9 (12.4 to 13.3)	14.3 (13.8 to 14.9)	14.9 (13.8 to 16.1)	9.7 (8.71 to 10.6)	10.8 (9.66 to 11.7)	11.1 (9.8 to 12.5)	0.25 (0.2 to 0.3)	0.25 (0.21 to 0.3)	0.25 (0.23 to 0.29)
Bolivia (Plurinational State of)	10.2 (9.73 to 10.7)	11.6 (11 to 12.1)	11.5 (10.7 to 12.6)	7.62 (6.81 to 8.42)	8.64 (7.71 to 9.49)	8.6 (7.48 to 9.8)	0.26 (0.22 to 0.3)	0.26 (0.21 to 0.3)	0.25 (0.22 to 0.3)

Supplementary Table 7: Life expectancy, Health-adjusted life expectancy, and proportion of years spent in ill health in all reporting locations, in 1990, 2005, 2019, Males	LE-70			HALE-70			PYIH-70		
location	1990	2005	2019	1990	2005	2019	1990	2005	2019
Ecuador	13.6 (13.5 to 13.7)	13.7 (13.1 to 14.3)	13.9 (12.6 to 15.2)	10.2 (9.3 to 11.1)	10.2 (9.16 to 11.2)	10.3 (8.91 to 11.7)	0.25 (0.19 to 0.31)	0.26 (0.22 to 0.3)	0.26 (0.23 to 0.29)
Peru	13.3 (12.5 to 14.1)	15.6 (14.6 to 16.6)	16.5 (14.7 to 18.3)	10.1 (8.96 to 11.2)	11.8 (10.5 to 13.1)	12.4 (10.6 to 14.2)	0.24 (0.21 to 0.28)	0.25 (0.21 to 0.28)	0.25 (0.23 to 0.28)
Caribbean	12.5 (12.3 to 12.6)	13.2 (12.9 to 13.4)	13.5 (12.7 to 14.4)	9.38 (8.52 to 10.2)	9.85 (8.92 to 10.7)	9.97 (8.84 to 11.1)	0.25 (0.19 to 0.31)	0.25 (0.2 to 0.31)	0.26 (0.23 to 0.3)
Antigua and Barbuda	11.1 (10.8 to 11.3)	13.1 (12.7 to 13.5)	12.8 (12 to 13.6)	8.41 (7.65 to 9.12)	9.81 (8.81 to 10.7)	9.51 (8.42 to 10.6)	0.24 (0.2 to 0.29)	0.25 (0.21 to 0.31)	0.26 (0.22 to 0.3)
Bahamas	11.4 (11 to 11.8)	12.9 (12.5 to 13.3)	12.9 (11.8 to 14)	8.68 (7.86 to 9.44)	9.71 (8.77 to 10.6)	9.72 (8.47 to 10.9)	0.24 (0.2 to 0.29)	0.24 (0.2 to 0.3)	0.25 (0.22 to 0.28)
Barbados	12.1 (11.9 to 12.4)	12.7 (12.4 to 12.9)	13 (12.2 to 14)	9.29 (8.43 to 10.1)	9.63 (8.71 to 10.4)	9.78 (8.65 to 10.9)	0.23 (0.19 to 0.29)	0.24 (0.19 to 0.3)	0.25 (0.22 to 0.29)
Belize	13.8 (13.4 to 14.3)	13.1 (12.8 to 13.4)	14 (13.3 to 14.8)	10.6 (9.6 to 11.5)	9.95 (8.99 to 10.8)	10.6 (9.47 to 11.7)	0.23 (0.19 to 0.28)	0.24 (0.19 to 0.3)	0.24 (0.21 to 0.29)
Bermuda	10.1 (9.97 to 10.3)	13 (12.5 to 13.5)	14 (13.1 to 14.7)	7.74 (7.07 to 8.36)	9.8 (8.81 to 10.7)	10.4 (9.26 to 11.5)	0.24 (0.19 to 0.29)	0.25 (0.2 to 0.29)	0.25 (0.22 to 0.29)
Cuba	12.9 (12.8 to 13)	13.6 (13.5 to 13.7)	13.9 (12.8 to 15.2)	9.67 (8.75 to 10.5)	10.2 (9.16 to 11)	10.2 (8.92 to 11.5)	0.25 (0.19 to 0.32)	0.25 (0.2 to 0.32)	0.27 (0.24 to 0.3)
Dominica	10.5 (10.2 to 10.9)	11.2 (10.5 to 11.8)	11.3 (10.4 to 12.1)	7.94 (7.13 to 8.65)	8.26 (7.33 to 9.16)	8.29 (7.21 to 9.35)	0.24 (0.21 to 0.3)	0.26 (0.23 to 0.3)	0.27 (0.23 to 0.3)
Dominican Republic	14.6 (13.9 to 15.3)	13.4 (12.6 to 14.4)	12.9 (11.7 to 14.2)	11.1 (10 to 12.2)	10.3 (9.09 to 11.3)	9.82 (8.51 to 11.2)	0.24 (0.2 to 0.28)	0.24 (0.21 to 0.28)	0.24 (0.21 to 0.27)
Grenada	9.68 (9.31 to 10.1)	8.1 (7.87 to 8.34)	11.1 (10.8 to 11.5)	7.32 (6.57 to 8.02)	6 (5.41 to 6.57)	8.12 (7.25 to 8.93)	0.24 (0.2 to 0.29)	0.26 (0.21 to 0.31)	0.27 (0.22 to 0.33)
Guyana	8.58 (7.97 to 9.3)	10.2 (9.5 to 10.9)	10.5 (9.36 to 11.8)	6.36 (5.61 to 7.09)	7.52 (6.6 to 8.42)	7.7 (6.57 to 8.94)	0.26 (0.24 to 0.3)	0.26 (0.23 to 0.3)	0.27 (0.24 to 0.3)
Haiti	9.09 (8.73 to 9.59)	10.5 (9.63 to 11.5)	10.8 (9.56 to 12)	6.83 (6.1 to 7.55)	7.84 (6.9 to 8.87)	8.06 (6.86 to 9.25)	0.25 (0.21 to 0.3)	0.25 (0.23 to 0.28)	0.25 (0.23 to 0.28)
Jamaica	13.6 (13.4 to 13.8)	14.6 (14.3 to 15)	13.4 (12.1 to 14.8)	10.3 (9.43 to 11.2)	11 (9.86 to 12)	9.98 (8.69 to 11.4)	0.24 (0.19 to 0.29)	0.25 (0.2 to 0.31)	0.26 (0.23 to 0.28)
Puerto Rico	12.8 (12.7 to 13)	14.4 (14.2 to 14.6)	16.1 (14.5 to 17.7)	9.6 (8.68 to 10.4)	10.6 (9.5 to 11.5)	11.7 (10.1 to 13.4)	0.25 (0.2 to 0.32)	0.27 (0.21 to 0.33)	0.27 (0.24 to 0.3)
Saint Kitts and Nevis	8.89 (8.55 to 9.28)	10.5 (10.2 to 10.8)	10.8 (10.4 to 11)	6.69 (6.02 to 7.32)	7.74 (6.96 to 8.49)	7.84 (6.99 to 8.6)	0.25 (0.21 to 0.3)	0.26 (0.21 to 0.32)	0.27 (0.22 to 0.33)
Saint Lucia	10.3 (10.1 to 10.6)	12.2 (11.9 to 12.4)	12.4 (11.6 to 13.2)	7.68 (6.91 to 8.41)	8.95 (8.03 to 9.81)	9.11 (8.02 to 10.2)	0.25 (0.2 to 0.31)	0.26 (0.21 to 0.32)	0.27 (0.23 to 0.31)
Saint Vincent and the Grenadines	11 (10.6 to 11.3)	12.7 (12.4 to 13)	12 (11.5 to 12.6)	8.26 (7.47 to 9.05)	9.47 (8.53 to 10.3)	8.92 (7.92 to 9.84)	0.25 (0.2 to 0.3)	0.25 (0.2 to 0.31)	0.26 (0.22 to 0.31)
Suriname	12.2 (12 to 12.5)	12 (11.8 to 12.3)	12.8 (11.9 to 13.9)	9.2 (8.33 to 9.98)	8.95 (8.04 to 9.78)	9.42 (8.26 to 10.6)	0.25 (0.2 to 0.3)	0.26 (0.2 to 0.32)	0.27 (0.24 to 0.3)
Trinidad and Tobago	10.6 (10.4 to 10.8)	12.2 (12 to 12.4)	13.2 (11.6 to 14.9)	7.95 (7.19 to 8.62)	9.02 (8.15 to 9.85)	9.73 (8.24 to 11.3)	0.25 (0.2 to 0.31)	0.26 (0.2 to 0.32)	0.26 (0.24 to 0.29)
United States Virgin Islands	12.2 (11.5 to 13)	12.3 (11.7 to 13)	11.1 (10.9 to 11.6)	9.33 (8.38 to 10.3)	9.27 (8.31 to 10.1)	8.24 (7.4 to 9.06)	0.24 (0.21 to 0.27)	0.25 (0.22 to 0.29)	0.26 (0.22 to 0.32)
Central Latin America	12.9 (12.8 to 13)	14 (13.9 to 14)	14.5 (13.7 to 15.3)	9.33 (8.35 to 10.2)	10.2 (9.21 to 11.2)	10.5 (9.3 to 11.7)	0.28 (0.21 to 0.35)	0.27 (0.2 to 0.34)	0.27 (0.23 to 0.32)
Colombia	13.2 (13.2 to 13.3)	15.1 (15 to 15.1)	16.5 (14.8 to 18.3)	9.67 (8.66 to 10.6)	11 (9.91 to 12)	12.2 (10.5 to 13.9)	0.27 (0.2 to 0.34)	0.27 (0.2 to 0.34)	0.26 (0.24 to 0.29)
Costa Rica	14 (13.8 to 14.2)	15.2 (15 to 15.4)	15.4 (13.9 to 16.9)	10.3 (9.27 to 11.2)	11.2 (10.1 to 12.2)	11.2 (9.73 to 12.9)	0.26 (0.21 to 0.33)	0.26 (0.21 to 0.33)	0.27 (0.24 to 0.3)
El Salvador	14.2 (14 to 14.3)	13.7 (13.6 to 13.8)	14.3 (12.8 to 16)	10.4 (9.32 to 11.3)	10 (8.99 to 11)	10.4 (8.88 to 12)	0.27 (0.21 to 0.34)	0.27 (0.21 to 0.34)	0.27 (0.25 to 0.3)
Guatemala	10.1 (9.38 to 10.9)	11.8 (11.2 to 12.6)	13.1 (12 to 14.2)	7.5 (6.57 to 8.44)	8.68 (7.74 to 9.63)	9.55 (8.31 to 10.8)	0.26 (0.23 to 0.3)	0.27 (0.23 to 0.31)	0.27 (0.24 to 0.3)
Honduras	13.1 (12.2 to 14.1)	13.2 (11.9 to 14.8)	11.3 (11.1 to 11.6)	9.73 (8.57 to 10.9)	9.8 (8.4 to 11.3)	8.4 (7.54 to 9.2)	0.26 (0.23 to 0.3)	0.26 (0.24 to 0.29)	0.26 (0.21 to 0.32)
Mexico	12.9 (12.9 to 12.9)	14 (14 to 14)	14.2 (13.5 to 15)	9.2 (8.2 to 10.1)	10.2 (9.2 to 11.2)	10.2 (9.08 to 11.4)	0.29 (0.22 to 0.36)	0.27 (0.21 to 0.34)	0.28 (0.24 to 0.33)
Nicaragua	12.2 (12 to 12.4)	11.2 (11 to 11.3)	11.5 (10.6 to 12.6)	9.04 (8.12 to 9.86)	8.34 (7.54 to 9.04)	8.52 (7.41 to 9.7)	0.26 (0.21 to 0.33)	0.25 (0.2 to 0.31)	0.26 (0.23 to 0.3)
Panama	14.7 (14.5 to 15)	15.2 (14.9 to 15.4)	16.1 (14.5 to 17.7)	10.8 (9.77 to 11.8)	11.2 (10.1 to 12.2)	11.8 (10.2 to 13.4)	0.26 (0.21 to 0.32)	0.26 (0.21 to 0.32)	0.27 (0.24 to 0.3)
Venezuela (Bolivarian Republic of)	12.9 (12.8 to 13)	12.9 (12.9 to 13)	13.4 (11.8 to 15)	9.52 (8.58 to 10.3)	9.61 (8.67 to 10.4)	9.91 (8.44 to 11.5)	0.26 (0.2 to 0.33)	0.26 (0.2 to 0.33)	0.26 (0.24 to 0.28)
Tropical Latin America	11.6 (11.5 to 11.7)	13.2 (13 to 13.3)	14 (13.7 to 14.2)	8.34 (7.46 to 9.15)	9.56 (8.57 to 10.5)	10.2 (9.16 to 11.1)	0.28 (0.22 to 0.35)	0.27 (0.21 to 0.34)	0.27 (0.22 to 0.33)
Brazil	11.5 (11.4 to 11.6)	13.1 (13 to 13.2)	14 (13.7 to 14.2)	8.28 (7.4 to 9.09)	9.53 (8.54 to 10.4)	10.2 (9.15 to 11.1)	0.28 (0.22 to 0.35)	0.27 (0.21 to 0.34)	0.27 (0.22 to 0.33)
Paraguay	14.6 (13.8 to 15.2)	14.1 (13.4 to 14.8)	13.7 (12.2 to 15.4)	10.9 (9.73 to 12)	10.5 (9.45 to 11.6)	10.2 (8.75 to 11.8)	0.25 (0.21 to 0.3)	0.25 (0.21 to 0.29)	0.26 (0.23 to 0.28)
**North Africa and Middle East**	10.9 (10.6 to 11.1)	11.9 (11.6 to 12.2)	12.6 (12.1 to 13.1)	8.05 (7.26 to 8.81)	8.78 (7.94 to 9.61)	9.19 (8.25 to 10.1)	0.26 (0.21 to 0.31)	0.26 (0.21 to 0.32)	0.27 (0.23 to 0.32)
North Africa and Middle East	10.9 (10.6 to 11.1)	11.9 (11.6 to 12.2)	12.6 (12.1 to 13.1)	8.05 (7.26 to 8.81)	8.78 (7.94 to 9.61)	9.19 (8.25 to 10.1)	0.26 (0.21 to 0.31)	0.26 (0.21 to 0.32)	0.27 (0.23 to 0.32)
Afghanistan	8.95 (8.3 to 9.71)	9.3 (8.67 to 10.1)	9.95 (9.47 to 10.9)	6.5 (5.61 to 7.28)	6.71 (5.84 to 7.57)	6.99 (6.1 to 7.94)	0.27 (0.25 to 0.32)	0.28 (0.25 to 0.33)	0.3 (0.28 to 0.36)
Algeria	10.3 (9.56 to 11.1)	12.2 (11.4 to 13.2)	13.5 (12.6 to 14.5)	7.8 (6.83 to 8.76)	9.13 (8.13 to 10.2)	9.93 (8.75 to 11.1)	0.25 (0.21 to 0.29)	0.25 (0.23 to 0.28)	0.26 (0.23 to 0.3)
Bahrain	8.41 (7.93 to 8.94)	9.15 (8.62 to 9.66)	11.8 (10.8 to 12.8)	6.07 (5.38 to 6.75)	6.48 (5.7 to 7.27)	8.27 (7.12 to 9.44)	0.28 (0.25 to 0.32)	0.29 (0.25 to 0.34)	0.3 (0.26 to 0.34)
Egypt	10.3 (10.2 to 10.3)	10.5 (10.5 to 10.5)	11.4 (10.2 to 12.8)	7.79 (7.1 to 8.41)	7.91 (7.22 to 8.55)	8.49 (7.31 to 9.77)	0.24 (0.18 to 0.31)	0.25 (0.19 to 0.31)	0.26 (0.24 to 0.28)
Iran (Islamic Republic of)	11.3 (11 to 11.6)	12.7 (12.6 to 12.9)	14.1 (13.8 to 14.3)	8.16 (7.3 to 8.95)	9.25 (8.31 to 10.1)	10.2 (9.14 to 11.1)	0.28 (0.23 to 0.34)	0.27 (0.22 to 0.34)	0.28 (0.22 to 0.34)

Supplementary Table 7: Life expectancy, Health-adjusted life expectancy, and proportion of years spent in ill health in all reporting locations, in 1990, 2005, 2019, Males									
	LE-70			HALE-70			PYIH-70		
location	1990	2005	2019	1990	2005	2019	1990	2005	2019
Iraq	11.1 (10.1 to 12.2)	10.8 (9.75 to 12)	11.4 (10.9 to 12.6)	7.81 (6.74 to 8.89)	7.84 (6.76 to 8.97)	8.35 (7.42 to 9.42)	0.3 (0.27 to 0.34)	0.27 (0.25 to 0.31)	0.27 (0.25 to 0.32)
Jordan	12.1 (11.3 to 13)	13.8 (13 to 14.7)	14.5 (13.3 to 15.9)	9.01 (8.05 to 10)	10.2 (8.97 to 11.3)	10.7 (9.3 to 12.1)	0.26 (0.23 to 0.29)	0.26 (0.23 to 0.31)	0.27 (0.24 to 0.3)
Kuwait	14.9 (14.6 to 15.3)	14.6 (14.5 to 14.8)	15.5 (14.3 to 16.7)	11 (9.91 to 12.1)	10.6 (9.52 to 11.6)	11.2 (9.78 to 12.6)	0.26 (0.21 to 0.32)	0.27 (0.22 to 0.34)	0.28 (0.24 to 0.32)
Lebanon	10.8 (10 to 11.5)	11.9 (11.2 to 12.5)	12 (11.5 to 12.7)	7.99 (7.08 to 8.88)	8.7 (7.72 to 9.64)	8.6 (7.61 to 9.57)	0.26 (0.23 to 0.29)	0.27 (0.23 to 0.31)	0.29 (0.24 to 0.34)
Libya	13.2 (12.2 to 14.3)	14.5 (14.2 to 14.8)	13.9 (12.7 to 15.3)	9.71 (8.57 to 11)	10.5 (9.32 to 11.5)	9.98 (8.61 to 11.4)	0.27 (0.24 to 0.3)	0.28 (0.22 to 0.34)	0.28 (0.25 to 0.32)
Morocco	11 (10.5 to 11.6)	12.5 (11.2 to 13.7)	11.3 (10.8 to 12.7)	8.33 (7.46 to 9.2)	9.24 (8.02 to 10.5)	8.32 (7.41 to 9.5)	0.25 (0.21 to 0.29)	0.26 (0.23 to 0.29)	0.26 (0.25 to 0.32)
Oman	8.85 (7.94 to 9.93)	10.2 (9.93 to 10.5)	10.2 (9.59 to 10.7)	6.46 (5.57 to 7.45)	7.35 (6.54 to 8.08)	7.3 (6.42 to 8.13)	0.27 (0.25 to 0.3)	0.28 (0.23 to 0.34)	0.28 (0.24 to 0.33)
Palestine	9.62 (8.66 to 10.7)	10.2 (9.75 to 10.6)	11.2 (10.5 to 11.8)	7.15 (6.2 to 8.18)	7.48 (6.7 to 8.24)	8.08 (7.14 to 9)	0.26 (0.24 to 0.28)	0.26 (0.22 to 0.31)	0.28 (0.24 to 0.32)
Qatar	9.02 (8.22 to 9.92)	10.7 (9.78 to 11.7)	11.5 (10.4 to 12.6)	6.54 (5.66 to 7.44)	7.48 (6.41 to 8.55)	8.11 (6.94 to 9.31)	0.27 (0.25 to 0.31)	0.3 (0.27 to 0.34)	0.29 (0.26 to 0.34)
Saudi Arabia	10.8 (10.1 to 11.8)	11.1 (10.8 to 11.4)	12.8 (12.3 to 13.4)	7.68 (6.73 to 8.71)	7.88 (6.94 to 8.71)	8.94 (7.84 to 9.98)	0.29 (0.26 to 0.33)	0.29 (0.24 to 0.36)	0.3 (0.26 to 0.36)
Sudan	9.81 (9.23 to 10.4)	10.8 (9.83 to 12)	11.5 (10.6 to 12.2)	7.33 (6.55 to 8.11)	7.99 (7.03 to 9.03)	8.43 (7.41 to 9.36)	0.25 (0.22 to 0.29)	0.26 (0.25 to 0.28)	0.26 (0.23 to 0.3)
Syrian Arab Republic	11.6 (10.6 to 12.8)	12.5 (11.6 to 13.5)	11.9 (10.5 to 13.2)	8.75 (7.68 to 9.83)	9.38 (8.26 to 10.4)	8.63 (7.37 to 9.95)	0.25 (0.23 to 0.28)	0.25 (0.23 to 0.29)	0.27 (0.25 to 0.3)
Tunisia	12.1 (11.5 to 12.7)	12.2 (11.1 to 13.6)	13 (11.5 to 14.6)	8.98 (7.96 to 9.87)	8.94 (7.71 to 10.2)	9.43 (8.03 to 10.9)	0.26 (0.22 to 0.31)	0.27 (0.24 to 0.3)	0.28 (0.26 to 0.3)
Turkey	11.7 (11.1 to 12.4)	13.3 (12.7 to 14)	13.8 (12.7 to 15.1)	8.75 (7.8 to 9.65)	9.76 (8.71 to 10.8)	10.1 (8.81 to 11.4)	0.25 (0.22 to 0.3)	0.27 (0.23 to 0.32)	0.27 (0.25 to 0.3)
United Arab Emirates	9.2 (8.54 to 10)	9.02 (8.49 to 9.64)	11.6 (10.9 to 12.4)	6.71 (5.89 to 7.57)	6.51 (5.73 to 7.28)	8.26 (7.2 to 9.32)	0.27 (0.24 to 0.31)	0.28 (0.25 to 0.33)	0.29 (0.25 to 0.34)
Yemen	9.4 (8.56 to 10.3)	10.4 (9.58 to 11.5)	10.8 (9.87 to 11.5)	7.05 (6.16 to 7.94)	7.8 (6.86 to 8.79)	8.03 (6.97 to 8.96)	0.25 (0.23 to 0.28)	0.25 (0.23 to 0.28)	0.26 (0.22 to 0.29)
**South Asia**	9.26 (8.97 to 9.56)	10.3 (10 to 10.7)	11.9 (11.1 to 12.8)	6.48 (5.78 to 7.2)	7.21 (6.37 to 8.02)	8.34 (7.23 to 9.47)	0.3 (0.25 to 0.36)	0.3 (0.25 to 0.36)	0.3 (0.26 to 0.35)
South Asia	9.26 (8.97 to 9.56)	10.3 (10 to 10.7)	11.9 (11.1 to 12.8)	6.48 (5.78 to 7.2)	7.21 (6.37 to 8.02)	8.34 (7.23 to 9.47)	0.3 (0.25 to 0.36)	0.3 (0.25 to 0.36)	0.3 (0.26 to 0.35)
Bangladesh	9.97 (9.52 to 10.4)	11.3 (10.9 to 11.7)	13.7 (12.6 to 14.8)	7.29 (6.55 to 8.03)	8.3 (7.42 to 9.12)	10 (8.82 to 11.2)	0.27 (0.23 to 0.31)	0.27 (0.22 to 0.32)	0.26 (0.24 to 0.3)
Bhutan	10.5 (9.66 to 11.3)	11.7 (11.2 to 12.6)	12.1 (11.4 to 13.1)	7.75 (6.82 to 8.64)	8.62 (7.73 to 9.53)	8.82 (7.82 to 9.92)	0.26 (0.23 to 0.29)	0.27 (0.24 to 0.31)	0.27 (0.24 to 0.31)
India	8.94 (8.58 to 9.31)	10.3 (9.88 to 10.7)	11.8 (10.8 to 12.9)	6.17 (5.45 to 6.92)	7.09 (6.24 to 7.93)	8.19 (7.02 to 9.39)	0.31 (0.26 to 0.36)	0.31 (0.26 to 0.37)	0.31 (0.27 to 0.35)
Nepal	9.66 (8.96 to 10.4)	11.1 (10.8 to 11.7)	10.7 (10.2 to 11.7)	6.91 (6.07 to 7.77)	7.93 (7.12 to 8.74)	7.77 (6.83 to 8.88)	0.28 (0.25 to 0.32)	0.28 (0.25 to 0.34)	0.27 (0.24 to 0.33)
Pakistan	10.4 (9.89 to 10.9)	9.82 (9.24 to 10.4)	10.9 (9.75 to 12.3)	7.49 (6.71 to 8.3)	7.02 (6.23 to 7.84)	7.87 (6.76 to 9.05)	0.28 (0.24 to 0.32)	0.29 (0.25 to 0.33)	0.28 (0.26 to 0.31)
**Southeast Asia, East Asia, and Oceania**	9.88 (9.34 to 10.5)	10.5 (10.1 to 10.9)	11.8 (11.1 to 12.7)	7.37 (6.6 to 8.17)	7.82 (7.07 to 8.54)	8.82 (7.89 to 9.83)	0.25 (0.22 to 0.29)	0.25 (0.21 to 0.3)	0.26 (0.22 to 0.29)
East Asia	9.68 (9.03 to 10.4)	10.3 (9.92 to 10.8)	11.9 (10.9 to 12.9)	7.3 (6.52 to 8.16)	7.78 (7.02 to 8.5)	8.91 (7.91 to 10)	0.25 (0.22 to 0.28)	0.25 (0.21 to 0.29)	0.25 (0.22 to 0.28)
China	9.63 (8.97 to 10.4)	10.3 (9.84 to 10.7)	11.8 (10.9 to 12.8)	7.26 (6.48 to 8.13)	7.72 (6.96 to 8.44)	8.87 (7.87 to 10)	0.25 (0.22 to 0.28)	0.25 (0.21 to 0.29)	0.25 (0.22 to 0.28)
Democratic People's Republic of Korea	10.2 (9.48 to 11.1)	10 (9.72 to 11)	10.9 (10.5 to 11.3)	7.84 (7.01 to 8.7)	7.7 (7.03 to 8.53)	8.33 (7.61 to 9.06)	0.23 (0.22 to 0.26)	0.23 (0.22 to 0.28)	0.23 (0.2 to 0.28)
Taiwan (Province of China)	12.3 (12.2 to 12.4)	14 (13.9 to 14.1)	14.7 (13.1 to 16.2)	9.29 (8.46 to 10)	10.5 (9.56 to 11.4)	11.1 (9.52 to 12.6)	0.24 (0.19 to 0.31)	0.25 (0.19 to 0.31)	0.25 (0.22 to 0.28)
Oceania	9.44 (8.91 to 10)	9.56 (8.98 to 10.2)	10 (9.27 to 10.8)	6.89 (6.1 to 7.7)	6.95 (6.11 to 7.72)	7.29 (6.34 to 8.23)	0.27 (0.23 to 0.32)	0.27 (0.24 to 0.32)	0.27 (0.24 to 0.32)
American Samoa	10.4 (10.2 to 10.7)	10.8 (10.5 to 11.1)	11.5 (11.3 to 11.8)	7.46 (6.64 to 8.2)	7.64 (6.75 to 8.49)	8.12 (7.18 to 9.05)	0.28 (0.23 to 0.35)	0.29 (0.24 to 0.36)	0.29 (0.23 to 0.36)
Cook Islands	10 (9.67 to 10.4)	11.4 (10.9 to 11.9)	12.6 (11.9 to 13.1)	7.28 (6.55 to 8.02)	8.08 (7.18 to 8.93)	8.88 (7.82 to 9.84)	0.27 (0.22 to 0.32)	0.29 (0.25 to 0.34)	0.29 (0.25 to 0.34)
Fiji	9.22 (8.53 to 9.89)	8.98 (8.87 to 9.11)	9.39 (8.48 to 10.4)	6.63 (5.75 to 7.47)	6.32 (5.57 to 6.99)	6.58 (5.6 to 7.59)	0.28 (0.24 to 0.33)	0.3 (0.23 to 0.37)	0.3 (0.27 to 0.34)
Guam	11.3 (10.9 to 11.8)	14.2 (13.6 to 14.8)	14.7 (13.8 to 15.7)	8.49 (7.68 to 9.24)	10.5 (9.43 to 11.5)	10.8 (9.64 to 12)	0.25 (0.22 to 0.29)	0.26 (0.22 to 0.31)	0.26 (0.23 to 0.3)
Kiribati	7.37 (6.85 to 8.02)	7.81 (7.19 to 8.56)	8.32 (7.56 to 9.17)	5.39 (4.7 to 6.11)	5.64 (4.93 to 6.49)	5.99 (5.17 to 6.87)	0.27 (0.24 to 0.31)	0.28 (0.24 to 0.31)	0.28 (0.25 to 0.32)
Marshall Islands	8.31 (7.98 to 8.66)	9.66 (9.2 to 10.2)	10.1 (9.06 to 11.2)	6.06 (5.41 to 6.69)	6.97 (6.17 to 7.76)	7.28 (6.17 to 8.37)	0.27 (0.23 to 0.32)	0.28 (0.24 to 0.33)	0.28 (0.25 to 0.32)
Micronesia (Federated States of)	8.79 (8.07 to 9.46)	9.24 (8.74 to 9.8)	9.19 (8.33 to 10.2)	6.51 (5.72 to 7.33)	6.78 (6.02 to 7.49)	6.75 (5.82 to 7.75)	0.26 (0.22 to 0.29)	0.27 (0.24 to 0.31)	0.27 (0.24 to 0.3)
Nauru	8.72 (7.99 to 9.57)	8 (7.37 to 8.74)	8.95 (8.09 to 9.94)	6.44 (5.6 to 7.28)	5.89 (5.19 to 6.69)	6.51 (5.61 to 7.49)	0.26 (0.24 to 0.3)	0.26 (0.23 to 0.3)	0.27 (0.25 to 0.31)
Niue	10 (9.47 to 10.5)	10 (9.73 to 10.4)	10.5 (10.3 to 10.8)	7.31 (6.48 to 8.13)	7.19 (6.36 to 7.95)	7.43 (6.59 to 8.26)	0.27 (0.23 to 0.32)	0.28 (0.24 to 0.35)	0.29 (0.23 to 0.36)
Northern Mariana Islands	11.5 (10.9 to 11.9)	11.7 (11.4 to 12)	11.4 (11 to 11.8)	8.49 (7.57 to 9.33)	8.54 (7.63 to 9.33)	8.29 (7.41 to 9.13)	0.26 (0.22 to 0.31)	0.27 (0.22 to 0.33)	0.27 (0.23 to 0.33)
Palau	9.45 (8.66 to 9.92)	10.1 (9.3 to 10.7)	10.2 (9.25 to 11.1)	6.89 (6.06 to 7.67)	7.19 (6.3 to 8.07)	7.3 (6.28 to 8.26)	0.27 (0.23 to 0.3)	0.29 (0.25 to 0.32)	0.29 (0.25 to 0.32)
Papua New Guinea	9.61 (8.87 to 10.4)	9.56 (8.73 to 10.5)	10.1 (9.19 to 10.9)	7 (6.13 to 7.9)	6.95 (6.03 to 7.86)	7.31 (6.34 to 8.31)	0.27 (0.24 to 0.31)	0.27 (0.25 to 0.31)	0.27 (0.24 to 0.31)
Samoa	9.61 (9.27 to 9.98)	10.8 (10.5 to 10.9)	11.4 (11 to 11.7)	7.06 (6.32 to 7.79)	7.86 (7.07 to 8.62)	8.33 (7.44 to 9.18)	0.27 (0.22 to 0.32)	0.27 (0.21 to 0.33)	0.27 (0.21 to 0.32)

Supplementary Table 7: Life expectancy, Health-adjusted life expectancy, and proportion of years spent in ill health in all reporting locations, in 1990, 2005, 2019, Males									
	LE-70			HALE-70			PYIH-70		
location	1990	2005	2019	1990	2005	2019	1990	2005	2019
Solomon Islands	7.62 (7 to 8.41)	7.71 (7.15 to 8.36)	8.13 (7.43 to 8.94)	5.76 (5.04 to 6.53)	5.75 (5.07 to 6.44)	6.01 (5.24 to 6.86)	0.24 (0.22 to 0.28)	0.25 (0.23 to 0.29)	0.26 (0.23 to 0.3)
Tokelau	11.5 (11.1 to 11.9)	12.2 (11.7 to 12.6)	13.2 (12.3 to 13.6)	8.53 (7.67 to 9.33)	8.94 (8 to 9.81)	9.55 (8.49 to 10.5)	0.26 (0.21 to 0.31)	0.27 (0.22 to 0.32)	0.27 (0.23 to 0.31)
Tonga	10.8 (10.3 to 11.4)	10.7 (10.2 to 11.1)	11 (10.4 to 11.8)	8.03 (7.16 to 8.83)	7.89 (7.1 to 8.63)	8.12 (7.23 to 9.01)	0.26 (0.23 to 0.3)	0.26 (0.23 to 0.31)	0.26 (0.23 to 0.31)
Tuvalu	8.83 (8.53 to 9.27)	10.2 (9.56 to 10.6)	10.8 (9.55 to 11.6)	6.6 (5.93 to 7.24)	7.49 (6.71 to 8.25)	7.9 (6.71 to 8.97)	0.25 (0.22 to 0.3)	0.26 (0.22 to 0.3)	0.27 (0.23 to 0.3)
Vanuatu	9.21 (8.4 to 9.76)	9.33 (8.5 to 9.83)	9.76 (8.72 to 10.4)	6.81 (5.93 to 7.61)	6.93 (6.1 to 7.68)	7.18 (6.16 to 8.04)	0.26 (0.22 to 0.29)	0.26 (0.22 to 0.28)	0.26 (0.23 to 0.29)
Southeast Asia	10.6 (10.3 to 10.9)	11.1 (10.8 to 11.4)	11.7 (11.2 to 12.3)	7.61 (6.8 to 8.36)	8 (7.18 to 8.78)	8.4 (7.47 to 9.29)	0.28 (0.23 to 0.34)	0.28 (0.23 to 0.34)	0.28 (0.24 to 0.33)
Cambodia	8.91 (8.59 to 9.38)	9.75 (9.41 to 10.2)	10.2 (9.85 to 11.2)	6.47 (5.75 to 7.16)	7.09 (6.34 to 7.83)	7.31 (6.47 to 8.28)	0.27 (0.24 to 0.33)	0.27 (0.24 to 0.33)	0.28 (0.26 to 0.34)
Indonesia	10.6 (10.1 to 11.1)	10.6 (10.1 to 11.3)	10.6 (9.81 to 11.7)	7.46 (6.63 to 8.33)	7.56 (6.63 to 8.42)	7.55 (6.58 to 8.67)	0.29 (0.25 to 0.34)	0.29 (0.25 to 0.34)	0.29 (0.26 to 0.33)
Lao People's Democratic Republic	8.42 (7.87 to 9.13)	9.46 (9.05 to 10.1)	10.5 (9.93 to 11.4)	6.24 (5.55 to 7.01)	7.01 (6.27 to 7.72)	7.71 (6.86 to 8.64)	0.26 (0.23 to 0.3)	0.26 (0.23 to 0.31)	0.27 (0.24 to 0.31)
Malaysia	11.5 (11.4 to 11.5)	11.7 (11.6 to 11.8)	12.2 (11 to 13.5)	8.18 (7.32 to 8.96)	8.35 (7.45 to 9.17)	8.79 (7.6 to 10.1)	0.29 (0.22 to 0.36)	0.29 (0.22 to 0.36)	0.28 (0.25 to 0.31)
Maldives	10.5 (10.3 to 10.7)	13.1 (12.7 to 13.4)	14.5 (13.5 to 15.5)	7.73 (6.99 to 8.45)	9.61 (8.69 to 10.5)	10.5 (9.31 to 11.8)	0.27 (0.21 to 0.32)	0.26 (0.22 to 0.32)	0.27 (0.24 to 0.31)
Mauritius	10.2 (10 to 10.4)	11.3 (11.1 to 11.5)	13 (11.9 to 14.2)	7.33 (6.61 to 8.05)	7.96 (7.1 to 8.77)	9.15 (7.88 to 10.4)	0.28 (0.23 to 0.34)	0.29 (0.24 to 0.36)	0.3 (0.27 to 0.34)
Myanmar	8.5 (7.9 to 9.22)	9.24 (8.83 to 9.81)	10.4 (9.77 to 10.7)	6.15 (5.39 to 6.92)	6.69 (5.98 to 7.44)	7.45 (6.6 to 8.22)	0.28 (0.25 to 0.32)	0.28 (0.24 to 0.32)	0.28 (0.23 to 0.32)
Philippines	11.8 (11.3 to 12.4)	11.4 (10.9 to 12)	11.5 (10.1 to 13.1)	8.54 (7.59 to 9.44)	8.31 (7.41 to 9.15)	8.35 (7 to 9.73)	0.28 (0.24 to 0.33)	0.27 (0.24 to 0.32)	0.28 (0.26 to 0.31)
Seychelles	9.92 (9.66 to 10.2)	10.1 (9.86 to 10.4)	11.5 (10.8 to 12.1)	7.26 (6.52 to 7.93)	7.26 (6.49 to 8.04)	8.1 (7.11 to 9.08)	0.27 (0.22 to 0.33)	0.28 (0.23 to 0.34)	0.29 (0.25 to 0.34)
Sri Lanka	10.5 (10.2 to 10.9)	11.4 (10.9 to 12)	13 (11.5 to 14.6)	7.61 (6.81 to 8.4)	8.02 (7.04 to 8.95)	9.03 (7.62 to 10.6)	0.28 (0.23 to 0.33)	0.3 (0.25 to 0.35)	0.31 (0.28 to 0.34)
Thailand	12.1 (11.5 to 12.7)	14.5 (13.6 to 15.3)	15.5 (13.8 to 17.5)	8.73 (7.77 to 9.7)	10.5 (9.42 to 11.7)	11.3 (9.72 to 13.1)	0.28 (0.24 to 0.32)	0.27 (0.24 to 0.31)	0.27 (0.25 to 0.29)
Timor-Leste	11.1 (10.3 to 11.7)	11.5 (10.9 to 12.1)	10.8 (10.3 to 11.8)	7.98 (7.02 to 8.84)	8.18 (7.27 to 9.11)	7.67 (6.73 to 8.76)	0.28 (0.24 to 0.32)	0.29 (0.25 to 0.33)	0.29 (0.26 to 0.35)
Viet Nam	10 (9.43 to 10.6)	10.5 (9.97 to 10.8)	10.7 (10.4 to 11.4)	7.49 (6.7 to 8.25)	7.8 (7.04 to 8.48)	7.83 (6.98 to 8.65)	0.25 (0.22 to 0.29)	0.26 (0.21 to 0.29)	0.27 (0.24 to 0.33)
**Sub-Saharan Africa**	9.28 (9 to 9.6)	9.51 (9.19 to 9.84)	10.7 (10.3 to 11.1)	6.79 (6.07 to 7.5)	6.97 (6.26 to 7.64)	7.88 (7.05 to 8.66)	0.27 (0.22 to 0.33)	0.27 (0.22 to 0.32)	0.26 (0.22 to 0.32)
Central Sub-Saharan Africa	8.35 (7.91 to 8.83)	8.82 (8.45 to 9.28)	9.91 (9.24 to 10.5)	6.08 (5.41 to 6.76)	6.42 (5.74 to 7.08)	7.28 (6.42 to 8.11)	0.27 (0.23 to 0.32)	0.27 (0.24 to 0.32)	0.27 (0.23 to 0.31)
Angola	7.89 (7.4 to 8.54)	8.81 (8.38 to 9.36)	9.99 (9.31 to 10.4)	5.82 (5.16 to 6.53)	6.49 (5.81 to 7.18)	7.36 (6.53 to 8.11)	0.26 (0.24 to 0.3)	0.26 (0.23 to 0.31)	0.26 (0.22 to 0.3)
Central African Republic	7.05 (6.83 to 7.38)	7.08 (6.77 to 7.55)	7.64 (7.15 to 8.31)	5.11 (4.55 to 5.66)	5.12 (4.56 to 5.7)	5.58 (4.9 to 6.32)	0.28 (0.23 to 0.33)	0.28 (0.25 to 0.33)	0.27 (0.24 to 0.31)
Congo	7.89 (7.61 to 8.24)	9.38 (8.96 to 9.95)	10.2 (9.81 to 10.6)	5.8 (5.24 to 6.38)	6.87 (6.09 to 7.56)	7.57 (6.74 to 8.34)	0.27 (0.23 to 0.31)	0.27 (0.24 to 0.32)	0.26 (0.21 to 0.31)
Democratic Republic of the Congo	8.59 (8.03 to 9.18)	8.86 (8.47 to 9.37)	10 (9.25 to 10.8)	6.23 (5.5 to 6.98)	6.43 (5.73 to 7.13)	7.33 (6.42 to 8.23)	0.27 (0.24 to 0.31)	0.27 (0.24 to 0.32)	0.27 (0.24 to 0.31)
Equatorial Guinea	7.4 (6.96 to 7.99)	10.3 (9.41 to 11.6)	10.7 (9.72 to 11.7)	5.28 (4.61 to 5.92)	7.42 (6.42 to 8.54)	7.76 (6.74 to 8.78)	0.29 (0.26 to 0.34)	0.28 (0.26 to 0.32)	0.27 (0.25 to 0.31)
Gabon	8.45 (8.05 to 8.87)	9.15 (8.85 to 9.59)	9.75 (9.41 to 10.5)	6.2 (5.51 to 6.84)	6.66 (5.94 to 7.34)	7.14 (6.33 to 7.98)	0.27 (0.23 to 0.32)	0.27 (0.23 to 0.33)	0.27 (0.24 to 0.33)
Eastern Sub-Saharan Africa	8.6 (8.37 to 8.84)	9.23 (9.03 to 9.44)	10.5 (10.2 to 10.9)	6.28 (5.65 to 6.87)	6.75 (6.06 to 7.37)	7.75 (6.97 to 8.51)	0.27 (0.22 to 0.32)	0.27 (0.22 to 0.33)	0.26 (0.22 to 0.31)
Burundi	7.6 (7.18 to 8.02)	8.55 (8.08 to 9.12)	9.95 (9.01 to 10.8)	5.65 (5.03 to 6.25)	6.31 (5.64 to 6.96)	7.4 (6.46 to 8.35)	0.26 (0.22 to 0.3)	0.26 (0.24 to 0.3)	0.26 (0.23 to 0.28)
Comoros	9.3 (8.71 to 10.1)	10.7 (10 to 11.2)	11.3 (10.4 to 11.8)	6.87 (6.07 to 7.79)	7.94 (7.09 to 8.76)	8.43 (7.41 to 9.26)	0.26 (0.23 to 0.3)	0.25 (0.22 to 0.29)	0.26 (0.22 to 0.29)
Djibouti	9.67 (8.79 to 10.6)	9.86 (8.99 to 11)	10.8 (9.85 to 11.3)	7.25 (6.33 to 8.17)	7.4 (6.5 to 8.45)	8.03 (7.06 to 8.83)	0.25 (0.23 to 0.28)	0.25 (0.23 to 0.28)	0.25 (0.22 to 0.28)
Eritrea	7.31 (7.07 to 7.64)	8.17 (7.78 to 8.71)	9.26 (8.74 to 9.79)	5.39 (4.86 to 5.92)	5.99 (5.38 to 6.63)	6.78 (6.04 to 7.51)	0.26 (0.23 to 0.31)	0.27 (0.24 to 0.31)	0.27 (0.23 to 0.31)
Ethiopia	7.62 (7.29 to 7.99)	9.57 (9.08 to 10)	11.2 (10.4 to 12.2)	5.56 (4.94 to 6.16)	7.05 (6.27 to 7.78)	8.33 (7.33 to 9.43)	0.27 (0.23 to 0.32)	0.26 (0.23 to 0.31)	0.26 (0.23 to 0.29)
Kenya	10.4 (9.76 to 11.1)	9.31 (8.95 to 9.68)	10.4 (9.71 to 11.3)	7.49 (6.62 to 8.39)	6.69 (5.93 to 7.37)	7.53 (6.69 to 8.55)	0.28 (0.24 to 0.32)	0.28 (0.24 to 0.34)	0.27 (0.24 to 0.31)
Madagascar	9.67 (9.22 to 10.2)	9.61 (9.09 to 10.1)	10.5 (9.59 to 11.6)	7.16 (6.4 to 7.89)	7.1 (6.33 to 7.82)	7.87 (6.86 to 8.92)	0.26 (0.22 to 0.31)	0.26 (0.23 to 0.3)	0.25 (0.23 to 0.28)
Malawi	8.72 (8.35 to 9.13)	8.38 (8.05 to 8.67)	9.82 (9.29 to 10.3)	6.38 (5.71 to 7.02)	6.09 (5.38 to 6.72)	7.22 (6.45 to 7.95)	0.27 (0.23 to 0.32)	0.27 (0.22 to 0.33)	0.26 (0.23 to 0.31)
Mozambique	8.62 (8.23 to 8.99)	8.32 (8.03 to 8.59)	8.82 (8.33 to 9.25)	6.23 (5.55 to 6.89)	5.97 (5.35 to 6.57)	6.35 (5.61 to 7.07)	0.28 (0.23 to 0.33)	0.28 (0.24 to 0.33)	0.28 (0.24 to 0.33)
Rwanda	7.74 (7.43 to 8.08)	9.31 (8.83 to 9.71)	10.7 (10 to 11.2)	5.71 (5.15 to 6.27)	6.84 (6.14 to 7.55)	7.92 (7.07 to 8.65)	0.26 (0.22 to 0.31)	0.26 (0.22 to 0.3)	0.26 (0.23 to 0.3)
Somalia	7.64 (7.13 to 8.28)	7.92 (7.38 to 8.6)	8.73 (7.95 to 9.57)	5.74 (5.1 to 6.39)	5.92 (5.25 to 6.69)	6.51 (5.64 to 7.41)	0.25 (0.23 to 0.28)	0.25 (0.22 to 0.29)	0.25 (0.23 to 0.29)
South Sudan	9.33 (8.52 to 10.3)	9.91 (8.96 to 11.2)	10.7 (9.74 to 12)	6.63 (5.73 to 7.58)	7.01 (6.04 to 8.17)	7.61 (6.5 to 8.81)	0.29 (0.27 to 0.33)	0.29 (0.27 to 0.33)	0.29 (0.27 to 0.33)
Uganda	8.55 (8.17 to 9.05)	8.75 (8.33 to 9.19)	10.1 (9.68 to 10.8)	6.22 (5.57 to 6.87)	6.38 (5.7 to 7.02)	7.43 (6.66 to 8.22)	0.27 (0.24 to 0.32)	0.27 (0.24 to 0.32)	0.26 (0.24 to 0.31)
United Republic of Tanzania	9.39 (8.94 to 9.84)	9.82 (9.47 to 10.2)	11.1 (10.6 to 11.8)	6.93 (6.22 to 7.6)	7.24 (6.46 to 7.93)	8.22 (7.38 to 9.04)	0.26 (0.23 to 0.3)	0.26 (0.23 to 0.32)	0.26 (0.24 to 0.3)

location	LE-70			HALE-70			PYIH-70		
	1990	2005	2019	1990	2005	2019	1990	2005	2019
Zambia	8.68 (8.38 to 9.14)	8.13 (7.8 to 8.53)	9.67 (9.08 to 10.3)	6.45 (5.76 to 7.14)	6 (5.36 to 6.6)	7.2 (6.39 to 8.01)	0.26 (0.22 to 0.31)	0.26 (0.23 to 0.31)	0.26 (0.22 to 0.3)
Southern Sub-Saharan Africa	11.5 (11.2 to 11.7)	9.62 (9.47 to 9.74)	11.2 (11 to 11.4)	8.27 (7.45 to 9.06)	6.91 (6.22 to 7.54)	8.02 (7.19 to 8.79)	0.28 (0.23 to 0.34)	0.28 (0.23 to 0.34)	0.28 (0.23 to 0.34)
Botswana	8.72 (8.29 to 9.28)	7.72 (7.27 to 8)	9.44 (8.88 to 10.1)	6.29 (5.54 to 7.06)	5.5 (4.84 to 6.09)	6.67 (5.83 to 7.5)	0.28 (0.24 to 0.33)	0.29 (0.24 to 0.34)	0.29 (0.26 to 0.34)
Eswatini	8.48 (8.09 to 9.12)	7.1 (6.6 to 7.6)	8.28 (7.68 to 8.6)	6.12 (5.47 to 6.81)	5.01 (4.33 to 5.61)	5.87 (5.1 to 6.53)	0.28 (0.25 to 0.32)	0.29 (0.26 to 0.34)	0.29 (0.24 to 0.34)
Lesotho	8.49 (8.02 to 8.8)	7.09 (6.8 to 7.34)	7.7 (7.12 to 8.13)	6.21 (5.56 to 6.83)	5.13 (4.58 to 5.66)	5.53 (4.84 to 6.16)	0.27 (0.22 to 0.31)	0.28 (0.23 to 0.33)	0.28 (0.24 to 0.32)
Namibia	8.8 (8.5 to 9.18)	8.44 (8.17 to 8.65)	9.54 (9.1 to 9.95)	6.37 (5.68 to 7.04)	6.06 (5.43 to 6.69)	6.86 (6.08 to 7.63)	0.28 (0.23 to 0.33)	0.28 (0.23 to 0.33)	0.28 (0.23 to 0.33)
South Africa	12.4 (12.1 to 12.8)	10.3 (10.1 to 10.4)	11.8 (11.6 to 12.1)	8.93 (8.02 to 9.79)	7.36 (6.62 to 8.05)	8.46 (7.56 to 9.26)	0.28 (0.23 to 0.34)	0.28 (0.23 to 0.34)	0.29 (0.24 to 0.35)
Zimbabwe	9.44 (9.08 to 9.84)	8.01 (7.65 to 8.32)	9.03 (8.65 to 9.61)	6.95 (6.24 to 7.61)	5.84 (5.21 to 6.46)	6.62 (5.89 to 7.32)	0.26 (0.23 to 0.31)	0.27 (0.22 to 0.32)	0.27 (0.24 to 0.32)
Western Sub-Saharan Africa	9.62 (9.06 to 10.2)	9.85 (9.21 to 10.6)	10.9 (10.1 to 11.6)	7.09 (6.25 to 7.9)	7.29 (6.42 to 8.19)	8.08 (7.15 to 9)	0.26 (0.23 to 0.31)	0.26 (0.23 to 0.3)	0.26 (0.22 to 0.3)
Benin	9.27 (8.83 to 9.74)	9.44 (8.79 to 10)	10.4 (9.49 to 11.3)	6.83 (6.11 to 7.52)	7.04 (6.29 to 7.77)	7.74 (6.75 to 8.71)	0.26 (0.23 to 0.31)	0.25 (0.23 to 0.28)	0.26 (0.23 to 0.29)
Burkina Faso	9.04 (8.58 to 9.52)	9.26 (8.82 to 9.69)	9.82 (9.2 to 10.4)	6.64 (5.91 to 7.37)	6.88 (6.17 to 7.56)	7.34 (6.54 to 8.1)	0.27 (0.23 to 0.31)	0.26 (0.22 to 0.3)	0.25 (0.22 to 0.29)
Cabo Verde	13.1 (12.8 to 13.4)	11.9 (11.2 to 12.7)	10.6 (10.5 to 10.9)	9.85 (8.93 to 10.7)	8.85 (7.89 to 9.85)	7.94 (7.18 to 8.63)	0.25 (0.2 to 0.3)	0.25 (0.22 to 0.29)	0.25 (0.21 to 0.31)
Cameroon	9.67 (9.17 to 10.1)	8.94 (8.58 to 9.5)	10.3 (9.44 to 11)	7.05 (6.28 to 7.81)	6.47 (5.79 to 7.22)	7.53 (6.58 to 8.42)	0.27 (0.23 to 0.32)	0.28 (0.24 to 0.32)	0.27 (0.24 to 0.3)
Chad	9.43 (8.91 to 9.95)	9.07 (8.58 to 9.62)	10.2 (9.36 to 10.9)	6.99 (6.24 to 7.77)	6.66 (5.93 to 7.36)	7.5 (6.6 to 8.38)	0.26 (0.22 to 0.3)	0.27 (0.23 to 0.31)	0.26 (0.23 to 0.29)
Côte d'Ivoire	8.88 (8.44 to 9.27)	8.68 (8.24 to 9.05)	10.3 (9.64 to 10.9)	6.38 (5.65 to 7.11)	6.37 (5.7 to 7.02)	7.57 (6.68 to 8.38)	0.28 (0.23 to 0.33)	0.27 (0.22 to 0.31)	0.26 (0.23 to 0.31)
Gambia	9.49 (8.74 to 10.3)	9.7 (9.22 to 10.4)	10.2 (9.66 to 11)	7.18 (6.32 to 8.02)	7.29 (6.54 to 8.07)	7.66 (6.84 to 8.46)	0.24 (0.22 to 0.28)	0.25 (0.22 to 0.29)	0.25 (0.23 to 0.29)
Ghana	9.59 (8.92 to 10.3)	9.35 (8.98 to 9.8)	10.1 (9.59 to 10.5)	7.22 (6.43 to 8.08)	7 (6.29 to 7.67)	7.56 (6.76 to 8.28)	0.25 (0.21 to 0.28)	0.25 (0.22 to 0.3)	0.25 (0.21 to 0.3)
Guinea	10 (9.48 to 10.5)	9.55 (9.02 to 10.1)	10.2 (9.39 to 11)	7.51 (6.72 to 8.29)	7.17 (6.45 to 7.89)	7.59 (6.64 to 8.56)	0.25 (0.21 to 0.29)	0.25 (0.22 to 0.29)	0.25 (0.22 to 0.29)
Guinea-Bissau	7.54 (7.09 to 8.09)	8.11 (7.7 to 8.52)	9.19 (8.51 to 9.95)	5.61 (5 to 6.24)	6.07 (5.45 to 6.63)	6.87 (6.06 to 7.7)	0.26 (0.23 to 0.3)	0.25 (0.22 to 0.29)	0.25 (0.23 to 0.29)
Liberia	9.75 (9.13 to 10.3)	10.4 (9.8 to 11.1)	11.4 (10.4 to 12.1)	7.12 (6.31 to 7.89)	7.6 (6.71 to 8.46)	8.27 (7.17 to 9.31)	0.27 (0.23 to 0.31)	0.27 (0.23 to 0.31)	0.28 (0.23 to 0.31)
Mali	9.57 (9.12 to 10.1)	10.7 (10.1 to 11.2)	11.3 (10.3 to 12.2)	7.07 (6.32 to 7.83)	7.9 (7.11 to 8.7)	8.44 (7.39 to 9.43)	0.26 (0.22 to 0.31)	0.26 (0.22 to 0.3)	0.25 (0.22 to 0.28)
Mauritania	9.77 (9.37 to 10.3)	11.8 (11 to 12.3)	12.5 (11.8 to 13)	7.38 (6.65 to 8.11)	8.88 (7.92 to 9.79)	9.42 (8.47 to 10.3)	0.24 (0.21 to 0.29)	0.25 (0.21 to 0.28)	0.24 (0.21 to 0.28)
Niger	9.19 (8.82 to 9.61)	10 (9.59 to 10.5)	10.7 (9.76 to 11.6)	6.95 (6.27 to 7.6)	7.6 (6.88 to 8.29)	8.12 (7.15 to 9.06)	0.24 (0.21 to 0.29)	0.24 (0.21 to 0.28)	0.24 (0.22 to 0.27)
Nigeria	9.83 (8.73 to 11.1)	10.2 (8.88 to 11.9)	11.4 (9.94 to 13.1)	7.22 (6.12 to 8.39)	7.56 (6.36 to 8.87)	8.46 (7.15 to 9.85)	0.27 (0.25 to 0.3)	0.26 (0.26 to 0.28)	0.26 (0.25 to 0.28)
Sao Tome and Principe	11 (10.3 to 11.6)	10.6 (10.2 to 11)	10.6 (10.4 to 11.3)	8.17 (7.28 to 9.01)	7.84 (7.07 to 8.61)	7.77 (6.97 to 8.57)	0.25 (0.22 to 0.29)	0.26 (0.22 to 0.31)	0.27 (0.24 to 0.33)
Senegal	9.55 (9.06 to 10.1)	10.3 (9.87 to 10.8)	11.1 (10.3 to 11.6)	7.07 (6.36 to 7.82)	7.57 (6.73 to 8.38)	8.22 (7.27 to 9.07)	0.26 (0.22 to 0.3)	0.27 (0.22 to 0.32)	0.26 (0.22 to 0.29)
Sierra Leone	8.97 (8.4 to 9.64)	9.22 (8.74 to 9.71)	10.7 (9.73 to 11.3)	6.69 (5.97 to 7.46)	6.84 (6.11 to 7.54)	7.97 (6.95 to 8.89)	0.25 (0.23 to 0.29)	0.26 (0.22 to 0.3)	0.25 (0.22 to 0.29)
Togo	9.56 (8.99 to 10.1)	8.99 (8.52 to 9.39)	10 (9.35 to 10.6)	7.15 (6.41 to 7.89)	6.73 (6.05 to 7.4)	7.51 (6.64 to 8.27)	0.25 (0.22 to 0.29)	0.25 (0.21 to 0.29)	0.25 (0.22 to 0.29)

Supplementary Table 8: Life expectancy, Health-adjusted life expectancy, and proportion of years spent in ill health in all reporting locations, in 1990, 2005, 2019, Females

location	LE-70			HALE-70			PYIH-70		
	1990	2005	2019	1990	2005	2019	1990	2005	2019
**Global**	12.8 (12.7 to 13)	13.9 (13.8 to 14)	15.2 (14.9 to 15.6)	9.1 (8.12 to 10)	9.85 (8.8 to 10.8)	10.7 (9.52 to 11.8)	0.29 (0.23 to 0.36)	0.29 (0.23 to 0.36)	0.3 (0.24 to 0.36)
**Low SDI**	10.1 (9.91 to 10.3)	10.9 (10.7 to 11.1)	12 (11.7 to 12.3)	7.13 (6.32 to 7.85)	7.72 (6.87 to 8.49)	8.47 (7.57 to 9.35)	0.3 (0.24 to 0.36)	0.29 (0.24 to 0.36)	0.29 (0.24 to 0.35)
**Low-middle SDI**	10.5 (10.3 to 10.8)	11.7 (11.5 to 11.9)	12.9 (12.4 to 13.5)	7.33 (6.52 to 8.1)	8.15 (7.23 to 9.01)	8.94 (7.91 to 9.94)	0.31 (0.25 to 0.37)	0.3 (0.25 to 0.37)	0.31 (0.26 to 0.36)
**Middle SDI**	11.8 (11.5 to 12.2)	12.8 (12.6 to 13)	14.3 (13.8 to 14.9)	8.37 (7.44 to 9.26)	9.06 (8.06 to 9.94)	10.1 (8.96 to 11.2)	0.29 (0.24 to 0.35)	0.29 (0.24 to 0.36)	0.3 (0.25 to 0.35)
**High-middle SDI**	12.7 (12.6 to 13)	13.7 (13.6 to 13.9)	15.6 (15.1 to 16.1)	9.16 (8.2 to 10.1)	9.87 (8.83 to 10.8)	11.2 (9.94 to 12.3)	0.28 (0.22 to 0.35)	0.28 (0.22 to 0.35)	0.29 (0.24 to 0.34)
**High SDI**	15.1 (15.1 to 15.1)	16.8 (16.8 to 16.8)	18 (17.9 to 18.1)	10.7 (9.51 to 11.8)	11.9 (10.6 to 13)	12.6 (11.2 to 13.9)	0.29 (0.22 to 0.37)	0.29 (0.22 to 0.37)	0.3 (0.23 to 0.38)
**Central Europe, Eastern Europe, and Central Asia**	12.5 (12.5 to 12.5)	12.4 (12.4 to 12.4)	14.2 (13.7 to 14.7)	9.06 (8.13 to 9.9)	9.08 (8.16 to 9.9)	10.4 (9.27 to 11.4)	0.28 (0.21 to 0.35)	0.27 (0.2 to 0.34)	0.27 (0.22 to 0.32)
Central Asia	13 (12.9 to 13.1)	10.7 (10.6 to 10.8)	12 (11.6 to 12.4)	9.71 (8.82 to 10.5)	8.04 (7.31 to 8.7)	8.93 (8.1 to 9.78)	0.25 (0.2 to 0.31)	0.25 (0.19 to 0.31)	0.25 (0.21 to 0.3)
Armenia	12.7 (12.5 to 12.8)	12.7 (12.5 to 12.8)	13.8 (12.9 to 14.6)	9.4 (8.53 to 10.2)	9.39 (8.5 to 10.2)	10.2 (9.1 to 11.2)	0.26 (0.2 to 0.32)	0.26 (0.2 to 0.32)	0.26 (0.23 to 0.3)
Azerbaijan	13.5 (13.1 to 13.9)	10.4 (10.1 to 10.7)	11 (10.3 to 11.7)	10.2 (9.21 to 11.1)	7.89 (7.15 to 8.58)	8.26 (7.34 to 9.16)	0.24 (0.2 to 0.29)	0.24 (0.2 to 0.29)	0.25 (0.22 to 0.28)
Georgia	11.8 (11.5 to 12.3)	12.5 (12.1 to 12.9)	13.7 (12.8 to 14.7)	9.01 (8.17 to 9.77)	9.39 (8.49 to 10.3)	10.2 (9.05 to 11.3)	0.24 (0.2 to 0.29)	0.25 (0.2 to 0.3)	0.26 (0.23 to 0.3)
Kazakhstan	12.7 (12.5 to 12.9)	11.4 (11.2 to 11.6)	13 (12.4 to 13.6)	9.37 (8.43 to 10.2)	8.42 (7.57 to 9.15)	9.5 (8.5 to 10.5)	0.26 (0.21 to 0.33)	0.26 (0.21 to 0.33)	0.27 (0.23 to 0.31)
Kyrgyzstan	13.8 (13.5 to 14)	11.8 (11.7 to 11.9)	13.8 (13.2 to 14.4)	10.2 (9.25 to 11.1)	8.91 (8.12 to 9.64)	10.4 (9.42 to 11.4)	0.26 (0.2 to 0.32)	0.24 (0.19 to 0.3)	0.24 (0.21 to 0.28)
Mongolia	11.1 (10.4 to 11.9)	10.4 (9.8 to 11.1)	12.1 (11 to 13.2)	8.4 (7.48 to 9.31)	7.93 (7.07 to 8.77)	9.22 (8.03 to 10.4)	0.24 (0.22 to 0.28)	0.24 (0.21 to 0.28)	0.24 (0.22 to 0.27)
Tajikistan	14.3 (13.8 to 14.8)	10.5 (10.1 to 10.8)	10.4 (9.52 to 11.3)	10.8 (9.81 to 11.7)	7.99 (7.26 to 8.71)	7.92 (6.96 to 8.89)	0.25 (0.21 to 0.29)	0.24 (0.2 to 0.28)	0.24 (0.21 to 0.27)
Turkmenistan	12.4 (12.3 to 12.6)	11.8 (11.7 to 11.9)	13.5 (12.2 to 14.7)	9.4 (8.53 to 10.1)	8.94 (8.15 to 9.69)	10.2 (8.86 to 11.5)	0.25 (0.19 to 0.31)	0.24 (0.19 to 0.3)	0.24 (0.22 to 0.27)
Uzbekistan	13.6 (13.5 to 13.6)	8.42 (8.28 to 8.56)	9.38 (8.85 to 9.94)	10.3 (9.34 to 11.1)	6.46 (5.9 to 6.97)	7.16 (6.4 to 7.87)	0.24 (0.19 to 0.31)	0.23 (0.19 to 0.29)	0.24 (0.21 to 0.28)
Central Europe	12.2 (12.2 to 12.2)	13.4 (13.4 to 13.5)	15 (14.3 to 15.7)	8.85 (7.95 to 9.68)	9.83 (8.85 to 10.7)	11 (9.76 to 12.1)	0.27 (0.21 to 0.35)	0.27 (0.2 to 0.34)	0.27 (0.23 to 0.32)
Albania	14 (13.8 to 14.2)	14.4 (14.2 to 14.6)	15.6 (14.2 to 17.2)	10.6 (9.64 to 11.5)	10.9 (9.91 to 11.8)	11.8 (10.4 to 13.3)	0.24 (0.19 to 0.3)	0.24 (0.19 to 0.3)	0.24 (0.22 to 0.27)
Bosnia and Herzegovina	12.5 (12.4 to 12.7)	12.8 (12.6 to 12.9)	13.6 (12.5 to 14.8)	9.23 (8.32 to 10.1)	9.33 (8.4 to 10.2)	9.87 (8.64 to 11.2)	0.26 (0.21 to 0.33)	0.27 (0.21 to 0.33)	0.27 (0.25 to 0.31)
Bulgaria	11.1 (10.9 to 11.3)	11.7 (11.5 to 11.9)	12.9 (11.9 to 14)	8.33 (7.54 to 9.05)	8.76 (7.93 to 9.53)	9.66 (8.5 to 10.8)	0.25 (0.2 to 0.31)	0.25 (0.2 to 0.31)	0.25 (0.23 to 0.29)
Croatia	12.2 (11.9 to 12.5)	13.6 (13.5 to 13.8)	15.3 (14.2 to 16.4)	8.77 (7.83 to 9.61)	9.8 (8.78 to 10.7)	10.8 (9.45 to 12.2)	0.28 (0.23 to 0.34)	0.28 (0.22 to 0.35)	0.29 (0.26 to 0.33)
Czechia	12 (11.9 to 12.1)	14.1 (14 to 14.3)	16 (15 to 17.1)	8.51 (7.53 to 9.38)	10.1 (8.95 to 11.1)	11.3 (9.92 to 12.7)	0.29 (0.23 to 0.37)	0.29 (0.22 to 0.36)	0.29 (0.25 to 0.34)
Hungary	12.1 (12 to 12.2)	13.6 (13.5 to 13.7)	15 (14 to 16)	8.53 (7.58 to 9.4)	9.74 (8.68 to 10.7)	10.8 (9.51 to 12.1)	0.29 (0.23 to 0.37)	0.29 (0.22 to 0.36)	0.28 (0.24 to 0.32)
Montenegro	14.3 (14 to 14.6)	13.2 (12.9 to 13.4)	13.6 (12.9 to 14.2)	10.6 (9.57 to 11.6)	9.79 (8.83 to 10.7)	10 (8.9 to 11)	0.26 (0.2 to 0.31)	0.26 (0.2 to 0.32)	0.26 (0.22 to 0.31)
North Macedonia	11 (10.8 to 11.2)	10.7 (10.6 to 10.9)	11.5 (10.6 to 12.5)	8.06 (7.26 to 8.78)	7.86 (7.04 to 8.6)	8.41 (7.34 to 9.46)	0.27 (0.22 to 0.33)	0.27 (0.21 to 0.33)	0.27 (0.24 to 0.31)
Poland	12.8 (12.8 to 12.9)	14.7 (14.7 to 14.8)	16.3 (15.1 to 17.5)	9.24 (8.25 to 10.1)	10.7 (9.6 to 11.7)	11.9 (10.4 to 13.3)	0.28 (0.21 to 0.35)	0.27 (0.21 to 0.35)	0.27 (0.24 to 0.31)
Romania	11.8 (11.7 to 11.8)	12.7 (12.7 to 12.8)	14.5 (13.6 to 15.5)	8.71 (7.9 to 9.46)	9.55 (8.69 to 10.3)	10.8 (9.6 to 12)	0.26 (0.2 to 0.32)	0.25 (0.19 to 0.32)	0.25 (0.22 to 0.29)
Serbia	11 (10.9 to 11.1)	11.1 (11 to 11.2)	12.7 (11.8 to 13.6)	8.1 (7.33 to 8.8)	8.22 (7.43 to 8.94)	9.3 (8.19 to 10.4)	0.26 (0.2 to 0.33)	0.26 (0.2 to 0.33)	0.27 (0.24 to 0.31)
Slovakia	12.6 (12.4 to 12.7)	13.8 (13.6 to 13.9)	15.2 (14 to 16.4)	9.22 (8.31 to 10)	10.2 (9.17 to 11.1)	11.1 (9.8 to 12.5)	0.27 (0.21 to 0.33)	0.26 (0.2 to 0.33)	0.26 (0.24 to 0.3)
Slovenia	13.3 (12 to 14.7)	15.8 (15.6 to 16.1)	17.8 (16.3 to 19.2)	9.54 (8.22 to 11)	11.2 (9.93 to 12.4)	12.7 (11 to 14.4)	0.28 (0.25 to 0.32)	0.29 (0.23 to 0.36)	0.29 (0.25 to 0.33)
Eastern Europe	12.6 (12.5 to 12.6)	12.1 (12.1 to 12.1)	14.3 (13.6 to 15)	9.07 (8.14 to 9.93)	8.84 (7.93 to 9.64)	10.4 (9.26 to 11.5)	0.28 (0.21 to 0.35)	0.27 (0.21 to 0.35)	0.27 (0.23 to 0.32)
Belarus	13.2 (13.1 to 13.3)	12.8 (12.7 to 12.9)	14.3 (13 to 15.5)	9.67 (8.71 to 10.5)	9.45 (8.54 to 10.3)	10.6 (9.18 to 12)	0.27 (0.21 to 0.33)	0.26 (0.2 to 0.33)	0.26 (0.23 to 0.29)
Estonia	12.5 (12.3 to 12.7)	14.4 (14.1 to 14.6)	16.1 (15 to 17.4)	9.2 (8.29 to 10)	10.6 (9.59 to 11.6)	12 (10.5 to 13.4)	0.26 (0.21 to 0.32)	0.26 (0.21 to 0.32)	0.26 (0.23 to 0.3)
Latvia	12.7 (12.5 to 12.9)	13.7 (13.5 to 13.9)	15.2 (13.8 to 16.5)	9.22 (8.27 to 10.1)	10 (8.99 to 10.9)	11.2 (9.72 to 12.6)	0.27 (0.21 to 0.34)	0.27 (0.21 to 0.33)	0.26 (0.23 to 0.3)
Lithuania	13.5 (13.4 to 13.7)	14.1 (14 to 14.3)	15.7 (14.8 to 16.7)	9.87 (8.86 to 10.8)	10.2 (9.22 to 11.2)	11.6 (10.2 to 12.8)	0.27 (0.21 to 0.34)	0.27 (0.21 to 0.34)	0.26 (0.23 to 0.31)
Republic of Moldova	11.7 (11.6 to 11.8)	12 (11.9 to 12.2)	14.6 (13.9 to 15.3)	8.62 (7.79 to 9.39)	8.93 (8.07 to 9.71)	10.8 (9.66 to 12)	0.26 (0.2 to 0.33)	0.26 (0.2 to 0.32)	0.26 (0.22 to 0.31)
Russian Federation	12.6 (12.5 to 12.6)	12.1 (12 to 12.1)	14.3 (13.4 to 15.3)	9.03 (8.08 to 9.89)	8.74 (7.82 to 9.55)	10.4 (9.11 to 11.6)	0.28 (0.21 to 0.35)	0.28 (0.21 to 0.35)	0.28 (0.24 to 0.32)
Ukraine	12.5 (12.4 to 12.5)	11.9 (11.9 to 11.9)	14.1 (13.1 to 15.1)	9.02 (8.1 to 9.86)	8.79 (7.93 to 9.57)	10.4 (9.24 to 11.6)	0.28 (0.21 to 0.35)	0.26 (0.2 to 0.33)	0.26 (0.23 to 0.3)

Supplementary Table 8: Life expectancy, Health-adjusted life expectancy, and proportion of years spent in ill health in all reporting locations, in 1990, 2005, 2019, Females									
	LE-70			HALE-70			PYIH-70		
location	1990	2005	2019	1990	2005	2019	1990	2005	2019
**High-income**	15.1 (15.1 to 15.2)	16.9 (16.9 to 16.9)	18 (17.9 to 18)	10.7 (9.56 to 11.8)	11.9 (10.6 to 13.1)	12.6 (11.2 to 13.9)	0.29 (0.22 to 0.37)	0.29 (0.22 to 0.37)	0.3 (0.23 to 0.38)
Australasia	15.2 (15.2 to 15.3)	17.3 (17.2 to 17.3)	18.2 (18.1 to 18.3)	10.8 (9.59 to 11.9)	12.1 (10.8 to 13.4)	12.7 (11.2 to 14)	0.29 (0.22 to 0.37)	0.3 (0.23 to 0.37)	0.3 (0.23 to 0.38)
Australia	15.3 (15.2 to 15.4)	17.4 (17.4 to 17.5)	18.3 (18.2 to 18.4)	10.8 (9.67 to 11.9)	12.2 (10.8 to 13.5)	12.7 (11.3 to 14.1)	0.29 (0.22 to 0.37)	0.3 (0.23 to 0.38)	0.3 (0.24 to 0.38)
New Zealand	14.8 (14.7 to 14.9)	16.5 (16.4 to 16.6)	17.6 (17.4 to 17.7)	10.4 (9.25 to 11.5)	11.7 (10.4 to 12.8)	12.3 (10.9 to 13.6)	0.3 (0.23 to 0.37)	0.29 (0.22 to 0.36)	0.3 (0.23 to 0.37)
High-income Asia Pacific	15.8 (15.8 to 15.8)	18.7 (18.7 to 18.7)	20.1 (20 to 20.1)	11.5 (10.3 to 12.5)	13.6 (12.3 to 14.9)	14.6 (13.1 to 15.9)	0.27 (0.21 to 0.35)	0.27 (0.21 to 0.34)	0.27 (0.21 to 0.34)
Brunei Darussalam	11 (10.6 to 11.4)	11.3 (11 to 11.7)	12.8 (12.3 to 13.2)	7.71 (6.85 to 8.56)	8.03 (7.13 to 8.92)	9.1 (8.09 to 10.1)	0.3 (0.25 to 0.35)	0.29 (0.23 to 0.35)	0.29 (0.24 to 0.34)
Japan	16.3 (16.3 to 16.4)	19.2 (19.2 to 19.2)	20.4 (20.3 to 20.5)	11.9 (10.7 to 13)	14 (12.6 to 15.3)	14.9 (13.3 to 16.2)	0.27 (0.21 to 0.35)	0.27 (0.21 to 0.34)	0.27 (0.21 to 0.34)
Republic of Korea	12.8 (12.8 to 12.9)	15.8 (15.7 to 15.8)	18.3 (18.1 to 18.5)	9.25 (8.3 to 10.1)	11.4 (10.3 to 12.5)	13.3 (12 to 14.6)	0.28 (0.21 to 0.35)	0.27 (0.21 to 0.35)	0.27 (0.21 to 0.34)
Singapore	14.2 (14.1 to 14.3)	17.4 (17.2 to 17.5)	19.4 (19.3 to 19.6)	10.3 (9.3 to 11.3)	12.8 (11.6 to 13.9)	14.5 (13.1 to 15.8)	0.27 (0.21 to 0.34)	0.26 (0.2 to 0.33)	0.25 (0.2 to 0.32)
High-income North America	15.7 (15.7 to 15.7)	16.2 (16.2 to 16.2)	17.2 (17.1 to 17.2)	10.6 (9.34 to 11.8)	10.9 (9.58 to 12.1)	11.2 (9.69 to 12.5)	0.32 (0.25 to 0.4)	0.33 (0.25 to 0.41)	0.35 (0.27 to 0.43)
Canada	15.9 (15.8 to 15.9)	17.1 (17.1 to 17.2)	18.2 (18.2 to 18.3)	11.4 (10.2 to 12.5)	12.3 (11 to 13.4)	12.8 (11.4 to 14.1)	0.28 (0.22 to 0.36)	0.28 (0.22 to 0.36)	0.3 (0.23 to 0.37)
Greenland	10.7 (10.1 to 11.4)	12.3 (11.6 to 12.9)	14.2 (13.1 to 15.1)	7.36 (6.49 to 8.29)	8.48 (7.48 to 9.42)	9.69 (8.44 to 11)	0.31 (0.27 to 0.36)	0.31 (0.27 to 0.36)	0.32 (0.27 to 0.36)
United States of America	15.7 (15.7 to 15.7)	16.1 (16.1 to 16.1)	17 (17 to 17.1)	10.6 (9.26 to 11.8)	10.8 (9.42 to 12)	11 (9.5 to 12.4)	0.33 (0.25 to 0.41)	0.33 (0.26 to 0.41)	0.35 (0.28 to 0.44)
Southern Latin America	14 (14 to 14)	15.3 (15.3 to 15.3)	15.9 (15.7 to 16)	10.4 (9.35 to 11.3)	11.3 (10.2 to 12.3)	11.6 (10.4 to 12.7)	0.26 (0.2 to 0.33)	0.26 (0.2 to 0.33)	0.27 (0.21 to 0.34)
Argentina	14.1 (14 to 14.1)	15.2 (15.2 to 15.2)	15.4 (15.3 to 15.5)	10.4 (9.42 to 11.3)	11.2 (10.2 to 12.2)	11.4 (10.3 to 12.4)	0.26 (0.2 to 0.33)	0.26 (0.2 to 0.33)	0.26 (0.2 to 0.33)
Chile	13.7 (13.6 to 13.7)	15.7 (15.6 to 15.7)	16.9 (16.7 to 17)	9.98 (8.98 to 10.9)	11.4 (10.2 to 12.4)	12.1 (10.8 to 13.3)	0.27 (0.21 to 0.34)	0.28 (0.21 to 0.35)	0.28 (0.22 to 0.35)
Uruguay	14.4 (14.3 to 14.5)	15.3 (15.2 to 15.4)	16.2 (16.1 to 16.4)	10.7 (9.66 to 11.6)	11.4 (10.3 to 12.4)	11.9 (10.7 to 13)	0.26 (0.2 to 0.33)	0.26 (0.2 to 0.33)	0.27 (0.21 to 0.33)
Western Europe	14.7 (14.7 to 14.8)	16.7 (16.6 to 16.7)	17.7 (17.7 to 17.8)	10.6 (9.52 to 11.6)	12 (10.7 to 13.1)	12.7 (11.3 to 13.9)	0.28 (0.21 to 0.35)	0.28 (0.21 to 0.36)	0.29 (0.22 to 0.36)
Andorra	16.8 (14.9 to 18.2)	18.7 (16.9 to 19.8)	18.3 (16.7 to 20.1)	12.2 (10.5 to 13.9)	13.4 (11.6 to 15)	13.1 (11.3 to 14.9)	0.27 (0.24 to 0.3)	0.28 (0.24 to 0.31)	0.29 (0.26 to 0.33)
Austria	14.3 (14.2 to 14.3)	16.3 (16.2 to 16.4)	17.8 (17.7 to 17.9)	10.4 (9.33 to 11.4)	11.7 (10.5 to 12.8)	12.8 (11.4 to 14)	0.27 (0.21 to 0.34)	0.28 (0.22 to 0.36)	0.28 (0.22 to 0.35)
Belgium	14.7 (14.7 to 14.8)	16.4 (16.3 to 16.5)	17.5 (17.4 to 17.7)	10.7 (9.57 to 11.7)	11.6 (10.4 to 12.8)	12.4 (11 to 13.7)	0.28 (0.21 to 0.35)	0.29 (0.22 to 0.36)	0.29 (0.23 to 0.37)
Cyprus	11.3 (11.2 to 11.4)	14 (13.8 to 14.1)	15.6 (15.2 to 16)	8.26 (7.41 to 9.02)	10 (8.95 to 11)	11.3 (10.1 to 12.4)	0.27 (0.21 to 0.34)	0.28 (0.22 to 0.35)	0.28 (0.23 to 0.34)
Denmark	14.5 (14.4 to 14.6)	15.4 (15.3 to 15.5)	17 (16.8 to 17.1)	10.4 (9.29 to 11.4)	11.1 (9.95 to 12.1)	12.2 (10.9 to 13.3)	0.28 (0.22 to 0.36)	0.28 (0.22 to 0.35)	0.28 (0.22 to 0.35)
Finland	14.3 (14.2 to 14.4)	16.7 (16.6 to 16.8)	17.9 (17.7 to 18.1)	10.3 (9.21 to 11.3)	11.9 (10.5 to 13.1)	12.8 (11.4 to 14.1)	0.28 (0.21 to 0.35)	0.29 (0.22 to 0.36)	0.28 (0.22 to 0.36)
France	16 (16 to 16.1)	17.9 (17.9 to 17.9)	19.2 (19.1 to 19.3)	11.7 (10.4 to 12.7)	13 (11.7 to 14.2)	13.9 (12.5 to 15.2)	0.27 (0.21 to 0.35)	0.27 (0.21 to 0.35)	0.27 (0.21 to 0.35)
Germany	14.1 (14.1 to 14.1)	16.2 (16.2 to 16.2)	17 (16.9 to 17.1)	10.2 (9.19 to 11.2)	11.7 (10.5 to 12.8)	12.1 (10.8 to 13.3)	0.27 (0.21 to 0.35)	0.28 (0.21 to 0.35)	0.29 (0.22 to 0.36)
Greece	14 (13.9 to 14.1)	15.2 (15.2 to 15.3)	16.7 (16.5 to 16.8)	10.2 (9.21 to 11.1)	11.1 (9.97 to 12.1)	12.1 (11 to 13.3)	0.27 (0.21 to 0.34)	0.27 (0.21 to 0.34)	0.27 (0.21 to 0.34)
Iceland	15.5 (15.2 to 15.8)	17.3 (16.7 to 17.9)	19.7 (19.2 to 20.2)	11.2 (10 to 12.2)	12.3 (11 to 13.6)	14.1 (12.6 to 15.5)	0.28 (0.23 to 0.34)	0.29 (0.24 to 0.35)	0.29 (0.23 to 0.34)
Ireland	13.4 (13.3 to 13.5)	15.7 (15.6 to 15.8)	17.2 (17 to 17.4)	9.74 (8.78 to 10.6)	11.3 (10.2 to 12.3)	12.3 (11.1 to 13.5)	0.27 (0.21 to 0.34)	0.28 (0.22 to 0.35)	0.28 (0.22 to 0.35)
Israel	14.2 (14.1 to 14.3)	15.8 (15.8 to 15.9)	17.6 (17.5 to 17.8)	10.4 (9.37 to 11.3)	11.5 (10.3 to 12.5)	12.8 (11.5 to 14)	0.27 (0.21 to 0.34)	0.28 (0.21 to 0.35)	0.27 (0.21 to 0.34)
Italy	15.1 (15.1 to 15.2)	17.1 (17.1 to 17.2)	18.2 (18.1 to 18.3)	10.6 (9.44 to 11.7)	12.1 (10.8 to 13.4)	12.9 (11.5 to 14.2)	0.3 (0.23 to 0.37)	0.29 (0.22 to 0.37)	0.29 (0.22 to 0.36)
Luxembourg	14.3 (14.1 to 14.5)	16.2 (15.9 to 16.5)	18.2 (17.5 to 18.8)	10.4 (9.3 to 11.3)	11.5 (10.3 to 12.6)	12.8 (11.4 to 14.2)	0.27 (0.22 to 0.34)	0.29 (0.23 to 0.35)	0.3 (0.24 to 0.35)
Malta	14 (13.8 to 14.3)	16.4 (16.1 to 16.7)	18.2 (17.6 to 18.8)	10.2 (9.16 to 11.2)	11.8 (10.5 to 12.9)	13 (11.6 to 14.4)	0.27 (0.22 to 0.34)	0.28 (0.22 to 0.34)	0.28 (0.23 to 0.34)
Monaco	15.8 (14.8 to 17.4)	16.1 (15.2 to 17.8)	16.6 (15.7 to 18.2)	11.6 (10.3 to 13.2)	11.8 (10.3 to 13.3)	12 (10.6 to 13.6)	0.26 (0.24 to 0.3)	0.27 (0.25 to 0.32)	0.28 (0.25 to 0.33)
Netherlands	15.3 (15.2 to 15.3)	16.1 (16 to 16.2)	17.1 (17 to 17.3)	11.1 (10.1 to 12.2)	11.8 (10.6 to 12.8)	12.2 (10.9 to 13.4)	0.27 (0.21 to 0.34)	0.27 (0.21 to 0.34)	0.29 (0.23 to 0.36)
Norway	15.1 (15 to 15.2)	16.7 (16.6 to 16.8)	17.8 (17.7 to 17.9)	10.8 (9.57 to 11.8)	11.9 (10.6 to 13)	12.4 (11 to 13.7)	0.29 (0.22 to 0.36)	0.29 (0.22 to 0.36)	0.3 (0.24 to 0.38)
Portugal	13.5 (13.4 to 13.5)	15.9 (15.8 to 16)	17.6 (17.5 to 17.7)	9.66 (8.68 to 10.6)	11.3 (10.1 to 12.4)	12.6 (11.2 to 13.8)	0.28 (0.22 to 0.35)	0.29 (0.22 to 0.36)	0.28 (0.22 to 0.36)
San Marino	16.4 (15.7 to 17.3)	17.3 (15.6 to 18.9)	18 (15.7 to 20.2)	12.1 (10.8 to 13.3)	12.7 (10.9 to 14.4)	13.1 (11 to 15.2)	0.27 (0.23 to 0.31)	0.27 (0.24 to 0.3)	0.27 (0.25 to 0.3)
Spain	15.1 (15.1 to 15.2)	17.2 (17.2 to 17.3)	18.6 (18.5 to 18.7)	10.9 (9.78 to 11.9)	12.4 (11.1 to 13.6)	13.3 (11.9 to 14.6)	0.28 (0.21 to 0.35)	0.28 (0.21 to 0.35)	0.28 (0.22 to 0.36)

Supplementary Table 8: Life expectancy, Health-adjusted life expectancy, and proportion of years spent in ill health in all reporting locations, in 1990, 2005, 2019, Females									
	LE-70			HALE-70			PYIH-70		
location	1990	2005	2019	1990	2005	2019	1990	2005	2019
Sweden	15.5 (15.4 to 15.6)	16.9 (16.8 to 17)	17.7 (17.6 to 17.8)	11.3 (10.1 to 12.3)	12.1 (10.8 to 13.2)	12.6 (11.2 to 13.8)	0.27 (0.21 to 0.35)	0.28 (0.22 to 0.36)	0.29 (0.22 to 0.36)
Switzerland	16 (15.9 to 16.1)	17.7 (17.6 to 17.7)	18.7 (18.6 to 18.9)	11.4 (10.2 to 12.5)	12.7 (11.3 to 13.9)	13.4 (12 to 14.7)	0.29 (0.22 to 0.36)	0.28 (0.22 to 0.36)	0.29 (0.22 to 0.36)
United Kingdom	14.3 (14.3 to 14.3)	15.9 (15.8 to 15.9)	16.8 (16.7 to 16.9)	10.4 (9.31 to 11.3)	11.4 (10.2 to 12.5)	11.9 (10.6 to 13.1)	0.27 (0.21 to 0.35)	0.28 (0.21 to 0.35)	0.29 (0.22 to 0.37)
**Latin America and Caribbean**	13.7 (13.7 to 13.8)	15.4 (15.4 to 15.5)	16.1 (15.7 to 16.6)	9.86 (8.8 to 10.8)	11.2 (10 to 12.2)	11.7 (10.4 to 12.8)	0.28 (0.22 to 0.36)	0.28 (0.21 to 0.35)	0.28 (0.23 to 0.33)
Andean Latin America	14.1 (13.8 to 14.5)	15.3 (14.9 to 15.7)	16 (15 to 16.9)	10.4 (9.37 to 11.4)	11.3 (10.1 to 12.4)	11.7 (10.4 to 13)	0.26 (0.21 to 0.32)	0.26 (0.21 to 0.32)	0.27 (0.23 to 0.31)
Bolivia (Plurinational State of)	11.2 (10.8 to 11.6)	12.1 (11.7 to 12.4)	12.1 (11.5 to 12.6)	8.21 (7.36 to 9.05)	8.92 (7.99 to 9.76)	8.88 (7.86 to 9.86)	0.27 (0.22 to 0.32)	0.26 (0.21 to 0.32)	0.26 (0.22 to 0.31)
Ecuador	14 (13.9 to 14.1)	15.1 (14.6 to 15.5)	15.3 (14.2 to 16.4)	10.4 (9.45 to 11.3)	11.1 (10 to 12.2)	11.2 (9.77 to 12.5)	0.26 (0.2 to 0.32)	0.26 (0.21 to 0.32)	0.27 (0.24 to 0.31)
Peru	15.1 (14.5 to 15.8)	16.6 (15.8 to 17.4)	17.7 (16.1 to 19.3)	11.2 (9.97 to 12.4)	12.2 (10.9 to 13.5)	13 (11.4 to 14.7)	0.26 (0.22 to 0.31)	0.26 (0.22 to 0.31)	0.26 (0.24 to 0.29)
Caribbean	13.9 (13.8 to 14)	15 (14.8 to 15.2)	15.6 (14.8 to 16.3)	10.4 (9.38 to 11.3)	11.1 (10 to 12.1)	11.4 (10.2 to 12.6)	0.25 (0.2 to 0.32)	0.26 (0.21 to 0.32)	0.27 (0.23 to 0.31)
Antigua and Barbuda	15.1 (14.6 to 15.5)	14.3 (13.8 to 14.6)	14.1 (13.6 to 14.6)	11.4 (10.3 to 12.4)	10.7 (9.59 to 11.7)	10.5 (9.42 to 11.5)	0.24 (0.2 to 0.29)	0.25 (0.2 to 0.3)	0.26 (0.21 to 0.31)
Bahamas	14.5 (14.2 to 14.9)	15.2 (14.8 to 15.6)	15.5 (14.4 to 16.5)	11 (9.96 to 12)	11.4 (10.3 to 12.5)	11.6 (10.2 to 12.9)	0.24 (0.2 to 0.3)	0.25 (0.2 to 0.3)	0.25 (0.22 to 0.29)
Barbados	13.9 (13.7 to 14.2)	14.2 (13.9 to 14.5)	14.4 (13.7 to 15.3)	10.6 (9.64 to 11.5)	10.7 (9.65 to 11.7)	10.8 (9.59 to 11.9)	0.24 (0.19 to 0.29)	0.25 (0.2 to 0.31)	0.25 (0.22 to 0.3)
Belize	14 (13.6 to 14.4)	14.7 (14.4 to 14.9)	16 (15.3 to 16.7)	10.6 (9.58 to 11.5)	11 (9.98 to 11.9)	11.9 (10.7 to 13.1)	0.24 (0.2 to 0.3)	0.25 (0.2 to 0.31)	0.25 (0.21 to 0.3)
Bermuda	13.7 (13.5 to 13.9)	17.1 (16.2 to 18)	18.7 (17.5 to 19.6)	10.5 (9.51 to 11.3)	12.9 (11.5 to 14.2)	14.1 (12.5 to 15.5)	0.24 (0.19 to 0.3)	0.24 (0.21 to 0.29)	0.25 (0.21 to 0.28)
Cuba	14.5 (14.4 to 14.6)	15.5 (15.4 to 15.6)	16.2 (15.2 to 17.4)	10.7 (9.6 to 11.6)	11.4 (10.2 to 12.4)	11.7 (10.3 to 13.1)	0.26 (0.2 to 0.33)	0.27 (0.21 to 0.34)	0.28 (0.24 to 0.32)
Dominica	12.7 (12.3 to 13.2)	13.6 (13 to 14.3)	13.4 (12.6 to 14.1)	9.58 (8.66 to 10.5)	10.2 (9.13 to 11.2)	9.99 (8.85 to 11.1)	0.24 (0.2 to 0.3)	0.25 (0.22 to 0.3)	0.26 (0.21 to 0.3)
Dominican Republic	15.6 (15 to 16.1)	15.8 (15.1 to 16.6)	15.1 (14 to 16)	11.9 (10.8 to 13)	12 (10.7 to 13.1)	11.4 (10.1 to 12.6)	0.24 (0.2 to 0.28)	0.24 (0.21 to 0.29)	0.25 (0.21 to 0.28)
Grenada	12.7 (12.3 to 13.1)	14.5 (14.1 to 14.8)	13.5 (13.1 to 13.9)	9.59 (8.61 to 10.5)	10.8 (9.73 to 11.8)	10 (8.97 to 11)	0.25 (0.2 to 0.3)	0.25 (0.2 to 0.31)	0.26 (0.21 to 0.32)
Guyana	10.2 (9.65 to 10.9)	11.5 (11 to 12.1)	12.3 (11.2 to 13.5)	7.52 (6.64 to 8.39)	8.44 (7.46 to 9.36)	8.97 (7.76 to 10.3)	0.26 (0.23 to 0.31)	0.27 (0.23 to 0.32)	0.27 (0.24 to 0.31)
Haiti	9.81 (9.43 to 10.2)	10.3 (9.68 to 10.9)	11.1 (9.69 to 12.2)	7.36 (6.63 to 8.11)	7.7 (6.82 to 8.56)	8.16 (6.88 to 9.35)	0.25 (0.21 to 0.3)	0.25 (0.22 to 0.3)	0.26 (0.24 to 0.29)
Jamaica	15.1 (14.9 to 15.3)	16.6 (16.3 to 16.9)	15.7 (14.5 to 17)	11.4 (10.4 to 12.4)	12.4 (11.1 to 13.5)	11.7 (10.3 to 13.2)	0.24 (0.19 to 0.3)	0.26 (0.2 to 0.32)	0.26 (0.22 to 0.29)
Puerto Rico	15.1 (14.9 to 15.2)	17.1 (16.9 to 17.3)	18.4 (17 to 19.9)	11.2 (10.2 to 12.2)	12.5 (11.3 to 13.8)	13.5 (11.8 to 15.2)	0.25 (0.2 to 0.32)	0.27 (0.21 to 0.33)	0.27 (0.24 to 0.31)
Saint Kitts and Nevis	10 (9.68 to 10.4)	12.9 (12.5 to 13.3)	13.3 (12.5 to 13.9)	7.47 (6.72 to 8.2)	9.54 (8.52 to 10.5)	9.82 (8.7 to 10.9)	0.26 (0.21 to 0.31)	0.26 (0.21 to 0.32)	0.26 (0.22 to 0.3)
Saint Lucia	12.4 (12.1 to 12.6)	14.9 (14.6 to 15.3)	15.5 (14.7 to 16.3)	9.08 (8.11 to 9.95)	10.9 (9.81 to 12)	11.2 (9.93 to 12.5)	0.27 (0.21 to 0.33)	0.27 (0.21 to 0.33)	0.28 (0.23 to 0.33)
Saint Vincent and the Grenadines	12.1 (11.9 to 12.3)	14.2 (13.9 to 14.5)	13.3 (12.7 to 13.9)	9.1 (8.24 to 9.87)	10.5 (9.5 to 11.5)	9.82 (8.71 to 10.9)	0.25 (0.2 to 0.31)	0.26 (0.21 to 0.31)	0.26 (0.22 to 0.31)
Suriname	13.5 (13.3 to 13.8)	14.9 (14.6 to 15.2)	15 (14.2 to 15.9)	10.1 (9.08 to 11)	10.9 (9.81 to 11.9)	10.9 (9.65 to 12.2)	0.26 (0.21 to 0.32)	0.27 (0.21 to 0.33)	0.27 (0.24 to 0.32)
Trinidad and Tobago	12.5 (12.3 to 12.7)	14.6 (14.3 to 14.8)	15.7 (14.1 to 17.3)	9.32 (8.4 to 10.1)	10.8 (9.72 to 11.8)	11.6 (10 to 13.3)	0.26 (0.2 to 0.32)	0.26 (0.2 to 0.32)	0.26 (0.23 to 0.29)
United States Virgin Islands	13.5 (13 to 14.2)	14.2 (13.9 to 14.5)	14.6 (14.1 to 15.2)	10.3 (9.32 to 11.2)	10.7 (9.72 to 11.7)	10.9 (9.84 to 12)	0.24 (0.21 to 0.28)	0.24 (0.2 to 0.3)	0.25 (0.21 to 0.3)
Central Latin America	13.8 (13.7 to 13.8)	15.4 (15.4 to 15.5)	16.2 (15.5 to 16.9)	9.76 (8.67 to 10.8)	11.1 (9.94 to 12.2)	11.6 (10.3 to 12.8)	0.29 (0.22 to 0.37)	0.28 (0.21 to 0.35)	0.28 (0.24 to 0.34)
Colombia	14.1 (14 to 14.1)	16.5 (16.4 to 16.5)	18.3 (16.9 to 19.8)	10.2 (9.12 to 11.1)	12 (10.8 to 13.1)	13.4 (11.8 to 15.1)	0.28 (0.21 to 0.35)	0.27 (0.21 to 0.34)	0.27 (0.24 to 0.3)
Costa Rica	15.7 (15.5 to 15.9)	17 (16.8 to 17.2)	17.8 (16.4 to 19.2)	11.3 (10.1 to 12.4)	12.3 (11 to 13.5)	12.8 (11.2 to 14.5)	0.28 (0.22 to 0.35)	0.28 (0.22 to 0.34)	0.28 (0.24 to 0.31)
El Salvador	15.9 (15.8 to 16.1)	16.8 (16.7 to 17)	16.5 (15 to 18.2)	11.6 (10.4 to 12.7)	12.2 (11 to 13.4)	12 (10.4 to 13.7)	0.28 (0.21 to 0.34)	0.28 (0.21 to 0.34)	0.27 (0.25 to 0.31)
Guatemala	10.7 (10.2 to 11.2)	13.1 (12.5 to 13.7)	14.6 (13.5 to 15.7)	7.74 (6.88 to 8.64)	9.42 (8.33 to 10.5)	10.4 (9.13 to 11.8)	0.28 (0.23 to 0.33)	0.28 (0.24 to 0.34)	0.28 (0.25 to 0.32)
Honduras	15.5 (14.7 to 16.3)	12.7 (11.6 to 13.8)	12.5 (11.7 to 12.8)	11.1 (9.83 to 12.4)	9.29 (8.05 to 10.5)	9.07 (8.04 to 9.96)	0.28 (0.24 to 0.33)	0.27 (0.24 to 0.31)	0.27 (0.22 to 0.31)
Mexico	13.4 (13.3 to 13.4)	14.9 (14.9 to 14.9)	15.6 (14.9 to 16.2)	9.28 (8.18 to 10.3)	10.6 (9.45 to 11.6)	11 (9.69 to 12.3)	0.31 (0.23 to 0.39)	0.29 (0.22 to 0.36)	0.29 (0.24 to 0.35)
Nicaragua	18.5 (18.2 to 18.7)	13.2 (13.1 to 13.4)	14.3 (13.8 to 14.9)	13.2 (11.8 to 14.5)	9.75 (8.81 to 10.6)	10.5 (9.39 to 11.5)	0.29 (0.22 to 0.35)	0.26 (0.21 to 0.33)	0.27 (0.23 to 0.32)
Panama	16.5 (16.2 to 16.7)	17.4 (17.2 to 17.7)	18.5 (17.1 to 19.9)	12 (10.7 to 13.1)	12.7 (11.4 to 13.9)	13.4 (11.7 to 15.1)	0.27 (0.22 to 0.34)	0.27 (0.21 to 0.34)	0.28 (0.24 to 0.31)
Venezuela (Bolivarian Republic of)	14.5 (14.5 to 14.6)	16.9 (16.8 to 17)	16.2 (14.6 to 17.7)	10.5 (9.38 to 11.5)	12.2 (11 to 13.4)	11.7 (10.2 to 13.4)	0.28 (0.21 to 0.35)	0.28 (0.21 to 0.35)	0.27 (0.24 to 0.3)
Tropical Latin America	13.4 (13.3 to 13.5)	15.5 (15.4 to 15.6)	16.3 (16.1 to 16.5)	9.56 (8.52 to 10.5)	11.2 (9.95 to 12.2)	11.8 (10.6 to 12.9)	0.29 (0.22 to 0.36)	0.28 (0.22 to 0.35)	0.28 (0.22 to 0.34)

Supplementary Table 8: Life expectancy, Health-adjusted life expectancy, and proportion of years spent in ill health in all reporting locations, in 1990, 2005, 2019, Females									
	LE-70			HALE-70			PYIH-70		
location	1990	2005	2019	1990	2005	2019	1990	2005	2019
Brazil	13.4 (13.3 to 13.5)	15.5 (15.4 to 15.6)	16.3 (16.1 to 16.5)	9.5 (8.47 to 10.5)	11.1 (9.94 to 12.2)	11.8 (10.6 to 12.9)	0.29 (0.22 to 0.36)	0.28 (0.22 to 0.36)	0.28 (0.22 to 0.34)
Paraguay	16 (15.4 to 16.7)	16.1 (15.5 to 16.7)	16.4 (15 to 17.9)	11.8 (10.4 to 13)	11.9 (10.6 to 13.1)	12 (10.5 to 13.6)	0.27 (0.22 to 0.32)	0.26 (0.22 to 0.31)	0.27 (0.24 to 0.3)
**North Africa and Middle East**	11.7 (11.5 to 11.9)	13 (12.7 to 13.2)	13.3 (12.9 to 13.8)	8.42 (7.52 to 9.26)	9.26 (8.28 to 10.2)	9.47 (8.43 to 10.5)	0.28 (0.22 to 0.35)	0.29 (0.23 to 0.35)	0.29 (0.24 to 0.35)
North Africa and Middle East	11.7 (11.5 to 11.9)	13 (12.7 to 13.2)	13.3 (12.9 to 13.8)	8.42 (7.52 to 9.26)	9.26 (8.28 to 10.2)	9.47 (8.43 to 10.5)	0.28 (0.22 to 0.35)	0.29 (0.23 to 0.35)	0.29 (0.24 to 0.35)
Afghanistan	9.39 (8.73 to 10.1)	9.44 (8.4 to 10.3)	9.61 (8.73 to 10.7)	6.47 (5.55 to 7.3)	6.5 (5.47 to 7.43)	6.53 (5.54 to 7.55)	0.31 (0.28 to 0.36)	0.31 (0.28 to 0.35)	0.32 (0.29 to 0.37)
Algeria	10.9 (10.3 to 11.4)	12.4 (11.7 to 13.1)	13.2 (12.6 to 13.8)	7.97 (7.06 to 8.86)	8.99 (8.03 to 10)	9.49 (8.4 to 10.5)	0.27 (0.23 to 0.32)	0.27 (0.23 to 0.32)	0.28 (0.24 to 0.33)
Bahrain	9.91 (9.45 to 10.4)	9.97 (9.52 to 10.4)	12.2 (11.4 to 13.1)	6.96 (6.15 to 7.73)	6.95 (6.12 to 7.79)	8.45 (7.34 to 9.56)	0.3 (0.26 to 0.35)	0.3 (0.25 to 0.36)	0.31 (0.27 to 0.36)
Egypt	9.91 (9.88 to 9.94)	9.86 (9.83 to 9.89)	9.88 (8.97 to 10.9)	7.26 (6.53 to 7.92)	7.18 (6.46 to 7.84)	7.15 (6.2 to 8.16)	0.27 (0.2 to 0.34)	0.27 (0.21 to 0.34)	0.28 (0.25 to 0.31)
Iran (Islamic Republic of)	12.4 (12.1 to 12.6)	13.6 (13.5 to 13.8)	14.6 (14.4 to 14.8)	8.7 (7.73 to 9.61)	9.61 (8.57 to 10.6)	10.2 (9.07 to 11.3)	0.3 (0.24 to 0.36)	0.3 (0.23 to 0.37)	0.3 (0.23 to 0.37)
Iraq	12.5 (11.7 to 13.4)	12.3 (11.2 to 13.3)	13.7 (12.9 to 14.5)	8.63 (7.52 to 9.74)	8.66 (7.45 to 9.8)	9.67 (8.5 to 10.8)	0.31 (0.28 to 0.36)	0.29 (0.27 to 0.34)	0.29 (0.26 to 0.34)
Jordan	10.9 (10.3 to 11.5)	11 (10.4 to 11.6)	14.5 (13.3 to 15.5)	7.83 (6.92 to 8.71)	7.89 (6.97 to 8.81)	10.4 (9.05 to 11.7)	0.28 (0.24 to 0.33)	0.28 (0.24 to 0.33)	0.28 (0.25 to 0.32)
Kuwait	14.5 (14.3 to 14.8)	17.1 (16.8 to 17.3)	18.3 (17.2 to 19.4)	10.5 (9.4 to 11.6)	12.1 (10.8 to 13.3)	13 (11.4 to 14.7)	0.28 (0.22 to 0.34)	0.29 (0.23 to 0.36)	0.29 (0.24 to 0.34)
Lebanon	12.4 (11.7 to 12.9)	13.7 (13.3 to 14.1)	14.9 (14.5 to 15.3)	8.9 (7.89 to 9.84)	9.79 (8.73 to 10.8)	10.5 (9.24 to 11.6)	0.28 (0.23 to 0.33)	0.29 (0.24 to 0.34)	0.3 (0.24 to 0.36)
Libya	14.5 (13.7 to 15.5)	16.2 (16 to 16.6)	15.2 (14.1 to 16.4)	10.4 (9.14 to 11.6)	11.4 (10.1 to 12.7)	10.7 (9.24 to 12.1)	0.29 (0.25 to 0.33)	0.3 (0.24 to 0.36)	0.3 (0.26 to 0.34)
Morocco	11.9 (11.7 to 12.3)	12 (11.3 to 12.6)	12.6 (12.1 to 13.5)	8.67 (7.79 to 9.53)	8.64 (7.66 to 9.63)	9.06 (8.05 to 10.2)	0.27 (0.22 to 0.33)	0.28 (0.23 to 0.32)	0.28 (0.24 to 0.33)
Oman	10.1 (9.22 to 11.2)	10.9 (10.6 to 11.2)	11.3 (10.7 to 11.8)	7.24 (6.29 to 8.3)	7.76 (6.86 to 8.58)	7.97 (7.03 to 8.91)	0.28 (0.26 to 0.32)	0.29 (0.23 to 0.35)	0.29 (0.25 to 0.34)
Palestine	11.3 (10.3 to 12.6)	12.5 (12.1 to 13)	12.3 (11.6 to 13)	8.13 (7.03 to 9.31)	8.95 (7.94 to 9.85)	8.74 (7.71 to 9.8)	0.28 (0.26 to 0.32)	0.29 (0.24 to 0.34)	0.29 (0.25 to 0.34)
Qatar	10.2 (9.43 to 11)	8.8 (8.16 to 9.5)	9.66 (8.99 to 10.4)	7.13 (6.24 to 8.08)	6.01 (5.17 to 6.84)	6.68 (5.77 to 7.6)	0.3 (0.26 to 0.34)	0.32 (0.28 to 0.37)	0.31 (0.27 to 0.36)
Saudi Arabia	11.4 (10.8 to 12.1)	12.5 (12.1 to 12.8)	13.7 (12.6 to 14.9)	7.79 (6.82 to 8.81)	8.54 (7.44 to 9.56)	9.41 (8.07 to 10.7)	0.32 (0.27 to 0.37)	0.31 (0.25 to 0.38)	0.31 (0.28 to 0.36)
Sudan	11.2 (10.8 to 11.6)	12.1 (11.4 to 12.9)	12.5 (11.8 to 12.8)	8.06 (7.2 to 8.87)	8.67 (7.67 to 9.63)	8.95 (7.89 to 9.92)	0.28 (0.23 to 0.33)	0.28 (0.25 to 0.33)	0.28 (0.23 to 0.33)
Syrian Arab Republic	11.6 (10.8 to 12.5)	11.9 (11.3 to 12.6)	11.8 (10.8 to 12.7)	8.44 (7.42 to 9.45)	8.69 (7.67 to 9.64)	8.42 (7.27 to 9.53)	0.27 (0.25 to 0.31)	0.27 (0.24 to 0.32)	0.29 (0.25 to 0.33)
Tunisia	13.7 (13.2 to 14.3)	14.3 (13.2 to 15.6)	15.1 (13.8 to 16.5)	10 (8.93 to 11)	10.4 (9.06 to 11.8)	10.8 (9.38 to 12.3)	0.27 (0.23 to 0.32)	0.27 (0.25 to 0.31)	0.28 (0.26 to 0.32)
Turkey	13.5 (12.9 to 14.1)	16.8 (16.1 to 17.6)	15.3 (14.2 to 16.5)	9.71 (8.6 to 10.8)	11.9 (10.5 to 13.2)	10.9 (9.53 to 12.3)	0.28 (0.24 to 0.33)	0.3 (0.25 to 0.35)	0.29 (0.25 to 0.33)
United Arab Emirates	9.69 (9.13 to 10.3)	7.32 (6.97 to 7.71)	13 (11.9 to 13.9)	6.83 (6.01 to 7.68)	5.14 (4.51 to 5.73)	8.93 (7.67 to 10.1)	0.3 (0.25 to 0.34)	0.3 (0.26 to 0.35)	0.31 (0.27 to 0.35)
Yemen	11.1 (10.3 to 11.7)	11.9 (11 to 12.5)	12.1 (11.1 to 12.7)	7.95 (6.99 to 8.83)	8.53 (7.55 to 9.57)	8.68 (7.5 to 9.7)	0.28 (0.24 to 0.32)	0.28 (0.23 to 0.32)	0.28 (0.24 to 0.32)
**South Asia**	9.73 (9.47 to 10)	11.2 (10.9 to 11.4)	12.8 (12 to 13.7)	6.54 (5.74 to 7.33)	7.53 (6.59 to 8.39)	8.59 (7.47 to 9.76)	0.33 (0.27 to 0.39)	0.33 (0.27 to 0.39)	0.33 (0.29 to 0.38)
South Asia	9.73 (9.47 to 10)	11.2 (10.9 to 11.4)	12.8 (12 to 13.7)	6.54 (5.74 to 7.33)	7.53 (6.59 to 8.39)	8.59 (7.47 to 9.76)	0.33 (0.27 to 0.39)	0.33 (0.27 to 0.39)	0.33 (0.29 to 0.38)
Bangladesh	11.1 (10.7 to 11.5)	12 (11.6 to 12.4)	14.3 (13.3 to 15.4)	7.83 (6.93 to 8.65)	8.52 (7.58 to 9.44)	10.2 (8.94 to 11.5)	0.29 (0.25 to 0.35)	0.29 (0.24 to 0.34)	0.29 (0.25 to 0.33)
Bhutan	10.9 (10.3 to 11.2)	12.2 (11.8 to 12.7)	12.6 (12.4 to 13.4)	7.7 (6.86 to 8.5)	8.65 (7.76 to 9.55)	8.96 (7.99 to 9.9)	0.29 (0.24 to 0.33)	0.29 (0.25 to 0.34)	0.29 (0.26 to 0.36)
India	9.38 (9.09 to 9.7)	11.1 (10.8 to 11.5)	12.7 (11.8 to 13.8)	6.22 (5.42 to 7.01)	7.43 (6.5 to 8.32)	8.49 (7.31 to 9.74)	0.34 (0.28 to 0.4)	0.33 (0.27 to 0.4)	0.33 (0.29 to 0.38)
Nepal	10.6 (9.97 to 11)	12.1 (11.9 to 12.5)	12.1 (11.7 to 12.9)	7.33 (6.47 to 8.19)	8.41 (7.51 to 9.26)	8.47 (7.47 to 9.51)	0.31 (0.26 to 0.35)	0.3 (0.26 to 0.37)	0.3 (0.26 to 0.36)
Pakistan	11 (10.6 to 11.3)	10.6 (10.3 to 11.1)	11.6 (10.4 to 12.5)	7.64 (6.76 to 8.46)	7.35 (6.45 to 8.19)	8 (6.89 to 9.17)	0.3 (0.25 to 0.36)	0.31 (0.26 to 0.37)	0.31 (0.27 to 0.34)
**Southeast Asia, East Asia, and Oceania**	11.6 (11.2 to 12.2)	12.9 (12.5 to 13.2)	14.8 (14 to 15.7)	8.25 (7.31 to 9.15)	9.1 (8.09 to 10)	10.4 (9.21 to 11.6)	0.29 (0.25 to 0.34)	0.29 (0.24 to 0.35)	0.3 (0.26 to 0.34)
East Asia	11.4 (10.9 to 12.2)	12.9 (12.4 to 13.3)	15.1 (14.1 to 16.2)	8.15 (7.2 to 9.09)	9.12 (8.08 to 10.1)	10.6 (9.33 to 12)	0.29 (0.25 to 0.34)	0.29 (0.25 to 0.35)	0.3 (0.26 to 0.34)
China	11.4 (10.8 to 12.1)	12.9 (12.4 to 13.3)	15.1 (14.1 to 16.3)	8.12 (7.16 to 9.05)	9.09 (8.04 to 10)	10.6 (9.3 to 11.9)	0.29 (0.25 to 0.34)	0.29 (0.25 to 0.35)	0.3 (0.27 to 0.34)
Democratic People's Republic of Korea	12.3 (11.7 to 13)	12.2 (11.9 to 12.8)	13.8 (12.7 to 14.5)	8.86 (7.98 to 9.76)	8.79 (7.97 to 9.64)	9.94 (8.82 to 11)	0.28 (0.25 to 0.31)	0.28 (0.25 to 0.33)	0.28 (0.24 to 0.31)
Taiwan (Province of China)	13.7 (13.6 to 13.8)	16.1 (16 to 16.2)	17.5 (16 to 18.9)	9.8 (8.82 to 10.7)	11.6 (10.4 to 12.7)	12.6 (11 to 14.2)	0.28 (0.22 to 0.35)	0.28 (0.22 to 0.35)	0.28 (0.25 to 0.31)
Oceania	11.2 (10.7 to 11.7)	11.2 (10.5 to 11.8)	11.5 (10.4 to 12.3)	7.86 (6.95 to 8.73)	7.78 (6.81 to 8.71)	7.94 (6.84 to 9.03)	0.3 (0.25 to 0.35)	0.3 (0.26 to 0.35)	0.31 (0.27 to 0.34)
American Samoa	12.7 (12.3 to 13.1)	13.1 (12.6 to 13.5)	13 (12 to 14)	8.82 (7.8 to 9.75)	8.99 (7.88 to 9.97)	8.85 (7.56 to 10.1)	0.3 (0.26 to 0.37)	0.32 (0.26 to 0.38)	0.32 (0.28 to 0.37)

Supplementary Table 8: Life expectancy, Health-adjusted life expectancy, and proportion of years spent in ill health in all reporting locations, in 1990, 2005, 2019, Females

location	LE-70			HALE-70			PYIH-70		
	1990	2005	2019	1990	2005	2019	1990	2005	2019
Cook Islands	12<br>(11.3 to 12.5)	14.4<br>(13.8 to 14.8)	14.7<br>(13.6 to 15.8)	8.32<br>(7.27 to 9.28)	9.76<br>(8.62 to 10.9)	9.99<br>(8.58 to 11.4)	0.31<br>(0.26 to 0.36)	0.32<br>(0.26 to 0.38)	0.32<br>(0.28 to 0.37)
Fiji	11.2<br>(10.4 to 12)	11.4<br>(11.2 to 11.5)	11.5<br>(10.4 to 12.7)	7.84<br>(6.85 to 8.89)	7.81<br>(6.86 to 8.71)	7.93<br>(6.76 to 9.13)	0.3<br>(0.26 to 0.34)	0.31<br>(0.24 to 0.39)	0.31<br>(0.28 to 0.35)
Guam	13.1<br>(12.7 to 13.5)	18.2<br>(17.6 to 18.7)	17.2<br>(16.1 to 18.1)	9.4<br>(8.41 to 10.3)	12.8<br>(11.4 to 14.1)	12.1<br>(10.6 to 13.5)	0.28<br>(0.24 to 0.34)	0.3<br>(0.24 to 0.35)	0.3<br>(0.25 to 0.34)
Kiribati	8.46<br>(7.88 to 9.18)	8.74<br>(8.23 to 9.42)	9.15<br>(8.45 to 10)	6.09<br>(5.34 to 6.89)	6.3<br>(5.54 to 7.04)	6.54<br>(5.67 to 7.42)	0.28<br>(0.25 to 0.32)	0.28<br>(0.25 to 0.33)	0.29<br>(0.26 to 0.33)
Marshall Islands	10.1<br>(9.75 to 10.5)	9.5<br>(9.07 to 10)	10.1<br>(9.05 to 11.2)	7.15<br>(6.34 to 7.96)	6.64<br>(5.84 to 7.47)	6.97<br>(5.87 to 8.13)	0.29<br>(0.24 to 0.35)	0.3<br>(0.26 to 0.36)	0.31<br>(0.27 to 0.35)
Micronesia (Federated States of)	9.55<br>(8.79 to 10.4)	9.81<br>(9.32 to 10.4)	9.77<br>(8.98 to 10.7)	6.88<br>(5.98 to 7.8)	6.96<br>(6.12 to 7.74)	6.92<br>(5.97 to 7.9)	0.28<br>(0.25 to 0.32)	0.29<br>(0.26 to 0.34)	0.29<br>(0.26 to 0.33)
Nauru	9.67<br>(8.97 to 10.6)	8.98<br>(8.37 to 9.73)	9.8<br>(8.98 to 10.7)	6.88<br>(6.04 to 7.74)	6.38<br>(5.62 to 7.21)	6.84<br>(5.89 to 7.81)	0.29<br>(0.27 to 0.33)	0.29<br>(0.26 to 0.33)	0.3<br>(0.27 to 0.34)
Niue	11.8<br>(10.8 to 12.6)	12<br>(11 to 12.7)	12.7<br>(11.5 to 13.8)	8.29<br>(7.19 to 9.32)	8.28<br>(7.19 to 9.35)	8.63<br>(7.38 to 9.86)	0.3<br>(0.26 to 0.34)	0.31<br>(0.26 to 0.35)	0.32<br>(0.28 to 0.36)
Northern Mariana Islands	11.8<br>(10.8 to 12.9)	13<br>(12.1 to 14)	13.7<br>(12.6 to 14.7)	8.44<br>(7.36 to 9.62)	9.17<br>(8.06 to 10.3)	9.63<br>(8.31 to 10.8)	0.29<br>(0.25 to 0.32)	0.3<br>(0.26 to 0.34)	0.3<br>(0.26 to 0.34)
Palau	10.8<br>(9.68 to 12)	11.1<br>(10.2 to 12.3)	11.3<br>(10.3 to 12.8)	7.56<br>(6.5 to 8.75)	7.65<br>(6.65 to 8.79)	7.75<br>(6.61 to 9.07)	0.3<br>(0.27 to 0.33)	0.31<br>(0.29 to 0.35)	0.32<br>(0.29 to 0.36)
Papua New Guinea	11.5<br>(10.8 to 12.1)	11.1<br>(10.2 to 11.9)	11.4<br>(10.2 to 12.3)	7.98<br>(6.99 to 8.92)	7.72<br>(6.66 to 8.7)	7.87<br>(6.75 to 8.98)	0.3<br>(0.27 to 0.36)	0.31<br>(0.27 to 0.35)	0.31<br>(0.27 to 0.34)
Samoa	11.5<br>(10.5 to 12)	11.8<br>(10.7 to 12.8)	11.9<br>(10.7 to 13.1)	8.21<br>(7.09 to 9.16)	8.3<br>(7.12 to 9.46)	8.31<br>(7.09 to 9.52)	0.29<br>(0.24 to 0.33)	0.3<br>(0.26 to 0.33)	0.3<br>(0.28 to 0.34)
Solomon Islands	8.02<br>(7.49 to 8.7)	7.98<br>(7.54 to 8.53)	8.32<br>(7.75 to 8.97)	5.84<br>(5.16 to 6.58)	5.78<br>(5.11 to 6.44)	5.97<br>(5.22 to 6.71)	0.27<br>(0.24 to 0.31)	0.28<br>(0.25 to 0.32)	0.28<br>(0.25 to 0.33)
Tokelau	10.4<br>(9.43 to 11.2)	11<br>(9.88 to 12.1)	11.5<br>(10.3 to 12.8)	7.41<br>(6.42 to 8.37)	7.7<br>(6.59 to 8.82)	8<br>(6.8 to 9.22)	0.29<br>(0.25 to 0.32)	0.3<br>(0.27 to 0.33)	0.3<br>(0.28 to 0.34)
Tonga	14<br>(13.3 to 14.7)	14.7<br>(14 to 15.4)	14.4<br>(13.1 to 15.5)	9.91<br>(8.78 to 11)	10.4<br>(9.22 to 11.6)	10.1<br>(8.74 to 11.4)	0.29<br>(0.25 to 0.34)	0.29<br>(0.25 to 0.34)	0.3<br>(0.26 to 0.33)
Tuvalu	10.1<br>(9.26 to 11)	10.4<br>(9.63 to 11.4)	10.9<br>(9.67 to 12.3)	7.22<br>(6.32 to 8.18)	7.36<br>(6.42 to 8.29)	7.63<br>(6.39 to 8.93)	0.29<br>(0.25 to 0.32)	0.3<br>(0.27 to 0.33)	0.3<br>(0.28 to 0.34)
Vanuatu	10.1<br>(9.12 to 11.4)	10.2<br>(9.17 to 11.6)	10.5<br>(9.37 to 11.8)	7.23<br>(6.23 to 8.31)	7.29<br>(6.29 to 8.45)	7.42<br>(6.24 to 8.65)	0.28<br>(0.27 to 0.32)	0.29<br>(0.27 to 0.31)	0.3<br>(0.27 to 0.33)
Southeast Asia	12.3<br>(12 to 12.5)	12.8<br>(12.5 to 13.1)	13.7<br>(13.2 to 14.3)	8.61<br>(7.66 to 9.52)	9.06<br>(8.09 to 10)	9.67<br>(8.59 to 10.7)	0.3<br>(0.24 to 0.36)	0.29<br>(0.24 to 0.35)	0.3<br>(0.25 to 0.35)
Cambodia	10.7<br>(10.3 to 11.2)	11.6<br>(11.2 to 12.1)	12.2<br>(11.9 to 13)	7.52<br>(6.63 to 8.35)	8.23<br>(7.29 to 9.12)	8.58<br>(7.57 to 9.59)	0.29<br>(0.25 to 0.36)	0.29<br>(0.25 to 0.35)	0.3<br>(0.26 to 0.36)
Indonesia	11.7<br>(11.2 to 12)	11.6<br>(11.2 to 12.1)	12.1<br>(11.5 to 13.2)	7.91<br>(6.87 to 8.83)	7.98<br>(7 to 8.94)	8.36<br>(7.32 to 9.4)	0.32<br>(0.27 to 0.39)	0.32<br>(0.26 to 0.37)	0.31<br>(0.29 to 0.36)
Lao People's Democratic Republic	10.1<br>(9.5 to 10.8)	11.2<br>(10.7 to 11.7)	12.1<br>(11.5 to 12.9)	7.28<br>(6.47 to 8.2)	8.05<br>(7.19 to 8.89)	8.67<br>(7.69 to 9.64)	0.28<br>(0.24 to 0.32)	0.28<br>(0.24 to 0.33)	0.28<br>(0.25 to 0.33)
Malaysia	12.8<br>(12.7 to 12.8)	12.6<br>(12.5 to 12.6)	13.3<br>(12.2 to 14.4)	8.95<br>(7.95 to 9.87)	8.87<br>(7.92 to 9.77)	9.35<br>(8.14 to 10.6)	0.3<br>(0.23 to 0.37)	0.29<br>(0.23 to 0.37)	0.29<br>(0.27 to 0.33)
Maldives	10.5<br>(10.3 to 10.6)	13.4<br>(13.1 to 13.8)	15.3<br>(14.4 to 16.4)	7.45<br>(6.68 to 8.21)	9.58<br>(8.6 to 10.5)	10.9<br>(9.6 to 12.2)	0.29<br>(0.23 to 0.35)	0.29<br>(0.23 to 0.34)	0.29<br>(0.26 to 0.34)
Mauritius	12.8<br>(12.5 to 13)	14.2<br>(13.9 to 14.4)	15.2<br>(14.1 to 16.4)	9.02<br>(8.04 to 9.98)	9.88<br>(8.71 to 10.9)	10.6<br>(9.16 to 12)	0.29<br>(0.23 to 0.36)	0.3<br>(0.24 to 0.37)	0.31<br>(0.27 to 0.35)
Myanmar	10.6<br>(9.92 to 11.3)	11.4<br>(10.9 to 12)	12.6<br>(12.2 to 12.9)	7.51<br>(6.55 to 8.41)	8.06<br>(7.17 to 8.97)	8.79<br>(7.76 to 9.72)	0.29<br>(0.26 to 0.34)	0.29<br>(0.25 to 0.34)	0.3<br>(0.24 to 0.36)
Philippines	12.8<br>(12.4 to 13.2)	13.2<br>(12.8 to 13.6)	13.5<br>(12.3 to 14.7)	9.07<br>(8 to 10)	9.31<br>(8.25 to 10.3)	9.54<br>(8.35 to 10.9)	0.29<br>(0.24 to 0.36)	0.29<br>(0.24 to 0.35)	0.29<br>(0.26 to 0.32)
Seychelles	13.9<br>(13.6 to 14.3)	13.9<br>(13.5 to 14.2)	13.9<br>(13.3 to 14.6)	10.1<br>(9 to 11.1)	9.88<br>(8.78 to 10.9)	9.78<br>(8.59 to 10.9)	0.28<br>(0.23 to 0.34)	0.29<br>(0.23 to 0.35)	0.3<br>(0.25 to 0.35)
Sri Lanka	12.7<br>(12.4 to 13.1)	13.7<br>(13.1 to 14.2)	15<br>(13.6 to 16.6)	9.17<br>(8.15 to 10.1)	9.59<br>(8.46 to 10.7)	10.4<br>(8.88 to 12)	0.28<br>(0.23 to 0.34)	0.3<br>(0.25 to 0.36)	0.31<br>(0.28 to 0.35)
Thailand	14.2<br>(13.6 to 14.8)	15.8<br>(15.2 to 16.5)	17.6<br>(16 to 19.3)	10.1<br>(9.03 to 11.2)	11.3<br>(10.1 to 12.6)	12.7<br>(11 to 14.4)	0.29<br>(0.25 to 0.34)	0.28<br>(0.24 to 0.33)	0.28<br>(0.25 to 0.31)
Timor-Leste	11.1<br>(10.6 to 11.4)	12<br>(11.6 to 12.2)	12<br>(11.6 to 12.7)	7.83<br>(6.94 to 8.66)	8.41<br>(7.45 to 9.26)	8.43<br>(7.4 to 9.48)	0.3<br>(0.24 to 0.35)	0.3<br>(0.24 to 0.36)	0.3<br>(0.25 to 0.36)
Viet Nam	12.6<br>(12.1 to 13.2)	13.7<br>(12.9 to 14.1)	14.3<br>(13.8 to 15.2)	9.17<br>(8.24 to 10.1)	9.96<br>(8.84 to 10.9)	10.3<br>(9.1 to 11.4)	0.27<br>(0.23 to 0.32)	0.27<br>(0.22 to 0.31)	0.28<br>(0.25 to 0.34)
**Sub-Saharan Africa**	10.9<br>(10.7 to 11.2)	11.2<br>(10.9 to 11.5)	12.2<br>(11.7 to 12.6)	7.86<br>(7.02 to 8.59)	8.09<br>(7.26 to 8.87)	8.86<br>(7.96 to 9.76)	0.28<br>(0.23 to 0.34)	0.28<br>(0.23 to 0.34)	0.27<br>(0.22 to 0.32)
Central Sub-Saharan Africa	10.4<br>(9.86 to 10.9)	10.6<br>(10.1 to 11.1)	11.6<br>(10.7 to 12.4)	7.45<br>(6.63 to 8.26)	7.68<br>(6.89 to 8.47)	8.47<br>(7.43 to 9.45)	0.28<br>(0.24 to 0.33)	0.28<br>(0.24 to 0.32)	0.27<br>(0.24 to 0.3)
Angola	10.1<br>(9.48 to 10.6)	10.7<br>(10.2 to 11.3)	12.1<br>(11.2 to 12.6)	7.33<br>(6.51 to 8.15)	7.82<br>(6.98 to 8.63)	8.85<br>(7.81 to 9.78)	0.27<br>(0.23 to 0.31)	0.27<br>(0.23 to 0.31)	0.27<br>(0.22 to 0.3)
Central African Republic	9.4<br>(8.61 to 10.1)	8.99<br>(7.89 to 10.3)	9.62<br>(8.3 to 11)	6.75<br>(5.85 to 7.61)	6.52<br>(5.48 to 7.71)	7.03<br>(5.79 to 8.29)	0.28<br>(0.25 to 0.32)	0.27<br>(0.25 to 0.31)	0.27<br>(0.25 to 0.3)
Congo	9.59<br>(8.75 to 10.7)	10.1<br>(9.36 to 10.9)	11<br>(9.98 to 11.7)	7.01<br>(6.13 to 8.04)	7.35<br>(6.42 to 8.16)	7.99<br>(6.96 to 8.95)	0.27<br>(0.25 to 0.3)	0.27<br>(0.25 to 0.31)	0.27<br>(0.23 to 0.3)
Democratic Republic of the Congo	10.6<br>(9.99 to 11.1)	10.7<br>(10.2 to 11.2)	11.6<br>(10.6 to 12.6)	7.55<br>(6.68 to 8.4)	7.72<br>(6.91 to 8.56)	8.47<br>(7.41 to 9.53)	0.29<br>(0.25 to 0.33)	0.28<br>(0.24 to 0.32)	0.27<br>(0.24 to 0.3)
Equatorial Guinea	9.82<br>(8.75 to 10.7)	11.8<br>(10.6 to 13.4)	12.2<br>(10.8 to 13.2)	6.93<br>(5.97 to 7.84)	8.36<br>(7.19 to 9.7)	8.7<br>(7.43 to 9.9)	0.29<br>(0.26 to 0.32)	0.29<br>(0.27 to 0.32)	0.28<br>(0.25 to 0.31)
Gabon	11.2<br>(10.7 to 11.6)	11.3<br>(10.4 to 12.1)	12.4<br>(11.6 to 13.4)	8.21<br>(7.33 to 9.04)	8.22<br>(7.23 to 9.17)	9.09<br>(8.06 to 10.2)	0.27<br>(0.22 to 0.31)	0.27<br>(0.24 to 0.3)	0.27<br>(0.24 to 0.3)

Supplementary Table 8: Life expectancy, Health-adjusted life expectancy, and proportion of years spent in ill health in all reporting locations, in 1990, 2005, 2019, Females									
	LE-70			HALE-70			PYIH-70		
location	1990	2005	2019	1990	2005	2019	1990	2005	2019
Eastern Sub-Saharan Africa	10.3 (10 to 10.5)	11.3 (11 to 11.5)	12.2 (11.8 to 12.5)	7.41 (6.62 to 8.11)	8.17 (7.32 to 8.92)	8.9 (8.02 to 9.71)	0.28 (0.23 to 0.34)	0.28 (0.23 to 0.34)	0.27 (0.22 to 0.32)
Burundi	9.44 (8.58 to 10.1)	10.2 (9.53 to 10.9)	11.5 (10.5 to 12.4)	6.95 (6.04 to 7.76)	7.48 (6.6 to 8.31)	8.53 (7.4 to 9.61)	0.26 (0.23 to 0.3)	0.26 (0.24 to 0.31)	0.26 (0.23 to 0.29)
Comoros	10.6 (10 to 11.3)	11.5 (10.9 to 11.8)	12.2 (11.2 to 12.6)	7.82 (7 to 8.69)	8.5 (7.64 to 9.29)	9.08 (8.03 to 9.95)	0.26 (0.23 to 0.3)	0.26 (0.22 to 0.3)	0.26 (0.21 to 0.28)
Djibouti	11.3 (10.5 to 11.9)	11.7 (10.7 to 12.9)	12.3 (11.2 to 12.9)	8.34 (7.39 to 9.17)	8.7 (7.64 to 9.83)	9.16 (8.03 to 10.1)	0.26 (0.23 to 0.3)	0.26 (0.24 to 0.29)	0.26 (0.22 to 0.28)
Eritrea	9.26 (8.54 to 9.94)	10.3 (9.12 to 11.5)	10.8 (9.5 to 11.9)	6.77 (5.94 to 7.59)	7.54 (6.48 to 8.65)	7.98 (6.77 to 9.13)	0.27 (0.24 to 0.3)	0.27 (0.25 to 0.29)	0.26 (0.23 to 0.29)
Ethiopia	8.97 (8.56 to 9.4)	10.8 (10.3 to 11.2)	12.6 (12.3 to 13.1)	6.43 (5.71 to 7.12)	7.74 (6.84 to 8.61)	9.15 (8.17 to 10)	0.28 (0.24 to 0.33)	0.28 (0.23 to 0.33)	0.27 (0.24 to 0.34)
Kenya	11.8 (11.4 to 12.2)	12 (11.6 to 12.6)	12.4 (11.6 to 13.3)	8.41 (7.47 to 9.24)	8.65 (7.72 to 9.51)	9.01 (7.91 to 10)	0.29 (0.24 to 0.34)	0.28 (0.24 to 0.33)	0.27 (0.24 to 0.32)
Madagascar	11 (10.6 to 11.5)	10.7 (10.1 to 11.2)	11.2 (10 to 12.4)	8.06 (7.22 to 8.88)	7.82 (6.98 to 8.59)	8.3 (7.14 to 9.44)	0.27 (0.23 to 0.32)	0.27 (0.23 to 0.31)	0.26 (0.24 to 0.29)
Malawi	10.2 (9.92 to 10.5)	11.3 (10.6 to 11.9)	12.4 (11.8 to 13.1)	7.39 (6.62 to 8.12)	8.24 (7.26 to 9.13)	9.16 (8.16 to 10.1)	0.28 (0.23 to 0.33)	0.27 (0.23 to 0.32)	0.26 (0.23 to 0.31)
Mozambique	10.4 (10.1 to 10.7)	11.6 (11 to 12.4)	11.6 (10.3 to 12.7)	7.48 (6.65 to 8.24)	8.35 (7.41 to 9.24)	8.49 (7.24 to 9.58)	0.28 (0.23 to 0.34)	0.28 (0.26 to 0.33)	0.27 (0.24 to 0.3)
Rwanda	9.29 (8.72 to 9.72)	11.4 (10.8 to 11.8)	12.4 (11.7 to 12.8)	6.83 (6.09 to 7.54)	8.29 (7.43 to 9.14)	9.14 (8.21 to 10)	0.26 (0.22 to 0.3)	0.27 (0.23 to 0.31)	0.26 (0.22 to 0.3)
Somalia	9.43 (8.52 to 10.2)	9.85 (8.6 to 10.8)	10.4 (8.91 to 11.5)	6.96 (6.1 to 7.79)	7.28 (6.22 to 8.3)	7.69 (6.43 to 8.77)	0.26 (0.24 to 0.28)	0.26 (0.23 to 0.28)	0.26 (0.24 to 0.28)
South Sudan	10.9 (10.3 to 11.6)	11.7 (10.9 to 13)	12.3 (11.5 to 13.5)	7.43 (6.46 to 8.39)	7.95 (6.9 to 9.17)	8.39 (7.25 to 9.61)	0.32 (0.28 to 0.37)	0.32 (0.29 to 0.37)	0.32 (0.29 to 0.37)
Uganda	11.2 (10.7 to 12.1)	11.6 (10.9 to 12)	12.6 (12.2 to 13.3)	8.1 (7.21 to 9.07)	8.45 (7.55 to 9.3)	9.28 (8.34 to 10.2)	0.28 (0.25 to 0.32)	0.27 (0.23 to 0.31)	0.26 (0.24 to 0.32)
United Republic of Tanzania	11.1 (10.7 to 11.4)	11.9 (11.5 to 12.4)	12.4 (12 to 12.9)	8.05 (7.24 to 8.83)	8.65 (7.75 to 9.51)	9.1 (8.2 to 9.94)	0.27 (0.23 to 0.32)	0.27 (0.23 to 0.33)	0.27 (0.23 to 0.32)
Zambia	10 (9.67 to 10.5)	10.7 (9.87 to 11.4)	11.8 (10.6 to 12.6)	7.41 (6.61 to 8.18)	7.89 (7.01 to 8.74)	8.74 (7.61 to 9.78)	0.26 (0.22 to 0.32)	0.26 (0.23 to 0.29)	0.26 (0.22 to 0.28)
Southern Sub-Saharan Africa	13.9 (13.7 to 14.2)	11.8 (11.6 to 12)	13.2 (12.9 to 13.5)	9.89 (8.83 to 10.9)	8.36 (7.51 to 9.21)	9.37 (8.4 to 10.3)	0.29 (0.23 to 0.36)	0.29 (0.23 to 0.35)	0.29 (0.24 to 0.35)
Botswana	11.4 (10 to 12.3)	10.8 (9.12 to 13.9)	11.7 (10.2 to 12.9)	8.3 (7.04 to 9.41)	7.83 (6.35 to 10)	8.37 (7.04 to 9.57)	0.27 (0.23 to 0.3)	0.27 (0.28 to 0.3)	0.29 (0.26 to 0.31)
Eswatini	11.4 (10.7 to 12.2)	9.37 (8.23 to 10.7)	11.4 (9.78 to 13.8)	8.26 (7.36 to 9.18)	6.72 (5.69 to 7.83)	8.19 (6.76 to 9.9)	0.28 (0.25 to 0.31)	0.28 (0.27 to 0.31)	0.28 (0.28 to 0.31)
Lesotho	11.9 (11.3 to 12.2)	9.6 (8.69 to 10.7)	9.92 (8.63 to 11.6)	8.59 (7.7 to 9.43)	6.92 (5.96 to 7.94)	7.11 (5.93 to 8.47)	0.28 (0.23 to 0.32)	0.28 (0.26 to 0.31)	0.28 (0.27 to 0.31)
Namibia	11.4 (10.9 to 11.8)	11.1 (10.1 to 12.2)	12.5 (11.3 to 13.7)	8.18 (7.29 to 9.06)	7.95 (6.95 to 9.11)	9.04 (7.81 to 10.4)	0.28 (0.23 to 0.33)	0.28 (0.25 to 0.31)	0.27 (0.24 to 0.31)
South Africa	14.7 (14.4 to 15)	12.2 (12.1 to 12.3)	13.8 (13.6 to 14)	10.4 (9.22 to 11.4)	8.6 (7.71 to 9.45)	9.73 (8.69 to 10.7)	0.29 (0.24 to 0.36)	0.29 (0.23 to 0.36)	0.29 (0.24 to 0.36)
Zimbabwe	11.8 (11.3 to 12.3)	10.7 (9.71 to 11.7)	11 (9.81 to 12.2)	8.61 (7.72 to 9.52)	7.71 (6.68 to 8.74)	7.97 (6.77 to 9.18)	0.27 (0.23 to 0.32)	0.28 (0.25 to 0.31)	0.28 (0.25 to 0.31)
Western Sub-Saharan Africa	10.7 (10.3 to 11.1)	11.1 (10.7 to 11.7)	12 (11.5 to 12.6)	7.69 (6.88 to 8.46)	8.06 (7.19 to 8.88)	8.77 (7.81 to 9.68)	0.28 (0.24 to 0.33)	0.27 (0.24 to 0.33)	0.27 (0.23 to 0.32)
Benin	10.8 (10.5 to 11.1)	11.4 (10.8 to 12)	12.1 (11.3 to 12.6)	7.82 (7 to 8.57)	8.37 (7.51 to 9.24)	8.82 (7.78 to 9.73)	0.28 (0.23 to 0.34)	0.27 (0.23 to 0.31)	0.27 (0.23 to 0.31)
Burkina Faso	10.5 (10.1 to 10.9)	10.9 (10.6 to 11.3)	11.8 (11.2 to 12.1)	7.59 (6.75 to 8.38)	8.05 (7.24 to 8.83)	8.67 (7.8 to 9.47)	0.28 (0.23 to 0.33)	0.26 (0.22 to 0.32)	0.26 (0.22 to 0.31)
Cabo Verde	14.7 (14.3 to 15.1)	15.4 (14.7 to 15.8)	14 (13.5 to 14.6)	10.9 (9.79 to 11.8)	11.2 (9.99 to 12.4)	10.3 (9.21 to 11.3)	0.26 (0.22 to 0.31)	0.27 (0.22 to 0.32)	0.27 (0.23 to 0.32)
Cameroon	10.9 (10.5 to 11.2)	10.9 (10.3 to 11.5)	12.1 (10.9 to 12.7)	7.84 (7.01 to 8.64)	7.78 (6.91 to 8.7)	8.76 (7.58 to 9.78)	0.28 (0.23 to 0.34)	0.28 (0.25 to 0.33)	0.27 (0.23 to 0.31)
Chad	10.5 (10.1 to 10.7)	10.5 (10.1 to 10.9)	11.3 (10.5 to 11.8)	7.63 (6.88 to 8.38)	7.55 (6.73 to 8.33)	8.18 (7.23 to 9.05)	0.27 (0.22 to 0.32)	0.28 (0.23 to 0.33)	0.28 (0.23 to 0.31)
Côte d'Ivoire	11.1 (10.7 to 11.5)	11.2 (10.5 to 11.8)	12.3 (11.5 to 12.7)	7.78 (6.81 to 8.67)	8.13 (7.22 to 9.03)	8.94 (7.9 to 9.84)	0.3 (0.25 to 0.36)	0.27 (0.23 to 0.31)	0.27 (0.23 to 0.31)
Gambia	11.3 (10.6 to 11.8)	11.4 (10.8 to 12.1)	11.6 (10.7 to 12.4)	8.21 (7.25 to 9.11)	8.25 (7.35 to 9.2)	8.41 (7.42 to 9.33)	0.27 (0.23 to 0.32)	0.28 (0.24 to 0.32)	0.28 (0.25 to 0.31)
Ghana	10.9 (10.3 to 11.4)	11.5 (10.9 to 12)	12.3 (11.6 to 12.6)	8.03 (7.19 to 8.86)	8.42 (7.54 to 9.3)	9.1 (8.09 to 9.99)	0.26 (0.22 to 0.3)	0.27 (0.23 to 0.31)	0.26 (0.21 to 0.3)
Guinea	10 (9.72 to 10.3)	10.6 (10.2 to 11.1)	11.2 (10.4 to 12.1)	7.35 (6.6 to 8.07)	7.81 (7.04 to 8.56)	8.25 (7.23 to 9.27)	0.27 (0.22 to 0.32)	0.27 (0.23 to 0.31)	0.27 (0.23 to 0.3)
Guinea-Bissau	9.7 (9.09 to 10.3)	10.2 (9.57 to 10.7)	10.9 (9.71 to 11.9)	7.08 (6.25 to 7.9)	7.44 (6.63 to 8.18)	7.96 (6.89 to 8.99)	0.27 (0.23 to 0.31)	0.27 (0.24 to 0.31)	0.27 (0.24 to 0.29)
Liberia	10.1 (9.78 to 10.4)	10.8 (10.3 to 11.3)	11.8 (10.8 to 12.5)	7.15 (6.36 to 7.92)	7.66 (6.77 to 8.48)	8.35 (7.23 to 9.43)	0.29 (0.24 to 0.35)	0.29 (0.25 to 0.35)	0.29 (0.24 to 0.33)
Mali	9.76 (9.53 to 10)	10.6 (10.3 to 10.9)	11.3 (10.6 to 11.8)	7.08 (6.34 to 7.78)	7.7 (6.94 to 8.46)	8.29 (7.37 to 9.14)	0.27 (0.23 to 0.34)	0.27 (0.22 to 0.32)	0.27 (0.22 to 0.31)
Mauritania	10.2 (9.81 to 10.7)	11.4 (10.9 to 11.8)	12.3 (11.6 to 12.4)	7.56 (6.78 to 8.3)	8.47 (7.6 to 9.28)	9.08 (8.14 to 9.89)	0.26 (0.22 to 0.31)	0.26 (0.21 to 0.3)	0.26 (0.21 to 0.3)
Niger	10 (9.81 to 10.3)	10.7 (10.4 to 10.9)	11.5 (10.8 to 11.9)	7.42 (6.7 to 8.08)	7.93 (7.19 to 8.65)	8.51 (7.56 to 9.33)	0.26 (0.22 to 0.32)	0.26 (0.21 to 0.31)	0.26 (0.22 to 0.3)
Nigeria	10.7 (10.1 to 11.6)	11.2 (10.3 to 12.6)	12.2 (11.3 to 13.6)	7.7 (6.79 to 8.68)	8.08 (7.06 to 9.23)	8.86 (7.71 to 10.1)	0.28 (0.25 to 0.32)	0.28 (0.27 to 0.32)	0.27 (0.25 to 0.32)

Supplementary Table 8: Life expectancy, Health-adjusted life expectancy, and proportion of years spent in ill health in all reporting locations, in 1990, 2005, 2019, Females									
	LE-70			HALE-70			PYIH-70		
location	1990	2005	2019	1990	2005	2019	1990	2005	2019
Sao Tome and Principe	11.6 (11.3 to 11.8)	11.5 (11.2 to 11.8)	11.8 (11.5 to 12.4)	8.46 (7.61 to 9.25)	8.36 (7.55 to 9.15)	8.55 (7.7 to 9.44)	0.27 (0.22 to 0.33)	0.27 (0.22 to 0.32)	0.28 (0.24 to 0.33)
Senegal	11 (10.7 to 11.3)	11.7 (11.4 to 12)	12.3 (11.4 to 12.7)	8.05 (7.16 to 8.83)	8.5 (7.57 to 9.33)	8.97 (7.95 to 9.86)	0.27 (0.22 to 0.33)	0.28 (0.22 to 0.33)	0.27 (0.22 to 0.3)
Sierra Leone	10.6 (10.2 to 11.2)	10.4 (10 to 10.8)	11.4 (10.5 to 11.9)	7.76 (6.98 to 8.59)	7.6 (6.83 to 8.32)	8.34 (7.3 to 9.27)	0.27 (0.23 to 0.31)	0.27 (0.23 to 0.32)	0.27 (0.22 to 0.31)
Togo	10.9 (10.5 to 11.2)	11.5 (10.8 to 12.1)	12.2 (11.3 to 12.7)	7.98 (7.17 to 8.74)	8.43 (7.53 to 9.32)	9 (7.9 to 9.95)	0.27 (0.22 to 0.32)	0.27 (0.23 to 0.3)	0.26 (0.22 to 0.3)

Supplementary Table 9. Data coverage index for all risk factors for 204 reporting locations, 1990–2005, 2005–2019 and in total by all ages and for the 70+ age						
	1990-2005		2005-2019		1990-2019	
	All ages	70+	All ages	70+	All ages	70+
Armenia	58.54%	46.34%	63.41%	56.10%	65.85%	56.10%
Azerbaijan	56.10%	48.78%	60.98%	53.66%	60.98%	56.10%
Georgia	56.10%	51.22%	63.41%	53.66%	63.41%	53.66%
Kazakhstan	63.41%	58.54%	60.98%	53.66%	68.29%	58.54%
Kyrgyzstan	51.22%	48.78%	63.41%	53.66%	63.41%	53.66%
Mongolia	53.66%	46.34%	58.54%	51.22%	60.98%	51.22%
Tajikistan	48.78%	39.02%	56.10%	48.78%	58.54%	48.78%
Turkmenistan	41.46%	24.39%	36.59%	36.59%	51.22%	36.59%
Uzbekistan	53.66%	46.34%	56.10%	41.46%	58.54%	53.66%
Albania	56.10%	46.34%	63.41%	53.66%	68.29%	53.66%
Bosnia and Herzegovina	53.66%	53.66%	58.54%	53.66%	58.54%	56.10%
Bulgaria	53.66%	51.22%	60.98%	56.10%	63.41%	58.54%
Croatia	60.98%	56.10%	58.54%	53.66%	70.73%	60.98%
Czechia	63.41%	58.54%	56.10%	51.22%	65.85%	58.54%
Hungary	63.41%	58.54%	63.41%	58.54%	70.73%	60.98%
Montenegro	41.46%	36.59%	58.54%	53.66%	60.98%	53.66%
North Macedonia	48.78%	41.46%	63.41%	51.22%	65.85%	51.22%
Poland	58.54%	51.22%	63.41%	56.10%	65.85%	56.10%
Romania	60.98%	51.22%	65.85%	58.54%	68.29%	58.54%
Serbia	53.66%	41.46%	70.73%	60.98%	75.61%	60.98%
Slovakia	58.54%	58.54%	63.41%	56.10%	68.29%	60.98%
Slovenia	60.98%	56.10%	63.41%	56.10%	68.29%	60.98%
Belarus	51.22%	48.78%	60.98%	53.66%	60.98%	53.66%
Estonia	58.54%	53.66%	63.41%	58.54%	70.73%	63.41%
Latvia	53.66%	53.66%	65.85%	56.10%	70.73%	60.98%
Lithuania	46.34%	39.02%	63.41%	58.54%	65.85%	58.54%
Republic of Moldova	56.10%	46.34%	60.98%	53.66%	60.98%	53.66%
Russian Federation	68.29%	60.98%	65.85%	58.54%	75.61%	60.98%
Ukraine	60.98%	58.54%	65.85%	58.54%	68.29%	60.98%
Australia	78.05%	60.98%	65.85%	53.66%	78.05%	60.98%
New Zealand	63.41%	51.22%	63.41%	53.66%	70.73%	58.54%
Brunei Darussalam	43.90%	41.46%	43.90%	36.59%	46.34%	41.46%
Japan	70.73%	58.54%	58.54%	53.66%	70.73%	58.54%
Republic of Korea	63.41%	56.10%	65.85%	56.10%	65.85%	58.54%
Singapore	46.34%	43.90%	46.34%	43.90%	48.78%	43.90%
Canada	75.61%	51.22%	65.85%	51.22%	75.61%	53.66%
Greenland	9.76%	0.00%	17.07%	14.63%	21.95%	14.63%
United States of America	82.93%	73.17%	73.17%	65.85%	82.93%	75.61%
Argentina	75.61%	56.10%	65.85%	58.54%	78.05%	60.98%
Chile	65.85%	60.98%	56.10%	51.22%	70.73%	63.41%
Uruguay	56.10%	51.22%	60.98%	53.66%	63.41%	56.10%
Andorra	14.63%	12.20%	9.76%	4.88%	17.07%	12.20%
Austria	65.85%	58.54%	65.85%	58.54%	70.73%	63.41%

Supplementary Table 9. Data coverage index for all risk factors for 204 reporting locations, 1990–2005, 2005–2019 and in total by all ages and for the 70+ age group						
	1990-2005		2005-2019		1990-2019	
	All ages	70+	All ages	70+	All ages	70+
Belgium	56.10%	51.22%	63.41%	56.10%	65.85%	56.10%
Cyprus	46.34%	39.02%	58.54%	53.66%	60.98%	53.66%
Denmark	70.73%	56.10%	68.29%	56.10%	75.61%	58.54%
Finland	70.73%	56.10%	68.29%	56.10%	73.17%	58.54%
France	73.17%	56.10%	65.85%	56.10%	75.61%	60.98%
Germany	65.85%	56.10%	70.73%	60.98%	73.17%	63.41%
Greece	65.85%	58.54%	68.29%	60.98%	70.73%	63.41%
Iceland	46.34%	43.90%	53.66%	43.90%	56.10%	48.78%
Ireland	63.41%	58.54%	63.41%	58.54%	70.73%	63.41%
Israel	63.41%	51.22%	53.66%	51.22%	65.85%	53.66%
Italy	73.17%	65.85%	65.85%	56.10%	73.17%	65.85%
Luxembourg	48.78%	43.90%	58.54%	53.66%	60.98%	53.66%
Malta	41.46%	39.02%	58.54%	53.66%	58.54%	53.66%
Monaco	0.00%	0.00%	14.63%	0.00%	14.63%	0.00%
Netherlands	65.85%	53.66%	70.73%	58.54%	73.17%	58.54%
Norway	68.29%	53.66%	63.41%	53.66%	70.73%	58.54%
Portugal	58.54%	58.54%	68.29%	60.98%	68.29%	60.98%
San Marino	12.20%	0.00%	14.63%	2.44%	14.63%	2.44%
Spain	78.05%	73.17%	70.73%	63.41%	78.05%	73.17%
Sweden	65.85%	60.98%	68.29%	60.98%	70.73%	65.85%
Switzerland	58.54%	51.22%	58.54%	48.78%	63.41%	51.22%
United Kingdom	65.85%	48.78%	58.54%	51.22%	68.29%	53.66%
Bolivia (Plurinational State of)	60.98%	51.22%	58.54%	46.34%	63.41%	51.22%
Ecuador	60.98%	56.10%	63.41%	51.22%	68.29%	56.10%
Peru	65.85%	56.10%	68.29%	60.98%	70.73%	60.98%
Antigua and Barbuda	48.78%	41.46%	41.46%	36.59%	48.78%	41.46%
Bahamas	46.34%	41.46%	41.46%	36.59%	46.34%	41.46%
Barbados	48.78%	43.90%	56.10%	51.22%	56.10%	51.22%
Belize	53.66%	48.78%	58.54%	58.54%	60.98%	58.54%
Bermuda	36.59%	34.15%	41.46%	39.02%	41.46%	39.02%
Cuba	56.10%	43.90%	53.66%	43.90%	60.98%	46.34%
Dominica	48.78%	41.46%	29.27%	21.95%	48.78%	41.46%
Dominican Republic	58.54%	56.10%	60.98%	56.10%	63.41%	58.54%
Grenada	46.34%	41.46%	46.34%	29.27%	46.34%	41.46%
Guyana	53.66%	46.34%	56.10%	36.59%	56.10%	48.78%
Haiti	58.54%	46.34%	46.34%	39.02%	60.98%	51.22%
Jamaica	58.54%	46.34%	56.10%	51.22%	63.41%	51.22%
Puerto Rico	36.59%	34.15%	36.59%	29.27%	41.46%	36.59%
Saint Kitts and Nevis	34.15%	17.07%	21.95%	17.07%	34.15%	17.07%
Saint Lucia	51.22%	46.34%	53.66%	36.59%	53.66%	48.78%
Saint Vincent and the Grenadines	34.15%	29.27%	41.46%	26.83%	46.34%	31.71%
Suriname	48.78%	43.90%	51.22%	46.34%	51.22%	46.34%
Trinidad and Tobago	48.78%	48.78%	53.66%	46.34%	53.66%	51.22%

Supplementary Table 9. Data coverage index for all risk factors for 204 reporting locations, 1990–2005, 2005–2019 and in total by all ages and for the 70+ age group						
	1990-2005		2005-2019		1990-2019	
	All ages	70+	All ages	70+	All ages	70+
United States Virgin Islands	9.76%	9.76%	12.20%	9.76%	14.63%	12.20%
Colombia	68.29%	58.54%	70.73%	58.54%	70.73%	60.98%
Costa Rica	60.98%	56.10%	60.98%	56.10%	68.29%	60.98%
El Salvador	58.54%	51.22%	58.54%	51.22%	58.54%	53.66%
Guatemala	65.85%	56.10%	60.98%	48.78%	65.85%	56.10%
Honduras	58.54%	46.34%	63.41%	53.66%	65.85%	53.66%
Mexico	60.98%	60.98%	70.73%	58.54%	70.73%	63.41%
Nicaragua	60.98%	51.22%	58.54%	53.66%	60.98%	58.54%
Panama	58.54%	51.22%	56.10%	51.22%	60.98%	51.22%
Venezuela (Bolivarian Republic of)	65.85%	58.54%	53.66%	48.78%	68.29%	60.98%
Brazil	68.29%	60.98%	70.73%	60.98%	73.17%	63.41%
Paraguay	56.10%	51.22%	58.54%	48.78%	60.98%	53.66%
Afghanistan	29.27%	24.39%	63.41%	36.59%	63.41%	36.59%
Algeria	53.66%	48.78%	51.22%	39.02%	56.10%	51.22%
Bahrain	24.39%	21.95%	21.95%	14.63%	26.83%	21.95%
Egypt	68.29%	58.54%	70.73%	58.54%	73.17%	60.98%
Iran (Islamic Republic of)	51.22%	41.46%	58.54%	48.78%	60.98%	48.78%
Iraq	58.54%	48.78%	58.54%	51.22%	60.98%	53.66%
Jordan	58.54%	48.78%	60.98%	39.02%	63.41%	51.22%
Kuwait	46.34%	29.27%	48.78%	36.59%	51.22%	41.46%
Lebanon	53.66%	41.46%	48.78%	34.15%	58.54%	41.46%
Libya	39.02%	31.71%	48.78%	34.15%	53.66%	36.59%
Morocco	63.41%	56.10%	60.98%	48.78%	68.29%	58.54%
Oman	51.22%	48.78%	43.90%	39.02%	53.66%	48.78%
Palestine	53.66%	43.90%	58.54%	51.22%	58.54%	51.22%
Qatar	21.95%	17.07%	26.83%	19.51%	29.27%	19.51%
Saudi Arabia	58.54%	48.78%	58.54%	48.78%	63.41%	48.78%
Sudan	17.07%	9.76%	34.15%	12.20%	36.59%	12.20%
Syrian Arab Republic	53.66%	48.78%	53.66%	46.34%	53.66%	51.22%
Tunisia	56.10%	43.90%	58.54%	41.46%	63.41%	48.78%
Turkey	75.61%	60.98%	68.29%	60.98%	75.61%	63.41%
United Arab Emirates	63.41%	58.54%	51.22%	43.90%	65.85%	58.54%
Yemen	58.54%	46.34%	56.10%	51.22%	60.98%	51.22%
Bangladesh	68.29%	48.78%	65.85%	51.22%	70.73%	51.22%
Bhutan	12.20%	9.76%	36.59%	31.71%	39.02%	34.15%
India	75.61%	63.41%	80.49%	63.41%	80.49%	65.85%
Nepal	56.10%	51.22%	63.41%	51.22%	65.85%	53.66%
Pakistan	58.54%	53.66%	65.85%	56.10%	68.29%	58.54%
China	75.61%	65.85%	73.17%	58.54%	78.05%	68.29%
Democratic People's Republic of Korea	34.15%	29.27%	39.02%	34.15%	39.02%	34.15%
Taiwan (Province of China)	65.85%	51.22%	58.54%	48.78%	65.85%	51.22%
American Samoa	29.27%	14.63%	19.51%	14.63%	29.27%	14.63%
Cook Islands	7.32%	2.44%	19.51%	2.44%	19.51%	2.44%

Supplementary Table 9. Data coverage index for all risk factors for 204 reporting locations, 1990–2005, 2005–2019 and in total by all ages and for the 70+ age group						
	1990-2005		2005-2019		1990-2019	
	All ages	70+	All ages	70+	All ages	70+
Fiji	53.66%	39.02%	51.22%	29.27%	56.10%	39.02%
Guam	19.51%	7.32%	19.51%	7.32%	19.51%	7.32%
Kiribati	41.46%	34.15%	46.34%	39.02%	48.78%	39.02%
Marshall Islands	21.95%	14.63%	21.95%	17.07%	26.83%	17.07%
Micronesia (Federated States of)	12.20%	4.88%	21.95%	4.88%	26.83%	4.88%
Nauru	4.88%	0.00%	19.51%	2.44%	19.51%	2.44%
Niue	17.07%	2.44%	7.32%	4.88%	19.51%	4.88%
Northern Mariana Islands	17.07%	14.63%	17.07%	2.44%	17.07%	14.63%
Palau	17.07%	2.44%	14.63%	2.44%	17.07%	4.88%
Papua New Guinea	48.78%	41.46%	48.78%	26.83%	56.10%	43.90%
Samoa	56.10%	41.46%	46.34%	41.46%	56.10%	41.46%
Solomon Islands	39.02%	21.95%	51.22%	29.27%	53.66%	29.27%
Tokelau	0.00%	0.00%	4.88%	2.44%	4.88%	2.44%
Tonga	21.95%	17.07%	14.63%	7.32%	29.27%	19.51%
Tuvalu	14.63%	0.00%	19.51%	2.44%	19.51%	2.44%
Vanuatu	29.27%	24.39%	53.66%	46.34%	56.10%	46.34%
Cambodia	60.98%	48.78%	63.41%	51.22%	65.85%	51.22%
Indonesia	68.29%	58.54%	68.29%	58.54%	73.17%	60.98%
Lao People's Democratic Republic	46.34%	41.46%	56.10%	36.59%	58.54%	41.46%
Malaysia	68.29%	63.41%	58.54%	48.78%	73.17%	63.41%
Maldives	48.78%	39.02%	58.54%	48.78%	60.98%	48.78%
Mauritius	53.66%	51.22%	56.10%	48.78%	60.98%	53.66%
Myanmar	56.10%	51.22%	58.54%	36.59%	60.98%	51.22%
Philippines	63.41%	60.98%	73.17%	58.54%	75.61%	60.98%
Seychelles	29.27%	12.20%	34.15%	26.83%	34.15%	26.83%
Sri Lanka	60.98%	53.66%	56.10%	41.46%	65.85%	53.66%
Thailand	70.73%	60.98%	70.73%	60.98%	75.61%	65.85%
Timor-Leste	41.46%	31.71%	56.10%	43.90%	58.54%	43.90%
Viet Nam	63.41%	58.54%	73.17%	58.54%	73.17%	60.98%
Angola	53.66%	46.34%	51.22%	31.71%	56.10%	46.34%
Central African Republic	41.46%	34.15%	43.90%	36.59%	46.34%	36.59%
Congo	36.59%	34.15%	48.78%	31.71%	51.22%	36.59%
Democratic Republic of the Congo	34.15%	29.27%	56.10%	36.59%	58.54%	36.59%
Equatorial Guinea	12.20%	9.76%	9.76%	4.88%	17.07%	12.20%
Gabon	46.34%	43.90%	53.66%	34.15%	53.66%	43.90%
Burundi	41.46%	36.59%	56.10%	34.15%	58.54%	36.59%
Comoros	39.02%	39.02%	43.90%	36.59%	43.90%	41.46%
Djibouti	43.90%	29.27%	39.02%	36.59%	51.22%	36.59%
Eritrea	24.39%	14.63%	12.20%	9.76%	24.39%	17.07%
Ethiopia	60.98%	51.22%	63.41%	51.22%	65.85%	53.66%
Kenya	58.54%	39.02%	56.10%	39.02%	70.73%	41.46%
Madagascar	58.54%	48.78%	58.54%	46.34%	63.41%	48.78%
Malawi	60.98%	53.66%	58.54%	53.66%	60.98%	56.10%

Supplementary Table 9. Data coverage index for all risk factors for 204 reporting locations, 1990–2005, 2005–2019 and in total by all ages and for the 70+ age group						
	1990-2005		2005-2019		1990-2019	
	All ages	70+	All ages	70+	All ages	70+
Mozambique	60.98%	48.78%	60.98%	51.22%	65.85%	53.66%
Rwanda	53.66%	46.34%	58.54%	48.78%	58.54%	48.78%
Somalia	31.71%	26.83%	41.46%	34.15%	41.46%	34.15%
South Sudan	9.76%	9.76%	26.83%	12.20%	26.83%	12.20%
Uganda	63.41%	51.22%	68.29%	51.22%	68.29%	53.66%
United Republic of Tanzania	63.41%	51.22%	68.29%	51.22%	68.29%	53.66%
Zambia	60.98%	53.66%	58.54%	48.78%	63.41%	56.10%
Botswana	58.54%	46.34%	53.66%	53.66%	60.98%	56.10%
Eswatini	41.46%	39.02%	60.98%	36.59%	60.98%	41.46%
Lesotho	56.10%	43.90%	53.66%	46.34%	58.54%	46.34%
Namibia	60.98%	51.22%	58.54%	51.22%	63.41%	53.66%
South Africa	68.29%	63.41%	73.17%	63.41%	75.61%	68.29%
Zimbabwe	63.41%	43.90%	60.98%	51.22%	65.85%	53.66%
Benin	48.78%	36.59%	56.10%	51.22%	60.98%	51.22%
Burkina Faso	56.10%	48.78%	58.54%	48.78%	58.54%	51.22%
Cabo Verde	29.27%	24.39%	46.34%	29.27%	48.78%	29.27%
Cameroon	46.34%	39.02%	58.54%	48.78%	60.98%	53.66%
Chad	41.46%	41.46%	43.90%	41.46%	46.34%	43.90%
Côte d'Ivoire	48.78%	39.02%	65.85%	36.59%	68.29%	41.46%
Gambia	41.46%	34.15%	58.54%	48.78%	60.98%	48.78%
Ghana	63.41%	53.66%	65.85%	53.66%	68.29%	53.66%
Guinea	41.46%	36.59%	41.46%	34.15%	46.34%	39.02%
Guinea-Bissau	39.02%	34.15%	36.59%	31.71%	39.02%	34.15%
Liberia	34.15%	24.39%	60.98%	51.22%	63.41%	51.22%
Mali	58.54%	53.66%	58.54%	36.59%	60.98%	53.66%
Mauritania	36.59%	36.59%	51.22%	34.15%	51.22%	39.02%
Niger	51.22%	31.71%	53.66%	39.02%	53.66%	39.02%
Nigeria	56.10%	46.34%	65.85%	51.22%	68.29%	56.10%
Sao Tome and Principe	46.34%	41.46%	56.10%	46.34%	58.54%	46.34%
Senegal	56.10%	43.90%	60.98%	56.10%	65.85%	58.54%
Sierra Leone	48.78%	46.34%	58.54%	51.22%	58.54%	51.22%
Togo	39.02%	34.15%	60.98%	36.59%	60.98%	39.02%

Supplementary Table 10. Data coverage index for all reporting locations for 69 risk factors, 1990–2005, 2005–2019 and in total by all ages and for the 70+ age group						
	1990-2005		2005-2019		1990-2019	
	All ages	70+	All ages	70+	All ages	70+
Unsafe water source	68.97%	68.47%	76.35%	75.37%	83.25%	82.76%
Unsafe sanitation	68.97%	68.97%	61.08%	61.08%	75.86%	75.86%
No access to handwashing facility	8.37%	4.93%	32.51%	31.53%	34.98%	32.02%
Ambient particulate matter pollution	100.00%	0.00%	100.00%	0.00%	100.00%	0.00%
Household air pollution from solid fuels	100.00%	65.02%	100.00%	68.97%	100.00%	78.33%
Ambient ozone pollution	100.00%	1.48%	100.00%	0.99%	100.00%	1.48%
High temperature	90.15%	1.48%	90.15%	1.97%	90.15%	1.97%
Low temperature	90.15%	1.48%	90.15%	1.97%	90.15%	1.97%
Residential radon	25.12%	4.43%	19.70%	0.49%	33.50%	4.43%
Lead exposure	0.00%	0.00%	100.00%	0.00%	100.00%	0.00%
Occupational exposure to asbestos	100.00%	0.00%	100.00%	0.00%	100.00%	0.00%
Occupational exposure to arsenic	100.00%	71.92%	100.00%	69.95%	100.00%	77.83%
Occupational exposure to benzene	100.00%	71.92%	100.00%	69.95%	100.00%	77.83%
Occupational exposure to beryllium	100.00%	71.92%	100.00%	69.95%	100.00%	77.83%
Occupational exposure to cadmium	100.00%	71.92%	100.00%	69.95%	100.00%	77.83%
Occupational exposure to chromium	100.00%	71.92%	100.00%	69.95%	100.00%	77.83%
Occupational exposure to diesel engine exhaust	100.00%	71.92%	100.00%	69.95%	100.00%	77.83%
Occupational exposure to formaldehyde	100.00%	71.92%	100.00%	69.95%	100.00%	77.83%
Occupational exposure to nickel	100.00%	71.92%	100.00%	69.95%	100.00%	77.83%
Occupational exposure to polycyclic aromatic hydrocarbons	100.00%	71.92%	100.00%	69.95%	100.00%	77.83%
Occupational exposure to silica	100.00%	71.92%	100.00%	69.95%	100.00%	77.83%
Occupational exposure to sulfuric acid	100.00%	71.92%	100.00%	69.95%	100.00%	77.83%
Occupational exposure to trichloroethylene	100.00%	71.92%	100.00%	69.95%	100.00%	77.83%
Occupational asthmagens	100.00%	76.85%	100.00%	73.89%	100.00%	82.76%
Occupational particulate matter, gases, and fumes	100.00%	71.92%	100.00%	69.95%	100.00%	77.83%
Occupational noise	100.00%	71.92%	100.00%	69.95%	100.00%	77.83%
Occupational injuries	83.25%	75.37%	90.15%	71.92%	95.57%	81.28%
Occupational ergonomic factors	100.00%	71.92%	100.00%	69.95%	100.00%	77.83%
Non-exclusive breastfeeding	67.49%	66.50%	66.50%	65.52%	79.80%	79.80%
Discontinued breastfeeding	62.56%	61.08%	61.58%	60.59%	74.88%	74.38%
Child underweight	100.00%	0.00%	62.07%	0.00%	100.00%	0.00%
Child wasting	100.00%	0.00%	63.05%	0.00%	100.00%	0.00%
Child stunting	100.00%	0.00%	63.05%	0.00%	100.00%	0.00%
Short gestation	84.24%	0.00%	91.63%	0.00%	94.09%	0.00%
Low birth weight	84.24%	0.00%	91.63%	0.00%	94.09%	0.00%
Iron deficiency	64.04%	15.76%	41.87%	4.93%	73.40%	17.73%
Vitamin A deficiency	44.33%	4.93%	8.37%	0.00%	45.32%	4.93%
Zinc deficiency	85.71%	85.71%	85.71%	85.71%	85.71%	85.71%
Smoking	100.00%	100.00%	100.00%	100.00%	100.00%	100.00%
Chewing tobacco	23.65%	12.81%	90.64%	40.39%	90.64%	41.87%
Secondhand smoke	100.00%	100.00%	100.00%	100.00%	100.00%	100.00%
Alcohol use	100.00%	43.35%	100.00%	33.50%	100.00%	54.19%
Drug use	14.78%	2.96%	33.50%	1.48%	36.95%	3.94%
Diet low in fruits	100.00%	100.00%	100.00%	100.00%	100.00%	100.00%
Diet low in vegetables	100.00%	88.18%	100.00%	88.18%	100.00%	88.18%

Supplementary Table 10. Data coverage index for all reporting locations for 69 risk factors, 1990–2005, 2005–2019 and in total by all ages and for the 70+ age group

	1990-2005		2005-2019		1990-2019	
	All ages	70+	All ages	70+	All ages	70+
Diet low in legumes	83.25%	82.27%	82.27%	82.27%	83.25%	82.27%
Diet low in whole grains	89.66%	89.66%	92.12%	92.12%	92.12%	92.12%
Diet low in nuts and seeds	79.31%	79.31%	79.31%	79.31%	79.31%	79.31%
Diet low in milk	88.67%	88.67%	88.67%	88.67%	88.67%	88.67%
Diet high in red meat	89.16%	89.16%	89.16%	89.16%	89.16%	89.16%
Diet high in processed meat	28.57%	28.57%	34.48%	33.50%	34.48%	33.50%
Diet high in sugar-sweetened beverages	28.57%	28.57%	33.50%	32.51%	33.99%	33.00%
Diet low in fiber	100.00%	100.00%	89.66%	89.66%	100.00%	100.00%
Diet low in calcium	87.19%	87.19%	86.70%	86.70%	87.19%	87.19%
Diet low in seafood omega-3 fatty acids	88.18%	87.68%	87.68%	87.68%	88.18%	87.68%
Diet low in polyunsaturated fatty acids	100.00%	87.68%	87.19%	87.19%	100.00%	87.68%
Diet high in trans fatty acids	33.99%	33.99%	34.98%	34.48%	34.98%	34.48%
Diet high in sodium	100.00%	4.93%	100.00%	9.85%	100.00%	11.82%
Intimate partner violence	41.38%	22.17%	60.10%	31.53%	64.53%	36.45%
Childhood sexual abuse	26.60%	6.90%	22.66%	4.93%	33.99%	9.36%
Bullying victimization	22.17%	0.00%	56.65%	0.00%	59.61%	0.00%
Unsafe sex	16.75%	16.75%	46.80%	46.80%	47.29%	47.29%
Low physical activity	47.78%	18.23%	50.74%	25.12%	64.04%	29.06%
High fasting plasma glucose	37.44%	26.60%	49.75%	31.03%	59.61%	39.90%
High LDL cholesterol	39.41%	26.60%	53.20%	28.08%	67.98%	38.92%
High systolic blood pressure	50.25%	38.92%	68.97%	39.41%	79.31%	52.71%
High body-mass index	79.31%	52.22%	87.68%	46.31%	92.61%	63.05%
Low bone mineral density	20.69%	19.21%	10.84%	9.36%	22.66%	21.67%
Kidney dysfunction	100.00%	100.00%	100.00%	100.00%	100.00%	100.00%

Supplementary Table 11. Data coverage index for all non-fatal causes for 204 locations, 1990–2005, 2005–2019 and in total by all ages and for the 70+ age group

	1990-2005		2005-2019		1990-2019	
	All ages	70+	All ages	70+	All ages	70+
Armenia	7.58%	2.53%	7.58%	2.53%	8.59%	3.03%
Azerbaijan	7.07%	3.03%	6.57%	2.02%	8.08%	3.54%
Georgia	7.58%	4.55%	23.74%	20.71%	25.25%	22.22%
Kazakhstan	8.08%	4.55%	6.57%	3.54%	9.09%	5.56%
Kyrgyzstan	7.58%	3.03%	10.61%	7.07%	12.12%	8.08%
Mongolia	9.60%	5.05%	9.09%	5.05%	10.61%	6.06%
Tajikistan	6.06%	2.53%	6.57%	2.02%	7.07%	3.03%
Turkmenistan	5.05%	2.53%	5.56%	1.52%	5.56%	2.53%
Uzbekistan	7.07%	3.03%	6.06%	1.52%	7.07%	3.03%
Albania	6.06%	2.02%	10.61%	5.56%	11.62%	6.57%
Bosnia and Herzegovina	10.61%	5.56%	9.60%	4.55%	11.62%	6.57%
Bulgaria	24.75%	20.20%	22.73%	20.20%	26.77%	21.72%
Croatia	39.90%	36.87%	39.39%	36.36%	39.90%	36.87%
Czechia	26.26%	22.22%	39.90%	37.37%	42.42%	38.89%
Hungary	24.24%	19.70%	22.22%	19.19%	25.76%	21.21%
Montenegro	3.03%	0.51%	6.06%	2.02%	6.06%	2.02%
North Macedonia	5.56%	1.52%	6.06%	2.02%	6.57%	2.02%
Poland	34.34%	30.30%	33.84%	31.82%	35.35%	32.32%
Romania	10.61%	4.04%	34.34%	31.82%	38.89%	32.32%
Serbia	23.23%	18.69%	32.32%	29.80%	34.34%	31.31%
Slovakia	32.32%	29.80%	31.31%	29.80%	33.33%	30.81%
Slovenia	37.88%	35.86%	38.38%	36.87%	39.39%	37.37%
Belarus	20.71%	18.18%	22.22%	18.18%	23.23%	19.19%
Estonia	24.75%	21.21%	25.25%	21.72%	27.78%	24.24%
Latvia	32.32%	29.80%	32.83%	31.31%	33.84%	31.82%
Lithuania	34.85%	30.81%	33.84%	30.81%	35.35%	31.82%
Republic of Moldova	5.56%	3.03%	7.58%	4.04%	8.08%	4.55%
Russian Federation	11.11%	6.57%	11.11%	9.60%	15.15%	11.11%
Ukraine	17.68%	15.15%	16.67%	13.13%	18.69%	15.66%
Australia	39.90%	33.84%	35.86%	31.31%	41.41%	35.86%
New Zealand	30.30%	25.25%	30.30%	25.76%	33.33%	28.79%
Brunei Darussalam	5.56%	3.54%	20.71%	18.18%	21.21%	18.69%
Japan	26.26%	22.73%	26.26%	23.23%	28.79%	25.25%
Republic of Korea	33.84%	27.78%	34.85%	29.29%	38.38%	32.83%
Singapore	30.81%	24.24%	30.81%	27.27%	33.84%	28.28%
Canada	33.84%	29.80%	32.32%	29.29%	36.36%	32.83%
Greenland	15.66%	14.14%	18.18%	16.16%	18.18%	16.16%
United States of America	54.55%	46.46%	56.06%	49.49%	58.59%	50.51%
Argentina	30.81%	26.26%	29.80%	26.77%	33.33%	28.79%
Chile	44.44%	40.40%	43.94%	40.91%	47.98%	43.94%
Uruguay	27.27%	22.73%	24.24%	20.20%	28.79%	24.24%
Andorra	2.53%	1.01%	3.54%	1.52%	3.54%	1.52%
Austria	33.33%	31.31%	34.85%	31.31%	35.86%	32.32%

Supplementary Table 11. Data coverage index for all non-fatal causes for 204 locations, 1990–2005, 2005–2019 and in total by all ages and for the 70+ age group						
	1990-2005		2005-2019		1990-2019	
	All ages	70+	All ages	70+	All ages	70+
Belgium	42.93%	38.38%	41.41%	38.38%	44.95%	40.40%
Cyprus	31.82%	29.29%	34.85%	31.31%	35.35%	31.82%
Denmark	42.42%	38.38%	40.91%	37.88%	42.93%	39.39%
Finland	47.98%	42.93%	42.93%	40.40%	48.48%	43.43%
France	36.36%	30.30%	35.35%	29.29%	39.90%	33.84%
Germany	36.36%	31.31%	42.93%	39.39%	45.45%	41.41%
Greece	13.64%	9.09%	16.16%	9.09%	19.19%	12.12%
Iceland	28.28%	22.73%	33.84%	31.31%	36.87%	32.83%
Ireland	30.30%	22.73%	28.28%	22.73%	31.82%	25.76%
Israel	31.82%	25.25%	29.80%	23.74%	33.84%	27.27%
Italy	27.78%	24.24%	30.81%	28.28%	34.85%	31.82%
Luxembourg	15.66%	13.64%	16.67%	14.14%	17.68%	15.15%
Malta	32.32%	29.29%	38.38%	35.86%	39.39%	36.36%
Monaco	1.01%	0.00%	1.52%	0.00%	1.52%	0.00%
Netherlands	40.91%	33.84%	39.39%	33.84%	43.94%	37.37%
Norway	30.30%	24.75%	30.81%	24.75%	33.33%	27.78%
Portugal	27.78%	24.24%	43.94%	41.41%	46.97%	43.43%
San Marino	1.52%	0.00%	1.52%	0.00%	1.52%	0.00%
Spain	38.89%	33.84%	38.89%	31.82%	44.95%	36.36%
Sweden	40.91%	37.88%	43.43%	40.91%	43.94%	40.91%
Switzerland	42.42%	38.89%	40.91%	39.39%	43.94%	40.40%
United Kingdom	5.05%	2.53%	19.19%	18.18%	20.71%	18.69%
Bolivia (Plurinational State of)	11.11%	6.06%	8.59%	4.55%	11.62%	7.07%
Ecuador	36.87%	33.84%	37.37%	34.34%	39.39%	36.87%
Peru	29.29%	23.23%	30.81%	25.25%	33.33%	27.78%
Antigua and Barbuda	3.54%	1.52%	4.04%	2.02%	4.04%	2.02%
Bahamas	4.55%	2.02%	5.56%	3.54%	6.06%	3.54%
Barbados	6.57%	3.54%	7.07%	4.55%	8.59%	5.05%
Belize	6.06%	2.53%	8.59%	4.55%	9.09%	5.05%
Bermuda	7.07%	6.06%	10.10%	9.09%	10.10%	9.09%
Cuba	27.78%	23.74%	28.79%	25.76%	31.31%	27.27%
Dominica	3.54%	1.52%	5.56%	3.03%	5.56%	3.03%
Dominican Republic	12.12%	6.57%	9.60%	5.56%	13.13%	8.08%
Grenada	16.67%	14.65%	6.06%	3.54%	19.19%	16.67%
Guyana	7.07%	3.54%	8.59%	4.04%	8.59%	4.55%
Haiti	7.58%	3.03%	8.08%	4.55%	9.60%	5.56%
Jamaica	22.22%	18.18%	23.23%	20.20%	24.24%	20.20%
Puerto Rico	22.22%	18.69%	25.25%	23.23%	26.77%	23.23%
Saint Kitts and Nevis	1.52%	0.00%	3.54%	2.02%	3.54%	2.02%
Saint Lucia	4.04%	2.02%	6.57%	2.53%	6.57%	2.53%
Saint Vincent and the Grenadines	4.04%	2.02%	5.56%	3.03%	5.56%	3.03%
Suriname	6.06%	2.02%	7.07%	3.03%	7.58%	3.54%
Trinidad and Tobago	21.72%	18.18%	21.72%	19.19%	22.73%	19.19%

Supplementary Table 11. Data coverage index for all non-fatal causes for 204 locations, 1990–2005, 2005–2019 and in total by all ages and for the 70+ age group						
	1990-2005		2005-2019		1990-2019	
	All ages	70+	All ages	70+	All ages	70+
United States Virgin Islands	3.03%	0.51%	6.06%	3.54%	6.06%	3.54%
Colombia	31.82%	22.73%	31.31%	23.23%	33.84%	25.76%
Costa Rica	25.25%	20.71%	24.75%	21.21%	26.77%	21.72%
El Salvador	8.08%	3.54%	9.09%	5.56%	9.60%	5.56%
Guatemala	12.63%	7.58%	10.10%	6.57%	13.64%	9.09%
Honduras	9.09%	5.05%	9.09%	5.56%	10.61%	6.57%
Mexico	17.68%	13.64%	18.69%	14.65%	22.22%	18.69%
Nicaragua	8.08%	2.53%	9.09%	4.04%	10.10%	4.55%
Panama	24.24%	18.69%	25.25%	20.71%	26.77%	21.21%
Venezuela (Bolivarian Republic of)	11.62%	6.57%	11.62%	8.08%	14.65%	9.60%
Brazil	14.14%	9.60%	15.15%	9.60%	18.69%	13.13%
Paraguay	11.62%	6.57%	8.08%	4.55%	12.12%	7.58%
Afghanistan	5.05%	2.02%	7.07%	4.04%	7.07%	4.04%
Algeria	23.23%	18.18%	22.73%	19.19%	25.76%	20.71%
Bahrain	19.70%	16.67%	22.22%	19.19%	22.22%	19.19%
Egypt	31.82%	24.75%	28.79%	21.72%	35.86%	27.78%
Iran (Islamic Republic of)	34.85%	30.30%	34.85%	27.78%	41.41%	32.83%
Iraq	7.58%	1.52%	25.76%	21.21%	27.78%	21.72%
Jordan	22.22%	17.68%	29.29%	26.77%	31.31%	27.27%
Kuwait	26.77%	22.22%	23.74%	19.70%	27.78%	23.23%
Lebanon	19.19%	15.66%	25.25%	20.20%	27.78%	22.22%
Libya	20.71%	17.68%	21.72%	18.69%	22.73%	19.19%
Morocco	22.73%	18.18%	18.69%	15.15%	27.27%	23.23%
Oman	22.22%	18.18%	24.24%	19.70%	25.76%	20.20%
Palestine	4.55%	1.01%	21.21%	18.69%	21.72%	18.69%
Qatar	28.28%	25.76%	32.32%	28.79%	33.33%	28.79%
Saudi Arabia	27.78%	21.21%	26.26%	20.71%	31.31%	22.73%
Sudan	7.58%	4.04%	8.59%	5.05%	10.10%	6.06%
Syrian Arab Republic	6.57%	1.01%	6.57%	2.02%	7.58%	2.02%
Tunisia	24.24%	20.71%	23.74%	19.70%	27.27%	22.73%
Turkey	35.35%	26.77%	40.91%	34.85%	46.46%	38.38%
United Arab Emirates	11.11%	4.04%	6.57%	4.04%	12.12%	5.56%
Yemen	8.08%	2.53%	24.75%	19.70%	26.26%	20.20%
Bangladesh	20.20%	9.09%	12.63%	8.08%	22.22%	11.62%
Bhutan	6.06%	2.53%	6.57%	3.54%	7.58%	3.54%
India	44.44%	32.32%	54.04%	47.98%	57.58%	49.49%
Nepal	14.65%	8.59%	29.80%	25.76%	32.83%	27.27%
Pakistan	12.63%	7.07%	10.10%	5.05%	15.15%	8.59%
China	17.17%	11.62%	18.18%	14.65%	23.23%	18.18%
Democratic People's Republic of Korea	4.55%	1.52%	4.55%	1.52%	5.05%	2.02%
Taiwan (Province of China)	33.84%	28.28%	46.46%	42.93%	47.98%	43.94%
American Samoa	3.54%	1.01%	5.05%	2.53%	5.56%	2.53%
Cook Islands	3.03%	1.01%	3.03%	1.52%	3.54%	1.52%

Supplementary Table 11. Data coverage index for all non-fatal causes for 204 locations, 1990–2005, 2005–2019 and in total by all ages and for the 70+ age group

	1990-2005		2005-2019		1990-2019	
	All ages	70+	All ages	70+	All ages	70+
Fiji	22.73%	17.68%	22.73%	19.19%	24.75%	19.70%
Guam	3.03%	1.01%	4.55%	2.02%	4.55%	2.02%
Kiribati	5.05%	2.53%	4.55%	2.53%	5.05%	2.53%
Marshall Islands	5.05%	2.53%	5.05%	2.53%	6.06%	3.03%
Micronesia (Federated States of)	6.06%	3.54%	5.05%	3.03%	6.57%	4.04%
Nauru	2.02%	0.51%	2.02%	1.01%	2.53%	1.01%
Niue	1.52%	0.51%	2.02%	1.01%	2.02%	1.01%
Northern Mariana Islands	2.53%	0.51%	3.54%	1.52%	3.54%	1.52%
Palau	1.52%	0.51%	2.53%	1.01%	2.53%	1.01%
Papua New Guinea	7.58%	2.53%	7.58%	4.04%	9.09%	4.55%
Samoa	5.56%	3.03%	4.04%	2.53%	5.56%	3.03%
Solomon Islands	4.04%	2.02%	6.57%	3.54%	6.57%	3.54%
Tokelau	0.00%	0.00%	0.51%	0.51%	0.51%	0.51%
Tonga	8.59%	5.05%	6.57%	4.04%	9.60%	5.56%
Tuvalu	1.52%	0.51%	1.52%	0.51%	1.52%	0.51%
Vanuatu	6.06%	3.54%	7.07%	3.03%	7.58%	3.54%
Cambodia	12.63%	7.58%	8.59%	5.05%	13.13%	8.59%
Indonesia	9.09%	4.04%	9.09%	6.57%	11.62%	7.58%
Lao People's Democratic Republic	9.09%	5.56%	9.09%	5.05%	12.63%	8.08%
Malaysia	27.78%	23.23%	27.78%	23.23%	31.31%	26.26%
Maldives	6.06%	2.53%	7.58%	3.54%	8.59%	3.54%
Mauritius	19.70%	15.66%	17.68%	15.15%	21.21%	17.17%
Myanmar	8.59%	5.05%	7.07%	3.03%	9.60%	5.56%
Philippines	12.12%	6.06%	24.75%	21.21%	27.78%	22.73%
Seychelles	4.04%	1.52%	21.21%	18.69%	21.72%	18.69%
Sri Lanka	28.79%	22.73%	22.73%	16.67%	30.81%	24.24%
Thailand	37.37%	31.31%	28.79%	24.75%	39.90%	33.84%
Timor-Leste	6.06%	2.53%	8.08%	4.04%	8.59%	4.55%
Viet Nam	33.33%	25.76%	41.41%	36.87%	46.46%	40.91%
Angola	8.08%	2.02%	9.09%	4.55%	10.61%	4.55%
Central African Republic	9.09%	4.04%	9.09%	5.56%	11.11%	6.57%
Congo	10.61%	6.06%	9.60%	4.04%	14.14%	8.08%
Democratic Republic of the Congo	8.08%	3.54%	11.62%	7.58%	12.12%	8.08%
Equatorial Guinea	6.06%	1.52%	6.57%	3.03%	7.58%	3.03%
Gabon	7.07%	2.53%	10.61%	3.54%	12.63%	4.04%
Burundi	7.58%	3.54%	8.59%	3.54%	10.10%	5.05%
Comoros	8.59%	4.55%	6.57%	3.03%	9.60%	5.05%
Djibouti	5.05%	1.52%	5.05%	2.02%	5.56%	2.02%
Eritrea	5.05%	1.01%	7.58%	4.04%	8.59%	4.04%
Ethiopia	13.13%	8.08%	13.13%	7.58%	16.67%	11.62%
Kenya	25.76%	21.72%	13.64%	6.06%	27.78%	23.23%
Madagascar	8.59%	3.03%	8.59%	4.04%	11.11%	5.56%
Malawi	28.28%	23.23%	28.28%	24.24%	31.82%	27.27%

Supplementary Table 11. Data coverage index for all non-fatal causes for 204 locations, 1990–2005, 2005–2019 and in total by all ages and for the 70+ age group						
	1990-2005		2005-2019		1990-2019	
	All ages	70+	All ages	70+	All ages	70+
Mozambique	12.12%	6.06%	11.11%	4.55%	15.66%	8.59%
Rwanda	6.57%	2.02%	13.64%	7.07%	14.14%	7.07%
Somalia	4.04%	1.01%	5.05%	2.02%	5.56%	2.02%
South Sudan	6.57%	2.02%	9.60%	4.55%	10.10%	5.05%
Uganda	27.78%	21.21%	31.82%	25.25%	33.33%	25.76%
United Republic of Tanzania	15.66%	7.07%	18.69%	11.11%	22.22%	13.64%
Zambia	10.61%	5.56%	10.10%	5.56%	13.13%	8.59%
Botswana	28.28%	24.24%	29.80%	25.76%	31.31%	26.77%
Eswatini	8.59%	4.55%	7.58%	3.03%	11.11%	5.56%
Lesotho	6.57%	2.02%	7.58%	3.54%	8.59%	4.04%
Namibia	23.74%	18.18%	21.72%	17.68%	25.76%	20.20%
South Africa	26.26%	21.21%	28.79%	25.76%	31.82%	27.78%
Zimbabwe	27.78%	21.21%	24.75%	20.71%	29.80%	23.74%
Benin	10.10%	4.55%	12.12%	7.07%	13.13%	7.58%
Burkina Faso	12.63%	7.07%	13.64%	8.59%	16.16%	10.61%
Cabo Verde	6.06%	2.02%	6.57%	3.54%	8.59%	4.04%
Cameroon	23.23%	18.69%	22.73%	17.68%	26.26%	20.71%
Chad	10.61%	5.56%	8.59%	4.04%	12.12%	7.07%
Côte d'Ivoire	12.12%	6.06%	10.61%	5.05%	14.14%	8.08%
Gambia	24.75%	19.19%	20.71%	16.16%	27.27%	21.21%
Ghana	16.16%	7.07%	21.72%	12.63%	23.23%	13.13%
Guinea	23.23%	18.18%	21.21%	16.67%	25.76%	20.71%
Guinea-Bissau	6.06%	2.02%	9.60%	5.56%	9.60%	5.56%
Liberia	5.05%	1.52%	8.59%	3.54%	9.09%	3.54%
Mali	28.79%	20.20%	22.73%	16.67%	33.84%	24.24%
Mauritania	8.59%	4.55%	7.58%	5.05%	10.61%	7.58%
Niger	21.21%	15.15%	21.21%	15.66%	22.73%	16.16%
Nigeria	15.15%	8.59%	13.64%	9.09%	19.70%	13.13%
Sao Tome and Principe	5.05%	1.52%	7.07%	2.53%	7.58%	2.53%
Senegal	13.13%	7.07%	12.63%	6.06%	16.67%	9.60%
Sierra Leone	6.57%	2.02%	9.60%	5.05%	9.60%	5.05%
Togo	8.59%	3.54%	9.60%	5.05%	11.11%	6.06%

Supplementary Table 12. Data coverage index for all locations for 286 non-fatal causes, 1990–2005, 2005–2019 and in total by all ages and for the 70+ age group						
	1990-2005		2005-2019		1990-2019	
	All ages	70+	All ages	70+	All ages	70+
HIV/AIDS - Drug-susceptible Tuberculosis	0.00%	0.00%	0.00%	0.00%	0.00%	0.00%
HIV/AIDS - Multidrug-resistant Tuberculosis without extensive drug resistance	0.00%	0.00%	0.00%	0.00%	0.00%	0.00%
HIV/AIDS - Extensively drug-resistant Tuberculosis	26.89%	26.89%	63.45%	63.45%	63.87%	63.87%
HIV/AIDS resulting in other diseases	0.00%	0.00%	0.00%	0.00%	0.00%	0.00%
Syphilis	100.00%	16.39%	100.00%	21.43%	100.00%	21.85%
Chlamydial infection	40.76%	3.36%	100.00%	3.78%	100.00%	5.88%
Gonococcal infection	36.55%	1.68%	100.00%	2.52%	100.00%	2.94%
Trichomoniasis	12.61%	0.84%	100.00%	2.94%	100.00%	3.36%
Genital herpes	27.31%	13.03%	13.03%	3.36%	29.83%	15.13%
Other sexually transmitted infections	2.10%	1.26%	0.84%	0.42%	2.10%	1.26%
Latent tuberculosis infection	14.71%	1.26%	11.76%	0.84%	19.75%	1.68%
Drug-susceptible tuberculosis	0.00%	0.00%	0.00%	0.00%	0.00%	0.00%
Multidrug-resistant tuberculosis without extensive drug resistance	77.73%	77.73%	80.67%	80.67%	81.09%	81.09%
Extensively drug-resistant tuberculosis	0.84%	0.84%	34.45%	34.45%	35.29%	35.29%
Lower respiratory infections	51.68%	13.03%	71.85%	34.45%	73.53%	34.45%
Upper respiratory infections	22.27%	0.42%	22.69%	0.42%	29.41%	0.42%
Otitis media	7.56%	1.26%	100.00%	1.26%	100.00%	1.68%
Diarrheal diseases	100.00%	100.00%	100.00%	100.00%	100.00%	100.00%
Typhoid fever	5.88%	5.88%	6.72%	6.30%	8.82%	8.40%
Paratyphoid fever	8.40%	8.40%	7.98%	7.56%	11.34%	10.92%
Invasive Non-typhoidal Salmonella (iNTS)	5.04%	3.36%	7.14%	5.88%	8.40%	6.30%
Other intestinal infectious diseases	0.00%	0.00%	0.00%	0.00%	0.00%	0.00%
Malaria	100.00%	1.26%	100.00%	0.00%	100.00%	1.26%
Chagas disease	3.78%	2.52%	8.82%	8.82%	8.82%	8.82%
Visceral leishmaniasis	21.85%	21.43%	29.41%	29.41%	29.83%	29.83%
Cutaneous and mucocutaneous leishmaniasis	20.59%	19.75%	30.25%	30.25%	30.25%	30.25%
African trypanosomiasis	12.61%	10.50%	12.18%	10.92%	12.61%	11.76%
Schistosomiasis	65.13%	58.40%	64.29%	58.40%	68.91%	62.18%
Cysticercosis	4.62%	4.20%	2.94%	2.52%	5.46%	5.04%
Cystic echinococcosis	81.93%	17.23%	81.93%	18.91%	81.93%	22.69%
Lymphatic filariasis	100.00%	100.00%	100.00%	100.00%	100.00%	100.00%
Onchocerciasis	12.61%	1.68%	12.61%	0.84%	12.61%	1.68%
Trachoma	9.66%	9.66%	19.75%	19.33%	21.01%	21.01%
Dengue	28.99%	28.99%	100.00%	100.00%	100.00%	100.00%
Yellow fever	74.79%	74.79%	81.93%	81.93%	81.93%	81.93%
Rabies	0.00%	0.00%	0.00%	0.00%	0.00%	0.00%
Ascariasis	57.98%	57.14%	57.56%	56.30%	58.40%	57.14%
Trichuriasis	57.98%	57.14%	57.56%	56.30%	58.40%	57.14%
Hookworm disease	57.98%	57.14%	57.56%	56.30%	58.40%	57.14%
Food-borne trematodiases	100.00%	100.00%	100.00%	100.00%	100.00%	100.00%
Leprosy	52.10%	52.10%	72.27%	72.27%	72.27%	72.27%
Ebola	100.00%	100.00%	100.00%	100.00%	100.00%	100.00%
Zika virus	0.00%	0.00%	62.61%	62.61%	62.61%	62.61%
Guinea worm disease	7.98%	7.98%	7.98%	7.98%	7.98%	7.98%
Other neglected tropical diseases	0.00%	0.00%	0.00%	0.00%	0.00%	0.00%
Meningitis	100.00%	20.59%	100.00%	25.63%	100.00%	29.41%
Encephalitis	100.00%	11.76%	100.00%	16.81%	100.00%	18.49%
Diphtheria	100.00%	3.78%	100.00%	2.10%	100.00%	4.62%
Whooping cough	100.00%	0.00%	100.00%	0.00%	100.00%	0.00%
Tetanus	100.00%	5.46%	100.00%	2.94%	100.00%	5.88%
Measles	100.00%	0.00%	100.00%	0.00%	100.00%	0.00%

Supplementary Table 12. Data coverage index for all locations for 286 non-fatal causes, 1990–2005, 2005–2019 and in total by all ages and for the 70+ age group						
	1990-2005		2005-2019		1990-2019	
	All ages	70+	All ages	70+	All ages	70+
Varicella and herpes zoster	100.00%	100.00%	100.00%	100.00%	100.00%	100.00%
Acute hepatitis A	39.08%	21.85%	19.33%	9.66%	43.28%	25.63%
Acute hepatitis B	34.45%	17.65%	26.89%	8.82%	44.54%	23.11%
Acute hepatitis C	35.71%	20.17%	22.27%	17.65%	43.70%	28.99%
Acute hepatitis E	15.97%	11.34%	2.94%	2.52%	17.23%	12.18%
Other unspecified infectious diseases	0.00%	0.00%	0.00%	0.00%	0.00%	0.00%
Maternal hemorrhage	21.85%	0.00%	28.99%	0.00%	34.45%	0.00%
Maternal sepsis and other maternal infections	16.81%	0.00%	24.37%	0.00%	28.57%	0.00%
Maternal hypertensive disorders	28.15%	0.00%	38.66%	0.00%	42.86%	0.00%
Maternal obstructed labor and uterine rupture	17.65%	0.00%	22.27%	0.42%	26.89%	0.42%
Maternal abortion and miscarriage	12.61%	0.00%	21.01%	0.00%	21.43%	0.00%
Ectopic pregnancy	9.24%	0.00%	18.07%	0.00%	18.49%	0.00%
Other maternal disorders	0.00%	0.00%	0.00%	0.00%	0.00%	0.00%
Neonatal preterm birth	50.84%	0.00%	61.76%	0.00%	65.55%	0.00%
Neonatal encephalopathy due to birth asphyxia and trauma	18.49%	0.00%	19.33%	0.00%	22.27%	0.00%
Neonatal sepsis and other neonatal infections	13.87%	0.00%	18.49%	0.00%	19.75%	0.00%
Hemolytic disease and other neonatal jaundice	33.19%	0.00%	100.00%	0.00%	100.00%	0.00%
Other neonatal disorders	0.00%	0.00%	0.00%	0.00%	0.00%	0.00%
Protein-energy malnutrition	59.24%	0.00%	56.30%	0.00%	64.29%	0.00%
Iodine deficiency	30.67%	11.34%	3.36%	0.84%	30.67%	11.34%
Vitamin A deficiency	37.39%	4.20%	7.14%	0.00%	38.66%	4.20%
Dietary iron deficiency	100.00%	0.00%	100.00%	0.00%	100.00%	0.00%
Other nutritional deficiencies	0.00%	0.00%	0.00%	0.00%	0.00%	0.00%
Lip and oral cavity cancer	100.00%	100.00%	100.00%	100.00%	100.00%	100.00%
Nasopharynx cancer	100.00%	100.00%	100.00%	100.00%	100.00%	100.00%
Other pharynx cancer	100.00%	100.00%	100.00%	100.00%	100.00%	100.00%
Esophageal cancer	100.00%	100.00%	100.00%	100.00%	100.00%	100.00%
Stomach cancer	100.00%	100.00%	100.00%	100.00%	100.00%	100.00%
Colon and rectum cancer	100.00%	100.00%	100.00%	100.00%	100.00%	100.00%
Liver cancer due to hepatitis B	23.95%	22.69%	34.03%	33.19%	41.18%	39.50%
Liver cancer due to hepatitis C	0.00%	0.00%	0.00%	0.00%	0.00%	0.00%
Liver cancer due to alcohol use	7.14%	6.72%	22.69%	22.69%	25.21%	24.79%
Liver cancer due to NASH	7.14%	6.72%	23.53%	23.53%	25.21%	24.79%
Hepatoblastoma	100.00%	0.00%	100.00%	0.00%	100.00%	0.00%
Liver cancer due to other causes (internal)	5.88%	5.88%	21.43%	21.43%	22.69%	22.69%
Gallbladder and biliary tract cancer	100.00%	100.00%	100.00%	100.00%	100.00%	100.00%
Pancreatic cancer	100.00%	100.00%	100.00%	100.00%	100.00%	100.00%
Larynx cancer	100.00%	100.00%	100.00%	100.00%	100.00%	100.00%
Tracheal, bronchus, and lung cancer	100.00%	100.00%	100.00%	100.00%	100.00%	100.00%
Malignant skin melanoma	100.00%	100.00%	100.00%	100.00%	100.00%	100.00%
Non-melanoma skin cancer (squamous-cell carcinoma)	31.51%	31.51%	26.05%	26.05%	33.61%	33.61%
Non-melanoma skin cancer (basal-cell carcinoma)	29.41%	29.41%	23.53%	23.53%	31.51%	31.51%
Soft tissue and other extraosseous sarcomas	100.00%	100.00%	100.00%	100.00%	100.00%	100.00%
Malignant neoplasm of bone and articular cartilage	100.00%	100.00%	100.00%	100.00%	100.00%	100.00%
Breast cancer	100.00%	100.00%	100.00%	100.00%	100.00%	100.00%
Cervical cancer	100.00%	100.00%	100.00%	100.00%	100.00%	100.00%
Uterine cancer	100.00%	100.00%	100.00%	100.00%	100.00%	100.00%
Ovarian cancer	100.00%	100.00%	100.00%	100.00%	100.00%	100.00%
Prostate cancer	100.00%	100.00%	100.00%	100.00%	100.00%	100.00%
Testicular cancer	100.00%	100.00%	100.00%	100.00%	100.00%	100.00%
Kidney cancer	100.00%	100.00%	100.00%	100.00%	100.00%	100.00%

Supplementary Table 12. Data coverage index for all locations for 286 non-fatal causes, 1990–2005, 2005–2019 and in total by all ages and for the 70+ age group						
	1990-2005		2005-2019		1990-2019	
	All ages	70+	All ages	70+	All ages	70+
Bladder cancer	100.00%	100.00%	100.00%	100.00%	100.00%	100.00%
Brain and central nervous system cancer	100.00%	100.00%	100.00%	100.00%	100.00%	100.00%
Retinoblastoma	100.00%	0.00%	100.00%	0.00%	100.00%	0.00%
Other eye cancers	100.00%	100.00%	100.00%	100.00%	100.00%	100.00%
Neuroblastoma and other peripheral nervous cell tumors	100.00%	100.00%	100.00%	100.00%	100.00%	100.00%
Thyroid cancer	100.00%	100.00%	100.00%	100.00%	100.00%	100.00%
Mesothelioma	100.00%	100.00%	100.00%	100.00%	100.00%	100.00%
Hodgkin lymphoma	100.00%	100.00%	100.00%	100.00%	100.00%	100.00%
Burkitt lymphoma	100.00%	100.00%	100.00%	100.00%	100.00%	100.00%
Other non-Hodgkin lymphoma	100.00%	100.00%	100.00%	100.00%	100.00%	100.00%
Multiple myeloma	100.00%	100.00%	100.00%	100.00%	100.00%	100.00%
Acute lymphoid leukemia	100.00%	100.00%	100.00%	100.00%	100.00%	100.00%
Chronic lymphoid leukemia	2.94%	2.94%	100.00%	100.00%	100.00%	100.00%
Acute myeloid leukemia	100.00%	100.00%	100.00%	100.00%	100.00%	100.00%
Chronic myeloid leukemia	100.00%	100.00%	100.00%	100.00%	100.00%	100.00%
Other leukemia	100.00%	100.00%	100.00%	100.00%	100.00%	100.00%
Other malignant neoplasms (internal)	100.00%	100.00%	100.00%	100.00%	100.00%	100.00%
Myelodysplastic, myeloproliferative, and other hematopoietic neoplasms	8.82%	8.82%	13.87%	13.87%	14.29%	14.29%
Benign and in situ intestinal neoplasms	9.24%	9.24%	14.71%	14.71%	15.13%	15.13%
Benign and in situ cervical and uterine neoplasms	8.82%	8.82%	13.87%	13.87%	14.29%	14.29%
Other benign and in situ neoplasms	9.24%	9.24%	14.71%	14.71%	15.13%	15.13%
Rheumatic heart disease	13.45%	7.14%	15.13%	10.08%	20.17%	10.92%
Ischemic heart disease	97.48%	97.48%	97.48%	97.48%	97.48%	97.48%
Ischemic stroke	22.27%	21.85%	23.53%	22.69%	28.99%	28.15%
Intracerebral hemorrhage	21.85%	21.43%	23.11%	22.69%	28.99%	28.15%
Subarachnoid hemorrhage	17.65%	17.23%	18.91%	18.49%	23.95%	23.11%
Hypertensive heart disease	0.00%	0.00%	0.00%	0.00%	0.00%	0.00%
Non-rheumatic calcific aortic valve disease	8.40%	8.40%	11.76%	11.76%	12.18%	12.18%
Non-rheumatic degenerative mitral valve disease	6.72%	6.72%	10.50%	10.50%	10.50%	10.50%
Other non-rheumatic valve diseases	0.00%	0.00%	0.00%	0.00%	0.00%	0.00%
Myocarditis	7.56%	7.56%	12.61%	12.61%	12.61%	12.61%
Alcoholic cardiomyopathy	0.00%	0.00%	0.00%	0.00%	0.00%	0.00%
Other cardiomyopathy	0.00%	0.00%	0.00%	0.00%	0.00%	0.00%
Pulmonary Arterial Hypertension	7.98%	7.14%	9.66%	9.24%	10.08%	9.66%
Atrial fibrillation and flutter	13.03%	13.03%	15.97%	15.97%	18.49%	18.49%
Peripheral artery disease	3.78%	3.78%	2.94%	2.94%	5.88%	5.88%
Endocarditis	9.24%	9.24%	13.87%	13.87%	13.87%	13.87%
Other cardiovascular and circulatory diseases (internal)	0.42%	0.42%	0.42%	0.42%	0.42%	0.42%
Chronic obstructive pulmonary disease	12.18%	10.08%	17.65%	16.39%	21.85%	20.17%
Silicosis	7.98%	7.98%	13.03%	13.03%	13.03%	13.03%
Asbestosis	8.40%	8.40%	12.18%	12.18%	12.18%	12.18%
Coal workers pneumoconiosis	7.98%	7.98%	10.92%	10.92%	11.34%	11.34%
Other pneumoconiosis	8.40%	8.40%	13.03%	13.03%	13.45%	13.45%
Asthma	54.20%	30.25%	23.95%	16.39%	58.40%	36.97%
Interstitial lung disease and pulmonary sarcoidosis	13.87%	13.03%	15.97%	15.97%	18.91%	18.07%
Other chronic respiratory diseases	0.00%	0.00%	0.00%	0.00%	0.00%	0.00%
Cirrhosis and other chronic liver diseases due to hepatitis B	9.24%	9.24%	4.62%	4.62%	10.08%	10.08%
Cirrhosis and other chronic liver diseases due to hepatitis C	10.50%	10.08%	4.62%	4.62%	11.34%	10.92%
Cirrhosis and other chronic liver diseases due to alcohol use	5.88%	5.88%	5.04%	5.04%	7.14%	7.14%
Cirrhosis and other chronic liver diseases due to NAFLD	5.04%	5.04%	7.14%	5.88%	8.82%	7.56%
Cirrhosis and other chronic liver diseases due to other causes	4.20%	3.78%	4.20%	3.78%	5.04%	4.62%

Supplementary Table 12. Data coverage index for all locations for 286 non-fatal causes, 1990–2005, 2005–2019 and in total by all ages and for the 70+ age group						
	1990-2005		2005-2019		1990-2019	
	All ages	70+	All ages	70+	All ages	70+
Peptic ulcer disease	10.92%	9.66%	16.81%	15.97%	18.07%	16.39%
Gastritis and duodenitis	9.66%	9.66%	15.13%	15.13%	15.55%	15.55%
Gastroesophageal reflux disease	15.13%	14.71%	15.97%	14.71%	18.49%	17.23%
Appendicitis	9.24%	9.24%	15.13%	15.13%	15.55%	15.55%
Paralytic ileus and intestinal obstruction	9.24%	9.24%	15.13%	15.13%	15.55%	15.55%
Inguinal, femoral, and abdominal hernia	9.24%	9.24%	15.13%	15.13%	15.55%	15.55%
Inflammatory bowel disease	29.41%	28.57%	27.73%	24.79%	35.29%	34.03%
Vascular intestinal disorders	9.24%	9.24%	14.29%	14.29%	14.71%	14.71%
Gallbladder and biliary diseases	12.61%	12.61%	15.13%	15.13%	18.49%	18.49%
Pancreatitis	12.61%	12.61%	20.17%	20.17%	21.01%	21.01%
Other digestive diseases	0.00%	0.00%	0.00%	0.00%	0.00%	0.00%
Alzheimer's disease and other dementias	100.00%	100.00%	100.00%	100.00%	100.00%	100.00%
Parkinson's disease	11.76%	9.66%	100.00%	7.14%	100.00%	13.03%
Idiopathic epilepsy	100.00%	100.00%	24.79%	22.27%	100.00%	100.00%
Multiple sclerosis	14.29%	11.34%	14.29%	10.92%	18.91%	15.55%
Motor neuron disease	5.04%	4.62%	2.94%	2.94%	5.46%	5.04%
Migraine	25.21%	23.11%	14.29%	11.76%	34.03%	30.67%
Tension-type headache	19.33%	18.49%	9.24%	4.20%	26.47%	21.85%
Other neurological disorders	0.00%	0.00%	0.00%	0.00%	0.00%	0.00%
Schizophrenia	10.92%	6.72%	7.56%	4.62%	12.61%	7.98%
Major depressive disorder	100.00%	100.00%	27.73%	22.27%	100.00%	100.00%
Dysthymia	8.40%	5.04%	8.40%	5.88%	12.61%	8.82%
Bipolar disorder	100.00%	100.00%	100.00%	100.00%	100.00%	100.00%
Anxiety disorders	17.65%	13.45%	17.23%	12.18%	23.53%	18.91%
Anorexia nervosa	9.24%	1.26%	7.98%	1.68%	12.18%	2.52%
Bulimia nervosa	9.66%	3.78%	7.98%	2.52%	13.45%	5.46%
Autism spectrum disorders	14.71%	0.42%	10.08%	0.42%	19.33%	0.42%
Attention-deficit/hyperactivity disorder	100.00%	0.42%	12.18%	1.26%	100.00%	1.26%
Conduct disorder	100.00%	0.42%	5.04%	0.42%	100.00%	0.42%
Idiopathic developmental intellectual disability	0.00%	0.00%	0.00%	0.00%	0.00%	0.00%
Other mental disorders	0.84%	0.84%	0.42%	0.42%	0.84%	0.84%
Alcohol use disorders	18.07%	7.98%	17.65%	8.82%	22.69%	13.03%
Opioid use disorders	10.50%	1.68%	100.00%	100.00%	100.00%	100.00%
Cocaine use disorders	21.43%	6.30%	23.53%	2.10%	28.15%	7.56%
Amphetamine use disorders	16.81%	2.52%	20.17%	1.68%	23.95%	3.36%
Cannabis use disorders	32.35%	9.24%	45.38%	5.04%	50.00%	10.92%
Other drug use disorders	0.84%	0.84%	0.84%	0.84%	0.84%	0.84%
Diabetes mellitus type 1	27.73%	2.10%	13.45%	2.94%	28.99%	3.36%
Diabetes mellitus type 2	0.00%	0.00%	0.00%	0.00%	0.00%	0.00%
Chronic kidney disease due to diabetes mellitus type 1	5.46%	5.46%	10.08%	10.08%	10.92%	10.92%
Chronic kidney disease due to diabetes mellitus type 2	5.46%	5.46%	10.08%	10.08%	10.92%	10.92%
Chronic kidney disease due to hypertension	14.29%	13.87%	20.59%	20.59%	22.27%	22.27%
Chronic kidney disease due to glomerulonephritis	14.29%	13.87%	20.59%	20.59%	22.27%	22.27%
Chronic kidney disease due to other and unspecified causes	14.29%	13.87%	20.59%	20.59%	22.27%	22.27%
Acute glomerulonephritis	7.56%	7.56%	12.61%	12.61%	13.03%	13.03%
Atopic dermatitis	50.84%	2.52%	24.37%	4.20%	53.78%	5.46%
Contact dermatitis	2.94%	1.68%	4.20%	2.10%	5.46%	2.94%
Seborrhoeic dermatitis	3.78%	1.68%	6.30%	2.94%	8.40%	4.20%
Psoriasis	26.47%	13.03%	8.82%	6.72%	29.41%	15.97%
Cellulitis	9.24%	9.24%	14.71%	14.71%	15.13%	15.13%
Pyoderma	10.50%	8.82%	15.97%	13.87%	17.23%	14.71%

Supplementary Table 12. Data coverage index for all locations for 286 non-fatal causes, 1990–2005, 2005–2019 and in total by all ages and for the 70+ age group	1990-2005		2005-2019		1990-2019	
	All ages	70+	All ages	70+	All ages	70+
Scabies	8.40%	5.46%	9.24%	5.46%	12.18%	8.40%
Fungal skin diseases	6.72%	2.52%	7.56%	2.52%	10.08%	3.78%
Viral skin diseases	7.14%	2.52%	7.14%	3.78%	10.08%	5.88%
Acne vulgaris	5.88%	2.94%	9.24%	2.94%	11.76%	5.46%
Alopecia areata	2.10%	1.26%	5.04%	2.10%	5.46%	2.94%
Pruritus	2.52%	1.26%	3.36%	3.36%	5.04%	3.78%
Urticaria	5.46%	3.78%	5.04%	2.94%	8.40%	6.30%
Decubitus ulcer	9.24%	9.24%	14.29%	14.29%	14.71%	14.71%
Other skin and subcutaneous diseases	0.42%	0.00%	0.84%	0.42%	0.84%	0.42%
Glaucoma	12.18%	12.18%	28.15%	28.15%	32.35%	32.35%
Cataract	15.55%	15.13%	29.83%	29.83%	34.87%	34.87%
Age-related macular degeneration	10.50%	10.50%	27.73%	27.73%	31.09%	31.09%
Refraction disorders	26.47%	12.61%	27.73%	27.73%	40.76%	31.09%
Near vision loss	1.26%	1.26%	5.04%	4.62%	5.88%	5.46%
Other vision loss	8.40%	8.40%	22.69%	22.69%	26.05%	26.05%
Age-related and other hearing loss	10.08%	1.26%	2.94%	0.42%	11.34%	1.26%
Other sense organ diseases	0.42%	0.00%	0.84%	0.42%	0.84%	0.42%
Rheumatoid arthritis	9.66%	9.24%	9.24%	9.24%	14.29%	13.87%
Osteoarthritis hip	3.78%	3.78%	3.78%	3.78%	6.30%	6.30%
Osteoarthritis knee	2.94%	2.94%	5.88%	5.46%	7.56%	7.14%
Osteoarthritis hand	6.30%	2.94%	16.39%	5.04%	18.49%	6.72%
Osteoarthritis other	0.42%	0.00%	0.42%	0.00%	0.42%	0.00%
Low back pain	36.55%	32.35%	28.57%	22.69%	43.70%	40.34%
Neck pain	18.91%	18.91%	19.33%	19.33%	19.75%	19.75%
Gout	6.30%	6.30%	5.46%	5.46%	9.66%	9.66%
Other musculoskeletal disorders	4.62%	4.62%	4.20%	4.20%	6.72%	6.72%
Neural tube defects	100.00%	100.00%	100.00%	18.49%	100.00%	100.00%
Congenital heart anomalies	100.00%	100.00%	100.00%	15.55%	100.00%	100.00%
Orofacial clefts	25.21%	9.24%	31.93%	14.29%	35.29%	14.71%
Down syndrome	100.00%	100.00%	26.05%	3.78%	100.00%	100.00%
Turner syndrome	100.00%	100.00%	14.71%	10.92%	100.00%	100.00%
Klinefelter syndrome	100.00%	100.00%	14.29%	10.92%	100.00%	100.00%
Other chromosomal abnormalities	100.00%	100.00%	24.37%	16.81%	100.00%	100.00%
Congenital musculoskeletal and limb anomalies	25.63%	8.82%	31.51%	14.29%	35.29%	14.71%
Urogenital congenital anomalies	100.00%	8.40%	33.19%	13.45%	100.00%	13.87%
Digestive congenital anomalies	27.73%	9.24%	28.57%	13.87%	32.35%	14.29%
Other congenital birth defects	0.00%	0.00%	0.00%	0.00%	0.00%	0.00%
Urinary tract infections	9.24%	9.24%	14.71%	14.71%	15.13%	15.13%
Urolithiasis	9.24%	9.24%	15.13%	15.13%	15.55%	15.55%
Benign prostatic hyperplasia	9.24%	9.24%	15.13%	15.13%	15.55%	15.55%
Male infertility	36.97%	0.00%	26.47%	0.00%	42.02%	0.00%
Other urinary diseases	0.00%	0.00%	0.00%	0.00%	0.00%	0.00%
Uterine fibroids	10.08%	9.24%	17.23%	14.29%	18.07%	14.71%
Polycystic ovarian syndrome	5.04%	0.00%	16.81%	0.00%	17.23%	0.00%
Female infertility	36.97%	0.00%	26.47%	0.00%	42.02%	0.00%
Endometriosis	10.50%	0.00%	15.55%	0.00%	16.39%	0.00%
Genital prolapse	11.34%	10.50%	16.39%	15.97%	17.65%	16.39%
Premenstrual syndrome	5.04%	0.00%	5.04%	0.00%	7.98%	0.00%
Other gynecological diseases	9.24%	9.24%	15.13%	15.13%	15.55%	15.55%
Thalassemias	34.45%	2.52%	34.87%	5.46%	35.29%	5.46%
Thalassemias trait	0.00%	0.00%	0.00%	0.00%	0.00%	0.00%

Supplementary Table 12: Data coverage index for all locations for 286 non-fatal causes, 1990–2005, 2005–2019 and in total by all ages and for the 70+ age group						
	1990-2005		2005-2019		1990-2019	
	All ages	70+	All ages	70+	All ages	70+
Sickle cell disorders	40.34%	9.66%	44.96%	15.13%	45.38%	16.39%
Sickle cell trait	0.00%	0.00%	0.00%	0.00%	0.00%	0.00%
G6PD deficiency	100.00%	13.87%	100.00%	9.24%	100.00%	17.23%
G6PD trait	0.00%	0.00%	0.00%	0.00%	0.00%	0.00%
Other hemoglobinopathies and hemolytic anemias	0.00%	0.00%	0.00%	0.00%	0.00%	0.00%
Endocrine, metabolic, blood, and immune disorders	9.24%	9.24%	15.13%	15.13%	15.55%	15.55%
Caries of deciduous teeth	23.11%	0.00%	23.95%	0.00%	32.77%	0.00%
Caries of permanent teeth	24.79%	5.04%	25.63%	5.88%	34.45%	8.82%
Periodontal diseases	14.29%	7.56%	6.72%	5.04%	16.81%	10.92%
Edentulism and severe tooth loss	33.19%	32.35%	23.53%	22.69%	37.82%	37.39%
Other oral disorders	0.42%	0.00%	0.42%	0.00%	0.42%	0.00%
Pedestrian road injuries	3.78%	3.36%	7.14%	5.88%	7.14%	6.30%
Cyclist road injuries	3.78%	3.36%	6.72%	5.88%	7.14%	6.30%
Motorcyclist road injuries	3.36%	3.36%	6.72%	5.88%	7.14%	6.30%
Motor vehicle road injuries	3.78%	3.36%	6.72%	5.88%	7.14%	6.30%
Other road injuries	3.36%	3.36%	5.04%	5.04%	5.46%	5.46%
Other transport injuries	3.78%	3.78%	5.88%	5.88%	6.30%	6.30%
Falls	9.24%	7.98%	11.34%	10.08%	14.29%	12.61%
Drowning	9.24%	8.40%	13.87%	13.45%	14.71%	14.29%
Fire, heat, and hot substances	7.14%	6.30%	10.50%	9.24%	12.61%	11.34%
Poisoning by carbon monoxide	3.36%	3.36%	5.46%	5.46%	5.88%	5.88%
Poisoning by other means	3.78%	3.78%	5.88%	5.88%	6.30%	6.30%
Unintentional firearm injuries	3.78%	3.78%	5.46%	5.46%	5.88%	5.88%
Other exposure to mechanical forces	3.78%	3.78%	6.30%	6.30%	6.72%	6.72%
Adverse effects of medical treatment	8.82%	8.82%	14.29%	14.29%	14.71%	14.71%
Venomous animal contact	3.78%	3.78%	6.30%	6.30%	6.72%	6.72%
Non-venomous animal contact	3.78%	3.78%	5.88%	5.88%	6.30%	6.30%
Pulmonary aspiration and foreign body in airway	4.20%	4.20%	6.30%	6.30%	6.72%	6.72%
Foreign body in eyes	4.20%	4.20%	6.30%	6.30%	6.30%	6.30%
Foreign body in other body part	4.62%	4.62%	7.14%	7.14%	7.56%	7.56%
Environmental heat and cold exposure	4.20%	3.78%	5.88%	5.88%	6.72%	6.30%
Exposure to forces of nature	0.00%	0.00%	0.00%	0.00%	0.00%	0.00%
Other unintentional injuries	3.78%	3.78%	5.88%	5.88%	6.72%	6.72%
Self-harm by firearm	6.30%	6.30%	9.66%	9.24%	9.66%	9.24%
Self-harm by other specified means	3.36%	3.36%	5.88%	5.46%	6.30%	5.88%
Physical violence by firearm	7.98%	7.98%	10.50%	10.50%	11.76%	11.76%
Physical violence by sharp object	4.20%	4.20%	8.82%	8.82%	9.24%	9.24%
Sexual violence	9.24%	0.42%	26.05%	1.68%	28.57%	1.68%
Physical violence by other means	3.78%	3.78%	6.30%	6.30%	6.72%	6.72%
Conflict and terrorism	0.00%	0.00%	0.00%	0.00%	0.00%	0.00%
Executions and police conflict	0.00%	0.00%	0.00%	0.00%	0.00%	0.00%
