# Supplementary material for: Global, regional, and national burden of diseases and injuries for adults 70 years and older: systematic analysis for the Global Burden of Disease 2019 Study
Source: BMJ. 2022 Mar 10;376:e068208. doi: 10.1136/bmj-2021-068208 (PMC9316948; doi:10.1136/bmj-2021-068208)
Supplement: Supplementary file 2 — Web appendix: Acknowledgments and declarations [file tyrs068208.ww2.pdf]

Declarations

Dr. Chatterji reports a disclaimer that the views expressed in this paper are those of the authors, and do not necessarily represent the views or policies of the World Health Organization. Dr. Ärnlöv reports personal fees from AstraZeneca, outside the submitted work. Dr. Ancuceanu reports consultancy and speakers' fees from various pharmaceutical companies. Dr. Antonio reports personal fees from Johnson & Johnson (Philippines), Inc., outside the submitted work. Dr. Béjot reports personal fees from AstraZeneca, personal fees from BMS, personal fees from Pfizer, personal fees from Medtronic, grants and personal fees from Boehringer-Ingelheim, personal fees from MSD, personal fees and non-financial support from Servier, personal fees from Amgen, and non-financial support from Biogen, outside the submitted work. Dr. Bell reports grants from the U.S. Environmental Protection Agency, grants from National Institutes of Health (NIH), grants from Wellcome Trust Foundation, grants from Yale University, during the conduct of the study; and Honorarium and/or travel reimbursement: NIH (for review of grant proposals) American Journal of Public Health (participation as editor), Global Research Laboratory and Seoul National University, Royal Society, London UK, Ohio University, Atmospheric Chemistry Gordon Research Conference, Johns Hopkins Bloomberg School of Public Health, Arizona State University, Ministry of the Environment, Japan, Hong Kong Polytechnic University, University of Illinois – Champaign, University of Tennessee – Knoxville, and University of Montana. Dr. Beghi reports grants from Italian Ministry of Health, grants from SOBI, personal fees from Arvelle Therapeutics, and grants from the American ALS Association, outside the submitted work. Dr. Briggs reports personal fees from the World Health Organization, outside the submitted work. Dr. Ivers reports grants from the National Health and Medical Research Council of Australia, during the conduct of the study. Dr. Jozwiak reports personal fees from AMGEN, personal fees from ALAB LABORATORIES, personal fees from TEVA, personal fees from BOEHRINGER INGELHEIM, personal fees from SYNEXUS, outside the submitted work. Dr. Kivimaki reports grants from the Medical Research Council (MR/R024227/1), during the conduct of the study. Dr. Krishan reports grants from the UGC Center of Advanced Study (CAS II), Department of Anthropology, Panjab University, Chandigarh, India, outside the submitted work. Dr. Lorkowski reports personal fees from AMGEN, personal fees from Berlin-Chemie, personal fees from MSD Sharp & Dohme, personal fees from Novo Nordisk, non-financial support from Preventicus, personal fees from Sanofi-Aventis, personal fees from Synlab, personal fees from Unilever, personal fees from Upfield, personal fees from Akcea Therapeutics, personal fees from Amedes, personal fees from Boehringer Ingelheim Pharma, and personal fees from Daiichi Sankyo, outside the submitted work. Dr. Massano reports personal fees from Abbvie, personal fees from Bial, personal fees from Boston Scientific, speaker honoraria from GE Healthcare, advisor honoraria from Merck Sharp & Dohme, financial support to attend scientific meetings from Medtronic, advisor honoraria from Zambon, and financial support to attend specific meetings from Roche, outside the submitted work. Dr. Mendoza is a Program Analyst in Population and Development at the United Nations Population Fund-UNFPA Country Office in Peru, institution which not necessarily endorses this study. Dr. Mitchell reports personal fees from Sanofi (Hangzhou), outside the submitted work. Dr. Nomura reports grants from the Ministry of Education, Culture, Sports, Science, and Technology. Dr. Norrving reports personal fees from Astra Zeneca, and personal fees from Bayer, outside the submitted work. Dr. Pearson-Stuttard reports personal fees from Novo Nordisk A/S and Lane Clark & Peacock outside of the submitted work. Dr. Pilgrim reports grants and personal fees from Boston Scientific, grants and personal fees from Biotronik, and personal fees from Highlife SAS, outside the submitted work. Dr. Postma reports grants and personal fees from MSD, grants and personal fees from GSK, grants and personal fees from Pfizer, grants and personal fees from Boehringer Ingelheim, grants and personal fees from Novavax, personal fees from Quintiles, grants from Bayer, grants and personal fees from BMS, grants and personal fees from Astra Zeneca, grants and personal fees from Sanofi, personal fees from Novartis, personal fees from Pharmerit, stock in Health-Ecore, stock in PAG Ltd, advising services to Asc Academics, grants and personal fees from IQVIA, grants from BioMerieux, grants from WHO, grants from EU, grants and personal fees from Seqirus, grants from FIND, grants from

Antilope, grants from WHO, and grants from DIKTI, LPDP, Budi, outside the submitted work. Dr. Schutte reports personal fees from Takeda, personal fees from Servier, personal fees from Novartis, and personal fees from Omron Healthcare, outside the submitted work. Dr. J. Singh reports personal fees from Crealta/Horizon, Medisys, Fidia, UBM LLC, Trio health, Medscape, WebMD, Clinical Care options, Clearview healthcare partners, Putnam associates, Focus forward, Navigant consulting, Spherix, Practice Point communications, the National Institutes of Health and the American College of Rheumatology, personal fees from Simply Speaking, previous stock in Amarin pharmaceuticals and Viking pharmaceuticals, non-financial support from Steering committee of OMERACT, an international organization that develops measures for clinical trials and receives arm's length funding from 12 pharmaceutical companies, non-financial support from Veterans Affairs Rheumatology Field Advisory Committee, and non-financial support from Editor and the Director of the UAB Cochrane Musculoskeletal Group Satellite Center on Network Meta-analysis, outside the submitted work. Dr. Skou reports personal fees from Board membership: Journal of Orthopaedic & Sports Physical Therapy, grants from The Lundbeck Foundation, personal fees from Munksgaard, outside the submitted work; and is co-founder of GLA:D. Dr. Stein reports personal fees from Lundbeck, and personal fees from Sun, outside the submitted work. Dr. Uddin worked as a visiting fellow at Deakin University Institute for Physical Activity and Nutrition (IPAN) with paid travel (including flights and transport, accommodation, and meals), outside the submitted work.

Acknowledgements

Research reported in this publication was supported by the National Institute on Aging of the National Institutes of Health under Award Number P30AG047845. The content is solely the responsibility of the authors and does not necessarily represent the official views of the National Institutes of Health. Lucas Guimarães Abreu would like to acknowledge support from Coordenação de Aperfeiçoamento de Pessoal de Nível Superior (CAPES), Brasil - Finance Code 001, Conselho Nacional de Desenvolvimento Científico e Tecnológico (CNPq) (404710/2018-2 and 310797/2019-5) and Fundação de Amparo à Pesquisa do Estado de Minas Gerais (FAPEMIG). Olatunji O Adetokunboh would like to acknowledge South African Department of Science and Innovation, National Research Foundation and DSI/NRF Centre of Excellence for Epidemiological Modelling and Analysis. Anurag Agrawal acknowledges Wellcome Trust DBT India Alliance IA/CPHS/14/1/501489. Syed Mohamed Aljunid would like to acknowledge the Department of Health Policy and Management, Faculty of Public Health, Kuwait University and International Centre for Casemix and Clinical Coding, Faculty of Medicine, National University of Malaysia for the approval and support to participate in this research project. Marcel Ausloos is partially supported by a grant of the Romanian National Authority for Scientific Research and Innovation, CNDS-UEFISCDI, project number PN-III-P4-ID-PCCF-2016-0084. Gabrielle B Britton is supported by Sistema Nacional de Investigación (SNI) of the Secretaría Nacional de Ciencia, Tecnología e Innovación (SENACYT) of Panamá. Josip Car's post at Imperial College London is supported by the NIHR NW London Applied Research Collaboration. Juan Jesus Carrero is funded by the Swedish Research Council (2019-01059). Felix Carvalho acknowledges UID/MULTI/04378/2019 and UID/QUI/50006/2019 support with funding from FCT/MCTES through national funds. Deborah Carvalho Malta acknowledges Conselho Nacional de Desenvolvimento Científico e Tecnológico (CNPq Brazil). Vera Marisa Costa acknowledges her grant (SFRH/BHD/110001/2015), received by Portuguese national funds through Fundação para a Ciência e Tecnologia (FCT), IP, under the Norma Transitória DL57/2016/CP1334/CT0006. Khalil Eskandari is thankful to Kerman University of Medical Sciences for the partial supporting of the work. Manuela L Ferreira holds a National Health and Medical Research Council of Australia Fellowship. Claudiu Herteliu is partially supported by a grant of the Romanian National Authority for Scientific Research and Innovation, CNDS-UEFISCDI, project number PN-III-P4-ID-PCCF-2016-0084, partially supported by a grant co-funded by European Fund for Regional Development through Operational Program for Competitiveness, project ID P_40_382, and partially

supported by a grant of the Romanian National Authority for Scientific Research and Innovation, CNDS-UEFISCDI, project number PN-III-P2-2.1-SOL-2020-2-0351. Praveen Hoogar acknowledges the Centre for Bio Cultural Studies(CBiCS), Directorate of Research, Manipal Academy of Higher Education, Manipal. Mihajlo Jakovljevic acknowledges that the Serbian part of this GBD Project related contribution was co-funded through Grant OI175014 by the Ministry of Education Science and Technological Development of the Republic of Serbia. Oommen John is recipient of an UIPA scholarship from UNSW, Sydney. Yun Jin Kim acknowledges support from Research Management Centre, Xiamen University Malaysia [No.: XMUMRF/2020-C6/ITCM/0004]. Mika Kivimäki reports grants from the Medical Research Council (MR/R024227/1), during the conduct of the study. Kewal Krishan is supported by a DST PURSE grant and UGC Centre of Advanced Study (CAS II) awarded to the Department of Anthropology, Panjab University, Chandigarh, India. Manasi Kumar acknowledges K43 TW010716-03 FIC/NIH. Ben Lacey acknowledges support from the NIHR Oxford Biomedical Research Centre and the BHF Centre of Research Excellence, Oxford. Tea Lallukka is supported by the Academy of Finland (Grant #330527). Iván Landires is member of the Sistema Nacional de Investigación (SNI), supported by the Secretaría Nacional de Ciencia, Tecnología e Innovación (SENACYT), Panama. Stefan Lorkowski acknowledges institutional support from the Competence Cluster for Nutrition and Cardiovascular Health (nutriCARD) Halle-Jena-Leipzig (Germany; German Federal Ministry of Education and Research; grant agreement number 01EA1808A). Phetole Walter Mahasha would like to acknowledge SAMRC, Grants,Innovation and Product Development Unit. John J McGrath is supported by the Danish National Research Foundation (Niels Bohr Professorship). Research funding by Australian National Health and Medical Research Council (Investigator Grant). Stefania Mondello is supported by the Italian Ministry of Health (grant number GR-2013-02354960). Ulrich Otto Mueller acknowledges funding by the german National Cohort Study BMBF grant #01ER1801D. Subas Neupane was partly supported by the Competitive State Research Financing of the Expert Responsibility area of Tampere University Hospital. Shuhei Nomura acknowledges the Ministry of Education, Culture, Sports, Science, and Technology of Japan (18K10082). Michael R Phillips was supported in part by the National Natural Science Foundation of China (NSFC, No. 81761128031). Alberto Raggi, Davide Sattin and Silvia Schiavolin acknowledge support by a grant from the Italian Ministry of Health (Ricerca Corrente, Fondazione Istituto Neurologico C. Besta, Linea 4—Outcome Research: dagli Indicatori alle Raccomandazioni Cliniche). Ana Isabel Ribeiro was supported by National Funds through FCT, under the programme of 'Stimulus of Scientific Employment – Individual Support' within the contract CEECIND/02386/2018. Perminder Sachdev acknowledges the funding support of the National Health and Medical Research Council of Australia. Abdallah M Samy was supported by a fellowship from the Egyptian Fulbright Mission Program. Milena Santric-Milicevic acknowledges a support of the Ministry of Education, Science and Technological Development of the Republic of Serbia (Contract No. 175087). João Pedro Silva acknowledges support from grant number UIDB/04378/2020 from the Applied Molecular Biosciences Unit (UCIBIO), supported through Portuguese national funds via FCT/MCTES. Søren T Skou is currently funded by a grant from the European Research Council (ERC) under the European Union's Horizon 2020 research and innovation program (grant agreement No 801790) and a program grant from Region Zealand (Exercise First). Mohammad Reza Sobhiyeh acknowledges the Clinical Research Development Center of Imam Reza Hospital Kermanshah University of Medical Sciences for their wise advice. Joan B Soriano acknowledges the Centro de Investigación Biomédica en Red Enfermedades Respiratorias (CIBERES, Center for Biomedical Research in Respiratory Diseases Network) at the Instituto de Salud Carlos III (ISCIII, Carlos III Health Institute). Rafael Tabarés-Seisdedos was supported in part by the national grant PI17/00719 from ISCIII-FEDER. Riaz Uddin is supported by Alfred Deakin Post-Doctoral Research Fellowship. Sojib Bin Zaman is a recipient of the Australian Government research training program (RTP) scholarship from Monash University.
